# A deep-learning algorithm to classify skin lesions from mpox virus infection

**DOI:** 10.1038/s41591-023-02225-7

**Published:** 2023-03-02

**Authors:** Alexander H. Thieme, Yuanning Zheng, Gautam Machiraju, Chris Sadee, Mirja Mittermaier, Maximilian Gertler, Jorge L. Salinas, Krithika Srinivasan, Prashnna Gyawali, Francisco Carrillo-Perez, Angelo Capodici, Maximilian Uhlig, Daniel Habenicht, Anastassia Löser, Maja Kohler, Maximilian Schuessler, David Kaul, Johannes Gollrad, Jackie Ma, Christoph Lippert, Kendall Billick, Isaac Bogoch, Tina Hernandez-Boussard, Pascal Geldsetzer, Olivier Gevaert

**Affiliations:** 1 Department of Medicine, Stanford University, Stanford, CA USA; 2 Stanford Center for Biomedical Informatics Research (BMIR), Department of Biomedical Data Science, Stanford University, Stanford, USA; 3 Department of Radiation Oncology, Charité—Universitätsmedizin Berlin, Berlin, Germany; 4 Berlin Institute of Health at Charité—Universitätsmedizin Berlin, BIH Biomedical Innovation Academy, BIH Charité Digital Clinician Scientist Program, Berlin, Berlin Germany; 5 Department of Biomedical Data Science, Stanford University, Stanford, CA USA; 6 Department of Infectious Diseases and Respiratory Medicine, Charité—Universitätsmedizin Berlin, Berlin, Germany; 7 Institute of Tropical Medicine and International Health, Charité—Universitätsmedizin Berlin, Berlin, Germany; 8 Division of Infectious Diseases and Geographic Medicine, Department of Medicine, Stanford University, Stanford, CA USA; 9 Department of Architecture and Computer Technology (ATC), University of Granada, Granada, Spain; 10 Department of Biomedical and Neuromotor Science, Alma Mater Studiorum–University of Bologna, Bologna, Italy; 11 Department of Medicine, Justus-Liebig-Universität Gießen, Gießen, Germany; 12 Technical University Berlin, Berlin, Germany; 13 Department of Radiotherapy, University Medical Center Schleswig-Holstein, Lübeck, Germany; 14 Heidelberg Institute of Global Health, Heidelberg University Hospital, Heidelberg, Germany; 15 University Basel, Department of Psychology, Center for Cognitive and Decision Sciences, Basel, Switzerland; 16 Department of Artificial Intelligence, Fraunhofer Heinrich Hertz Institute, Berlin, Germany; 17 Digital Health & Machine Learning, Hasso Plattner Institute, University of Potsdam, Potsdam, Germany; 18 Hasso Plattner Institute for Digital Health at Mount Sinai, Icahn School of Medicine at Mount Sinai, New York, NY USA; 19 Division of Dermatology, Toronto Western Hospital, University Health Network, Toronto, Ontario Canada; 20 Division of Infectious Diseases, Toronto General Hospital, University Health Network, Toronto, Ontario Canada; 21 Department of Surgery, Stanford University, Stanford, CA USA; 22 Division of Primary Care and Population Health, Department of Medicine, Stanford University, Stanford, CA USA; 23 Chan Zuckerberg Biohub, San Francisco, CA USA

**Keywords:** Viral infection, Public health

## Abstract

Undetected infection and delayed isolation of infected individuals are key factors driving the monkeypox virus (now termed mpox virus or MPXV) outbreak. To enable earlier detection of MPXV infection, we developed an image-based deep convolutional neural network (named MPXV-CNN) for the identification of the characteristic skin lesions caused by MPXV. We assembled a dataset of 139,198 skin lesion images, split into training/validation and testing cohorts, comprising non-MPXV images (*n* = 138,522) from eight dermatological repositories and MPXV images (*n* = 676) from the scientific literature, news articles, social media and a prospective cohort of the Stanford University Medical Center (*n* = 63 images from 12 patients, all male). In the validation and testing cohorts, the sensitivity of the MPXV-CNN was 0.83 and 0.91, the specificity was 0.965 and 0.898 and the area under the curve was 0.967 and 0.966, respectively. In the prospective cohort, the sensitivity was 0.89. The classification performance of the MPXV-CNN was robust across various skin tones and body regions. To facilitate the usage of the algorithm, we developed a web-based app by which the MPXV-CNN can be accessed for patient guidance. The capability of the MPXV-CNN for identifying MPXV lesions has the potential to aid in MPXV outbreak mitigation.

## Abstract (web summary)

A deep-learning algorithm was developed to identify skin lesions caused by the mpox virus and was then implemented in a web-based app designed for patient use.

## Main

The monkeypox virus (now termed mpox virus or MPXV), a double-stranded DNA virus belonging to the Orthopoxvirus genus and causative agent of a zoonotic disease, has caused an ongoing outbreak with more than 28,700 confirmed cases in 93 countries as of 5 August 2022. The World Health Organization (WHO) has declared this outbreak a Public Health Emergency of International Concern^1^. Animal-to-human transmission was generally assumed and confirmed in numerous recent MPXV outbreaks. Sustained human-to-human transmission was considered limited as infection chains in the human populations were short in endemic regions of Central and West Africa^2^. This outbreak showed for the first time sustained human-to-human community transmission in nonendemic countries^3^. Cases were reported primarily in men who have sex with men and in some cases in women and children^4–9^.

Modeling by the European Centre for Disease Prevention and Control identified undetected infections and delayed isolation as key parameters that drive MPXV outbreaks^10^. With WHO case definitions^11^, a significant proportion of infections remained undetected^5^ such as a person with a characteristic vesicular-pustular rash without a history of contact with a confirmed infection. Therefore, multiple authors have suggested a review and broadening of case definitions^5,12^. Artificial intelligence (AI)-assisted case definitions have not been explored so far but could represent a solution.

Deep convolutional neural networks (CNN) have shown promise in classifying skin lesions in dermatology^13–20^ with some authors reporting above expert-level accuracy^14^. In recent studies, the majority of MPXV infections (up to 95.2%) were associated with skin lesions^4,5,21^ which appear in different stages over the course of the disease. Informing individuals who are worried about having been infected with MPXV as to whether their skin lesions likely stems from an MPXV infection or not could accelerate appropriate care-seeking and improve the adoption of behaviors to reduce onward transmission. This could be accomplished through the integration of an image-based CNN into an app that allows users to analyze an image of their skin lesion.

The aim of this study was, therefore, to develop and evaluate the performance of a CNN for the detection of MPXV skin lesions (MPXV-CNN) in photographic images and to integrate the MPXV-CNN into an app. To identify biases and weaknesses, we evaluated the performance of the MPXV-CNN in multiple large image datasets for different skin tones^20^ and locations of the skin lesion. We also specifically evaluated the performance of the model in classifying MPXV skin lesions versus other acute skin diseases and differential diagnoses with skin lesions of similar appearance, including varicella, drug-induced allergies, impetigo, measles, molluscum contagiosum, orf, scabies and syphilis^22^.

## Results

### Sample characteristics

**Fig. 1:**
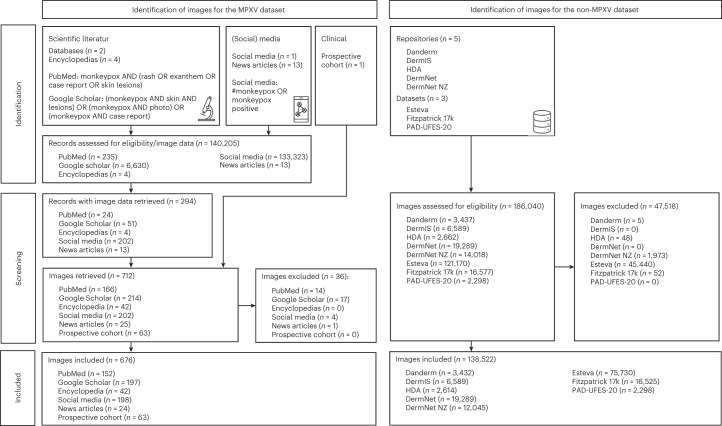
Flow diagram for the MPXV and non-MPXV image datasets. The flow diagram showed the identification and screening procedures of images to create the MPXV and non-MPXV datasets. MPXV images were collected from publications of the scientific literature, encyclopedia articles, new articles, social media and a prospective cohort of patients from the Stanford University Medical Center, while non-MPXV images originated from eight repositories and datasets.

The image characteristics were summarized in Table 1. We constructed a new dataset of photographic images of skin diseases (*n* = 139,198) originating from multiple publicly available sources and institutional data as follows: 676 images of MPXV skin lesions (MPXV dataset) aggregated from publications of the scientific literature, encyclopedia articles, news articles, social media and prospectively collected MPXV skin lesion images of patients of the Stanford University Medical Center (prospective cohort) and 138,522 images of non-MPXV skin lesions (non-MPXV dataset) from five public dermatological repositories (Danderm, DermIS, Hellenic Dermatological Atlas (HDA), DermNet, DermNet NZ), two public datasets (PAD-UFES-20 (ref. ^23^), Fitzpatrick 17k^24^) and one institutional dataset (Esteva^13^). Image screening and filtering were performed as described in Fig. 1 and Methods. The following metadata was made available per image: diagnoses for Danderm, DermIS, HDA, DermNet, DermNet NZ, PAD-UFES-20, Fitzpatrick 17k, Esteva and the prospective cohort; skin tone for PAD-UFES-20, Fitzpatrick 17k and the prospective cohort; body region for DermIS and the prospective cohort; age group for DermIS, PAD-UFES-20 and the prospective cohort; sex for DermIS, PAD-UFES-20 and the prospective cohort. We mapped diagnoses of all non-MPXV sources to a uniform taxonomy of 2,013 skin diagnoses previously developed at our institute^13^. Uniform diagnoses could be associated with 94.5% (130,852 of 138,522) of skin lesion images in the non-MPXV dataset. All evaluations on non-MPXV diagnoses were pooled analyses on the entire non-MPXV dataset. Frequency tables for uniform diagnoses in the training and testing non-MPXV datasets are collated in Supplementary Tables 1–11.

**Table 1:** Number of skin lesion images per category and per data source in the MPXV and non-MPXV datasets used for training and testing the MPXV-CNN

	MPXV dataset						Non-MPXV dataset								
Category	Publications (*n* = 75)	Encyclopedia (*n* = 4)	News articles (*n* = 13)	Social media (*n* = 1)	Prospective cohort	Total	Danderm	DermIS	HDA	Fitzpatrick 17k	DermNet	DermNet NZ	PAD-UFES-20	Esteva	Total
All	380	42	25	202	63	712	3,437	6,589	2,662	16,577	19,289	14,018	2,298	121,170	186,040
Excluded	31	0	1	4	0	36	5	0	48	52	0	1,973	0	45,440	47,518
Included	349	42	24	198	63	676	3,432	6,589	2,614	16,525	19,289	12,045	2,298	75,730	138,522
Training	254	42	24	198	0	518	0	0	0	0	0	12,045	0	0	12,045
Testing	95	0	0	0	63	158	3,432	6,589	2,614	16,525	19,289	0	2,298	75,730	126,477
Age															
Child (<18 years)	35	6	8	7	0	56	–	979	–	–	–	^a^	39	–	1,018
Adult (≥18 years)	292	32	11	183	63	581	–	2,557	–	–	–	^a^	2,259	–	4,816
Unknown	22	4	5	8	0	39	3,432	3,053	2,614	16,525	19,289	12,045	0	75,730	132,688
Sex															
Male	277	22	12	184	63	558	–	2,593	–	–	–	^b^	741	–	3,334
Female	19	2	1	4	0	26	–	2,520	–	–	–	^b^	753	–	3,273
Unknown	53	18	11	10	0	92	3,432	1,476	2,614	16,525	19,289	12,045	804	75,730	131,915
Skin tone (Fitzpatrick type)															
I	7	0	1	19	0	27	–	–	–	2,941	–	–	153	–	3,094
II	87	16	6	72	26	207	–	–	–	4,796	–	–	876	–	5,672
III	115	0	5	49	27	196	–	–	–	3,296	–	–	392	–	3,688
IV	32	22	3	24	0	81	–	–	–	2,775	–	–	62	–	2,837
V	30	0	0	27	10	67	–	–	–	1,527	–	–	10	–	1,537
VI	78	4	9	7	0	98	–	–	–	628	–	–	1	–	629
Unknown	0	0	0	0	0	0	3,432	6,589	2,614	562	19,289	12,045	804	75,730	121,065
Region of body															
Head	55	11	1	56	2	125	–	1,443	–	–	–	–	–	–	1,443
Neck	2	0	0	0	1	3	–	96	–	–	–	–	–	–	96
Torso	50	12	3	16	8	89	–	705	–	–	–	–	–	–	705
Upper extremity	62	9	5	59	26	161	–	916	–	–	–	–	–	–	916
Lower extremity	33	1	2	12	12	60	–	813	–	–	–	–	–	–	813
Anogenital	103	4	0	35	9	151	–	223	–	–	–	–	–	–	223
Anal	16	0	0	12	0	28	–	5	–	–	–	–	–	–	5
Perianal	10	0	0	1	3	14	–	18	–	–	–	–	–	–	18
Genital	77	4	0	22	6	109	–	106	–	–	–	–	–	–	106
Unknown	0	0	0	0	0	0	–	94	–	–	–	–	–	–	94
Multiple body regions	27	1	9	11	4	52	–	110	–	–	–	–	–	–	110
Unknown or zoomed in	17	4	4	9	1	35	3,432	2,283	2,614	16,525	19,289	12,045	2,298	75,730	134,216
Origin^c^															
Europe	110	0	0	65	0	175	^c^	^c^	^c^	–	–	^c^	–	–	–
Africa	70	0	5	1	0	76	–	–	–	–	–	^c^	–	–	–
Asia	6	0	3	1	0	10	–	–	–	^c^	–	–	–	–	–
South America	7	0	0	28	0	35	–	–	–	^c^	–	–	–	–	–
North America	41	0	6	92	63	202	–	–	–	–	–	^c^	–	–	–
Antarctica	0	0	0	0	0	0	–	–	–	–	–	–	–	–	–
Australia	3	0	0	0	0	3	–	–	–	–	–	^c^	–	–	–
Unknown	112	42	10	11	0	175	3,432	6,589	2,614	16,525	19,289	12,045	2,298	75,730	138,522
Lesions (*N)*															
*N* = 0 (rash)	5	0	0	4	0	9	–	–	–	–	–	–	–	–	–
*N* = 1	118	16	6	87	30	257	–	–	–	–	–	–	–	–	–
*N* = 2	38	10	2	42	20	112	–	–	–	–	–	–	–	–	–
*N* = 3	26	6	0	18	3	53	–	–	–	–	–	–	–	–	–
4 ≤ *N* ≤ 5	16	3	0	13	5	37	–	–	–	–	–	–	–	–	–
6 ≤ *N* ≤ 10	30	3	1	12	4	50	–	–	–	–	–	–	–	–	–
*N* > 10	116	4	15	21	1	157	–	–	–	–	–	–	–	–	–
Unknown	0	0	0	1	0	1	3,432	6,589	2,614	16,525	19,289	12,045	2,298	75,730	138,522
Duration of presence															
<7 d	49	6	0	12	0	67	–	–	–	–	–	–	–	–	–
≥7 d	80	0	1	43	3	127	–	–	–	–	–	–	–	–	–
Unknown	220	36	23	143	60	482	3,432	6,589	2,614	16,525	19,289	12,045	2,298	75,730	138,522
Coalesced lesions															
Yes	132	11	14	27	2	186	N/A	N/A	N/A	N/A	N/A	N/A	N/A	N/A	N/A
No	212	31	10	167	61	481	N/A	N/A	N/A	N/A	N/A	N/A	N/A	N/A	N/A
N/A	5	0	0	4	0	9	N/A	N/A	N/A	N/A	N/A	N/A	N/A	N/A	N/A
2022 MPXV outbreak associated															
Yes	264	26	24	198	63	575	N/A	N/A	N/A	N/A	N/A	N/A	N/A	N/A	N/A
No	85	16	0	0	0	101	N/A	N/A	N/A	N/A	N/A	N/A	N/A	N/A	N/A
MPXV clade															
Clade 1	38	0	0	0	0	38	N/A	N/A	N/A	N/A	N/A	N/A	N/A	N/A	N/A
Clade 2	303	26	21	198	63	611	N/A	N/A	N/A	N/A	N/A	N/A	N/A	N/A	N/A
Unknown	8	16	3	0	0	27	N/A	N/A	N/A	N/A	N/A	N/A	N/A	N/A	N/A

^a^No classification per image available, but the database owners reported the following ratios: child 14% and adult 86%.

^b^No classification per image available, but the database owners reported the following ratios: 48% male and 52% female.

^c^No classification per image is available for non-MPXV repositories and datasets, however the origin of most images can be assigned to the following continents: Danderm—Europe, DermIS—Europe, HDA—Europe, Fitzpatrick 17k—South America and Asia, DermNet—unknown, DermNet NZ—Europe, Africa, North America, Australia, PAD—unknown.

All, number of all available skin lesion images; excluded, number of excluded images; included, number of images included in this study; N/A, not applicable; training, number of images used for training the MPXV-CNN; testing, number of images used for testing the MPXV-CNN;–, not available.

### Algorithm performance in the training cohort

**Fig. 2:**
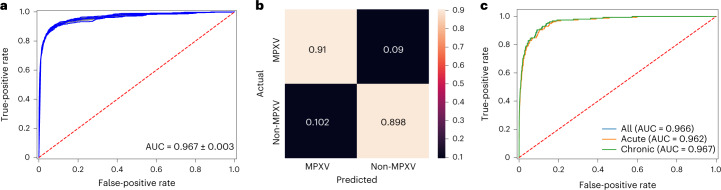
Performance diagrams of the MPXV-CNN for the validation and testing cohorts. **a**, ROC curve derived from repeated fivefold cross-validation on the validation cohort (AUC = 0.967 ± 0.003). **b**, Confusion matrix on the testing cohort showing the ratios of TPs (0.91), TNs (0.898), FPs (0.102) and FNs (0.09). **c**, ROC curve of the testing cohort that included MPXV skin lesions and either acute non-MPXV skin lesions (AUC = 0.962), chronic non-MPXV skin lesions (AUC = 0.967) or all non-MPXV skin lesions (AUC = 0.966). FPs, false positives; FNs, false negatives; ROC, receiver operating characteristic; TPs, true positives; TNs, true negatives.

We used images of MPXV skin lesions (*n* = 518) and non-MPXV skin lesions (*n* = 12,045) for the training and validation of the MPXV-CNN (Methods: Data splitting). We performed stratified fivefold cross-validation, wherein in each fold, images from 80% of patients were used for training and 20% for validation. The cross-validation was repeated five times. In the validation dataset, the sensitivity was 0.83 (s.d.: 0.01), specificity was 0.965 (s.d.: 0.002) and the area under curve (AUC) was 0.967 (s.d.: 0.003; Fig. 2a). Performance results for other architectures than ResNet34 can be found in Supplementary Table 12.

### Algorithm performance in the testing cohort

**Extended Data Fig. 1:**
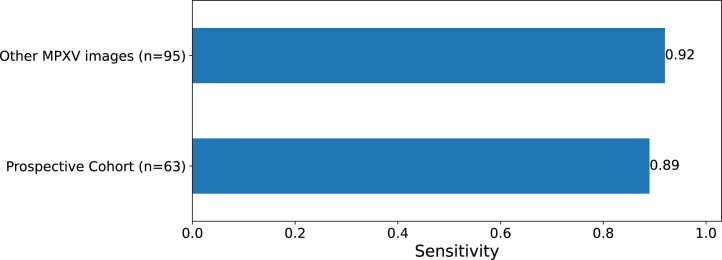
Subgroup analysis of the sensitivity in the testing cohort. The observed sensitivity was high in the prospective cohort (0.89) with patients from the Stanford University Medical Center and in other MPXV images (0.92). MPXV, mpox virus; n, Number of available images per testing cohort.

**Extended Data Fig. 2:**
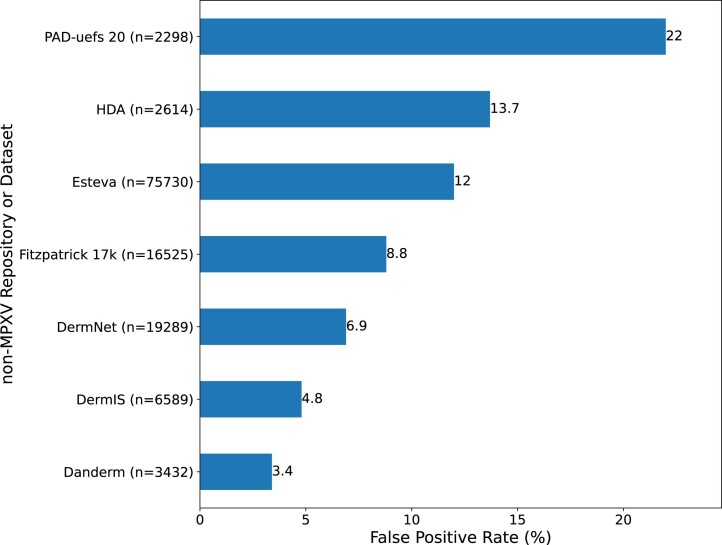
False Positive Rates in 7 non-MPXV image repositories and datasets of the testing cohort. n, Number of available images per image repository.

After we evaluated the MPXV-CNN using cross-validation, we trained a final model on images (*n* = 12,563) from the entire training cohort. The final model was evaluated using images from an external testing cohort (Methods: Data splitting). The testing cohort contained 158 MPXV images and 126,477 non-MPXV images. Sensitivity was 0.91, specificity 0.898 (Fig. 2b) and the AUC 0.966 (Fig. 2c). Specifically, sensitivity was 0.89 in MPXV skin lesion images prospectively collected from patients (*n* = 63 images from 12 patients, all male) of the Stanford University Medical Center and 0.92 in other MPXV skin lesion images (Extended Data Fig. 1). The false-positive rates (FPRs) in non-MPXV skin lesions of the seven dermatological repositories and databases varied between 3.4% and 22.0% (Extended Data Fig. 2).

### Variation in algorithm performance by image characteristics

We evaluated the performance of the MPXV-CNN in regard to the following image characteristics: number of MPXV skin lesions, duration of the presence of the MPXV skin lesion(s) and coalescing of MPXV skin lesions.

**Extended Data Fig. 3:**
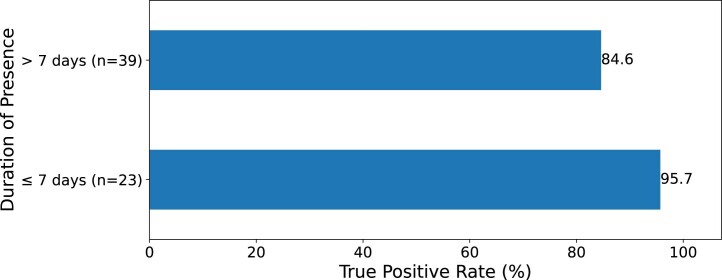
True Positive Rates by duration of presence of the MPXV skin lesion in the testing cohort. n, Number of available images per group.

**Extended Data Fig. 4:**
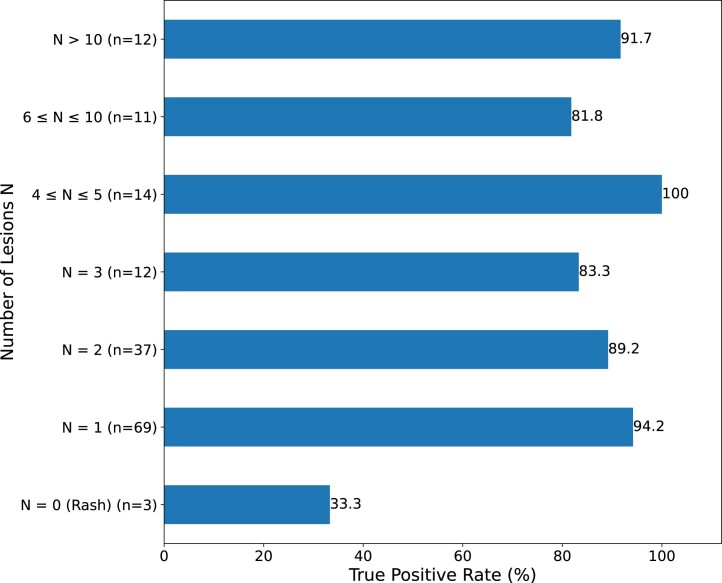
True Positive Rates by number of visible MPXV skin lesions N in the testing cohort. n, Number of available images per group; N, Number of visible MPXV skin lesions in the image.

We observed a high detection performance of MPXV lesions with a duration of the presence of less than 7 d (true-positive rate (TPR) = 95.7%; Extended Data Fig. 3) which demonstrates the early detection ability of the MPXV-CNN. Also, MPXV skin lesions with a duration of the presence of 7 d or more were detected reliably (TPR = 84.6%) illustrating the ability of the MPXV-CNN to recognize skin lesions in different disease stages. The observed median number of skin lesions in the testing cohort was two (interquartile range: (8)). We evaluated the performance in regard to the number of MPXV lesions visible in each skin lesion image. If at least one skin lesion was present, we observed a high detection performance with TPRs ranging from 81.8% (6–10 lesions) to 100% (4–5 lesions; Extended Data Fig. 4). For images showing an MPXV rash without a visible MPXV skin lesion, the detection rate was low (TPR = 33.3%) with a limited number of available images in this category (*n* = 3). The observed TPR was higher in images showing coalesced (95.5%) versus noncoalesced (91%) MPXV skin lesion images (Supplementary Fig. 1).

### Variation in algorithm performance by skin disease

**Extended Data Fig. 5:**
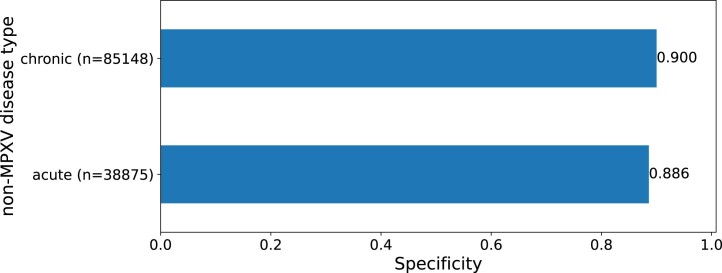
Specificity for classifying MPXV skin lesions versus acute and chronic non-MPXV skin diseases. n, Number of available images per group.

Because MPXV skin lesions present as acute skin disease, we assessed the performance in classifying MPXV skin lesions versus acute and chronic skin diseases. The testing cohort contained 38,875 images for acute and 85,148 images for chronic skin diseases. For the classification of MPXV versus other acute skin diagnoses, the specificity was 0.886 (Extended Data Fig. 5) and AUC was 0.962 (Fig. 2c). For the classification of MPXV versus chronic skin lesions, the specificity was 0.900 (Extended Data Fig. 5) and AUC was 0.967 (Fig. 2c). We also evaluated the FPRs by the category of the non-MPXV skin disease and observed the highest FPRs for the category genodermatoses and supernumerary growths (15.7%; Supplementary Fig. 2).

**Extended Data Fig. 6:**
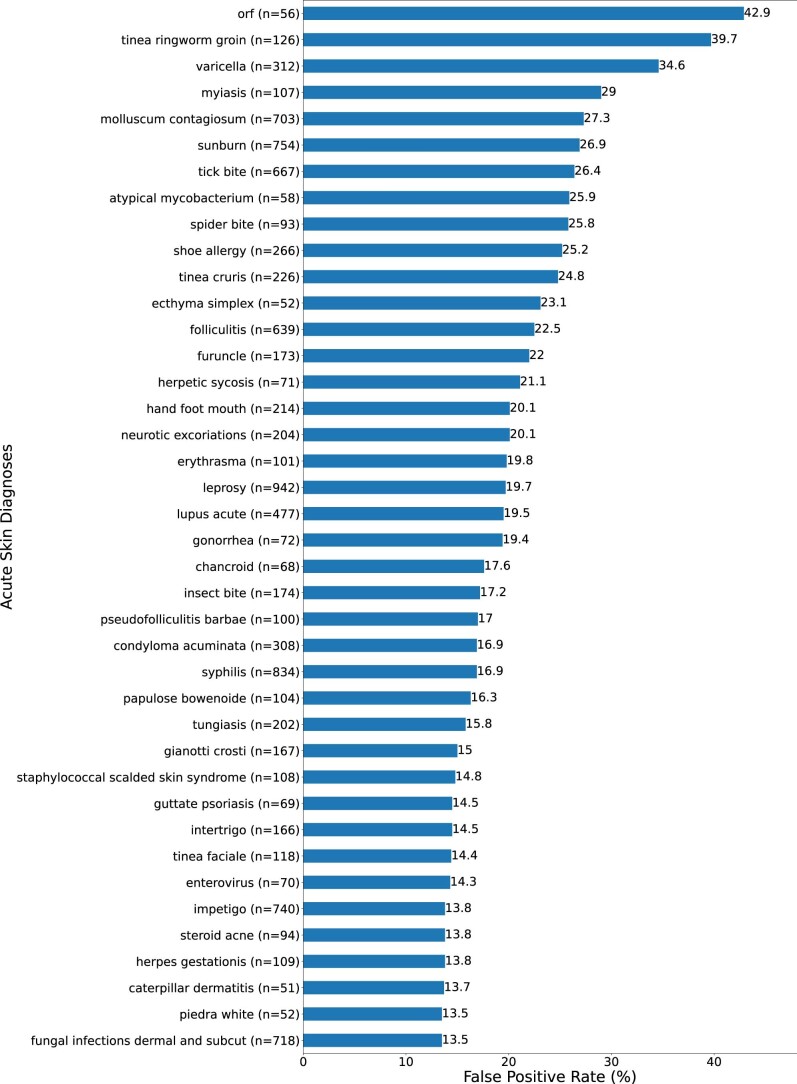
Top 30 False Positive Rates of acute diagnoses in the testing cohort with at least 50 available images. The full list of diagnoses and False Positive Rates can be found in Supplementary Tables 1–11. n, Number of available images per diagnosis.

**Extended Data Fig. 7:**
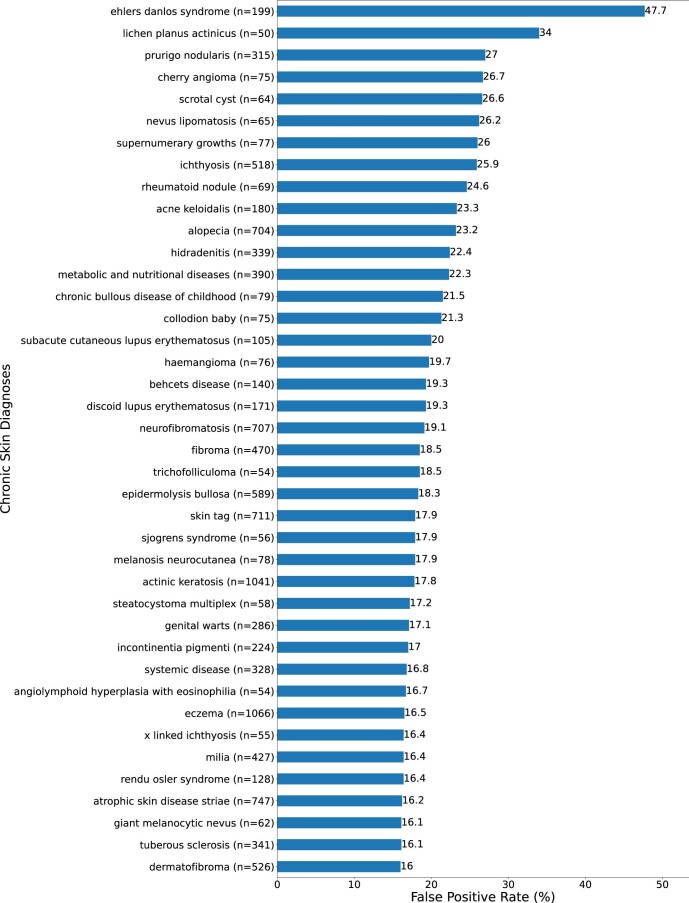
Top 30 False Positive Rates of chronic diagnoses in the testing cohort with at least 50 available images. The full list of diagnoses and False Positive Rates can be found in Supplementary Tables 1–11. n, Number of available images per diagnosis.

The number of different skin diseases with at least one available image in the non-MPXV dataset, Esteva, DermNet, DermIS, DermNet NZ, HDA, Fitzpatrick 17k, Danderm, DermNet NZ and PAD-UFES was 809, 792, 496, 458, 310, 297, 220, 178 and 6, respectively. When evaluating the performance of the MPXV-CNN in individual skin diseases with at least 50 available images, the highest FPRs were observed for the following acute skin diseases: orf (42.9%), tinea ringworm groin (39.7%) and varicella (34.6%) (Extended Data Fig. 6). We also observed a comparatively high FPR of 26.9% in images with sunburn. We observed the highest FPRs in the following chronic skin diseases: Ehlers–Danlos syndrome (47.7%), lichen planus actinicus (34%) and prurigo nodularis (27%; Extended Data Fig. 7). We found a low number of images (*n* = 20) for the Ehlers–Danlos syndrome in the training database (Supplementary Table 7). The FPR for eight differential diagnoses of MPXV was highest with orf (42.9%), followed by varicella (34.6%) and molluscum contagiosum (27.3%) (Supplementary Fig. 3). FPRs for common skin diseases such as cherry angioma, skin tags, dermatofibroma, acne vulgaris, eczema, rosacea and allergic contact dermatitis were 26.7%, 17.9%, 16.0%, 16.0%, 16.5%, 7.6% and 6.5%, respectively (Supplementary Table 2). Frequency tables and FPRs of all diagnoses in the non-MPXV dataset and per repository are available in Supplementary Tables 1–11.

### Variation in algorithm performance by body region

**Extended Data Fig. 8:**
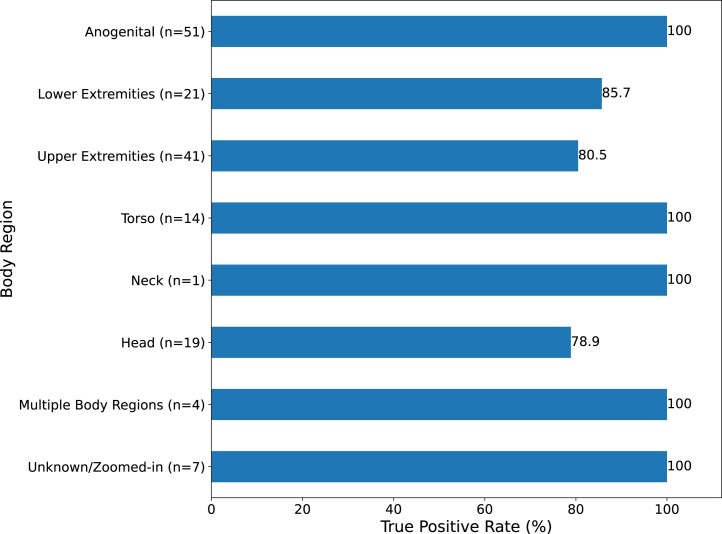
True Positive Rates by body region in the testing cohort. n, Number of available images per body region.

The performance also varied by body region of the skin lesion, with the lowest TPR at the head (TPR = 78.9%) and a high detection performance for other body regions ranging from TPR = 80.5% (upper extremities) to TPR = 100% including the anogenital body region (Extended Data Fig. 8). For MPXV skin lesion images with an ‘unknown’ body region, meaning that these images were zoomed in without visible cues of the body region, a high classification performance (TPR = 100%) could be observed (Extended Data Fig. 8). The highest FPR in non-MPXV images was observed in images showing multiple body regions (19.1%). For other body parts, the FPRs were generally low ranging from 3.6% for the anogenital to 8.8% for the torso body region (Supplementary Fig. 4).

### Variation in algorithm performance by population

We evaluated the performance of the MPXV-CNN in regard to the following population characteristics: skin tone, age group and sex.

**Extended Data Fig. 9:**
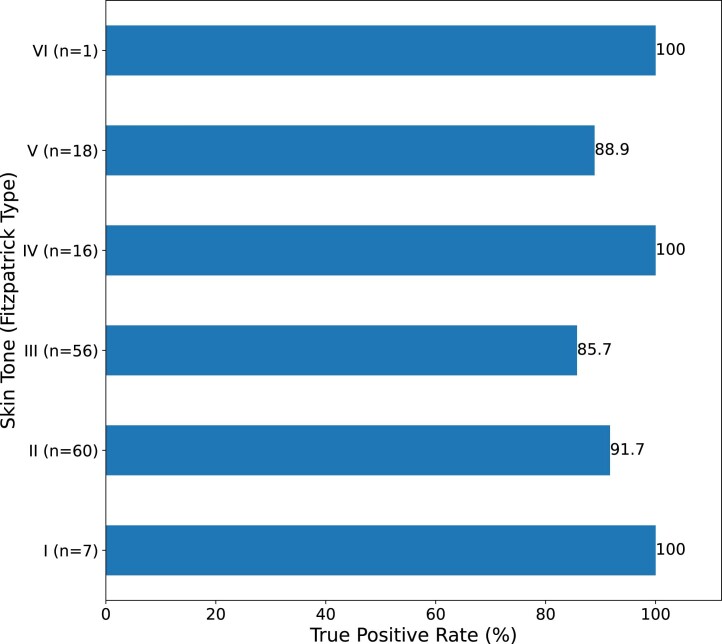
True Positive Rates by skin tone (Fitzpatrick Type) in the testing cohort. n, Number of available images per group.

**Extended Data Fig. 10:**
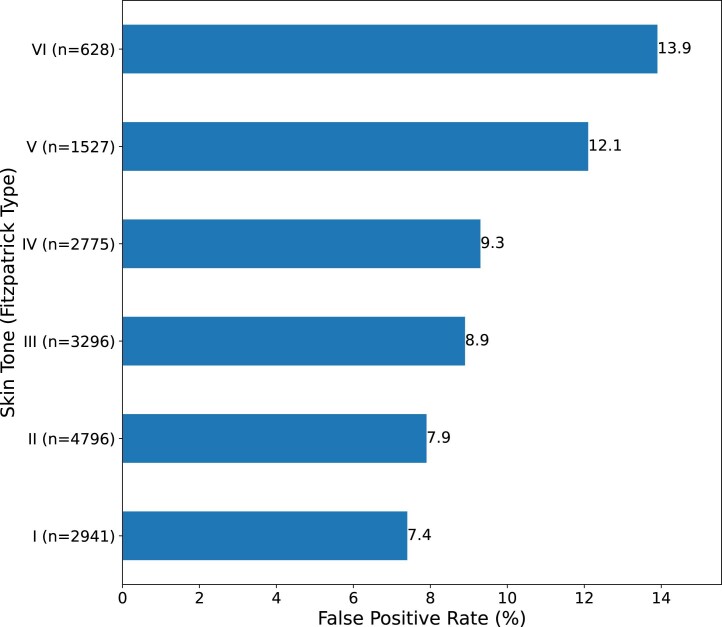
False Positive Rates by skin tone (Fitzpatrick Type) of non-MPXV images of the Fitzpatrick 17k dataset. The highest False Positive Rates could be observed in skin tone Fitzpatrick Types V and VI. n, Number of available images per group.

The TPRs varied by skin tones, with the lowest performance in Fitzpatrick type III (TPR = 85.7%) and ranging from TPR = 88.9% to TPR = 100% in other skin tones with very limited data for type 1 (*n* = 7) and type VI (*n* = 1; Extended Data Fig. 9). We observed low FPRs for type I to IV on the Fitzpatrick scale ranging from 7.4% for type I to 9.3% for type IV and higher FPRs for type V (12.1%) and 6 (13.9%; Extended Data Fig. 10). A higher FPR could be observed in children (6.8%) versus adults (4%; Supplementary Fig. 5) and male (9.7%) versus female (7.3%) individuals (Supplementary Fig. 6).

### Explanation maps

**Fig. 3:**
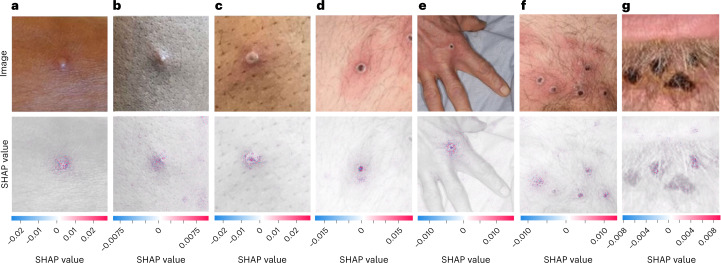
SHAP analysis of the MPXV-CNN. Photographic images of MPXV skin lesions (top) are shown with the corresponding SHAP analysis (bottom) overlaid on the original image to highlight the discriminative image regions used for detection **(a**–**g).** The MPXV lesions shown represent different stages as follows: early-stage vesicle (**a**), small pustule (**b**), umbilicated pustule (**c**), papule with central necrosis (**d**), hand with one ulcerated skin lesion (**e**), pubic region with multiple ulcerated skin lesions (**f**) and late-stage crusted plaques (**g**). Positive SHAP values, shown in red, indicated areas of the image that contributed to the prediction of MPXV skin lesion, whereas negative SHAP values, shown in blue, indicated areas that detracted from the prediction. All MPXV lesions shown in **a**–**g** were part of the testing dataset and were classified correctly by the MPXV-CNN. Photo credit (**a**–**g**): UK Health Security Agency, licensed under the Open Government License 3.0.

SHapley Additive exPlanations (SHAP) were a method to explain the prediction of an instance by computing the contribution of each feature (for example, pixel) to the prediction^25^. The SHAP method computed Shapley values from coalitional game theory. By calculating SHAP values, we were able to visualize which portions of an image the MPXV-CNN was focusing on to make a specific prediction. In the MPXV images correctly classified by MPXV-CNN, we found that the regions with high feature importance overlapped with the areas of MPXV skin lesions (Fig. 3). Correspondence between positive SHAP values and the location of the MPXV skin lesion(s) (Fig. 3a–g) and the perilesional inflammation could be observed (Fig. 3c–f).

### Personalized recommendation system for patient guidance

**Fig. 4:**
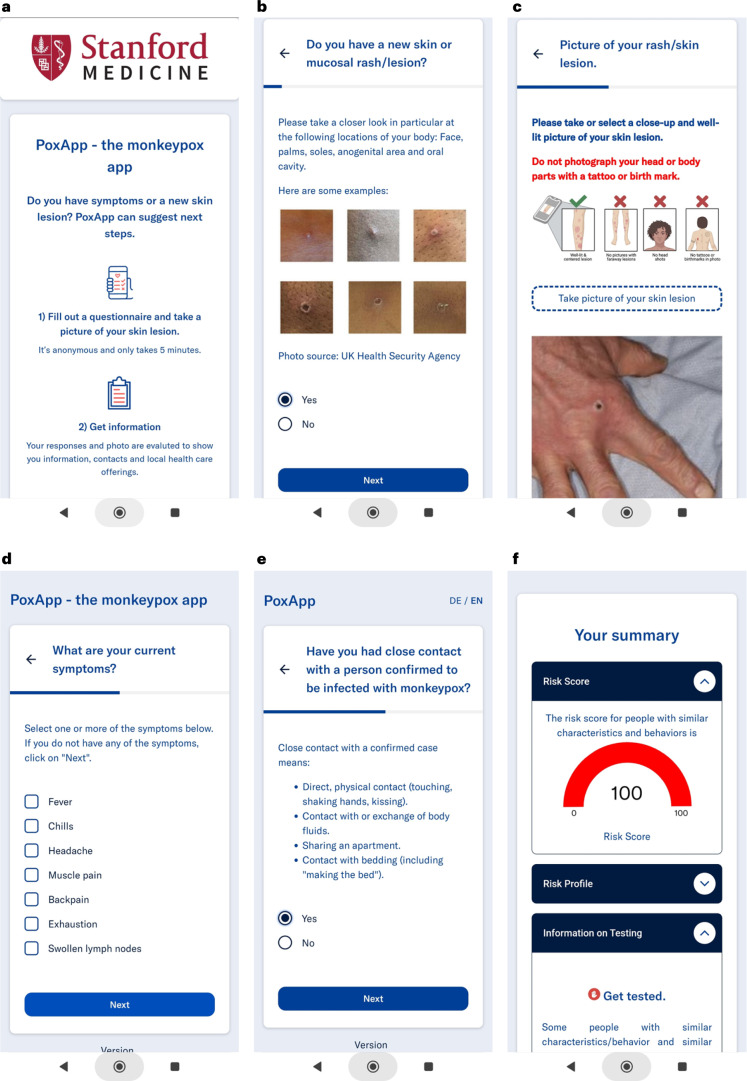
Screenshots of PoxApp. **a**, Screenshots of the start screen are shown. **b**, Question regarding the presence of new lesions. **c**, Prompt for taking a photograph of the skin lesion. **d**, Question regarding further symptoms. **e**, Question regarding close contacts with infected individuals. **f**, A personalized recommendation computed from the information provided and the MPXV-CNN classification of the skin lesion image.

**Fig. 5:**
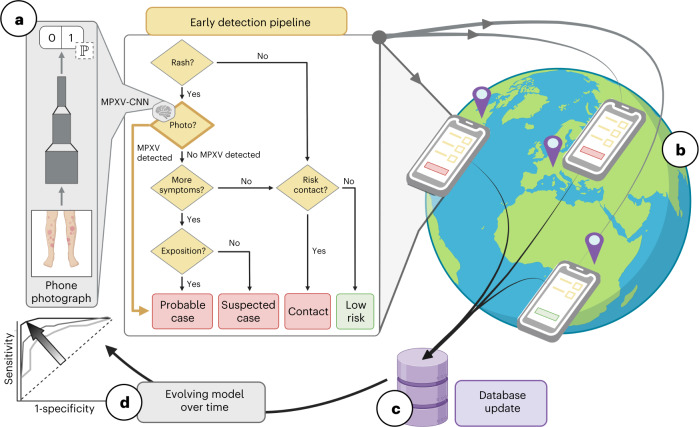
Components of the PRS for MPXV patient guidance. **a**, Simplified decision tree for MPXV infection risk stratification derived from WHO case definitions with the addition of an AI-assisted case definition based on predictions of the MPXV-CNN. An IDE was used to create and update the survey for risk stratification (boxes) based on these questions (rhombuses), logical expressions (arrows) and the MPXV-CNN (rhombus with a brain and AI model). An API distributed the most up-to-date survey, logical expressions and MPXV-CNN to web-based apps. **b**, The web-based app ‘PoxApp’ implemented the PRS for end users allowing them to answer surveys and take photos of their skin lesions and get personalized recommendations, such as MPXV testing or vaccination. **c**, Component for voluntary data donation with an API to collect, anonymize and store data in a central database. **d**, New evolving models with higher sensitivity and specificity could potentially be created based on new user data. API, application programming interface.

We developed a prototype of a personalized recommendation system (PRS) for MPXV patient guidance implemented as a web-based app named ‘PoxApp’ which could be used on web-enabled devices such as smartphones (Figs. 4 and 5). PoxApp was released as open-source on Github^26^ and published online by Charité—Universitätsmedizin Berlin in June 2022 (ref. ^27^) and Stanford University in August 2022 (ref. ^28^). The PRS combined a survey (Fig. 4b,d,e) with picture-taking of a skin lesion (Fig. 4c). The survey consisted of seven items regarding symptoms, risk contacts, sexual behavior and location (Supplementary Figs. 7–14). The PRS estimated the risk of an MPXV infection using a mobile version of the MPXV-CNN (MobileNet V3) and a decision tree (Supplementary Fig. 15). Personalized recommendations provided information on MPXV testing, postexposure vaccination and quarantine (Fig. 4f). MPXV testing was recommended if the MPXV-CNN detected an MPXV skin lesion or criteria derived from WHO case definitions for suspected and probable MPXV cases were met. Postexposure vaccination was recommended if the user encountered a risk contact within the past 21 d. Local healthcare offerings for MPXV testing and vaccination were shown based on the zip code provided by the user. We invited users to participate in a study to donate their data comprising survey answers and skin lesion images. In July 2022, we announced PoxApp to a national mailing list addressed to infectious diseases specialists. Users could find PoxApp via popular search engines and links provided by a variety of institutes such as the German National Center for Disease Control, the Ministry of Foreign Affairs, Federal Center for Health Education and Local Departments of Health.

## Discussion

We report the first proof-of-concept of an MPXV-CNN able to classify MPXV skin lesions using photographic images. The MPXV-CNN showed a high classification performance in the validation and testing datasets. We observed a sensitivity of 0.89 in prospectively collected MPXV images from patients of the Stanford University Medical Center and an overall sensitivity of 0.91 and specificity of 0.898 in the whole testing dataset. The MPXV-CNN achieved a high detection performance in MPXV skin lesions that were present for less than 7 d demonstrating its early detection capabilities. Classification performance was robust across various skin tones and body regions, and in MPXV images with a varying number of lesions with and without coalescing. Explanations of the model with SHAP demonstrated that MPXV-CNN identified the locations of MPXV skin lesions in images and their perilesional inflammation.

We performed detailed analyses and identified several parameters that impacted the performance, including the body region of the skin lesion, skin tones and non-MPXV diagnoses. The TPR for skin lesions at the head was lower compared to other body locations. This might be related to the complex facial anatomy and the presence of hair. MPXV-CNN’s best performance was achieved in the anogenital and lower extremities regions with TPR of 100% and 85.7% and FPRs of 3.6% and 3.8% which could be considered preferred locations for classification if a patient has multiple lesions. When testing performance across different body regions, we observed the highest FPR for images showing multiple body regions. It is, thus, preferable to avoid taking images at a distance. We generally observed high TPRs ranging from 85.7 to 100% across all skin tones with the lowest values in skin tone Fitzpatrick type III and very limited data for type VI. In addition, we observed higher FPRs in skin tones with Fitzpatrick type V (12.1%) and 5 (13.9%), which may be due to the challenging detection of perilesional inflammation in the darker-pigmented skin tones. In addition, we evaluated the FPRs of diagnoses in non-MPXV skin lesions using a uniform taxonomy of 2,031 skin diseases and a pooled analysis across the entire non-MPXV dataset. Because MPXV causes acute skin lesions, we specifically evaluated the classification performance of the MPXV-CNN when compared to other acute skin diseases. We observed a high performance with a specificity of 0.886 and an AUC of 0.962. The classification performance compared to chronic skin diseases was nearly identical with a specificity of 0.900 and an AUC of 0.967. While the FPRs were low in common diagnoses such as acne, eczema, rosacea and allergic contact dermatitis, we also identified common diagnoses with relatively high FPRs such as in cherry angioma which could substantially reduce the classification performance of the MPXV-CNN in elderly patients. Acute diseases with the highest FPRs were orf, tinea ringworm groin and varicella. Genetic skin disorders such as Ehlers–Danlos syndrome and neurofibromatosis yielded worse performance and could be defined as an exclusion criterion when the MPXV-CNN should not be used. Presumably, the performance could be improved by adding more images of these diagnoses to the training dataset. We conducted a preliminary analysis of known differential diagnoses and found the highest FPR in orf which is known to be hardly distinguishable from MPXV by human experts. For non-MPXV images in the testing cohort, we observed a higher FRP in male versus female individuals. For MPXV images in the testing cohort, sex-based analyses could not be performed due to the nonavailability of data for female patients. However, MPXV images without visible sexual anatomy such as zoomed-in images or images of the extremities had a high classification performance. Additionally, SHAP explanations showed that the MPXV-CNN specifically used the region of the image that contained the skin lesion and there is no evidence that MPXV lesions have a difference in appearance between male and female patients.

The main limitation of our study is related to the current scarcity of MPXV photographic images. Due to a lack of public datasets with MPXV images, we created a new dataset from publications of the scientific literature, encyclopedia articles, news articles, social media and a prospective cohort. This approach, however, is prone to biases. Authors might report pictures not of typical, but of extraordinary cases, such as patients with a generalized exanthem or superinfected lesions. Additionally, because MPXV is endemic in Africa, a significant proportion of individuals in the MPXV dataset had darkly pigmented skin. We diversified our dataset by incorporating up-to-date publications on case reports and media articles related to the current MPXV outbreak, which provided images from regions where the virus was not previously endemic. For the same reason, we integrated photos of individuals reporting an MPXV infection and sharing their pictures on social media. To prove the performance of the MPXV-CNN, we used prospectively collected images of patients with a laboratory-confirmed MPXV infection as a testing cohort. To compensate for any biases that might be present in the MPXV-negative images, we performed our analyses on a high number of images from eight different image repositories and datasets.

As pointed out by the WHO, AI has great potential for neglected tropical infections such as MPXV, but ethical and privacy considerations for AI tools have to be carefully taken into account, such as where user data are stored and data stewardship^29^. As with any infectious disease, and as is the case with MPXV, recognizing early symptoms to guide the patient toward a timely diagnosis is critical, potentially preventing severe disease, complications and secondary infections^30^. Therefore, the most benefit of an MPXV-CNN may be generated by integrating the algorithm into a mobile app usable by the public. This approach however raises concerns and comes with significant challenges. A mobile app, that takes a photo of a skin lesion as only input and returns a probability of a MPXV infection, is not sufficient in regard to the guidance for a user. Such a system could be dangerously mistaken as a substitute for a medical test such as a PCR test for MPXV or medical evaluation and treatment. Predictions of the MPXV-CNN need to be evaluated in context with a variety of factors influencing the pretest probability for an infection such as further symptoms reported by the users, close contact with infected individuals and the incidence of infectious cases at the location of the user, or factors that increase the probability for severe diseases such as pregnancy or immune compromise. A system was needed that combines the prediction of the MPXV-CNN with expert knowledge of healthcare professionals considering all the aforementioned factors to generate easy-to-understand recommendations for users.

Therefore, we proposed the combination of the MPXV-CNN with a PRS and developed a prototype that (1) asked survey questions to get a clinical picture of the user, (2) provided instructions to mitigate weaknesses of the MPXV-CNN such as taking a picture of the body regions with the highest predictive power and (3) gave easy to understand personalized recommendation based on the estimated risk of infection. At the time of writing, the PRS was evaluated in a prospective trial. Additionally, by integrating the function of a voluntary data donation into such a system, a PRS could become a source of big data for skin lesion images reflecting closely the true distribution of the users’ age, sex, skin tone, ratio of MPXV and non-MPXV skin lesions and non-MPXV diagnoses. However, the MPXV infection status is unknown at the time the user uses the PRS. This limitation can be overcome with modern, semisupervised machine learning techniques that could use large amounts of skin lesion images with unknown infection status for pretraining and would require just a fraction of images with known infection status for learning^31^ which could be acquired by recalling the user or by a clinical trial.

Further investigations are needed to assess whether the high predictive power of MPXV-CNN obtained from our experiments can be translated into other settings such as an app used by the general public. The high classification performance observed in MPXV images collected from patients is promising. However, a prospective trial with patients under real-world conditions and larger datasets of MPXV skin lesion images will be required for this evaluation.

In this first version of the MPXV-CNN, predictions will also be made if the image has a low quality such as in low-light conditions or with significant blurriness. New methods like uncertainty quantifications of CNNs could help detect cases where the prediction of the MPXV-CNN should not be used^32^. Additional evaluations such as the analysis of the MPXV-CNN of multiple images from different body locations of the same patient could help to improve the performance of the MPXV-CNN. Lastly, the ResNet34 architecture researched in this study was not optimized for mobile devices due to its model complexity and the high number of parameters (21.5 million). Additional evaluations will be necessary to compare the performance with mobile-optimized architectures such as EfficientNet^33^.

We propose the following next steps. First, skin lesion images from patients who suspect they are infected with MPXV should be acquired as part of a prospective, multicentered trial. The MPXV and non-MPXV skin lesion images could be used as a testing dataset for next-generation MPXV-CNNs. Second, a prospective, clinical trial on the PRS should be conducted to assess the real-world performance of the MPXV-CNN, risks of misclassifications, compliance of patients to PRS recommendations and cost impact on the healthcare system. Third, efforts for a successful deployment should be made by targeting populations with a high prevalence of MPXV and endemic areas in low-income countries. Fourth, the proposed PRS could be integrated into local early warning systems at a national level that processes additional orthogonal information that enhances the PRS and increases its merit. From a scientific perspective, the combination of imagery data, disease information, demographic data and governmental policies creates a unique multimodal dataset.

This first MPXV-CNN could classify photos of skin lesions as being from an MPXV infection or not with a comparatively high degree of discrimination in a testing cohort that included prospectively collected MPXV images of patients. Technologies like the MPXV-CNN can lead the way to AI-assisted case definitions of MPXV and other infectious diseases. We developed an app-based PRS with the integration of a mobile version of the MPXV-CNN that allowed users to upload a photo of their skin lesion and get personalized recommendations. In such a setting, the MPXV-CNN has the potential to accelerate appropriate care-seeking and increase the adoption of behaviors that reduce onward transmission. The images sourced with a PRS could become a rich source of data for the further development and improvement of AI-assisted approaches to address the current and future MPXV outbreaks.

## Methods

Ethical oversight was provided by the Stanford institutional review board (Protocol: 36050, 67068 and 66980). In this study, we evaluated publicly available images and clinical images acquired prospectively from patients with a laboratory-confirmed MPXV infection at the Stanford University Medical Center. Informed consent was obtained from patients for clinical images, but not for images sourced from publicly available datasets and repositories as it was not required after having received permission to use the images from the database manager(s). We followed the MINimum Information for Medical AI Reporting^34^ recommendations for reporting (1) data source, (2) detailed information on model architecture and development and (3) approaches to optimize, evaluate and validate the model performance.

### Data sources

To train and test the MPXV-CNN, we constructed a new dataset of photographic images of skin diseases (*n* = 139,198) originating from multiple publicly available sources, an institutional cohort (Esteva Dataset)^13^ and patients (Fig. 1): 676 images of MPXV skin lesions were aggregated from publications of the scientific literature, encyclopedia articles, news articles, social media (Twitter) and the prospective cohort (MPXV dataset) and 138,522 images of non-MPXV skin lesions (non-MPXV dataset) from five dermatological repositories and three datasets (Table 1). Patients of the prospective cohort were recruited from the Stanford University Medical Center between July and August 2022. We included all patients with a laboratory-confirmed MPXV infection and visible skin lesions. We excluded patients who received any prior treatment due to their MPXV infection. Skin lesion images were taken from all affected body regions with a smartphone camera by a healthcare professional. The original Esteva dataset has been improved since its initial release and received several rounds of data cleansing. We identified duplicate images in the MPXV and non-MPXV datasets by comparing the visual contents of the images using a conservative cutoff value of 80% for similarity. We provided instructions for obtaining publicly available MPXV and non-MPXV images in Data Availability. A bibliography of sources with MPXV images and a list of URLs to non-MPXV images of Danderm, DermIS and HDA were provided as Supplementary Notes 1 and 2.

### Image selection and annotation

We observed a higher number of duplicate images in the Esteva dataset and the other non-MPXV datasets of this study (*n* = 45,440). We excluded images (total *n* = 47,518) from the MPXV dataset (*n* = 36) and non-MPXV dataset (*n* = 47,554) if the following criteria were met: absence of a skin lesion or rash, containing more than one photographic image, showing surgical or other medical interventions, nonphotographic images such as histopathology slides or radiology imaging, duplicate image or inaccessibility. We performed a reverse image search for all MPXV skin lesion images sourced from social media and excluded images that had been published previously in another context. We manually labeled the MPXV dataset for the age group (child: < 18 years, adult: ≥ 18 years, unknown), sex (male, female, unknown), skin tone (type I–VI, Fitzpatrick scale^35^), continent where the image was taken (Europe, Africa, Asia, South America, North America, Antarctica, Australia, unknown), number of skin lesions (*n* up to 50, more than 50 lesions were labeled as 50, and highly coalesced lesions as unknown), body region of the skin lesion(s) (head, neck, torso, upper extremity, lower extremity, anogenital, multiple locations, zoomed in/unknown), duration of skin lesion presence (less than 7 d, 7 d or more, unknown) and association with the 2022 MPXV outbreak (yes/no), defined as the publication of the image after May 1, 2022. For the prospective cohort, sex was defined as sex at birth self-reported by the patient. For other sources, sex was defined as reported in the textual information of the source. If no information on sex was reported, sex was assigned following evaluation of the image if sexual anatomy was visible. If the age information was not available, we labeled the age group of the individual from the image using a panel and labeled the age group as unknown if no consensus could be reached. We labeled MPXV images as coalesced if at least two MPXV lesions had grown together (yes/no or not applicable for MPXV rash). We evaluated the diagnoses found in the metadata of the Fitzpatrick 17k, PAD-UFES-20, DermNet and Esteva datasets and scraped metadata from websites of Danderm, DermIS, HDA, DermNet NZ repositories. To enable evaluations of non-MPXV diagnoses of all repositories and datasets, we mapped all diagnoses to a taxonomy of 2,032 individual skin diseases and classified them into nine main categories (benign dermal tumors, cysts, sinuses; cutaneous lymphoma and lymphoid infiltrates; epidermal tumors, hamartomas and milia; epidermal premalignant and malignant tumors; genodermatoses and supernumerary growths; inflammatory; malignant dermal tumor; pigmented benign lesions; pigmented malignant lesions) previously developed at our institute^13^. All diagnoses were classified as acute or chronic (defined as a persistent, progressive or recurring disease). Diagnoses with the possibility of acute and chronic courses were classified as acute. We specifically analyzed differential diagnoses with a similar appearance: varicella, drug-induced allergies, impetigo, measles, orf, molluscum contagiosum, scabies and syphilis. Where available, we evaluated information in the non-MPXV datasets and repositories in regard to the age group, sex, skin tone and location of the skin lesion(s) using identical definitions as for MPXV lesions.

### Data splitting

After image filtering, there were 676 images for MPXV lesions and 138,522 images for non-MPXV lesions. We split these images into training and testing cohorts. The training cohort was used for training, hyperparameter tuning and internal validation, while the testing cohort was used as a hold-out dataset for external validation. For the MPXV lesions, we used 63 skin lesion images from the Stanford University Medical Center, 87 images from a recent publication with the largest MPXV case series to date from 16 countries^4^ and 8 images from a publication showing MPXV skin lesions in different stages^36^ as the MPXV testing cohort (total *n* = 158). The remaining MPXV images (*n* = 518) were used as the training cohort. While the training cohort contained skin lesion images of the 2022 MPXV outbreak and before, the testing cohort only contained images of the 2022 MPXV outbreak. In the training cohort, we used MPXV images sourced from publications of the scientific literature, news articles and social media. In the testing cohort, we exclusively used MPXV images with a laboratory-confirmed MPXV infection originating from publications and patients from our own institute. For the non-MPXV lesions, we used images (*n* = 12,045) from the DermNet NZ repository in the training cohort, due to the high number of available pictures, known ratios of sex and age groups and a high variety of diagnoses, races and origins. The remaining non-MPXV images (*n* = 126,477) were used in the testing cohort. For internal validation, we split the training cohort into 80% for training and 20% for validation.

### Image processing and training algorithm

We treated the problem as a binary image classification task for which the model aimed to predict whether a provided photographic image was an MPXV or non-MPXV skin lesion. Several challenges were encountered while developing a robust classification model. First, because the images were collected from different sources such as publications of the scientific literature, encyclopedias, news articles and social media, there was high variability in image features, such as resolution, lighting, angle, zoom, color profiles and filters. Second, despite our best efforts, the number of images collected for the MPXV cases was much smaller compared to the non-MPXV cases. Therefore, the class distribution was highly imbalanced, which caused bias in the predictions toward the majority class (that is, non-MPXV).

To overcome these issues, we incorporated several strategies into image processing, model selection and training algorithms. First, we made use of data augmentation. All images were first resized to 448 × 448 pixels in size, and we then performed random cropping and resizing (224 × 224 pixels), random horizontal flip, random rotation (max degree = 360°), random zoom (max scale = 1.1), perspective warping (max value = 0.2), random brightness and contrast, random affine transformations and random reflections. This data augmentation was performed on both MPXV and non-MPXV images in the training cohort to account for the aforementioned high image variation. Secondly, we pursued a Transfer Learning strategy using a pretrained model, which was later fine-tuned on our domain-specific data. We experimented with a variety of different CNN architectures implementing Transfer Learning, including ResNet18 (ref. ^37^), ResNet34 (ref. ^37^), ResNet50 (ref. ^37^), Resnet152 (ref. ^37^), DenseNet169 (ref. ^38^) and VGG19_bn^39^. We adopted the ResNet34 (ref. ^37^) CNN architecture, where the weights of the model were initialized using the weights of a model pretrained on ImageNet^40^ (approximately 14 million images), and we fine-tuned the model using our images of skin lesions. Third, we implemented a weighted categorical cross-entropy loss to account for class imbalance. Because the number of images for MPXV skin lesions was lower than the number of non-MPXV skin lesions, we assigned a higher class weight to MPXV skin lesions in the cost function of the training algorithm so that it could provide a higher penalty to the misclassification of the minority class. To find the optimal pair of class weight for the MPXV and non-MPXV skin lesions, we tested different weight pairs *W*, where *W* ∈ {(1.0, 0.005), (1.0, 0.01), (1.0, 0.05), (1.0, 0.1), (1.0, 0.5), (1.0, 1.0)}. Using each different *W*, we fine-tuned the model for one epoch on the last layer and 20 epochs on all layers. The minibatch size was set to 64 and the base learning rate *lr* was set to 0.002. We computed the cross-entropy loss, sensitivity, specificity and AUC for the validation set. The optimal performance was achieved with a class weight *W* of (1.0, 0.01). Finally, to qualitatively verify that the MPXV-CNN learned to detect MPXV lesions, we generated explanation maps on a subset of images in the testing cohort using SHAP^25^. This method quantitatively annotated which image area(s) are critical for the final decision made from the MPXV-CNN.

### Algorithm evaluation

#### Cross-validation

We carried out stratified fivefold cross-validation, where images from the training cohort were split into 80% for training and 20% for validation. Because images from the same source may originate from the same patient and share similar image features, we grouped images by the source such that MPXV images coming from the same patient were not split between the training and validation sets. Running the cross-validation for only a single time may result in a noisy estimate of model performance because different splits of the data may result in different results. Therefore, we repeated the cross-validation five times. In each repeat, we shuffled the order of images so that we could implement a different split of the dataset into the k(5)-folds.

#### Evaluation metrics

To evaluate our model performance, we used three metrics: sensitivity, specificity and AUC score. For each repeat of the fivefold cross-validation, we averaged the scores evaluated from each fold, and we reported the mean and standard deviation of scores obtained from the five repeats.

#### Explainability

SHAP^25^ uses game theoretic approaches to calculate the importance of a feature when the model makes a specific prediction. A higher SHAP value indicates higher importance of the feature. To approximate SHAP values, we used the Gradient Explainer, which explains a model using expected gradients (an extension of integrated gradients^41^). We applied the explainer to the final model trained on the entire training cohort and used it to generate the SHAP values of the MPXV images from the testing cohort. The SHAP values were then overlaid on the gray-scaled images for visualization.

### Development of the PRS

We developed a web-based app named ‘PoxApp’ that implemented a PRS for MPXV patient guidance. The source code was derived from an open-source PRS that we previously created for the SARS-CoV-2 pandemic^42^. Because the original PRS was purely survey-based, extensive development was necessary to integrate a mobile version of the MPXV-CNN. Survey questions and logical expression were derived from WHO case definitions for suspected and probable MPXV cases,^11^ and we added an AI-assisted case definition based on the MPXV-CNN classification. Because many MPXV patients developed lesions in the anogenital region, privacy concerns might be a major issue for users when uploading images to the PRS. To increase user acceptance, we, therefore, made design decisions that allowed anonymous usage of the PRS. The PRS had the following components (Fig. 5).

#### Integrated development environment

We developed a web-based integrated development environment (IDE) to create and update PoxApp’s survey, the MPXV-CNN and logical expressions for MPXV infection risk estimation and personalized recommendations (Fig. 5a). We developed a module for picture-taking that could be integrated into the survey. Using the IDE’s script language, we translated clinical expert knowledge to logical expressions to estimate the risk of an MPXV infection from survey answers and the MPXV-CNN classification. We created personalized recommendations according to the estimated risk of infection. Using an application programming interface, the survey, MPXV-CNN, logical expressions and personalized recommendations were sent to web-based apps.

#### Web-based app

We developed a web-based app named PoxApp for end users to answer survey questions, take photos of their skin lesion(s) and get personalized recommendations (Fig. 5b). PoxApp could be used from web-enabled devices such as smartphones, tablets or personal computers. A built-in engine used the computing power of the user device to execute logical expression and the MPXV-CNN. This resulted in two key advantages as follows: (1) because the user data was analyzed locally on the user device, there was no need to send survey answers and images to external servers resulting in maximum data privacy; and (2) the system was scalable to a high number of users at a relatively low cost because no expensive servers with high computational power were necessary. We aimed to release PoxApp in the United States and Germany. For this reason, we translated PoxApp’s user interface to English and German and adapted the Terms of Use and Privacy Policies to the US and European jurisdictions.

#### Data donation service

We developed a data donation service, so users of PoxApp could volunteer to donate their answers and skin lesion images (Fig. 5c). The data donation service removed personal identifiers such as an IP address and forwarded the anonymized information to a database server. The donated data could potentially be used to generate next-generation MPXV-CNNs with higher performance (Fig. 5d).

### Reporting summary

Further information on research design is available in the Nature Portfolio Reporting Summary linked to this article.

## Online content

Any methods, additional references, Nature Portfolio reporting summaries, source data, extended data, supplementary information, acknowledgements, peer review information; details of author contributions and competing interests; and statements of data and code availability are available at 10.1038/s41591-023-02225-7.

## Supplementary information

Supplementary Information Supplementary Tables 1–10 and 12, Supplementary Figs. 1–15 and Supplementary Notes 1 and 2.

Reporting Summary

Supplementary Table 11 Frequency tables and false-positive rates of diagnoses of the non-MPXV dataset.

## Data Availability

This study used publicly available data from publications of the scientific literature, dermatological repositories, news articles and social media. A bibliography of sources with MPXV skin lesion images was provided as Supplementary Note 1. Dermatological repositories with non-MPXV images can be accessed using the following addresses: Danderm: danderm-pdv.is.kkh.dk; DermIS: dermis.net; HDA: hellenicdermatlas.com; DermNet: dermnet.com/ DermNet NZ: dermnetnz.org A list of URLs to cleaned non-MPXV skin disease images of Danderm, DermIS, HDA, was provided as Supplementary Note 2. The images and metadata of datasets can be obtained from the following addresses: DermNet: https://www.kaggle.com/datasets/shubhamgoel27/dermnet PAD-UFES-20: data.mendeley.com/datasets/zr7vgbcyr2/1 Fitzpatrick 17k: github.com/mattgroh/fitzpatrick17k Social media references are available upon request. MPXV images of the prospective cohort from the Stanford University Medical Center and the Esteva dataset are nonpublic and cannot be shared.

## Code availability

The deep-learning framework (FastAI v2) used in this study is available at https://www.fast.ai/. The pretrained ResNet34 architecture used for the MPXV-CNN in this work is publicly available within the FastAI framework. The SHAP library used for explainability in this study is available at https://github.com/slundberg/shap. The code of PoxApp is available at https://github.com/PoxApp/PoxApp. The code for training the MPXV-CNN is available at https://github.com/PoxApp/Model. The following packages were used which can be installed with the python package installer (pip): pytorch 1.12.0, fastai 2.7.7,scikit-image 0.19.3, python 3.7.13, torchvision 0.13.0, cudatoolkit 11.6.0, matplotlib 3.5.2. We used dupeGuru 4.31 to identify duplicate images which is available at https://dupeguru.voltaicideas.net/.
