# Supplementary material for: A deep-learning algorithm to classify skin lesions from mpox virus infection
Source: Nat Med. 2023 Mar 2;29(3):738–47. doi: 10.1038/s41591-023-02225-7 (PMC10033450; doi:10.1038/s41591-023-02225-7)
Supplement: Supplementary file 1 — Supplementary Tables 1–10 and 12, Supplementary Figs. 1–15 and Supplementary Notes 1 and 2. [file 41591_2023_2225_MOESM1_ESM.pdf]

nature medicine

Article https://doi.org/10.1038/s41591-023-02225-7

A deep-learning algorithm to classify skin lesions from mpox virus infection

In the format provided by the authors and unedited

Table of Contents

Supplementary Table 1: Frequency Table of Diagnoses in the non-MPXV Dataset **3**

Supplementary Table 2: Frequency Table and False Positive Rates of Diagnoses in the non-MPXV testing cohort **29**

Supplementary Table 3: Frequency Table and False Positive Rates of Diagnoses in the Esteva Dataset **55**

Supplementary Table 4: Frequency Table and False Positive Rates of Diagnoses in the Danderm Repository **80**

Supplementary Table 5: Frequency Table and False Positive Rates of Diagnoses in the DermIS Repository **86**

Supplementary Table 6: Frequency Table and False Positive Rates of Diagnoses in the DermNet dataset **101**

Supplementary Table 7: Frequency Table of Diagnoses in the DermNet NZ Repository **117**

Supplementary Table 8: Frequency Table and False Positive Rates of Diagnoses in the Fitzpatrick 17k Dataset **127**

Supplementary Table 9: Frequency Table and False Positive Rates of Diagnoses in the HDA Repository **135**

Supplementary Table 10: Frequency Table and False Positive Rates of Diagnoses in the PAD-uefs 20 Dataset **145**

Supplementary Table 11: Frequency Tables and False Positive Rates of Diagnoses of the non-MPXV dataset as Excel File **146**

Supplementary Table 12: Performance in the detection of MPXV skin lesions using different model architectures. **147**

Supplementary Figure 1: True Positive Rates of the MPXV-CNN in the testing cohort in coalesced and non-coalesced MPXV skin lesions. **148**

Supplementary Figure 2: False Positive Rates by category of non-MPXV skin disease in the testing cohort. **149**

Supplementary Figure 3: False Positive Rates by differential diagnoses of MPXV in the testing cohort. **150**

Supplementary Figure 4: False Positive Rates by body region of non-MPXV images in the testing cohort (DermIS repository). **151**

Supplementary Figure 5: False Positive Rates by age group in non-MPXV images of the testing cohort (DermIS repository). **152**

Supplementary Figure 6: False Positive Rates by sex in the testing cohort (DermIS and PAD-uefs 20). **153**

Supplementary Figure 7: Screenshot of PoxApp for the question on the presence of a skin lesion. **154**

Supplementary Figure 8: Screenshot of PoxApp of interface for taking or selecting a picture of a skin lesion. **155**

Supplementary Figure 10: Screenshot of PoxApp for date of onset of symptoms. **157**

Supplementary Figure 11: Screenshot of PoxApp for the question on risk contacts. 158

Supplementary Figure 12: Screenshot of PoxApp for the question on the date of risk contact. 159

Supplementary Figure 13: Screenshot of PoxApp for the question on sexual preferences. 160

Supplementary Figure 14: Screenshot of PoxApp for the zip code question. 161

Supplementary Figure 15: Logic decision tree of PoxApp for MPXV infection risk classification incorporating the MPXV-CNN. 162

Supplementary Note 1: Bibliography of sources with images of MPXV lesions. 163

Supplementary Note 2: URLs of non-MPXV images from Danderm, DermIS and HDA. 172

Supplementary Table 1: Frequency Table of Diagnoses in the non-MPXV Dataset

Frequency table of diagnoses of evaluated skin lesion images in the repositories and datasets comprising the non-MPXV dataset used for training and testing the MPXV-CNN.
All, Number of all evaluated skin lesion images in the non-MPXV dataset; MPXV, Mpox virus; MPXV-CNN, Mpox virus convolutional neural network; n, Number of occurrences of diagnosis; Unknown, Diagnosis unknown or unmapped; %, Percentage of diagnosis in relation to all evaluated skin lesion images in the non-MPXV dataset.

Category	Diagnosis	n	%
	**pilar cyst**	879	0.63
	**skin tag**	735	0.53
	**hemangioma**	655	0.47
	**pyogenic granuloma**	554	0.40
	**dermatofibroma**	545	0.39
	**telangiectases**	492	0.36
	**fibroma**	470	0.34
	**syringoma**	394	0.28
	**sebaceous hyperplasia**	365	0.26
	**fordyce spots**	318	0.23
	**mucous cyst**	305	0.22
**Benign dermal tumors cysts sinuses**	**becker nevus**	250	0.18
	**angiokeratomas**	226	0.16
	**lymphangioma**	215	0.16
	**pilomatricoma**	155	0.11
	**venous lake**	132	0.10
	**adenoma sebaceum**	131	0.09
	**port wine stain**	120	0.09
	**venous malformations**	108	0.08
	**myxoid cyst**	105	0.08
	**trichoepithelioma**	105	0.08
	**granuloma pyogenic**	97	0.07
	**granulation tissue**	95	0.07

**leiomyoma**	92	0.07
**cherry angioma**	87	0.06
**hidrocystoma**	87	0.06
**angioma**	83	0.06
**haemangioma**	76	0.05
**hemangioma infancy**	67	0.05
**nevus lipomatosis**	65	0.05
**scrotal cyst**	64	0.05
**steatocystoma multiplex**	64	0.05
**spider angioma**	63	0.05
**spider nevus**	63	0.05
**apocrine hydrocystoma**	59	0.04
**cutis marmorata telangiectatica congenita**	56	0.04
**eccrine poroma**	55	0.04
**glomus tumor**	54	0.04
**trichofolliculoma**	54	0.04
**cylindroma**	50	0.04
**dilated pore of winer**	48	0.03
**klippel trenaunay syndrome**	46	0.03
**nevus anemicus**	44	0.03
**ganglion**	41	0.03
**chondroma**	40	0.03
**eruptive vellus hair cysts**	38	0.03
**dermoid cyst**	36	0.03
**connective tissue nevus**	35	0.03
**acrotrichoma**	34	0.02
**pilonidal sinus**	34	0.02
**infantile digital fibromatosis**	33	0.02
**spiradenoma**	33	0.02
**strawberry hemangioma**	29	0.02
**neurolemmoma**	28	0.02
**periungual fibroma**	27	0.02
**ectopic respiratory epithelia**	26	0.02

	nevus flammeus	25	0.02
	vascular anomaly	24	0.02
	collagen nevus	23	0.02
	dental sinus	22	0.02
	endometriosis	20	0.01
	aponeurotic fibroma	19	0.01
	hemangioma racemosum	19	0.01
	myxoma	19	0.01
	angioma serpiginosum	18	0.01
	epithelioma adenoides cysticum	18	0.01
	lymphatic malformations	18	0.01
	fibrolipoma	17	0.01
	arteriovenous malformation	16	0.01
	trichoepithelioma papulosum multiplex	15	0.01
	cirsoid aneurysm	14	0.01
	myofibromatosis	13	0.01
	trichoblastoma	13	0.01
	tenosynovitis nodular	12	0.01
	cyst synovial	11	0.01
	trichoadenoma	10	0.01
	perifolicular fibroma	8	0.01
Cutaneous lymphoma and lymphoid infiltrates	cutaneous t cell lymphoma	793	0.57
	lymphoma b cell	180	0.13
	lymphomatoid papulosis	131	0.09
	erythema arciforme et palpabile migrans	43	0.03
	pseudo lymphoma other types	23	0.02
	lymphomas lymphocytic infiltrations	12	0.01
Epidermal tumors hamartomas milia and growths benign	seborrheic keratosis	1294	0.93
	epidermal nevus	876	0.63
	porokeratosis	594	0.43
	seborrheic keratoses ruff	478	0.35
	milia	448	0.32
	benign keratosis	414	0.30

	prurigo nodularis	361	0.26
	seborrheic keratoses smooth	156	0.11
	seborrheic keratosis irritated	150	0.11
	corns	101	0.07
	dermatosis papulosa nigra	99	0.07
	callus	81	0.06
	stucco keratoses	67	0.05
	acquired digital fibrokeratoma	58	0.04
	stucco keratosis	36	0.03
	acral keratosis	34	0.02
	giant comedo	34	0.02
	keratosis seborrheic	26	0.02
	clear cell acanthoma	23	0.02
	leser trelat sign	19	0.01
	warty dyskeratoma	11	0.01
Epidermal tumors pre malignant and malignant	basal cell carcinoma	2664	1.92
	squamous cell carcinoma	1333	0.96
	actinic keratosis	1214	0.88
	solar damage actinic keratosis	951	0.69
	keratoacanthoma	828	0.60
	bowens disease	703	0.51
	cutaneous horn	218	0.16
	solid cystic basal cell carcinoma	168	0.12
	actinic cheilitis	158	0.11
	basal cell carcinoma lesion	138	0.10
	pagets disease	135	0.10
	leukoplakia	133	0.10
	basal cell carcinoma morpheiform	115	0.08
	extramammary paget disease	88	0.06
	arsenical keratoses	84	0.06
	erythroplasia queyrat	81	0.06
	basal cell carcinoma nose	79	0.06
	basal cell nevus syndrome	79	0.06

**actinic keratosis face**	77	0.06
**actinic keratosis lesion**	66	0.05
**basal cell carcinoma superficial**	65	0.05
**solar damage**	63	0.05
**actinic keratosis fu**	59	0.04
**basal cell carcinoma lid**	55	0.04
**basal cell carcinoma ear**	53	0.04
**pigmented basal cell carcinoma**	53	0.04
**basal cell carcinoma face**	50	0.04
**verrucous carcinoma**	49	0.04
**actinic keratosis hand**	47	0.03
**squamous cell carcinoma ear**	43	0.03
**superficial basal cell carcinoma**	42	0.03
**basal cell carcinoma ulcerating**	37	0.03
**nevoid basal cell carcinoma syndrome**	37	0.03
**basal cell carcinoma lip**	36	0.03
**solar damage actinex treatment**	36	0.03
**keratosis solar**	35	0.03
**squamous cell carcinoma lesion**	34	0.02
**carcinoma of lip**	32	0.02
**basal cell carcinoma scalp**	29	0.02
**squamous cell carcinoma lip**	29	0.02
**actinic keratosis ear**	28	0.02
**hypertrophic actinic keratosis**	27	0.02
**actinic keratosis horn**	26	0.02
**leucoplakia praecancerosa**	23	0.02
**ulcus terebrans**	22	0.02
**solar damage actinic cheilitis**	20	0.01
**actinic keratosis nose**	17	0.01
**squamous cell carcinoma face**	17	0.01
**basal cell carcinoma trunk ext**	15	0.01
**squamous cell carcinoma nose**	15	0.01
**actinic keratosis pigmented**	14	0.01

	squamous cell carcinoma hand arm	12	0.01
	basal cell carcinoma aldara	11	0.01
	keratoacanthoma pendulum	11	0.01
	solar damage fu treatment	10	0.01
	basal cell carcinoma sclerosing	1	0.00
Genodermatoses and supernumerary growths	neurofibromatosis	731	0.53
	epidermolysis bullosa	597	0.43
	ichthyosis	555	0.40
	dariers disease	543	0.39
	tuberous sclerosis	375	0.27
	hailey hailey disease	293	0.21
	incontinentia pigmenti	229	0.17
	ehlers danlos syndrome	219	0.16
	ichthyosis vulgaris	219	0.16
	acrodermatitis enteropathica	137	0.10
	rendu osler syndrome	128	0.09
	xeroderma pigmentosum	127	0.09
	vascular tumors and malformations	120	0.09
	pseudoxanthoma elasticum	113	0.08
	epidermolytic hyperkeratosis	94	0.07
	collodion baby	77	0.06
	supernumerary growths	77	0.06
	ichthyosis congenita	70	0.05
	erythrokeratodermia variabilis	65	0.05
	erythrokeratodermia progressive symmetric	63	0.05
	ichthyosis sex linked	62	0.04
	x linked ichthyosis	61	0.04
	lipoid proteinosis urbach wiethe disease	58	0.04
	leopard syndrome	57	0.04
	rothmund thomson syndrome	47	0.03
	ichthyosis other forms	42	0.03
	acrokeratoelastoidosis	41	0.03

	**congenital ichthyosiform erythroderma**	41	0.03
	**peutz jeghers syndrome**	41	0.03
	**ichthyosis dominant**	38	0.03
	**congenital erythropoietic porphyria**	37	0.03
	**muir torre syndrome**	37	0.03
	**acrokeratosis verruciformis**	36	0.03
	**albinism**	34	0.02
	**birt hogg dube**	34	0.02
	**cowden disease**	34	0.02
	**congenital dyskeratosis**	27	0.02
	**buschke ollendorf syndrome**	25	0.02
	**congenital anomalies**	22	0.02
	**sjgren larsson syndrome**	17	0.01
	**laugier hunziker s syndrome**	15	0.01
	**lamellar icthyosis**	13	0.01
	**dyschromatosis universalis**	11	0.01
	**ichthyosis hystrix**	11	0.01
	**erythrodermia exfoliativa leiner**	9	0.01
	**gargoylism**	9	0.01
	**knuckle pads**	9	0.01
	**leprechaunism syndrome**	9	0.01
	**phakomatosis pigmentovascularis**	7	0.01
	**harlequim ichthyosis**	6	0.00
**Inflammatory**	**acne vulgaris**	2713	1.96
	**psoriasis**	1575	1.14
	**lichen planus**	1345	0.97
	**rosacea**	1150	0.83
	**scabies**	1082	0.78
	**herpes zoster**	1076	0.78
	**eczema**	1073	0.77
	**erythema multiforme and fixed drug eruptions**	962	0.69
	**leprosy**	953	0.69

**seborrheic dermatitis**	951	0.69
**photodermatoses**	947	0.68
**drug eruption**	946	0.68
**pigmentation diseases**	929	0.67
**syphilis**	918	0.66
**lichen**	914	0.66
**allergic contact dermatitis**	905	0.65
**onychomycosis**	895	0.65
**vasculitis**	843	0.61
**scarring**	804	0.58
**molluscum contagiosum**	788	0.57
**non scarring alopecia**	782	0.56
**impetigo**	778	0.56
**depositional diseases calcinosis cutis amyloid mucinosis gout and others**	776	0.56
**sarcoidosis**	772	0.56
**sunburn**	764	0.55
**atrophic skin disease striae and elastic fiber disorders**	747	0.54
**urticaria**	746	0.54
**alopecia**	731	0.53
**granuloma annulare**	731	0.53
**tinea corporis**	731	0.53
**fungal infections dermal and subcut**	718	0.52
**hair disorderstrichorrhexis nodosa etc**	709	0.51
**scarring alopecia**	703	0.51
**tick bite**	667	0.48
**pityriasis rosea**	661	0.48
**folliculitis**	656	0.47
**pustular skin disease non infectious**	656	0.47
**malformations of extremities**	653	0.47
**dermatomyositis**	629	0.45
**candidiasis**	581	0.42
**bullous pemphigoid**	580	0.42

**xanthomas**	545	0.39
**tinea pedis**	538	0.39
**histiocytic disorders and proliferations**	529	0.38
**lichen sclerosus skin**	523	0.38
**perioral dermatitis**	520	0.38
**gyrate erythemas**	510	0.37
**factitial dermatitis**	488	0.35
**pityriasis rubra pilaris**	487	0.35
**lupus acute**	477	0.34
**scleroderma**	476	0.34
**nematode infection**	462	0.33
**stasis ulcer**	459	0.33
**keratosis pilaris and related follicular disorders**	454	0.33
**atopic dermatitis**	451	0.33
**mastocytosis**	448	0.32
**lichen simplex**	447	0.32
**diaper dermatitis**	435	0.31
**panniculitis lipodystrophy and diseases of subcutis**	430	0.31
**hidradenitis**	426	0.31
**herpes simplex**	424	0.31
**toxic epidermal necrolysis and stevens johnson**	424	0.31
**dyshidrotic eczema**	416	0.30
**rhinophyma**	392	0.28
**metabolic and nutritional diseases**	390	0.28
**cheilitis**	385	0.28
**pediculosis lids**	382	0.28
**dermatitis herpetiformis**	376	0.27
**acanthosis nigricans and carp**	367	0.26
**atopic dermatitis feet**	367	0.26
**morphea**	360	0.26
**necrobiosis lipoidica**	359	0.26

**psoriasis chronic plaque**	355	0.26
**porphyria**	354	0.26
**varicella**	353	0.25
**atopic dermatitis adult phase**	345	0.25
**cutaneous manifestations of systemic disease**	328	0.24
**lupus subacute**	327	0.24
**dry skin eczema**	322	0.23
**pemphigus vulgaris**	322	0.23
**condyloma acuminata**	308	0.22
**pityriasis versicolor**	307	0.22
**erysipelas**	301	0.22
**eczema hand**	300	0.22
**virus exanthem**	297	0.21
**pityriasis lichenoides and pleva**	294	0.21
**genital warts**	293	0.21
**pemphigus**	293	0.21
**hand foot mouth**	288	0.21
**tinea capitis**	286	0.21
**tinea versicolor**	286	0.21
**stasis dermatitis**	284	0.21
**sun damaged skin**	282	0.20
**contact dermatitis**	280	0.20
**solar elastosis**	276	0.20
**erythroderma**	267	0.19
**shoe allergy**	266	0.19
**eczema herpeticum**	255	0.18
**diseases of the ear**	250	0.18
**eczema nummular**	246	0.18
**paronychia**	245	0.18
**reiters disease**	240	0.17
**tinea cruris**	235	0.17
**pigmented purpuric dermatosis**	228	0.16

**pernio and mimics**	226	0.16
**discoid lupus erythematosus**	224	0.16
**tinea ringworm body**	219	0.16
**irritant contact dermatitis**	218	0.16
**gianotti crosti**	215	0.16
**livedo reticularis**	214	0.15
**tuberculosis**	214	0.15
**insect bite**	211	0.15
**solar purpura**	211	0.15
**biting fleas**	209	0.15
**pityrosporum folliculitis**	206	0.15
**solar damage atrophy**	206	0.15
**nummular eczema**	205	0.15
**onycholysis**	205	0.15
**neurotic excoriations**	204	0.15
**tinea ringworm versicolor**	203	0.15
**pitted keratolysis**	202	0.15
**tungiasis**	202	0.15
**acne cystic**	198	0.14
**atopic eczema**	194	0.14
**psoriasis palms soles**	192	0.14
**planar warts**	185	0.13
**acne keloidalis**	184	0.13
**misuse dermatitis**	184	0.13
**rhus dermatitis**	181	0.13
**lupus chronic cutaneous**	180	0.13
**behcets disease**	179	0.13
**nail psoriasis**	177	0.13
**furuncle**	173	0.12
**intertrigo**	170	0.12
**neurodermatitis**	170	0.12
**stasis dermatitis and ulcers**	169	0.12
**warts**	168	0.12

**eczema fingertips**	166	0.12
**verruca vulgaris**	165	0.12
**ulcer**	161	0.12
**psoriasis nails**	160	0.12
**grovers disease**	159	0.11
**tattoo and foreign body reactions**	159	0.11
**lichen nitidus**	157	0.11
**perforating disorders**	156	0.11
**cellulitis**	154	0.11
**herpes type recurrent**	154	0.11
**scleromyxedema**	154	0.11
**herpes gestationis**	153	0.11
**scarlet fever**	149	0.11
**stasis edema**	148	0.11
**tinea manus**	146	0.11
**balanitis**	143	0.10
**psoriasis scalp**	143	0.10
**leukocytoclastic vasculitis**	142	0.10
**warts vulgaris**	141	0.10
**herpes type primary**	139	0.10
**clubbing**	138	0.10
**ashy dermatosis**	137	0.10
**hypereosinophilic syndrome**	134	0.10
**radiodermatitis**	134	0.10
**staphylococcal scalded skin syndrome**	132	0.10
**bacterial infections**	130	0.09
**herpes cutaneous**	130	0.09
**ingrown nail**	129	0.09
**pemphigus foliaceous**	128	0.09
**id reaction**	127	0.09
**psoriasis guttate**	126	0.09
**solar damage favre racouchot**	126	0.09
**tinea ringworm groin**	126	0.09

**angioedema**	125	0.09
**beaus lines**	125	0.09
**subacute cutaneous lupus erythematosus**	125	0.09
**dermographia**	124	0.09
**acquired autoimmune bullous diseaseherpes gestationis**	122	0.09
**lupus vulgaris**	122	0.09
**atopic dermatitis infant phase**	120	0.09
**median nail dystrophy**	120	0.09
**erythrasma**	119	0.09
**eczema subacute**	118	0.09
**henoch schonlein purpura**	118	0.09
**patch testing**	118	0.09
**pincer nails**	118	0.09
**tinea faciale**	118	0.09
**erythema ab igne**	117	0.08
**pemphigus foliaceus**	117	0.08
**warts common**	117	0.08
**subacute cutaneous lupus erythematosus scle**	116	0.08
**warts plantar**	116	0.08
**chronic recurrent aphthae**	115	0.08
**inverse psoriasis**	114	0.08
**diabetic ulcer**	113	0.08
**lupus erythematosus chronicus discoides**	112	0.08
**lupus erythematosus systemic**	112	0.08
**tinea faciei**	112	0.08
**atopic dermatitis childhood phase**	111	0.08
**lymphedema**	111	0.08
**measles**	110	0.08
**eczema foot**	109	0.08
**thermal burn**	109	0.08
**erythema infectiosum**	107	0.08
**myiasis**	107	0.08

**nail diseases**	107	0.08
**pseudofolliculitis barbae**	107	0.08
**tinea nigra**	107	0.08
**raynauds disease**	106	0.08
**crest syndrome**	105	0.08
**herpes simplex analis**	105	0.08
**keratolysis exfoliativa**	105	0.08
**miliaria**	105	0.08
**steroid acne**	105	0.08
**enterovirus**	104	0.08
**geographic tongue**	104	0.08
**papulose bowenoide**	104	0.08
**purpura**	104	0.08
**wart**	102	0.07
**rosacea nose**	101	0.07
**urticaria acute**	100	0.07
**acne pustular**	99	0.07
**atypical mycobacterium**	99	0.07
**orf**	99	0.07
**poikiloderma civatte**	98	0.07
**acne excoriee**	97	0.07
**psoriasis hand**	97	0.07
**spider bite**	97	0.07
**psoriasis inversa**	95	0.07
**neurotrophic ulcer**	93	0.07
**tinea ringworm face**	93	0.07
**habit tic deformity**	89	0.06
**pseudomonas**	89	0.06
**progressive systemic scleroderma**	88	0.06
**epidermodysplasia verruciformis**	87	0.06
**scalp psoriasis**	87	0.06
**acne conglobata**	85	0.06
**erythema elevatum diutinum**	84	0.06

**terry nails**	83	0.06
**tinea ringworm foot webs**	82	0.06
**leukonychia**	81	0.06
**myxoedema pretibial**	80	0.06
**splinter hemorrhage**	80	0.06
**wet to dry foot syndrome**	80	0.06
**chronic bullous disease of childhood**	79	0.06
**pigmented bands**	79	0.06
**warts flat**	79	0.06
**tinea ringworm scalp**	78	0.06
**acne closed comedo**	77	0.06
**diseases of the eye**	77	0.06
**urticarial vasculitis**	77	0.06
**tinea ringworm hand dorsum**	76	0.05
**acne open comedo**	75	0.05
**hairy tongue**	75	0.05
**lichen planus oral**	74	0.05
**psoriasis treatment**	74	0.05
**epidermolysis bullosa acquisita**	73	0.05
**guttate psoriasis**	73	0.05
**nail dystrophy**	73	0.05
**gonorrhea**	72	0.05
**tinea ringworm laboratory**	72	0.05
**chancroid**	71	0.05
**cimicosis**	71	0.05
**herpetic sycosis**	71	0.05
**hyperhidrosis**	71	0.05
**koilonychia**	71	0.05
**rheumatoid nodule**	71	0.05
**lichen striatus**	70	0.05
**tinea ringworm foot dorsum**	70	0.05
**herpes simplex labialis**	69	0.05
**pachyonychia congenita**	68	0.05

**relapsing polychondritis**	68	0.05
**desquamation**	67	0.05
**pemphigus vegetans**	67	0.05
**psoriasis vulgaris chronic stationary type**	67	0.05
**tinea facei**	67	0.05
**diseases of bone**	66	0.05
**eosinophilic pustular folliculitis**	65	0.05
**erythema craquele**	65	0.05
**fibromatosis**	64	0.05
**panarteritis nodosa kussmaul maier**	64	0.05
**candidiasis chronic mucocutaneos**	63	0.05
**warts periungual**	63	0.05
**atrophy blanche**	62	0.04
**ecthyma simplex**	62	0.04
**onychogryphosis**	62	0.04
**twenty nail dystrophy**	62	0.04
**acne scar**	61	0.04
**hidroa vacciniforme**	61	0.04
**lupus erythematosus subacute cutaneous lupus erythematosus**	60	0.04
**sjogrens syndrome**	60	0.04
**solar damage telangiectasia**	60	0.04
**warts digitate**	60	0.04
**localized scleroderma**	59	0.04
**cholinergic urticaria**	58	0.04
**pemphigus mucosae**	57	0.04
**tinea incognito**	57	0.04
**tinea ringworm primary lesion**	57	0.04
**acne fulminans**	56	0.04
**green nail**	56	0.04
**lichen planus penis**	55	0.04
**nail trauma**	55	0.04
**tinea ringworm incognito**	55	0.04

**angiolymphoid hyperplasia with eosinophilia**	54	0.04
**kawasaki syndrome**	54	0.04
**psoriasis vulgaris guttate type**	54	0.04
**warts cryotherapy**	54	0.04
**dermatoliposclerosis**	53	0.04
**acne papulopustulosa**	52	0.04
**eczema chronic**	52	0.04
**piedra white**	52	0.04
**stomatitis**	52	0.04
**trichomycosis**	52	0.04
**caterpillar dermatitis**	51	0.04
**fissure**	51	0.04
**parapsoriasis en plaques**	51	0.04
**tinea ringworm foot plantar**	51	0.04
**tinea ringworm palm**	51	0.04
**abrasion**	50	0.04
**chronic venous insufficiency grade ii**	50	0.04
**lichen planus actinicus**	50	0.04
**lichen planus of the mucosa**	50	0.04
**acne inversa**	49	0.04
**meningococcemia**	49	0.04
**psoriasis palmoplantaris**	49	0.04
**acne cystica**	48	0.03
**lichen planus follicularis**	48	0.03
**maceration**	48	0.03
**skin diseases of pregnancy**	47	0.03
**acne rosacea**	46	0.03
**cicatricial pemphigoid**	46	0.03
**erosio interdigitalis blastomycetica**	46	0.03
**granuloma**	46	0.03
**lichen planus linearis**	46	0.03
**morphea linear**	46	0.03

**nail pigmentation**	46	0.03
**psoriatic erythroderma**	46	0.03
**reticular erythematous mucinosis**	46	0.03
**warts filiformis**	46	0.03
**lichen planus of the mucosa erosive**	45	0.03
**staphylococcal folliculitis**	45	0.03
**cercaria dermatitis**	44	0.03
**eczema arms**	44	0.03
**eczema leg**	44	0.03
**herpes buttocks**	44	0.03
**psoriasis penis**	44	0.03
**rocky mountain spotted fever**	44	0.03
**fox fordyce disease**	43	0.03
**tylotic dermatitis**	43	0.03
**degos disease**	42	0.03
**lichen planopilaris**	42	0.03
**nail beading**	42	0.03
**cutis marmorata**	41	0.03
**diabetic bullae**	41	0.03
**folliculitis cheloidalis**	41	0.03
**infected eczema**	41	0.03
**pseudomonas folliculitis**	41	0.03
**dermatitis swimming**	40	0.03
**psoriasis vulgaris nail changes**	40	0.03
**thrombocytopenic purpura idiopatic**	40	0.03
**half and half nail**	39	0.03
**lichen planus drug**	39	0.03
**nodulo cystic acne vulgaris**	39	0.03
**psoriasis arthropathica**	39	0.03
**acne infantile**	38	0.03
**cat scratch disease**	38	0.03
**eczema face**	38	0.03
**infantile atopic dermatitis**	38	0.03

**nail polish dermatitis**	38	0.03
**psoriasis inversus**	38	0.03
**schistosomiasis cutaneous**	38	0.03
**trichostasis spinulosa**	38	0.03
**urticaria vasculitis**	38	0.03
**candidiasis mouth**	37	0.03
**gangrene**	37	0.03
**rosacea steroid**	37	0.03
**lichen planus annular**	36	0.03
**metal dermatitis**	36	0.03
**peripheral occlusive arterial disease**	36	0.03
**roseola infantum**	36	0.03
**scrophuloderma**	36	0.03
**cryoglobulinemia**	35	0.03
**eczema areola**	35	0.03
**gingival disease**	35	0.03
**herpes simplex genitalis**	35	0.03
**linear scleroderma**	35	0.03
**purpura fulminans**	35	0.03
**ridging beading**	35	0.03
**varicosis**	35	0.03
**acroangiodermatitis**	34	0.02
**herpes simplex virus**	34	0.02
**pomade acne**	34	0.02
**tinea barbae**	34	0.02
**erysipeloid**	33	0.02
**herpetic gingivostomatite**	33	0.02
**morphoea generalized**	33	0.02
**small pox**	33	0.02
**wound infection**	33	0.02
**acne neonatorum**	32	0.02
**connective tissue disorders**	32	0.02
**ischemia**	32	0.02

**acute eczema**	31	0.02
**eczematid**	31	0.02
**fourniers gangrene**	31	0.02
**painful bruising syndrome**	31	0.02
**scrotal tongue**	31	0.02
**thrombangiitis obliterans**	31	0.02
**dermatitis ekzema**	30	0.02
**wells syndrome**	30	0.02
**lichen planus hypertrophic**	29	0.02
**lichen planus vesicular**	29	0.02
**pemphigoid bullous pemphigoid**	29	0.02
**sycosis barbae**	29	0.02
**tinea manuum**	29	0.02
**verrucae planae juveniles**	29	0.02
**axillary granular parakeratosis**	28	0.02
**lupus erythematosus chronicus verrucous**	28	0.02
**nail cosmesis**	28	0.02
**psoriasis anus**	28	0.02
**acanthoma fissuratum**	27	0.02
**dry nails**	27	0.02
**lichen planus exanthematicus**	27	0.02
**solar damage cutis rhomboidalis nuchae**	27	0.02
**torus palantine**	27	0.02
**warts mouth**	27	0.02
**distal splitting hang nail**	26	0.02
**eczema lids**	26	0.02
**granuloma multiforme**	26	0.02
**malassezia folliculitis**	26	0.02
**decubitus ulcer**	25	0.02
**diabetic gangrene**	25	0.02
**eczema acute**	25	0.02
**eczema trunk generalized**	25	0.02
**nail ridging**	25	0.02

**onychoschizia**	25	0.02
**interstitial granulomatous dermatitis**	24	0.02
**racquet nail**	24	0.02
**tinea ringworm beard**	24	0.02
**benign mucosal pemphigoid**	23	0.02
**eosinophilic ulcer of the tongue**	23	0.02
**pyoderma faciale**	23	0.02
**erysipelas bullosum**	22	0.02
**mucous membrane psoriasis**	22	0.02
**notalgia paraesthetica**	22	0.02
**streptococci anal penis**	22	0.02
**urticaria factitia**	22	0.02
**warts oral**	22	0.02
**gloves and socks syndrome**	21	0.02
**heels dry cracked**	21	0.02
**lupus erythematosus tumidus**	21	0.02
**pincer nail syndrome**	21	0.02
**eczema anal**	20	0.01
**hecks disease**	20	0.01
**acne necrotica**	19	0.01
**culicosis bullosa**	19	0.01
**eczema ears**	19	0.01
**lichen planus verrucosus**	19	0.01
**lupus erythematosus chronicus disseminatus superficialis**	19	0.01
**palmar wart**	19	0.01
**atopic eczema of the hands**	18	0.01
**disseminated lokalized scleroderma**	18	0.01
**erythromelalgia**	18	0.01
**gram negative infection of the foot**	18	0.01
**localized pemphigoid**	18	0.01
**nail shedding**	18	0.01
**warts immunocompromised**	18	0.01

ant bite	17	0.01
dermatosis neglecta	17	0.01
glanzmanns thrombasthenia	17	0.01
morphoea nodular	17	0.01
morsicatio buccarum	17	0.01
onychodystrophia mediana canaliformis	17	0.01
psoriasis light sensative	17	0.01
psoriasis nail psoriasis	17	0.01
acne comedonica	16	0.01
leukoedema oral	16	0.01
parasitoses bites	16	0.01
allergic hand eczema	15	0.01
balanitis bacterial	15	0.01
eczema impetiginized	15	0.01
eczema scrotum	15	0.01
eczema vulva	15	0.01
psoriasis infants	15	0.01
warts plantaris	15	0.01
atopic dermatitis hyperlinear creases	14	0.01
lichen planus vaginal	14	0.01
mononucleosis ampicillin syndrome	14	0.01
warts plane	14	0.01
cosmetic fragrance allergy	13	0.01
elephantiasis nostras verrucosa	13	0.01
lupus miliaris disseminatus faciei	13	0.01
morphoea localized	13	0.01
uvl burn	13	0.01
wound dehiscence	13	0.01
alopecia acquisitum centrifugum	12	0.01
hematogenous allergic contact dermatitis	12	0.01
herpes immunocompromised	12	0.01
herpes simplex oralis	12	0.01

**hyperkeratotic fissured hand and foot eczema**	12	0.01
**lupus erythematosus mucosae oris**	12	0.01
**polychondritis**	12	0.01
**pterygium inversum unguis**	12	0.01
**syringolymphoid hyperplasia with alopecia**	12	0.01
**tinea ringworm border**	12	0.01
**lichen planus palm soles**	11	0.01
**pachyonychia congenita hereditaria**	11	0.01
**phlebitis superficial**	11	0.01
**psoriasis hiv**	11	0.01
**blue nails minocycline**	10	0.01
**chronic cutaneous graft versus host reaction**	10	0.01
**folliculitis of barbae**	10	0.01
**lichen planus annularis**	10	0.01
**lymphangitis acute lymphangitis**	10	0.01
**onychogryphosis and onychauxis**	10	0.01
**primary tufted hairs**	10	0.01
**ulcus cruris mixtum**	10	0.01
**contact dermatitis leg ulcers**	9	0.01
**lingua plicata**	9	0.01
**pemphigus foliaceus localized form**	9	0.01
**portuguese man of war dermatitis**	9	0.01
**psoriasis psoriasis after erysipelas**	9	0.01
**shaving folliculitis**	9	0.01
**ulcus cruris venosum**	9	0.01
**zoster gangraenosus**	9	0.01
**eczema microbic**	8	0.01
**pemphigus seborrhoicus**	8	0.01
**pemphigus vegitant**	8	0.01
**tinea barbae profunda**	8	0.01
**drug lichenoid eruption**	7	0.01
**primary herpes simplex virus**	7	0.01

	superficial fungal infections	7	0.01
	lipoatrophy postherpertic lipoatrophy	6	0.00
	psoriasis digits	6	0.00
	tinea capitis profunda	6	0.00
	tinea capitis superficialis	6	0.00
	autoerythrocyte sensitization	5	0.00
	lupus erythematosus systemic lupus erythematosus	5	0.00
	parasitosis psychogenic	5	0.00
	natural rubber latex allergy	4	0.00
	tinea ringworm nigra	4	0.00
	cow pox	3	0.00
	lichen planus hypertrophicus	3	0.00
	cat pox	1	0.00
Malignant dermal tumor	systemic carcinomas	512	0.37
	intermediate or malignant vascular tumors	372	0.27
	dermatofibrosarcoma protuberans	102	0.07
	merkel cell carcinoma	71	0.05
	sarcoma	66	0.05
	abrikossofs tumour of skin	22	0.02
	malignant adnexal tumors	21	0.02
	soft tissue tumors	15	0.01
	adenocarcinoma in the submandibular salivary glands	5	0.00
Pigmented lesions benign	melanocytic nevi	1513	1.09
	blue nevus	694	0.50
	atypical nevi	528	0.38
	intradermal nevus	439	0.32
	nevus cell nevus	332	0.24
	congenital nevus	267	0.19
	halo nevus	237	0.17
	solar lentigo	205	0.15
	nevocytic nevus	183	0.13
	nevus spilus	108	0.08

	nevus incipiens	105	0.08
	café au lait spot	90	0.06
	melanosis neurocutanea	78	0.06
	mongolian spot	67	0.05
	giant melanocytic nevus	62	0.04
	black heel	61	0.04
	naevus blue	57	0.04
	melanoma mimic	52	0.04
	combined nevus	51	0.04
	melanonychia striata	51	0.04
	blue nevus ota	48	0.03
	mucosal lentigines	47	0.03
	naevus halo	45	0.03
	spitz nevus	40	0.03
	ephelides	38	0.03
	hereditary dysplastic nevus syndrome	37	0.03
	spindle cell nevus	36	0.03
	melanotic macule	33	0.02
	naevus achromic	33	0.02
	lentigo simplex	29	0.02
	lentigo senilis	27	0.02
	nevus pigmentosus et pilosus	27	0.02
	nail nevus	22	0.02
	nevus repigmented	22	0.02
	ink spot nevus	16	0.01
	naevus of ota bilateral type	15	0.01
	naevus giant pigmented	11	0.01
	labial melanotic macule	7	0.01
Pigmented lesions malignant	malignant melanoma	1890	1.36
	superficial spreading melanoma ssm	312	0.23
	lentigo maligna	216	0.16
	lentigo maligna melanoma	122	0.09
	nodular melanoma nm	78	0.06

	**metastatic melanoma**	68	0.05
	**amelanotic malignant melanoma amm**	61	0.04
	**nail melanoma**	61	0.04
	**acrolentiginous melanoma alm**	44	0.03
	**malignant melanoma metastatic**	22	0.02
	**mucosal melanoma**	12	0.01
	**melanoma of the parotid**	11	0.01
	**Unknown**	7670	5.54
	**All**	138522	100.00

Supplementary Table 2: Frequency Table and False Positive Rates of Diagnoses in the non-MPXV testing cohort

Frequency table of diagnoses and False Positive Rates of evaluated skin lesion images in the repositories and datasets comprising the non-MPXV testing cohort (Esteva, Danderm, DermIS, DermNet, Fitzpatrick 17k, HDA, and PAD-uefs 20)
All, Number of all evaluated skin lesion images in the non-MPXV testing cohort; FPR, False positive rate; MPXV, Mpox virus; n, Number of occurrences of diagnosis; Unknown, Diagnosis unknown or unmapped; %, Percentage of diagnosis in relation to all evaluated skin lesion images in the non-MPXV testing cohort.

Category	Diagnosis	n	%	FPR
**Benign dermal tumors cysts sinuses**	**pilar cyst**	877	0.69	14.25
	**skin tag**	711	0.56	17.86
	**hemangioma**	655	0.52	12.06
	**pyogenic granuloma**	541	0.43	10.54
	**dermatofibroma**	526	0.42	15.97
	**fibroma**	470	0.37	18.51
	**telangiectases**	455	0.36	9.23
	**syringoma**	390	0.31	7.18
	**sebaceous hyperplasia**	362	0.29	8.01
	**fordyce spots**	318	0.25	11.01
	**mucous cyst**	305	0.24	12.79
	**becker nevus**	226	0.18	15.93
	**lymphangioma**	211	0.17	9.48
	**angiokeratomas**	167	0.13	11.98
	**pilomatricoma**	150	0.12	12.67
	**adenoma sebaceum**	131	0.10	12.21
	**venous lake**	123	0.10	6.50
	**port wine stain**	114	0.09	7.89
	**venous malformations**	108	0.09	4.63
	**trichoepithelioma**	101	0.08	9.90
	**granuloma pyogenic**	97	0.08	6.19
	**granulation tissue**	95	0.08	3.16
	**leiomyoma**	90	0.07	6.67
	**myxoid cyst**	84	0.07	4.76

**angioma**	83	0.07	12.05
**hidrocystoma**	83	0.07	7.23
**haemangioma**	76	0.06	19.74
**cherry angioma**	75	0.06	26.67
**hemangioma infancy**	67	0.05	11.94
**nevus lipomatosis**	65	0.05	26.15
**scrotal cyst**	64	0.05	26.56
**spider angioma**	63	0.05	12.70
**apocrine hydrocystoma**	59	0.05	5.08
**steatocystoma multiplex**	58	0.05	17.24
**trichofolliculoma**	54	0.04	18.52
**cylindroma**	50	0.04	12.00
**eccrine poroma**	50	0.04	10.00
**glomus tumor**	50	0.04	10.00
**spider nevus**	49	0.04	16.33
**dilated pore of winer**	48	0.04	14.58
**klippel trenaunay syndrome**	45	0.04	6.67
**cutis marmorata telangiectatica congenita**	43	0.03	6.98
**ganglion**	41	0.03	19.51
**chondroma**	40	0.03	5.00
**nevus anemicus**	37	0.03	8.11
**eruptive vellus hair cysts**	35	0.03	14.29
**acrotrichoma**	34	0.03	23.53
**dermoid cyst**	33	0.03	9.09
**infantile digital fibromatosis**	33	0.03	9.09
**spiradenoma**	33	0.03	6.06
**pilonidal sinus**	32	0.03	21.88
**connective tissue nevus**	30	0.02	6.67
**strawberry hemangioma**	29	0.02	3.45
**neurolemmoma**	28	0.02	7.14
**ectopic respiratory epithelia**	26	0.02	0.00
**nevus flammeus**	25	0.02	16.00

	vascular anomaly	24	0.02	16.67
	collagen nevus	23	0.02	26.09
	dental sinus	22	0.02	18.18
	periungual fibroma	21	0.02	14.29
	endometriosis	20	0.02	30.00
	aponeurotic fibroma	19	0.02	5.26
	hemangioma racemosum	19	0.02	15.79
	myxoma	19	0.02	5.26
	epithelioma adenoides cysticum	18	0.01	11.11
	fibrolipoma	17	0.01	0.00
	arteriovenous malformation	16	0.01	12.50
	lymphatic malformations	16	0.01	6.25
	trichoepithelioma papulosum multiplex	15	0.01	0.00
	angioma serpiginosum	14	0.01	21.43
	cirsoid aneurysm	14	0.01	7.14
	myofibromatosis	13	0.01	23.08
	trichoblastoma	13	0.01	7.69
	tenosynovitis nodular	12	0.01	0.00
	cyst synovial	11	0.01	9.09
	trichoadenoma	10	0.01	10.00
	perifolicular fibroma	8	0.01	0.00
Cutaneous lymphoma and lymphoid infiltrates	cutaneous t cell lymphoma	744	0.59	9.14
	lymphoma b cell	155	0.12	4.52
	lymphomatoid papulosis	122	0.10	11.48
	erythema arciforme et palpabile migrans	27	0.02	7.41
	pseudo lymphoma other types	23	0.02	0.00
	lymphomas lymphocytic infiltrations	12	0.01	0.00
Epidermal tumors hamartomas milia and growths benign	seborrheic keratosis	1257	0.99	8.27
	epidermal nevus	860	0.68	9.19
	porokeratosis	585	0.46	3.59
	seborrheic keratoses ruff	478	0.38	0.42
	milia	427	0.34	16.39

	benign keratosis	414	0.33	7.00
	prurigo nodularis	315	0.25	26.98
	seborrheic keratoses smooth	156	0.12	0.00
	seborrheic keratosis irritated	150	0.12	0.67
	dermatosis papulosa nigra	92	0.07	10.87
	corns	88	0.07	7.95
	callus	76	0.06	5.26
	stucco keratoses	67	0.05	1.49
	acquired digital fibrokeratoma	58	0.05	15.52
	stucco keratosis	35	0.03	5.71
	acral keratosis	34	0.03	11.76
	giant comedo	34	0.03	5.88
	keratosis seborrheic	26	0.02	7.69
	clear cell acanthoma	19	0.02	5.26
	leser trelat sign	19	0.02	0.00
	warty dyskeratoma	11	0.01	0.00
Epidermal tumors pre malignant and malignant	basal cell carcinoma	2430	1.92	11.69
	squamous cell carcinoma	1251	0.99	12.47
	actinic keratosis	1041	0.82	17.77
	solar damage actinic keratosis	951	0.75	4.21
	keratoacanthoma	776	0.61	8.76
	bowens disease	648	0.51	5.71
	cutaneous horn	197	0.16	7.61
	solid cystic basal cell carcinoma	168	0.13	4.76
	actinic cheilitis	153	0.12	0.65
	basal cell carcinoma lesion	138	0.11	2.90
	pagets disease	126	0.10	10.32
	leukoplakia	120	0.09	5.83
	basal cell carcinoma morpheiform	115	0.09	2.61
	arsenical keratoses	83	0.07	12.05
	erythroplasia queyrat	81	0.06	11.11
	basal cell carcinoma nose	79	0.06	5.06
	actinic keratosis face	77	0.06	1.30

**basal cell nevus syndrome**	76	0.06	13.16
**actinic keratosis lesion**	66	0.05	3.03
**basal cell carcinoma superficial**	65	0.05	0.00
**solar damage**	62	0.05	3.23
**actinic keratosis fu**	59	0.05	0.00
**extramammary paget disease**	59	0.05	10.17
**basal cell carcinoma lid**	55	0.04	5.45
**basal cell carcinoma ear**	53	0.04	0.00
**pigmented basal cell carcinoma**	52	0.04	5.77
**basal cell carcinoma face**	50	0.04	6.00
**verrucous carcinoma**	49	0.04	2.04
**actinic keratosis hand**	47	0.04	0.00
**squamous cell carcinoma ear**	43	0.03	0.00
**superficial basal cell carcinoma**	38	0.03	0.00
**basal cell carcinoma ulcerating**	37	0.03	0.00
**nevoid basal cell carcinoma syndrome**	37	0.03	13.51
**basal cell carcinoma lip**	36	0.03	2.78
**solar damage actinex treatment**	36	0.03	2.78
**keratosis solar**	35	0.03	0.00
**squamous cell carcinoma lesion**	34	0.03	5.88
**carcinoma of lip**	32	0.03	6.25
**basal cell carcinoma scalp**	29	0.02	13.79
**squamous cell carcinoma lip**	29	0.02	6.90
**actinic keratosis ear**	28	0.02	0.00
**hypertrophic actinic keratosis**	27	0.02	3.70
**actinic keratosis horn**	26	0.02	3.85
**leucoplakia praecancerosa**	23	0.02	0.00
**ulcus terebrans**	22	0.02	4.55
**solar damage actinic cheilitis**	20	0.02	5.00
**actinic keratosis nose**	17	0.01	0.00
**basal cell carcinoma trunk ext**	15	0.01	13.33
**squamous cell carcinoma nose**	15	0.01	0.00

	actinic keratosis pigmented	14	0.01	7.14
	squamous cell carcinoma hand arm	12	0.01	0.00
	basal cell carcinoma aldara	11	0.01	18.18
	keratoacanthoma pendulum	11	0.01	18.18
	solar damage fu treatment	10	0.01	0.00
	squamous cell carcinoma face	10	0.01	0.00
	basal cell carcinoma sclerosing	1	0.00	0.00
Genodermatoses and supernumerary growths	neurofibromatosis	707	0.56	19.09
	epidermolysis bullosa	589	0.47	18.34
	dariers disease	530	0.42	6.42
	ichthyosis	518	0.41	25.87
	tuberous sclerosis	341	0.27	16.13
	hailey hailey disease	286	0.23	4.20
	incontinentia pigmenti	224	0.18	16.96
	ichthyosis vulgaris	215	0.17	13.02
	ehlers danlos syndrome	199	0.16	47.74
	rendu osler syndrome	128	0.10	16.41
	acrodermatitis enteropathica	127	0.10	15.75
	xeroderma pigmentosum	118	0.09	13.56
	pseudoxanthoma elasticum	97	0.08	9.28
	epidermolytic hyperkeratosis	86	0.07	4.65
	supernumerary growths	77	0.06	25.97
	collodion baby	75	0.06	21.33
	ichthyosis congenita	70	0.06	2.86
	vascular tumors and malformations	67	0.05	10.45
	erythrokeratodermia variabilis	65	0.05	15.38
	erythrokeratodermia progressive symmetric	63	0.05	14.29
	ichthyosis sex linked	62	0.05	0.00
	lipoid proteinosis urbach wiethe disease	58	0.05	13.79
	x linked ichthyosis	55	0.04	16.36
	leopard syndrome	54	0.04	14.81
	ichthyosis other forms	42	0.03	19.05

	**congenital ichthyosiform erythroderma**	41	0.03	2.44
	**peutz jeghers syndrome**	41	0.03	9.76
	**rothmund thomson syndrome**	41	0.03	7.32
	**acrokeratoelastoidosis**	40	0.03	5.00
	**ichthyosis dominant**	38	0.03	0.00
	**acrokeratosis verruciformis**	36	0.03	0.00
	**albinism**	34	0.03	11.76
	**muir torre syndrome**	34	0.03	8.82
	**congenital erythropoietic porphyria**	29	0.02	10.34
	**congenital dyskeratosis**	27	0.02	14.81
	**birt hogg dube**	25	0.02	4.00
	**buschke ollendorf syndrome**	25	0.02	12.00
	**cowden disease**	23	0.02	8.70
	**congenital anomalies**	22	0.02	0.00
	**sjgren larsson syndrome**	17	0.01	5.88
	**laugier hunziker s syndrome**	15	0.01	6.67
	**lamellar icthyosis**	13	0.01	30.77
	**dyschromatosis universalis**	11	0.01	45.45
	**ichthyosis hystrix**	11	0.01	9.09
	**erythrodermia exfoliativa leiner**	9	0.01	11.11
	**gargoylism**	9	0.01	33.33
	**leprechaunism syndrome**	9	0.01	0.00
	**phakomatosis pigmentovascularis**	7	0.01	0.00
	**harlequim ichthyosis**	6	0.00	16.67
	**knuckle pads**	6	0.00	0.00
**Inflammatory**	**acne vulgaris**	2273	1.80	16.01
	**psoriasis**	1404	1.11	7.48
	**lichen planus**	1229	0.97	6.92
	**rosacea**	1110	0.88	7.57
	**eczema**	1066	0.84	16.51
	**herpes zoster**	1036	0.82	11.58
	**scabies**	994	0.79	13.08

**leprosy**	942	0.74	19.75
**erythema multiforme and fixed drug eruptions**	935	0.74	11.34
**pigmentation diseases**	929	0.73	15.72
**drug eruption**	911	0.72	7.14
**lichen**	911	0.72	10.76
**photodermatoses**	909	0.72	8.80
**seborrheic dermatitis**	890	0.70	9.89
**onychomycosis**	882	0.70	2.15
**allergic contact dermatitis**	864	0.68	6.48
**syphilis**	834	0.66	16.91
**vasculitis**	811	0.64	8.01
**non scarring alopecia**	782	0.62	10.87
**depositional diseases calcinosis cutis amyloid mucinosis gout and others**	776	0.61	13.40
**sunburn**	754	0.60	26.92
**scarring**	750	0.59	10.67
**atrophic skin disease striae and elastic fiber disorders**	747	0.59	16.20
**impetigo**	740	0.59	13.78
**sarcoidosis**	720	0.57	7.50
**fungal infections dermal and subcut**	718	0.57	13.51
**tinea corporis**	716	0.57	9.08
**hair disorderstrichorrhexis nodosa etc**	709	0.56	10.86
**alopecia**	704	0.56	23.15
**molluscum contagiosum**	703	0.56	27.31
**scarring alopecia**	703	0.56	9.82
**urticaria**	683	0.54	10.98
**granuloma annulare**	679	0.54	5.89
**tick bite**	667	0.53	26.39
**pustular skin disease non infectious**	656	0.52	5.64
**malformations of extremities**	653	0.52	7.20
**folliculitis**	639	0.51	22.54
**pityriasis rosea**	606	0.48	9.08

**candidiasis**	551	0.44	7.80
**dermatomyositis**	540	0.43	7.96
**bullous pemphigoid**	524	0.41	11.45
**histiocytic disorders and proliferations**	523	0.41	15.11
**tinea pedis**	515	0.41	4.27
**gyrate erythemas**	510	0.40	8.04
**perioral dermatitis**	504	0.40	3.77
**xanthomas**	493	0.39	15.01
**factitial dermatitis**	488	0.39	12.09
**lichen sclerosus skin**	483	0.38	11.18
**pityriasis rubra pilaris**	480	0.38	4.79
**lupus acute**	477	0.38	19.50
**scleroderma**	476	0.38	14.71
**nematode infection**	462	0.37	10.39
**stasis ulcer**	459	0.36	2.83
**keratosis pilaris and related follicular disorders**	441	0.35	4.76
**mastocytosis**	430	0.34	7.44
**dyshidrotic eczema**	416	0.33	4.81
**lichen simplex**	416	0.33	12.74
**panniculitis lipodystrophy and diseases of subcutis**	412	0.33	12.62
**herpes simplex**	406	0.32	12.32
**diaper dermatitis**	401	0.32	11.47
**metabolic and nutritional diseases**	390	0.31	22.31
**cheilitis**	385	0.30	6.75
**rhinophyma**	383	0.30	7.57
**pediculosis lids**	382	0.30	9.95
**dermatitis herpetiformis**	370	0.29	13.24
**atopic dermatitis feet**	367	0.29	4.63
**necrobiosis lipoidica**	355	0.28	4.51
**atopic dermatitis adult phase**	345	0.27	2.61
**porphyria**	345	0.27	11.01

**acanthosis nigricans and carp**	344	0.27	12.50
**hidradenitis**	339	0.27	22.42
**psoriasis chronic plaque**	337	0.27	2.37
**morphea**	336	0.27	8.63
**cutaneous manifestations of systemic disease**	328	0.26	16.77
**lupus subacute**	327	0.26	8.87
**toxic epidermal necrolysis and stevens johnson**	320	0.25	9.38
**dry skin eczema**	317	0.25	3.79
**varicella**	312	0.25	34.62
**condyloma acuminata**	308	0.24	16.88
**eczema hand**	300	0.24	1.00
**erysipelas**	294	0.23	8.84
**pemphigus**	292	0.23	9.93
**genital warts**	286	0.23	17.13
**tinea versicolor**	286	0.23	9.79
**stasis dermatitis**	284	0.22	2.11
**sun damaged skin**	282	0.22	9.57
**solar elastosis**	274	0.22	10.58
**pemphigus vulgaris**	273	0.22	5.86
**shoe allergy**	266	0.21	25.19
**virus exanthem**	265	0.21	5.28
**pityriasis versicolor**	262	0.21	9.92
**contact dermatitis**	261	0.21	4.60
**tinea capitis**	261	0.21	6.90
**pityriasis lichenoides and pleva**	252	0.20	13.49
**diseases of the ear**	250	0.20	6.80
**erythroderma**	243	0.19	6.17
**eczema nummular**	241	0.19	3.32
**reiters disease**	240	0.19	7.92
**eczema herpeticum**	236	0.19	8.90
**paronychia**	236	0.19	2.12
**atopic dermatitis**	231	0.18	8.66

**pigmented purpuric dermatosis**	228	0.18	4.82
**pernio and mimics**	226	0.18	7.96
**tinea cruris**	226	0.18	24.78
**tinea ringworm body**	219	0.17	9.13
**hand foot mouth**	214	0.17	20.09
**irritant contact dermatitis**	213	0.17	5.63
**livedo reticularis**	211	0.17	6.16
**solar purpura**	211	0.17	5.21
**biting fleas**	209	0.17	11.96
**pityrosporum folliculitis**	206	0.16	11.65
**solar damage atrophy**	206	0.16	16.02
**neurotic excoriations**	204	0.16	20.10
**onycholysis**	203	0.16	2.46
**tinea ringworm versicolor**	203	0.16	6.90
**tungiasis**	202	0.16	15.84
**tuberculosis**	199	0.16	10.05
**acne cystic**	198	0.16	6.57
**psoriasis palms soles**	192	0.15	2.60
**planar warts**	185	0.15	5.95
**misuse dermatitis**	184	0.15	7.61
**pitted keratolysis**	181	0.14	4.42
**rhus dermatitis**	181	0.14	0.55
**acne keloidalis**	180	0.14	23.33
**lupus chronic cutaneous**	180	0.14	8.33
**insect bite**	174	0.14	17.24
**atopic eczema**	173	0.14	6.36
**furuncle**	173	0.14	21.97
**discoid lupus erythematosus**	171	0.14	19.30
**neurodermatitis**	170	0.13	7.65
**stasis dermatitis and ulcers**	169	0.13	0.00
**warts**	168	0.13	7.74
**gianotti crosti**	167	0.13	14.97
**eczema fingertips**	166	0.13	0.00

**intertrigo**	166	0.13	14.46
**verruca vulgaris**	165	0.13	12.12
**tattoo and foreign body reactions**	159	0.13	9.43
**perforating disorders**	156	0.12	8.33
**lichen nitidus**	155	0.12	8.39
**herpes type recurrent**	154	0.12	8.44
**scleromyxedema**	148	0.12	4.73
**stasis edema**	148	0.12	4.73
**tinea manus**	146	0.12	1.37
**scarlet fever**	144	0.11	4.17
**balanitis**	143	0.11	6.29
**cellulitis**	143	0.11	9.79
**psoriasis scalp**	143	0.11	4.20
**leukocytoclastic vasculitis**	142	0.11	11.27
**warts vulgaris**	141	0.11	9.93
**behcets disease**	140	0.11	19.29
**herpes type primary**	139	0.11	12.23
**ashy dermatosis**	137	0.11	13.87
**nail psoriasis**	136	0.11	3.68
**grovers disease**	134	0.11	5.97
**clubbing**	132	0.10	3.03
**radiodermatitis**	132	0.10	7.58
**hypereosinophilic syndrome**	131	0.10	4.58
**bacterial infections**	130	0.10	3.08
**herpes cutaneous**	130	0.10	11.54
**ingrown nail**	128	0.10	5.47
**pemphigus foliaceous**	128	0.10	5.47
**id reaction**	127	0.10	5.51
**psoriasis nails**	127	0.10	3.94
**psoriasis guttate**	126	0.10	7.14
**solar damage favre racouchot**	126	0.10	7.14
**tinea ringworm groin**	126	0.10	39.68
**dermographia**	124	0.10	4.84

**acquired autoimmune bullous diseaseherpes gestationis**	122	0.10	8.20
**beaus lines**	122	0.10	5.74
**lupus vulgaris**	121	0.10	6.61
**atopic dermatitis infant phase**	120	0.09	10.83
**median nail dystrophy**	120	0.09	4.17
**eczema subacute**	118	0.09	8.47
**tinea faciale**	118	0.09	14.41
**warts common**	117	0.09	9.40
**patch testing**	116	0.09	12.93
**subacute cutaneous lupus erythematosus scle**	116	0.09	0.86
**warts plantar**	116	0.09	6.90
**chronic recurrent aphthae**	115	0.09	12.17
**erythema ab igne**	114	0.09	7.89
**henoch schonlein purpura**	114	0.09	7.89
**inverse psoriasis**	114	0.09	12.28
**atopic dermatitis childhood phase**	111	0.09	6.31
**pemphigus foliaceus**	111	0.09	0.90
**eczema foot**	109	0.09	0.00
**herpes gestationis**	109	0.09	13.76
**lymphedema**	108	0.09	11.11
**staphylococcal scalded skin syndrome**	108	0.09	14.81
**myiasis**	107	0.08	28.97
**nail diseases**	107	0.08	6.54
**thermal burn**	107	0.08	4.67
**tinea faciei**	107	0.08	11.21
**angioedema**	106	0.08	11.32
**raynauds disease**	106	0.08	5.66
**crest syndrome**	105	0.08	6.67
**herpes simplex analis**	105	0.08	5.71
**subacute cutaneous lupus erythematosus**	105	0.08	20.00
**nummular eczema**	104	0.08	6.73

**papulose bowenoide**	104	0.08	16.35
**purpura**	103	0.08	6.80
**tinea nigra**	103	0.08	4.85
**geographic tongue**	102	0.08	3.92
**erythrasma**	101	0.08	19.80
**miliaria**	101	0.08	4.95
**rosacea nose**	101	0.08	4.95
**lupus erythematosus chronicus discoides**	100	0.08	10.00
**pseudofolliculitis barbae**	100	0.08	17.00
**urticaria acute**	100	0.08	4.00
**acne pustular**	99	0.08	9.09
**pincer nails**	99	0.08	10.10
**erythema infectiosum**	97	0.08	9.28
**psoriasis hand**	97	0.08	3.09
**psoriasis inversa**	95	0.08	4.21
**acne excoriee**	94	0.07	13.83
**steroid acne**	94	0.07	13.83
**neurotrophic ulcer**	93	0.07	10.75
**spider bite**	93	0.07	25.81
**tinea ringworm face**	93	0.07	3.23
**diabetic ulcer**	92	0.07	9.78
**poikiloderma civatte**	92	0.07	2.17
**habit tic deformity**	89	0.07	7.87
**pseudomonas**	89	0.07	4.49
**ulcer**	89	0.07	2.25
**progressive systemic scleroderma**	88	0.07	6.82
**epidermodysplasia verruciformis**	85	0.07	9.41
**acne conglobata**	84	0.07	9.52
**tinea ringworm foot webs**	82	0.06	3.66
**erythema elevatum diutinum**	81	0.06	8.64
**leukonychia**	81	0.06	3.70
**splinter hemorrhage**	80	0.06	7.50

**terry nails**	80	0.06	3.75
**wet to dry foot syndrome**	80	0.06	5.00
**chronic bullous disease of childhood**	79	0.06	21.52
**pigmented bands**	79	0.06	11.39
**warts flat**	79	0.06	3.80
**measles**	78	0.06	6.41
**tinea ringworm scalp**	78	0.06	6.41
**acne closed comedo**	77	0.06	3.90
**diseases of the eye**	77	0.06	2.60
**keratolysis exfoliativa**	77	0.06	0.00
**tinea ringworm hand dorsum**	76	0.06	2.63
**acne open comedo**	75	0.06	2.67
**hairy tongue**	75	0.06	13.33
**psoriasis treatment**	74	0.06	8.11
**nail dystrophy**	73	0.06	8.22
**gonorrhea**	72	0.06	19.44
**scalp psoriasis**	72	0.06	2.78
**tinea ringworm laboratory**	72	0.06	4.17
**cimicosis**	71	0.06	12.68
**herpetic sycosis**	71	0.06	21.13
**hyperhidrosis**	71	0.06	2.82
**enterovirus**	70	0.06	14.29
**koilonychia**	70	0.06	0.00
**tinea ringworm foot dorsum**	70	0.06	0.00
**guttate psoriasis**	69	0.05	14.49
**rheumatoid nodule**	69	0.05	24.64
**chancroid**	68	0.05	17.65
**lichen planus oral**	68	0.05	8.82
**desquamation**	67	0.05	2.99
**pemphigus vegetans**	67	0.05	5.97
**psoriasis vulgaris chronic stationary type**	67	0.05	2.99
**tinea facei**	67	0.05	7.46

**diseases of bone**	66	0.05	4.55
**epidermolysis bullosa acquisita**	65	0.05	15.38
**fibromatosis**	64	0.05	12.50
**lichen striatus**	64	0.05	9.38
**panarteritis nodosa kussmaul maier**	64	0.05	7.81
**candidiasis chronic mucocutaneos**	63	0.05	9.52
**myxoedema pretibial**	63	0.05	7.94
**warts periungual**	63	0.05	3.17
**twenty nail dystrophy**	62	0.05	3.23
**eosinophilic pustular folliculitis**	61	0.05	13.11
**onychogryphosis**	60	0.05	5.00
**solar damage telangiectasia**	60	0.05	1.67
**warts digitate**	60	0.05	0.00
**localized scleroderma**	59	0.05	13.56
**pachyonychia congenita**	59	0.05	8.47
**atypical mycobacterium**	58	0.05	25.86
**pemphigus mucosae**	57	0.05	0.00
**tinea ringworm primary lesion**	57	0.05	3.51
**green nail**	56	0.04	3.57
**hidroa vacciniforme**	56	0.04	12.50
**orf**	56	0.04	42.86
**relapsing polychondritis**	56	0.04	7.14
**sjogrens syndrome**	56	0.04	17.86
**lichen planus penis**	55	0.04	7.27
**nail trauma**	55	0.04	1.82
**tinea ringworm incognito**	55	0.04	10.91
**angiolymphoid hyperplasia with eosinophilia**	54	0.04	16.67
**atrophy blanche**	54	0.04	7.41
**kawasaki syndrome**	54	0.04	12.96
**psoriasis vulgaris guttate type**	54	0.04	3.70
**warts cryotherapy**	54	0.04	3.70
**acne fulminans**	53	0.04	5.66

**dermatoliposclerosis**	53	0.04	5.66
**tinea incognito**	53	0.04	11.32
**acne papulopustulosa**	52	0.04	7.69
**acne scar**	52	0.04	3.85
**ecthyma simplex**	52	0.04	23.08
**eczema chronic**	52	0.04	0.00
**piedra white**	52	0.04	13.46
**trichomycosis**	52	0.04	9.62
**caterpillar dermatitis**	51	0.04	13.73
**erythema craquele**	51	0.04	5.88
**fissure**	51	0.04	0.00
**lupus erythematosus systemic**	51	0.04	9.80
**parapsoriasis en plaques**	51	0.04	3.92
**stomatitis**	51	0.04	9.80
**tinea ringworm foot plantar**	51	0.04	0.00
**tinea ringworm palm**	51	0.04	1.96
**urticarial vasculitis**	51	0.04	3.92
**abrasion**	50	0.04	14.00
**lichen planus actinicus**	50	0.04	34.00
**lichen planus of the mucosa**	50	0.04	2.00
**acne inversa**	49	0.04	14.29
**meningococcemia**	49	0.04	14.29
**acne cystica**	48	0.04	12.50
**lichen planus follicularis**	48	0.04	4.17
**maceration**	48	0.04	6.25
**cholinergic urticaria**	47	0.04	2.13
**skin diseases of pregnancy**	47	0.04	6.38
**acne rosacea**	46	0.04	6.52
**granuloma**	46	0.04	4.35
**lichen planus linearis**	46	0.04	15.22
**morphea linear**	46	0.04	8.70
**nail pigmentation**	46	0.04	8.70
**wart**	46	0.04	8.70

**warts filiformis**	46	0.04	13.04
**erosio interdigitalis blastomycetica**	45	0.04	0.00
**lichen planus of the mucosa erosive**	45	0.04	6.67
**psoriatic erythroderma**	45	0.04	8.89
**staphylococcal folliculitis**	45	0.04	28.89
**cercaria dermatitis**	44	0.03	11.36
**eczema arms**	44	0.03	2.27
**eczema leg**	44	0.03	2.27
**herpes buttocks**	44	0.03	11.36
**psoriasis penis**	44	0.03	0.00
**rocky mountain spotted fever**	44	0.03	11.36
**fox fordyce disease**	43	0.03	9.30
**reticular erythematous mucinosis**	43	0.03	13.95
**tylotic dermatitis**	43	0.03	2.33
**cicatricial pemphigoid**	42	0.03	2.38
**nail beading**	42	0.03	9.52
**folliculitis cheloidalis**	41	0.03	21.95
**pseudomonas folliculitis**	41	0.03	21.95
**degos disease**	40	0.03	25.00
**psoriasis vulgaris nail changes**	40	0.03	0.00
**thrombocytopenic purpura idiopatic**	40	0.03	25.00
**cutis marmorata**	39	0.03	5.13
**lichen planus drug**	39	0.03	2.56
**psoriasis arthropathica**	39	0.03	0.00
**cat scratch disease**	38	0.03	34.21
**chronic venous insufficiency grade ii**	38	0.03	0.00
**diabetic bullae**	38	0.03	5.26
**eczema face**	38	0.03	2.63
**half and half nail**	38	0.03	5.26
**infantile atopic dermatitis**	38	0.03	7.89
**lichen planopilaris**	38	0.03	7.89
**nail polish dermatitis**	38	0.03	0.00
**psoriasis inversus**	38	0.03	18.42

**schistosomiasis cutaneous**	38	0.03	7.89
**trichostasis spinulosa**	38	0.03	2.63
**urticaria vasculitis**	38	0.03	2.63
**gangrene**	37	0.03	10.81
**infected eczema**	37	0.03	8.11
**lichen planus annular**	36	0.03	13.89
**metal dermatitis**	36	0.03	13.89
**peripheral occlusive arterial disease**	36	0.03	0.00
**scrophuloderma**	36	0.03	19.44
**gingival disease**	35	0.03	11.43
**herpes simplex genitalis**	35	0.03	20.00
**linear scleroderma**	35	0.03	17.14
**purpura fulminans**	35	0.03	17.14
**ridging beading**	35	0.03	0.00
**varicosis**	35	0.03	11.43
**acroangiodermatitis**	34	0.03	17.65
**herpes simplex virus**	34	0.03	11.76
**pomade acne**	34	0.03	5.88
**dermatitis swimming**	33	0.03	0.00
**herpetic gingivostomatite**	33	0.03	12.12
**morphoea generalized**	33	0.03	15.15
**small pox**	33	0.03	18.18
**acne infantile**	32	0.03	9.38
**acne neonatorum**	32	0.03	9.38
**candidiasis mouth**	32	0.03	3.12
**connective tissue disorders**	32	0.03	40.62
**eczematid**	31	0.02	3.23
**painful bruising syndrome**	31	0.02	0.00
**scrotal tongue**	31	0.02	3.23
**dermatitis ekzema**	30	0.02	6.67
**erysipeloid**	30	0.02	3.33
**ischemia**	30	0.02	6.67
**lichen planus hypertrophic**	29	0.02	34.48

**lichen planus vesicular**	29	0.02	20.69
**pemphigoid bullous pemphigoid**	29	0.02	10.34
**thrombangiitis obliterans**	29	0.02	0.00
**tinea barbae**	29	0.02	6.90
**verrucae planae juveniles**	29	0.02	13.79
**wells syndrome**	29	0.02	27.59
**acute eczema**	28	0.02	0.00
**cryoglobulinemia**	28	0.02	0.00
**lupus erythematosus chronicus verrucous**	28	0.02	35.71
**nail cosmesis**	28	0.02	7.14
**psoriasis anus**	28	0.02	21.43
**wound infection**	28	0.02	10.71
**dry nails**	27	0.02	11.11
**lichen planus exanthematicus**	27	0.02	7.41
**solar damage cutis rhomboidalis nuchae**	27	0.02	11.11
**torus palantine**	27	0.02	7.41
**warts mouth**	27	0.02	3.70
**distal splitting hang nail**	26	0.02	0.00
**eczema lids**	26	0.02	0.00
**granuloma multiforme**	26	0.02	7.69
**sycosis barbae**	26	0.02	0.00
**decubitus ulcer**	25	0.02	16.00
**diabetic gangrene**	25	0.02	12.00
**eczema acute**	25	0.02	0.00
**eczema trunk generalized**	25	0.02	4.00
**nail ridging**	25	0.02	0.00
**onychoschizia**	25	0.02	0.00
**roseola infantum**	25	0.02	16.00
**acanthoma fissuratum**	24	0.02	0.00
**eczema areola**	24	0.02	0.00
**malassezia folliculitis**	24	0.02	20.83
**racquet nail**	24	0.02	4.17

**tinea ringworm beard**	24	0.02	12.50
**benign mucosal pemphigoid**	23	0.02	0.00
**eosinophilic ulcer of the tongue**	23	0.02	4.35
**tinea manuum**	23	0.02	0.00
**erysipelas bullosum**	22	0.02	0.00
**fourniers gangrene**	22	0.02	4.55
**mucous membrane psoriasis**	22	0.02	13.64
**streptococci anal penis**	22	0.02	13.64
**warts oral**	22	0.02	4.55
**gloves and socks syndrome**	21	0.02	0.00
**heels dry cracked**	21	0.02	23.81
**interstitial granulomatous dermatitis**	21	0.02	4.76
**lupus erythematosus tumidus**	21	0.02	0.00
**pincer nail syndrome**	21	0.02	0.00
**eczema anal**	20	0.02	10.00
**hecks disease**	20	0.02	0.00
**nodulo cystic acne vulgaris**	20	0.02	5.00
**acne necrotica**	19	0.02	15.79
**culicosis bullosa**	19	0.02	5.26
**eczema ears**	19	0.02	10.53
**lichen planus verrucosus**	19	0.02	5.26
**lupus erythematosus chronicus disseminatus superficialis**	19	0.02	5.26
**palmar wart**	19	0.02	15.79
**atopic eczema of the hands**	18	0.01	0.00
**disseminated lokalized scleroderma**	18	0.01	0.00
**gram negative infection of the foot**	18	0.01	5.56
**herpes simplex labialis**	18	0.01	5.56
**localized pemphigoid**	18	0.01	16.67
**nail shedding**	18	0.01	5.56
**pyoderma faciale**	18	0.01	22.22
**rosacea steroid**	18	0.01	0.00
**warts immunocompromised**	18	0.01	0.00

**ant bite**	17	0.01	29.41
**dermatosis neglecta**	17	0.01	0.00
**glanzmanns thrombasthenia**	17	0.01	0.00
**morphoea nodular**	17	0.01	0.00
**morsicatio buccarum**	17	0.01	0.00
**notalgia paraesthetica**	17	0.01	0.00
**onychodystrophia mediana canaliformis**	17	0.01	0.00
**psoriasis light sensative**	17	0.01	5.88
**psoriasis nail psoriasis**	17	0.01	11.76
**acne comedonica**	16	0.01	0.00
**axillary granular parakeratosis**	16	0.01	0.00
**leukoedema oral**	16	0.01	18.75
**parasitoses bites**	16	0.01	31.25
**allergic hand eczema**	15	0.01	0.00
**balanitis bacterial**	15	0.01	6.67
**eczema impetiginized**	15	0.01	0.00
**eczema scrotum**	15	0.01	6.67
**eczema vulva**	15	0.01	0.00
**erythromelalgia**	15	0.01	6.67
**lupus erythematosus subacute cutaneous lupus erythematosus**	15	0.01	6.67
**psoriasis infants**	15	0.01	6.67
**warts plantaris**	15	0.01	0.00
**atopic dermatitis hyperlinear creases**	14	0.01	0.00
**lichen planus vaginal**	14	0.01	7.14
**mononucleosis ampicillin syndrome**	14	0.01	0.00
**warts plane**	14	0.01	0.00
**cosmetic fragrance allergy**	13	0.01	7.69
**elephantiasis nostras verrucosa**	13	0.01	15.38
**lupus miliaris disseminatus faciei**	13	0.01	7.69
**morphoea localized**	13	0.01	0.00
**uvl burn**	13	0.01	0.00
**wound dehiscence**	13	0.01	7.69

**alopecia acquisitum centrifugum**	12	0.01	33.33
**hematogenous allergic contact dermatitis**	12	0.01	8.33
**herpes immunocompromised**	12	0.01	8.33
**herpes simplex oralis**	12	0.01	8.33
**hyperkeratotic fissured hand and foot eczema**	12	0.01	8.33
**lupus erythematosus mucosae oris**	12	0.01	0.00
**polychondritis**	12	0.01	0.00
**pterygium inversum unguis**	12	0.01	0.00
**syringolymphoid hyperplasia with alopecia**	12	0.01	16.67
**lichen planus palm soles**	11	0.01	0.00
**pachyonychia congenita hereditaria**	11	0.01	9.09
**phlebitis superficial**	11	0.01	0.00
**psoriasis hiv**	11	0.01	9.09
**blue nails minocycline**	10	0.01	10.00
**chronic cutaneous graft versus host reaction**	10	0.01	10.00
**folliculitis of barbae**	10	0.01	0.00
**lichen planus annularis**	10	0.01	10.00
**lymphangitis acute lymphangitis**	10	0.01	40.00
**onychogryphosis and onychauxis**	10	0.01	0.00
**primary tufted hairs**	10	0.01	10.00
**ulcus cruris mixtum**	10	0.01	10.00
**contact dermatitis leg ulcers**	9	0.01	44.44
**lingua plicata**	9	0.01	0.00
**pemphigus foliaceus localized form**	9	0.01	11.11
**portuguese man of war dermatitis**	9	0.01	0.00
**psoriasis psoriasis after erysipelas**	9	0.01	11.11
**shaving folliculitis**	9	0.01	11.11
**tinea ringworm border**	9	0.01	0.00
**ulcus cruris venosum**	9	0.01	0.00
**zoster gangraenosus**	9	0.01	0.00
**eczema microbic**	8	0.01	0.00

	pemphigus seborrhoicus	8	0.01	0.00
	pemphigus vegitant	8	0.01	50.00
	psoriasis palmoplantaris	8	0.01	0.00
	tinea barbae profunda	8	0.01	12.50
	drug lichenoid eruption	7	0.01	0.00
	primary herpes simplex virus	7	0.01	28.57
	superficial fungal infections	7	0.01	0.00
	lipoatrophy postherpertic lipoatrophy	6	0.00	0.00
	psoriasis digits	6	0.00	16.67
	tinea capitis profunda	6	0.00	0.00
	tinea capitis superficialis	6	0.00	0.00
	urticaria factitia	6	0.00	0.00
	autoerythrocyte sensitization	5	0.00	20.00
	lupus erythematosus systemic lupus erythematosus	5	0.00	0.00
	parasitosis psychogenic	5	0.00	20.00
	natural rubber latex allergy	4	0.00	0.00
	tinea ringworm nigra	4	0.00	0.00
	cow pox	3	0.00	0.00
	lichen planus hypertrophicus	3	0.00	0.00
	cat pox	1	0.00	0.00
Malignant dermal tumor	systemic carcinomas	512	0.40	9.57
	intermediate or malignant vascular tumors	372	0.29	13.44
	dermatofibrosarcoma protuberans	100	0.08	8.00
	merkel cell carcinoma	66	0.05	3.03
	sarcoma	45	0.04	4.44
	abrikossofs tumour of skin	22	0.02	13.64
	malignant adnexal tumors	21	0.02	9.52
	soft tissue tumors	15	0.01	6.67
	adenocarcinoma in the submandibular salivary glands	5	0.00	0.00
Pigmented lesions benign	melanocytic nevi	1464	1.16	3.96
	blue nevus	682	0.54	4.55

**atypical nevi**	510	0.40	8.04
**intradermal nevus**	439	0.35	8.88
**nevus cell nevus**	301	0.24	14.29
**congenital nevus**	260	0.21	8.08
**halo nevus**	230	0.18	15.65
**nevocytic nevus**	183	0.14	3.83
**solar lentigo**	140	0.11	2.14
**nevus spilus**	108	0.09	6.48
**nevus incipiens**	105	0.08	2.86
**melanosis neurocutanea**	78	0.06	17.95
**café au lait spot**	71	0.06	9.86
**mongolian spot**	67	0.05	10.45
**giant melanocytic nevus**	62	0.05	16.13
**black heel**	61	0.05	6.56
**naevus blue**	57	0.05	14.04
**melanoma mimic**	52	0.04	0.00
**combined nevus**	51	0.04	0.00
**melanonychia striata**	51	0.04	5.88
**blue nevus ota**	48	0.04	14.58
**mucosal lentigines**	47	0.04	0.00
**naevus halo**	45	0.04	17.78
**ephelides**	37	0.03	2.70
**hereditary dysplastic nevus syndrome**	37	0.03	18.92
**spindle cell nevus**	36	0.03	11.11
**melanotic macule**	33	0.03	3.03
**naevus achromic**	33	0.03	9.09
**spitz nevus**	32	0.03	3.12
**lentigo senilis**	27	0.02	0.00
**nevus pigmentosus et pilosus**	27	0.02	3.70
**lentigo simplex**	26	0.02	0.00
**nevus repigmented**	22	0.02	9.09
**nail nevus**	18	0.01	11.11
**ink spot nevus**	16	0.01	0.00

	**naevus of ota bilateral type**	15	0.01	0.00
	**naevus giant pigmented**	11	0.01	0.00
	**labial melanotic macule**	4	0.00	25.00
**Pigmented lesions malignant**	**malignant melanoma**	1769	1.40	5.99
	**superficial spreading melanoma ssm**	285	0.23	4.91
	**lentigo maligna**	199	0.16	3.02
	**lentigo maligna melanoma**	117	0.09	1.71
	**nail melanoma**	60	0.05	6.67
	**nodular melanoma nm**	59	0.05	1.69
	**metastatic melanoma**	45	0.04	13.33
	**acrolentiginous melanoma alm**	34	0.03	2.94
	**amelanotic malignant melanoma amm**	26	0.02	0.00
	**malignant melanoma metastatic**	22	0.02	4.55
	**mucosal melanoma**	12	0.01	8.33
	**melanoma of the parotid**	11	0.01	18.18
	**Unknown**	2454	1.94	6.40
	**All**	126477	100.00	10.38

Supplementary Table 3: Frequency Table and False Positive Rates of Diagnoses in the Esteva Dataset

Frequency table of diagnoses and False Positive Rates of evaluated skin lesion images in the Esteva dataset.
All, Number of all evaluated skin lesion images in the Esteva dataset; FPR, False positive rate (%) of the diagnosis in the Esteva dataset; n, Number of occurrences of diagnosis; %, Percentage of diagnosis in relation to all evaluated skin lesion images in the Esteva dataset.

Category	Diagnosis	n	%	FPR
Benign dermal tumors cysts sinuses	**pilar cyst**	601	0.79	14.14
	**skin tag**	600	0.79	19.17
	**hemangioma**	480	0.63	15.83
	**fibroma**	453	0.60	18.76
	**pyogenic granuloma**	291	0.38	14.43
	**dermatofibroma**	283	0.37	20.85
	**telangiectases**	254	0.34	9.45
	**sebaceous hyperplasia**	243	0.32	10.29
	**syringoma**	202	0.27	8.91
	**fordyce spots**	181	0.24	12.15
	**adenoma sebaceum**	120	0.16	13.33
	**mucous cyst**	117	0.15	17.95
	**angiokeratomas**	113	0.15	9.73
	**becker nevus**	112	0.15	15.18
	**trichoepithelioma**	86	0.11	11.63
	**angioma**	83	0.11	12.05
	**myxoid cyst**	78	0.10	2.56
	**pilomatricoma**	78	0.10	10.26
	**haemangioma**	76	0.10	19.74
	**venous lake**	74	0.10	9.46
	**leiomyoma**	70	0.09	5.71
	**hidrocystoma**	67	0.09	8.96
	**lymphangioma**	62	0.08	20.97
	**nevus lipomatosis**	61	0.08	27.87
	**scrotal cyst**	60	0.08	28.33

**apocrine hydrocystoma**	59	0.08	5.08
**steatocystoma multiplex**	54	0.07	18.52
**port wine stain**	52	0.07	13.46
**eccrine poroma**	50	0.07	10.00
**dilated pore of winer**	48	0.06	14.58
**trichofolliculoma**	48	0.06	20.83
**cherry angioma**	47	0.06	12.77
**spider angioma**	47	0.06	17.02
**spider nevus**	42	0.06	11.90
**cutis marmorata telangiectatica congenita**	41	0.05	7.32
**klippel trenaunay syndrome**	41	0.05	7.32
**chondroma**	40	0.05	5.00
**granulation tissue**	40	0.05	2.50
**ganglion**	39	0.05	20.51
**glomus tumor**	37	0.05	8.11
**venous malformations**	36	0.05	8.33
**dermoid cyst**	33	0.04	9.09
**eruptive vellus hair cysts**	33	0.04	15.15
**infantile digital fibromatosis**	29	0.04	10.34
**pilonidal sinus**	29	0.04	24.14
**strawberry hemangioma**	29	0.04	3.45
**acrotrichoma**	27	0.04	25.93
**spiradenoma**	26	0.03	7.69
**cylindroma**	25	0.03	4.00
**collagen nevus**	23	0.03	26.09
**nevus anemicus**	23	0.03	4.35
**dental sinus**	22	0.03	18.18
**ectopic respiratory epithelia**	22	0.03	0.00
**connective tissue nevus**	20	0.03	5.00
**neurolemmoma**	20	0.03	10.00
**myxoma**	19	0.03	5.26
**endometriosis**	18	0.02	33.33

	epithelioma adenoides cysticum	18	0.02	11.11
	fibrolipoma	17	0.02	0.00
	lymphatic malformations	16	0.02	6.25
	nevus flammeus	16	0.02	25.00
	aponeurotic fibroma	15	0.02	6.67
	cirsoid aneurysm	14	0.02	7.14
	vascular anomaly	14	0.02	28.57
	myofibromatosis	13	0.02	23.08
	arteriovenous malformation	12	0.02	16.67
	periungual fibroma	12	0.02	25.00
	tenosynovitis nodular	12	0.02	0.00
	cyst synovial	11	0.01	9.09
	granuloma pyogenic	11	0.01	0.00
	hemangioma infancy	10	0.01	10.00
	hemangioma racemosum	10	0.01	0.00
	trichoblastoma	10	0.01	10.00
	angioma serpiginosum	9	0.01	22.22
	perifolicular fibroma	8	0.01	0.00
	trichoadenoma	8	0.01	12.50
	trichoepithelioma papulosum multiplex	8	0.01	0.00
Cutaneous lymphoma and lymphoid infiltrates	cutaneous t cell lymphoma	360	0.48	11.67
	lymphoma b cell	112	0.15	6.25
	lymphomatoid papulosis	110	0.15	9.09
	pseudo lymphoma other types	15	0.02	0.00
	erythema arciforme et palpabile migrans	4	0.01	0.00
	lymphomas lymphocytic infiltrations	2	0.00	0.00
Epidermal tumors hamartomas milia and growths benign	seborrheic keratosis	876	1.16	4.34
	benign keratosis	414	0.55	7.00
	epidermal nevus	388	0.51	11.08
	seborrheic keratoses ruff	331	0.44	0.60
	milia	276	0.36	18.48
	porokeratosis	176	0.23	5.68
	prurigo nodularis	86	0.11	34.88

	**dermatosis papulosa nigra**	75	0.10	10.67
	**callus**	72	0.10	5.56
	**acquired digital fibrokeratoma**	50	0.07	12.00
	**corns**	40	0.05	10.00
	**seborrheic keratosis irritated**	40	0.05	0.00
	**seborrheic keratoses smooth**	34	0.04	0.00
	**giant comedo**	30	0.04	6.67
	**acral keratosis**	27	0.04	11.11
	**keratosis seborrheic**	26	0.03	7.69
	**stucco keratosis**	23	0.03	8.70
	**stucco keratoses**	19	0.03	5.26
	**leser trelat sign**	17	0.02	0.00
	**clear cell acanthoma**	16	0.02	6.25
	**warty dyskeratoma**	11	0.01	0.00
**Epidermal tumors pre malignant and malignant**	**solar damage actinic keratosis**	951	1.26	4.21
	**basal cell carcinoma**	900	1.19	8.22
	**squamous cell carcinoma**	657	0.87	14.31
	**bowens disease**	416	0.55	7.21
	**keratoacanthoma**	413	0.55	13.56
	**cutaneous horn**	160	0.21	8.75
	**actinic keratosis**	108	0.14	8.33
	**pagets disease**	102	0.13	11.76
	**leukoplakia**	101	0.13	6.93
	**solar damage**	62	0.08	3.23
	**actinic cheilitis**	60	0.08	1.67
	**arsenical keratoses**	54	0.07	11.11
	**basal cell nevus syndrome**	48	0.06	12.50
	**erythroplasia queyrat**	48	0.06	18.75
	**extramammary paget disease**	46	0.06	10.87
	**pigmented basal cell carcinoma**	39	0.05	7.69
	**solid cystic basal cell carcinoma**	38	0.05	21.05
	**solar damage actinex treatment**	36	0.05	2.78
	**nevoid basal cell carcinoma syndrome**	35	0.05	11.43

**superficial basal cell carcinoma**	26	0.03	0.00
**solar damage actinic cheilitis**	20	0.03	5.00
**basal cell carcinoma ulcerating**	18	0.02	0.00
**actinic keratosis face**	16	0.02	6.25
**hypertrophic actinic keratosis**	16	0.02	0.00
**verrucous carcinoma**	16	0.02	6.25
**actinic keratosis lesion**	14	0.02	14.29
**leucoplakia praecancerosa**	14	0.02	0.00
**ulcus terebrans**	14	0.02	7.14
**basal cell carcinoma ear**	13	0.02	0.00
**carcinoma of lip**	13	0.02	15.38
**actinic keratosis ear**	12	0.02	0.00
**basal cell carcinoma lid**	11	0.01	18.18
**basal cell carcinoma scalp**	11	0.01	18.18
**actinic keratosis hand**	10	0.01	0.00
**basal cell carcinoma nose**	10	0.01	0.00
**solar damage fu treatment**	10	0.01	0.00
**squamous cell carcinoma nose**	9	0.01	0.00
**basal cell carcinoma morpheiform**	7	0.01	0.00
**basal cell carcinoma face**	6	0.01	16.67
**basal cell carcinoma lesion**	6	0.01	16.67
**squamous cell carcinoma ear**	6	0.01	0.00
**squamous cell carcinoma lesion**	5	0.01	20.00
**keratosis solar**	4	0.01	0.00
**squamous cell carcinoma lip**	4	0.01	25.00
**actinic keratosis nose**	3	0.00	0.00
**basal cell carcinoma aldara**	3	0.00	66.67
**squamous cell carcinoma face**	3	0.00	0.00
**actinic keratosis pigmented**	2	0.00	0.00
**basal cell carcinoma superficial**	2	0.00	0.00
**actinic keratosis horn**	1	0.00	100.00
**basal cell carcinoma lip**	1	0.00	0.00

Genodermatoses and supernumerary growths	ichthyosis	505	0.67	26.14
	epidermolysis bullosa	435	0.57	20.92
	neurofibromatosis	342	0.45	16.67
	dariers disease	247	0.33	8.50
	ichthyosis vulgaris	122	0.16	8.20
	tuberous sclerosis	122	0.16	27.87
	rendu osler syndrome	113	0.15	15.93
	incontinentia pigmenti	85	0.11	29.41
	epidermolytic hyperkeratosis	74	0.10	5.41
	hailey hailey disease	73	0.10	12.33
	pseudoxanthoma elasticum	71	0.09	11.27
	erythrokeratodermia progressive symmetric	63	0.08	14.29
	collodion baby	61	0.08	26.23
	ehlers danlos syndrome	58	0.08	27.59
	supernumerary growths	58	0.08	29.31
	vascular tumors and malformations	57	0.08	10.53
	ichthyosis congenita	43	0.06	2.33
	x linked ichthyosis	41	0.05	19.51
	leopard syndrome	39	0.05	17.95
	acrokeratoelastoidosis	36	0.05	5.56
	erythrokeratodermia variabilis	34	0.04	26.47
	muir torre syndrome	29	0.04	10.34
	peutz jeghers syndrome	29	0.04	10.34
	lipoid proteinosis urbach wiethe disease	27	0.04	14.81
	congenital ichthyosiform erythroderma	26	0.03	3.85
	rothmund thomson syndrome	26	0.03	11.54
	xeroderma pigmentosum	25	0.03	32.00
	albinism	24	0.03	12.50
	buschke ollendorf syndrome	22	0.03	13.64
	acrokeratosis verruciformis	21	0.03	0.00
	congenital erythropoietic porphyria	21	0.03	14.29
	congenital dyskeratosis	20	0.03	20.00

	**acrodermatitis enteropathica**	19	0.03	52.63
	**ichthyosis sex linked**	16	0.02	0.00
	**sjgren larsson syndrome**	16	0.02	6.25
	**cowden disease**	15	0.02	13.33
	**congenital anomalies**	13	0.02	0.00
	**lamellar icthyosis**	13	0.02	30.77
	**birt hogg dube**	11	0.01	9.09
	**dyschromatosis universalis**	11	0.01	45.45
	**ichthyosis hystrix**	11	0.01	9.09
	**laugier hunziker s syndrome**	11	0.01	9.09
	**gargoylism**	9	0.01	33.33
	**harlequim ichthyosis**	6	0.01	16.67
	**leprechaunism syndrome**	6	0.01	0.00
	**erythrodermia exfoliativa leiner**	4	0.01	0.00
	**phakomatosis pigmentovascularis**	4	0.01	0.00
	**ichthyosis dominant**	3	0.00	0.00
	**ichthyosis other forms**	1	0.00	0.00
	**knuckle pads**	1	0.00	0.00
**Inflammatory**	**acne vulgaris**	1671	2.21	19.21
	**leprosy**	921	1.22	19.87
	**eczema**	836	1.10	19.26
	**rosacea**	821	1.08	8.65
	**herpes zoster**	799	1.06	11.89
	**syphilis**	743	0.98	17.63
	**lichen**	734	0.97	12.53
	**alopecia**	685	0.90	23.65
	**psoriasis**	683	0.90	9.37
	**sunburn**	678	0.90	28.47
	**tinea corporis**	676	0.89	9.02
	**onychomycosis**	652	0.86	2.45
	**hair disorderstrichorrhexis nodosa etc**	640	0.85	10.47
	**impetigo**	590	0.78	15.76
	**fungal infections dermal and subcut**	585	0.77	15.21

**pigmentation diseases**	578	0.76	19.20
**vasculitis**	572	0.76	11.01
**scarring alopecia**	558	0.74	9.86
**lichen planus**	551	0.73	6.72
**seborrheic dermatitis**	536	0.71	10.26
**atrophic skin disease striae and elastic fiber disorders**	535	0.71	19.07
**tick bite**	528	0.70	28.60
**candidiasis**	522	0.69	7.66
**tinea pedis**	486	0.64	4.53
**scabies**	472	0.62	13.77
**urticaria**	466	0.62	12.66
**non scarring alopecia**	460	0.61	11.52
**stasis ulcer**	447	0.59	2.91
**scarring**	439	0.58	11.16
**depositional diseases calcinosis cutis amyloid mucinosis gout and others**	422	0.56	12.80
**molluscum contagiosum**	414	0.55	27.54
**lupus acute**	410	0.54	22.20
**drug eruption**	395	0.52	11.39
**allergic contact dermatitis**	394	0.52	9.64
**pustular skin disease non infectious**	393	0.52	6.36
**folliculitis**	382	0.50	23.04
**factitial dermatitis**	377	0.50	13.00
**metabolic and nutritional diseases**	363	0.48	23.69
**atopic dermatitis feet**	339	0.45	5.01
**erythema multiforme and fixed drug eruptions**	336	0.44	16.96
**xanthomas**	320	0.42	16.25
**photodermatoses**	313	0.41	11.18
**scleroderma**	300	0.40	19.33
**herpes simplex**	293	0.39	13.65
**bullous pemphigoid**	289	0.38	14.53
**perioral dermatitis**	280	0.37	4.29

**condyloma acuminata**	265	0.35	16.98
**sarcoidosis**	264	0.35	11.74
**tinea versicolor**	263	0.35	10.65
**keratosis pilaris and related follicular disorders**	261	0.34	6.90
**rhinophyma**	261	0.34	10.34
**dermatomyositis**	253	0.33	8.70
**histiocytic disorders and proliferations**	253	0.33	18.18
**dry skin eczema**	249	0.33	4.82
**shoe allergy**	247	0.33	25.91
**solar elastosis**	247	0.33	10.53
**erysipelas**	246	0.32	9.35
**tinea capitis**	242	0.32	6.20
**granuloma annulare**	238	0.31	7.14
**gyrate erythemas**	234	0.31	11.11
**mastocytosis**	234	0.31	7.69
**panniculitis lipodystrophy and diseases of subcutis**	234	0.31	12.82
**dermatitis herpetiformis**	231	0.31	15.15
**pemphigus**	231	0.31	12.55
**pityriasis rosea**	229	0.30	9.17
**dyshidrotic eczema**	222	0.29	3.15
**stasis dermatitis**	221	0.29	2.26
**pityriasis versicolor**	218	0.29	10.55
**cutaneous manifestations of systemic disease**	214	0.28	18.22
**pemphigus vulgaris**	213	0.28	5.63
**tinea cruris**	212	0.28	25.47
**atopic dermatitis adult phase**	209	0.28	1.91
**varicella**	209	0.28	40.19
**lichen simplex**	205	0.27	8.29
**lichen sclerosus skin**	195	0.26	14.36
**erythroderma**	194	0.26	7.73
**malformations of extremities**	190	0.25	10.53

**tuberculosis**	190	0.25	10.53
**solar damage atrophy**	187	0.25	17.11
**pityrosporum folliculitis**	185	0.24	12.97
**misuse dermatitis**	184	0.24	7.61
**reiters disease**	182	0.24	7.14
**psoriasis chronic plaque**	178	0.24	3.37
**eczema hand**	176	0.23	1.70
**pediculosis lids**	172	0.23	16.86
**solar purpura**	172	0.23	5.23
**hand foot mouth**	164	0.22	17.07
**cheilitis**	161	0.21	8.70
**lupus subacute**	161	0.21	13.66
**eczema herpeticum**	153	0.20	8.50
**toxic epidermal necrolysis and stevens johnson**	150	0.20	6.67
**pernio and mimics**	148	0.20	10.14
**diseases of the ear**	144	0.19	11.11
**insect bite**	143	0.19	16.78
**morphea**	143	0.19	13.99
**nematode infection**	143	0.19	13.99
**warts vulgaris**	141	0.19	9.93
**planar warts**	138	0.18	7.97
**stasis edema**	138	0.18	5.07
**pigmented purpuric dermatosis**	135	0.18	6.67
**virus exanthem**	134	0.18	6.72
**onycholysis**	133	0.18	3.76
**pitted keratolysis**	133	0.18	6.02
**leukocytoclastic vasculitis**	131	0.17	8.40
**acanthosis nigricans and carp**	130	0.17	13.08
**perforating disorders**	130	0.17	9.23
**tinea manus**	130	0.17	1.54
**diaper dermatitis**	129	0.17	13.95
**furuncle**	129	0.17	22.48

**hidradenitis**	129	0.17	36.43
**tattoo and foreign body reactions**	129	0.17	10.85
**cellulitis**	127	0.17	9.45
**ashy dermatosis**	126	0.17	15.08
**nail psoriasis**	124	0.16	3.23
**sun damaged skin**	123	0.16	20.33
**atopic dermatitis**	120	0.16	15.00
**gianotti crosti**	120	0.16	14.17
**verruca vulgaris**	119	0.16	13.45
**eczema nummular**	116	0.15	4.31
**necrobiosis lipoidica**	115	0.15	8.70
**acne keloidalis**	113	0.15	15.93
**balanitis**	113	0.15	7.96
**livedo reticularis**	112	0.15	6.25
**biting fleas**	111	0.15	8.11
**solar damage favre racouchot**	107	0.14	8.41
**nail diseases**	104	0.14	6.73
**clubbing**	103	0.14	3.88
**genital warts**	103	0.14	26.21
**id reaction**	103	0.14	6.80
**porphyria**	103	0.14	10.68
**scarlet fever**	103	0.14	3.88
**tinea faciale**	103	0.14	13.59
**tinea nigra**	102	0.13	4.90
**pemphigus foliaceous**	101	0.13	6.93
**herpes simplex analis**	100	0.13	6.00
**inverse psoriasis**	99	0.13	13.13
**tinea faciei**	98	0.13	12.24
**contact dermatitis**	97	0.13	10.31
**lupus vulgaris**	97	0.13	8.25
**pityriasis lichenoides and pleva**	97	0.13	14.43
**chronic recurrent aphthae**	96	0.13	13.54
**hypereosinophilic syndrome**	95	0.13	4.21

**intertrigo**	95	0.13	4.21
**beaus lines**	94	0.12	7.45
**staphylococcal scalded skin syndrome**	93	0.12	16.13
**radiodermatitis**	92	0.12	6.52
**subacute cutaneous lupus erythematosus**	92	0.12	14.13
**ingrown nail**	91	0.12	6.59
**discoid lupus erythematosus**	90	0.12	28.89
**neurodermatitis**	90	0.12	12.22
**henoch schonlein purpura**	89	0.12	6.74
**thermal burn**	89	0.12	5.62
**herpes gestationis**	88	0.12	13.64
**paronychia**	87	0.11	4.60
**pityriasis rubra pilaris**	87	0.11	9.20
**diabetic ulcer**	86	0.11	10.47
**geographic tongue**	86	0.11	4.65
**lymphedema**	85	0.11	11.76
**neurotrophic ulcer**	85	0.11	11.76
**tinea ringworm versicolor**	85	0.11	4.71
**warts**	85	0.11	10.59
**papulose bowenoide**	84	0.11	20.24
**pemphigus foliaceus**	84	0.11	1.19
**tinea ringworm body**	82	0.11	4.88
**erythema ab igne**	81	0.11	11.11
**median nail dystrophy**	81	0.11	6.17
**raynauds disease**	81	0.11	7.41
**steroid acne**	81	0.11	16.05
**nummular eczema**	80	0.11	5.00
**chronic bullous disease of childhood**	78	0.10	21.79
**epidermodysplasia verruciformis**	77	0.10	10.39
**miliaria**	76	0.10	6.58
**angioedema**	75	0.10	13.33
**diseases of the eye**	74	0.10	2.70

**nail dystrophy**	72	0.10	8.33
**pincer nails**	72	0.10	13.89
**herpetic sycosis**	71	0.09	21.13
**poikiloderma civatte**	70	0.09	1.43
**purpura**	70	0.09	8.57
**measles**	69	0.09	5.80
**psoriasis palms soles**	69	0.09	1.45
**hairy tongue**	67	0.09	13.43
**scalp psoriasis**	66	0.09	3.03
**tinea facei**	65	0.09	7.69
**grovers disease**	64	0.08	3.12
**leukonychia**	64	0.08	4.69
**pemphigus vegetans**	64	0.08	6.25
**acne conglobata**	63	0.08	11.11
**acne excoriee**	63	0.08	12.70
**desquamation**	63	0.08	3.17
**erythrasma**	63	0.08	15.87
**guttate psoriasis**	62	0.08	12.90
**chancroid**	61	0.08	19.67
**fibromatosis**	61	0.08	13.11
**pigmented bands**	61	0.08	11.48
**lichen striatus**	60	0.08	10.00
**solar damage telangiectasia**	60	0.08	1.67
**gonorrhea**	59	0.08	20.34
**lichen nitidus**	58	0.08	8.62
**splinter hemorrhage**	58	0.08	10.34
**ulcer**	57	0.08	3.51
**candidiasis chronic mucocutaneos**	56	0.07	8.93
**spider bite**	54	0.07	35.19
**behcets disease**	53	0.07	18.87
**sjogrens syndrome**	53	0.07	18.87
**terry nails**	53	0.07	5.66
**green nail**	52	0.07	3.85

**piedra white**	52	0.07	13.46
**rheumatoid nodule**	52	0.07	19.23
**stasis dermatitis and ulcers**	52	0.07	0.00
**crest syndrome**	51	0.07	9.80
**dermographia**	51	0.07	7.84
**orf**	51	0.07	43.14
**abrasion**	50	0.07	14.00
**hyperhidrosis**	50	0.07	4.00
**tungiasis**	50	0.07	36.00
**acne cystic**	49	0.06	14.29
**acquired autoimmune bullous diseaseherpes gestationis**	49	0.06	2.04
**dermatoliposclerosis**	49	0.06	4.08
**diseases of bone**	49	0.06	6.12
**irritant contact dermatitis**	49	0.06	2.04
**neurotic excoriations**	48	0.06	18.75
**pachyonychia congenita**	47	0.06	10.64
**panarteritis nodosa kussmaul maier**	47	0.06	10.64
**wet to dry foot syndrome**	47	0.06	8.51
**psoriasis treatment**	46	0.06	10.87
**warts filiformis**	46	0.06	13.04
**angiolymphoid hyperplasia with eosinophilia**	45	0.06	17.78
**atrophy blanche**	45	0.06	6.67
**ecthyma simplex**	45	0.06	26.67
**relapsing polychondritis**	45	0.06	6.67
**skin diseases of pregnancy**	45	0.06	6.67
**koilonychia**	44	0.06	0.00
**nail pigmentation**	44	0.06	9.09
**acne rosacea**	43	0.06	6.98
**cimicosis**	43	0.06	11.63
**tylotic dermatitis**	43	0.06	2.33
**cicatricial pemphigoid**	42	0.06	2.38
**epidermolysis bullosa acquisita**	42	0.06	9.52

**onychogryphosis**	42	0.06	7.14
**psoriasis nails**	42	0.06	11.90
**acne papulopustulosa**	41	0.05	9.76
**trichomycosis**	41	0.05	4.88
**erythema craquele**	40	0.05	2.50
**erythema infectiosum**	40	0.05	20.00
**folliculitis cheloidalis**	40	0.05	20.00
**fox fordyce disease**	40	0.05	7.50
**hidroa vacciniforme**	40	0.05	12.50
**rocky mountain spotted fever**	40	0.05	10.00
**meningococcemia**	39	0.05	15.38
**urticarial vasculitis**	39	0.05	5.13
**acne inversa**	38	0.05	13.16
**fissure**	38	0.05	0.00
**habit tic deformity**	38	0.05	18.42
**infantile atopic dermatitis**	38	0.05	7.89
**schistosomiasis cutaneous**	38	0.05	7.89
**stomatitis**	38	0.05	13.16
**acne fulminans**	36	0.05	5.56
**bacterial infections**	36	0.05	2.78
**trichostasis spinulosa**	36	0.05	2.78
**cutis marmorata**	35	0.05	5.71
**granuloma**	35	0.05	2.86
**maceration**	35	0.05	8.57
**nail beading**	35	0.05	5.71
**nail polish dermatitis**	35	0.05	0.00
**peripheral occlusive arterial disease**	35	0.05	0.00
**pseudofolliculitis barbae**	35	0.05	14.29
**thrombocytopenic purpura idiopatic**	35	0.05	28.57
**tinea ringworm face**	35	0.05	5.71
**atopic dermatitis infant phase**	34	0.04	2.94
**herpes simplex virus**	34	0.04	11.76
**tinea incognito**	34	0.04	17.65

**acne scar**	33	0.04	6.06
**half and half nail**	33	0.04	6.06
**patch testing**	33	0.04	15.15
**twenty nail dystrophy**	33	0.04	6.06
**atopic eczema**	32	0.04	3.12
**gangrene**	32	0.04	12.50
**pomade acne**	32	0.04	6.25
**herpetic gingivostomatite**	31	0.04	12.90
**painful bruising syndrome**	31	0.04	0.00
**psoriatic erythroderma**	31	0.04	9.68
**scrotal tongue**	31	0.04	3.23
**cat scratch disease**	30	0.04	33.33
**eczematid**	30	0.04	3.33
**lichen planus actinicus**	30	0.04	50.00
**lichen planus linearis**	30	0.04	10.00
**purpura fulminans**	30	0.04	16.67
**scrophuloderma**	30	0.04	20.00
**dermatitis ekzema**	29	0.04	6.90
**eczema face**	29	0.04	3.45
**gingival disease**	29	0.04	10.34
**herpes cutaneous**	29	0.04	17.24
**ischemia**	29	0.04	6.90
**tinea barbae**	29	0.04	6.90
**warts periungual**	29	0.04	6.90
**wells syndrome**	29	0.04	27.59
**chronic venous insufficiency grade ii**	28	0.04	0.00
**degos disease**	28	0.04	25.00
**nail cosmesis**	28	0.04	7.14
**parapsoriasis en plaques**	28	0.04	3.57
**wart**	28	0.04	7.14
**wound infection**	28	0.04	10.71
**acne closed comedo**	27	0.04	11.11
**acne neonatorum**	27	0.04	11.11

**infected eczema**	27	0.04	7.41
**pemphigoid bullous pemphigoid**	27	0.04	11.11
**rhus dermatitis**	27	0.04	3.70
**varicosis**	27	0.04	11.11
**caterpillar dermatitis**	26	0.03	15.38
**cercaria dermatitis**	26	0.03	15.38
**keratolysis exfoliativa**	26	0.03	0.00
**myxoedema pretibial**	26	0.03	11.54
**rosacea nose**	26	0.03	15.38
**torus palantine**	26	0.03	7.69
**connective tissue disorders**	25	0.03	52.00
**myiasis**	25	0.03	24.00
**nail ridging**	25	0.03	0.00
**nail trauma**	25	0.03	4.00
**onychoschizia**	25	0.03	0.00
**solar damage cutis rhomboidalis nuchae**	25	0.03	12.00
**warts flat**	25	0.03	8.00
**acroangiodermatitis**	24	0.03	20.83
**cholinergic urticaria**	24	0.03	4.17
**kawasaki syndrome**	24	0.03	8.33
**racquet nail**	24	0.03	4.17
**verrucae planae juveniles**	24	0.03	16.67
**acute eczema**	23	0.03	0.00
**eczema fingertips**	23	0.03	0.00
**erysipeloid**	23	0.03	4.35
**granuloma multiforme**	23	0.03	8.70
**psoriasis scalp**	23	0.03	4.35
**urticaria vasculitis**	23	0.03	4.35
**acne pustular**	22	0.03	22.73
**atypical mycobacterium**	22	0.03	18.18
**reticular erythematous mucinosis**	22	0.03	0.00
**fourniers gangrene**	21	0.03	0.00
**lichen planus penis**	21	0.03	4.76

**pincer nail syndrome**	21	0.03	0.00
**thrombangiitis obliterans**	21	0.03	0.00
**acanthoma fissuratum**	20	0.03	0.00
**cryoglobulinemia**	20	0.03	0.00
**dry nails**	20	0.03	15.00
**lupus chronic cutaneous**	20	0.03	10.00
**mucous membrane psoriasis**	20	0.03	15.00
**small pox**	20	0.03	25.00
**decubitus ulcer**	19	0.03	15.79
**eczema arms**	19	0.03	5.26
**lichen planus oral**	19	0.03	26.32
**morphea linear**	19	0.03	5.26
**nodulo cystic acne vulgaris**	19	0.03	5.26
**palmar wart**	19	0.03	15.79
**roseola infantum**	19	0.03	21.05
**scleromyxedema**	19	0.03	0.00
**warts oral**	19	0.03	5.26
**lichen planopilaris**	18	0.02	5.56
**lichen planus of the mucosa erosive**	18	0.02	16.67
**pemphigus mucosae**	18	0.02	0.00
**psoriasis inversa**	18	0.02	11.11
**streptococci anal penis**	18	0.02	11.11
**tinea manuum**	18	0.02	0.00
**enterovirus**	17	0.02	11.76
**glanzmanns thrombasthenia**	17	0.02	0.00
**nail shedding**	17	0.02	5.88
**pseudomonas**	17	0.02	5.88
**warts cryotherapy**	17	0.02	5.88
**dermatosis neglecta**	16	0.02	0.00
**diabetic gangrene**	16	0.02	18.75
**eosinophilic pustular folliculitis**	16	0.02	6.25
**morphoea generalized**	16	0.02	12.50
**parasitoses bites**	16	0.02	31.25

**acne cystica**	15	0.02	13.33
**eosinophilic ulcer of the tongue**	15	0.02	6.67
**gram negative infection of the foot**	15	0.02	6.67
**herpes buttocks**	15	0.02	13.33
**lichen planus vesicular**	15	0.02	40.00
**warts plantaris**	15	0.02	0.00
**culicosis bullosa**	14	0.02	7.14
**eczema leg**	14	0.02	7.14
**erythema elevatum diutinum**	14	0.02	14.29
**gloves and socks syndrome**	14	0.02	0.00
**mononucleosis ampicillin syndrome**	14	0.02	0.00
**pyoderma faciale**	14	0.02	28.57
**rosacea steroid**	14	0.02	0.00
**warts mouth**	14	0.02	7.14
**warts plane**	14	0.02	0.00
**benign mucosal pemphigoid**	13	0.02	0.00
**erysipelas bullosum**	13	0.02	0.00
**heels dry cracked**	13	0.02	30.77
**notalgia paraesthetica**	13	0.02	0.00
**progressive systemic scleroderma**	13	0.02	7.69
**psoriasis guttate**	13	0.02	7.69
**subacute cutaneous lupus erythematosus scle**	13	0.02	0.00
**uvl burn**	13	0.02	0.00
**wound dehiscence**	13	0.02	7.69
**acne necrotica**	12	0.02	25.00
**allergic hand eczema**	12	0.02	0.00
**alopecia acquisitum centrifugum**	12	0.02	33.33
**eczema anal**	12	0.02	0.00
**elephantiasis nostras verrucosa**	12	0.02	16.67
**hecks disease**	12	0.02	0.00
**herpes simplex genitalis**	12	0.02	41.67
**pterygium inversum unguis**	12	0.02	0.00

**syringolymphoid hyperplasia with alopecia**	12	0.02	16.67
**atopic eczema of the hands**	11	0.01	0.00
**eczema scrotum**	11	0.01	9.09
**herpes simplex labialis**	11	0.01	0.00
**leukoedema oral**	11	0.01	27.27
**lichen planus hypertrophic**	11	0.01	72.73
**metal dermatitis**	11	0.01	18.18
**polychondritis**	11	0.01	0.00
**psoriasis hand**	11	0.01	27.27
**tinea ringworm hand dorsum**	11	0.01	0.00
**balanitis bacterial**	10	0.01	0.00
**candidiasis mouth**	10	0.01	10.00
**diabetic bullae**	10	0.01	10.00
**eczema ears**	10	0.01	20.00
**lymphangitis acute lymphangitis**	10	0.01	40.00
**pseudomonas folliculitis**	10	0.01	10.00
**tinea ringworm scalp**	10	0.01	20.00
**erosio interdigitalis blastomycetica**	9	0.01	0.00
**erythromelalgia**	9	0.01	11.11
**localized pemphigoid**	9	0.01	0.00
**morphoea nodular**	9	0.01	0.00
**morsicatio buccarum**	9	0.01	0.00
**pemphigus foliaceus localized form**	9	0.01	11.11
**portuguese man of war dermatitis**	9	0.01	0.00
**acne infantile**	8	0.01	0.00
**atopic dermatitis childhood phase**	8	0.01	0.00
**eczema microbic**	8	0.01	0.00
**morphoea localized**	8	0.01	0.00
**onychogryphosis and onychauxis**	8	0.01	0.00
**pemphigus vegitant**	8	0.01	50.00
**psoriasis vulgaris chronic stationary type**	8	0.01	0.00
**shaving folliculitis**	8	0.01	12.50

**acne comedonica**	7	0.01	0.00
**axillary granular parakeratosis**	7	0.01	0.00
**distal splitting hang nail**	7	0.01	0.00
**drug lichenoid eruption**	7	0.01	0.00
**eczema foot**	7	0.01	0.00
**eczema impetiginized**	7	0.01	0.00
**eczema subacute**	7	0.01	14.29
**lichen planus of the mucosa**	7	0.01	0.00
**linear scleroderma**	7	0.01	14.29
**lingua plicata**	7	0.01	0.00
**onychodystrophia mediana canaliformis**	7	0.01	0.00
**psoriasis arthropathica**	7	0.01	0.00
**psoriasis hiv**	7	0.01	0.00
**psoriasis infants**	7	0.01	14.29
**warts plantar**	7	0.01	14.29
**acne open comedo**	6	0.01	0.00
**ant bite**	6	0.01	16.67
**blue nails minocycline**	6	0.01	16.67
**chronic cutaneous graft versus host reaction**	6	0.01	16.67
**dermatitis swimming**	6	0.01	0.00
**disseminated lokalized scleroderma**	6	0.01	0.00
**herpes type recurrent**	6	0.01	0.00
**lichen planus annular**	6	0.01	50.00
**lipoatrophy postherpertic lipoatrophy**	6	0.01	0.00
**localized scleroderma**	6	0.01	16.67
**lupus erythematosus tumidus**	6	0.01	0.00
**phlebitis superficial**	6	0.01	0.00
**primary tufted hairs**	6	0.01	16.67
**psoriasis anus**	6	0.01	16.67
**staphylococcal folliculitis**	6	0.01	16.67
**warts immunocompromised**	6	0.01	0.00
**contact dermatitis leg ulcers**	5	0.01	60.00

**eczema areola**	5	0.01	0.00
**herpes immunocompromised**	5	0.01	20.00
**herpes simplex oralis**	5	0.01	0.00
**hyperkeratotic fissured hand and foot eczema**	5	0.01	20.00
**lichen planus exanthematicus**	5	0.01	20.00
**lupus miliaris disseminatus faciei**	5	0.01	0.00
**malassezia folliculitis**	5	0.01	20.00
**pachyonychia congenita hereditaria**	5	0.01	20.00
**primary herpes simplex virus**	5	0.01	20.00
**psoriasis penis**	5	0.01	0.00
**psoriasis psoriasis after erysipelas**	5	0.01	20.00
**superficial fungal infections**	5	0.01	0.00
**sycosis barbae**	5	0.01	0.00
**tinea ringworm foot dorsum**	5	0.01	0.00
**ulcus cruris mixtum**	5	0.01	20.00
**urticaria acute**	5	0.01	0.00
**cosmetic fragrance allergy**	4	0.01	25.00
**folliculitis of barbae**	4	0.01	0.00
**herpes type primary**	4	0.01	0.00
**lichen planus follicularis**	4	0.01	0.00
**lichen planus palm soles**	4	0.01	0.00
**lichen planus verrucosus**	4	0.01	0.00
**lupus erythematosus subacute cutaneous lupus erythematosus**	4	0.01	25.00
**tinea barbae profunda**	4	0.01	25.00
**tinea capitis profunda**	4	0.01	0.00
**tinea ringworm border**	4	0.01	0.00
**zoster gangraenosus**	4	0.01	0.00
**eczema lids**	3	0.00	0.00
**eczema trunk generalized**	3	0.00	0.00
**eczema vulva**	3	0.00	0.00
**lichen planus annularis**	3	0.00	0.00
**lichen planus hypertrophicus**	3	0.00	0.00

	**lupus erythematosus systemic**	3	0.00	33.33
	**psoriasis light sensative**	3	0.00	33.33
	**psoriasis vulgaris nail changes**	3	0.00	0.00
	**ridging beading**	3	0.00	0.00
	**tinea capitis superficialis**	3	0.00	0.00
	**tinea ringworm beard**	3	0.00	0.00
	**tinea ringworm groin**	3	0.00	0.00
	**tinea ringworm palm**	3	0.00	33.33
	**urticaria factitia**	3	0.00	0.00
	**warts digitate**	3	0.00	0.00
	**atopic dermatitis hyperlinear creases**	2	0.00	0.00
	**tinea ringworm foot plantar**	2	0.00	0.00
	**tinea ringworm foot webs**	2	0.00	0.00
	**tinea ringworm incognito**	2	0.00	0.00
	**ulcus cruris venosum**	2	0.00	0.00
	**eczema acute**	1	0.00	0.00
	**eczema chronic**	1	0.00	0.00
	**hematogenous allergic contact dermatitis**	1	0.00	0.00
	**interstitial granulomatous dermatitis**	1	0.00	100.00
	**lichen planus drug**	1	0.00	0.00
	**lichen planus vaginal**	1	0.00	0.00
	**lupus erythematosus chronicus discoides**	1	0.00	0.00
	**lupus erythematosus mucosae oris**	1	0.00	0.00
	**psoriasis nail psoriasis**	1	0.00	0.00
	**psoriasis palmoplantaris**	1	0.00	0.00
	**psoriasis vulgaris guttate type**	1	0.00	0.00
	**tinea ringworm nigra**	1	0.00	0.00
	**tinea ringworm primary lesion**	1	0.00	0.00
	**warts common**	1	0.00	0.00
	**systemic carcinomas**	431	0.57	9.98
**Malignant dermal tumor**	**intermediate or malignant vascular tumors**	172	0.23	22.09

	dermatofibrosarcoma protuberans	89	0.12	8.99
	merkel cell carcinoma	59	0.08	3.39
	sarcoma	34	0.04	2.94
	malignant adnexal tumors	21	0.03	9.52
	abrikossofs tumour of skin	16	0.02	12.50
	soft tissue tumors	15	0.02	6.67
	adenocarcinoma in the submandibular salivary glands	5	0.01	0.00
Pigmented lesions benign	melanocytic nevi	1359	1.79	4.19
	blue nevus	651	0.86	4.76
	intradermal nevus	439	0.58	8.88
	atypical nevi	385	0.51	10.13
	congenital nevus	138	0.18	6.52
	halo nevus	105	0.14	14.29
	nevus incipiens	105	0.14	2.86
	nevus spilus	66	0.09	9.09
	café au lait spot	65	0.09	9.23
	melanosis neurocutanea	65	0.09	16.92
	solar lentigo	60	0.08	3.33
	combined nevus	51	0.07	0.00
	mongolian spot	50	0.07	6.00
	nevocytic nevus	46	0.06	4.35
	naevus blue	44	0.06	11.36
	giant melanocytic nevus	40	0.05	17.50
	black heel	39	0.05	10.26
	melanonychia striata	38	0.05	7.89
	mucosal lentigines	36	0.05	0.00
	blue nevus ota	35	0.05	17.14
	spindle cell nevus	35	0.05	11.43
	naevus halo	34	0.04	20.59
	ephelides	33	0.04	3.03
	nevus cell nevus	30	0.04	16.67
	hereditary dysplastic nevus syndrome	29	0.04	20.69

	**naevus achromic**	28	0.04	7.14
	**melanoma mimic**	26	0.03	0.00
	**lentigo senilis**	25	0.03	0.00
	**lentigo simplex**	25	0.03	0.00
	**melanotic macule**	22	0.03	4.55
	**spitz nevus**	20	0.03	0.00
	**nevus repigmented**	19	0.03	10.53
	**nail nevus**	18	0.02	11.11
	**ink spot nevus**	16	0.02	0.00
	**naevus of ota bilateral type**	15	0.02	0.00
	**nevus pigmentosus et pilosus**	13	0.02	7.69
	**naevus giant pigmented**	11	0.01	0.00
	**labial melanotic macule**	4	0.01	25.00
**Pigmented lesions malignant**	**malignant melanoma**	1166	1.54	7.29
	**lentigo maligna melanoma**	97	0.13	2.06
	**lentigo maligna**	64	0.08	6.25
	**nail melanoma**	51	0.07	7.84
	**metastatic melanoma**	45	0.06	13.33
	**nodular melanoma nm**	35	0.05	0.00
	**superficial spreading melanoma ssm**	24	0.03	0.00
	**amelanotic malignant melanoma amm**	19	0.03	0.00
	**melanoma of the parotid**	11	0.01	18.18
	**mucosal melanoma**	9	0.01	11.11
	**acrolentiginous melanoma alm**	7	0.01	14.29
	**malignant melanoma metastatic**	3	0.00	0.00
	**All**	75730	100.00	11.96

Supplementary Table 4: Frequency Table and False Positive Rates of Diagnoses in the Danderm Repository

Frequency table of diagnoses and False Positive Rates of evaluated skin lesion images in the Danderm repository.
All, Number of all evaluated skin lesion images in the Danderm repository; FPR, False positive rate (%) of the diagnosis in the Danderm repository; n, Number of occurrences of diagnosis; Unknown, Diagnosis unknown or unmapped; %, Percentage of diagnosis in relation to all evaluated skin lesion images in the Danderm repository.

Category	Diagnosis	n	%	FPR
Benign dermal tumors cysts sinuses	hemangioma	44	1.28	0.00
	pyogenic granuloma	10	0.29	0.00
	dermatofibroma	8	0.23	12.50
	nevus flammeus	8	0.23	0.00
	sebaceous hyperplasia	6	0.17	0.00
	leiomyoma	5	0.15	0.00
	myxoid cyst	5	0.15	40.00
	skin tag	5	0.15	0.00
	cylindroma	3	0.09	0.00
	lymphangioma	3	0.09	0.00
	pilar cyst	3	0.09	0.00
	spider angioma	1	0.03	0.00
Cutaneous lymphoma and lymphoid infiltrates	cutaneous t cell lymphoma	19	0.55	0.00
	erythema arciforme et palpabile migrans	11	0.32	9.09
	lymphomas lymphocytic infiltrations	10	0.29	0.00
	lymphoma b cell	2	0.06	0.00
Epidermal tumors hamartomas milia and growths benign	seborrheic keratosis	29	0.84	3.45
	epidermal nevus	12	0.35	0.00
	porokeratosis	11	0.32	0.00
	dermatosis papulosa nigra	9	0.26	11.11
	prurigo nodularis	7	0.20	57.14
	milia	5	0.15	20.00

Epidermal tumors pre malignant and malignant	**basal cell carcinoma**	51	1.49	0.00
	**actinic keratosis**	28	0.82	0.00
	**squamous cell carcinoma**	16	0.47	0.00
	**bowens disease**	9	0.26	0.00
	**keratoacanthoma**	9	0.26	0.00
	**cutaneous horn**	7	0.20	0.00
	**actinic cheilitis**	5	0.15	0.00
	**pagets disease**	5	0.15	0.00
	**extramammary paget disease**	3	0.09	0.00
Genodermatoses and supernumerary growths	**dariers disease**	13	0.38	7.69
	**congenital ichthyosiform erythroderma**	9	0.26	0.00
	**ichthyosis**	8	0.23	0.00
	**incontinentia pigmenti**	6	0.17	0.00
	**rendu osler syndrome**	6	0.17	0.00
	**neurofibromatosis**	5	0.15	0.00
	**pseudoxanthoma elasticum**	5	0.15	0.00
	**acrokeratosis verruciformis**	4	0.12	0.00
	**erythrokeratodermia variabilis**	4	0.12	0.00
Inflammatory	**allergic contact dermatitis**	129	3.76	3.10
	**irritant contact dermatitis**	93	2.71	8.60
	**bacterial infections**	91	2.65	2.20
	**atopic dermatitis**	88	2.56	1.14
	**lichen planus**	76	2.21	2.63
	**acne vulgaris**	75	2.19	5.33
	**fungal infections dermal and subcut**	68	1.98	1.47
	**seborrheic dermatitis**	65	1.89	0.00
	**psoriasis**	64	1.86	4.69
	**drug eruption**	53	1.54	5.66
	**virus exanthem**	48	1.40	4.17
	**non scarring alopecia**	46	1.34	4.35
	**bullous pemphigoid**	39	1.14	7.69
	**urticaria**	39	1.14	7.69
	**lichen**	34	0.99	2.94

**nail trauma**	29	0.84	0.00
**erythema multiforme and fixed drug eruptions**	26	0.76	0.00
**rosacea**	26	0.76	0.00
**impetigo**	24	0.70	0.00
**lupus erythematosus systemic**	24	0.70	0.00
**ulcer**	24	0.70	0.00
**purpura**	23	0.67	0.00
**herpes zoster**	22	0.64	4.55
**planar warts**	22	0.64	0.00
**psoriasis chronic plaque**	22	0.64	0.00
**mastocytosis**	20	0.58	0.00
**psoriasis nails**	20	0.58	0.00
**genital warts**	18	0.52	0.00
**scleroderma**	18	0.52	0.00
**sun damaged skin**	18	0.52	0.00
**varicella**	16	0.47	12.50
**onychomycosis**	15	0.44	0.00
**scarring alopecia**	15	0.44	0.00
**acne cystic**	14	0.41	0.00
**dermatomyositis**	14	0.41	7.14
**herpes simplex**	14	0.41	0.00
**parapsoriasis en plaques**	14	0.41	0.00
**psoriasis scalp**	14	0.41	7.14
**tinea manus**	14	0.41	0.00
**tinea versicolor**	14	0.41	0.00
**candidiasis**	13	0.38	7.69
**granuloma annulare**	13	0.38	7.69
**molluscum contagiosum**	13	0.38	15.38
**vasculitis**	13	0.38	0.00
**wart**	13	0.38	7.69
**alopecia**	12	0.35	8.33
**contact dermatitis**	12	0.35	0.00

**eczema herpeticum**	12	0.35	0.00
**herpes simplex genitalis**	12	0.35	0.00
**miliaria**	12	0.35	0.00
**neurotic excoriations**	12	0.35	0.00
**pityriasis rubra pilaris**	12	0.35	0.00
**psoriasis hand**	12	0.35	0.00
**scabies**	12	0.35	0.00
**factitial dermatitis**	11	0.32	0.00
**hyperhidrosis**	11	0.32	0.00
**photodermatoses**	11	0.32	9.09
**stomatitis**	11	0.32	0.00
**sunburn**	11	0.32	18.18
**syphilis**	10	0.29	0.00
**sarcoidosis**	9	0.26	0.00
**acne keloidalis**	8	0.23	0.00
**biting fleas**	8	0.23	12.50
**discoid lupus erythematosus**	8	0.23	0.00
**erysipelas**	8	0.23	0.00
**erythema craquele**	8	0.23	25.00
**erythrasma**	8	0.23	0.00
**necrobiosis lipoidica**	8	0.23	0.00
**poikiloderma civatte**	8	0.23	0.00
**psoriasis arthropathica**	8	0.23	0.00
**acne fulminans**	7	0.20	0.00
**acne scar**	7	0.20	0.00
**eczema nummular**	7	0.20	0.00
**lichen planopilaris**	7	0.20	0.00
**lichen planus penis**	7	0.20	0.00
**pernio and mimics**	7	0.20	14.29
**pincer nails**	7	0.20	0.00
**pityriasis rosea**	7	0.20	0.00
**cholinergic urticaria**	6	0.17	0.00
**clubbing**	6	0.17	0.00

**connective tissue disorders**	6	0.17	0.00
**diabetic ulcer**	6	0.17	0.00
**eczema**	6	0.17	0.00
**hidradenitis**	6	0.17	0.00
**lupus vulgaris**	6	0.17	0.00
**pseudofolliculitis barbae**	6	0.17	0.00
**psoriatic erythroderma**	6	0.17	16.67
**raynauds disease**	6	0.17	0.00
**reiters disease**	6	0.17	0.00
**solar elastosis**	6	0.17	0.00
**staphylococcal folliculitis**	6	0.17	0.00
**behcets disease**	5	0.15	20.00
**eczema acute**	5	0.15	0.00
**eczema hand**	5	0.15	0.00
**guttate psoriasis**	5	0.15	20.00
**hand foot mouth**	5	0.15	80.00
**inverse psoriasis**	5	0.15	20.00
**koilonychia**	5	0.15	0.00
**leukonychia**	5	0.15	0.00
**lichen nitidus**	5	0.15	0.00
**lichen planus hypertrophic**	5	0.15	20.00
**lupus erythematosus subacute cutaneous lupus erythematosus**	5	0.15	0.00
**onychodystrophia mediana canaliformis**	5	0.15	0.00
**papulose bowenoide**	5	0.15	0.00
**relapsing polychondritis**	5	0.15	0.00
**scarring**	5	0.15	0.00
**steroid acne**	5	0.15	0.00
**tinea faciei**	5	0.15	0.00
**caterpillar dermatitis**	4	0.12	0.00
**folliculitis**	4	0.12	0.00
**onycholysis**	4	0.12	0.00
**staphylococcal scalded skin syndrome**	4	0.12	0.00

	xanthomas	4	0.12	0.00
	acne neonatorum	3	0.09	0.00
	balanitis	3	0.09	0.00
	cimicosis	3	0.09	0.00
	dermatitis herpetiformis	3	0.09	33.33
	erythema infectiosum	3	0.09	0.00
	erythroderma	3	0.09	0.00
	gangrene	3	0.09	0.00
	geographic tongue	3	0.09	0.00
	pemphigus foliaceus	3	0.09	0.00
	pemphigus vulgaris	3	0.09	0.00
	pitted keratolysis	3	0.09	0.00
	stasis dermatitis	3	0.09	0.00
	sycosis barbae	3	0.09	0.00
	tinea cruris	3	0.09	0.00
	thermal burn	2	0.06	0.00
	trichostasis spinulosa	2	0.06	0.00
	half and half nail	1	0.03	0.00
	nevus cell nevus	27	0.79	7.41
Pigmented lesions benign	naevus halo	6	0.17	16.67
	black heel	3	0.09	0.00
	malignant melanoma	28	0.82	3.57
	nodular melanoma nm	14	0.41	7.14
Pigmented lesions malignant	superficial spreading melanoma ssm	5	0.15	0.00
	lentigo maligna	4	0.12	0.00
	malignant melanoma metastatic	3	0.09	33.33
	Unknown	840	24.48	3.57
	All	3432	100.00	3.35

Supplementary Table 5: Frequency Table and False Positive Rates of Diagnoses in the DermIS Repository

Frequency table of diagnoses and False Positive Rates of evaluated skin lesion images in the DermIS repository.
All, Number of all evaluated skin lesion images in the DermIS repository; FPR, False positive rate (%) of the diagnosis in the DermIS repository; n, Number of occurrences of diagnosis; Unknown, Diagnosis unknown or unmapped; %, percentage of diagnosis in relation to all evaluated skin lesion images in the DermIS repository.

Category	Diagnosis	n	%	FPR
Benign dermal tumors cysts sinuses	**hemangioma**	40	0.61	2.50
	**dermatofibroma**	14	0.21	14.29
	**lymphangioma**	14	0.21	0.00
	**pilomatricoma**	13	0.20	15.38
	**adenoma sebaceum**	11	0.17	0.00
	**glomus tumor**	11	0.17	0.00
	**pilar cyst**	11	0.17	9.09
	**granuloma pyogenic**	10	0.15	10.00
	**telangiectases**	10	0.15	0.00
	**becker nevus**	8	0.12	0.00
	**neurolemmoma**	8	0.12	0.00
	**syringoma**	8	0.12	0.00
	**trichoepithelioma**	8	0.12	0.00
	**acrotrichoma**	7	0.11	14.29
	**trichoepithelioma papulosum multiplex**	7	0.11	0.00
	**connective tissue nevus**	6	0.09	0.00
	**cylindroma**	6	0.09	0.00
	**hemangioma racemosum**	6	0.09	16.67
	**hidrocystoma**	6	0.09	0.00
	**angioma serpiginosum**	5	0.08	20.00
	**fibroma**	5	0.08	20.00
	**aponeurotic fibroma**	4	0.06	0.00
	**ectopic respiratory epithelia**	4	0.06	0.00
	**infantile digital fibromatosis**	4	0.06	0.00

	**klippel trenaunay syndrome**	4	0.06	0.00
	**nevus lipomatosis**	4	0.06	0.00
	**sebaceous hyperplasia**	4	0.06	0.00
	**spider nevus**	4	0.06	25.00
	**spiradenoma**	4	0.06	0.00
	**angiokeratomas**	3	0.05	0.00
	**leiomyoma**	3	0.05	33.33
	**port wine stain**	3	0.05	0.00
	**pyogenic granuloma**	3	0.05	0.00
	**scrotal cyst**	3	0.05	0.00
	**trichoblastoma**	3	0.05	0.00
	**ganglion**	2	0.03	0.00
	**mucous cyst**	2	0.03	0.00
	**pilonidal sinus**	2	0.03	0.00
	**vascular anomaly**	2	0.03	0.00
	**cutis marmorata telangiectatica congenita**	1	0.02	0.00
	**myxoid cyst**	1	0.02	0.00
	**nevus flammeus**	1	0.02	0.00
	**spider angioma**	1	0.02	0.00
	**trichoadenoma**	1	0.02	0.00
	**trichofolliculoma**	1	0.02	0.00
	**venous lake**	1	0.02	0.00
**Cutaneous lymphoma and lymphoid infiltrates**	**cutaneous t cell lymphoma**	88	1.34	4.55
	**lymphoma b cell**	35	0.53	0.00
	**erythema arciforme et palpabile migrans**	12	0.18	8.33
	**lymphomatoid papulosis**	10	0.15	40.00
	**pseudo lymphoma other types**	7	0.11	0.00
**Epidermal tumors hamartomas milia and growths benign**	**epidermal nevus**	44	0.67	11.36
	**seborrheic keratosis**	33	0.50	0.00
	**porokeratosis**	26	0.39	0.00
	**acral keratosis**	7	0.11	14.29
	**prurigo nodularis**	5	0.08	80.00

	**stucco keratosis**	5	0.08	0.00
	**clear cell acanthoma**	3	0.05	0.00
	**giant comedo**	3	0.05	0.00
	**milia**	3	0.05	0.00
**Epidermal tumors pre malignant and malignant**	**squamous cell carcinoma**	78	1.18	1.28
	**solid cystic basal cell carcinoma**	63	0.96	0.00
	**basal cell carcinoma**	57	0.87	1.75
	**bowens disease**	47	0.71	0.00
	**basal cell carcinoma morpheiform**	46	0.70	4.35
	**actinic cheilitis**	26	0.39	0.00
	**actinic keratosis**	26	0.39	3.85
	**carcinoma of lip**	19	0.29	0.00
	**basal cell carcinoma ulcerating**	18	0.27	0.00
	**keratoacanthoma**	15	0.23	0.00
	**pigmented basal cell carcinoma**	13	0.20	0.00
	**superficial basal cell carcinoma**	11	0.17	0.00
	**leucoplakia praecancerosa**	9	0.14	0.00
	**ulcus terebrans**	8	0.12	0.00
	**basal cell nevus syndrome**	6	0.09	0.00
	**cutaneous horn**	4	0.06	0.00
	**arsenical keratoses**	2	0.03	0.00
	**erythroplasia queyrat**	2	0.03	0.00
	**extramammary paget disease**	2	0.03	0.00
	**hypertrophic actinic keratosis**	2	0.03	0.00
	**leukoplakia**	2	0.03	0.00
	**pagets disease**	2	0.03	0.00
	**verrucous carcinoma**	2	0.03	0.00
	**basal cell carcinoma nose**	1	0.02	0.00
**Genodermatoses and supernumerary growths**	**neurofibromatosis**	40	0.61	17.50
	**dariers disease**	35	0.53	0.00
	**incontinentia pigmenti**	31	0.47	9.68
	**ichthyosis congenita**	27	0.41	3.70
	**erythrokeratodermia variabilis**	23	0.35	4.35

	epidermolysis bullosa	19	0.29	5.26
	rothmund thomson syndrome	15	0.23	0.00
	collodion baby	14	0.21	0.00
	lipoid proteinosis urbach wiethe disease	13	0.20	7.69
	epidermolytic hyperkeratosis	12	0.18	0.00
	x linked ichthyosis	11	0.17	0.00
	xeroderma pigmentosum	11	0.17	9.09
	acrodermatitis enteropathica	10	0.15	10.00
	ehlers danlos syndrome	10	0.15	0.00
	leopard syndrome	10	0.15	10.00
	pseudoxanthoma elasticum	10	0.15	0.00
	rendu osler syndrome	9	0.14	33.33
	vascular tumors and malformations	9	0.14	11.11
	hailey hailey disease	8	0.12	0.00
	congenital dyskeratosis	7	0.11	0.00
	congenital ichthyosiform erythroderma	6	0.09	0.00
	congenital erythropoietic porphyria	5	0.08	0.00
	erythrodermia exfoliativa leiner	5	0.08	20.00
	ichthyosis vulgaris	5	0.08	20.00
	knuckle pads	5	0.08	0.00
	tuberous sclerosis	5	0.08	20.00
	acrokeratoelastoidosis	4	0.06	0.00
	laugier hunziker s syndrome	4	0.06	0.00
	acrokeratosis verruciformis	3	0.05	0.00
	buschke ollendorf syndrome	3	0.05	0.00
	ichthyosis	3	0.05	66.67
	leprechaunism syndrome	3	0.05	0.00
	peutz jeghers syndrome	3	0.05	0.00
	phakomatosis pigmentovascularis	3	0.05	0.00
	supernumerary growths	2	0.03	0.00
	sjgren larsson syndrome	1	0.02	0.00
Inflammatory	atopic eczema	141	2.14	7.09
	pustular skin disease non infectious	91	1.38	2.20

**lichen planus**	84	1.27	9.52
**vasculitis**	80	1.21	0.00
**malformations of extremities**	71	1.08	4.23
**allergic contact dermatitis**	69	1.05	0.00
**bullous pemphigoid**	67	1.02	7.46
**photodermatoses**	60	0.91	3.33
**psoriasis vulgaris chronic stationary type**	59	0.90	3.39
**sarcoidosis**	56	0.85	1.79
**toxic epidermal necrolysis and stevens johnson**	54	0.82	5.56
**dermatomyositis**	53	0.80	0.00
**depositional diseases calcinosis cutis amyloid mucinosis gout and others**	50	0.76	4.00
**lichen sclerosus skin**	50	0.76	0.00
**atrophic skin disease striae and elastic fiber disorders**	49	0.74	4.08
**panniculitis lipodystrophy and diseases of subcutis**	48	0.73	8.33
**subacute cutaneous lupus erythematosus scle**	46	0.70	0.00
**verruca vulgaris**	45	0.68	8.89
**histiocytic disorders and proliferations**	44	0.67	15.91
**drug eruption**	43	0.65	0.00
**cheilitis**	41	0.62	0.00
**granuloma annulare**	40	0.61	2.50
**mastocytosis**	40	0.61	7.50
**discoid lupus erythematosus**	39	0.59	5.13
**pemphigus mucosae**	39	0.59	0.00
**lichen planus of the mucosa**	38	0.58	0.00
**lupus subacute**	38	0.58	2.63
**pemphigus vulgaris**	38	0.58	10.53
**psoriasis inversa**	38	0.58	0.00
**progressive systemic scleroderma**	37	0.56	5.41
**psoriasis vulgaris nail changes**	37	0.56	0.00

**dermatitis herpetiformis**	36	0.55	11.11
**herpes zoster**	36	0.55	5.56
**syphilis**	36	0.55	11.11
**pigmentation diseases**	34	0.52	5.88
**acanthosis nigricans and carp**	33	0.50	9.09
**hair disorderstrichorrhexis nodosa etc**	33	0.50	0.00
**scarring alopecia**	31	0.47	0.00
**gianotti crosti**	29	0.44	27.59
**impetigo**	29	0.44	10.34
**perioral dermatitis**	29	0.44	0.00
**scarring**	29	0.44	6.90
**tattoo and foreign body reactions**	29	0.44	3.45
**tinea corporis**	29	0.44	3.45
**livedo reticularis**	28	0.42	3.57
**non scarring alopecia**	28	0.42	7.14
**radiodermatitis**	28	0.42	3.57
**pernio and mimics**	27	0.41	0.00
**cutaneous manifestations of systemic disease**	26	0.39	19.23
**lichen planus of the mucosa erosive**	26	0.39	0.00
**pityriasis rubra pilaris**	26	0.39	0.00
**porphyria**	26	0.39	3.85
**rhinophyma**	26	0.39	0.00
**localized scleroderma**	25	0.38	8.00
**urticaria**	25	0.38	4.00
**acne cystica**	23	0.35	17.39
**pityriasis versicolor**	23	0.35	4.35
**scabies**	23	0.35	8.70
**factitial dermatitis**	22	0.33	13.64
**herpes simplex**	22	0.33	13.64
**lichen planus exanthematicus**	22	0.33	4.55
**varicella**	22	0.33	4.55
**xanthomas**	22	0.33	9.09

**acne conglobata**	21	0.32	4.76
**erysipelas**	21	0.32	0.00
**gyrate erythemas**	21	0.32	9.52
**pigmented purpuric dermatosis**	21	0.32	4.76
**psoriasis vulgaris guttate type**	21	0.32	0.00
**molluscum contagiosum**	20	0.30	35.00
**pityriasis lichenoides and pleva**	20	0.30	5.00
**atypical mycobacterium**	19	0.29	10.53
**solar purpura**	19	0.29	0.00
**balanitis**	18	0.27	0.00
**chronic recurrent aphthae**	18	0.27	0.00
**fungal infections dermal and subcut**	18	0.27	11.11
**condyloma acuminata**	17	0.26	0.00
**keratosis pilaris and related follicular disorders**	17	0.26	5.88
**cimicosis**	16	0.24	18.75
**erythema multiforme and fixed drug eruptions**	16	0.24	0.00
**herpes gestationis**	16	0.24	6.25
**lupus vulgaris**	16	0.24	0.00
**necrobiosis lipoidica**	16	0.24	6.25
**onychomycosis**	16	0.24	0.00
**lymphedema**	15	0.23	6.67
**nummular eczema**	15	0.23	13.33
**panarteritis nodosa kussmaul maier**	15	0.23	0.00
**pemphigus foliaceus**	15	0.23	0.00
**scleromyxedema**	15	0.23	6.67
**twenty nail dystrophy**	15	0.23	0.00
**morphea linear**	14	0.21	14.29
**pityriasis rosea**	14	0.21	0.00
**raynauds disease**	14	0.21	0.00
**rosacea**	14	0.21	0.00
**furuncle**	13	0.20	7.69
**diseases of bone**	12	0.18	0.00

**geographic tongue**	12	0.18	0.00
**nematode infection**	12	0.18	0.00
**perforating disorders**	12	0.18	8.33
**acne papulopustulosa**	11	0.17	0.00
**disseminated lokalized scleroderma**	11	0.17	0.00
**dry skin eczema**	11	0.17	0.00
**eczema herpeticum**	11	0.17	18.18
**erythroderma**	11	0.17	0.00
**hand foot mouth**	11	0.17	0.00
**hypereosinophilic syndrome**	11	0.17	18.18
**lichen**	11	0.17	9.09
**metabolic and nutritional diseases**	11	0.17	0.00
**thermal burn**	11	0.17	0.00
**acne inversa**	10	0.15	10.00
**acne vulgaris**	10	0.15	10.00
**benign mucosal pemphigoid**	10	0.15	0.00
**caterpillar dermatitis**	10	0.15	10.00
**chronic venous insufficiency grade ii**	10	0.15	0.00
**erythema elevatum diutinum**	10	0.15	30.00
**erythema infectiosum**	10	0.15	0.00
**tinea pedis**	10	0.15	0.00
**angiolymphoid hyperplasia with eosinophilia**	9	0.14	11.11
**diabetic gangrene**	9	0.14	0.00
**diseases of the ear**	9	0.14	11.11
**epidermolysis bullosa acquisita**	9	0.14	11.11
**erysipelas bullosum**	9	0.14	0.00
**herpes simplex genitalis**	9	0.14	22.22
**lichen nitidus**	9	0.14	22.22
**lichen planus verrucosus**	9	0.14	0.00
**neurotic excoriations**	9	0.14	0.00
**reiters disease**	9	0.14	11.11
**tinea capitis**	9	0.14	11.11

**behcets disease**	8	0.12	0.00
**hecks disease**	8	0.12	0.00
**hidradenitis**	8	0.12	0.00
**hidroa vacciniforme**	8	0.12	0.00
**ingrown nail**	8	0.12	0.00
**lupus erythematosus tumidus**	8	0.12	0.00
**morphea**	8	0.12	0.00
**morsicatio buccarum**	8	0.12	0.00
**neurotrophic ulcer**	8	0.12	0.00
**pemphigus seborrhoicus**	8	0.12	0.00
**seborrheic dermatitis**	8	0.12	0.00
**thrombangiitis obliterans**	8	0.12	0.00
**urticarial vasculitis**	8	0.12	0.00
**virus exanthem**	8	0.12	0.00
**acne fulminans**	7	0.11	0.00
**candidiasis mouth**	7	0.11	0.00
**crest syndrome**	7	0.11	0.00
**dyshidrotic eczema**	7	0.11	28.57
**eosinophilic ulcer of the tongue**	7	0.11	0.00
**erythema ab igne**	7	0.11	0.00
**gloves and socks syndrome**	7	0.11	0.00
**hyperkeratotic fissured hand and foot eczema**	7	0.11	0.00
**insect bite**	7	0.11	14.29
**lichen planus annularis**	7	0.11	14.29
**lupus erythematosus mucosae oris**	7	0.11	0.00
**measles**	7	0.11	14.29
**psoriasis palmoplantaris**	7	0.11	0.00
**purpura**	7	0.11	0.00
**solar damage atrophy**	7	0.11	0.00
**staphylococcal scalded skin syndrome**	7	0.11	0.00
**ulcus cruris venosum**	7	0.11	0.00
**varicosis**	7	0.11	14.29

**acroangiodermatitis**	6	0.09	16.67
**ashy dermatosis**	6	0.09	0.00
**atopic eczema of the hands**	6	0.09	0.00
**decubitus ulcer**	6	0.09	16.67
**degos disease**	6	0.09	0.00
**diaper dermatitis**	6	0.09	0.00
**eczema anal**	6	0.09	0.00
**granuloma**	6	0.09	0.00
**lupus miliaris disseminatus faciei**	6	0.09	16.67
**miliaria**	6	0.09	0.00
**pachyonychia congenita hereditaria**	6	0.09	0.00
**papulose bowenoide**	6	0.09	0.00
**parapsoriasis en plaques**	6	0.09	0.00
**psoriasis arthropathica**	6	0.09	0.00
**psoriasis hand**	6	0.09	0.00
**acne comedonica**	5	0.08	0.00
**autoerythrocyte sensitization**	5	0.08	20.00
**cercaria dermatitis**	5	0.08	20.00
**eczema**	5	0.08	0.00
**gingival disease**	5	0.08	0.00
**hematogenous allergic contact dermatitis**	5	0.08	0.00
**herpes simplex analis**	5	0.08	0.00
**herpes simplex oralis**	5	0.08	20.00
**leukoedema oral**	5	0.08	0.00
**median nail dystrophy**	5	0.08	0.00
**onychodystrophia mediana canaliformis**	5	0.08	0.00
**onychogryphosis**	5	0.08	0.00
**poikiloderma civatte**	5	0.08	0.00
**psoriatic erythroderma**	5	0.08	0.00
**scrophuloderma**	5	0.08	20.00
**solar elastosis**	5	0.08	0.00
**stasis dermatitis**	5	0.08	0.00

**tuberculosis**	5	0.08	0.00
**ulcus cruris mixtum**	5	0.08	0.00
**zoster gangraenosus**	5	0.08	0.00
**acanthoma fissuratum**	4	0.06	0.00
**alopecia**	4	0.06	0.00
**angioedema**	4	0.06	0.00
**chronic cutaneous graft versus host reaction**	4	0.06	0.00
**contact dermatitis**	4	0.06	0.00
**culicosis bullosa**	4	0.06	0.00
**cutis marmorata**	4	0.06	0.00
**ecthyma simplex**	4	0.06	0.00
**eosinophilic pustular folliculitis**	4	0.06	0.00
**erythrasma**	4	0.06	0.00
**half and half nail**	4	0.06	0.00
**lichen simplex**	4	0.06	0.00
**linear scleroderma**	4	0.06	0.00
**lupus acute**	4	0.06	0.00
**myxoedema pretibial**	4	0.06	0.00
**primary tufted hairs**	4	0.06	0.00
**pyoderma faciale**	4	0.06	0.00
**solar damage favre racouchot**	4	0.06	0.00
**tinea barbae profunda**	4	0.06	0.00
**tinea faciei**	4	0.06	0.00
**verrucae planae juveniles**	4	0.06	0.00
**atrophy blanche**	3	0.05	0.00
**candidiasis chronic mucocutaneos**	3	0.05	0.00
**clubbing**	3	0.05	0.00
**cow pox**	3	0.05	0.00
**diseases of the eye**	3	0.05	0.00
**eczema hand**	3	0.05	0.00
**folliculitis**	3	0.05	0.00
**gram negative infection of the foot**	3	0.05	0.00

**hairy tongue**	3	0.05	0.00
**herpes simplex labialis**	3	0.05	0.00
**herpes type primary**	3	0.05	33.33
**myiasis**	3	0.05	0.00
**pemphigus vegetans**	3	0.05	0.00
**reticular erythematous mucinosis**	3	0.05	66.67
**sjogrens syndrome**	3	0.05	0.00
**steroid acne**	3	0.05	0.00
**tinea capitis superficialis**	3	0.05	0.00
**ulcer**	3	0.05	0.00
**urticaria factitia**	3	0.05	0.00
**acne excoriee**	2	0.03	0.00
**acne neonatorum**	2	0.03	0.00
**atopic dermatitis infant phase**	2	0.03	0.00
**candidiasis**	2	0.03	0.00
**eczema fingertips**	2	0.03	0.00
**erythema craquele**	2	0.03	0.00
**fox fordyce disease**	2	0.03	0.00
**gangrene**	2	0.03	0.00
**gonorrhea**	2	0.03	50.00
**herpetic gingivostomatite**	2	0.03	0.00
**intertrigo**	2	0.03	0.00
**leprosy**	2	0.03	50.00
**lichen planopilaris**	2	0.03	50.00
**lingua plicata**	2	0.03	0.00
**metal dermatitis**	2	0.03	0.00
**mucous membrane psoriasis**	2	0.03	0.00
**neurodermatitis**	2	0.03	0.00
**onycholysis**	2	0.03	0.00
**pediculosis lids**	2	0.03	0.00
**psoriasis infants**	2	0.03	0.00
**purpura fulminans**	2	0.03	50.00
**relapsing polychondritis**	2	0.03	0.00

**scarlet fever**	2	0.03	0.00
**solar damage cutis rhomboidalis nuchae**	2	0.03	0.00
**stomatitis**	2	0.03	0.00
**sunburn**	2	0.03	0.00
**tick bite**	2	0.03	0.00
**tinea capitis profunda**	2	0.03	0.00
**tinea manus**	2	0.03	0.00
**acne infantile**	1	0.02	0.00
**acne pustular**	1	0.02	0.00
**allergic hand eczema**	1	0.02	0.00
**cat pox**	1	0.02	0.00
**cellulitis**	1	0.02	0.00
**chancroid**	1	0.02	0.00
**dermatitis ekzema**	1	0.02	0.00
**dermatosis neglecta**	1	0.02	0.00
**eczema areola**	1	0.02	0.00
**eczema chronic**	1	0.02	0.00
**eczema lids**	1	0.02	0.00
**eczematid**	1	0.02	0.00
**elephantiasis nostras verrucosa**	1	0.02	0.00
**epidermodysplasia verruciformis**	1	0.02	0.00
**erysipeloid**	1	0.02	0.00
**fourniers gangrene**	1	0.02	100.00
**grovers disease**	1	0.02	0.00
**herpes cutaneous**	1	0.02	0.00
**irritant contact dermatitis**	1	0.02	0.00
**ischemia**	1	0.02	0.00
**kawasaki syndrome**	1	0.02	0.00
**koilonychia**	1	0.02	0.00
**nail pigmentation**	1	0.02	0.00
**nail psoriasis**	1	0.02	0.00
**nail trauma**	1	0.02	0.00
**onychogryphosis and onychauxis**	1	0.02	0.00

	paronychia	1	0.02	0.00
	pemphigus	1	0.02	0.00
	peripheral occlusive arterial disease	1	0.02	0.00
	pityrosporum folliculitis	1	0.02	0.00
	planar warts	1	0.02	0.00
	polychondritis	1	0.02	0.00
	primary herpes simplex virus	1	0.02	100.00
	psoriasis	1	0.02	0.00
	psoriasis penis	1	0.02	0.00
	rheumatoid nodule	1	0.02	100.00
	subacute cutaneous lupus erythematosus	1	0.02	0.00
	tinea faciale	1	0.02	0.00
	trichomycosis	1	0.02	0.00
Malignant dermal tumor	systemic carcinomas	33	0.50	6.06
	dermatofibrosarcoma protuberans	9	0.14	0.00
	sarcoma	7	0.11	0.00
	abrikossofs tumour of skin	6	0.09	16.67
	intermediate or malignant vascular tumors	6	0.09	0.00
	merkel cell carcinoma	3	0.05	0.00
Pigmented lesions benign	nevocytic nevus	55	0.83	1.82
	nevus pigmentosus et pilosus	14	0.21	0.00
	halo nevus	13	0.20	23.08
	melanosis neurocutanea	13	0.20	23.08
	blue nevus	11	0.17	0.00
	melanonychia striata	10	0.15	0.00
	nevus spilus	8	0.12	0.00
	hereditary dysplastic nevus syndrome	6	0.09	16.67
	mucosal lentigines	6	0.09	0.00
	mongolian spot	5	0.08	0.00
	spitz nevus	5	0.08	20.00
	atypical nevi	4	0.06	0.00
	ephelides	4	0.06	0.00

	**giant melanocytic nevus**	4	0.06	25.00
	**congenital nevus**	3	0.05	0.00
	**black heel**	2	0.03	0.00
	**café au lait spot**	2	0.03	0.00
	**lentigo senilis**	2	0.03	0.00
	**blue nevus ota**	1	0.02	0.00
	**melanotic macule**	1	0.02	0.00
**Pigmented lesions malignant**	**superficial spreading melanoma ssm**	138	2.09	3.62
	**acrolentiginous melanoma alm**	27	0.41	0.00
	**lentigo maligna**	20	0.30	5.00
	**lentigo maligna melanoma**	16	0.24	0.00
	**malignant melanoma metastatic**	15	0.23	0.00
	**nodular melanoma nm**	10	0.15	0.00
	**amelanotic malignant melanoma amm**	7	0.11	0.00
	**malignant melanoma**	7	0.11	14.29
	**mucosal melanoma**	3	0.05	0.00
	**nail melanoma**	3	0.05	0.00
	**Unknown**	608	9.23	5.10
	**All**	6589	100.00	4.78

Supplementary Table 6: Frequency Table and False Positive Rates of Diagnoses in the DermNet dataset

Frequency table of diagnoses and False Positive Rates of evaluated skin lesion images in the DermNet dataset.
All, Number of all evaluated skin lesion images in the DermNet dataset; FPR, False positive rate (%) of the diagnosis in the DermNet dataset; n, Number of occurrences of diagnosis; Unknown, Diagnosis unknown or unmapped; %, Percentage of diagnosis in relation to all evaluated skin lesion images in the DermNet dataset.

Category	Diagnosis	n	%	FPR
	**pilar cyst**	167	0.87	14.97
	**dermatofibroma**	135	0.70	5.19
	**pyogenic granuloma**	112	0.58	6.25
	**sebaceous hyperplasia**	107	0.55	2.80
	**mucous cyst**	103	0.53	4.85
	**skin tag**	97	0.50	10.31
	**hemangioma**	75	0.39	1.33
	**venous malformations**	72	0.37	2.78
	**telangiectases**	64	0.33	6.25
	**hemangioma infancy**	57	0.30	12.28
	**syringoma**	50	0.26	8.00
	**angiokeratomas**	48	0.25	12.50
**Benign dermal tumors cysts sinuses**	**venous lake**	46	0.24	2.17
	**granulation tissue**	45	0.23	0.00
	**fordyce spots**	32	0.17	0.00
	**becker nevus**	29	0.15	20.69
	**cherry angioma**	28	0.15	50.00
	**lymphangioma**	24	0.12	4.17
	**cylindroma**	15	0.08	26.67
	**nevus anemicus**	14	0.07	14.29
	**spider angioma**	14	0.07	0.00
	**fibroma**	11	0.06	9.09
	**hidrocystoma**	10	0.05	0.00
	**leiomyoma**	10	0.05	10.00
	**periungual fibroma**	9	0.05	0.00

	vascular anomaly	8	0.04	0.00
	trichoepithelioma	6	0.03	0.00
	pilomatricoma	5	0.03	0.00
	trichofolliculoma	5	0.03	0.00
	arteriovenous malformation	4	0.02	0.00
	connective tissue nevus	3	0.02	33.33
	spiradenoma	3	0.02	0.00
	endometriosis	2	0.01	0.00
	glomus tumor	2	0.01	100.00
	cutis marmorata telangiectatica congenita	1	0.01	0.00
	pilonidal sinus	1	0.01	0.00
	scrotal cyst	1	0.01	0.00
	trichoadenoma	1	0.01	0.00
Cutaneous lymphoma and lymphoid infiltrates	cutaneous t cell lymphoma	85	0.44	11.76
	lymphoma b cell	4	0.02	0.00
	lymphomatoid papulosis	2	0.01	0.00
Epidermal tumors hamartomas milia and growths benign	seborrheic keratoses ruff	147	0.76	0.00
	epidermal nevus	142	0.74	3.52
	seborrheic keratoses smooth	122	0.63	0.00
	seborrheic keratosis irritated	110	0.57	0.91
	porokeratosis	79	0.41	0.00
	prurigo nodularis	48	0.25	12.50
	stucco keratoses	48	0.25	0.00
	corns	40	0.21	5.00
	milia	34	0.18	11.76
	dermatosis papulosa nigra	8	0.04	12.50
	acquired digital fibrokeratoma	7	0.04	42.86
	stucco keratosis	7	0.04	0.00
	leser trelat sign	2	0.01	0.00
	seborrheic keratosis	2	0.01	0.00
Epidermal tumors pre malignant and malignant	keratoacanthoma	147	0.76	2.72
	basal cell carcinoma lesion	128	0.66	2.34

**bowens disease**	97	0.50	3.09
**basal cell carcinoma**	79	0.41	3.80
**basal cell carcinoma nose**	65	0.34	6.15
**actinic cheilitis**	61	0.32	0.00
**actinic keratosis face**	60	0.31	0.00
**actinic keratosis fu**	59	0.31	0.00
**basal cell carcinoma superficial**	59	0.31	0.00
**actinic keratosis lesion**	50	0.26	0.00
**basal cell carcinoma face**	44	0.23	4.55
**basal cell carcinoma lid**	38	0.20	0.00
**actinic keratosis hand**	37	0.19	0.00
**basal cell carcinoma ear**	37	0.19	0.00
**squamous cell carcinoma ear**	37	0.19	0.00
**basal cell carcinoma lip**	32	0.17	3.12
**squamous cell carcinoma lesion**	29	0.15	3.45
**cutaneous horn**	26	0.13	3.85
**actinic keratosis horn**	25	0.13	0.00
**squamous cell carcinoma lip**	25	0.13	4.00
**basal cell nevus syndrome**	21	0.11	14.29
**actinic keratosis ear**	16	0.08	0.00
**arsenical keratoses**	16	0.08	6.25
**squamous cell carcinoma**	15	0.08	6.67
**actinic keratosis**	14	0.07	0.00
**actinic keratosis nose**	14	0.07	0.00
**basal cell carcinoma scalp**	14	0.07	14.29
**pagets disease**	14	0.07	7.14
**leukoplakia**	13	0.07	0.00
**basal cell carcinoma trunk ext**	12	0.06	16.67
**squamous cell carcinoma hand arm**	12	0.06	0.00
**actinic keratosis pigmented**	8	0.04	0.00
**extramammary paget disease**	8	0.04	12.50
**squamous cell carcinoma face**	7	0.04	0.00
**squamous cell carcinoma nose**	6	0.03	0.00

	**basal cell carcinoma aldara**	5	0.03	0.00
	**erythroplasia queyrat**	5	0.03	0.00
	**verrucous carcinoma**	5	0.03	0.00
	**keratosis solar**	2	0.01	0.00
	**basal cell carcinoma sclerosing**	1	0.01	0.00
	**superficial basal cell carcinoma**	1	0.01	0.00
**Genodermatoses and supernumerary growths**	**neurofibromatosis**	88	0.46	6.82
	**dariers disease**	64	0.33	3.12
	**epidermolysis bullosa**	58	0.30	15.52
	**tuberous sclerosis**	56	0.29	5.36
	**ichthyosis sex linked**	44	0.23	0.00
	**ichthyosis other forms**	41	0.21	19.51
	**ichthyosis dominant**	35	0.18	0.00
	**hailey hailey disease**	30	0.16	0.00
	**supernumerary growths**	16	0.08	18.75
	**birt hogg dube**	14	0.07	0.00
	**lipoid proteinosis urbach wiethe disease**	11	0.06	9.09
	**albinism**	9	0.05	0.00
	**congenital anomalies**	9	0.05	0.00
	**cowden disease**	8	0.04	0.00
	**pseudoxanthoma elasticum**	8	0.04	12.50
	**acrodermatitis enteropathica**	6	0.03	0.00
	**muir torre syndrome**	5	0.03	0.00
	**erythrokeratodermia variabilis**	4	0.02	0.00
	**congenital erythropoietic porphyria**	3	0.02	0.00
	**ichthyosis**	2	0.01	0.00
	**vascular tumors and malformations**	1	0.01	0.00
**Inflammatory**	**diaper dermatitis**	266	1.38	10.53
	**lichen planus**	261	1.35	5.36
	**molluscum contagiosum**	241	1.25	25.73
	**non scarring alopecia**	213	1.10	8.45
	**lichen sclerosus skin**	205	1.06	10.73

**erythema multiforme and fixed drug eruptions**	199	1.03	8.04
**photodermatoses**	160	0.83	4.38
**onychomycosis**	158	0.82	0.63
**genital warts**	153	0.79	14.38
**herpes type recurrent**	148	0.77	8.78
**vasculitis**	146	0.76	1.37
**psoriasis**	144	0.75	2.78
**lichen simplex**	142	0.74	14.08
**eczema fingertips**	141	0.73	0.00
**lupus chronic cutaneous**	141	0.73	8.51
**rosacea**	140	0.73	3.57
**tinea ringworm body**	137	0.71	11.68
**atopic dermatitis adult phase**	136	0.71	3.68
**scabies**	135	0.70	9.63
**psoriasis chronic plaque**	134	0.69	0.75
**granuloma annulare**	133	0.69	6.77
**herpes zoster**	133	0.69	15.04
**herpes type primary**	132	0.68	12.12
**seborrheic dermatitis**	132	0.68	9.85
**allergic contact dermatitis**	131	0.68	6.11
**pityriasis rosea**	129	0.67	2.33
**sun damaged skin**	129	0.67	1.55
**perioral dermatitis**	125	0.65	4.00
**tinea ringworm groin**	123	0.64	40.65
**pigmentation diseases**	122	0.63	6.56
**drug eruption**	119	0.62	1.68
**tinea ringworm versicolor**	118	0.61	8.47
**eczema nummular**	117	0.61	2.56
**psoriasis palms soles**	116	0.60	3.45
**warts common**	116	0.60	9.48
**acne cystic**	114	0.59	3.51
**eczema hand**	114	0.59	0.00

**psoriasis guttate**	113	0.59	7.08
**eczema subacute**	111	0.58	8.11
**stasis dermatitis and ulcers**	111	0.58	0.00
**bullous pemphigoid**	110	0.57	7.27
**warts plantar**	109	0.57	6.42
**atopic dermatitis childhood phase**	103	0.53	6.80
**hidradenitis**	103	0.53	15.53
**eczema foot**	102	0.53	0.00
**psoriasis scalp**	102	0.53	3.92
**dermatitis herpetiformis**	100	0.52	9.00
**dyshidrotic eczema**	100	0.52	0.00
**herpes cutaneous**	100	0.52	10.00
**diseases of the ear**	97	0.50	0.00
**morphea**	96	0.50	6.25
**urticaria acute**	95	0.49	4.21
**biting fleas**	89	0.46	15.73
**scarring alopecia**	88	0.46	13.64
**necrobiosis lipoidica**	85	0.44	1.18
**rhus dermatitis**	85	0.44	0.00
**atopic dermatitis infant phase**	84	0.44	14.29
**depositional diseases calcinosis cutis amyloid mucinosis gout and others**	84	0.44	4.76
**keratosis pilaris and related follicular disorders**	84	0.44	2.38
**paronychia**	84	0.44	0.00
**xanthomas**	83	0.43	13.25
**porphyria**	82	0.43	8.54
**scarring**	81	0.42	8.64
**tinea ringworm foot webs**	80	0.41	3.75
**acne pustular**	76	0.39	5.26
**impetigo**	76	0.39	5.26
**rosacea nose**	74	0.38	1.35
**virus exanthem**	74	0.38	4.05
**cheilitis**	72	0.37	0.00

**pigmented purpuric dermatosis**	72	0.37	1.39
**pseudomonas**	72	0.37	4.17
**tinea ringworm laboratory**	72	0.37	4.17
**acne open comedo**	69	0.36	2.90
**intertrigo**	68	0.35	29.41
**pustular skin disease non infectious**	68	0.35	5.88
**tinea ringworm scalp**	68	0.35	4.41
**pityriasis rubra pilaris**	67	0.35	7.46
**psoriasis hand**	67	0.35	0.00
**tinea ringworm foot dorsum**	65	0.34	0.00
**tinea ringworm hand dorsum**	65	0.34	3.08
**varicella**	65	0.34	32.31
**cutaneous manifestations of systemic disease**	64	0.33	14.06
**onycholysis**	64	0.33	0.00
**herpes simplex**	61	0.32	11.48
**neurotic excoriations**	61	0.32	14.75
**warts**	61	0.32	3.28
**eczema herpeticum**	60	0.31	10.00
**patch testing**	60	0.31	10.00
**grovers disease**	59	0.31	10.17
**pemphigus**	59	0.31	0.00
**tick bite**	59	0.31	6.78
**tinea ringworm face**	58	0.30	1.72
**acne keloidalis**	57	0.30	42.11
**warts digitate**	57	0.30	0.00
**dermatomyositis**	56	0.29	5.36
**dry skin eczema**	56	0.29	0.00
**pediculosis lids**	56	0.29	5.36
**tinea ringworm primary lesion**	56	0.29	3.57
**lupus acute**	55	0.29	3.64
**warts flat**	54	0.28	1.85
**enterovirus**	53	0.27	15.09

**pityriasis lichenoides and pleva**	53	0.27	18.87
**tinea ringworm incognito**	53	0.27	11.32
**habit tic deformity**	51	0.26	0.00
**keratolysis exfoliativa**	51	0.26	0.00
**eczema chronic**	50	0.26	0.00
**lichen planus oral**	49	0.25	2.04
**tinea ringworm foot plantar**	49	0.25	0.00
**tinea ringworm palm**	48	0.25	0.00
**acne closed comedo**	47	0.24	0.00
**nematode infection**	47	0.24	10.64
**gyrate erythemas**	46	0.24	6.52
**panniculitis lipodystrophy and diseases of subcutis**	45	0.23	11.11
**psoriasis nails**	44	0.23	0.00
**scleroderma**	44	0.23	4.55
**crest syndrome**	43	0.22	2.33
**pitted keratolysis**	42	0.22	0.00
**dermographia**	41	0.21	2.44
**lichen**	41	0.21	0.00
**pernio and mimics**	41	0.21	4.88
**folliculitis**	39	0.20	20.51
**scarlet fever**	39	0.20	5.13
**lichen planus drug**	38	0.20	2.63
**psoriasis inversus**	38	0.20	18.42
**erythema infectiosum**	37	0.19	2.70
**warts cryotherapy**	37	0.19	2.70
**erosio interdigitalis blastomycetica**	36	0.19	0.00
**fungal infections dermal and subcut**	36	0.19	8.33
**malformations of extremities**	36	0.19	16.67
**erythroderma**	35	0.18	0.00
**spider bite**	35	0.18	14.29
**median nail dystrophy**	33	0.17	0.00
**warts periungual**	33	0.17	0.00

**wet to dry foot syndrome**	33	0.17	0.00
**ridging beading**	32	0.17	0.00
**staphylococcal folliculitis**	32	0.17	37.50
**sarcoidosis**	31	0.16	3.23
**eczema leg**	30	0.16	0.00
**hand foot mouth**	30	0.16	33.33
**acne excoriee**	29	0.15	17.24
**herpes buttocks**	29	0.15	10.34
**kawasaki syndrome**	29	0.15	17.24
**psoriasis penis**	29	0.15	0.00
**beaus lines**	28	0.15	0.00
**diabetic bullae**	28	0.15	3.57
**furuncle**	27	0.14	29.63
**myxoedema pretibial**	27	0.14	7.41
**pemphigus foliaceous**	27	0.14	0.00
**angioedema**	26	0.13	7.69
**atopic dermatitis feet**	26	0.13	0.00
**pseudomonas folliculitis**	26	0.13	26.92
**terry nails**	26	0.13	0.00
**eczema**	25	0.13	4.00
**hair disorderstrichorrhexis nodosa etc**	25	0.13	32.00
**henoch schonlein purpura**	25	0.13	12.00
**histiocytic disorders and proliferations**	25	0.13	16.00
**eczema arms**	24	0.12	0.00
**ingrown nail**	24	0.12	0.00
**erythema ab igne**	23	0.12	0.00
**eczema trunk generalized**	22	0.11	4.55
**id reaction**	22	0.11	0.00
**psoriasis anus**	22	0.11	22.73
**dermatitis swimming**	21	0.11	0.00
**reiters disease**	21	0.11	23.81
**splinter hemorrhage**	21	0.11	0.00

**syphilis**	21	0.11	19.05
**tinea ringworm beard**	21	0.11	14.29
**interstitial granulomatous dermatitis**	20	0.10	0.00
**koilonychia**	20	0.10	0.00
**pincer nails**	20	0.10	0.00
**distal splitting hang nail**	19	0.10	0.00
**eczema lids**	19	0.10	0.00
**hypereosinophilic syndrome**	19	0.10	0.00
**leprosy**	19	0.10	10.53
**planar warts**	19	0.10	0.00
**psoriasis treatment**	19	0.10	0.00
**erythrasma**	18	0.09	50.00
**gianotti crosti**	18	0.09	0.00
**pigmented bands**	18	0.09	11.11
**stasis dermatitis**	18	0.09	0.00
**sycosis barbae**	18	0.09	0.00
**acne infantile**	17	0.09	0.00
**atypical mycobacterium**	17	0.09	52.94
**eczema acute**	17	0.09	0.00
**eczema areola**	17	0.09	0.00
**atrophic skin disease striae and elastic fiber disorders**	16	0.08	18.75
**lichen nitidus**	16	0.08	6.25
**rheumatoid nodule**	16	0.08	37.50
**candidiasis mouth**	15	0.08	0.00
**cellulitis**	15	0.08	13.33
**clubbing**	15	0.08	0.00
**solar damage favre racouchot**	15	0.08	0.00
**urticaria vasculitis**	15	0.08	0.00
**cholinergic urticaria**	14	0.07	0.00
**twenty nail dystrophy**	14	0.07	0.00
**acquired autoimmune bullous diseaseherpes gestationis**	13	0.07	38.46
**fissure**	13	0.07	0.00

**lichen planus hypertrophic**	13	0.07	7.69
**lichen planus vaginal**	13	0.07	7.69
**lupus subacute**	13	0.07	7.69
**maceration**	13	0.07	0.00
**metal dermatitis**	13	0.07	15.38
**perforating disorders**	13	0.07	0.00
**psoriasis light sensative**	13	0.07	0.00
**warts mouth**	13	0.07	0.00
**acne scar**	12	0.06	0.00
**atopic dermatitis hyperlinear creases**	12	0.06	0.00
**cercaria dermatitis**	12	0.06	0.00
**metabolic and nutritional diseases**	12	0.06	8.33
**shoe allergy**	12	0.06	0.00
**warts immunocompromised**	12	0.06	0.00
**ant bite**	11	0.06	36.36
**caterpillar dermatitis**	11	0.06	18.18
**erysipelas**	11	0.06	18.18
**gonorrhea**	11	0.06	9.09
**livedo reticularis**	11	0.06	0.00
**mastocytosis**	11	0.06	9.09
**myiasis**	11	0.06	36.36
**onychogryphosis**	11	0.06	0.00
**contact dermatitis**	10	0.05	10.00
**eczema vulva**	10	0.05	0.00
**factitial dermatitis**	10	0.05	0.00
**toxic epidermal necrolysis and stevens johnson**	10	0.05	30.00
**acanthosis nigricans and carp**	9	0.05	0.00
**atopic dermatitis**	9	0.05	0.00
**axillary granular parakeratosis**	9	0.05	0.00
**eczema ears**	9	0.05	0.00
**eczema face**	9	0.05	0.00
**irritant contact dermatitis**	9	0.05	0.00

**lichen planus penis**	9	0.05	0.00
**localized pemphigoid**	9	0.05	33.33
**pityrosporum folliculitis**	9	0.05	0.00
**poikiloderma civatte**	9	0.05	11.11
**tinea versicolor**	9	0.05	0.00
**trichomycosis**	9	0.05	33.33
**cat scratch disease**	8	0.04	37.50
**cimicosis**	8	0.04	12.50
**cryoglobulinemia**	8	0.04	0.00
**eczema impetiginized**	8	0.04	0.00
**heels dry cracked**	8	0.04	12.50
**hidroa vacciniforme**	8	0.04	25.00
**leukonychia**	8	0.04	0.00
**lymphedema**	8	0.04	12.50
**meningococcemia**	8	0.04	0.00
**pachyonychia congenita**	8	0.04	0.00
**papulose bowenoide**	8	0.04	0.00
**sunburn**	8	0.04	37.50
**dry nails**	7	0.04	0.00
**herpes immunocompromised**	7	0.04	0.00
**hyperhidrosis**	7	0.04	0.00
**lichen planus palm soles**	7	0.04	0.00
**radiodermatitis**	7	0.04	28.57
**acne cystica**	6	0.03	0.00
**atrophy blanche**	6	0.03	16.67
**chancroid**	6	0.03	0.00
**cosmetic fragrance allergy**	6	0.03	0.00
**degos disease**	6	0.03	50.00
**erythromelalgia**	6	0.03	0.00
**nummular eczema**	6	0.03	16.67
**psoriasis digits**	6	0.03	16.67
**psoriasis infants**	6	0.03	0.00
**roseola infantum**	6	0.03	0.00

**scleromyxedema**	6	0.03	0.00
**tinea incognito**	6	0.03	0.00
**urticaria**	6	0.03	0.00
**acute eczema**	5	0.03	0.00
**ashy dermatosis**	5	0.03	0.00
**diseases of bone**	5	0.03	0.00
**erysipeloid**	5	0.03	0.00
**orf**	5	0.03	40.00
**parasitosis psychogenic**	5	0.03	20.00
**phlebitis superficial**	5	0.03	0.00
**raynauds disease**	5	0.03	0.00
**scalp psoriasis**	5	0.03	0.00
**steroid acne**	5	0.03	0.00
**tinea ringworm border**	5	0.03	0.00
**balanitis bacterial**	4	0.02	0.00
**blue nails minocycline**	4	0.02	0.00
**candidiasis chronic mucocutaneos**	4	0.02	25.00
**desquamation**	4	0.02	0.00
**infected eczema**	4	0.02	0.00
**lichen planopilaris**	4	0.02	0.00
**lichen striatus**	4	0.02	0.00
**natural rubber latex allergy**	4	0.02	0.00
**notalgia paraesthetica**	4	0.02	0.00
**psoriasis hiv**	4	0.02	25.00
**relapsing polychondritis**	4	0.02	25.00
**rocky mountain spotted fever**	4	0.02	25.00
**rosacea steroid**	4	0.02	0.00
**small pox**	4	0.02	25.00
**staphylococcal scalded skin syndrome**	4	0.02	25.00
**streptococci anal penis**	4	0.02	25.00
**wart**	4	0.02	0.00
**acne vulgaris**	3	0.02	33.33

**alopecia**	3	0.02	0.00
**bacterial infections**	3	0.02	33.33
**epidermodysplasia verruciformis**	3	0.02	0.00
**fibromatosis**	3	0.02	0.00
**hematogenous allergic contact dermatitis**	3	0.02	33.33
**pemphigus foliaceus**	3	0.02	0.00
**purpura fulminans**	3	0.02	0.00
**tinea pedis**	3	0.02	0.00
**tinea ringworm nigra**	3	0.02	0.00
**ulcer**	3	0.02	0.00
**urticarial vasculitis**	3	0.02	0.00
**warts oral**	3	0.02	0.00
**acne rosacea**	2	0.01	0.00
**allergic hand eczema**	2	0.01	0.00
**ecthyma simplex**	2	0.01	0.00
**eczema anal**	2	0.01	100.00
**eczema scrotum**	2	0.01	0.00
**erythema elevatum diutinum**	2	0.01	0.00
**granuloma**	2	0.01	0.00
**granuloma multiforme**	2	0.01	0.00
**herpes gestationis**	2	0.01	50.00
**herpes simplex genitalis**	2	0.01	0.00
**herpes simplex oralis**	2	0.01	0.00
**insect bite**	2	0.01	50.00
**lupus vulgaris**	2	0.01	0.00
**measles**	2	0.01	0.00
**nail diseases**	2	0.01	0.00
**panarteritis nodosa kussmaul maier**	2	0.01	0.00
**pomade acne**	2	0.01	0.00
**psoriatic erythroderma**	2	0.01	0.00
**behcets disease**	1	0.01	0.00

	**candidiasis**	1	0.01	100.00
	**chronic bullous disease of childhood**	1	0.01	0.00
	**condyloma acuminata**	1	0.01	0.00
	**contact dermatitis leg ulcers**	1	0.01	0.00
	**disseminated lokalized scleroderma**	1	0.01	0.00
	**eosinophilic ulcer of the tongue**	1	0.01	0.00
	**erythema craquele**	1	0.01	0.00
	**gingival disease**	1	0.01	100.00
	**guttate psoriasis**	1	0.01	0.00
	**malassezia folliculitis**	1	0.01	100.00
	**miliaria**	1	0.01	0.00
	**nail dystrophy**	1	0.01	0.00
	**nail psoriasis**	1	0.01	0.00
	**nail shedding**	1	0.01	0.00
	**nodulo cystic acne vulgaris**	1	0.01	0.00
	**primary herpes simplex virus**	1	0.01	0.00
	**psoriasis inversa**	1	0.01	0.00
	**psoriasis nail psoriasis**	1	0.01	0.00
	**psoriasis vulgaris guttate type**	1	0.01	0.00
	**purpura**	1	0.01	0.00
	**reticular erythematous mucinosis**	1	0.01	0.00
	**solar elastosis**	1	0.01	0.00
	**solar purpura**	1	0.01	0.00
	**stasis edema**	1	0.01	0.00
	**stasis ulcer**	1	0.01	0.00
	**thermal burn**	1	0.01	0.00
	**tinea facei**	1	0.01	0.00
	**torus palantine**	1	0.01	0.00
	**verrucae planae juveniles**	1	0.01	0.00
**Malignant dermal tumor**	**intermediate or malignant vascular tumors**	37	0.19	5.41
	**systemic carcinomas**	28	0.15	10.71

	merkel cell carcinoma	4	0.02	0.00
	sarcoma	4	0.02	25.00
	dermatofibrosarcoma protuberans	2	0.01	0.00
Pigmented lesions benign	atypical nevi	118	0.61	1.69
	melanocytic nevi	97	0.50	0.00
	solar lentigo	80	0.41	1.25
	congenital nevus	53	0.27	5.66
	nevus spilus	34	0.18	2.94
	halo nevus	30	0.16	10.00
	melanoma mimic	26	0.13	0.00
	black heel	17	0.09	0.00
	blue nevus	14	0.07	0.00
	melanotic macule	10	0.05	0.00
	blue nevus ota	9	0.05	11.11
	mongolian spot	8	0.04	25.00
	spitz nevus	7	0.04	0.00
	café au lait spot	3	0.02	33.33
	nevus repigmented	3	0.02	0.00
	lentigo simplex	1	0.01	0.00
Pigmented lesions malignant	malignant melanoma	130	0.67	1.54
	lentigo maligna	26	0.13	0.00
	nail melanoma	5	0.03	0.00
	lentigo maligna melanoma	3	0.02	0.00
	Unknown	359	1.86	11.42
	All	19289	100.00	6.86

Supplementary Table 7: Frequency Table of Diagnoses in the DermNet NZ Repository

Frequency table of diagnoses of evaluated skin lesion images in the DermNet NZ repository. False Positive Rates are not reported because this dataset has been used for training and is not part of testing cohort.
All, Number of all evaluated skin lesion images in the DermNet NZ repository; n, Number of occurrences of diagnosis; Unknown, Diagnosis unknown or unmapped; %, percentage of diagnosis in relation to all evaluated skin lesion images in the DermNet NZ repository.

Category	Diagnosis	n	%
Benign dermal tumors cysts sinuses	angiokeratomas	59	0.49
	telangiectases	37	0.31
	becker nevus	24	0.20
	skin tag	24	0.20
	myxoid cyst	21	0.17
	dermatofibroma	19	0.16
	spider nevus	14	0.12
	cutis marmorata telangiectatica congenita	13	0.11
	pyogenic granuloma	13	0.11
	cherry angioma	12	0.10
	venous lake	9	0.07
	nevus anemicus	7	0.06
	periungual fibroma	6	0.05
	port wine stain	6	0.05
	steatocystoma multiplex	6	0.05
	connective tissue nevus	5	0.04
	eccrine poroma	5	0.04
	pilomatricoma	5	0.04
	angioma serpiginosum	4	0.03
	glomus tumor	4	0.03
	hidrocystoma	4	0.03
	lymphangioma	4	0.03
	syringoma	4	0.03

	**trichoepithelioma**	4	0.03
	**dermoid cyst**	3	0.02
	**eruptive vellus hair cysts**	3	0.02
	**sebaceous hyperplasia**	3	0.02
	**leiomyoma**	2	0.02
	**lymphatic malformations**	2	0.02
	**pilar cyst**	2	0.02
	**pilonidal sinus**	2	0.02
	**klippel trenaunay syndrome**	1	0.01
**Cutaneous lymphoma and lymphoid infiltrates**	**cutaneous t cell lymphoma**	49	0.41
	**lymphoma b cell**	25	0.21
	**erythema arciforme et palpabile migrans**	16	0.13
	**lymphomatoid papulosis**	9	0.07
**Epidermal tumors hamartomas milia and growths benign**	**prurigo nodularis**	46	0.38
	**seborrheic keratosis**	37	0.31
	**milia**	21	0.17
	**epidermal nevus**	16	0.13
	**corns**	13	0.11
	**porokeratosis**	9	0.07
	**dermatosis papulosa nigra**	7	0.06
	**callus**	5	0.04
	**clear cell acanthoma**	4	0.03
	**stucco keratosis**	1	0.01
**Epidermal tumors pre malignant and malignant**	**basal cell carcinoma**	234	1.94
	**actinic keratosis**	173	1.44
	**squamous cell carcinoma**	82	0.68
	**bowens disease**	55	0.46
	**keratoacanthoma**	52	0.43
	**extramammary paget disease**	29	0.24
	**cutaneous horn**	21	0.17
	**leukoplakia**	13	0.11
	**pagets disease**	9	0.07

	squamous cell carcinoma face	7	0.06
	actinic cheilitis	5	0.04
	superficial basal cell carcinoma	4	0.03
	basal cell nevus syndrome	3	0.02
	arsenical keratoses	1	0.01
	pigmented basal cell carcinoma	1	0.01
	solar damage	1	0.01
Genodermatoses and supernumerary growths	vascular tumors and malformations	53	0.44
	ichthyosis	37	0.31
	tuberous sclerosis	34	0.28
	neurofibromatosis	24	0.20
	ehlers danlos syndrome	20	0.17
	pseudoxanthoma elasticum	16	0.13
	dariers disease	13	0.11
	cowden disease	11	0.09
	acrodermatitis enteropathica	10	0.08
	birt hogg dube	9	0.07
	xeroderma pigmentosum	9	0.07
	congenital erythropoietic porphyria	8	0.07
	epidermolysis bullosa	8	0.07
	epidermolytic hyperkeratosis	8	0.07
	hailey hailey disease	7	0.06
	rothmund thomson syndrome	6	0.05
	x linked ichthyosis	6	0.05
	incontinentia pigmenti	5	0.04
	ichthyosis vulgaris	4	0.03
	knuckle pads	3	0.02
	leopard syndrome	3	0.02
	muir torre syndrome	3	0.02
	collodion baby	2	0.02
	acrokeratoelastoidosis	1	0.01
Inflammatory	acne vulgaris	440	3.65
	atopic dermatitis	220	1.83

**psoriasis**	171	1.42
**lichen planus**	116	0.96
**toxic epidermal necrolysis and stevens johnson**	104	0.86
**nummular eczema**	101	0.84
**dermatomyositis**	89	0.74
**scabies**	88	0.73
**hidradenitis**	87	0.72
**molluscum contagiosum**	85	0.71
**syphilis**	84	0.70
**hand foot mouth**	74	0.61
**ulcer**	72	0.60
**urticaria**	63	0.52
**lupus erythematosus systemic**	61	0.51
**seborrheic dermatitis**	61	0.51
**bullous pemphigoid**	56	0.46
**wart**	56	0.46
**pityriasis rosea**	55	0.46
**scarring**	54	0.45
**discoid lupus erythematosus**	53	0.44
**granuloma annulare**	52	0.43
**sarcoidosis**	52	0.43
**xanthomas**	52	0.43
**herpes simplex labialis**	51	0.42
**pemphigus vulgaris**	49	0.41
**gianotti crosti**	48	0.40
**lupus erythematosus subacute cutaneous lupus erythematosus**	45	0.37
**pityriasis versicolor**	45	0.37
**herpes gestationis**	44	0.37
**orf**	43	0.36
**pityriasis lichenoides and pleva**	42	0.35
**allergic contact dermatitis**	41	0.34
**atypical mycobacterium**	41	0.34

**nail psoriasis**	41	0.34
**psoriasis palmoplantaris**	41	0.34
**varicella**	41	0.34
**herpes zoster**	40	0.33
**lichen sclerosus skin**	40	0.33
**rosacea**	40	0.33
**behcets disease**	39	0.32
**impetigo**	38	0.32
**photodermatoses**	38	0.32
**insect bite**	37	0.31
**drug eruption**	35	0.29
**diaper dermatitis**	34	0.28
**enterovirus**	34	0.28
**psoriasis nails**	33	0.27
**measles**	32	0.27
**vasculitis**	32	0.27
**virus exanthem**	32	0.27
**lichen simplex**	31	0.26
**candidiasis**	30	0.25
**keratolysis exfoliativa**	28	0.23
**alopecia**	27	0.22
**erythema multiforme and fixed drug eruptions**	27	0.22
**urticarial vasculitis**	26	0.22
**grovers disease**	25	0.21
**tinea capitis**	25	0.21
**erythroderma**	24	0.20
**morphea**	24	0.20
**staphylococcal scalded skin syndrome**	24	0.20
**acanthosis nigricans and carp**	23	0.19
**tinea pedis**	23	0.19
**atopic eczema**	21	0.17
**diabetic ulcer**	21	0.17

**pitted keratolysis**	21	0.17
**subacute cutaneous lupus erythematosus**	20	0.17
**angioedema**	19	0.16
**contact dermatitis**	19	0.16
**eczema herpeticum**	19	0.16
**nodulo cystic acne vulgaris**	19	0.16
**pincer nails**	19	0.16
**rosacea steroid**	19	0.16
**erythrasma**	18	0.15
**herpes simplex**	18	0.15
**mastocytosis**	18	0.15
**panniculitis lipodystrophy and diseases of subcutis**	18	0.15
**psoriasis chronic plaque**	18	0.15
**folliculitis**	17	0.14
**myxoedema pretibial**	17	0.14
**perioral dermatitis**	16	0.13
**urticaria factitia**	16	0.13
**scalp psoriasis**	15	0.12
**tinea corporis**	15	0.12
**tuberculosis**	15	0.12
**erythema craquele**	14	0.12
**keratosis pilaris and related follicular disorders**	13	0.11
**onychomycosis**	13	0.11
**axillary granular parakeratosis**	12	0.10
**chronic venous insufficiency grade ii**	12	0.10
**lupus erythematosus chronicus discoides**	12	0.10
**relapsing polychondritis**	12	0.10
**cellulitis**	11	0.09
**cholinergic urticaria**	11	0.09
**eczema areola**	11	0.09
**leprosy**	11	0.09

**roseola infantum**	11	0.09
**steroid acne**	11	0.09
**ecthyma simplex**	10	0.08
**erythema infectiosum**	10	0.08
**sunburn**	10	0.08
**acne scar**	9	0.07
**fourniers gangrene**	9	0.07
**pachyonychia congenita**	9	0.07
**paronychia**	9	0.07
**porphyria**	9	0.07
**rhinophyma**	9	0.07
**tinea cruris**	9	0.07
**atrophy blanche**	8	0.07
**epidermolysis bullosa acquisita**	8	0.07
**cryoglobulinemia**	7	0.06
**dermatitis swimming**	7	0.06
**eczema**	7	0.06
**erysipelas**	7	0.06
**genital warts**	7	0.06
**pityriasis rubra pilaris**	7	0.06
**pseudofolliculitis barbae**	7	0.06
**acne infantile**	6	0.05
**clubbing**	6	0.05
**dermatitis herpetiformis**	6	0.05
**histiocytic disorders and proliferations**	6	0.05
**lichen planus oral**	6	0.05
**lichen striatus**	6	0.05
**pemphigus foliaceus**	6	0.05
**poikiloderma civatte**	6	0.05
**scleromyxedema**	6	0.05
**tinea manuum**	6	0.05
**candidiasis mouth**	5	0.04

**dry skin eczema**	5	0.04
**eczema nummular**	5	0.04
**hidroa vacciniforme**	5	0.04
**irritant contact dermatitis**	5	0.04
**notalgia paraesthetica**	5	0.04
**pyoderma faciale**	5	0.04
**scarlet fever**	5	0.04
**tinea barbae**	5	0.04
**tinea faciei**	5	0.04
**wound infection**	5	0.04
**acne keloidalis**	4	0.03
**cicatricial pemphigoid**	4	0.03
**eosinophilic pustular folliculitis**	4	0.03
**guttate psoriasis**	4	0.03
**henoch schonlein purpura**	4	0.03
**infected eczema**	4	0.03
**intertrigo**	4	0.03
**lichen planopilaris**	4	0.03
**miliaria**	4	0.03
**necrobiosis lipoidica**	4	0.03
**sjogrens syndrome**	4	0.03
**spider bite**	4	0.03
**tinea incognito**	4	0.03
**tinea nigra**	4	0.03
**acanthoma fissuratum**	3	0.02
**acne excoriee**	3	0.02
**acne fulminans**	3	0.02
**acute eczema**	3	0.02
**beaus lines**	3	0.02
**chancroid**	3	0.02
**diabetic bullae**	3	0.02
**erysipeloid**	3	0.02
**erythema ab igne**	3	0.02

**erythema elevatum diutinum**	3	0.02
**erythromelalgia**	3	0.02
**hypereosinophilic syndrome**	3	0.02
**interstitial granulomatous dermatitis**	3	0.02
**lichen**	3	0.02
**livedo reticularis**	3	0.02
**lymphedema**	3	0.02
**reticular erythematous mucinosis**	3	0.02
**sycosis barbae**	3	0.02
**terry nails**	3	0.02
**tinea ringworm border**	3	0.02
**cutis marmorata**	2	0.02
**degos disease**	2	0.02
**epidermodysplasia verruciformis**	2	0.02
**geographic tongue**	2	0.02
**ischemia**	2	0.02
**lichen nitidus**	2	0.02
**malassezia folliculitis**	2	0.02
**onychogryphosis**	2	0.02
**onycholysis**	2	0.02
**patch testing**	2	0.02
**radiodermatitis**	2	0.02
**rheumatoid nodule**	2	0.02
**solar elastosis**	2	0.02
**thermal burn**	2	0.02
**thrombangiitis obliterans**	2	0.02
**acne conglobata**	1	0.01
**erosio interdigitalis blastomycetica**	1	0.01
**half and half nail**	1	0.01
**ingrown nail**	1	0.01
**koilonychia**	1	0.01
**lupus vulgaris**	1	0.01
**pemphigus**	1	0.01

	psoriatic erythroderma	1	0.01
	purpura	1	0.01
	stomatitis	1	0.01
	wells syndrome	1	0.01
	sarcoma	21	0.17
Malignant dermal tumor	merkel cell carcinoma	5	0.04
	dermatofibrosarcoma protuberans	2	0.02
	solar lentigo	65	0.54
	melanocytic nevi	49	0.41
	nevus cell nevus	31	0.26
	café au lait spot	19	0.16
	atypical nevi	18	0.15
	blue nevus	12	0.10
Pigmented lesions benign	spitz nevus	8	0.07
	congenital nevus	7	0.06
	halo nevus	7	0.06
	nail nevus	4	0.03
	labial melanotic macule	3	0.02
	lentigo simplex	3	0.02
	ephelides	1	0.01
	malignant melanoma	121	1.00
	amelanotic malignant melanoma amm	35	0.29
	superficial spreading melanoma ssm	27	0.22
	metastatic melanoma	23	0.19
Pigmented lesions malignant	nodular melanoma nm	19	0.16
	lentigo maligna	17	0.14
	acrolentiginous melanoma alm	10	0.08
	lentigo maligna melanoma	5	0.04
	nail melanoma	1	0.01
	Unknown	5216	43.30
	All	12045	100.00

Supplementary Table 8: Frequency Table and False Positive Rates of Diagnoses in the Fitzpatrick 17k Dataset

Frequency table of diagnoses and False Positive Rates of evaluated skin lesion images in the Fitzpatrick 17k dataset.
All, Number of all evaluated skin lesion images in the Fitzpatrick 17k dataset; FPR, False positive rate (%) of the diagnosis in the Fitzpatrick 17k dataset; n, Number of occurrences of diagnosis; Unknown, Diagnosis unknown or unmapped; %, percentage of diagnosis in relation to all evaluated skin lesion images in the Fitzpatrick 17k dataset.

Category	Diagnosis	n	%	FPR
Benign dermal tumors cysts sinuses	syringoma	127	0.77	4.72
	telangiectases	125	0.76	10.40
	pyogenic granuloma	113	0.68	5.31
	fordyce spots	100	0.61	10.00
	lymphangioma	100	0.61	6.00
	pilar cyst	93	0.56	15.05
	dermatofibroma	79	0.48	15.19
	mucous cyst	78	0.47	15.38
	granuloma pyogenic	75	0.45	5.33
	becker nevus	63	0.38	12.70
	port wine stain	59	0.36	3.39
	pilomatricoma	53	0.32	16.98
	connective tissue nevus	1	0.01	0.00
	sebaceous hyperplasia	1	0.01	0.00
Cutaneous lymphoma and lymphoid infiltrates	cutaneous t cell lymphoma	159	0.96	5.03
Epidermal tumors hamartomas milia and growths benign	porokeratosis	288	1.74	3.47
	epidermal nevus	246	1.49	8.13
	prurigo nodularis	169	1.02	24.26
	milia	106	0.64	13.21
	seborrheic keratosis	69	0.42	5.80
	acquired digital fibrokeratoma	1	0.01	0.00
Epidermal tumors pre malignant and malignant	basal cell carcinoma	434	2.63	2.30
	squamous cell carcinoma	261	1.58	1.53

	keratoacanthoma	184	1.11	3.80
	actinic keratosis	128	0.77	2.34
	bowens disease	72	0.44	5.56
	solid cystic basal cell carcinoma	67	0.41	0.00
	basal cell carcinoma morpheiform	62	0.38	1.61
	keratosis solar	29	0.18	0.00
	verrucous carcinoma	26	0.16	0.00
	erythroplasia queyrat	24	0.15	0.00
	arsenical keratoses	11	0.07	27.27
	keratoacanthoma pendulum	9	0.05	22.22
	basal cell carcinoma lesion	4	0.02	0.00
	basal cell carcinoma lid	4	0.02	0.00
	basal cell carcinoma scalp	4	0.02	0.00
	hypertrophic actinic keratosis	4	0.02	25.00
	basal cell carcinoma aldara	3	0.02	0.00
	basal cell carcinoma ear	3	0.02	0.00
	basal cell carcinoma lip	3	0.02	0.00
	basal cell carcinoma trunk ext	3	0.02	0.00
	basal cell carcinoma nose	2	0.01	0.00
	basal cell carcinoma superficial	2	0.01	0.00
	actinic cheilitis	1	0.01	0.00
	basal cell nevus syndrome	1	0.01	100.00
Genodermatoses and supernumerary growths	neurofibromatosis	189	1.14	30.69
	hailey hailey disease	174	1.05	1.72
	dariers disease	152	0.92	5.92
	tuberous sclerosis	141	0.85	9.22
	ehlers danlos syndrome	127	0.77	61.42
	incontinentia pigmenti	102	0.62	9.80
	acrodermatitis enteropathica	92	0.56	9.78
	xeroderma pigmentosum	81	0.49	7.41
	epidermolysis bullosa	70	0.42	10.00
	ichthyosis vulgaris	65	0.39	13.85
Inflammatory	acne vulgaris	513	3.10	7.21

**psoriasis**	481	2.91	6.24
**sarcoidosis**	350	2.12	6.00
**photodermatoses**	349	2.11	9.74
**erythema multiforme and fixed drug eruptions**	339	2.05	8.55
**scabies**	339	2.05	14.16
**malformations of extremities**	324	1.96	4.63
**pityriasis rubra pilaris**	278	1.68	3.24
**drug eruption**	265	1.60	5.28
**nematode infection**	260	1.57	8.85
**lichen planus**	254	1.54	9.45
**depositional diseases calcinosis cutis amyloid mucinosis gout and others**	219	1.33	20.09
**granuloma annulare**	210	1.27	3.33
**folliculitis**	204	1.23	23.04
**gyrate erythemas**	204	1.23	4.90
**histiocytic disorders and proliferations**	194	1.17	11.34
**pityriasis rosea**	192	1.16	13.54
**eczema**	188	1.14	7.45
**pigmentation diseases**	165	1.00	10.91
**acanthosis nigricans and carp**	163	0.99	14.11
**tungiasis**	152	0.92	9.21
**pediculosis lids**	151	0.91	3.97
**dermatomyositis**	150	0.91	9.33
**scarring**	148	0.90	9.46
**contact dermatitis**	137	0.83	0.73
**atrophic skin disease striae and elastic fiber disorders**	131	0.79	8.40
**allergic contact dermatitis**	128	0.77	3.91
**porphyria**	128	0.77	10.94
**seborrheic dermatitis**	125	0.76	10.40
**necrobiosis lipoidica**	123	0.74	2.44
**urticaria**	120	0.73	10.00
**lupus subacute**	109	0.66	3.67

**scleromyxedema**	108	0.65	5.56
**mastocytosis**	107	0.65	7.48
**cheilitis**	106	0.64	10.38
**scleroderma**	105	0.64	5.71
**rosacea**	103	0.62	6.80
**lupus erythematosus chronicus discoides**	99	0.60	10.10
**toxic epidermal necrolysis and stevens johnson**	99	0.60	14.14
**rhinophyma**	90	0.54	1.11
**hidradenitis**	89	0.54	14.61
**dyshidrotic eczema**	83	0.50	13.25
**pustular skin disease non infectious**	80	0.48	3.75
**lichen**	78	0.47	5.13
**panniculitis lipodystrophy and diseases of subcutis**	77	0.47	11.69
**keratosis pilaris and related follicular disorders**	75	0.45	0.00
**morphea**	75	0.45	2.67
**neurotic excoriations**	74	0.45	31.08
**tick bite**	72	0.44	27.78
**neurodermatitis**	69	0.42	2.90
**myiasis**	67	0.41	31.34
**factitial dermatitis**	66	0.40	10.61
**pityriasis lichenoides and pleva**	66	0.40	6.06
**lichen simplex**	65	0.39	24.62
**behcets disease**	64	0.39	18.75
**perioral dermatitis**	61	0.37	1.64
**acquired autoimmune bullous diseaseherpes gestationis**	60	0.36	6.67
**livedo reticularis**	60	0.36	8.33
**irritant contact dermatitis**	59	0.36	5.08
**paronychia**	59	0.36	1.69
**pseudofolliculitis barbae**	57	0.34	21.05
**rhus dermatitis**	57	0.34	0.00

**subacute cutaneous lupus erythematosus scle**	57	0.34	1.75
**xanthomas**	57	0.34	12.28
**erythema elevatum diutinum**	55	0.33	3.64
**lichen nitidus**	55	0.33	9.09
**sunburn**	54	0.33	9.26
**lichen planus follicularis**	44	0.27	4.55
**eosinophilic pustular folliculitis**	41	0.25	17.07
**psoriasis inversa**	38	0.23	5.26
**stasis dermatitis**	36	0.22	2.78
**progressive systemic scleroderma**	31	0.19	0.00
**psoriasis vulgaris guttate type**	31	0.19	6.45
**dermographia**	28	0.17	3.57
**localized scleroderma**	28	0.17	17.86
**lupus erythematosus chronicus verrucous**	28	0.17	35.71
**linear scleroderma**	24	0.15	20.83
**lupus erythematosus systemic**	24	0.15	16.67
**patch testing**	23	0.14	17.39
**lichen planus actinicus**	20	0.12	10.00
**lupus chronic cutaneous**	19	0.11	5.26
**malassezia folliculitis**	18	0.11	16.67
**psoriasis arthropathica**	18	0.11	0.00
**morphoea generalized**	17	0.10	17.65
**lichen planus linearis**	15	0.09	26.67
**psoriasis nail psoriasis**	15	0.09	13.33
**lichen planus vesicular**	14	0.08	0.00
**morphea linear**	12	0.07	8.33
**sun damaged skin**	12	0.07	0.00
**reticular erythematous mucinosis**	11	0.07	27.27
**stasis ulcer**	11	0.07	0.00
**lupus erythematosus chronicus disseminatus superficialis**	10	0.06	10.00
**pityrosporum folliculitis**	10	0.06	0.00

metal dermatitis	9	0.05	11.11
stasis edema	9	0.05	0.00
lupus acute	8	0.05	0.00
morphoea nodular	8	0.05	0.00
inverse psoriasis	7	0.04	0.00
lupus erythematosus tumidus	7	0.04	0.00
psoriasis palms soles	7	0.04	0.00
shoe allergy	7	0.04	42.86
folliculitis of barbae	6	0.04	0.00
lichen planus verrucosus	6	0.04	16.67
lupus erythematosus subacute cutaneous lupus erythematosus	6	0.04	0.00
miliaria	6	0.04	0.00
myxoedema pretibial	6	0.04	0.00
stasis dermatitis and ulcers	6	0.04	0.00
lupus erythematosus systemic lupus erythematosus	5	0.03	0.00
morphoea localized	5	0.03	0.00
nail psoriasis	5	0.03	20.00
pseudomonas folliculitis	5	0.03	20.00
psoriasis penis	5	0.03	0.00
herpes zoster	4	0.02	0.00
lichen planus annular	4	0.02	25.00
lupus erythematosus mucosae oris	4	0.02	0.00
psoriasis psoriasis after erysipelas	4	0.02	0.00
psoriasis scalp	4	0.02	0.00
subacute cutaneous lupus erythematosus	4	0.02	50.00
acne closed comedo	3	0.02	0.00
contact dermatitis leg ulcers	3	0.02	33.33
cosmetic fragrance allergy	3	0.02	0.00
eczema lids	3	0.02	0.00
grovers disease	3	0.02	0.00
hematogenous allergic contact dermatitis	3	0.02	0.00

	lichen planus of the mucosa	3	0.02	0.00
	acne cystic	2	0.01	0.00
	dermatoliposclerosis	2	0.01	50.00
	eczema acute	2	0.01	0.00
	eczema hand	2	0.01	0.00
	eczema scrotum	2	0.01	0.00
	eczema vulva	2	0.01	0.00
	impetigo	2	0.01	0.00
	psoriasis chronic plaque	2	0.01	0.00
	psoriasis nails	2	0.01	0.00
	acne inversa	1	0.01	100.00
	biting fleas	1	0.01	100.00
	bullous pemphigoid	1	0.01	0.00
	culicosis bullosa	1	0.01	0.00
	eczema areola	1	0.01	0.00
	eczema arms	1	0.01	0.00
	eczema nummular	1	0.01	0.00
	granuloma multiforme	1	0.01	0.00
	id reaction	1	0.01	0.00
	metabolic and nutritional diseases	1	0.01	0.00
	molluscum contagiosum	1	0.01	0.00
	parapsoriasis en plaques	1	0.01	0.00
	perforating disorders	1	0.01	0.00
	psoriasis hand	1	0.01	0.00
	reiters disease	1	0.01	0.00
	rosacea nose	1	0.01	0.00
	scalp psoriasis	1	0.01	0.00
	scarring alopecia	1	0.01	0.00
	skin diseases of pregnancy	1	0.01	0.00
	staphylococcal folliculitis	1	0.01	0.00
	varicosis	1	0.01	0.00
	verruca vulgaris	1	0.01	0.00
**Malignant dermal tumor**	**intermediate or malignant vascular**	127	0.77	6.30

	**tumors**			
**Pigmented lesions benign**	**halo nevus**	82	0.50	18.29
	**nevocytic nevus**	82	0.50	4.88
	**congenital nevus**	66	0.40	13.64
	**blue nevus**	1	0.01	0.00
**Pigmented lesions malignant**	**malignant melanoma**	369	2.23	3.25
	**superficial spreading melanoma ssm**	118	0.71	7.63
	**lentigo maligna**	80	0.48	0.00
	**malignant melanoma metastatic**	1	0.01	0.00
	**Unknown**	473	2.86	6.13
	**All**	16525	100.00	8.85

Supplementary Table 9: Frequency Table and False Positive Rates of Diagnoses in the HDA Repository

Frequency table of diagnoses and False Positive Rates of evaluated skin lesion images in the HDA repository.
All, Number of all evaluated skin lesion images in the HDA repository; HDA, hellenic dermatlogical atlas; n, Number of occurrences of diagnosis; FPR, False positive rate (%) of the diagnosis in the HDA repository; Unknown, Diagnosis unknown or unmapped; %, Percentage of diagnosis in relation to all evaluated skin lesion images in the HDA repository.

Category	Diagnosis	n	%	FPR
Benign dermal tumors cysts sinuses	hemangioma	16	0.61	6.25
	becker nevus	14	0.54	35.71
	pyogenic granuloma	12	0.46	16.67
	granulation tissue	10	0.38	20.00
	skin tag	9	0.34	22.22
	lymphangioma	8	0.31	0.00
	dermatofibroma	7	0.27	42.86
	fordyce spots	5	0.19	60.00
	mucous cyst	5	0.19	20.00
	steatocystoma multiplex	4	0.15	0.00
	angiokeratomas	3	0.11	100.00
	hemangioma racemosum	3	0.11	66.67
	spider nevus	3	0.11	66.67
	syringoma	3	0.11	0.00
	eruptive vellus hair cysts	2	0.08	0.00
	leiomyoma	2	0.08	0.00
	pilar cyst	2	0.08	0.00
	telangiectases	2	0.08	50.00
	venous lake	2	0.08	0.00
	cylindroma	1	0.04	100.00
	fibroma	1	0.04	0.00
	granuloma pyogenic	1	0.04	100.00
	pilomatricoma	1	0.04	0.00
	sebaceous hyperplasia	1	0.04	100.00

	trichoepithelioma	1	0.04	0.00
Cutaneous lymphoma and lymphoid infiltrates	cutaneous t cell lymphoma	33	1.26	12.12
	lymphoma b cell	2	0.08	0.00
	pseudo lymphoma other types	1	0.04	0.00
Epidermal tumors hamartomas milia and growths benign	epidermal nevus	28	1.07	21.43
	seborrheic keratosis	13	0.50	0.00
	corns	8	0.31	12.50
	porokeratosis	5	0.19	20.00
	callus	4	0.15	0.00
	milia	3	0.11	0.00
	giant comedo	1	0.04	0.00
Epidermal tumors pre malignant and malignant	basal cell carcinoma	64	2.45	25.00
	squamous cell carcinoma	32	1.22	12.50
	keratoacanthoma	8	0.31	12.50
	actinic keratosis	7	0.27	0.00
	bowens disease	7	0.27	0.00
	hypertrophic actinic keratosis	5	0.19	0.00
	actinic keratosis pigmented	4	0.15	25.00
	leukoplakia	4	0.15	0.00
	pagets disease	3	0.11	0.00
	actinic keratosis lesion	2	0.08	0.00
	basal cell carcinoma lid	2	0.08	50.00
	basal cell carcinoma superficial	2	0.08	0.00
	erythroplasia queyrat	2	0.08	0.00
	keratoacanthoma pendulum	2	0.08	0.00
	nevoid basal cell carcinoma syndrome	2	0.08	50.00
	actinic keratosis face	1	0.04	0.00
	basal cell carcinoma nose	1	0.04	0.00
	basal cell carcinoma ulcerating	1	0.04	0.00
Genodermatoses and supernumerary growths	neurofibromatosis	43	1.64	16.28
	ichthyosis vulgaris	23	0.88	34.78
	dariers disease	19	0.73	5.26
	tuberous sclerosis	17	0.65	23.53

	peutz jeghers syndrome	9	0.34	11.11
	acrokeratosis verruciformis	8	0.31	0.00
	epidermolysis bullosa	7	0.27	0.00
	lipoid proteinosis urbach wiethe disease	7	0.27	28.57
	leopard syndrome	5	0.19	0.00
	ehlers danlos syndrome	4	0.15	25.00
	pseudoxanthoma elasticum	3	0.11	0.00
	x linked ichthyosis	3	0.11	33.33
	ichthyosis sex linked	2	0.08	0.00
	albinism	1	0.04	100.00
	hailey hailey disease	1	0.04	0.00
	supernumerary growths	1	0.04	0.00
	xeroderma pigmentosum	1	0.04	100.00
Inflammatory	scarring	48	1.84	16.67
	granuloma annulare	45	1.72	11.11
	herpes zoster	42	1.61	4.76
	onychomycosis	41	1.57	4.88
	drug eruption	36	1.38	2.78
	non scarring alopecia	35	1.34	28.57
	pityriasis rosea	35	1.34	14.29
	discoid lupus erythematosus	34	1.30	14.71
	lichen sclerosus skin	33	1.26	12.12
	malformations of extremities	32	1.22	9.38
	psoriasis	31	1.19	12.90
	pigmentation diseases	30	1.15	23.33
	urticaria	27	1.03	0.00
	lichen planus annular	26	0.99	3.85
	condyloma acuminata	25	0.96	28.00
	cutaneous manifestations of systemic disease	24	0.92	8.33
	pustular skin disease non infectious	24	0.92	12.50
	seborrheic dermatitis	24	0.92	29.17
	syphilis	24	0.92	8.33

**insect bite**	22	0.84	18.18
**warts**	22	0.84	9.09
**pityriasis versicolor**	21	0.80	9.52
**reiters disease**	21	0.80	0.00
**acne cystic**	19	0.73	10.53
**erythema multiforme and fixed drug eruptions**	19	0.73	21.05
**impetigo**	19	0.73	10.53
**pemphigus vulgaris**	19	0.73	0.00
**psoriasis nails**	19	0.73	0.00
**solar purpura**	19	0.73	10.53
**bullous pemphigoid**	18	0.69	11.11
**lichen planus penis**	18	0.69	16.67
**mastocytosis**	18	0.69	11.11
**atrophic skin disease striae and elastic fiber disorders**	16	0.61	18.75
**herpes simplex**	16	0.61	0.00
**photodermatoses**	16	0.61	6.25
**pityriasis lichenoides and pleva**	16	0.61	31.25
**tinea pedis**	16	0.61	0.00
**solar elastosis**	15	0.57	20.00
**atopic dermatitis**	14	0.54	7.14
**dermatomyositis**	14	0.54	21.43
**epidermolysis bullosa acquisita**	14	0.54	35.71
**molluscum contagiosum**	14	0.54	50.00
**morphea**	14	0.54	7.14
**tinea faciale**	14	0.54	21.43
**allergic contact dermatitis**	13	0.50	7.69
**candidiasis**	13	0.50	7.69
**lichen**	13	0.50	0.00
**scabies**	13	0.50	15.38
**tinea incognito**	13	0.50	0.00
**genital warts**	12	0.46	0.00
**lichen nitidus**	12	0.46	0.00

**rhus dermatitis**	12	0.46	0.00
**solar damage atrophy**	12	0.46	8.33
**fungal infections dermal and subcut**	11	0.42	18.18
**hair disorderstrichorrhexis nodosa etc**	11	0.42	18.18
**leukocytoclastic vasculitis**	11	0.42	45.45
**tinea corporis**	11	0.42	27.27
**tinea cruris**	11	0.42	18.18
**pityriasis rubra pilaris**	10	0.38	10.00
**sarcoidosis**	10	0.38	0.00
**scarring alopecia**	10	0.38	20.00
**tinea capitis**	10	0.38	20.00
**acanthosis nigricans and carp**	9	0.34	0.00
**balanitis**	9	0.34	0.00
**behcets disease**	9	0.34	44.44
**lupus erythematosus chronicus disseminatus superficialis**	9	0.34	0.00
**neurodermatitis**	9	0.34	0.00
**perioral dermatitis**	9	0.34	11.11
**psoriasis treatment**	9	0.34	11.11
**scleroderma**	9	0.34	44.44
**small pox**	9	0.34	0.00
**erysipelas**	8	0.31	12.50
**erythrasma**	8	0.31	12.50
**necrobiosis lipoidica**	8	0.31	12.50
**panniculitis lipodystrophy and diseases of subcutis**	8	0.31	50.00
**subacute cutaneous lupus erythematosus**	8	0.31	75.00
**acne necrotica**	7	0.27	0.00
**erythema infectiosum**	7	0.27	0.00
**folliculitis**	7	0.27	14.29
**grovers disease**	7	0.27	0.00
**histiocytic disorders and proliferations**	7	0.27	0.00
**lichen planopilaris**	7	0.27	14.29

**nail beading**	7	0.27	28.57
**progressive systemic scleroderma**	7	0.27	42.86
**toxic epidermal necrolysis and stevens johnson**	7	0.27	0.00
**xanthomas**	7	0.27	28.57
**acne infantile**	6	0.23	50.00
**dermatitis swimming**	6	0.23	0.00
**eczema**	6	0.23	0.00
**hypereosinophilic syndrome**	6	0.23	0.00
**infected eczema**	6	0.23	16.67
**lupus subacute**	6	0.23	16.67
**pemphigus foliaceus**	6	0.23	0.00
**porphyria**	6	0.23	83.33
**reticular erythematous mucinosis**	6	0.23	16.67
**rhinophyma**	6	0.23	16.67
**rosacea**	6	0.23	16.67
**tick bite**	6	0.23	16.67
**cheilitis**	5	0.19	20.00
**clubbing**	5	0.19	0.00
**gyrate erythemas**	5	0.19	0.00
**hairy tongue**	5	0.19	20.00
**ingrown nail**	5	0.19	20.00
**nail psoriasis**	5	0.19	0.00
**paronychia**	5	0.19	0.00
**planar warts**	5	0.19	0.00
**radiodermatitis**	5	0.19	20.00
**thrombocytopenic purpura idiopatic**	5	0.19	0.00
**tinea manuum**	5	0.19	0.00
**acne comedonica**	4	0.15	0.00
**acne cystica**	4	0.15	0.00
**acroangiodermatitis**	4	0.15	0.00
**crest syndrome**	4	0.15	25.00
**dermographia**	4	0.15	0.00

**dyshidrotic eczema**	4	0.15	0.00
**epidermodysplasia verruciformis**	4	0.15	0.00
**furuncle**	4	0.15	0.00
**green nail**	4	0.15	0.00
**hand foot mouth**	4	0.15	25.00
**herpes simplex labialis**	4	0.15	25.00
**hidradenitis**	4	0.15	0.00
**keratosis pilaris and related follicular disorders**	4	0.15	0.00
**leukonychia**	4	0.15	0.00
**pachyonychia congenita**	4	0.15	0.00
**psoriasis penis**	4	0.15	0.00
**spider bite**	4	0.15	0.00
**thermal burn**	4	0.15	0.00
**tuberculosis**	4	0.15	0.00
**acne fulminans**	3	0.11	33.33
**cholinergic urticaria**	3	0.11	0.00
**erythema ab igne**	3	0.11	0.00
**granuloma**	3	0.11	33.33
**herpes gestationis**	3	0.11	33.33
**hyperhidrosis**	3	0.11	0.00
**inverse psoriasis**	3	0.11	0.00
**lichen planus**	3	0.11	0.00
**metabolic and nutritional diseases**	3	0.11	0.00
**nail polish dermatitis**	3	0.11	0.00
**nummular eczema**	3	0.11	0.00
**pernio and mimics**	3	0.11	0.00
**pitted keratolysis**	3	0.11	0.00
**acne keloidalis**	2	0.08	0.00
**atopic dermatitis feet**	2	0.08	0.00
**dermatoliposclerosis**	2	0.08	0.00
**factitial dermatitis**	2	0.08	0.00
**irritant contact dermatitis**	2	0.08	0.00

**lichen planus of the mucosa**	2	0.08	50.00
**lupus miliaris disseminatus faciei**	2	0.08	0.00
**meningococcemia**	2	0.08	50.00
**onychogryphosis**	2	0.08	0.00
**parapsoriasis en plaques**	2	0.08	50.00
**pemphigoid bullous pemphigoid**	2	0.08	0.00
**pseudofolliculitis barbae**	2	0.08	0.00
**purpura**	2	0.08	50.00
**superficial fungal infections**	2	0.08	0.00
**ulcer**	2	0.08	0.00
**acne rosacea**	1	0.04	0.00
**acne vulgaris**	1	0.04	0.00
**angioedema**	1	0.04	0.00
**atopic eczema of the hands**	1	0.04	0.00
**balanitis bacterial**	1	0.04	100.00
**cercaria dermatitis**	1	0.04	0.00
**chronic recurrent aphthae**	1	0.04	100.00
**cimicosis**	1	0.04	0.00
**connective tissue disorders**	1	0.04	0.00
**contact dermatitis**	1	0.04	0.00
**depositional diseases calcinosis cutis amyloid mucinosis gout and others**	1	0.04	0.00
**dry skin eczema**	1	0.04	0.00
**ecthyma simplex**	1	0.04	0.00
**erysipeloid**	1	0.04	0.00
**folliculitis cheloidalis**	1	0.04	100.00
**fox fordyce disease**	1	0.04	100.00
**geographic tongue**	1	0.04	0.00
**guttate psoriasis**	1	0.04	100.00
**id reaction**	1	0.04	0.00
**intertrigo**	1	0.04	0.00
**lichen planus linearis**	1	0.04	0.00
**lichen planus of the mucosa erosive**	1	0.04	0.00

	median nail dystrophy	1	0.04	0.00
	metal dermatitis	1	0.04	0.00
	morphea linear	1	0.04	0.00
	myiasis	1	0.04	0.00
	nail diseases	1	0.04	0.00
	nail pigmentation	1	0.04	0.00
	onychogryphosis and onychauxis	1	0.04	0.00
	papulose bowenoide	1	0.04	0.00
	pediculosis lids	1	0.04	0.00
	pemphigus	1	0.04	0.00
	pityrosporum folliculitis	1	0.04	0.00
	psoriasis chronic plaque	1	0.04	100.00
	psoriasis light sensative	1	0.04	0.00
	psoriatic erythroderma	1	0.04	0.00
	scrophuloderma	1	0.04	0.00
	shaving folliculitis	1	0.04	0.00
	skin diseases of pregnancy	1	0.04	0.00
	splinter hemorrhage	1	0.04	0.00
	stasis dermatitis	1	0.04	0.00
	sunburn	1	0.04	0.00
	tattoo and foreign body reactions	1	0.04	0.00
	terry nails	1	0.04	0.00
	tinea facei	1	0.04	0.00
	tinea nigra	1	0.04	0.00
	trichomycosis	1	0.04	0.00
	urticarial vasculitis	1	0.04	0.00
	virus exanthem	1	0.04	0.00
	wart	1	0.04	100.00
	warts periungual	1	0.04	0.00
**Malignant dermal tumor**	**intermediate or malignant vascular tumors**	30	1.15	6.67
	**systemic carcinomas**	20	0.77	5.00
**Pigmented lesions benign**	**giant melanocytic nevus**	18	0.69	11.11

	**naevus blue**	13	0.50	23.08
	**melanocytic nevi**	8	0.31	12.50
	**blue nevus**	5	0.19	0.00
	**mucosal lentigines**	5	0.19	0.00
	**naevus achromic**	5	0.19	20.00
	**naevus halo**	5	0.19	0.00
	**mongolian spot**	4	0.15	50.00
	**atypical nevi**	3	0.11	0.00
	**blue nevus ota**	3	0.11	0.00
	**melanonychia striata**	3	0.11	0.00
	**hereditary dysplastic nevus syndrome**	2	0.08	0.00
	**café au lait spot**	1	0.04	0.00
	**spindle cell nevus**	1	0.04	0.00
**Pigmented lesions malignant**	**malignant melanoma**	17	0.65	0.00
	**lentigo maligna**	5	0.19	20.00
	**lentigo maligna melanoma**	1	0.04	0.00
	**nail melanoma**	1	0.04	0.00
	**Unknown**	174	6.66	14.94
	**All**	2614	100.00	13.70

Supplementary Table 10: Frequency Table and False Positive Rates of Diagnoses in the PAD-uefs 20 Dataset

Frequency table of diagnoses and False Positive Rates of evaluated skin lesion images in the PAD-uefs 20 dataset.
All, Number of all evaluated skin lesion images in the PAD-uefs 20 dataset; FPR, False positive rate (%) of the diagnosis in the PAD-uefs 20 dataset; n, Number of occurrences of diagnosis; Unknown, Diagnosis unknown or unmapped; %, Percentage of diagnosis in relation to all evaluated skin lesion images in the PAD-uefs 20 dataset.

Category	Diagnosis	n	%	FPR
**Epidermal tumors hamartomas milia and growths benign**	**seborrheic keratosis**	235	10.23	25.96
**Epidermal tumors pre malignant and malignant**	**basal cell carcinoma**	845	36.77	21.30
	**actinic keratosis**	730	31.77	23.56
	**squamous cell carcinoma**	192	8.36	27.08
**Pigmented lesions benign**	**nevus cell nevus**	244	10.62	14.75
**Pigmented lesions malignant**	**malignant melanoma**	52	2.26	9.62
	**All**	2298	100.00	22.02

Supplementary Table 11: Frequency Tables and False Positive Rates of Diagnoses of the non-MPXV dataset as Excel File

The content of supplementary tables 1-10 is additionally provided as a separate file (Microsoft Excel) as supplementary table 11.

Supplementary Table 12: Performance in the detection of MPXV skin lesions using different model architectures.

Each model was trained and evaluated using a 5-fold cross-validation with the same data split in each fold. All the tested models converged after 18 to 20 epochs of training. The mean and standard deviation of sensitivity, specificity, and AUC from the 5-fold cross-validation were shown.
AUC, Area under receiver operating characteristic curve; M, Million; MPXV, Mpox virus; SD, Standard deviation.

Architecture	Number of training parameters	Sensitivity	Specificity	AUC
ResNet18	11.4M	0.84 (SD: 0.056)	0.964 (SD: 0.006)	0.963 (SD: 0.015)
ResNet34	21.5M	0.85 (SD: 0.055)	0.964 (SD: 0.012)	0.971 (SD: 0.009)
ResNet50	23.9M	0.83 (SD: 0.0986)	0.970 (SD: 0.006)	0.968 (SD: 0.010)
ResNet152	58.5M	0.86 (SD: 0.0658)	0.963 (SD: 0.005)	0.969 (SD: 0.009)
DenseNet169	12.8M	0.84 (SD: 0.074)	0.977 (SD: 0.008)	0.972 (SD: 0.011)
VGG19_bn	143M	0.85 (SD: 0.073)	0.970 (SD:0.001)	0.970 (SD: 0.014)

Supplementary Figure 1: True Positive Rates of the MPXV-CNN in the testing cohort in coalesced and non-coalesced MPXV skin lesions.

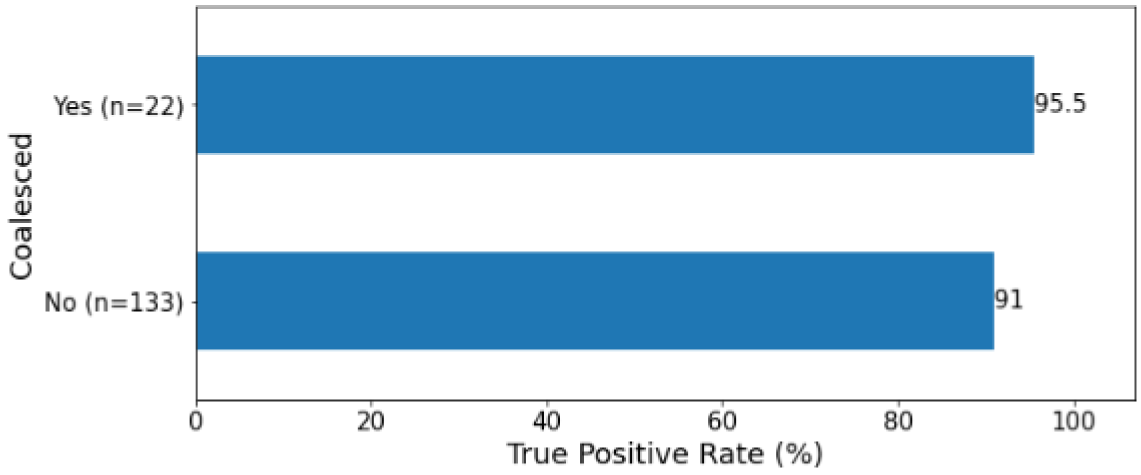

MPXV, mpox virus; MPXV-CNN, mpox virus convolutional neural network; n Number of available skin lesion images per group.

Supplementary Figure 2: False Positive Rates by category of non-MPXV skin disease in the testing cohort.

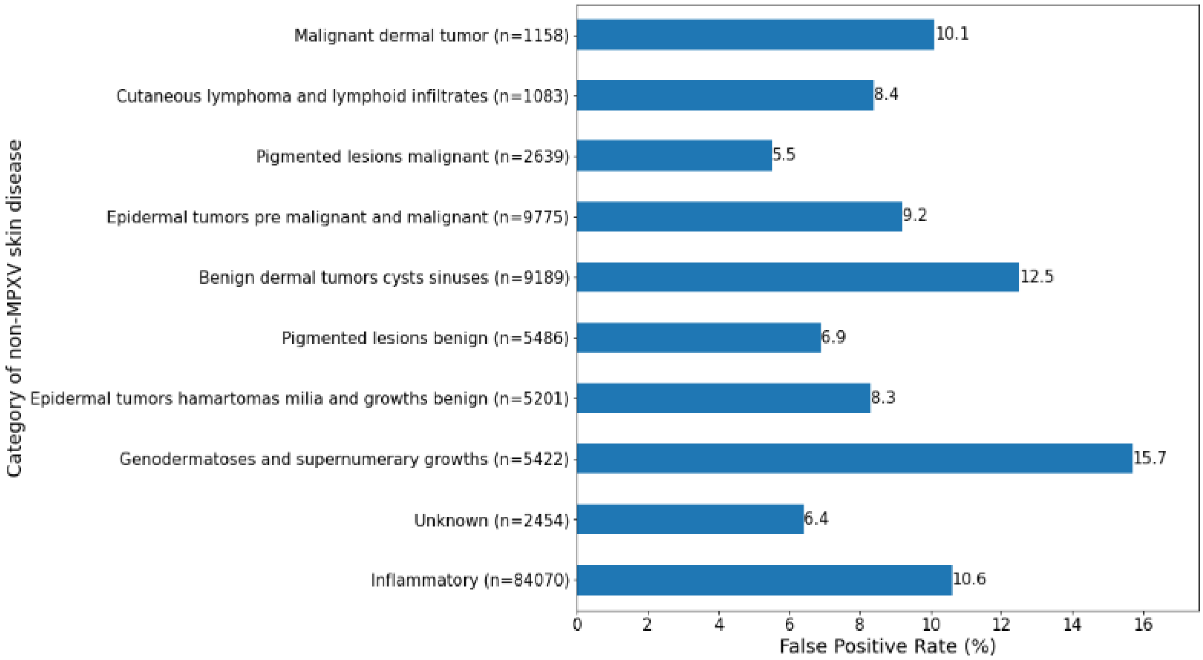

MPXV, mpox virus; n, Number of available skin lesion images per category in the non-MPXV dataset.

Supplementary Figure 3: False Positive Rates by differential diagnoses of MPXV in the testing cohort.

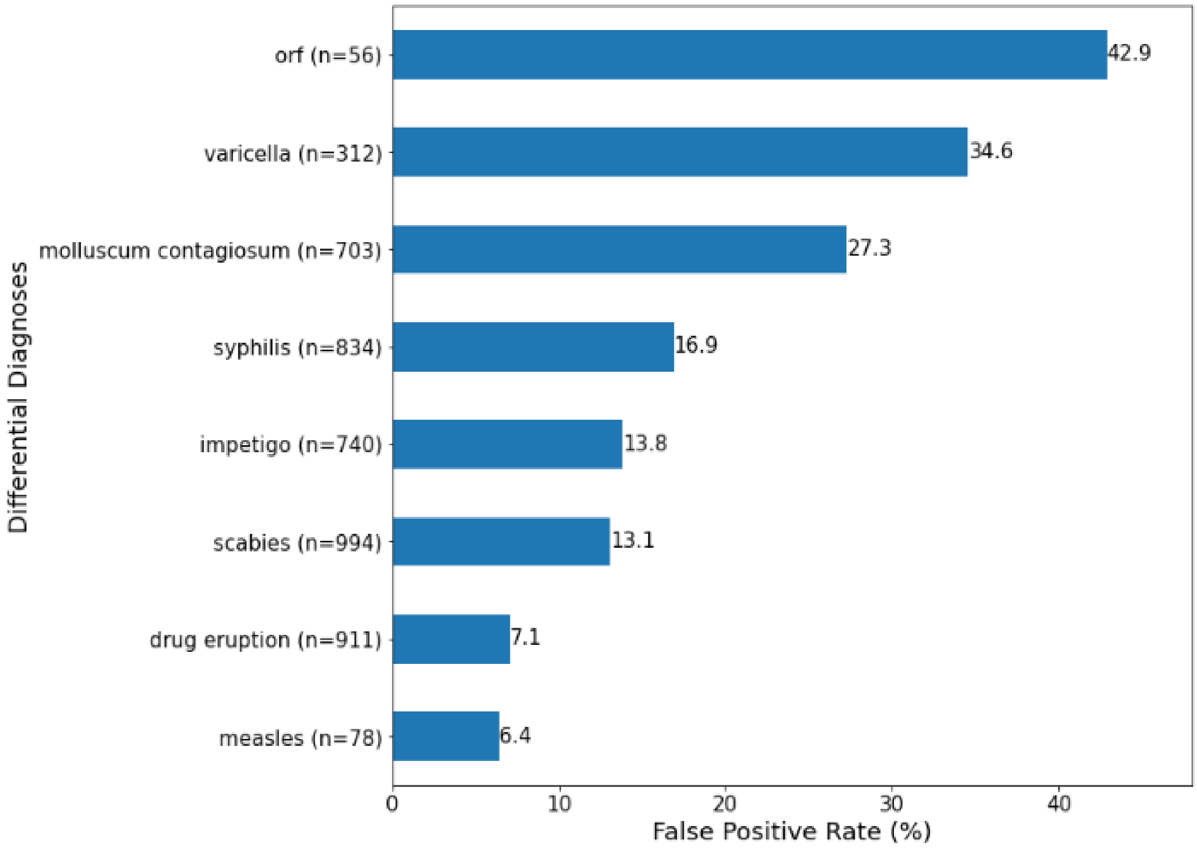

MPXV, mpox virus; n, Number of available skin lesion images per differential diagnoses in the non-MPXV dataset.

Supplementary Figure 4: False Positive Rates by body region of non-MPXV images in the testing cohort (DermIS repository).

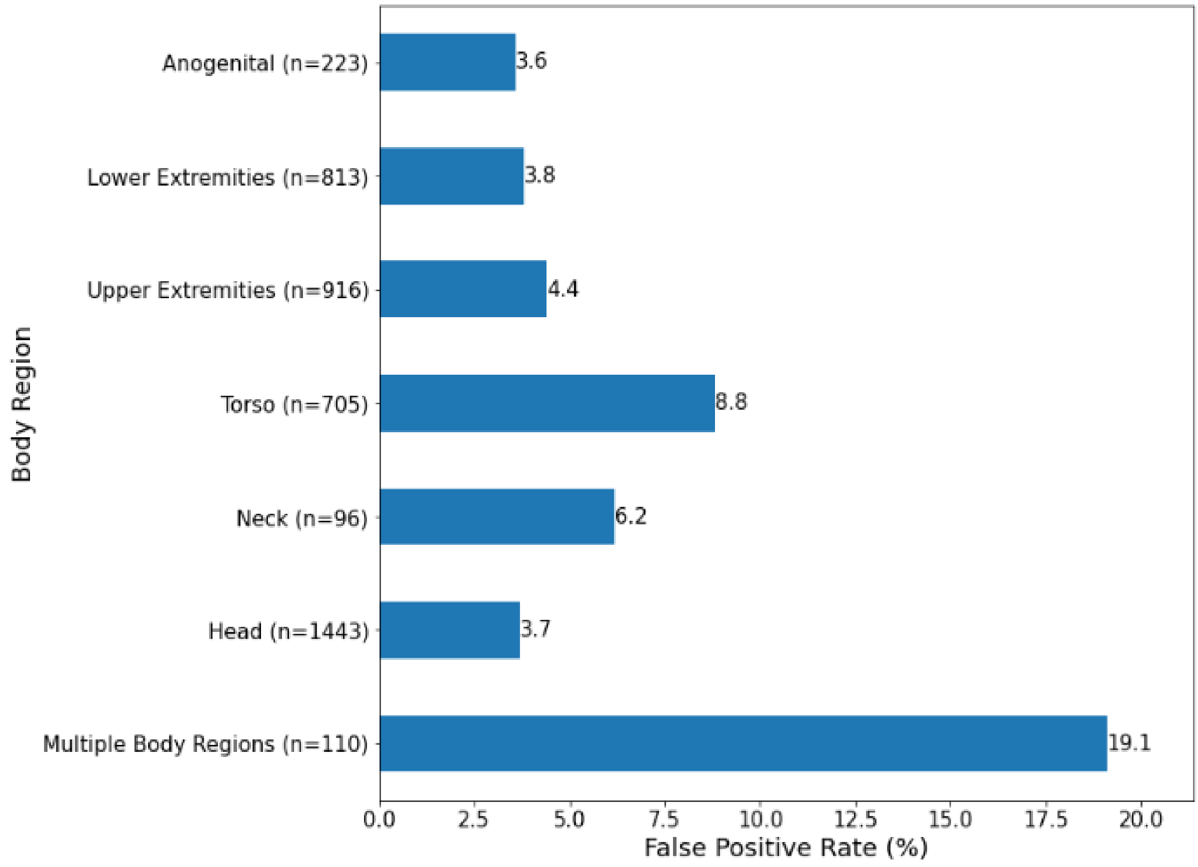

The highest FPR could be observed in images showing multiple body regions. MPXV, mpox virus; n, Number of available skin lesion images per body region.

Supplementary Figure 5: False Positive Rates by age group in non-MPXV images of the testing cohort (DermIS repository).

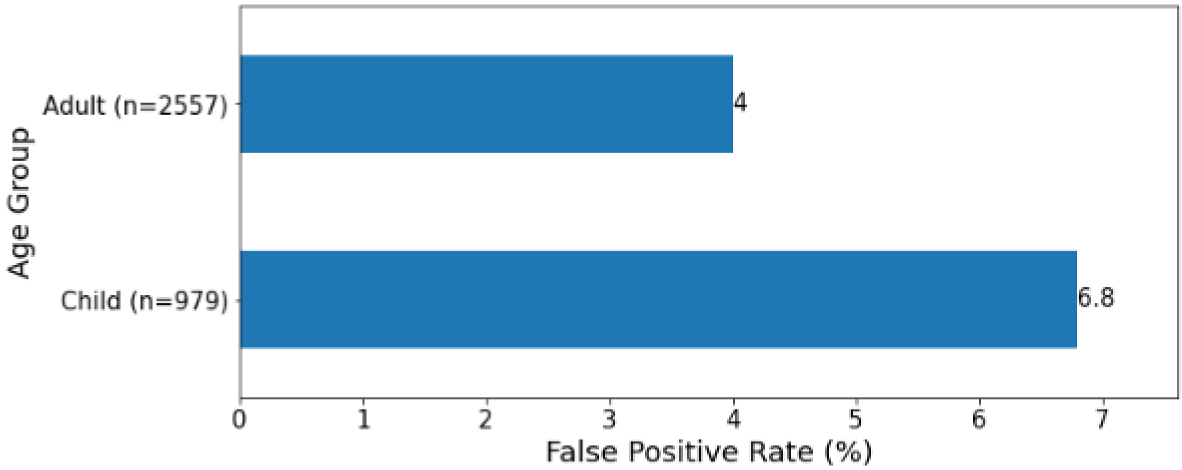

MPXV, mpox virus; n, Number of available skin lesion images per age group.

Supplementary Figure 6: False Positive Rates by sex in the testing cohort (DermIS and PAD-uefs 20).

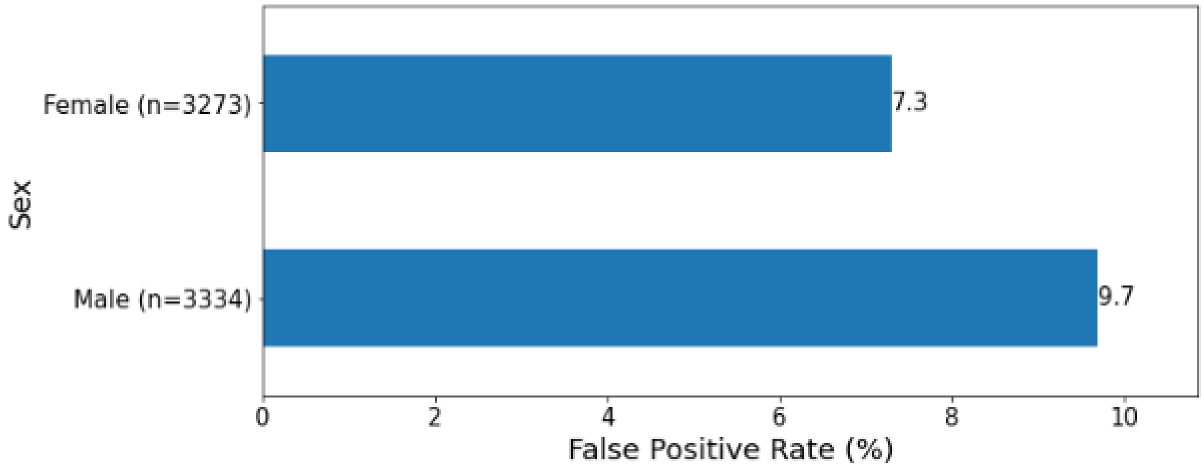

n, Number of available skin lesion images per sex.

Supplementary Figure 7: Screenshot of PoxApp for the question on the presence of a skin lesion.

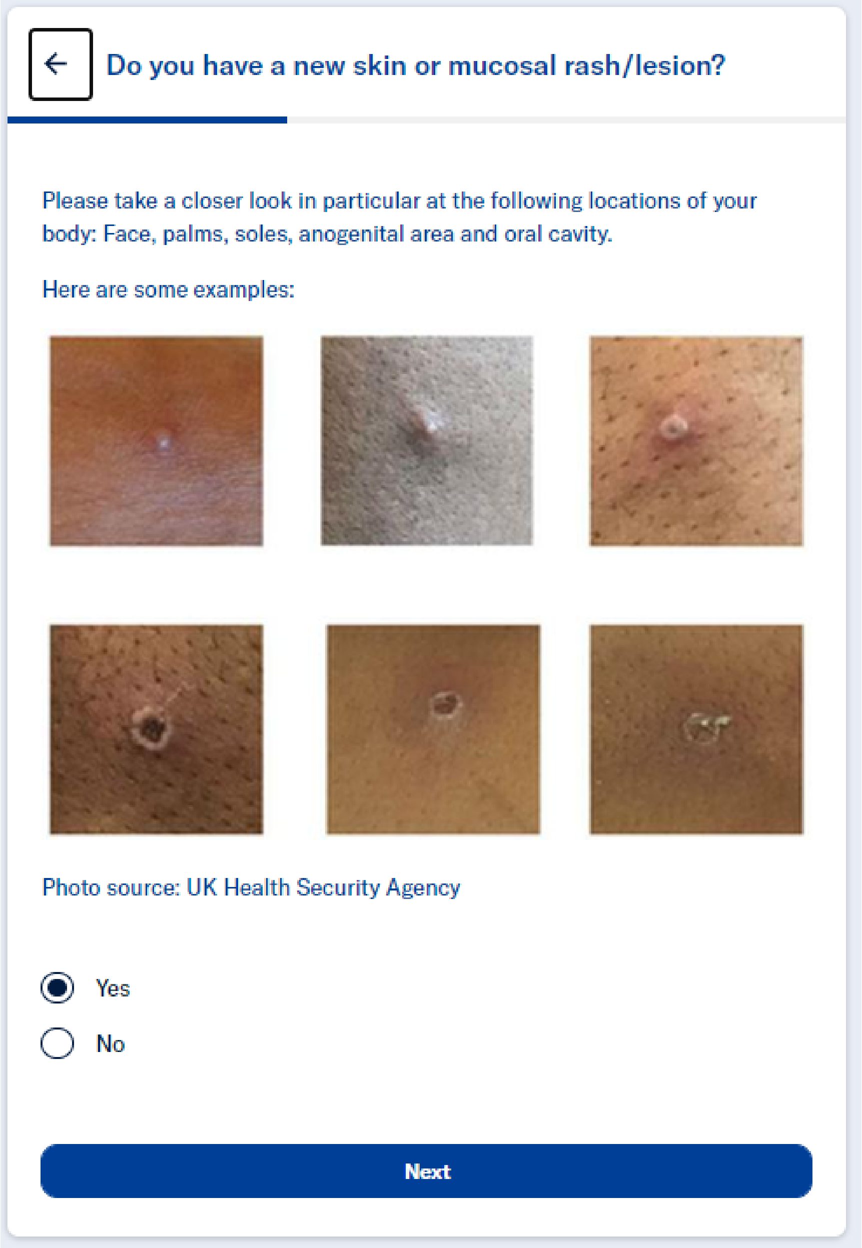

The user is intentionally asked very broadly to report any skin or mucosal lesion or rash. The sites of predilection of MPXV are named. Example images of MPXV skin lesions in different stages are shown without revealing to the user the underlying diagnosis. The sense of this approach is to prevent a filter effect of the question but to still sensitize users to the diverse appearance of MPXV skin lesions.

MPXV, mpox virus.

Supplementary Figure 8: Screenshot of PoxApp of interface for taking or selecting a picture of a skin lesion.

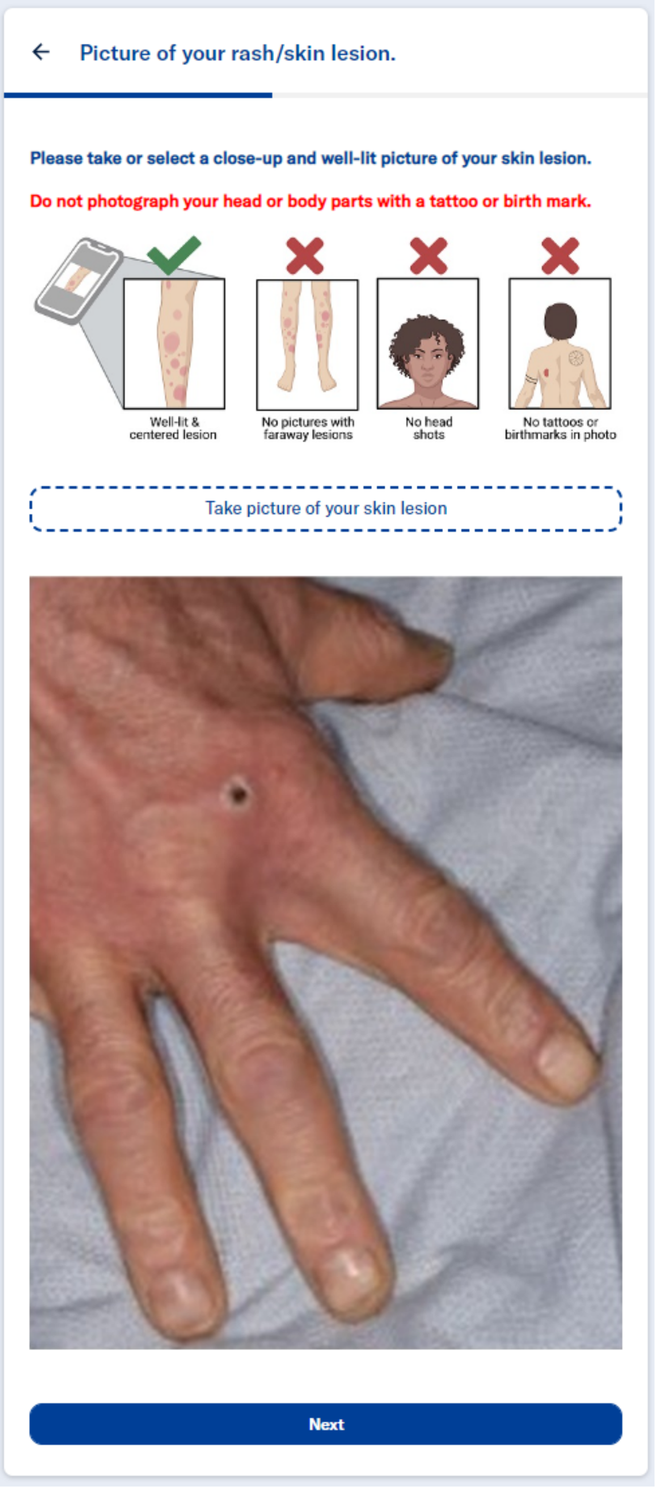

This question is optional and displayed only if a skin lesion is reported by the user. Instructions are given for taking the picture.

Supplementary Figure 9: Screenshot of PoxApp for question on typical MPXV symptoms.

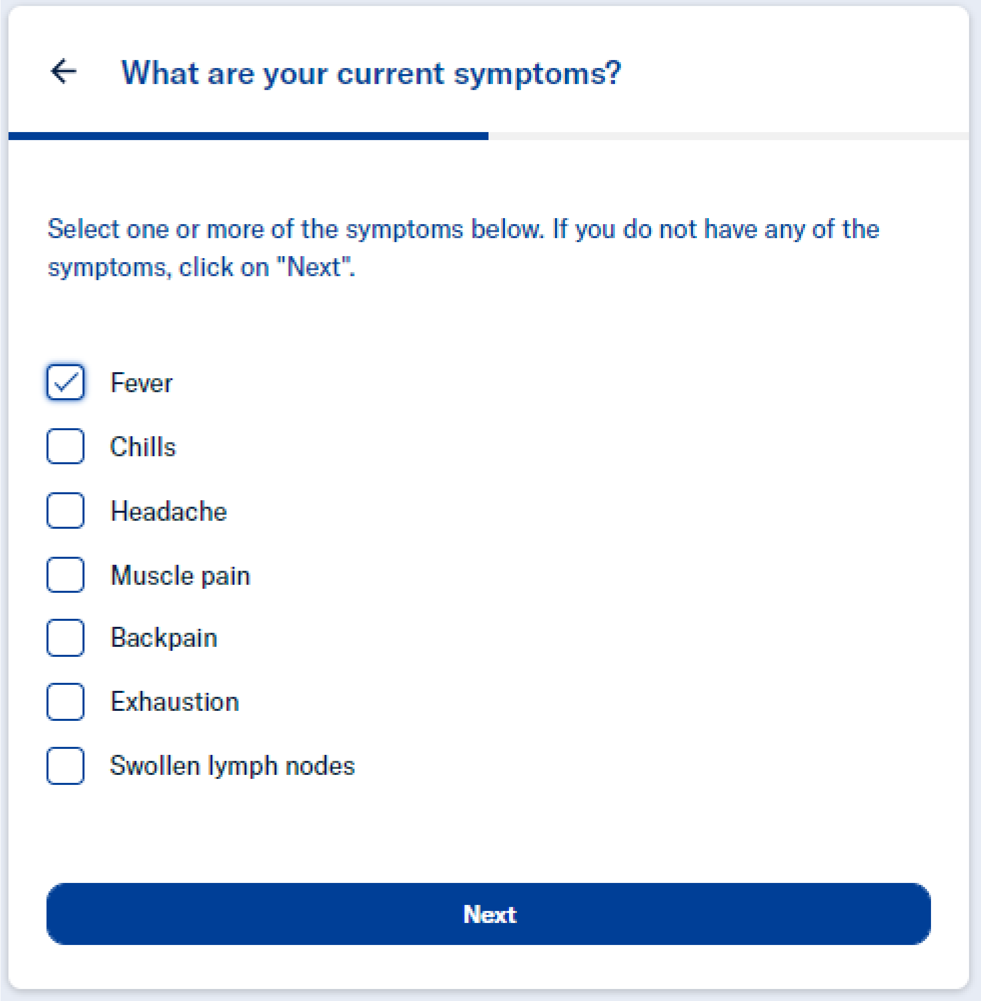

MPXV, mpox virus.

Supplementary Figure 10: Screenshot of PoxApp for date of onset of symptoms.

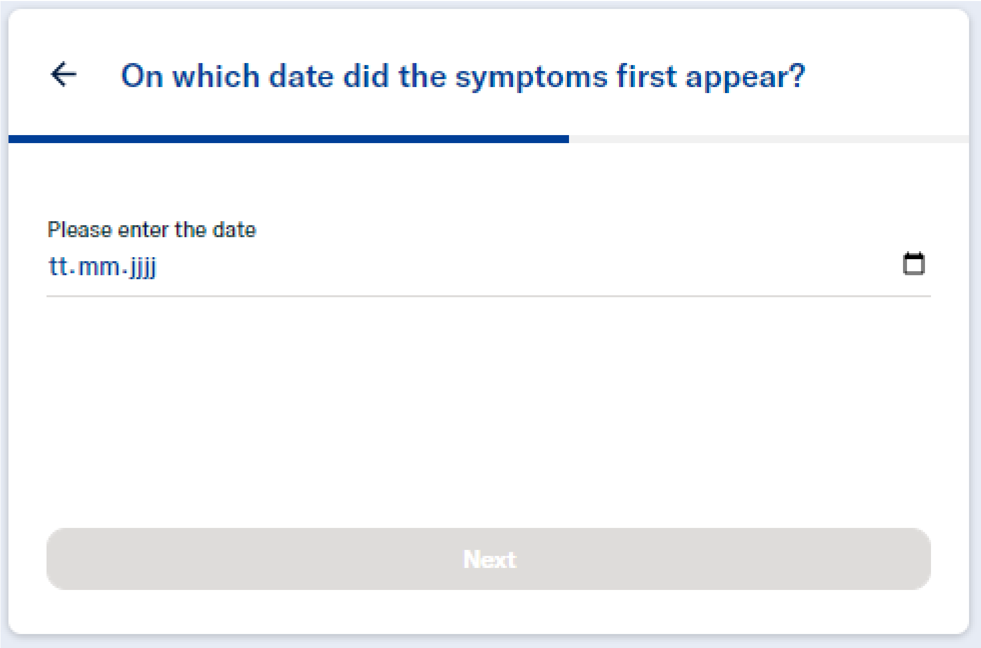

The date is used to differentiate between acute and chronic symptoms and the relevancy of the symptoms. This question is optional and displayed only if symptoms are reported by the user.

Supplementary Figure 11: Screenshot of PoxApp for the question on risk contacts.

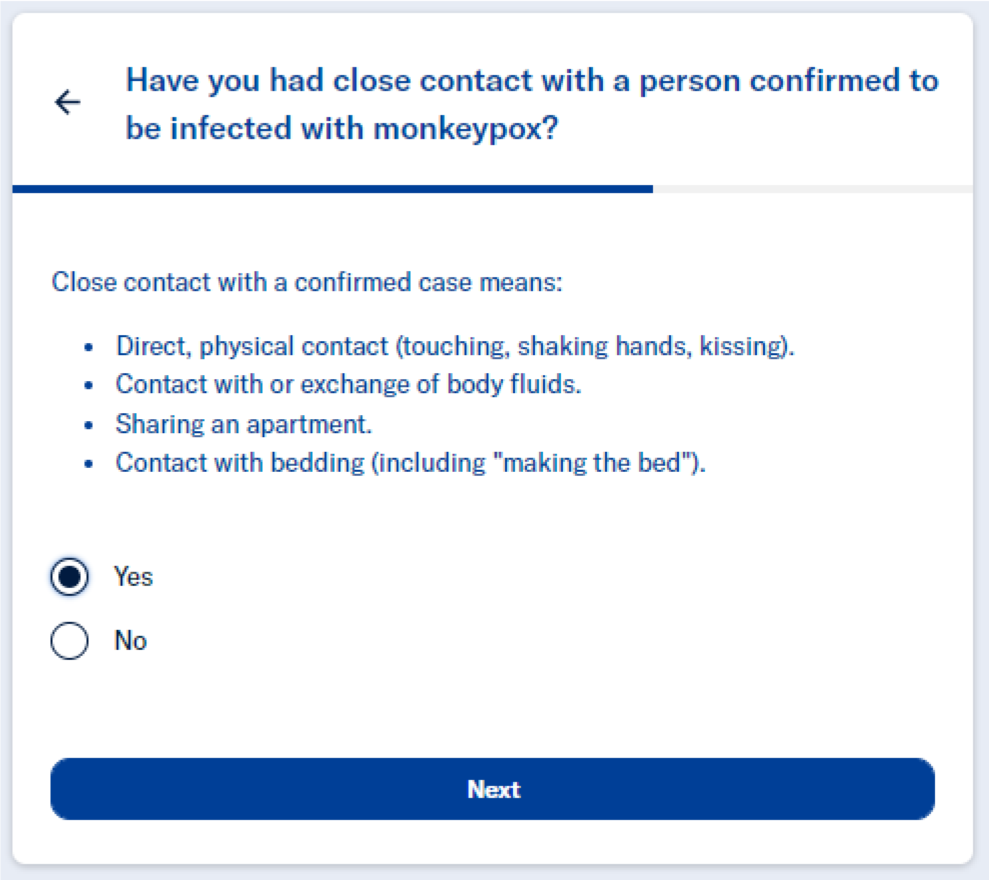

Question contains details on what is considered a risk contact.

Supplementary Figure 12: Screenshot of PoxApp for the question on the date of risk contact.

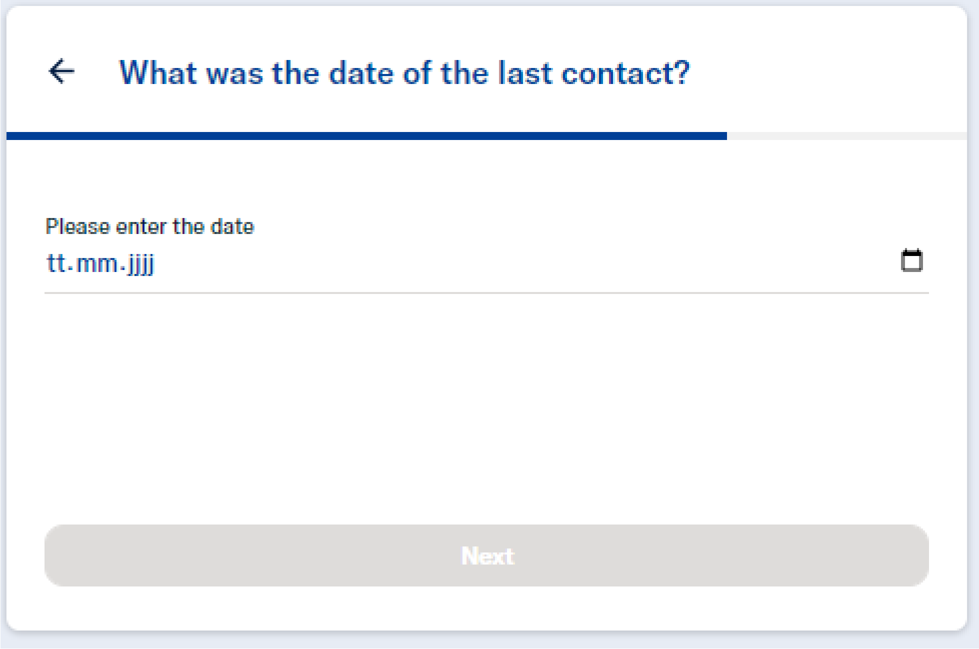

The date is used to evaluate the relevancy of this contact considering the incubation time of MPXV. This question is optional and displayed only if a risk contact is reported by the user.

MPXV, mpox virus.

Supplementary Figure 13: Screenshot of PoxApp for the question on sexual preferences.

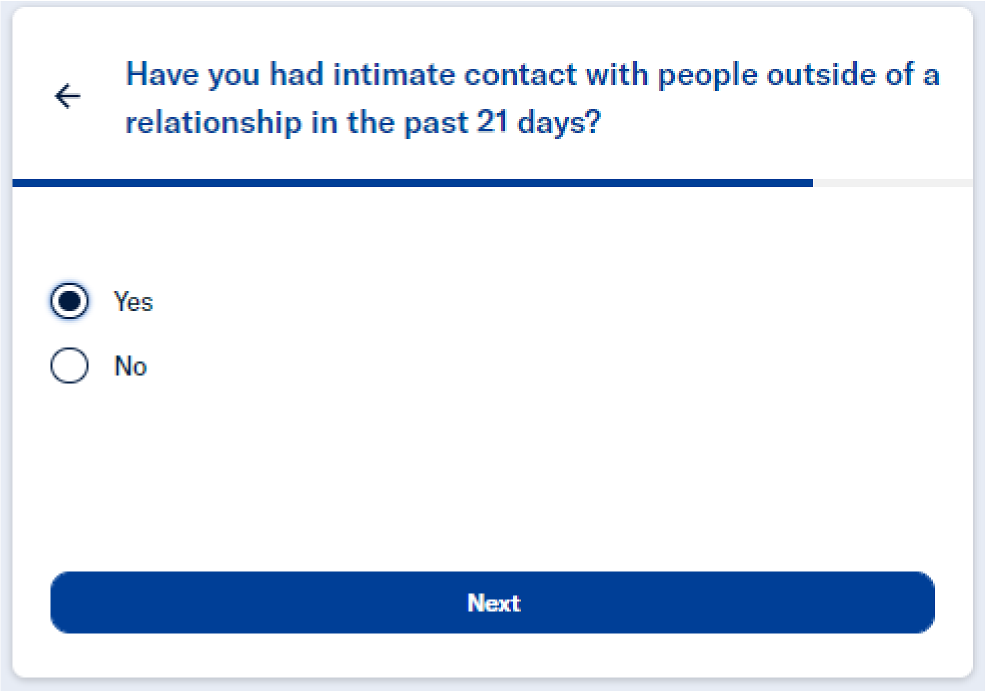

Supplementary Figure 14: Screenshot of PoxApp for the zip code question.

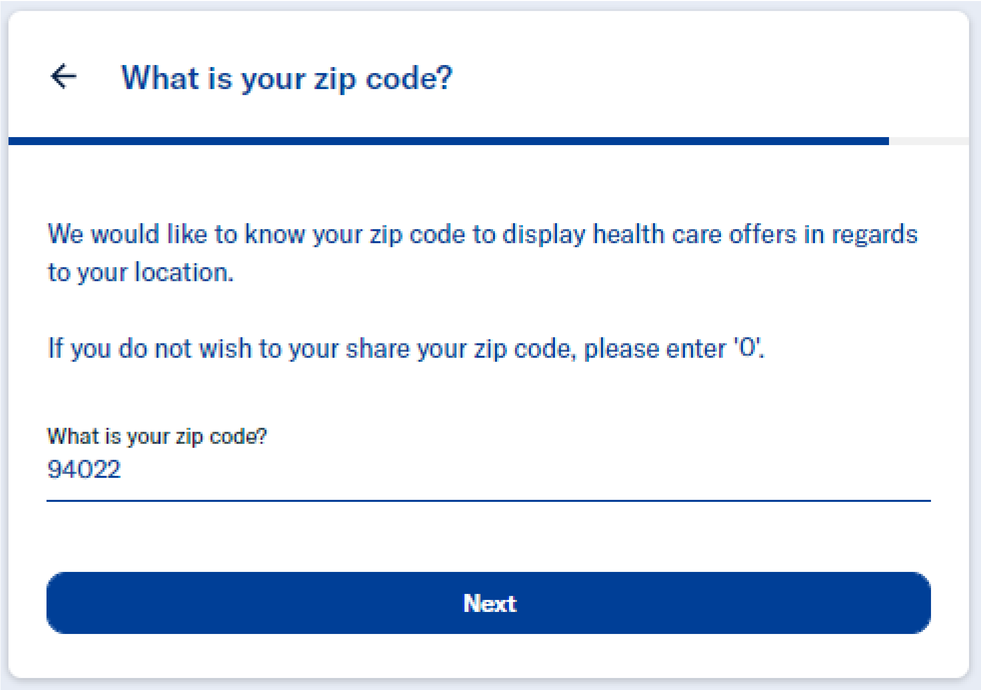

The zip code was evaluated by the app’s logic to display location-based healthcare offerings such as testing or vaccination sites.

Supplementary Figure 15: Logic decision tree of PoxApp for MPXV infection risk classification incorporating the MPXV-CNN.

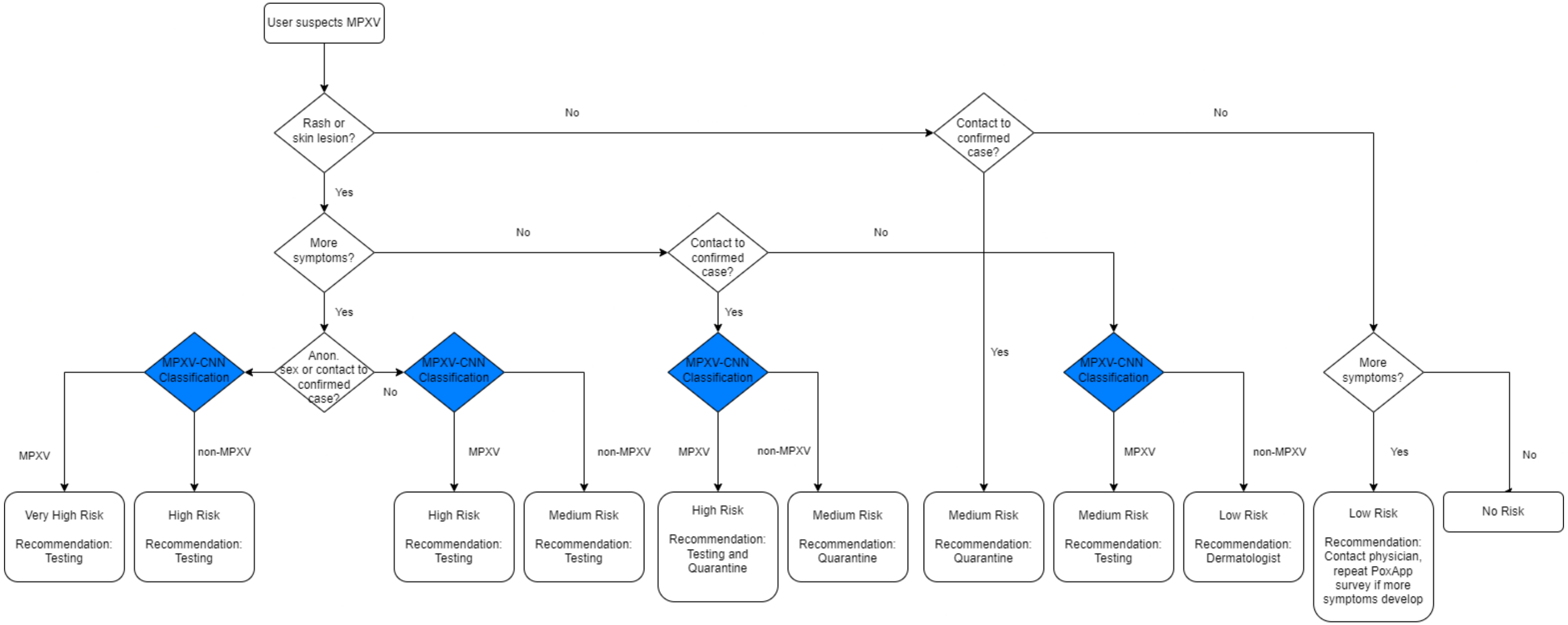

White rhombuses symbolize questions, blue rhombuses the MPXV-CNN, arrows logical expressions, and boxes risk classifications and recommendations.

MPXV, mpox virus; MPXV-CNN, mpox virus convolutional neural network.

Supplementary Note 1: Bibliography of sources with images of MPXV lesions.

1. Mateos Moreno D, Clavo Escribano P. Infección por virus de la viruela del mono. Caso clínico. Actas Dermosifiliogr [Internet]. [cited 2022 Jul 13]; Available from: http://www.actasdermo.org/es-infeccion-por-virus-viruela-del-articulo-S0001731022006020

2. AJN. Preocupación en Israel por la «viruela del mono»: intentos por adquirir vacunas contra la enfermedad [Internet]. AJN Agencia de Noticias. 2022 [cited 2022 Jul 13]. Available from: https://agenciaajn.com/noticia/preocupacion-en-israel-por-la-viruela-del-mono-intentos-por-adquirir-vacunas-contra-la-enfermedad-193923

3. Sklenovská N. Monkeypox Virus. In: Malik YS, Singh RK, Dhama K, editors. Animal-Origin Viral Zoonoses [Internet]. Singapore: Springer; 2020 [cited 2022 Jul 12]. p. 39–68. (Livestock Diseases and Management). Available from: https://doi.org/10.1007/978-981-15-2651-0_2

4. Lucar J, Roberts A, Saardi KM, Yee R, Siegel MO, Palmore TN. Monkeypox Virus–Associated Severe Proctitis Treated With Oral Tecovirimat: A Report of Two Cases. Ann Intern Med [Internet]. 2022 Aug 18 [cited 2022 Aug 27]; Available from: https://www.acpjournals.org/doi/full/10.7326/L22-0300

5. Català A, Clavo-Escribano P, Riera-Monroig J, Martín-Ezquerra G, Fernandez-Gonzalez P, Revelles-Peñas L, et al. Monkeypox outbreak in Spain: clinical and epidemiological findings in a prospective cross-sectional study of 185 cases. British Journal of Dermatology [Internet]. [cited 2022 Aug 22]. Available from: https://onlinelibrary.wiley.com/doi/abs/10.1111/bjd.21790

6. Eltvedt AK, Christiansen M, Poulsen A. A Case Report of Monkeypox in a 4-Year-Old Boy from the DR Congo: Challenges of Diagnosis and Management. Case Reports in Pediatrics. 2020 Apr 9;2020:e8572596.

7. CDC. Monkeypox in the U.S. [Internet]. Centers for Disease Control and Prevention. 2022 [cited 2022 Jul 12]. Available from: https://www.cdc.gov/poxvirus/monkeypox/clinicians/clinical-recognition.html

8. Huhn GD, Bauer AM, Yorita K, Graham MB, Sejvar J, Likos A, et al. Clinical Characteristics of Human Monkeypox, and Risk Factors for Severe Disease. Clinical Infectious Diseases. 2005 Dec 15;41(12):1742–51.

9. McCollum AM, Damon IK. Human Monkeypox. Clinical Infectious Diseases. 2014 Jan 15;58(2):260–7.

10. Ogoina D, Iroezindu M, James HI, Oladokun R, Yinka-Ogunleye A, Wakama P, et al. Clinical Course and Outcome of Human Monkeypox in Nigeria. Clinical Infectious Diseases. 2020 Nov 5;71(8):e210–4.

11. Weinstein RA, Nalca A, Rimoin AW, Bavari S, Whitehouse CA. Reemergence of Monkeypox: Prevalence, Diagnostics, and Countermeasures. Clinical Infectious Diseases. 2005 Dec 15;41(12):1765–71.

12. Mailhe M, Beaumont AL, Thy M, Le Pluart D, Perrineau S, Houhou-Fidouh N, et al. Clinical characteristics of ambulatory and hospitalised patients with monkeypox virus infection: an observational cohort study. Clinical Microbiology and Infection [Internet]. 2022 Aug 23 [cited 2022 Aug 27]; Available from: https://www.sciencedirect.com/science/article/pii/S1198743X22004281

13. Sukhdeo SS, Aldhaheri K, Lam PW, Walmsley S. A case of human monkeypox in Canada. CMAJ [Internet]. 2022 Jan 1 [cited 2022 Jul 12]; Available from: https://www.cmaj.ca/content/early/2022/07/06/cmaj.220886

14. Reynolds MG, Carroll DS, Karem KL. Factors affecting the likelihood of monkeypox's emergence and spread in the post-smallpox era. Current Opinion in Virology. 2012 Jun 1;2(3):335–43.

15. Costello V, Sowash M, Gaur A, Cardis M, Pasieka H, Wortmann G, et al. Imported Monkeypox from International Traveler, Maryland, USA, 2021. Emerg Infect Dis. 2022 May;28(5):1002–5.

16. Erez N, Achdout H, Milrot E, Schwartz Y, Wiener-Well Y, Paran N, et al. Diagnosis of Imported Monkeypox, Israel, 2018. Emerg Infect Dis. 2019 May;25(5):980–3.

17. Yinka-Ogunleye A, Aruna O, Ogoina D, Aworabhi N, Eteng W, Badaru S, et al. Reemergence of Human Monkeypox in Nigeria, 2017. Emerg Infect Dis. 2018 Jun;24(6):1149–51.

18. Furth AMT van, Kuip M van der, Els AL van, Fievez LC, Rijckevorsel GG van, Ouden A van den, et al. Paediatric monkeypox patient with unknown source of infection, the Netherlands, June 2022. Eurosurveillance. 2022 Jul 21;27(29):2200552.

19. Hammerschlag Y, MacLeod G, Papadakis G, Sanchez AA, Druce J, Taiaroa G, et al. Monkeypox infection presenting as genital rash, Australia, May 2022. Eurosurveillance. 2022 Jun 2;27(22):2200411.

20. Monkeypox: background information [Internet]. GOV.UK. [cited 2022 Jul 12]. Available from: https://www.gov.uk/guidance/monkeypox

21. Mandja BAM, Gonzalez JP. Unveiling the Arcane of an Elusive Virus from the Heart of the African Continent: The Monkeypox. In: Ahmad SI, editor. Human Viruses: Diseases, Treatments and Vaccines : The New Insights [Internet]. Cham: Springer International Publishing; 2021 [cited 2022 Jul 12]. p. 477–99. Available from: https://doi.org/10.1007/978-3-030-71165-8_22

22. Ajmera KM, Goyal L, Pandit T, Pandit R. Monkeypox – An emerging pandemic. IDCases. 2022 Jan 1;29:e01587.

23. Weaver JR, Isaacs SN. Monkeypox virus and insights into its immunomodulatory proteins. Immunological Reviews. 2008;225(1):96–113.

24. Noe S, Zange S, Seilmaier M, Antwerpen MH, Fenzl T, Schneider J, et al. Clinical and virological features of first human monkeypox cases in Germany. Infection. 2022 Jul 11;

25. de Sousa D, Patrocínio J, Frade J, Correia C, Borges-Costa J, Filipe P. Human monkeypox coinfection with acute HIV: an exuberant presentation. Int J STD AIDS. 2022 Jul 13;09564624221114998.

26. Khan S, Razi S, Rao B. It's Here, Monkeypox: A Case Report. JAAD Case Reports. 2022;

27. Heskin J, Belfield A, Milne C, Brown N, Walters Y, Scott C, et al. Transmission of monkeypox virus through sexual contact – A novel route of infection. Journal of Infection [Internet]. 2022 May 31 [cited 2022 Jul 12];0(0). Available from: https://www.journalofinfection.com/article/S0163-4453(22)00335-8/fulltext#relatedArticles

28. Nolasco S, Vitale F, Geremia A, Tramuto F, Maida CM, Sciuto A, et al. First case of monkeypox virus, SARS-CoV-2 and HIV co-infection. Journal of Infection [Internet]. 2022 Aug 19 [cited 2022 Aug 27];0(0). Available from: https://www.journalofinfection.com/article/S0163-4453(22)00479-0/fulltext

29. Orviz E, Negredo A, Ayerdi O, Vázquez A, Muñoz-Gomez A, Monzón S, et al. Monkeypox outbreak in Madrid (Spain): clinical and virological aspects. Journal of Infection [Internet]. 2022 Jul 10 [cited 2022 Jul 19]; Available from: https://www.sciencedirect.com/science/article/pii/S0163445322004157

30. Monkeypox in Nigeria: a case report of re-emerged disease outbreak. [Internet]. 2018 Mar 20 [cited 2022 Jul 13];Volume 6(Issue 2). Available from: https://medcraveonline.com/JMEN/JMEN-06-00193.pdf

31. de Nicolas-Ruanes B, Vivancos M j., Azcarraga-Llobet C, Moreno A m., Rodriguez-Dominguez M, Berna-Rico E d., et al. Monkeypox virus case with maculopapular exanthem and proctitis during the Spanish outbreak in 2022. Journal of the European Academy of Dermatology and Venereology [Internet]. [cited 2022 Jul 13]. Available from: https://onlinelibrary.wiley.com/doi/abs/10.1111/jdv.18300

32. Kreuter A, Grossmann M, Müller VL, Michalowitz AL, Oellig F, Silling S, et al. Monkeypox infection with concomitant penile lymphoedema. Journal of the European Academy of Dermatology and Venereology [Internet]. [cited 2022 Aug 27]. Available from: https://onlinelibrary.wiley.com/doi/abs/10.1111/jdv.18502

33. Paparizos V, Nicolaidou E, Tryfinopoulou K, Papa A, Rigopoulos D, Tsiodras S, et al. Monkeypox virus infection: First reported case in Greece in a patient with a genital

rash. Journal of the European Academy of Dermatology and Venereology [Internet]. [cited 2022 Aug 27]. Available from: https://onlinelibrary.wiley.com/doi/abs/10.1111/jdv.18521

34. Kalthan E, Tenguere J, Ndjapou SG, Koyazengbe TA, Mbomba J, Marada RM, et al. Investigation of an outbreak of monkeypox in an area occupied by armed groups, Central African Republic. Médecine et Maladies Infectieuses. 2018 Jun 1;48(4):263–8.

35. Morand A, Delaigue S, Morand J. Panorama des poxvirus : émergence du monkeypox. Médecine et Santé Tropicales. 2017 Jan 1;27(1):29–39.

36. Minhaj FS. Monkeypox Outbreak — Nine States, May 2022. MMWR Morb Mortal Wkly Rep [Internet]. 2022 [cited 2022 Jul 12];71. Available from: https://www.cdc.gov/mmwr/volumes/71/wr/mm7123e1.htm

37. Basgoz N, Brown CM, Smole SC, Madoff LC, Biddinger PD, Baugh JJ, et al. Case 24-2022: A 31-Year-Old Man with Perianal and Penile Ulcers, Rectal Pain, and Rash. N Engl J Med [Internet]. 2022 Jun 15 [cited 2022 Jul 12]; Available from: https://www.nejm.org/doi/10.1056/NEJMcpc2201244

38. Damon IK, Roth CE, Chowdhary V. Discovery of Monkeypox in Sudan. New England Journal of Medicine. 2006 Aug 31;355(9):962–3.

39. Patrocinio-Jesus R, Peruzzu F. Monkeypox Genital Lesions. N Engl J Med. 2022 Jul 7;387(1):66–66.

40. Reed KD, Melski JW, Graham MB, Regnery RL, Sotir MJ, Wegner MV, et al. The Detection of Monkeypox in Humans in the Western Hemisphere. New England Journal of Medicine. 2004 Jan 22;350(4):342–50.

41. Meaney-Delman DM, Galang RR, Petersen BW, Jamieson DJ. A Primer on Monkeypox Virus for Obstetrician–Gynecologists: Diagnosis, Prevention, and Treatment. Obstetrics & Gynecology. 2022 May 5;10.1097/AOG.0000000000004909.

42. Nakoune E, Lampaert E, Ndjapou SG, Janssens C, Zuniga I, Van Herp M, et al. A Nosocomial Outbreak of Human Monkeypox in the Central African Republic. Open Forum Infectious Diseases. 2017 Oct 1;4(4):ofx168.

43. Carannante N, Tiberio C, Bellopede R, Liguori M, Di Martino F, Maturo N, et al. Monkeypox Clinical Features and Differential Diagnosis: First Case in Campania Region. Pathogens. 2022 Aug;11(8):869.

44. Reed KD. Monkeypox Virus Infections. In: Tabor E, editor. Perspectives in Medical Virology [Internet]. Elsevier; 2006 [cited 2022 Jul 12]. p. 149–60. (Emerging Viruses in Human Populations; vol. 16). Available from: https://www.sciencedirect.com/science/article/pii/S0168706906160073

45. Ogoina D, Izibewule JH, Ogunleye A, Ederiane E, Anebonam U, Neni A, et al. The 2017 human monkeypox outbreak in Nigeria—Report of outbreak experience and response in the Niger Delta University Teaching Hospital, Bayelsa State, Nigeria. PLOS ONE. 2019 Apr 17;14(4):e0214229.

46. Koenig KL, Beÿ CK, Marty AM. Monkeypox 2022: A Primer and Identify-Isolate-Inform (3I) Tool for Emergency Medical Services Professionals. Prehospital and Disaster Medicine. 2022 Aug 4;1–6.

47. Moore DA. Monkeypox Rises Around the World as CDC and WHO Respond [Internet]. QnotesCarolinas.com. 2022 [cited 2022 Jul 13]. Available from: http://qnotescarolinas.com/cdc-and-who-responding-to-monkey-pox-cases-around-the-world-and-in-the-u-s/

48. Ligon BL. Monkeypox: A review of the history and emergence in the Western hemisphere. Seminars in Pediatric Infectious Diseases. 2004 Oct 1;15(4):280–7.

49. Brito Caldeira M, Fernandes C. Cutaneous Lesions From Monkeypox Infection. Sexually Transmitted Diseases. 2022 Sep;49(9):595.

50. Zlámal M, Bartovská Z, Burantová A, Zákoucká H, Jiřincová H, Chmel M, et al. Monkeypox and herpes simplex virus type 2 coinfection: Case report of perianal lesions in HIV positive patient. Sexually Transmitted Diseases. :10.1097/OLQ.0000000000001694.

51. Learned LA, Reynolds MG, Wassa DW, Li Y, Olson VA, Karem K, et al. EXTENDED INTERHUMAN TRANSMISSION OF MONKEYPOX IN A HOSPITAL COMMUNITY IN THE REPUBLIC OF THE CONGO, 2003. The American Journal of Tropical Medicine and Hygiene. 2005 Aug 1;73(2):428–34.

52. McCollum AM, Nakazawa Y, Ndongala GM, Pukuta E, Karhemere S, Lushima RS, et al. Human Monkeypox in the Kivus, a Conflict Region of the Democratic Republic of the Congo. Am J Trop Med Hyg. 2015 Oct 7;93(4):718–21.

53. Reynolds MG, Emerson GL, Pukuta E, Karhemere S, Muyembe JJ, Bikindou A, et al. Detection of Human Monkeypox in the Republic of the Congo Following Intensive Community Education. Am J Trop Med Hyg. 2013 May 1;88(5):982–5.

54. Brundu M, Marinello S, Scaglione V, Ferrari A, Franchin E, Mazzitelli M, et al. The first case of monkeypox virus and acute HIV infection: Should we consider monkeypox a new possible sexually transmitted infection? The Journal of Dermatology [Internet]. [cited 2022 Aug 27]. Available from: https://onlinelibrary.wiley.com/doi/abs/10.1111/1346-8138.16556

55. Sejvar JJ, Chowdary Y, Schomogyi M, Stevens J, Patel J, Karem K, et al. Human Monkeypox Infection: A Family Cluster in the Midwestern United States. The Journal of Infectious Diseases. 2004 Nov 15;190(10):1833–40.

56. Berthet N, Nakouné E, Whist E, Selekon B, Burguière AM, Manuguerra JC, et al. Maculopapular lesions in the Central African Republic. The Lancet. 2011 Oct 8;378(9799):1354.

57. Seang S, Burrel S, Todesco E, Leducq V, Monsel G, Pluart DL, et al. Evidence of human-to-dog transmission of monkeypox virus. The Lancet. 2022 Aug 27;400(10353):658–9.

58. Tarín-Vicente EJ, Alemany A, Agud-Dios M, Ubals M, Suñer C, Antón A, et al. Clinical presentation and virological assessment of confirmed human monkeypox virus cases in Spain: a prospective observational cohort study. The Lancet [Internet]. 2022 Aug 8 [cited 2022 Aug 22]; Available from: https://www.sciencedirect.com/science/article/pii/S0140673622014362

59. Adler H, Gould S, Hine P, Snell LB, Wong W, Houlihan CF, et al. Clinical features and management of human monkeypox: a retrospective observational study in the UK. The Lancet Infectious Diseases [Internet]. 2022 May 24 [cited 2022 Jul 12];0(0). Available from: https://www.thelancet.com/journals/laninf/article/PIIS1473-3099%2822%2900228-6/fulltext#back-bib32

60. Girometti N, Byrne R, Bracchi M, Heskin J, McOwan A, Tittle V, et al. Demographic and clinical characteristics of confirmed human monkeypox virus cases in individuals attending a sexual health centre in London, UK: an observational analysis. The Lancet Infectious Diseases [Internet]. 2022 Jul 1 [cited 2022 Jul 12];0(0). Available from: https://www.thelancet.com/journals/laninf/article/PIIS1473-3099(22)00411-X/fulltext

61. Giulio DBD, Eckburg PB. Human monkeypox: an emerging zoonosis. The Lancet Infectious Diseases. 2004 Jan 1;4(1):15–25.

62. Ortiz-Martínez Y, Rodríguez-Morales AJ, Franco-Paredes C, Chastain DB, Gharamti AA, Vargas Barahona L, et al. Monkeypox – a description of the clinical progression of skin lesions: a case report from Colorado, USA. Therapeutic Advances in Infection. 2022 Jan 1;9:20499361221117730.

63. Mileto D, Riva A, Cutrera M, Moschese D, Mancon A, Meroni L, et al. New challenges in human monkeypox outside Africa: A review and case report from Italy. Travel Medicine and Infectious Disease. 2022 Sep 1;49:102386.

64. Moschese D, Giacomelli A, Beltrami M, Pozza G, Mileto D, Reato S, et al. Hospitalisation for monkeypox in Milan, Italy. Travel Medicine and Infectious Disease. 2022 Sep 1;49:102417.

65. Oprea C, Ianache I, Piscu S, Tardei G, Nica M, Ceausu E, et al. First report of monkeypox in a patient living with HIV from Romania. Travel Medicine and Infectious Disease. 2022 Sep 1;49:102395.

66. Quattri E, Avallone G, Maronese CA, Cusini M, Carrera CG, Marzano AV, et al. Unilesional monkeypox: A report of two cases from Italy. Travel Medicine and Infectious Disease. 2022 Sep 1;49:102424.

67. Vallée A, Farfour E, Zucman D. Monkeypox virus: A novel sexually transmitted disease? A case report from France. Travel Medicine and Infectious Disease. 2022 Sep 1;49:102394.

68. Reynolds MG, Damon IK. Outbreaks of human monkeypox after cessation of smallpox vaccination. Trends in Microbiology. 2012 Feb 1;20(2):80–7.

69. Brites C, Deminco F, Sá MS, Brito JT, Luz E, Stocker A. The First Two Cases of Monkeypox Infection in MSM in Bahia, Brazil, and Viral Sequencing. Viruses. 2022 Sep;14(9):1841.

70. Reynolds MG, McCollum AM, Nguete B, Shongo Lushima R, Petersen BW. Improving the Care and Treatment of Monkeypox Patients in Low-Resource Settings: Applying Evidence from Contemporary Biomedical and Smallpox Biodefense Research. Viruses. 2017 Dec 12;9(12):380.

71. Petersen E, Kantele A, Koopmans M, Asogun D, Yinka-Ogunleye A, Ihekweazu C, et al. Human Monkeypox.

72. A case of human monkeypox in Canada | CMAJ [Internet]. [cited 2022 Aug 22]. Available from: https://www.cmaj.ca/content/194/29/E1031/tab-article-info

73. What clinicians need to know about monkeypox in the United States and other countries [Internet]. [cited 2022 Jul 12]. Available from: https://stacks.cdc.gov/view/cdc/117869

74. Webinar June 29, 2022 - Monkeypox: Updates about Clinical Diagnosis and Treatment [Internet]. 2022 [cited 2022 Jul 13]. Available from: https://emergency.cdc.gov/coca/calls/2022/callinfo_062922.asp

75. Coinfection of Monkeypox, COVID-19 and Syphilis in a PrEP using MSM: A case report from Barcelona. [Internet]. 2022 [cited 2022 Aug 27]. Available from: https://www.researchsquare.com

Supplementary Note 2: URLs of non-MPXV images from Danderm, DermIS and HDA.

https://danderm-pdv.is.kkh.dk/atlas/pics/2/2-01.jpg
https://danderm-pdv.is.kkh.dk/atlas/pics/2/2-02.jpg
https://danderm-pdv.is.kkh.dk/atlas/pics/2/2-03.jpg
https://danderm-pdv.is.kkh.dk/atlas/pics/2/2-04.jpg
https://danderm-pdv.is.kkh.dk/atlas/pics/2/2-05.jpg
https://danderm-pdv.is.kkh.dk/atlas/pics/2/2-06_S.jpg
https://danderm-pdv.is.kkh.dk/atlas/pics/2/2-07.jpg
https://danderm-pdv.is.kkh.dk/atlas/pics/2/2-08.jpg
https://danderm-pdv.is.kkh.dk/atlas/pics/2/2-09.jpg
https://danderm-pdv.is.kkh.dk/atlas/pics/2/2-10.jpg
https://danderm-pdv.is.kkh.dk/atlas/pics/2/2-10-2.jpg
https://danderm-pdv.is.kkh.dk/atlas/pics/2/2-10-3.jpg
https://danderm-pdv.is.kkh.dk/atlas/pics/2/2-10-4.jpg
https://danderm-pdv.is.kkh.dk/atlas/pics/2/2-10-5.jpg
https://danderm-pdv.is.kkh.dk/atlas/pics/2/2-10-6.jpg
https://danderm-pdv.is.kkh.dk/atlas/pics/2/2-10-7.jpg
https://danderm-pdv.is.kkh.dk/atlas/pics/2/2-10-8.jpg
https://danderm-pdv.is.kkh.dk/atlas/pics/2/2-10-9.jpg
https://danderm-pdv.is.kkh.dk/atlas/pics/2/2-10-10.jpg
https://danderm-pdv.is.kkh.dk/atlas/pics/2/2-10-11.jpg
https://danderm-pdv.is.kkh.dk/atlas/pics/2/2-10-12.jpg
https://danderm-pdv.is.kkh.dk/atlas/pics/2/2-10-13.jpg
https://danderm-pdv.is.kkh.dk/atlas/pics/2/2-11.jpg
https://danderm-pdv.is.kkh.dk/atlas/pics/2/2-13-1.jpg
https://danderm-pdv.is.kkh.dk/atlas/pics/2/2-13-2.jpg
https://danderm-pdv.is.kkh.dk/atlas/pics/2/2-13-3.jpg
https://danderm-pdv.is.kkh.dk/atlas/pics/2/2-13-4.jpg
https://danderm-pdv.is.kkh.dk/atlas/pics/2/2-13-5.jpg
https://danderm-pdv.is.kkh.dk/atlas/pics/2/2-13-6.jpg
https://danderm-pdv.is.kkh.dk/atlas/pics/2/2-13-7.jpg
https://danderm-pdv.is.kkh.dk/atlas/pics/2/2-13-8.jpg
https://danderm-pdv.is.kkh.dk/atlas/pics/2/2-13-9.jpg
https://danderm-pdv.is.kkh.dk/atlas/pics/2/2-13-10.jpg
https://danderm-pdv.is.kkh.dk/atlas/pics/2/2-13-11.jpg
https://danderm-pdv.is.kkh.dk/atlas/pics/2/2-14.jpg
https://danderm-pdv.is.kkh.dk/atlas/pics/2/2-14-1.jpg
https://danderm-pdv.is.kkh.dk/atlas/pics/2/2-14-2.jpg
https://danderm-pdv.is.kkh.dk/atlas/pics/2/2-14-3.jpg
https://danderm-pdv.is.kkh.dk/atlas/pics/2/2-15.jpg
https://danderm-pdv.is.kkh.dk/atlas/pics/2/2-16.jpg
https://danderm-pdv.is.kkh.dk/atlas/pics/2/2-17.jpg
https://danderm-pdv.is.kkh.dk/atlas/pics/2/2-17-1.jpg
https://danderm-pdv.is.kkh.dk/atlas/pics/2/2-18.jpg
https://danderm-pdv.is.kkh.dk/atlas/pics/2/2-19.jpg

https://danderm-pdv.is.kkh.dk/atlas/pics/2/2-19-1.jpg
https://danderm-pdv.is.kkh.dk/atlas/pics/2/2-20.jpg
https://danderm-pdv.is.kkh.dk/atlas/pics/2/2-21.jpg
https://danderm-pdv.is.kkh.dk/atlas/pics/2/2-22.jpg
https://danderm-pdv.is.kkh.dk/atlas/pics/2/2-22-1.jpg
https://danderm-pdv.is.kkh.dk/atlas/pics/2/2-22-2.jpg
https://danderm-pdv.is.kkh.dk/atlas/pics/2/2-22-3.jpg
https://danderm-pdv.is.kkh.dk/atlas/pics/2/2-23.jpg
https://danderm-pdv.is.kkh.dk/atlas/pics/2/2-23-1.jpg
https://danderm-pdv.is.kkh.dk/atlas/pics/2/2-24.jpg
https://danderm-pdv.is.kkh.dk/atlas/pics/2/2-24-1.jpg
https://danderm-pdv.is.kkh.dk/atlas/pics/2/2-24-2.jpg
https://danderm-pdv.is.kkh.dk/atlas/pics/2/2-25.jpg
https://danderm-pdv.is.kkh.dk/atlas/pics/2/2-26.jpg
https://danderm-pdv.is.kkh.dk/atlas/pics/2/2-26-1.jpg
https://danderm-pdv.is.kkh.dk/atlas/pics/2/2-26-2.jpg
https://danderm-pdv.is.kkh.dk/atlas/pics/2/2-27.jpg
https://danderm-pdv.is.kkh.dk/atlas/pics/2/2-27-1.jpg
https://danderm-pdv.is.kkh.dk/atlas/pics/2/2-27-2.jpg
https://danderm-pdv.is.kkh.dk/atlas/pics/2/2-27-3.jpg
https://danderm-pdv.is.kkh.dk/atlas/pics/2/2-27-3-1.jpg
https://danderm-pdv.is.kkh.dk/atlas/pics/2/2-27-3-2.jpg
https://danderm-pdv.is.kkh.dk/atlas/pics/2/2-27-3-3.jpg
https://danderm-pdv.is.kkh.dk/atlas/pics/2/2-27-3-4.jpg
https://danderm-pdv.is.kkh.dk/atlas/pics/2/2-27-3-5.jpg
https://danderm-pdv.is.kkh.dk/atlas/pics/2/2-27-3-6.jpg
https://danderm-pdv.is.kkh.dk/atlas/pics/2/2-27-3-7.jpg
https://danderm-pdv.is.kkh.dk/atlas/pics/2/2-27-3-8.jpg
https://danderm-pdv.is.kkh.dk/atlas/pics/2/2-27-3-9.jpg
https://danderm-pdv.is.kkh.dk/atlas/pics/2/2-27-3-10.jpg
https://danderm-pdv.is.kkh.dk/atlas/pics/2/2-27-3-11.jpg
https://danderm-pdv.is.kkh.dk/atlas/pics/2/2-27-3-12.jpg
https://danderm-pdv.is.kkh.dk/atlas/pics/2/2-27-3-13.jpg
https://danderm-pdv.is.kkh.dk/atlas/pics/2/2-27-3-14.jpg
https://danderm-pdv.is.kkh.dk/atlas/pics/2/2-27-4.jpg
https://danderm-pdv.is.kkh.dk/atlas/pics/2/2-27-5.jpg
https://danderm-pdv.is.kkh.dk/atlas/pics/2/2-27-8.jpg
https://danderm-pdv.is.kkh.dk/atlas/pics/2/2-27-9.jpg
https://danderm-pdv.is.kkh.dk/atlas/pics/2/2-27-10.jpg
https://danderm-pdv.is.kkh.dk/atlas/pics/2/2-27-11.jpg
https://danderm-pdv.is.kkh.dk/atlas/pics/2/2-27-12.jpg
https://danderm-pdv.is.kkh.dk/atlas/pics/2/2-28.jpg
https://danderm-pdv.is.kkh.dk/atlas/pics/2/2-29.jpg
https://danderm-pdv.is.kkh.dk/atlas/pics/2/2-30.jpg
https://danderm-pdv.is.kkh.dk/atlas/pics/2/2-30-1.jpg
https://danderm-pdv.is.kkh.dk/atlas/pics/2/2-30-2.jpg
https://danderm-pdv.is.kkh.dk/atlas/pics/2/2-31.jpg
https://danderm-pdv.is.kkh.dk/atlas/pics/2/2-32.jpg

https://danderm-pdv.is.kkh.dk/atlas/pics/2/2-34.jpg
https://danderm-pdv.is.kkh.dk/atlas/pics/2/2-35.jpg
https://danderm-pdv.is.kkh.dk/atlas/pics/2/2-36.jpg
https://danderm-pdv.is.kkh.dk/atlas/pics/2/2-37.jpg
https://danderm-pdv.is.kkh.dk/atlas/pics/2/2-38.jpg
https://danderm-pdv.is.kkh.dk/atlas/pics/2/2-39.jpg
https://danderm-pdv.is.kkh.dk/atlas/pics/2/2-40.jpg
https://danderm-pdv.is.kkh.dk/atlas/pics/2/2-41.jpg
https://danderm-pdv.is.kkh.dk/atlas/pics/2/2-42.jpg
https://danderm-pdv.is.kkh.dk/atlas/pics/2/2-43.jpg
https://danderm-pdv.is.kkh.dk/atlas/pics/2/2-43-1.jpg
https://danderm-pdv.is.kkh.dk/atlas/pics/2/2-43-2.jpg
https://danderm-pdv.is.kkh.dk/atlas/pics/2/2-43-3.jpg
https://danderm-pdv.is.kkh.dk/atlas/pics/2/2-43-5.jpg
https://danderm-pdv.is.kkh.dk/atlas/pics/2/2-43-6.jpg
https://danderm-pdv.is.kkh.dk/atlas/pics/2/2-44_S.jpg
https://danderm-pdv.is.kkh.dk/atlas/pics/2/2-45.jpg
https://danderm-pdv.is.kkh.dk/atlas/pics/2/2-46.jpg
https://danderm-pdv.is.kkh.dk/atlas/pics/2/2-46-1.jpg
https://danderm-pdv.is.kkh.dk/atlas/pics/2/2-46-2.jpg
https://danderm-pdv.is.kkh.dk/atlas/pics/2/2-46-3.jpg
https://danderm-pdv.is.kkh.dk/atlas/pics/2/2-46-4.jpg
https://danderm-pdv.is.kkh.dk/atlas/pics/2/2-46-5.jpg
https://danderm-pdv.is.kkh.dk/atlas/pics/2/2-46-6.jpg
https://danderm-pdv.is.kkh.dk/atlas/pics/2/2-46-7.jpg
https://danderm-pdv.is.kkh.dk/atlas/pics/2/2-46-9.jpg
https://danderm-pdv.is.kkh.dk/atlas/pics/2/2-46-10.jpg
https://danderm-pdv.is.kkh.dk/atlas/pics/2/2-47.jpg
https://danderm-pdv.is.kkh.dk/atlas/pics/2/2-48.jpg
https://danderm-pdv.is.kkh.dk/atlas/pics/2/2-49.jpg
https://danderm-pdv.is.kkh.dk/atlas/pics/2/2-49-1.jpg
https://danderm-pdv.is.kkh.dk/atlas/pics/2/2-50.jpg
https://danderm-pdv.is.kkh.dk/atlas/pics/2/2-51.jpg
https://danderm-pdv.is.kkh.dk/atlas/pics/2/2-52.jpg
https://danderm-pdv.is.kkh.dk/atlas/pics/2/2-53.jpg
https://danderm-pdv.is.kkh.dk/atlas/pics/2/2-54.jpg
https://danderm-pdv.is.kkh.dk/atlas/pics/2/2-54-1.jpg
https://danderm-pdv.is.kkh.dk/atlas/pics/2/2-54-2.jpg
https://danderm-pdv.is.kkh.dk/atlas/pics/2/2-54-3.jpg
https://danderm-pdv.is.kkh.dk/atlas/pics/2/2-54-4.jpg
https://danderm-pdv.is.kkh.dk/atlas/pics/2/2-54-5.jpg
https://danderm-pdv.is.kkh.dk/atlas/pics/2/2-54-6.jpg
https://danderm-pdv.is.kkh.dk/atlas/pics/2/2-54-7.jpg
https://danderm-pdv.is.kkh.dk/atlas/pics/2/2-55.jpg
https://danderm-pdv.is.kkh.dk/atlas/pics/2/2-56_S.jpg
https://danderm-pdv.is.kkh.dk/atlas/pics/2/2-56-1.jpg
https://danderm-pdv.is.kkh.dk/atlas/pics/2/2-56-2.jpg
https://danderm-pdv.is.kkh.dk/atlas/pics/2/2-56-3.jpg

https://danderm-pdv.is.kkh.dk/atlas/pics/2/2-56-4.jpg
https://danderm-pdv.is.kkh.dk/atlas/pics/2/2-57.jpg
https://danderm-pdv.is.kkh.dk/atlas/pics/2/2-58.jpg
https://danderm-pdv.is.kkh.dk/atlas/pics/2/2-58-2.jpg
https://danderm-pdv.is.kkh.dk/atlas/pics/2/2-58-3.jpg
https://danderm-pdv.is.kkh.dk/atlas/pics/2/2-58-4.jpg
https://danderm-pdv.is.kkh.dk/atlas/pics/2/2-58-5.jpg
https://danderm-pdv.is.kkh.dk/atlas/pics/2/2-58-6.jpg
https://danderm-pdv.is.kkh.dk/atlas/pics/2/2-58-7.jpg
https://danderm-pdv.is.kkh.dk/atlas/pics/2/2-58-8.jpg
https://danderm-pdv.is.kkh.dk/atlas/pics/2/2-58-10.jpg
https://danderm-pdv.is.kkh.dk/atlas/pics/2/2-58-11.jpg
https://danderm-pdv.is.kkh.dk/atlas/pics/2/2-58-12.jpg
https://danderm-pdv.is.kkh.dk/atlas/pics/2/2-58-13.jpg
https://danderm-pdv.is.kkh.dk/atlas/pics/2/2-58-14.jpg
https://danderm-pdv.is.kkh.dk/atlas/pics/2/2-58-15.jpg
https://danderm-pdv.is.kkh.dk/atlas/pics/2/2-58-16.jpg
https://danderm-pdv.is.kkh.dk/atlas/pics/2/2-58-17.jpg
https://danderm-pdv.is.kkh.dk/atlas/pics/2/2-58-18.jpg
https://danderm-pdv.is.kkh.dk/atlas/pics/2/2-58-19.jpg
https://danderm-pdv.is.kkh.dk/atlas/pics/2/2-58-20.jpg
https://danderm-pdv.is.kkh.dk/atlas/pics/2/2-58-21.jpg
https://danderm-pdv.is.kkh.dk/atlas/pics/2/2-59.jpg
https://danderm-pdv.is.kkh.dk/atlas/pics/2/2-59-1.jpg
https://danderm-pdv.is.kkh.dk/atlas/pics/2/2-59-2.jpg
https://danderm-pdv.is.kkh.dk/atlas/pics/2/2-59-3.jpg
https://danderm-pdv.is.kkh.dk/atlas/pics/2/2-59-4.jpg
https://danderm-pdv.is.kkh.dk/atlas/pics/2/2-59-5.jpg
https://danderm-pdv.is.kkh.dk/atlas/pics/2/2-59-6.jpg
https://danderm-pdv.is.kkh.dk/atlas/pics/2/2-61_S.jpg
https://danderm-pdv.is.kkh.dk/atlas/pics/2/2-62.jpg
https://danderm-pdv.is.kkh.dk/atlas/pics/2/2-63.jpg
https://danderm-pdv.is.kkh.dk/atlas/pics/2/2-64.jpg
https://danderm-pdv.is.kkh.dk/atlas/pics/2/2-65.jpg
https://danderm-pdv.is.kkh.dk/atlas/pics/2/2-66_S.jpg
https://danderm-pdv.is.kkh.dk/atlas/pics/2/2-66-1.jpg
https://danderm-pdv.is.kkh.dk/atlas/pics/2/2-66-2.jpg
https://danderm-pdv.is.kkh.dk/atlas/pics/2/2-66-3.jpg
https://danderm-pdv.is.kkh.dk/atlas/pics/2/2-66-4.jpg
https://danderm-pdv.is.kkh.dk/atlas/pics/2/2-66-5.jpg
https://danderm-pdv.is.kkh.dk/atlas/pics/2/2-66-6.jpg
https://danderm-pdv.is.kkh.dk/atlas/pics/2/2-66-7.jpg
https://danderm-pdv.is.kkh.dk/atlas/pics/2/2-66-10.jpg
https://danderm-pdv.is.kkh.dk/atlas/pics/2/2-66-11.jpg
https://danderm-pdv.is.kkh.dk/atlas/pics/2/2-66-12.jpg
https://danderm-pdv.is.kkh.dk/atlas/pics/2/2-66-14.jpg
https://danderm-pdv.is.kkh.dk/atlas/pics/2/2-66-16.jpg
https://danderm-pdv.is.kkh.dk/atlas/pics/2/2-67_S.jpg

https://danderm-pdv.is.kkh.dk/atlas/pics/2/2-67-1.jpg
https://danderm-pdv.is.kkh.dk/atlas/pics/2/2-67-2.jpg
https://danderm-pdv.is.kkh.dk/atlas/pics/2/2-67-3.jpg
https://danderm-pdv.is.kkh.dk/atlas/pics/2/2-67-4.jpg
https://danderm-pdv.is.kkh.dk/atlas/pics/2/2-67-5.jpg
https://danderm-pdv.is.kkh.dk/atlas/pics/2/2-67-6.jpg
https://danderm-pdv.is.kkh.dk/atlas/pics/2/2-68.jpg
https://danderm-pdv.is.kkh.dk/atlas/pics/2/2-69.jpg
https://danderm-pdv.is.kkh.dk/atlas/pics/2/2-69-1.jpg
https://danderm-pdv.is.kkh.dk/atlas/pics/2/2-70_S.jpg
https://danderm-pdv.is.kkh.dk/atlas/pics/2/2-70-1.jpg
https://danderm-pdv.is.kkh.dk/atlas/pics/2/2-70-2.jpg
https://danderm-pdv.is.kkh.dk/atlas/pics/2/2-70-3.jpg
https://danderm-pdv.is.kkh.dk/atlas/pics/2/2-70-4.jpg
https://danderm-pdv.is.kkh.dk/atlas/pics/2/2-70-5.jpg
https://danderm-pdv.is.kkh.dk/atlas/pics/2/2-70-6.jpg
https://danderm-pdv.is.kkh.dk/atlas/pics/2/2-70-7.jpg
https://danderm-pdv.is.kkh.dk/atlas/pics/2/2-70-8.jpg
https://danderm-pdv.is.kkh.dk/atlas/pics/2/2-70-9.jpg
https://danderm-pdv.is.kkh.dk/atlas/pics/2/2-70-10.jpg
https://danderm-pdv.is.kkh.dk/atlas/pics/2/2-70-11.jpg
https://danderm-pdv.is.kkh.dk/atlas/pics/2/2-71.jpg
https://danderm-pdv.is.kkh.dk/atlas/pics/2/2-72.jpg
https://danderm-pdv.is.kkh.dk/atlas/pics/2/2-72-1.jpg
https://danderm-pdv.is.kkh.dk/atlas/pics/2/2-72-2.jpg
https://danderm-pdv.is.kkh.dk/atlas/pics/2/2-72-3.jpg
https://danderm-pdv.is.kkh.dk/atlas/pics/2/2-73_S.jpg
https://danderm-pdv.is.kkh.dk/atlas/pics/2/2-74_S.jpg
https://danderm-pdv.is.kkh.dk/atlas/pics/2/2-75.jpg
https://danderm-pdv.is.kkh.dk/atlas/pics/2/2-76.jpg
https://danderm-pdv.is.kkh.dk/atlas/pics/2/2-77.jpg
https://danderm-pdv.is.kkh.dk/atlas/pics/2/2-77-1.jpg
https://danderm-pdv.is.kkh.dk/atlas/pics/2/2-78.jpg
https://danderm-pdv.is.kkh.dk/atlas/pics/2/2-79.jpg
https://danderm-pdv.is.kkh.dk/atlas/pics/2/2-79-1.jpg
https://danderm-pdv.is.kkh.dk/atlas/pics/2/2-79-2.jpg
https://danderm-pdv.is.kkh.dk/atlas/pics/2/2-80.jpg
https://danderm-pdv.is.kkh.dk/atlas/pics/2/2-81.jpg
https://danderm-pdv.is.kkh.dk/atlas/pics/2/2-82.jpg
https://danderm-pdv.is.kkh.dk/atlas/pics/2/2-83.jpg
https://danderm-pdv.is.kkh.dk/atlas/pics/2/2-84.jpg
https://danderm-pdv.is.kkh.dk/atlas/pics/2/2-85.jpg
https://danderm-pdv.is.kkh.dk/atlas/pics/2/2-85-1.jpg
https://danderm-pdv.is.kkh.dk/atlas/pics/2/2-85-2.jpg
https://danderm-pdv.is.kkh.dk/atlas/pics/2/2-85-3.jpg
https://danderm-pdv.is.kkh.dk/atlas/pics/2/2-86.jpg
https://danderm-pdv.is.kkh.dk/atlas/pics/2/2-87.jpg
https://danderm-pdv.is.kkh.dk/atlas/pics/2/2-87-1.jpg

https://danderm-pdv.is.kkh.dk/atlas/pics/2/2-87-2.jpg
https://danderm-pdv.is.kkh.dk/atlas/pics/2/2-88.jpg
https://danderm-pdv.is.kkh.dk/atlas/pics/2/2-88-1.jpg
https://danderm-pdv.is.kkh.dk/atlas/pics/2/2-88-2.jpg
https://danderm-pdv.is.kkh.dk/atlas/pics/2/2-88-3.jpg
https://danderm-pdv.is.kkh.dk/atlas/pics/2/2-88-4.jpg
https://danderm-pdv.is.kkh.dk/atlas/pics/2/2-88-5.jpg
https://danderm-pdv.is.kkh.dk/atlas/pics/2/2-88-6.jpg
https://danderm-pdv.is.kkh.dk/atlas/pics/2/2-88-7.jpg
https://danderm-pdv.is.kkh.dk/atlas/pics/2/2-88-8.jpg
https://danderm-pdv.is.kkh.dk/atlas/pics/2/2-88-9.jpg
https://danderm-pdv.is.kkh.dk/atlas/pics/2/2-88-10.jpg
https://danderm-pdv.is.kkh.dk/atlas/pics/2/2-89.jpg
https://danderm-pdv.is.kkh.dk/atlas/pics/2/2-90-1.jpg
https://danderm-pdv.is.kkh.dk/atlas/pics/2/2-90-2.jpg
https://danderm-pdv.is.kkh.dk/atlas/pics/2/2-95-3.jpg
https://danderm-pdv.is.kkh.dk/atlas/pics/2/2-95-4.jpg
https://danderm-pdv.is.kkh.dk/atlas/pics/2/2-96.jpg
https://danderm-pdv.is.kkh.dk/atlas/pics/2/2-97.jpg
https://danderm-pdv.is.kkh.dk/atlas/pics/2/2-97-1.jpg
https://danderm-pdv.is.kkh.dk/atlas/pics/2/2-97-2.jpg
https://danderm-pdv.is.kkh.dk/atlas/pics/2/2-97-3.jpg
https://danderm-pdv.is.kkh.dk/atlas/pics/2/2-97-5.jpg
https://danderm-pdv.is.kkh.dk/atlas/pics/2/2-98_S.jpg
https://danderm-pdv.is.kkh.dk/atlas/pics/2/2-99_S.jpg
https://danderm-pdv.is.kkh.dk/atlas/pics/2/2-100_S.jpg
https://danderm-pdv.is.kkh.dk/atlas/pics/2/2-101.jpg
https://danderm-pdv.is.kkh.dk/atlas/pics/2/2-102.jpg
https://danderm-pdv.is.kkh.dk/atlas/pics/2/2-102-1.jpg
https://danderm-pdv.is.kkh.dk/atlas/pics/2/2-103-1.jpg
https://danderm-pdv.is.kkh.dk/atlas/pics/2/2-104.jpg
https://danderm-pdv.is.kkh.dk/atlas/pics/2/2-105.jpg
https://danderm-pdv.is.kkh.dk/atlas/pics/2/2-105-1.jpg
https://danderm-pdv.is.kkh.dk/atlas/pics/2/2-106.jpg
https://danderm-pdv.is.kkh.dk/atlas/pics/2/2-106-1.jpg
https://danderm-pdv.is.kkh.dk/atlas/pics/2/2-106-2.jpg
https://danderm-pdv.is.kkh.dk/atlas/pics/2/2-106-3.jpg
https://danderm-pdv.is.kkh.dk/atlas/pics/2/2-106-4.jpg
https://danderm-pdv.is.kkh.dk/atlas/pics/2/2-106-5.jpg
https://danderm-pdv.is.kkh.dk/atlas/pics/2/2-106-6.jpg
https://danderm-pdv.is.kkh.dk/atlas/pics/2/2-106-7.jpg
https://danderm-pdv.is.kkh.dk/atlas/pics/2/2-106-8.jpg
https://danderm-pdv.is.kkh.dk/atlas/pics/2/2-106-9.jpg
https://danderm-pdv.is.kkh.dk/atlas/pics/2/2-106-10.jpg
https://danderm-pdv.is.kkh.dk/atlas/pics/2/2-106-11.jpg
https://danderm-pdv.is.kkh.dk/atlas/pics/2/2-106-12.jpg
https://danderm-pdv.is.kkh.dk/atlas/pics/2/2-107_S.jpg
https://danderm-pdv.is.kkh.dk/atlas/pics/2/2-108_S.jpg

https://danderm-pdv.is.kkh.dk/atlas/pics/2/2-108-1.jpg
https://danderm-pdv.is.kkh.dk/atlas/pics/2/2-108-1-1.jpg
https://danderm-pdv.is.kkh.dk/atlas/pics/2/2-108-1-2.jpg
https://danderm-pdv.is.kkh.dk/atlas/pics/2/2-108-2.jpg
https://danderm-pdv.is.kkh.dk/atlas/pics/2/2-108-3.jpg
https://danderm-pdv.is.kkh.dk/atlas/pics/2/2-108-4.jpg
https://danderm-pdv.is.kkh.dk/atlas/pics/2/2-108-6.jpg
https://danderm-pdv.is.kkh.dk/atlas/pics/2/2-108-7.jpg
https://danderm-pdv.is.kkh.dk/atlas/pics/2/2-108-8.jpg
https://danderm-pdv.is.kkh.dk/atlas/pics/2/2-108-9.jpg
https://danderm-pdv.is.kkh.dk/atlas/pics/2/2-108-10.jpg
https://danderm-pdv.is.kkh.dk/atlas/pics/2/2-108-11.jpg
https://danderm-pdv.is.kkh.dk/atlas/pics/2/2-108-12.jpg
https://danderm-pdv.is.kkh.dk/atlas/pics/2/2-108-13.jpg
https://danderm-pdv.is.kkh.dk/atlas/pics/2/2-108-14.jpg
https://danderm-pdv.is.kkh.dk/atlas/pics/2/2-108-15.jpg
https://danderm-pdv.is.kkh.dk/atlas/pics/2/2-108-16.jpg
https://danderm-pdv.is.kkh.dk/atlas/pics/2/2-108-17.jpg
https://danderm-pdv.is.kkh.dk/atlas/pics/2/2-108-18.jpg
https://danderm-pdv.is.kkh.dk/atlas/pics/2/2-108-19.jpg
https://danderm-pdv.is.kkh.dk/atlas/pics/2/2-108-20.jpg
https://danderm-pdv.is.kkh.dk/atlas/pics/2/2-108-21.jpg
https://danderm-pdv.is.kkh.dk/atlas/pics/2/2-108-22.jpg
https://danderm-pdv.is.kkh.dk/atlas/pics/2/2-108-23.jpg
https://danderm-pdv.is.kkh.dk/atlas/pics/2/2-108-24.jpg
https://danderm-pdv.is.kkh.dk/atlas/pics/2/2-108-25.jpg
https://danderm-pdv.is.kkh.dk/atlas/pics/2/2-108-26.jpg
https://danderm-pdv.is.kkh.dk/atlas/pics/2/2-108-27.jpg
https://danderm-pdv.is.kkh.dk/atlas/pics/2/2-108-28.jpg
https://danderm-pdv.is.kkh.dk/atlas/pics/2/2-108-29.jpg
https://danderm-pdv.is.kkh.dk/atlas/pics/2/2-108-30.jpg
https://danderm-pdv.is.kkh.dk/atlas/pics/2/2-108-31.jpg
https://danderm-pdv.is.kkh.dk/atlas/pics/2/2-108-32.jpg
https://danderm-pdv.is.kkh.dk/atlas/pics/2/2-108-33.jpg
https://danderm-pdv.is.kkh.dk/atlas/pics/2/2-108-34.jpg
https://danderm-pdv.is.kkh.dk/atlas/pics/2/2-108-35.jpg
https://danderm-pdv.is.kkh.dk/atlas/pics/2/2-108-36.jpg
https://danderm-pdv.is.kkh.dk/atlas/pics/2/2-108-37.jpg
https://danderm-pdv.is.kkh.dk/atlas/pics/2/2-108-38.jpg
https://danderm-pdv.is.kkh.dk/atlas/pics/2/2-108-39.jpg
https://danderm-pdv.is.kkh.dk/atlas/pics/2/2-108-40.jpg
https://danderm-pdv.is.kkh.dk/atlas/pics/2/2-108-41.jpg
https://danderm-pdv.is.kkh.dk/atlas/pics/2/2-108-42.jpg
https://danderm-pdv.is.kkh.dk/atlas/pics/2/2-108-43.jpg
https://danderm-pdv.is.kkh.dk/atlas/pics/2/2-108-44.jpg
https://danderm-pdv.is.kkh.dk/atlas/pics/2/2-108-45.jpg
https://danderm-pdv.is.kkh.dk/atlas/pics/2/2-108-46.jpg
https://danderm-pdv.is.kkh.dk/atlas/pics/2/2-108-47.jpg

https://danderm-pdv.is.kkh.dk/atlas/pics/2/2-108-48.jpg
https://danderm-pdv.is.kkh.dk/atlas/pics/2/2-108-49.jpg
https://danderm-pdv.is.kkh.dk/atlas/pics/2/2-108-50.jpg
https://danderm-pdv.is.kkh.dk/atlas/pics/2/2-108-51.jpg
https://danderm-pdv.is.kkh.dk/atlas/pics/2/2-108-52.jpg
https://danderm-pdv.is.kkh.dk/atlas/pics/2/2-108-53.jpg
https://danderm-pdv.is.kkh.dk/atlas/pics/2/2-108-54.jpg
https://danderm-pdv.is.kkh.dk/atlas/pics/2/2-108-55.jpg
https://danderm-pdv.is.kkh.dk/atlas/pics/2/2-108-56.jpg
https://danderm-pdv.is.kkh.dk/atlas/pics/2/2-108-57.jpg
https://danderm-pdv.is.kkh.dk/atlas/pics/2/2-108-58.jpg
https://danderm-pdv.is.kkh.dk/atlas/pics/2/2-108-59.jpg
https://danderm-pdv.is.kkh.dk/atlas/pics/2/2-108-60.jpg
https://danderm-pdv.is.kkh.dk/atlas/pics/2/2-108-61.jpg
https://danderm-pdv.is.kkh.dk/atlas/pics/2/2-108-62.jpg
https://danderm-pdv.is.kkh.dk/atlas/pics/2/2-109.jpg
https://danderm-pdv.is.kkh.dk/atlas/pics/2/2-110.jpg
https://danderm-pdv.is.kkh.dk/atlas/pics/2/2-110-1.jpg
https://danderm-pdv.is.kkh.dk/atlas/pics/2/2-111.jpg
https://danderm-pdv.is.kkh.dk/atlas/pics/2/2-112.jpg
https://danderm-pdv.is.kkh.dk/atlas/pics/2/2-113.jpg
https://danderm-pdv.is.kkh.dk/atlas/pics/2/2-113-1.jpg
https://danderm-pdv.is.kkh.dk/atlas/pics/2/2-113-2.jpg
https://danderm-pdv.is.kkh.dk/atlas/pics/2/2-113-3.jpg
https://danderm-pdv.is.kkh.dk/atlas/pics/2/2-113-4.jpg
https://danderm-pdv.is.kkh.dk/atlas/pics/2/2-113-5.jpg
https://danderm-pdv.is.kkh.dk/atlas/pics/2/2-113-6.jpg
https://danderm-pdv.is.kkh.dk/atlas/pics/2/2-113-7.jpg
https://danderm-pdv.is.kkh.dk/atlas/pics/2/2-113-8.jpg
https://danderm-pdv.is.kkh.dk/atlas/pics/2/2-114.jpg
https://danderm-pdv.is.kkh.dk/atlas/pics/2/2-114-1.jpg
https://danderm-pdv.is.kkh.dk/atlas/pics/2/2-114-1-1.jpg
https://danderm-pdv.is.kkh.dk/atlas/pics/2/2-114-1-2.jpg
https://danderm-pdv.is.kkh.dk/atlas/pics/2/2-114-1-3.jpg
https://danderm-pdv.is.kkh.dk/atlas/pics/2/2-114-2.jpg
https://danderm-pdv.is.kkh.dk/atlas/pics/2/2-115_S.jpg
https://danderm-pdv.is.kkh.dk/atlas/pics/2/2-116_S.jpg
https://danderm-pdv.is.kkh.dk/atlas/pics/2/2-117_S.jpg
https://danderm-pdv.is.kkh.dk/atlas/pics/2/2-117-1.jpg
https://danderm-pdv.is.kkh.dk/atlas/pics/2/2-117-2.jpg
https://danderm-pdv.is.kkh.dk/atlas/pics/2/2-117-3.jpg
https://danderm-pdv.is.kkh.dk/atlas/pics/2/2-117-4.jpg
https://danderm-pdv.is.kkh.dk/atlas/pics/2/2-117-5.jpg
https://danderm-pdv.is.kkh.dk/atlas/pics/2/2-117-6.jpg
https://danderm-pdv.is.kkh.dk/atlas/pics/2/2-117-7.jpg
https://danderm-pdv.is.kkh.dk/atlas/pics/2/2-117-8.jpg
https://danderm-pdv.is.kkh.dk/atlas/pics/2/2-117-9.jpg
https://danderm-pdv.is.kkh.dk/atlas/pics/2/2-117-10.jpg

https://danderm-pdv.is.kkh.dk/atlas/pics/2/2-117-11.jpg
https://danderm-pdv.is.kkh.dk/atlas/pics/2/2-117-12.jpg
https://danderm-pdv.is.kkh.dk/atlas/pics/2/2-117-13.jpg
https://danderm-pdv.is.kkh.dk/atlas/pics/2/2-117-14.jpg
https://danderm-pdv.is.kkh.dk/atlas/pics/2/2-117-15.jpg
https://danderm-pdv.is.kkh.dk/atlas/pics/2/2-117-16.jpg
https://danderm-pdv.is.kkh.dk/atlas/pics/2/2-117-16a.jpg
https://danderm-pdv.is.kkh.dk/atlas/pics/2/2-117-17.jpg
https://danderm-pdv.is.kkh.dk/atlas/pics/2/2-117-18.jpg
https://danderm-pdv.is.kkh.dk/atlas/pics/2/2-117-18-1.jpg
https://danderm-pdv.is.kkh.dk/atlas/pics/2/2-117-18-2.jpg
https://danderm-pdv.is.kkh.dk/atlas/pics/2/2-117-18-3.jpg
https://danderm-pdv.is.kkh.dk/atlas/pics/2/2-117-18-4.jpg
https://danderm-pdv.is.kkh.dk/atlas/pics/2/2-117-18-5.jpg
https://danderm-pdv.is.kkh.dk/atlas/pics/2/2-117-18-6.jpg
https://danderm-pdv.is.kkh.dk/atlas/pics/2/2-117-18-7.jpg
https://danderm-pdv.is.kkh.dk/atlas/pics/2/2-117-18-8.jpg
https://danderm-pdv.is.kkh.dk/atlas/pics/2/2-117-18-9.jpg
https://danderm-pdv.is.kkh.dk/atlas/pics/2/2-117-18-10.jpg
https://danderm-pdv.is.kkh.dk/atlas/pics/2/2-117-19.jpg
https://danderm-pdv.is.kkh.dk/atlas/pics/2/2-117-19-1.jpg
https://danderm-pdv.is.kkh.dk/atlas/pics/2/2-117-19-2.jpg
https://danderm-pdv.is.kkh.dk/atlas/pics/2/2-117-19-3.jpg
https://danderm-pdv.is.kkh.dk/atlas/pics/2/2-117-19-4.jpg
https://danderm-pdv.is.kkh.dk/atlas/pics/2/2-117-19-5.jpg
https://danderm-pdv.is.kkh.dk/atlas/pics/2/2-117-20.jpg
https://danderm-pdv.is.kkh.dk/atlas/pics/2/2-117-21.jpg
https://danderm-pdv.is.kkh.dk/atlas/pics/2/2-117-22.jpg
https://danderm-pdv.is.kkh.dk/atlas/pics/2/2-118.jpg
https://danderm-pdv.is.kkh.dk/atlas/pics/2/2-119.jpg
https://danderm-pdv.is.kkh.dk/atlas/pics/2/2-120_S.jpg
https://danderm-pdv.is.kkh.dk/atlas/pics/2/2-121.jpg
https://danderm-pdv.is.kkh.dk/atlas/pics/2/2-122.jpg
https://danderm-pdv.is.kkh.dk/atlas/pics/2/2-123.jpg
https://danderm-pdv.is.kkh.dk/atlas/pics/2/2-123-1.jpg
https://danderm-pdv.is.kkh.dk/atlas/pics/2/2-123-2.jpg
https://danderm-pdv.is.kkh.dk/atlas/pics/2/2-124.jpg
https://danderm-pdv.is.kkh.dk/atlas/pics/2/2-125.jpg
https://danderm-pdv.is.kkh.dk/atlas/pics/2/2-126.jpg
https://danderm-pdv.is.kkh.dk/atlas/pics/2/2-127.jpg
https://danderm-pdv.is.kkh.dk/atlas/pics/2/2-128.jpg
https://danderm-pdv.is.kkh.dk/atlas/pics/2/2-129.jpg
https://danderm-pdv.is.kkh.dk/atlas/pics/2/2-130.jpg
https://danderm-pdv.is.kkh.dk/atlas/pics/2/2-131.jpg
https://danderm-pdv.is.kkh.dk/atlas/pics/2/2-131-1.jpg
https://danderm-pdv.is.kkh.dk/atlas/pics/2/2-131-2.jpg
https://danderm-pdv.is.kkh.dk/atlas/pics/2/2-131-3.jpg
https://danderm-pdv.is.kkh.dk/atlas/pics/2/2-132.jpg

https://danderm-pdv.is.kkh.dk/atlas/pics/2/2-133.jpg
https://danderm-pdv.is.kkh.dk/atlas/pics/2/2-134.jpg
https://danderm-pdv.is.kkh.dk/atlas/pics/2/2-135.jpg
https://danderm-pdv.is.kkh.dk/atlas/pics/2/2-136.jpg
https://danderm-pdv.is.kkh.dk/atlas/pics/2/2-137.jpg
https://danderm-pdv.is.kkh.dk/atlas/pics/2/2-139.jpg
https://danderm-pdv.is.kkh.dk/atlas/pics/2/2-140.jpg
https://danderm-pdv.is.kkh.dk/atlas/pics/2/2-141.jpg
https://danderm-pdv.is.kkh.dk/atlas/pics/2/2-142.jpg
https://danderm-pdv.is.kkh.dk/atlas/pics/2/2-143.jpg
https://danderm-pdv.is.kkh.dk/atlas/pics/2/2-144.jpg
https://danderm-pdv.is.kkh.dk/atlas/pics/2/2-146.jpg
https://danderm-pdv.is.kkh.dk/atlas/pics/2/2-147.jpg
https://danderm-pdv.is.kkh.dk/atlas/pics/2/2-148.jpg
https://danderm-pdv.is.kkh.dk/atlas/pics/2/2-149.jpg
https://danderm-pdv.is.kkh.dk/atlas/pics/2/2-150.jpg
https://danderm-pdv.is.kkh.dk/atlas/pics/2/2-151.jpg
https://danderm-pdv.is.kkh.dk/atlas/pics/2/2-152.jpg
https://danderm-pdv.is.kkh.dk/atlas/pics/2/2-153.jpg
https://danderm-pdv.is.kkh.dk/atlas/pics/2/2-154.jpg
https://danderm-pdv.is.kkh.dk/atlas/pics/2/2-155.jpg
https://danderm-pdv.is.kkh.dk/atlas/pics/2/2-156.jpg
https://danderm-pdv.is.kkh.dk/atlas/pics/2/2-157.jpg
https://danderm-pdv.is.kkh.dk/atlas/pics/2/2-158.jpg
https://danderm-pdv.is.kkh.dk/atlas/pics/2/2-159.jpg
https://danderm-pdv.is.kkh.dk/atlas/pics/2/2-160.jpg
https://danderm-pdv.is.kkh.dk/atlas/pics/2/2-161.jpg
https://danderm-pdv.is.kkh.dk/atlas/pics/2/2-162.jpg
https://danderm-pdv.is.kkh.dk/atlas/pics/2/2-163.jpg
https://danderm-pdv.is.kkh.dk/atlas/pics/3/3-1-slor.jpg
https://danderm-pdv.is.kkh.dk/atlas/pics/3/3-2.jpg
https://danderm-pdv.is.kkh.dk/atlas/pics/3/3-3.jpg
https://danderm-pdv.is.kkh.dk/atlas/pics/3/3-3-1.jpg
https://danderm-pdv.is.kkh.dk/atlas/pics/3/3-3-2.jpg
https://danderm-pdv.is.kkh.dk/atlas/pics/3/3-3-3.jpg
https://danderm-pdv.is.kkh.dk/atlas/pics/3/3-3-4.jpg
https://danderm-pdv.is.kkh.dk/atlas/pics/3/3-3-5.jpg
https://danderm-pdv.is.kkh.dk/atlas/pics/3/3-3-6.jpg
https://danderm-pdv.is.kkh.dk/atlas/pics/3/3-4.jpg
https://danderm-pdv.is.kkh.dk/atlas/pics/3/3-5.jpg
https://danderm-pdv.is.kkh.dk/atlas/pics/3/3-6.jpg
https://danderm-pdv.is.kkh.dk/atlas/pics/3/3-7.jpg
https://danderm-pdv.is.kkh.dk/atlas/pics/3/3-8-slor.jpg
https://danderm-pdv.is.kkh.dk/atlas/pics/3/3-9.jpg
https://danderm-pdv.is.kkh.dk/atlas/pics/3/3-10-slor.jpg
https://danderm-pdv.is.kkh.dk/atlas/pics/3/3-11.jpg
https://danderm-pdv.is.kkh.dk/atlas/pics/3/3-12.jpg
https://danderm-pdv.is.kkh.dk/atlas/pics/3/3-13.jpg

https://danderm-pdv.is.kkh.dk/atlas/pics/3/3-14.jpg
https://danderm-pdv.is.kkh.dk/atlas/pics/3/3-15.jpg
https://danderm-pdv.is.kkh.dk/atlas/pics/3/3-16.jpg
https://danderm-pdv.is.kkh.dk/atlas/pics/3/3-17.jpg
https://danderm-pdv.is.kkh.dk/atlas/pics/3/3-18.jpg
https://danderm-pdv.is.kkh.dk/atlas/pics/3/3-19.jpg
https://danderm-pdv.is.kkh.dk/atlas/pics/3/3-20.jpg
https://danderm-pdv.is.kkh.dk/atlas/pics/3/3-20-1.jpg
https://danderm-pdv.is.kkh.dk/atlas/pics/3/3-21.jpg
https://danderm-pdv.is.kkh.dk/atlas/pics/3/3-22.jpg
https://danderm-pdv.is.kkh.dk/atlas/pics/3/3-23-slor.jpg
https://danderm-pdv.is.kkh.dk/atlas/pics/3/3-23-1.jpg
https://danderm-pdv.is.kkh.dk/atlas/pics/3/3-23-2.jpg
https://danderm-pdv.is.kkh.dk/atlas/pics/3/3-23-3.jpg
https://danderm-pdv.is.kkh.dk/atlas/pics/3/3-24-slor.jpg
https://danderm-pdv.is.kkh.dk/atlas/pics/3/3-24-1.jpg
https://danderm-pdv.is.kkh.dk/atlas/pics/3/3-25.jpg
https://danderm-pdv.is.kkh.dk/atlas/pics/3/3-26.jpg
https://danderm-pdv.is.kkh.dk/atlas/pics/3/3-27.jpg
https://danderm-pdv.is.kkh.dk/atlas/pics/3/3-28.jpg
https://danderm-pdv.is.kkh.dk/atlas/pics/3/3-28-1.jpg
https://danderm-pdv.is.kkh.dk/atlas/pics/3/3-29.jpg
https://danderm-pdv.is.kkh.dk/atlas/pics/3/3-30.jpg
https://danderm-pdv.is.kkh.dk/atlas/pics/3/3-31.jpg
https://danderm-pdv.is.kkh.dk/atlas/pics/3/3-31-1.jpg
https://danderm-pdv.is.kkh.dk/atlas/pics/3/3-31-2.jpg
https://danderm-pdv.is.kkh.dk/atlas/pics/3/3-31-3.jpg
https://danderm-pdv.is.kkh.dk/atlas/pics/3/3-31-4.jpg
https://danderm-pdv.is.kkh.dk/atlas/pics/3/3-31-5.jpg
https://danderm-pdv.is.kkh.dk/atlas/pics/3/3-31-6.jpg
https://danderm-pdv.is.kkh.dk/atlas/pics/3/3-31-7.jpg
https://danderm-pdv.is.kkh.dk/atlas/pics/3/3-31-8.jpg
https://danderm-pdv.is.kkh.dk/atlas/pics/3/3-32.jpg
https://danderm-pdv.is.kkh.dk/atlas/pics/3/3-33.jpg
https://danderm-pdv.is.kkh.dk/atlas/pics/3/3-33-1.jpg
https://danderm-pdv.is.kkh.dk/atlas/pics/3/3-34.jpg
https://danderm-pdv.is.kkh.dk/atlas/pics/3/3-35.jpg
https://danderm-pdv.is.kkh.dk/atlas/pics/3/3-36.jpg
https://danderm-pdv.is.kkh.dk/atlas/pics/3/3-36-1.jpg
https://danderm-pdv.is.kkh.dk/atlas/pics/3/3-37.jpg
https://danderm-pdv.is.kkh.dk/atlas/pics/3/3-38.jpg
https://danderm-pdv.is.kkh.dk/atlas/pics/3/3-39.jpg
https://danderm-pdv.is.kkh.dk/atlas/pics/3/3-39-1.jpg
https://danderm-pdv.is.kkh.dk/atlas/pics/3/3-39-2.jpg
https://danderm-pdv.is.kkh.dk/atlas/pics/3/3-39-3.jpg
https://danderm-pdv.is.kkh.dk/atlas/pics/3/3-39-4.jpg
https://danderm-pdv.is.kkh.dk/atlas/pics/3/3-39-5.jpg
https://danderm-pdv.is.kkh.dk/atlas/pics/3/3-39-6.jpg

https://danderm-pdv.is.kkh.dk/atlas/pics/3/3-40.jpg
https://danderm-pdv.is.kkh.dk/atlas/pics/3/3-41-slor.jpg
https://danderm-pdv.is.kkh.dk/atlas/pics/3/3-42-slor.jpg
https://danderm-pdv.is.kkh.dk/atlas/pics/3/3-42-1.jpg
https://danderm-pdv.is.kkh.dk/atlas/pics/3/3-42-2.jpg
https://danderm-pdv.is.kkh.dk/atlas/pics/3/3-42-3.jpg
https://danderm-pdv.is.kkh.dk/atlas/pics/3/3-42-4.jpg
https://danderm-pdv.is.kkh.dk/atlas/pics/3/3-43-slor.jpg
https://danderm-pdv.is.kkh.dk/atlas/pics/3/3-44.jpg
https://danderm-pdv.is.kkh.dk/atlas/pics/3/3-45.jpg
https://danderm-pdv.is.kkh.dk/atlas/pics/3/3-46-slor.jpg
https://danderm-pdv.is.kkh.dk/atlas/pics/3/3-46-1.jpg
https://danderm-pdv.is.kkh.dk/atlas/pics/3/3-46-1-1.jpg
https://danderm-pdv.is.kkh.dk/atlas/pics/3/3-46-1-2.jpg
https://danderm-pdv.is.kkh.dk/atlas/pics/3/3-46-1-17.jpg
https://danderm-pdv.is.kkh.dk/atlas/pics/3/3-46-1-19 .jpg
https://danderm-pdv.is.kkh.dk/atlas/pics/3/3-46-1-20.jpg
https://danderm-pdv.is.kkh.dk/atlas/pics/3/3-46-1-21.jpg
https://danderm-pdv.is.kkh.dk/atlas/pics/3/3-46-2.jpg
https://danderm-pdv.is.kkh.dk/atlas/pics/3/3-46-3.jpg
https://danderm-pdv.is.kkh.dk/atlas/pics/3/3-46-4.jpg
https://danderm-pdv.is.kkh.dk/atlas/pics/3/3-46-5.jpg
https://danderm-pdv.is.kkh.dk/atlas/pics/3/3-46-6.jpg
https://danderm-pdv.is.kkh.dk/atlas/pics/3/3-46-7.jpg
https://danderm-pdv.is.kkh.dk/atlas/pics/3/3-46-8.jpg
https://danderm-pdv.is.kkh.dk/atlas/pics/3/3-46-9.jpg
https://danderm-pdv.is.kkh.dk/atlas/pics/3/3-46-10.jpg
https://danderm-pdv.is.kkh.dk/atlas/pics/3/3-46-11.jpg
https://danderm-pdv.is.kkh.dk/atlas/pics/3/3-46-12.jpg
https://danderm-pdv.is.kkh.dk/atlas/pics/3/3-46-13.jpg
https://danderm-pdv.is.kkh.dk/atlas/pics/3/3-46-14.jpg
https://danderm-pdv.is.kkh.dk/atlas/pics/3/3-46-15.jpg
https://danderm-pdv.is.kkh.dk/atlas/pics/3/3-46-16.jpg
https://danderm-pdv.is.kkh.dk/atlas/pics/3/3-46-17.jpg
https://danderm-pdv.is.kkh.dk/atlas/pics/3/3-46-18.jpg
https://danderm-pdv.is.kkh.dk/atlas/pics/3/3-46-19.jpg
https://danderm-pdv.is.kkh.dk/atlas/pics/3/3-46-20.jpg
https://danderm-pdv.is.kkh.dk/atlas/pics/3/3-46-21.jpg
https://danderm-pdv.is.kkh.dk/atlas/pics/3/3-47-slor.jpg
https://danderm-pdv.is.kkh.dk/atlas/pics/3/3-47-1.jpg
https://danderm-pdv.is.kkh.dk/atlas/pics/3/3-47-2.jpg
https://danderm-pdv.is.kkh.dk/atlas/pics/3/3-47-3.jpg
https://danderm-pdv.is.kkh.dk/atlas/pics/3/3-47-4.jpg
https://danderm-pdv.is.kkh.dk/atlas/pics/3/3-47-5.jpg
https://danderm-pdv.is.kkh.dk/atlas/pics/3/3-48.jpg
https://danderm-pdv.is.kkh.dk/atlas/pics/3/3-49.jpg
https://danderm-pdv.is.kkh.dk/atlas/pics/3/3-49-1.jpg
https://danderm-pdv.is.kkh.dk/atlas/pics/3/3-49-2.jpg

https://danderm-pdv.is.kkh.dk/atlas/pics/3/3-49-3.jpg
https://danderm-pdv.is.kkh.dk/atlas/pics/3/3-49-4.jpg
https://danderm-pdv.is.kkh.dk/atlas/pics/3/3-49-5.jpg
https://danderm-pdv.is.kkh.dk/atlas/pics/3/3-49-6.jpg
https://danderm-pdv.is.kkh.dk/atlas/pics/3/3-50.jpg
https://danderm-pdv.is.kkh.dk/atlas/pics/3/3-50-1.jpg
https://danderm-pdv.is.kkh.dk/atlas/pics/3/3-50-2.jpg
https://danderm-pdv.is.kkh.dk/atlas/pics/3/3-50-3.jpg
https://danderm-pdv.is.kkh.dk/atlas/pics/3/3-50-4.jpg
https://danderm-pdv.is.kkh.dk/atlas/pics/3/3-50-5.jpg
https://danderm-pdv.is.kkh.dk/atlas/pics/3/3-50-6.jpg
https://danderm-pdv.is.kkh.dk/atlas/pics/3/3-51.jpg
https://danderm-pdv.is.kkh.dk/atlas/pics/3/3-54-7.jpg
https://danderm-pdv.is.kkh.dk/atlas/pics/3/3-54-8.jpg
https://danderm-pdv.is.kkh.dk/atlas/pics/3/3-54-9.jpg
https://danderm-pdv.is.kkh.dk/atlas/pics/3/3-54-10.jpg
https://danderm-pdv.is.kkh.dk/atlas/pics/3/3-54-11.jpg
https://danderm-pdv.is.kkh.dk/atlas/pics/3/3-54-12.jpg
https://danderm-pdv.is.kkh.dk/atlas/pics/3/3-54-13.jpg
https://danderm-pdv.is.kkh.dk/atlas/pics/3/3-55.jpg
https://danderm-pdv.is.kkh.dk/atlas/pics/3/3-56.jpg
https://danderm-pdv.is.kkh.dk/atlas/pics/3/3-56-1.jpg
https://danderm-pdv.is.kkh.dk/atlas/pics/3/3-56-2.jpg
https://danderm-pdv.is.kkh.dk/atlas/pics/3/3-57-slor.jpg
https://danderm-pdv.is.kkh.dk/atlas/pics/3/3-58.jpg
https://danderm-pdv.is.kkh.dk/atlas/pics/3/3-58-1.jpg
https://danderm-pdv.is.kkh.dk/atlas/pics/3/3-59.jpg
https://danderm-pdv.is.kkh.dk/atlas/pics/3/3-60.jpg
https://danderm-pdv.is.kkh.dk/atlas/pics/3/3-60-1.jpg
https://danderm-pdv.is.kkh.dk/atlas/pics/3/3-61.jpg
https://danderm-pdv.is.kkh.dk/atlas/pics/3/3-61-1.jpg
https://danderm-pdv.is.kkh.dk/atlas/pics/3/3-62.jpg
https://danderm-pdv.is.kkh.dk/atlas/pics/3/3-63.jpg
https://danderm-pdv.is.kkh.dk/atlas/pics/3/3-63-1.jpg
https://danderm-pdv.is.kkh.dk/atlas/pics/3/3-63-2.jpg
https://danderm-pdv.is.kkh.dk/atlas/pics/3/3-63-3.jpg
https://danderm-pdv.is.kkh.dk/atlas/pics/3/3-63-4.jpg
https://danderm-pdv.is.kkh.dk/atlas/pics/3/3-63-5.jpg
https://danderm-pdv.is.kkh.dk/atlas/pics/3/3-64.jpg
https://danderm-pdv.is.kkh.dk/atlas/pics/3/3-65.jpg
https://danderm-pdv.is.kkh.dk/atlas/pics/3/3-65-1.jpg
https://danderm-pdv.is.kkh.dk/atlas/pics/3/3-66-slor.jpg
https://danderm-pdv.is.kkh.dk/atlas/pics/3/3-66-1.jpg
https://danderm-pdv.is.kkh.dk/atlas/pics/3/3-66-2.jpg
https://danderm-pdv.is.kkh.dk/atlas/pics/3/3-66-3.jpg
https://danderm-pdv.is.kkh.dk/atlas/pics/3/3-67.jpg
https://danderm-pdv.is.kkh.dk/atlas/pics/3/3-67-1.jpg
https://danderm-pdv.is.kkh.dk/atlas/pics/3/3-67-2.jpg

https://danderm-pdv.is.kkh.dk/atlas/pics/3/3-67-3.jpg
https://danderm-pdv.is.kkh.dk/atlas/pics/3/3-68.jpg
https://danderm-pdv.is.kkh.dk/atlas/pics/3/3-69.jpg
https://danderm-pdv.is.kkh.dk/atlas/pics/3/3-69-1.jpg
https://danderm-pdv.is.kkh.dk/atlas/pics/3/3-69-2.jpg
https://danderm-pdv.is.kkh.dk/atlas/pics/3/3-70.jpg
https://danderm-pdv.is.kkh.dk/atlas/pics/3/3-71.jpg
https://danderm-pdv.is.kkh.dk/atlas/pics/3/3-72.jpg
https://danderm-pdv.is.kkh.dk/atlas/pics/3/3-73.jpg
https://danderm-pdv.is.kkh.dk/atlas/pics/3/3-73-1.jpg
https://danderm-pdv.is.kkh.dk/atlas/pics/3/3-73-2.jpg
https://danderm-pdv.is.kkh.dk/atlas/pics/3/3-73-3.jpg
https://danderm-pdv.is.kkh.dk/atlas/pics/3/3-73-4.jpg
https://danderm-pdv.is.kkh.dk/atlas/pics/3/3-73-5.jpg
https://danderm-pdv.is.kkh.dk/atlas/pics/3/3-73-6.jpg
https://danderm-pdv.is.kkh.dk/atlas/pics/3/3-73-10.jpg
https://danderm-pdv.is.kkh.dk/atlas/pics/3/3-73-11.jpg
https://danderm-pdv.is.kkh.dk/atlas/pics/3/3-73-12.jpg
https://danderm-pdv.is.kkh.dk/atlas/pics/3/3-73-13.jpg
https://danderm-pdv.is.kkh.dk/atlas/pics/3/3-73-14.jpg
https://danderm-pdv.is.kkh.dk/atlas/pics/3/3-73-15.jpg
https://danderm-pdv.is.kkh.dk/atlas/pics/3/3-73-16.jpg
https://danderm-pdv.is.kkh.dk/atlas/pics/3/3-74.jpg
https://danderm-pdv.is.kkh.dk/atlas/pics/3/3-75.jpg
https://danderm-pdv.is.kkh.dk/atlas/pics/3/3-75-1.jpg
https://danderm-pdv.is.kkh.dk/atlas/pics/3/3-75-2.jpg
https://danderm-pdv.is.kkh.dk/atlas/pics/3/3-75-3.jpg
https://danderm-pdv.is.kkh.dk/atlas/pics/3/3-76.jpg
https://danderm-pdv.is.kkh.dk/atlas/pics/3/3-73-16-3 .jpg
https://danderm-pdv.is.kkh.dk/atlas/pics/3/3-73-16-4 .jpg
https://danderm-pdv.is.kkh.dk/atlas/pics/3/3-77.jpg
https://danderm-pdv.is.kkh.dk/atlas/pics/3/3-78.jpg
https://danderm-pdv.is.kkh.dk/atlas/pics/3/3-78-1.jpg
https://danderm-pdv.is.kkh.dk/atlas/pics/3/3-78-2.jpg
https://danderm-pdv.is.kkh.dk/atlas/pics/3/3-79-slor.jpg
https://danderm-pdv.is.kkh.dk/atlas/pics/3/3-80-slor.jpg
https://danderm-pdv.is.kkh.dk/atlas/pics/3/3-81-slor.jpg
https://danderm-pdv.is.kkh.dk/atlas/pics/3/3-82.jpg
https://danderm-pdv.is.kkh.dk/atlas/pics/3/3-82-5.jpg
https://danderm-pdv.is.kkh.dk/atlas/pics/3/3-83.jpg
https://danderm-pdv.is.kkh.dk/atlas/pics/3/3-83-1.jpg
https://danderm-pdv.is.kkh.dk/atlas/pics/3/3-83-2.jpg
https://danderm-pdv.is.kkh.dk/atlas/pics/3/3-83-3.jpg
https://danderm-pdv.is.kkh.dk/atlas/pics/3/3-84.jpg
https://danderm-pdv.is.kkh.dk/atlas/pics/3/3-85.jpg
https://danderm-pdv.is.kkh.dk/atlas/pics/3/3-85-1.jpg
https://danderm-pdv.is.kkh.dk/atlas/pics/3/3-85-2.jpg
https://danderm-pdv.is.kkh.dk/atlas/pics/3/3-85-3.jpg

https://danderm-pdv.is.kkh.dk/atlas/pics/3/3-85-4.jpg
https://danderm-pdv.is.kkh.dk/atlas/pics/3/3-86.jpg
https://danderm-pdv.is.kkh.dk/atlas/pics/3/3-87.jpg
https://danderm-pdv.is.kkh.dk/atlas/pics/3/3-88.jpg
https://danderm-pdv.is.kkh.dk/atlas/pics/3/3-89.jpg
https://danderm-pdv.is.kkh.dk/atlas/pics/3/3-90.jpg
https://danderm-pdv.is.kkh.dk/atlas/pics/3/3-91-1.jpg
https://danderm-pdv.is.kkh.dk/atlas/pics/3/3-92-slor.jpg
https://danderm-pdv.is.kkh.dk/atlas/pics/3/3-92-1.jpg
https://danderm-pdv.is.kkh.dk/atlas/pics/3/3-93.jpg
https://danderm-pdv.is.kkh.dk/atlas/pics/3/3-94.jpg
https://danderm-pdv.is.kkh.dk/atlas/pics/3/3-94-1.jpg
https://danderm-pdv.is.kkh.dk/atlas/pics/3/3-94-1-1.jpg
https://danderm-pdv.is.kkh.dk/atlas/pics/3/3-94-1-2.jpg
https://danderm-pdv.is.kkh.dk/atlas/pics/3/3-94-1-3.jpg
https://danderm-pdv.is.kkh.dk/atlas/pics/3/3-94-1-4.jpg
https://danderm-pdv.is.kkh.dk/atlas/pics/3/3-94-1-5.jpg
https://danderm-pdv.is.kkh.dk/atlas/pics/3/3-94-1-6.jpg
https://danderm-pdv.is.kkh.dk/atlas/pics/3/3-94-1-7.jpg
https://danderm-pdv.is.kkh.dk/atlas/pics/3/3-94-1-8.jpg
https://danderm-pdv.is.kkh.dk/atlas/pics/3/3-94-1-9.jpg
https://danderm-pdv.is.kkh.dk/atlas/pics/3/3-94-1-10.jpg
https://danderm-pdv.is.kkh.dk/atlas/pics/3/3-94-1-11.jpg
https://danderm-pdv.is.kkh.dk/atlas/pics/3/3-94-1-12.jpg
https://danderm-pdv.is.kkh.dk/atlas/pics/3/3-94-2.jpg
https://danderm-pdv.is.kkh.dk/atlas/pics/3/3-94-3.jpg
https://danderm-pdv.is.kkh.dk/atlas/pics/3/3-94-3-1.jpg
https://danderm-pdv.is.kkh.dk/atlas/pics/3/3-94-3-2.jpg
https://danderm-pdv.is.kkh.dk/atlas/pics/3/3-94-3-3.jpg
https://danderm-pdv.is.kkh.dk/atlas/pics/3/3-94-4.jpg
https://danderm-pdv.is.kkh.dk/atlas/pics/3/3-94-5.jpg
https://danderm-pdv.is.kkh.dk/atlas/pics/3/3-94-6.jpg
https://danderm-pdv.is.kkh.dk/atlas/pics/3/3-94-7.jpg
https://danderm-pdv.is.kkh.dk/atlas/pics/3/3-94-8.jpg
https://danderm-pdv.is.kkh.dk/atlas/pics/3/3-95.jpg
https://danderm-pdv.is.kkh.dk/atlas/pics/3/3-96.jpg
https://danderm-pdv.is.kkh.dk/atlas/pics/3/3-96-1.jpg
https://danderm-pdv.is.kkh.dk/atlas/pics/3/3-96-2.jpg
https://danderm-pdv.is.kkh.dk/atlas/pics/3/3-96-3.jpg
https://danderm-pdv.is.kkh.dk/atlas/pics/3/3-96-4.jpg
https://danderm-pdv.is.kkh.dk/atlas/pics/3/3-96-5.jpg
https://danderm-pdv.is.kkh.dk/atlas/pics/3/3-96-6.jpg
https://danderm-pdv.is.kkh.dk/atlas/pics/3/3-96-7.jpg
https://danderm-pdv.is.kkh.dk/atlas/pics/3/3-97.jpg
https://danderm-pdv.is.kkh.dk/atlas/pics/3/3-98.jpg
https://danderm-pdv.is.kkh.dk/atlas/pics/3/3-99.jpg
https://danderm-pdv.is.kkh.dk/atlas/pics/3/3-100.jpg
https://danderm-pdv.is.kkh.dk/atlas/pics/3/3-101.jpg

https://danderm-pdv.is.kkh.dk/atlas/pics/3/3-101-1.jpg
https://danderm-pdv.is.kkh.dk/atlas/pics/3/3-101-2.jpg
https://danderm-pdv.is.kkh.dk/atlas/pics/3/3-101-3.jpg
https://danderm-pdv.is.kkh.dk/atlas/pics/3/3-101-4.jpg
https://danderm-pdv.is.kkh.dk/atlas/pics/3/3-101-5.jpg
https://danderm-pdv.is.kkh.dk/atlas/pics/3/3-101-6.jpg
https://danderm-pdv.is.kkh.dk/atlas/pics/3/3-102.jpg
https://danderm-pdv.is.kkh.dk/atlas/pics/3/3-106.jpg
https://danderm-pdv.is.kkh.dk/atlas/pics/3/3-107.jpg
https://danderm-pdv.is.kkh.dk/atlas/pics/3/3-107-2.jpg
https://danderm-pdv.is.kkh.dk/atlas/pics/3/3-107-1.jpg
https://danderm-pdv.is.kkh.dk/atlas/pics/3/3-108.jpg
https://danderm-pdv.is.kkh.dk/atlas/pics/3/3-108-1.jpg
https://danderm-pdv.is.kkh.dk/atlas/pics/3/3-109.jpg
https://danderm-pdv.is.kkh.dk/atlas/pics/3/3-109-1.jpg
https://danderm-pdv.is.kkh.dk/atlas/pics/3/3-109-2.jpg
https://danderm-pdv.is.kkh.dk/atlas/pics/3/3-109-3.jpg
https://danderm-pdv.is.kkh.dk/atlas/pics/3/3-110-slor.jpg
https://danderm-pdv.is.kkh.dk/atlas/pics/3/3-110-1.jpg
https://danderm-pdv.is.kkh.dk/atlas/pics/3/3-111.jpg
https://danderm-pdv.is.kkh.dk/atlas/pics/3/3-111-1.jpg
https://danderm-pdv.is.kkh.dk/atlas/pics/3/3-111-2.jpg
https://danderm-pdv.is.kkh.dk/atlas/pics/3/3-111-3.jpg
https://danderm-pdv.is.kkh.dk/atlas/pics/3/3-111-4.jpg
https://danderm-pdv.is.kkh.dk/atlas/pics/3/3-111-5 .jpg
https://danderm-pdv.is.kkh.dk/atlas/pics/3/3-112.jpg
https://danderm-pdv.is.kkh.dk/atlas/pics/3/3-112-1.jpg
https://danderm-pdv.is.kkh.dk/atlas/pics/3/3-112-2.jpg
https://danderm-pdv.is.kkh.dk/atlas/pics/3/3-113.jpg
https://danderm-pdv.is.kkh.dk/atlas/pics/3/3-114.jpg
https://danderm-pdv.is.kkh.dk/atlas/pics/3/3-115.jpg
https://danderm-pdv.is.kkh.dk/atlas/pics/3/3-116.jpg
https://danderm-pdv.is.kkh.dk/atlas/pics/3/3-117.jpg
https://danderm-pdv.is.kkh.dk/atlas/pics/3/3-118.jpg
https://danderm-pdv.is.kkh.dk/atlas/pics/3/3-118-6.jpg
https://danderm-pdv.is.kkh.dk/atlas/pics/3/3-118-7.jpg
https://danderm-pdv.is.kkh.dk/atlas/pics/3/3-118-8.jpg
https://danderm-pdv.is.kkh.dk/atlas/pics/3/3-118-9.jpg
https://danderm-pdv.is.kkh.dk/atlas/pics/3/3-118-10.jpg
https://danderm-pdv.is.kkh.dk/atlas/pics/3/3-118-11.jpg
https://danderm-pdv.is.kkh.dk/atlas/pics/3/3-119.jpg
https://danderm-pdv.is.kkh.dk/atlas/pics/3/3-119-1.jpg
https://danderm-pdv.is.kkh.dk/atlas/pics/3/3-119-2.jpg
https://danderm-pdv.is.kkh.dk/atlas/pics/3/3-119-3.jpg
https://danderm-pdv.is.kkh.dk/atlas/pics/3/3-119-4.jpg
https://danderm-pdv.is.kkh.dk/atlas/pics/3/3-119-6 .jpg
https://danderm-pdv.is.kkh.dk/atlas/pics/3/3-119-7.jpg
https://danderm-pdv.is.kkh.dk/atlas/pics/3/3-119-8.jpg

https://danderm-pdv.is.kkh.dk/atlas/pics/3/3-119-9 .jpg
https://danderm-pdv.is.kkh.dk/atlas/pics/3/3-119-10.jpg
https://danderm-pdv.is.kkh.dk/atlas/pics/3/3-119-11.jpg
https://danderm-pdv.is.kkh.dk/atlas/pics/3/3-119-12.jpg
https://danderm-pdv.is.kkh.dk/atlas/pics/3/3-119-13.jpg
https://danderm-pdv.is.kkh.dk/atlas/pics/3/3-119-14 .jpg
https://danderm-pdv.is.kkh.dk/atlas/pics/3/3-119-15.jpg
https://danderm-pdv.is.kkh.dk/atlas/pics/3/3-119-16.jpg
https://danderm-pdv.is.kkh.dk/atlas/pics/3/3-119-17.jpg
https://danderm-pdv.is.kkh.dk/atlas/pics/3/3-119-18 .jpg
https://danderm-pdv.is.kkh.dk/atlas/pics/3/3-119-19.jpg
https://danderm-pdv.is.kkh.dk/atlas/pics/3/3-119-20.jpg
https://danderm-pdv.is.kkh.dk/atlas/pics/3/3-119-21.jpg
https://danderm-pdv.is.kkh.dk/atlas/pics/3/3-119-22.jpg
https://danderm-pdv.is.kkh.dk/atlas/pics/3/3-119-25.jpg
https://danderm-pdv.is.kkh.dk/atlas/pics/3/3-119-26.jpg
https://danderm-pdv.is.kkh.dk/atlas/pics/3/3-119-27.jpg
https://danderm-pdv.is.kkh.dk/atlas/pics/3/3-120.jpg
https://danderm-pdv.is.kkh.dk/atlas/pics/3/3-121.jpg
https://danderm-pdv.is.kkh.dk/atlas/pics/3/3-122.jpg
https://danderm-pdv.is.kkh.dk/atlas/pics/3/3-123.jpg
https://danderm-pdv.is.kkh.dk/atlas/pics/3/3-123-1.jpg
https://danderm-pdv.is.kkh.dk/atlas/pics/3/3-123-2.jpg
https://danderm-pdv.is.kkh.dk/atlas/pics/3/3-123-3.jpg
https://danderm-pdv.is.kkh.dk/atlas/pics/3/3-123-4.jpg
https://danderm-pdv.is.kkh.dk/atlas/pics/3/3-123-5.jpg
https://danderm-pdv.is.kkh.dk/atlas/pics/3/3-123-6.jpg
https://danderm-pdv.is.kkh.dk/atlas/pics/3/3-123-7.jpg
https://danderm-pdv.is.kkh.dk/atlas/pics/3/3-123-8.jpg
https://danderm-pdv.is.kkh.dk/atlas/pics/3/3-123-9.jpg
https://danderm-pdv.is.kkh.dk/atlas/pics/3/3-123-10.jpg
https://danderm-pdv.is.kkh.dk/atlas/pics/3/3-123-11.jpg
https://danderm-pdv.is.kkh.dk/atlas/pics/3/3-123-12.jpg
https://danderm-pdv.is.kkh.dk/atlas/pics/3/3-123-13.jpg
https://danderm-pdv.is.kkh.dk/atlas/pics/3/3-124.jpg
https://danderm-pdv.is.kkh.dk/atlas/pics/3/3-124-1.jpg
https://danderm-pdv.is.kkh.dk/atlas/pics/3/3-124-2.jpg
https://danderm-pdv.is.kkh.dk/atlas/pics/3/3-124-3.jpg
https://danderm-pdv.is.kkh.dk/atlas/pics/3/3-124-4.jpg
https://danderm-pdv.is.kkh.dk/atlas/pics/3/3-124-5.jpg
https://danderm-pdv.is.kkh.dk/atlas/pics/3/3-125.jpg
https://danderm-pdv.is.kkh.dk/atlas/pics/3/3-126.jpg
https://danderm-pdv.is.kkh.dk/atlas/pics/3/3-126-1.jpg
https://danderm-pdv.is.kkh.dk/atlas/pics/3/3-126-2.jpg
https://danderm-pdv.is.kkh.dk/atlas/pics/3/3-126-3.jpg
https://danderm-pdv.is.kkh.dk/atlas/pics/3/3-126-4.jpg
https://danderm-pdv.is.kkh.dk/atlas/pics/3/3-127.jpg
https://danderm-pdv.is.kkh.dk/atlas/pics/3/3-128.jpg

https://danderm-pdv.is.kkh.dk/atlas/pics/3/3-128-1.jpg
https://danderm-pdv.is.kkh.dk/atlas/pics/3/3-128-2.jpg
https://danderm-pdv.is.kkh.dk/atlas/pics/3/3-128-3.jpg
https://danderm-pdv.is.kkh.dk/atlas/pics/3/3-128-4.jpg
https://danderm-pdv.is.kkh.dk/atlas/pics/3/3-129.jpg
https://danderm-pdv.is.kkh.dk/atlas/pics/3/3-130.jpg
https://danderm-pdv.is.kkh.dk/atlas/pics/3/3-130-1.jpg
https://danderm-pdv.is.kkh.dk/atlas/pics/3/3-130-2.jpg
https://danderm-pdv.is.kkh.dk/atlas/pics/3/3-130-3.jpg
https://danderm-pdv.is.kkh.dk/atlas/pics/3/3-130-4.jpg
https://danderm-pdv.is.kkh.dk/atlas/pics/3/3-130-5.jpg
https://danderm-pdv.is.kkh.dk/atlas/pics/3/3-131.jpg
https://danderm-pdv.is.kkh.dk/atlas/pics/3/3-132.jpg
https://danderm-pdv.is.kkh.dk/atlas/pics/3/3-133.jpg
https://danderm-pdv.is.kkh.dk/atlas/pics/3/3-134.jpg
https://danderm-pdv.is.kkh.dk/atlas/pics/3/3-135.jpg
https://danderm-pdv.is.kkh.dk/atlas/pics/3/3-135-8.jpg
https://danderm-pdv.is.kkh.dk/atlas/pics/3/3-135-9.jpg
https://danderm-pdv.is.kkh.dk/atlas/pics/3/3-136.jpg
https://danderm-pdv.is.kkh.dk/atlas/pics/3/3-137.jpg
https://danderm-pdv.is.kkh.dk/atlas/pics/3/3-138.jpg
https://danderm-pdv.is.kkh.dk/atlas/pics/3/3-139.jpg
https://danderm-pdv.is.kkh.dk/atlas/pics/3/3-140.jpg
https://danderm-pdv.is.kkh.dk/atlas/pics/3/3-140-1.jpg
https://danderm-pdv.is.kkh.dk/atlas/pics/3/3-140-2.jpg
https://danderm-pdv.is.kkh.dk/atlas/pics/3/3-141.jpg
https://danderm-pdv.is.kkh.dk/atlas/pics/3/3-142.jpg
https://danderm-pdv.is.kkh.dk/atlas/pics/3/3-142-1.jpg
https://danderm-pdv.is.kkh.dk/atlas/pics/3/3-142-2.jpg
https://danderm-pdv.is.kkh.dk/atlas/pics/3/3-143.jpg
https://danderm-pdv.is.kkh.dk/atlas/pics/3/3-143-2.jpg
https://danderm-pdv.is.kkh.dk/atlas/pics/3/3-143-3.jpg
https://danderm-pdv.is.kkh.dk/atlas/pics/3/3-144.jpg
https://danderm-pdv.is.kkh.dk/atlas/pics/3/3-145.jpg
https://danderm-pdv.is.kkh.dk/atlas/pics/3/3-146-slor.jpg
https://danderm-pdv.is.kkh.dk/atlas/pics/3/3-147.jpg
https://danderm-pdv.is.kkh.dk/atlas/pics/3/3-148.jpg
https://danderm-pdv.is.kkh.dk/atlas/pics/3/3-149.jpg
https://danderm-pdv.is.kkh.dk/atlas/pics/3/3-150.jpg
https://danderm-pdv.is.kkh.dk/atlas/pics/3/3-151.jpg
https://danderm-pdv.is.kkh.dk/atlas/pics/3/3-152.jpg
https://danderm-pdv.is.kkh.dk/atlas/pics/3/3-153.jpg
https://danderm-pdv.is.kkh.dk/atlas/pics/3/3-154.jpg
https://danderm-pdv.is.kkh.dk/atlas/pics/3/3-155.jpg
https://danderm-pdv.is.kkh.dk/atlas/pics/3/3-155-1.jpg
https://danderm-pdv.is.kkh.dk/atlas/pics/3/3-156.jpg
https://danderm-pdv.is.kkh.dk/atlas/pics/3/3-157.jpg
https://danderm-pdv.is.kkh.dk/atlas/pics/3/3-158.jpg

https://danderm-pdv.is.kkh.dk/atlas/pics/3/3-159.jpg
https://danderm-pdv.is.kkh.dk/atlas/pics/3/3-160.jpg
https://danderm-pdv.is.kkh.dk/atlas/pics/3/3-161.jpg
https://danderm-pdv.is.kkh.dk/atlas/pics/3/3-162.jpg
https://danderm-pdv.is.kkh.dk/atlas/pics/3/3-163.jpg
https://danderm-pdv.is.kkh.dk/atlas/pics/3/3-163-1.jpg
https://danderm-pdv.is.kkh.dk/atlas/pics/3/3-163-2.jpg
https://danderm-pdv.is.kkh.dk/atlas/pics/3/3-163-3.jpg
https://danderm-pdv.is.kkh.dk/atlas/pics/3/3-163-4.jpg
https://danderm-pdv.is.kkh.dk/atlas/pics/3/3-164.jpg
https://danderm-pdv.is.kkh.dk/atlas/pics/3/3-165.jpg
https://danderm-pdv.is.kkh.dk/atlas/pics/3/3-166.jpg
https://danderm-pdv.is.kkh.dk/atlas/pics/3/3-167.jpg
https://danderm-pdv.is.kkh.dk/atlas/pics/3/3-168.jpg
https://danderm-pdv.is.kkh.dk/atlas/pics/3/3-168-2.jpg
https://danderm-pdv.is.kkh.dk/atlas/pics/3/3-169.jpg
https://danderm-pdv.is.kkh.dk/atlas/pics/3/3-170.jpg
https://danderm-pdv.is.kkh.dk/atlas/pics/3/3-170-1.jpg
https://danderm-pdv.is.kkh.dk/atlas/pics/3/3-170-2.jpg
https://danderm-pdv.is.kkh.dk/atlas/pics/3/3-170-3.jpg
https://danderm-pdv.is.kkh.dk/atlas/pics/3/3-171.jpg
https://danderm-pdv.is.kkh.dk/atlas/pics/3/3-172.jpg
https://danderm-pdv.is.kkh.dk/atlas/pics/3/3-172-1.jpg
https://danderm-pdv.is.kkh.dk/atlas/pics/3/3-172-2.jpg
https://danderm-pdv.is.kkh.dk/atlas/pics/3/3-173.jpg
https://danderm-pdv.is.kkh.dk/atlas/pics/3/3-173-1.jpg
https://danderm-pdv.is.kkh.dk/atlas/pics/3/3-174.jpg
https://danderm-pdv.is.kkh.dk/atlas/pics/3/3-174-1.jpg
https://danderm-pdv.is.kkh.dk/atlas/pics/3/3-174-2.jpg
https://danderm-pdv.is.kkh.dk/atlas/pics/3/3-174-3.jpg
https://danderm-pdv.is.kkh.dk/atlas/pics/3/3-174-4.jpg
https://danderm-pdv.is.kkh.dk/atlas/pics/3/3-174-5.jpg
https://danderm-pdv.is.kkh.dk/atlas/pics/3/3-174-6.jpg
https://danderm-pdv.is.kkh.dk/atlas/pics/3/3-174-7.jpg
https://danderm-pdv.is.kkh.dk/atlas/pics/3/3-174-8.jpg
https://danderm-pdv.is.kkh.dk/atlas/pics/3/3-175.jpg
https://danderm-pdv.is.kkh.dk/atlas/pics/3/3-176.jpg
https://danderm-pdv.is.kkh.dk/atlas/pics/3/3-177.jpg
https://danderm-pdv.is.kkh.dk/atlas/pics/3/3-178.jpg
https://danderm-pdv.is.kkh.dk/atlas/pics/3/3-178-1.jpg
https://danderm-pdv.is.kkh.dk/atlas/pics/3/3-178-2.jpg
https://danderm-pdv.is.kkh.dk/atlas/pics/3/3-178-3.jpg
https://danderm-pdv.is.kkh.dk/atlas/pics/3/3-178-4.jpg
https://danderm-pdv.is.kkh.dk/atlas/pics/3/3-178-5.jpg
https://danderm-pdv.is.kkh.dk/atlas/pics/3/3-178-6.jpg
https://danderm-pdv.is.kkh.dk/atlas/pics/3/3-178-7.jpg
https://danderm-pdv.is.kkh.dk/atlas/pics/3/3-178-8 .jpg
https://danderm-pdv.is.kkh.dk/atlas/pics/3/3-178-9.jpg

https://danderm-pdv.is.kkh.dk/atlas/pics/3/3-178-10.jpg
https://danderm-pdv.is.kkh.dk/atlas/pics/3/3-209.jpg
https://danderm-pdv.is.kkh.dk/atlas/pics/4/4-1.jpg
https://danderm-pdv.is.kkh.dk/atlas/pics/4/4-2.jpg
https://danderm-pdv.is.kkh.dk/atlas/pics/4/4-2-1.jpg
https://danderm-pdv.is.kkh.dk/atlas/pics/4/4-2-2.jpg
https://danderm-pdv.is.kkh.dk/atlas/pics/4/4-3.jpg
https://danderm-pdv.is.kkh.dk/atlas/pics/4/4-4.jpg
https://danderm-pdv.is.kkh.dk/atlas/pics/4/4-5.jpg
https://danderm-pdv.is.kkh.dk/atlas/pics/4/4-6_S.jpg
https://danderm-pdv.is.kkh.dk/atlas/pics/4/4-6-1.jpg
https://danderm-pdv.is.kkh.dk/atlas/pics/4/4-6-2.jpg
https://danderm-pdv.is.kkh.dk/atlas/pics/4/4-6-3.jpg
https://danderm-pdv.is.kkh.dk/atlas/pics/4/4-6-7.jpg
https://danderm-pdv.is.kkh.dk/atlas/pics/4/4-6-8.jpg
https://danderm-pdv.is.kkh.dk/atlas/pics/4/4-6-9.jpg
https://danderm-pdv.is.kkh.dk/atlas/pics/4/4-7.jpg
https://danderm-pdv.is.kkh.dk/atlas/pics/4/4-7-1.jpg
https://danderm-pdv.is.kkh.dk/atlas/pics/4/4-7-2.jpg
https://danderm-pdv.is.kkh.dk/atlas/pics/4/4-8.jpg
https://danderm-pdv.is.kkh.dk/atlas/pics/4/4-9.jpg
https://danderm-pdv.is.kkh.dk/atlas/pics/4/4-10.jpg
https://danderm-pdv.is.kkh.dk/atlas/pics/4/4-10-1.jpg
https://danderm-pdv.is.kkh.dk/atlas/pics/4/4-11-1.jpg
https://danderm-pdv.is.kkh.dk/atlas/pics/4/4-11-2.jpg
https://danderm-pdv.is.kkh.dk/atlas/pics/4/4-11-3.jpg
https://danderm-pdv.is.kkh.dk/atlas/pics/4/4-11-4.jpg
https://danderm-pdv.is.kkh.dk/atlas/pics/4/4-11-5.jpg
https://danderm-pdv.is.kkh.dk/atlas/pics/4/4-12.jpg
https://danderm-pdv.is.kkh.dk/atlas/pics/4/4-12-1.jpg
https://danderm-pdv.is.kkh.dk/atlas/pics/4/4-13.jpg
https://danderm-pdv.is.kkh.dk/atlas/pics/4/4-14.jpg
https://danderm-pdv.is.kkh.dk/atlas/pics/4/4-14-1.jpg
https://danderm-pdv.is.kkh.dk/atlas/pics/4/4-14-2.jpg
https://danderm-pdv.is.kkh.dk/atlas/pics/4/4-14-3.jpg
https://danderm-pdv.is.kkh.dk/atlas/pics/4/4-14-4.jpg
https://danderm-pdv.is.kkh.dk/atlas/pics/4/4-14-5.jpg
https://danderm-pdv.is.kkh.dk/atlas/pics/4/4-14-6.jpg
https://danderm-pdv.is.kkh.dk/atlas/pics/4/4-14-7.jpg
https://danderm-pdv.is.kkh.dk/atlas/pics/4/4-15.jpg
https://danderm-pdv.is.kkh.dk/atlas/pics/4/4-16.jpg
https://danderm-pdv.is.kkh.dk/atlas/pics/4/4-16-1.jpg
https://danderm-pdv.is.kkh.dk/atlas/pics/4/4-17.jpg
https://danderm-pdv.is.kkh.dk/atlas/pics/4/4-18.jpg
https://danderm-pdv.is.kkh.dk/atlas/pics/4/4-18-1.jpg
https://danderm-pdv.is.kkh.dk/atlas/pics/4/4-18-2.jpg
https://danderm-pdv.is.kkh.dk/atlas/pics/4/4-18-3.jpg
https://danderm-pdv.is.kkh.dk/atlas/pics/4/4-18-4.jpg

https://danderm-pdv.is.kkh.dk/atlas/pics/4/4-18-4-1.jpg
https://danderm-pdv.is.kkh.dk/atlas/pics/4/4-18-4-2.jpg
https://danderm-pdv.is.kkh.dk/atlas/pics/4/4-18-4-3.jpg
https://danderm-pdv.is.kkh.dk/atlas/pics/4/4-18-4-4.jpg
https://danderm-pdv.is.kkh.dk/atlas/pics/4/4-18-4-5.jpg
https://danderm-pdv.is.kkh.dk/atlas/pics/4/4-18-4-6.jpg
https://danderm-pdv.is.kkh.dk/atlas/pics/4/4-18-4-9.jpg
https://danderm-pdv.is.kkh.dk/atlas/pics/4/4-18-4-10.jpg
https://danderm-pdv.is.kkh.dk/atlas/pics/4/4-18-4-11.jpg
https://danderm-pdv.is.kkh.dk/atlas/pics/4/4-18-5.jpg
https://danderm-pdv.is.kkh.dk/atlas/pics/4/4-18-6.jpg
https://danderm-pdv.is.kkh.dk/atlas/pics/4/4-19.jpg
https://danderm-pdv.is.kkh.dk/atlas/pics/4/4-20-1.jpg
https://danderm-pdv.is.kkh.dk/atlas/pics/4/4-21.jpg
https://danderm-pdv.is.kkh.dk/atlas/pics/4/4-21-1.jpg
https://danderm-pdv.is.kkh.dk/atlas/pics/4/4-21-2.jpg
https://danderm-pdv.is.kkh.dk/atlas/pics/4/4-21-3.jpg
https://danderm-pdv.is.kkh.dk/atlas/pics/4/4-21-4.jpg
https://danderm-pdv.is.kkh.dk/atlas/pics/4/4-22_S.jpg
https://danderm-pdv.is.kkh.dk/atlas/pics/4/4-22-1.jpg
https://danderm-pdv.is.kkh.dk/atlas/pics/4/4-23.jpg
https://danderm-pdv.is.kkh.dk/atlas/pics/4/4-24.jpg
https://danderm-pdv.is.kkh.dk/atlas/pics/4/4-24-1.jpg
https://danderm-pdv.is.kkh.dk/atlas/pics/4/4-24-2.jpg
https://danderm-pdv.is.kkh.dk/atlas/pics/4/4-25_S.jpg
https://danderm-pdv.is.kkh.dk/atlas/pics/4/4-25-1.jpg
https://danderm-pdv.is.kkh.dk/atlas/pics/4/4-25-2.jpg
https://danderm-pdv.is.kkh.dk/atlas/pics/4/4-25-3.jpg
https://danderm-pdv.is.kkh.dk/atlas/pics/4/4-25-4.jpg
https://danderm-pdv.is.kkh.dk/atlas/pics/4/4-25-5.jpg
https://danderm-pdv.is.kkh.dk/atlas/pics/4/4-26.jpg
https://danderm-pdv.is.kkh.dk/atlas/pics/4/4-27.jpg
https://danderm-pdv.is.kkh.dk/atlas/pics/4/4-27-1.jpg
https://danderm-pdv.is.kkh.dk/atlas/pics/4/4-27-2.jpg
https://danderm-pdv.is.kkh.dk/atlas/pics/4/4-27-1-1.jpg
https://danderm-pdv.is.kkh.dk/atlas/pics/4/4-27-1-2.jpg
https://danderm-pdv.is.kkh.dk/atlas/pics/4/4-27-1-3.jpg
https://danderm-pdv.is.kkh.dk/atlas/pics/4/4-27-1-4.jpg
https://danderm-pdv.is.kkh.dk/atlas/pics/4/4-27-1-5.jpg
https://danderm-pdv.is.kkh.dk/atlas/pics/4/4-27-1-6.jpg
https://danderm-pdv.is.kkh.dk/atlas/pics/4/4-27-1-7.jpg
https://danderm-pdv.is.kkh.dk/atlas/pics/4/4-27-1-8.jpg
https://danderm-pdv.is.kkh.dk/atlas/pics/4/4-27-3.jpg
https://danderm-pdv.is.kkh.dk/atlas/pics/4/4-27-4.jpg
https://danderm-pdv.is.kkh.dk/atlas/pics/4/4-27-5.jpg
https://danderm-pdv.is.kkh.dk/atlas/pics/4/4-27-6.jpg
https://danderm-pdv.is.kkh.dk/atlas/pics/4/4-28.jpg
https://danderm-pdv.is.kkh.dk/atlas/pics/4/4-29.jpg

https://danderm-pdv.is.kkh.dk/atlas/pics/4/4-29-1.jpg
https://danderm-pdv.is.kkh.dk/atlas/pics/4/4-29-2.jpg
https://danderm-pdv.is.kkh.dk/atlas/pics/4/4-29-3.jpg
https://danderm-pdv.is.kkh.dk/atlas/pics/4/4-29-4.jpg
https://danderm-pdv.is.kkh.dk/atlas/pics/4/4-29-5.jpg
https://danderm-pdv.is.kkh.dk/atlas/pics/4/4-29-6.jpg
https://danderm-pdv.is.kkh.dk/atlas/pics/4/4-29-7.jpg
https://danderm-pdv.is.kkh.dk/atlas/pics/4/4-29-8.jpg
https://danderm-pdv.is.kkh.dk/atlas/pics/4/4-30.jpg
https://danderm-pdv.is.kkh.dk/atlas/pics/4/4-30-1.jpg
https://danderm-pdv.is.kkh.dk/atlas/pics/4/4-31.jpg
https://danderm-pdv.is.kkh.dk/atlas/pics/4/4-32.jpg
https://danderm-pdv.is.kkh.dk/atlas/pics/4/4-33.jpg
https://danderm-pdv.is.kkh.dk/atlas/pics/4/4-34.jpg
https://danderm-pdv.is.kkh.dk/atlas/pics/4/4-34-1.jpg
https://danderm-pdv.is.kkh.dk/atlas/pics/4/4-34-2.jpg
https://danderm-pdv.is.kkh.dk/atlas/pics/4/4-34-3.jpg
https://danderm-pdv.is.kkh.dk/atlas/pics/4/4-35_S.jpg
https://danderm-pdv.is.kkh.dk/atlas/pics/4/4-36.jpg
https://danderm-pdv.is.kkh.dk/atlas/pics/4/4-37.jpg
https://danderm-pdv.is.kkh.dk/atlas/pics/4/4-37-1.jpg
https://danderm-pdv.is.kkh.dk/atlas/pics/4/4-38.jpg
https://danderm-pdv.is.kkh.dk/atlas/pics/4/4-38-1.jpg
https://danderm-pdv.is.kkh.dk/atlas/pics/4/4-38-2.jpg
https://danderm-pdv.is.kkh.dk/atlas/pics/4/4-38-3.jpg
https://danderm-pdv.is.kkh.dk/atlas/pics/4/4-39.jpg
https://danderm-pdv.is.kkh.dk/atlas/pics/4/4-40.jpg
https://danderm-pdv.is.kkh.dk/atlas/pics/4/4-40-1.jpg
https://danderm-pdv.is.kkh.dk/atlas/pics/4/4-40-2.jpg
https://danderm-pdv.is.kkh.dk/atlas/pics/4/4-40-3.jpg
https://danderm-pdv.is.kkh.dk/atlas/pics/4/4-40-4.jpg
https://danderm-pdv.is.kkh.dk/atlas/pics/4/4-40-5.jpg
https://danderm-pdv.is.kkh.dk/atlas/pics/4/4-40-6.jpg
https://danderm-pdv.is.kkh.dk/atlas/pics/4/4-40-7.jpg
https://danderm-pdv.is.kkh.dk/atlas/pics/4/4-40-8.jpg
https://danderm-pdv.is.kkh.dk/atlas/pics/4/4-40-9.jpg
https://danderm-pdv.is.kkh.dk/atlas/pics/4/4-40-10.jpg
https://danderm-pdv.is.kkh.dk/atlas/pics/4/4-41.jpg
https://danderm-pdv.is.kkh.dk/atlas/pics/4/4-41-1.jpg
https://danderm-pdv.is.kkh.dk/atlas/pics/4/4-41-2.jpg
https://danderm-pdv.is.kkh.dk/atlas/pics/4/4-41-3.jpg
https://danderm-pdv.is.kkh.dk/atlas/pics/4/4-41-4.jpg
https://danderm-pdv.is.kkh.dk/atlas/pics/4/4-42.jpg
https://danderm-pdv.is.kkh.dk/atlas/pics/4/4-43.jpg
https://danderm-pdv.is.kkh.dk/atlas/pics/4/4-43-1 .jpg
https://danderm-pdv.is.kkh.dk/atlas/pics/4/4-43-2.jpg
https://danderm-pdv.is.kkh.dk/atlas/pics/4/4-43-3 .jpg
https://danderm-pdv.is.kkh.dk/atlas/pics/4/4-44.jpg

https://danderm-pdv.is.kkh.dk/atlas/pics/4/4-44-1.jpg
https://danderm-pdv.is.kkh.dk/atlas/pics/4/4-44-2.jpg
https://danderm-pdv.is.kkh.dk/atlas/pics/4/4-44-4.jpg
https://danderm-pdv.is.kkh.dk/atlas/pics/4/4-44-5.jpg
https://danderm-pdv.is.kkh.dk/atlas/pics/4/4-45.jpg
https://danderm-pdv.is.kkh.dk/atlas/pics/4/4-45-1.jpg
https://danderm-pdv.is.kkh.dk/atlas/pics/4/4-45-2.jpg
https://danderm-pdv.is.kkh.dk/atlas/pics/4/4-45-3.jpg
https://danderm-pdv.is.kkh.dk/atlas/pics/4/4-45-4.jpg
https://danderm-pdv.is.kkh.dk/atlas/pics/4/4-45-5.jpg
https://danderm-pdv.is.kkh.dk/atlas/pics/4/4-46.jpg
https://danderm-pdv.is.kkh.dk/atlas/pics/4/4-47.jpg
https://danderm-pdv.is.kkh.dk/atlas/pics/4/4-48.jpg
https://danderm-pdv.is.kkh.dk/atlas/pics/4/4-49.jpg
https://danderm-pdv.is.kkh.dk/atlas/pics/4/4-49-1.jpg
https://danderm-pdv.is.kkh.dk/atlas/pics/4/4-50.jpg
https://danderm-pdv.is.kkh.dk/atlas/pics/4/4-51.jpg
https://danderm-pdv.is.kkh.dk/atlas/pics/4/4-52.jpg
https://danderm-pdv.is.kkh.dk/atlas/pics/4/4-52-1.jpg
https://danderm-pdv.is.kkh.dk/atlas/pics/4/4-53.jpg
https://danderm-pdv.is.kkh.dk/atlas/pics/4/4-54.jpg
https://danderm-pdv.is.kkh.dk/atlas/pics/4/4-54-1.jpg
https://danderm-pdv.is.kkh.dk/atlas/pics/4/4-55.jpg
https://danderm-pdv.is.kkh.dk/atlas/pics/4/4-55-1.jpg
https://danderm-pdv.is.kkh.dk/atlas/pics/4/4-56.jpg
https://danderm-pdv.is.kkh.dk/atlas/pics/4/4-56-1.jpg
https://danderm-pdv.is.kkh.dk/atlas/pics/4/4-57.jpg
https://danderm-pdv.is.kkh.dk/atlas/pics/4/4-58.jpg
https://danderm-pdv.is.kkh.dk/atlas/pics/4/4-59.jpg
https://danderm-pdv.is.kkh.dk/atlas/pics/4/4-59-1.jpg
https://danderm-pdv.is.kkh.dk/atlas/pics/4/4-59-2.jpg
https://danderm-pdv.is.kkh.dk/atlas/pics/4/4-59-3.jpg
https://danderm-pdv.is.kkh.dk/atlas/pics/4/4-59-4.jpg
https://danderm-pdv.is.kkh.dk/atlas/pics/4/4-59-5.jpg
https://danderm-pdv.is.kkh.dk/atlas/pics/4/4-59-6.jpg
https://danderm-pdv.is.kkh.dk/atlas/pics/4/4-60.jpg
https://danderm-pdv.is.kkh.dk/atlas/pics/4/4-61.jpg
https://danderm-pdv.is.kkh.dk/atlas/pics/4/4-62.jpg
https://danderm-pdv.is.kkh.dk/atlas/pics/4/4-63.jpg
https://danderm-pdv.is.kkh.dk/atlas/pics/4/4-63-1.jpg
https://danderm-pdv.is.kkh.dk/atlas/pics/4/4-63-2.jpg
https://danderm-pdv.is.kkh.dk/atlas/pics/4/4-63-3.jpg
https://danderm-pdv.is.kkh.dk/atlas/pics/4/4-63-5.jpg
https://danderm-pdv.is.kkh.dk/atlas/pics/4/4-63-6 .jpg
https://danderm-pdv.is.kkh.dk/atlas/pics/4/4-64.jpg
https://danderm-pdv.is.kkh.dk/atlas/pics/4/4-65.jpg
https://danderm-pdv.is.kkh.dk/atlas/pics/4/4-66.jpg
https://danderm-pdv.is.kkh.dk/atlas/pics/4/4-67.jpg

https://danderm-pdv.is.kkh.dk/atlas/pics/4/4-67-1.jpg
https://danderm-pdv.is.kkh.dk/atlas/pics/4/4-67-2.jpg
https://danderm-pdv.is.kkh.dk/atlas/pics/4/4-67-3.jpg
https://danderm-pdv.is.kkh.dk/atlas/pics/4/4-67-4.jpg
https://danderm-pdv.is.kkh.dk/atlas/pics/4/4-67-5.jpg
https://danderm-pdv.is.kkh.dk/atlas/pics/4/4-67-6.jpg
https://danderm-pdv.is.kkh.dk/atlas/pics/4/4-68.jpg
https://danderm-pdv.is.kkh.dk/atlas/pics/4/4-69.jpg
https://danderm-pdv.is.kkh.dk/atlas/pics/4/4-70.jpg
https://danderm-pdv.is.kkh.dk/atlas/pics/4/4-71.jpg
https://danderm-pdv.is.kkh.dk/atlas/pics/4/4-72-1.jpg
https://danderm-pdv.is.kkh.dk/atlas/pics/4/4-73-1.jpg
https://danderm-pdv.is.kkh.dk/atlas/pics/4/4-74-1.jpg
https://danderm-pdv.is.kkh.dk/atlas/pics/4/4-74-2.jpg
https://danderm-pdv.is.kkh.dk/atlas/pics/4/4-74-4.jpg
https://danderm-pdv.is.kkh.dk/atlas/pics/4/4-74-5.jpg
https://danderm-pdv.is.kkh.dk/atlas/pics/4/4-74-6.jpg
https://danderm-pdv.is.kkh.dk/atlas/pics/4/4-74-7.jpg
https://danderm-pdv.is.kkh.dk/atlas/pics/4/4-74-9.jpg
https://danderm-pdv.is.kkh.dk/atlas/pics/4/4-74-10.jpg
https://danderm-pdv.is.kkh.dk/atlas/pics/4/4-74-11.jpg
https://danderm-pdv.is.kkh.dk/atlas/pics/4/4-74-12.jpg
https://danderm-pdv.is.kkh.dk/atlas/pics/4/4-74-13.jpg
https://danderm-pdv.is.kkh.dk/atlas/pics/4/4-74-14.jpg
https://danderm-pdv.is.kkh.dk/atlas/pics/4/4-74-15.jpg
https://danderm-pdv.is.kkh.dk/atlas/pics/4/4-77.jpg
https://danderm-pdv.is.kkh.dk/atlas/pics/4/4-77-1.jpg
https://danderm-pdv.is.kkh.dk/atlas/pics/4/4-78.jpg
https://danderm-pdv.is.kkh.dk/atlas/pics/4/4-78-1.jpg
https://danderm-pdv.is.kkh.dk/atlas/pics/4/4-78-2.jpg
https://danderm-pdv.is.kkh.dk/atlas/pics/4/4-78-3 .jpg
https://danderm-pdv.is.kkh.dk/atlas/pics/4/4-79.jpg
https://danderm-pdv.is.kkh.dk/atlas/pics/4/4-79-1.jpg
https://danderm-pdv.is.kkh.dk/atlas/pics/4/4-79-2.jpg
https://danderm-pdv.is.kkh.dk/atlas/pics/4/4-79-3.jpg
https://danderm-pdv.is.kkh.dk/atlas/pics/4/4-79-4 .jpg
https://danderm-pdv.is.kkh.dk/atlas/pics/4/4-80.jpg
https://danderm-pdv.is.kkh.dk/atlas/pics/4/4-80-1.jpg
https://danderm-pdv.is.kkh.dk/atlas/pics/4/4-80-2.jpg
https://danderm-pdv.is.kkh.dk/atlas/pics/4/4-80-3.jpg
https://danderm-pdv.is.kkh.dk/atlas/pics/4/4-80-4.jpg
https://danderm-pdv.is.kkh.dk/atlas/pics/4/4-80-5.jpg
https://danderm-pdv.is.kkh.dk/atlas/pics/4/4-80-6.jpg
https://danderm-pdv.is.kkh.dk/atlas/pics/4/4-80-7.jpg
https://danderm-pdv.is.kkh.dk/atlas/pics/4/4-80-10.jpg
https://danderm-pdv.is.kkh.dk/atlas/pics/4/4-80-11.jpg
https://danderm-pdv.is.kkh.dk/atlas/pics/4/4-81.jpg
https://danderm-pdv.is.kkh.dk/atlas/pics/4/4-81-1.jpg

https://danderm-pdv.is.kkh.dk/atlas/pics/4/4-81-3.jpg
https://danderm-pdv.is.kkh.dk/atlas/pics/4/4-81-4.jpg
https://danderm-pdv.is.kkh.dk/atlas/pics/4/4-81-5.jpg
https://danderm-pdv.is.kkh.dk/atlas/pics/4/4-83.jpg
https://danderm-pdv.is.kkh.dk/atlas/pics/4/4-83-1.jpg
https://danderm-pdv.is.kkh.dk/atlas/pics/4/4-84.jpg
https://danderm-pdv.is.kkh.dk/atlas/pics/4/4-84-1.jpg
https://danderm-pdv.is.kkh.dk/atlas/pics/4/4-84-2.jpg
https://danderm-pdv.is.kkh.dk/atlas/pics/4/4-84-3.jpg
https://danderm-pdv.is.kkh.dk/atlas/pics/4/4-84-4.jpg
https://danderm-pdv.is.kkh.dk/atlas/pics/4/4-84-5.jpg
https://danderm-pdv.is.kkh.dk/atlas/pics/4/4-85.jpg
https://danderm-pdv.is.kkh.dk/atlas/pics/4/4-86.jpg
https://danderm-pdv.is.kkh.dk/atlas/pics/4/4-86-1.jpg
https://danderm-pdv.is.kkh.dk/atlas/pics/4/4-86-2.jpg
https://danderm-pdv.is.kkh.dk/atlas/pics/4/4-86-3.jpg
https://danderm-pdv.is.kkh.dk/atlas/pics/4/4-86-4.jpg
https://danderm-pdv.is.kkh.dk/atlas/pics/4/4-86-6.jpg
https://danderm-pdv.is.kkh.dk/atlas/pics/4/4-86-7.jpg
https://danderm-pdv.is.kkh.dk/atlas/pics/4/4-86-8.jpg
https://danderm-pdv.is.kkh.dk/atlas/pics/4/4-86-9.jpg
https://danderm-pdv.is.kkh.dk/atlas/pics/4/4-86-10.jpg
https://danderm-pdv.is.kkh.dk/atlas/pics/4/4-87.jpg
https://danderm-pdv.is.kkh.dk/atlas/pics/4/4-87-1 .jpg
https://danderm-pdv.is.kkh.dk/atlas/pics/4/4-87-2.jpg
https://danderm-pdv.is.kkh.dk/atlas/pics/4/4-87-3 .jpg
https://danderm-pdv.is.kkh.dk/atlas/pics/4/4-88.jpg
https://danderm-pdv.is.kkh.dk/atlas/pics/4/4-89.jpg
https://danderm-pdv.is.kkh.dk/atlas/pics/4/4-90.jpg
https://danderm-pdv.is.kkh.dk/atlas/pics/4/4-90-1.jpg
https://danderm-pdv.is.kkh.dk/atlas/pics/4/4-91.jpg
https://danderm-pdv.is.kkh.dk/atlas/pics/4/4-91-1.jpg
https://danderm-pdv.is.kkh.dk/atlas/pics/4/4-91-2.jpg
https://danderm-pdv.is.kkh.dk/atlas/pics/4/4-91-3.jpg
https://danderm-pdv.is.kkh.dk/atlas/pics/4/4-92.jpg
https://danderm-pdv.is.kkh.dk/atlas/pics/4/4-92-1.jpg
https://danderm-pdv.is.kkh.dk/atlas/pics/4/4-92-2.jpg
https://danderm-pdv.is.kkh.dk/atlas/pics/4/4-92-3.jpg
https://danderm-pdv.is.kkh.dk/atlas/pics/4/4-92-4.jpg
https://danderm-pdv.is.kkh.dk/atlas/pics/4/4-92-5.jpg
https://danderm-pdv.is.kkh.dk/atlas/pics/4/4-92-6 .jpg
https://danderm-pdv.is.kkh.dk/atlas/pics/4/4-93.jpg
https://danderm-pdv.is.kkh.dk/atlas/pics/4/4-94.jpg
https://danderm-pdv.is.kkh.dk/atlas/pics/4/4-94-1.jpg
https://danderm-pdv.is.kkh.dk/atlas/pics/4/4-94-2.jpg
https://danderm-pdv.is.kkh.dk/atlas/pics/4/4-94-3.jpg
https://danderm-pdv.is.kkh.dk/atlas/pics/4/4-94-4.jpg
https://danderm-pdv.is.kkh.dk/atlas/pics/4/4-95.jpg

https://danderm-pdv.is.kkh.dk/atlas/pics/4/4-96.jpg
https://danderm-pdv.is.kkh.dk/atlas/pics/4/4-96-1.jpg
https://danderm-pdv.is.kkh.dk/atlas/pics/4/4-97_S.jpg
https://danderm-pdv.is.kkh.dk/atlas/pics/4/4-98.jpg
https://danderm-pdv.is.kkh.dk/atlas/pics/4/4-98-1.jpg
https://danderm-pdv.is.kkh.dk/atlas/pics/4/4-99.jpg
https://danderm-pdv.is.kkh.dk/atlas/pics/4/4-100.jpg
https://danderm-pdv.is.kkh.dk/atlas/pics/4/4-100-a.jpg
https://danderm-pdv.is.kkh.dk/atlas/pics/4/4-100-b.jpg
https://danderm-pdv.is.kkh.dk/atlas/pics/4/4-101_S.jpg
https://danderm-pdv.is.kkh.dk/atlas/pics/4/4-101-1.jpg
https://danderm-pdv.is.kkh.dk/atlas/pics/4/4-101-2.jpg
https://danderm-pdv.is.kkh.dk/atlas/pics/4/4-102.jpg
https://danderm-pdv.is.kkh.dk/atlas/pics/4/4-102-1.jpg
https://danderm-pdv.is.kkh.dk/atlas/pics/4/4-102-2.jpg
https://danderm-pdv.is.kkh.dk/atlas/pics/4/4-102-3.jpg
https://danderm-pdv.is.kkh.dk/atlas/pics/4/4-102-4.jpg
https://danderm-pdv.is.kkh.dk/atlas/pics/4/4-102-5.jpg
https://danderm-pdv.is.kkh.dk/atlas/pics/4/4-102-6.jpg
https://danderm-pdv.is.kkh.dk/atlas/pics/4/4-102-7.jpg
https://danderm-pdv.is.kkh.dk/atlas/pics/4/4-102-8.jpg
https://danderm-pdv.is.kkh.dk/atlas/pics/4/4-102-9.jpg
https://danderm-pdv.is.kkh.dk/atlas/pics/4/4-102-10.jpg
https://danderm-pdv.is.kkh.dk/atlas/pics/4/4-102-11.jpg
https://danderm-pdv.is.kkh.dk/atlas/pics/4/4-102-12.jpg
https://danderm-pdv.is.kkh.dk/atlas/pics/4/4-102-13.jpg
https://danderm-pdv.is.kkh.dk/atlas/pics/4/4-103.jpg
https://danderm-pdv.is.kkh.dk/atlas/pics/4/4-104_S.jpg
https://danderm-pdv.is.kkh.dk/atlas/pics/4/4-105.jpg
https://danderm-pdv.is.kkh.dk/atlas/pics/4/4-105-1.jpg
https://danderm-pdv.is.kkh.dk/atlas/pics/4/4-105-2.jpg
https://danderm-pdv.is.kkh.dk/atlas/pics/4/4-105-3.jpg
https://danderm-pdv.is.kkh.dk/atlas/pics/4/4-105-4.jpg
https://danderm-pdv.is.kkh.dk/atlas/pics/4/4-105-5.jpg
https://danderm-pdv.is.kkh.dk/atlas/pics/4/4-105-6.jpg
https://danderm-pdv.is.kkh.dk/atlas/pics/4/4-105-7.jpg
https://danderm-pdv.is.kkh.dk/atlas/pics/4/4-105-8.jpg
https://danderm-pdv.is.kkh.dk/atlas/pics/4/4-105-9.jpg
https://danderm-pdv.is.kkh.dk/atlas/pics/4/4-105-10.jpg
https://danderm-pdv.is.kkh.dk/atlas/pics/4/4-105-11.jpg
https://danderm-pdv.is.kkh.dk/atlas/pics/4/4-105-12.jpg
https://danderm-pdv.is.kkh.dk/atlas/pics/4/4-106.jpg
https://danderm-pdv.is.kkh.dk/atlas/pics/4/4-107.jpg
https://danderm-pdv.is.kkh.dk/atlas/pics/4/4-108.jpg
https://danderm-pdv.is.kkh.dk/atlas/pics/4/4-109.jpg
https://danderm-pdv.is.kkh.dk/atlas/pics/4/4-110.jpg
https://danderm-pdv.is.kkh.dk/atlas/pics/4/4-111.jpg
https://danderm-pdv.is.kkh.dk/atlas/pics/4/4-112.jpg

https://danderm-pdv.is.kkh.dk/atlas/pics/4/4-113_S.jpg
https://danderm-pdv.is.kkh.dk/atlas/pics/4/4-114.jpg
https://danderm-pdv.is.kkh.dk/atlas/pics/4/4-115.jpg
https://danderm-pdv.is.kkh.dk/atlas/pics/4/4-115-1.jpg
https://danderm-pdv.is.kkh.dk/atlas/pics/4/4-115-2.jpg
https://danderm-pdv.is.kkh.dk/atlas/pics/4/4-116_S.jpg
https://danderm-pdv.is.kkh.dk/atlas/pics/4/4-116-1.jpg
https://danderm-pdv.is.kkh.dk/atlas/pics/4/4-116-2.jpg
https://danderm-pdv.is.kkh.dk/atlas/pics/4/4-117.jpg
https://danderm-pdv.is.kkh.dk/atlas/pics/4/4-117-1.jpg
https://danderm-pdv.is.kkh.dk/atlas/pics/4/4-117-2.jpg
https://danderm-pdv.is.kkh.dk/atlas/pics/4/4-117-3.jpg
https://danderm-pdv.is.kkh.dk/atlas/pics/4/4-118_S.jpg
https://danderm-pdv.is.kkh.dk/atlas/pics/4/4-119_S.jpg
https://danderm-pdv.is.kkh.dk/atlas/pics/4/4-120-1.jpg
https://danderm-pdv.is.kkh.dk/atlas/pics/4/4-120-2.jpg
https://danderm-pdv.is.kkh.dk/atlas/pics/4/4-120-3.jpg
https://danderm-pdv.is.kkh.dk/atlas/pics/4/4-120-4.jpg
https://danderm-pdv.is.kkh.dk/atlas/pics/4/4-120-5.jpg
https://danderm-pdv.is.kkh.dk/atlas/pics/4/4-120-6.jpg
https://danderm-pdv.is.kkh.dk/atlas/pics/4/4-120-7.jpg
https://danderm-pdv.is.kkh.dk/atlas/pics/4/4-120-8.jpg
https://danderm-pdv.is.kkh.dk/atlas/pics/4/4-120-9.jpg
https://danderm-pdv.is.kkh.dk/atlas/pics/4/4-120-10.jpg
https://danderm-pdv.is.kkh.dk/atlas/pics/4/4-120-11.jpg
https://danderm-pdv.is.kkh.dk/atlas/pics/4/4-120-12.jpg
https://danderm-pdv.is.kkh.dk/atlas/pics/4/4-120-13.jpg
https://danderm-pdv.is.kkh.dk/atlas/pics/4/4-121_S.jpg
https://danderm-pdv.is.kkh.dk/atlas/pics/4/4-122_S.jpg
https://danderm-pdv.is.kkh.dk/atlas/pics/4/4-123.jpg
https://danderm-pdv.is.kkh.dk/atlas/pics/4/4-124.jpg
https://danderm-pdv.is.kkh.dk/atlas/pics/4/4-124-1.jpg
https://danderm-pdv.is.kkh.dk/atlas/pics/4/4-124-2.jpg
https://danderm-pdv.is.kkh.dk/atlas/pics/4/4-124-3.jpg
https://danderm-pdv.is.kkh.dk/atlas/pics/4/4-124-5.jpg
https://danderm-pdv.is.kkh.dk/atlas/pics/4/4-124-6.jpg
https://danderm-pdv.is.kkh.dk/atlas/pics/4/4-124-7.jpg
https://danderm-pdv.is.kkh.dk/atlas/pics/4/4-125_S.jpg
https://danderm-pdv.is.kkh.dk/atlas/pics/4/4-126.jpg
https://danderm-pdv.is.kkh.dk/atlas/pics/4/4-126-1.jpg
https://danderm-pdv.is.kkh.dk/atlas/pics/4/4-126-2.jpg
https://danderm-pdv.is.kkh.dk/atlas/pics/4/4-128.jpg
https://danderm-pdv.is.kkh.dk/atlas/pics/4/4-128-1.jpg
https://danderm-pdv.is.kkh.dk/atlas/pics/4/4-128-2.jpg
https://danderm-pdv.is.kkh.dk/atlas/pics/4/4-128-3.jpg
https://danderm-pdv.is.kkh.dk/atlas/pics/4/4-129.jpg
https://danderm-pdv.is.kkh.dk/atlas/pics/4/4-129-1.jpg
https://danderm-pdv.is.kkh.dk/atlas/pics/4/4-129-2.jpg

https://danderm-pdv.is.kkh.dk/atlas/pics/4/4-129-3.jpg
https://danderm-pdv.is.kkh.dk/atlas/pics/4/4-130.jpg
https://danderm-pdv.is.kkh.dk/atlas/pics/4/4-131.jpg
https://danderm-pdv.is.kkh.dk/atlas/pics/4/4-131-1.jpg
https://danderm-pdv.is.kkh.dk/atlas/pics/4/4-131-2.jpg
https://danderm-pdv.is.kkh.dk/atlas/pics/4/4-131-3.jpg
https://danderm-pdv.is.kkh.dk/atlas/pics/4/4-132-1.jpg
https://danderm-pdv.is.kkh.dk/atlas/pics/4/4-132-2.jpg
https://danderm-pdv.is.kkh.dk/atlas/pics/4/4-132-3.jpg
https://danderm-pdv.is.kkh.dk/atlas/pics/4/4-132-4.jpg
https://danderm-pdv.is.kkh.dk/atlas/pics/4/4-132-5.jpg
https://danderm-pdv.is.kkh.dk/atlas/pics/4/4-132-6.jpg
https://danderm-pdv.is.kkh.dk/atlas/pics/4/4-132-7.jpg
https://danderm-pdv.is.kkh.dk/atlas/pics/4/4-132-8.jpg
https://danderm-pdv.is.kkh.dk/atlas/pics/4/4-132-9.jpg
https://danderm-pdv.is.kkh.dk/atlas/pics/4/4-132-10.jpg
https://danderm-pdv.is.kkh.dk/atlas/pics/4/4-132-11.jpg
https://danderm-pdv.is.kkh.dk/atlas/pics/4/4-132-12.jpg
https://danderm-pdv.is.kkh.dk/atlas/pics/4/4-133_S.jpg
https://danderm-pdv.is.kkh.dk/atlas/pics/4/4-134.jpg
https://danderm-pdv.is.kkh.dk/atlas/pics/4/4-135.jpg
https://danderm-pdv.is.kkh.dk/atlas/pics/4/4-135-1.jpg
https://danderm-pdv.is.kkh.dk/atlas/pics/4/4-135-2.jpg
https://danderm-pdv.is.kkh.dk/atlas/pics/4/4-135-3.jpg
https://danderm-pdv.is.kkh.dk/atlas/pics/4/4-135-4.jpg
https://danderm-pdv.is.kkh.dk/atlas/pics/4/4-136_S.jpg
https://danderm-pdv.is.kkh.dk/atlas/pics/4/4-137.jpg
https://danderm-pdv.is.kkh.dk/atlas/pics/4/4-137-1.jpg
https://danderm-pdv.is.kkh.dk/atlas/pics/4/4-137-2.jpg
https://danderm-pdv.is.kkh.dk/atlas/pics/4/4-137-3.jpg
https://danderm-pdv.is.kkh.dk/atlas/pics/4/4-137-4.jpg
https://danderm-pdv.is.kkh.dk/atlas/pics/4/4-137-5.jpg
https://danderm-pdv.is.kkh.dk/atlas/pics/4/4-137-6.jpg
https://danderm-pdv.is.kkh.dk/atlas/pics/4/4-137-7.jpg
https://danderm-pdv.is.kkh.dk/atlas/pics/4/4-137-8.jpg
https://danderm-pdv.is.kkh.dk/atlas/pics/4/4-138_S.jpg
https://danderm-pdv.is.kkh.dk/atlas/pics/4/4-139.jpg
https://danderm-pdv.is.kkh.dk/atlas/pics/4/4-140_S.jpg
https://danderm-pdv.is.kkh.dk/atlas/pics/4/4-141.jpg
https://danderm-pdv.is.kkh.dk/atlas/pics/4/4-141-1.jpg
https://danderm-pdv.is.kkh.dk/atlas/pics/4/4-141-2.jpg
https://danderm-pdv.is.kkh.dk/atlas/pics/4/4-142_S.jpg
https://danderm-pdv.is.kkh.dk/atlas/pics/4/4-142-1.jpg
https://danderm-pdv.is.kkh.dk/atlas/pics/4/4-142-2.jpg
https://danderm-pdv.is.kkh.dk/atlas/pics/4/4-143_S.jpg
https://danderm-pdv.is.kkh.dk/atlas/pics/4/4-144_S.jpg
https://danderm-pdv.is.kkh.dk/atlas/pics/4/4-145_S.jpg
https://danderm-pdv.is.kkh.dk/atlas/pics/4/4-145-1.jpg

https://danderm-pdv.is.kkh.dk/atlas/pics/4/4-145-2.jpg
https://danderm-pdv.is.kkh.dk/atlas/pics/4/4-145-3.jpg
https://danderm-pdv.is.kkh.dk/atlas/pics/4/4-145-4.jpg
https://danderm-pdv.is.kkh.dk/atlas/pics/4/4-146.jpg
https://danderm-pdv.is.kkh.dk/atlas/pics/4/4-146-1.jpg
https://danderm-pdv.is.kkh.dk/atlas/pics/4/4-146-2.jpg
https://danderm-pdv.is.kkh.dk/atlas/pics/4/4-146-3.jpg
https://danderm-pdv.is.kkh.dk/atlas/pics/4/4-146-4.jpg
https://danderm-pdv.is.kkh.dk/atlas/pics/4/4-146-5.jpg
https://danderm-pdv.is.kkh.dk/atlas/pics/4/4-146-6.jpg
https://danderm-pdv.is.kkh.dk/atlas/pics/4/4-146-7.jpg
https://danderm-pdv.is.kkh.dk/atlas/pics/4/4-146-8.jpg
https://danderm-pdv.is.kkh.dk/atlas/pics/4/4-146-9.jpg
https://danderm-pdv.is.kkh.dk/atlas/pics/4/4-146-10.jpg
https://danderm-pdv.is.kkh.dk/atlas/pics/4/4-146-11.jpg
https://danderm-pdv.is.kkh.dk/atlas/pics/4/4-147_S.jpg
https://danderm-pdv.is.kkh.dk/atlas/pics/4/4-148_S.jpg
https://danderm-pdv.is.kkh.dk/atlas/pics/4/4-149_S.jpg
https://danderm-pdv.is.kkh.dk/atlas/pics/4/4-150_S.jpg
https://danderm-pdv.is.kkh.dk/atlas/pics/4/4-150-1.jpg
https://danderm-pdv.is.kkh.dk/atlas/pics/4/4-151_S.jpg
https://danderm-pdv.is.kkh.dk/atlas/pics/4/4-152.jpg
https://danderm-pdv.is.kkh.dk/atlas/pics/4/4-153.jpg
https://danderm-pdv.is.kkh.dk/atlas/pics/4/4-154.jpg
https://danderm-pdv.is.kkh.dk/atlas/pics/4/4-155.jpg
https://danderm-pdv.is.kkh.dk/atlas/pics/4/4-155-1.jpg
https://danderm-pdv.is.kkh.dk/atlas/pics/4/4-155-2.jpg
https://danderm-pdv.is.kkh.dk/atlas/pics/4/4-155-3.jpg
https://danderm-pdv.is.kkh.dk/atlas/pics/4/4-156.jpg
https://danderm-pdv.is.kkh.dk/atlas/pics/4/4-157.jpg
https://danderm-pdv.is.kkh.dk/atlas/pics/4/4-158.jpg
https://danderm-pdv.is.kkh.dk/atlas/pics/4/4-158-1.jpg
https://danderm-pdv.is.kkh.dk/atlas/pics/4/4-158-2.jpg
https://danderm-pdv.is.kkh.dk/atlas/pics/4/4-158-3.jpg
https://danderm-pdv.is.kkh.dk/atlas/pics/4/4-158-4.jpg
https://danderm-pdv.is.kkh.dk/atlas/pics/4/4-158-5.jpg
https://danderm-pdv.is.kkh.dk/atlas/pics/4/4-158-6.jpg
https://danderm-pdv.is.kkh.dk/atlas/pics/4/4-158-7.jpg
https://danderm-pdv.is.kkh.dk/atlas/pics/4/4-158-8.jpg
https://danderm-pdv.is.kkh.dk/atlas/pics/4/4-159.jpg
https://danderm-pdv.is.kkh.dk/atlas/pics/4/4-160.jpg
https://danderm-pdv.is.kkh.dk/atlas/pics/4/4-160-1.jpg
https://danderm-pdv.is.kkh.dk/atlas/pics/4/4-160-2.jpg
https://danderm-pdv.is.kkh.dk/atlas/pics/4/4-160-3.jpg
https://danderm-pdv.is.kkh.dk/atlas/pics/4/4-160-4.jpg
https://danderm-pdv.is.kkh.dk/atlas/pics/4/4-160-5.jpg
https://danderm-pdv.is.kkh.dk/atlas/pics/4/4-161_S.jpg
https://danderm-pdv.is.kkh.dk/atlas/pics/4/4-161-1.jpg

https://danderm-pdv.is.kkh.dk/atlas/pics/4/4-161-1a.jpg
https://danderm-pdv.is.kkh.dk/atlas/pics/4/4-161-2.jpg
https://danderm-pdv.is.kkh.dk/atlas/pics/4/4-161-3.jpg
https://danderm-pdv.is.kkh.dk/atlas/pics/4/4-162.jpg
https://danderm-pdv.is.kkh.dk/atlas/pics/4/4-162-1.jpg
https://danderm-pdv.is.kkh.dk/atlas/pics/4/4-162-2.jpg
https://danderm-pdv.is.kkh.dk/atlas/pics/4/4-162-3.jpg
https://danderm-pdv.is.kkh.dk/atlas/pics/4/4-163.jpg
https://danderm-pdv.is.kkh.dk/atlas/pics/4/4-164_S.jpg
https://danderm-pdv.is.kkh.dk/atlas/pics/4/4-165.jpg
https://danderm-pdv.is.kkh.dk/atlas/pics/4/4-165-1.jpg
https://danderm-pdv.is.kkh.dk/atlas/pics/4/4-165-2.jpg
https://danderm-pdv.is.kkh.dk/atlas/pics/4/4-166.jpg
https://danderm-pdv.is.kkh.dk/atlas/pics/4/4-166-1.jpg
https://danderm-pdv.is.kkh.dk/atlas/pics/4/4-166-2.jpg
https://danderm-pdv.is.kkh.dk/atlas/pics/4/4-169.jpg
https://danderm-pdv.is.kkh.dk/atlas/pics/4/4-170.jpg
https://danderm-pdv.is.kkh.dk/atlas/pics/4/4-170-1.jpg
https://danderm-pdv.is.kkh.dk/atlas/pics/4/4-170-2.jpg
https://danderm-pdv.is.kkh.dk/atlas/pics/4/4-170-3.jpg
https://danderm-pdv.is.kkh.dk/atlas/pics/4/4-171.jpg
https://danderm-pdv.is.kkh.dk/atlas/pics/4/4-172.jpg
https://danderm-pdv.is.kkh.dk/atlas/pics/4/4-173.jpg
https://danderm-pdv.is.kkh.dk/atlas/pics/4/4-174.jpg
https://danderm-pdv.is.kkh.dk/atlas/pics/4/4-175.jpg
https://danderm-pdv.is.kkh.dk/atlas/pics/4/4-175-1.jpg
https://danderm-pdv.is.kkh.dk/atlas/pics/4/4-175-2.jpg
https://danderm-pdv.is.kkh.dk/atlas/pics/4/4-175-3.jpg
https://danderm-pdv.is.kkh.dk/atlas/pics/4/4-175-4.jpg
https://danderm-pdv.is.kkh.dk/atlas/pics/4/4-175-5.jpg
https://danderm-pdv.is.kkh.dk/atlas/pics/4/4-175-6.jpg
https://danderm-pdv.is.kkh.dk/atlas/pics/4/4-176.jpg
https://danderm-pdv.is.kkh.dk/atlas/pics/5/5-1.jpg
https://danderm-pdv.is.kkh.dk/atlas/pics/5/5-1-1.jpg
https://danderm-pdv.is.kkh.dk/atlas/pics/5/5-1-2.jpg
https://danderm-pdv.is.kkh.dk/atlas/pics/5/5-1-3.jpg
https://danderm-pdv.is.kkh.dk/atlas/pics/5/5-1-4.jpg
https://danderm-pdv.is.kkh.dk/atlas/pics/5/5-1-5.jpg
https://danderm-pdv.is.kkh.dk/atlas/pics/5/5-1-6.jpg
https://danderm-pdv.is.kkh.dk/atlas/pics/5/5-1-7.jpg
https://danderm-pdv.is.kkh.dk/atlas/pics/5/5-1-8.jpg
https://danderm-pdv.is.kkh.dk/atlas/pics/5/5-2-slor.jpg
https://danderm-pdv.is.kkh.dk/atlas/pics/5/5-3.jpg
https://danderm-pdv.is.kkh.dk/atlas/pics/5/5-4.jpg
https://danderm-pdv.is.kkh.dk/atlas/pics/5/5-4-1.jpg
https://danderm-pdv.is.kkh.dk/atlas/pics/5/5-4-2.jpg
https://danderm-pdv.is.kkh.dk/atlas/pics/5/5-4-3.jpg
https://danderm-pdv.is.kkh.dk/atlas/pics/5/5-4-4.jpg

https://danderm-pdv.is.kkh.dk/atlas/pics/5/5-5.jpg
https://danderm-pdv.is.kkh.dk/atlas/pics/5/5-6.jpg
https://danderm-pdv.is.kkh.dk/atlas/pics/5/5-7.jpg
https://danderm-pdv.is.kkh.dk/atlas/pics/5/5-8.jpg
https://danderm-pdv.is.kkh.dk/atlas/pics/5/5-8-2.jpg
https://danderm-pdv.is.kkh.dk/atlas/pics/5/5-9.jpg
https://danderm-pdv.is.kkh.dk/atlas/pics/5/5-10.jpg
https://danderm-pdv.is.kkh.dk/atlas/pics/5/5-11.jpg
https://danderm-pdv.is.kkh.dk/atlas/pics/5/5-12.jpg
https://danderm-pdv.is.kkh.dk/atlas/pics/5/5-13.jpg
https://danderm-pdv.is.kkh.dk/atlas/pics/5/5-14.jpg
https://danderm-pdv.is.kkh.dk/atlas/pics/5/5-14-1.jpg
https://danderm-pdv.is.kkh.dk/atlas/pics/5/5-14-2.jpg
https://danderm-pdv.is.kkh.dk/atlas/pics/5/5-14-3.jpg
https://danderm-pdv.is.kkh.dk/atlas/pics/5/5-14-4.jpg
https://danderm-pdv.is.kkh.dk/atlas/pics/5/5-14-5.jpg
https://danderm-pdv.is.kkh.dk/atlas/pics/5/5-14-6.jpg
https://danderm-pdv.is.kkh.dk/atlas/pics/5/5-14-7.jpg
https://danderm-pdv.is.kkh.dk/atlas/pics/5/5-14-8.jpg
https://danderm-pdv.is.kkh.dk/atlas/pics/5/5-14-9.jpg
https://danderm-pdv.is.kkh.dk/atlas/pics/5/5-14-10.jpg
https://danderm-pdv.is.kkh.dk/atlas/pics/5/5-14-11.jpg
https://danderm-pdv.is.kkh.dk/atlas/pics/5/5-14-12.jpg
https://danderm-pdv.is.kkh.dk/atlas/pics/5/5-14-13.jpg
https://danderm-pdv.is.kkh.dk/atlas/pics/5/5-14-14.jpg
https://danderm-pdv.is.kkh.dk/atlas/pics/5/5-14-15.jpg
https://danderm-pdv.is.kkh.dk/atlas/pics/5/5-14-16.jpg
https://danderm-pdv.is.kkh.dk/atlas/pics/5/5-15.jpg
https://danderm-pdv.is.kkh.dk/atlas/pics/5/5-15-1.jpg
https://danderm-pdv.is.kkh.dk/atlas/pics/5/5-15-2.jpg
https://danderm-pdv.is.kkh.dk/atlas/pics/5/5-15-3.jpg
https://danderm-pdv.is.kkh.dk/atlas/pics/5/5-16.jpg
https://danderm-pdv.is.kkh.dk/atlas/pics/5/5-17.jpg
https://danderm-pdv.is.kkh.dk/atlas/pics/5/5-17-1.jpg
https://danderm-pdv.is.kkh.dk/atlas/pics/5/5-17-2.jpg
https://danderm-pdv.is.kkh.dk/atlas/pics/5/5-17-3.jpg
https://danderm-pdv.is.kkh.dk/atlas/pics/5/5-17-4.jpg
https://danderm-pdv.is.kkh.dk/atlas/pics/5/5-17-5.jpg
https://danderm-pdv.is.kkh.dk/atlas/pics/5/5-17-6.jpg
https://danderm-pdv.is.kkh.dk/atlas/pics/5/5-17-7.jpg
https://danderm-pdv.is.kkh.dk/atlas/pics/5/5-17-8.jpg
https://danderm-pdv.is.kkh.dk/atlas/pics/5/5-17-9.jpg
https://danderm-pdv.is.kkh.dk/atlas/pics/5/5-17-10.jpg
https://danderm-pdv.is.kkh.dk/atlas/pics/5/5-17-11.jpg
https://danderm-pdv.is.kkh.dk/atlas/pics/5/5-17-12.jpg
https://danderm-pdv.is.kkh.dk/atlas/pics/5/5-18.jpg
https://danderm-pdv.is.kkh.dk/atlas/pics/5/5-18-1.jpg
https://danderm-pdv.is.kkh.dk/atlas/pics/5/5-18-2.jpg

https://danderm-pdv.is.kkh.dk/atlas/pics/5/5-18-3.jpg
https://danderm-pdv.is.kkh.dk/atlas/pics/5/5-18-4.jpg
https://danderm-pdv.is.kkh.dk/atlas/pics/5/5-19.jpg
https://danderm-pdv.is.kkh.dk/atlas/pics/5/5-19-1.jpg
https://danderm-pdv.is.kkh.dk/atlas/pics/5/5-19-2.jpg
https://danderm-pdv.is.kkh.dk/atlas/pics/5/5-19-3.jpg
https://danderm-pdv.is.kkh.dk/atlas/pics/5/5-20.jpg
https://danderm-pdv.is.kkh.dk/atlas/pics/5/5-20-1.jpg
https://danderm-pdv.is.kkh.dk/atlas/pics/5/5-21.jpg
https://danderm-pdv.is.kkh.dk/atlas/pics/5/5-22.jpg
https://danderm-pdv.is.kkh.dk/atlas/pics/5/5-23.jpg
https://danderm-pdv.is.kkh.dk/atlas/pics/5/5-23-1.jpg
https://danderm-pdv.is.kkh.dk/atlas/pics/5/5-23-2.jpg
https://danderm-pdv.is.kkh.dk/atlas/pics/5/5-24-slor.jpg
https://danderm-pdv.is.kkh.dk/atlas/pics/5/5-24-1.jpg
https://danderm-pdv.is.kkh.dk/atlas/pics/5/5-24-2.jpg
https://danderm-pdv.is.kkh.dk/atlas/pics/5/5-24-3.jpg
https://danderm-pdv.is.kkh.dk/atlas/pics/5/5-25-slor.jpg
https://danderm-pdv.is.kkh.dk/atlas/pics/5/5-26.jpg
https://danderm-pdv.is.kkh.dk/atlas/pics/5/5-27.jpg
https://danderm-pdv.is.kkh.dk/atlas/pics/5/5-27-1.jpg
https://danderm-pdv.is.kkh.dk/atlas/pics/5/5-27-2.jpg
https://danderm-pdv.is.kkh.dk/atlas/pics/5/5-27-3.jpg
https://danderm-pdv.is.kkh.dk/atlas/pics/5/5-27-4.jpg
https://danderm-pdv.is.kkh.dk/atlas/pics/5/5-27-5.jpg
https://danderm-pdv.is.kkh.dk/atlas/pics/5/5-27-6.jpg
https://danderm-pdv.is.kkh.dk/atlas/pics/5/5-28-slor.jpg
https://danderm-pdv.is.kkh.dk/atlas/pics/5/5-29.jpg
https://danderm-pdv.is.kkh.dk/atlas/pics/5/5-30.jpg
https://danderm-pdv.is.kkh.dk/atlas/pics/5/5-31.jpg
https://danderm-pdv.is.kkh.dk/atlas/pics/5/5-32.jpg
https://danderm-pdv.is.kkh.dk/atlas/pics/5/5-32-1.jpg
https://danderm-pdv.is.kkh.dk/atlas/pics/5/5-32-2.jpg
https://danderm-pdv.is.kkh.dk/atlas/pics/5/5-32-3.jpg
https://danderm-pdv.is.kkh.dk/atlas/pics/5/5-32-4.jpg
https://danderm-pdv.is.kkh.dk/atlas/pics/5/5-32-5.jpg
https://danderm-pdv.is.kkh.dk/atlas/pics/5/5-32-6.jpg
https://danderm-pdv.is.kkh.dk/atlas/pics/5/5-32-7.jpg
https://danderm-pdv.is.kkh.dk/atlas/pics/5/5-32-8.jpg
https://danderm-pdv.is.kkh.dk/atlas/pics/5/5-33.jpg
https://danderm-pdv.is.kkh.dk/atlas/pics/5/5-34.jpg
https://danderm-pdv.is.kkh.dk/atlas/pics/5/5-35.jpg
https://danderm-pdv.is.kkh.dk/atlas/pics/5/5-35-1.jpg
https://danderm-pdv.is.kkh.dk/atlas/pics/5/5-35-2.jpg
https://danderm-pdv.is.kkh.dk/atlas/pics/5/5-35-3.jpg
https://danderm-pdv.is.kkh.dk/atlas/pics/5/5-35-4.jpg
https://danderm-pdv.is.kkh.dk/atlas/pics/5/5-35-5.jpg
https://danderm-pdv.is.kkh.dk/atlas/pics/5/5-36.jpg

https://danderm-pdv.is.kkh.dk/atlas/pics/5/5-37.jpg
https://danderm-pdv.is.kkh.dk/atlas/pics/5/5-39-1.jpg
https://danderm-pdv.is.kkh.dk/atlas/pics/5/5-39-2.jpg
https://danderm-pdv.is.kkh.dk/atlas/pics/5/5-39-3.jpg
https://danderm-pdv.is.kkh.dk/atlas/pics/5/5-40-1.jpg
https://danderm-pdv.is.kkh.dk/atlas/pics/5/5-40-2.jpg
https://danderm-pdv.is.kkh.dk/atlas/pics/5/5-40-3.jpg
https://danderm-pdv.is.kkh.dk/atlas/pics/5/5-40-4.jpg
https://danderm-pdv.is.kkh.dk/atlas/pics/5/5-41.jpg
https://danderm-pdv.is.kkh.dk/atlas/pics/5/5-42.jpg
https://danderm-pdv.is.kkh.dk/atlas/pics/5/5-43.jpg
https://danderm-pdv.is.kkh.dk/atlas/pics/5/5-43-1.jpg
https://danderm-pdv.is.kkh.dk/atlas/pics/5/5-43-2.jpg
https://danderm-pdv.is.kkh.dk/atlas/pics/5/5-43-3.jpg
https://danderm-pdv.is.kkh.dk/atlas/pics/5/5-43-4.jpg
https://danderm-pdv.is.kkh.dk/atlas/pics/5/5-43-5.jpg
https://danderm-pdv.is.kkh.dk/atlas/pics/5/5-43-6.jpg
https://danderm-pdv.is.kkh.dk/atlas/pics/5/5-43-7.jpg
https://danderm-pdv.is.kkh.dk/atlas/pics/5/5-43-8.jpg
https://danderm-pdv.is.kkh.dk/atlas/pics/5/5-44.jpg
https://danderm-pdv.is.kkh.dk/atlas/pics/5/5-45.jpg
https://danderm-pdv.is.kkh.dk/atlas/pics/5/5-46.jpg
https://danderm-pdv.is.kkh.dk/atlas/pics/5/5-46-1.jpg
https://danderm-pdv.is.kkh.dk/atlas/pics/5/5-46-2.jpg
https://danderm-pdv.is.kkh.dk/atlas/pics/5/5-46-3.jpg
https://danderm-pdv.is.kkh.dk/atlas/pics/5/5-46-4.jpg
https://danderm-pdv.is.kkh.dk/atlas/pics/5/5-46-5.jpg
https://danderm-pdv.is.kkh.dk/atlas/pics/5/5-46-6.jpg
https://danderm-pdv.is.kkh.dk/atlas/pics/5/5-47.jpg
https://danderm-pdv.is.kkh.dk/atlas/pics/5/5-48.jpg
https://danderm-pdv.is.kkh.dk/atlas/pics/5/5-48-1.jpg
https://danderm-pdv.is.kkh.dk/atlas/pics/5/5-48-2.jpg
https://danderm-pdv.is.kkh.dk/atlas/pics/5/5-48-3.jpg
https://danderm-pdv.is.kkh.dk/atlas/pics/5/5-48-4.jpg
https://danderm-pdv.is.kkh.dk/atlas/pics/5/5-48-5.jpg
https://danderm-pdv.is.kkh.dk/atlas/pics/5/5-48-6.jpg
https://danderm-pdv.is.kkh.dk/atlas/pics/5/5-48-7.jpg
https://danderm-pdv.is.kkh.dk/atlas/pics/5/5-49.jpg
https://danderm-pdv.is.kkh.dk/atlas/pics/5/5-50.jpg
https://danderm-pdv.is.kkh.dk/atlas/pics/5/5-50-1.jpg
https://danderm-pdv.is.kkh.dk/atlas/pics/5/5-50-2.jpg
https://danderm-pdv.is.kkh.dk/atlas/pics/5/5-50-3.jpg
https://danderm-pdv.is.kkh.dk/atlas/pics/5/5-50-5.jpg
https://danderm-pdv.is.kkh.dk/atlas/pics/5/5-50-4.jpg
https://danderm-pdv.is.kkh.dk/atlas/pics/5/5-50-6.jpg
https://danderm-pdv.is.kkh.dk/atlas/pics/5/5-50-7.jpg
https://danderm-pdv.is.kkh.dk/atlas/pics/5/5-50-8.jpg
https://danderm-pdv.is.kkh.dk/atlas/pics/5/5-50-9.jpg

https://danderm-pdv.is.kkh.dk/atlas/pics/5/5-50-10.jpg
https://danderm-pdv.is.kkh.dk/atlas/pics/5/5-51.jpg
https://danderm-pdv.is.kkh.dk/atlas/pics/5/5-52.jpg
https://danderm-pdv.is.kkh.dk/atlas/pics/5/5-53.jpg
https://danderm-pdv.is.kkh.dk/atlas/pics/5/5-53-1.jpg
https://danderm-pdv.is.kkh.dk/atlas/pics/5/5-53-2.jpg
https://danderm-pdv.is.kkh.dk/atlas/pics/5/5-53-3.jpg
https://danderm-pdv.is.kkh.dk/atlas/pics/5/5-54.jpg
https://danderm-pdv.is.kkh.dk/atlas/pics/5/5-55.jpg
https://danderm-pdv.is.kkh.dk/atlas/pics/5/5-55-1.jpg
https://danderm-pdv.is.kkh.dk/atlas/pics/5/5-55-2.jpg
https://danderm-pdv.is.kkh.dk/atlas/pics/5/5-55-3.jpg
https://danderm-pdv.is.kkh.dk/atlas/pics/5/5-55-4.jpg
https://danderm-pdv.is.kkh.dk/atlas/pics/5/5-55-5.jpg
https://danderm-pdv.is.kkh.dk/atlas/pics/5/5-55-6.jpg
https://danderm-pdv.is.kkh.dk/atlas/pics/5/5-56-slor.jpg
https://danderm-pdv.is.kkh.dk/atlas/pics/5/5-57.jpg
https://danderm-pdv.is.kkh.dk/atlas/pics/5/5-58.jpg
https://danderm-pdv.is.kkh.dk/atlas/pics/5/5-59.jpg
https://danderm-pdv.is.kkh.dk/atlas/pics/5/5-60.jpg
https://danderm-pdv.is.kkh.dk/atlas/pics/5/5-61.jpg
https://danderm-pdv.is.kkh.dk/atlas/pics/5/5-61-1.jpg
https://danderm-pdv.is.kkh.dk/atlas/pics/5/5-61-2.jpg
https://danderm-pdv.is.kkh.dk/atlas/pics/5/5-61-3.jpg
https://danderm-pdv.is.kkh.dk/atlas/pics/5/5-62.jpg
https://danderm-pdv.is.kkh.dk/atlas/pics/5/5-63.jpg
https://danderm-pdv.is.kkh.dk/atlas/pics/5/5-63-1 .jpg
https://danderm-pdv.is.kkh.dk/atlas/pics/5/5-63-2.jpg
https://danderm-pdv.is.kkh.dk/atlas/pics/5/5-63-3.jpg
https://danderm-pdv.is.kkh.dk/atlas/pics/5/5-63-4 .jpg
https://danderm-pdv.is.kkh.dk/atlas/pics/5/5-63-5.jpg
https://danderm-pdv.is.kkh.dk/atlas/pics/5/5-63-6 .jpg
https://danderm-pdv.is.kkh.dk/atlas/pics/5/5-63-7.jpg
https://danderm-pdv.is.kkh.dk/atlas/pics/5/5-63-8 .jpg
https://danderm-pdv.is.kkh.dk/atlas/pics/5/5-63-9 .jpg
https://danderm-pdv.is.kkh.dk/atlas/pics/5/5-64.jpg
https://danderm-pdv.is.kkh.dk/atlas/pics/5/5-64-1.jpg
https://danderm-pdv.is.kkh.dk/atlas/pics/5/5-65.jpg
https://danderm-pdv.is.kkh.dk/atlas/pics/5/5-65-1.jpg
https://danderm-pdv.is.kkh.dk/atlas/pics/5/5-66.jpg
https://danderm-pdv.is.kkh.dk/atlas/pics/5/5-67-0.jpg
https://danderm-pdv.is.kkh.dk/atlas/pics/5/5-67.jpg
https://danderm-pdv.is.kkh.dk/atlas/pics/5/5-67-2 .jpg
https://danderm-pdv.is.kkh.dk/atlas/pics/5/5-67-3 .jpg
https://danderm-pdv.is.kkh.dk/atlas/pics/5/5-67-4.jpg
https://danderm-pdv.is.kkh.dk/atlas/pics/5/5-67-5.jpg
https://danderm-pdv.is.kkh.dk/atlas/pics/5/5-67-6.jpg
https://danderm-pdv.is.kkh.dk/atlas/pics/5/5-67-7 .jpg

https://danderm-pdv.is.kkh.dk/atlas/pics/5/5-67-7-1.jpg
https://danderm-pdv.is.kkh.dk/atlas/pics/5/5-67-8.jpg
https://danderm-pdv.is.kkh.dk/atlas/pics/5/5-67-9.jpg
https://danderm-pdv.is.kkh.dk/atlas/pics/5/5-67-10.jpg
https://danderm-pdv.is.kkh.dk/atlas/pics/5/5-67-11 .jpg
https://danderm-pdv.is.kkh.dk/atlas/pics/5/5-67-12 .jpg
https://danderm-pdv.is.kkh.dk/atlas/pics/5/5-68.jpg
https://danderm-pdv.is.kkh.dk/atlas/pics/5/5-69.jpg
https://danderm-pdv.is.kkh.dk/atlas/pics/5/5-69-1.jpg
https://danderm-pdv.is.kkh.dk/atlas/pics/5/5-69-2.jpg
https://danderm-pdv.is.kkh.dk/atlas/pics/5/5-69-3.jpg
https://danderm-pdv.is.kkh.dk/atlas/pics/5/5-69-4.jpg
https://danderm-pdv.is.kkh.dk/atlas/pics/5/5-70.jpg
https://danderm-pdv.is.kkh.dk/atlas/pics/5/5-71.jpg
https://danderm-pdv.is.kkh.dk/atlas/pics/5/5-72.jpg
https://danderm-pdv.is.kkh.dk/atlas/pics/5/5-73.jpg
https://danderm-pdv.is.kkh.dk/atlas/pics/5/5-73-1.jpg
https://danderm-pdv.is.kkh.dk/atlas/pics/5/5-73-2.jpg
https://danderm-pdv.is.kkh.dk/atlas/pics/5/5-73-3.jpg
https://danderm-pdv.is.kkh.dk/atlas/pics/5/5-73-4.jpg
https://danderm-pdv.is.kkh.dk/atlas/pics/5/5-73-5.jpg
https://danderm-pdv.is.kkh.dk/atlas/pics/5/5-73-6.jpg
https://danderm-pdv.is.kkh.dk/atlas/pics/5/5-74.jpg
https://danderm-pdv.is.kkh.dk/atlas/pics/5/5-75.jpg
https://danderm-pdv.is.kkh.dk/atlas/pics/5/5-76.jpg
https://danderm-pdv.is.kkh.dk/atlas/pics/5/5-77.jpg
https://danderm-pdv.is.kkh.dk/atlas/pics/5/5-78.jpg
https://danderm-pdv.is.kkh.dk/atlas/pics/5/5-78-1.jpg
https://danderm-pdv.is.kkh.dk/atlas/pics/5/5-78-2.jpg
https://danderm-pdv.is.kkh.dk/atlas/pics/5/5-78-3.jpg
https://danderm-pdv.is.kkh.dk/atlas/pics/5/5-78-4.jpg
https://danderm-pdv.is.kkh.dk/atlas/pics/5/5-78-5.jpg
https://danderm-pdv.is.kkh.dk/atlas/pics/5/5-78-6.jpg
https://danderm-pdv.is.kkh.dk/atlas/pics/5/5-78-7 .jpg
https://danderm-pdv.is.kkh.dk/atlas/pics/5/5-79.jpg
https://danderm-pdv.is.kkh.dk/atlas/pics/5/5-80.jpg
https://danderm-pdv.is.kkh.dk/atlas/pics/5/5-80-1.jpg
https://danderm-pdv.is.kkh.dk/atlas/pics/5/5-80-2.jpg
https://danderm-pdv.is.kkh.dk/atlas/pics/5/5-80-3.jpg
https://danderm-pdv.is.kkh.dk/atlas/pics/5/5-80-4..jpg
https://danderm-pdv.is.kkh.dk/atlas/pics/5/5-80-5.jpg
https://danderm-pdv.is.kkh.dk/atlas/pics/5/5-80-6.jpg
https://danderm-pdv.is.kkh.dk/atlas/pics/5/5-80-7.jpg
https://danderm-pdv.is.kkh.dk/atlas/pics/5/5-81.jpg
https://danderm-pdv.is.kkh.dk/atlas/pics/5/5-81-1.jpg
https://danderm-pdv.is.kkh.dk/atlas/pics/5/5-81-2.jpg
https://danderm-pdv.is.kkh.dk/atlas/pics/5/5-81-3.jpg
https://danderm-pdv.is.kkh.dk/atlas/pics/5/5-81-4.jpg

https://danderm-pdv.is.kkh.dk/atlas/pics/5/5-81-5.jpg
https://danderm-pdv.is.kkh.dk/atlas/pics/5/5-81-6.jpg
https://danderm-pdv.is.kkh.dk/atlas/pics/5/5-81-7.jpg
https://danderm-pdv.is.kkh.dk/atlas/pics/5/5-81-8.jpg
https://danderm-pdv.is.kkh.dk/atlas/pics/5/5-81-10.jpg
https://danderm-pdv.is.kkh.dk/atlas/pics/5/5-81-11.jpg
https://danderm-pdv.is.kkh.dk/atlas/pics/5/5-81-12 .jpg
https://danderm-pdv.is.kkh.dk/atlas/pics/5/5-81-13.jpg
https://danderm-pdv.is.kkh.dk/atlas/pics/5/5-81-14.jpg
https://danderm-pdv.is.kkh.dk/atlas/pics/5/5-81-15.jpg
https://danderm-pdv.is.kkh.dk/atlas/pics/5/5-81-16.jpg
https://danderm-pdv.is.kkh.dk/atlas/pics/5/5-81-17.jpg
https://danderm-pdv.is.kkh.dk/atlas/pics/5/5-82.jpg
https://danderm-pdv.is.kkh.dk/atlas/pics/5/5-83.jpg
https://danderm-pdv.is.kkh.dk/atlas/pics/5/5-83-1.jpg
https://danderm-pdv.is.kkh.dk/atlas/pics/5/5-83-2.jpg
https://danderm-pdv.is.kkh.dk/atlas/pics/5/5-83-3.jpg
https://danderm-pdv.is.kkh.dk/atlas/pics/5/5-83-4.jpg
https://danderm-pdv.is.kkh.dk/atlas/pics/5/5-83-5.jpg
https://danderm-pdv.is.kkh.dk/atlas/pics/5/5-83-6.jpg
https://danderm-pdv.is.kkh.dk/atlas/pics/5/5-83-7.jpg
https://danderm-pdv.is.kkh.dk/atlas/pics/5/5-83-8.jpg
https://danderm-pdv.is.kkh.dk/atlas/pics/5/5-83-9.jpg
https://danderm-pdv.is.kkh.dk/atlas/pics/5/5-83-10.jpg
https://danderm-pdv.is.kkh.dk/atlas/pics/5/5-83-11.jpg
https://danderm-pdv.is.kkh.dk/atlas/pics/5/5-83-12.jpg
https://danderm-pdv.is.kkh.dk/atlas/pics/5/5-83-13.jpg
https://danderm-pdv.is.kkh.dk/atlas/pics/5/5-84.jpg
https://danderm-pdv.is.kkh.dk/atlas/pics/5/5-84-1.jpg
https://danderm-pdv.is.kkh.dk/atlas/pics/5/5-84-2.jpg
https://danderm-pdv.is.kkh.dk/atlas/pics/5/5-85.jpg
https://danderm-pdv.is.kkh.dk/atlas/pics/5/5-85-1.jpg
https://danderm-pdv.is.kkh.dk/atlas/pics/5/5-86.jpg
https://danderm-pdv.is.kkh.dk/atlas/pics/5/5-86-1.jpg
https://danderm-pdv.is.kkh.dk/atlas/pics/5/5-87.jpg
https://danderm-pdv.is.kkh.dk/atlas/pics/5/5-88.jpg
https://danderm-pdv.is.kkh.dk/atlas/pics/5/5-89.jpg
https://danderm-pdv.is.kkh.dk/atlas/pics/5/5-90.jpg
https://danderm-pdv.is.kkh.dk/atlas/pics/5/5-91.jpg
https://danderm-pdv.is.kkh.dk/atlas/pics/5/5-92.jpg
https://danderm-pdv.is.kkh.dk/atlas/pics/5/5-93.jpg
https://danderm-pdv.is.kkh.dk/atlas/pics/5/5-94.jpg
https://danderm-pdv.is.kkh.dk/atlas/pics/5/5-95.jpg
https://danderm-pdv.is.kkh.dk/atlas/pics/5/5-96.jpg
https://danderm-pdv.is.kkh.dk/atlas/pics/5/5-96-1.jpg
https://danderm-pdv.is.kkh.dk/atlas/pics/5/5-97-slor.jpg
https://danderm-pdv.is.kkh.dk/atlas/pics/5/5-98.jpg
https://danderm-pdv.is.kkh.dk/atlas/pics/5/5-99.jpg

https://danderm-pdv.is.kkh.dk/atlas/pics/5/5-100.jpg
https://danderm-pdv.is.kkh.dk/atlas/pics/5/5-100-1.jpg
https://danderm-pdv.is.kkh.dk/atlas/pics/5/5-100-2.jpg
https://danderm-pdv.is.kkh.dk/atlas/pics/5/5-101.jpg
https://danderm-pdv.is.kkh.dk/atlas/pics/5/5-101-1.jpg
https://danderm-pdv.is.kkh.dk/atlas/pics/5/5-101-2.jpg
https://danderm-pdv.is.kkh.dk/atlas/pics/5/5-101-3.jpg
https://danderm-pdv.is.kkh.dk/atlas/pics/5/5-102.jpg
https://danderm-pdv.is.kkh.dk/atlas/pics/5/5-103.jpg
https://danderm-pdv.is.kkh.dk/atlas/pics/5/5-103-1.jpg
https://danderm-pdv.is.kkh.dk/atlas/pics/5/5-103-2.jpg
https://danderm-pdv.is.kkh.dk/atlas/pics/5/5-104.jpg
https://danderm-pdv.is.kkh.dk/atlas/pics/5/5-105.jpg
https://danderm-pdv.is.kkh.dk/atlas/pics/5/5-106.jpg
https://danderm-pdv.is.kkh.dk/atlas/pics/5/5-106-1.jpg
https://danderm-pdv.is.kkh.dk/atlas/pics/5/5-106-2.jpg
https://danderm-pdv.is.kkh.dk/atlas/pics/5/5-106-3.jpg
https://danderm-pdv.is.kkh.dk/atlas/pics/5/5-106-4.jpg
https://danderm-pdv.is.kkh.dk/atlas/pics/5/5-106-5.jpg
https://danderm-pdv.is.kkh.dk/atlas/pics/5/5-106-6.jpg
https://danderm-pdv.is.kkh.dk/atlas/pics/5/5-106-7.jpg
https://danderm-pdv.is.kkh.dk/atlas/pics/5/5-106-8.jpg
https://danderm-pdv.is.kkh.dk/atlas/pics/5/5-107.jpg
https://danderm-pdv.is.kkh.dk/atlas/pics/5/5-108.jpg
https://danderm-pdv.is.kkh.dk/atlas/pics/5/5-109.jpg
https://danderm-pdv.is.kkh.dk/atlas/pics/5/5-110.jpg
https://danderm-pdv.is.kkh.dk/atlas/pics/5/5-110-1.jpg
https://danderm-pdv.is.kkh.dk/atlas/pics/5/5-111.jpg
https://danderm-pdv.is.kkh.dk/atlas/pics/5/5-112.jpg
https://danderm-pdv.is.kkh.dk/atlas/pics/5/5-112-1.jpg
https://danderm-pdv.is.kkh.dk/atlas/pics/5/5-112-2.jpg
https://danderm-pdv.is.kkh.dk/atlas/pics/5/5-112-3.jpg
https://danderm-pdv.is.kkh.dk/atlas/pics/5/5-113.jpg
https://danderm-pdv.is.kkh.dk/atlas/pics/5/5-114.jpg
https://danderm-pdv.is.kkh.dk/atlas/pics/5/5-116-1.jpg
https://danderm-pdv.is.kkh.dk/atlas/pics/5/5-116-2.jpg
https://danderm-pdv.is.kkh.dk/atlas/pics/5/5-116-3.jpg
https://danderm-pdv.is.kkh.dk/atlas/pics/5/5-117.jpg
https://danderm-pdv.is.kkh.dk/atlas/pics/5/5-118.jpg
https://danderm-pdv.is.kkh.dk/atlas/pics/5/5-118-2.jpg
https://danderm-pdv.is.kkh.dk/atlas/pics/5/5-121.jpg
https://danderm-pdv.is.kkh.dk/atlas/pics/5/5-121-1.jpg
https://danderm-pdv.is.kkh.dk/atlas/pics/5/5-121-2.jpg
https://danderm-pdv.is.kkh.dk/atlas/pics/5/5-121-3.jpg
https://danderm-pdv.is.kkh.dk/atlas/pics/5/5-121-4.jpg
https://danderm-pdv.is.kkh.dk/atlas/pics/5/5-121-5.jpg
https://danderm-pdv.is.kkh.dk/atlas/pics/5/5-121-6.jpg
https://danderm-pdv.is.kkh.dk/atlas/pics/5/5-121-7.jpg

https://danderm-pdv.is.kkh.dk/atlas/pics/5/5-121-8.jpg
https://danderm-pdv.is.kkh.dk/atlas/pics/5/5-121-9.jpg
https://danderm-pdv.is.kkh.dk/atlas/pics/5/5-122.jpg
https://danderm-pdv.is.kkh.dk/atlas/pics/5/5-123.jpg
https://danderm-pdv.is.kkh.dk/atlas/pics/5/5-124.jpg
https://danderm-pdv.is.kkh.dk/atlas/pics/5/5-125.jpg
https://danderm-pdv.is.kkh.dk/atlas/pics/5/5-126-slor.jpg
https://danderm-pdv.is.kkh.dk/atlas/pics/5/5-126-1.jpg
https://danderm-pdv.is.kkh.dk/atlas/pics/5/5-127.jpg
https://danderm-pdv.is.kkh.dk/atlas/pics/5/5-128.jpg
https://danderm-pdv.is.kkh.dk/atlas/pics/5/5-128-1.jpg
https://danderm-pdv.is.kkh.dk/atlas/pics/5/5-128-2.jpg
https://danderm-pdv.is.kkh.dk/atlas/pics/5/5-129.jpg
https://danderm-pdv.is.kkh.dk/atlas/pics/5/5-129-1.jpg
https://danderm-pdv.is.kkh.dk/atlas/pics/5/5-129-2.jpg
https://danderm-pdv.is.kkh.dk/atlas/pics/5/5-129-2a.jpg
https://danderm-pdv.is.kkh.dk/atlas/pics/5/5-129-3.jpg
https://danderm-pdv.is.kkh.dk/atlas/pics/5/5-129-3a.jpg
https://danderm-pdv.is.kkh.dk/atlas/pics/5/5-130.jpg
https://danderm-pdv.is.kkh.dk/atlas/pics/5/5-131.jpg
https://danderm-pdv.is.kkh.dk/atlas/pics/5/5-131-1.jpg
https://danderm-pdv.is.kkh.dk/atlas/pics/5/5-131-2.jpg
https://danderm-pdv.is.kkh.dk/atlas/pics/5/5-131-3.jpg
https://danderm-pdv.is.kkh.dk/atlas/pics/5/5-131-4.jpg
https://danderm-pdv.is.kkh.dk/atlas/pics/5/5-131-5.jpg
https://danderm-pdv.is.kkh.dk/atlas/pics/5/5-131-6.jpg
https://danderm-pdv.is.kkh.dk/atlas/pics/5/5-131-7.jpg
https://danderm-pdv.is.kkh.dk/atlas/pics/5/5-131-8.jpg
https://danderm-pdv.is.kkh.dk/atlas/pics/5/5-131-9.jpg
https://danderm-pdv.is.kkh.dk/atlas/pics/5/5-132-slor.jpg
https://danderm-pdv.is.kkh.dk/atlas/pics/5/5-133.jpg
https://danderm-pdv.is.kkh.dk/atlas/pics/5/5-134-slor.jpg
https://danderm-pdv.is.kkh.dk/atlas/pics/5/5-134-1.jpg
https://danderm-pdv.is.kkh.dk/atlas/pics/5/5-135-slor.jpg
https://danderm-pdv.is.kkh.dk/atlas/pics/5/5-136.jpg
https://danderm-pdv.is.kkh.dk/atlas/pics/5/5-136-1.jpg
https://danderm-pdv.is.kkh.dk/atlas/pics/5/5-137.jpg
https://danderm-pdv.is.kkh.dk/atlas/pics/5/5-138.jpg
https://danderm-pdv.is.kkh.dk/atlas/pics/5/5-138-1.jpg
https://danderm-pdv.is.kkh.dk/atlas/pics/5/5-138-2.jpg
https://danderm-pdv.is.kkh.dk/atlas/pics/5/5-138-3.jpg
https://danderm-pdv.is.kkh.dk/atlas/pics/5/5-138-4.jpg
https://danderm-pdv.is.kkh.dk/atlas/pics/5/5-138-5.jpg
https://danderm-pdv.is.kkh.dk/atlas/pics/5/5-138-6.jpg
https://danderm-pdv.is.kkh.dk/atlas/pics/5/5-140.jpg
https://danderm-pdv.is.kkh.dk/atlas/pics/5/5-140-3.jpg
https://danderm-pdv.is.kkh.dk/atlas/pics/5/5-140-4.jpg
https://danderm-pdv.is.kkh.dk/atlas/pics/5/5-140-5.jpg

https://danderm-pdv.is.kkh.dk/atlas/pics/5/5-140-6.jpg
https://danderm-pdv.is.kkh.dk/atlas/pics/5/5-140-7 .jpg
https://danderm-pdv.is.kkh.dk/atlas/pics/6/6-1-slor.jpg
https://danderm-pdv.is.kkh.dk/atlas/pics/6/6-1-1.jpg
https://danderm-pdv.is.kkh.dk/atlas/pics/6/6-1-2.jpg
https://danderm-pdv.is.kkh.dk/atlas/pics/6/6-1-3.jpg
https://danderm-pdv.is.kkh.dk/atlas/pics/6/6-1-4.jpg
https://danderm-pdv.is.kkh.dk/atlas/pics/6/6-1-5.jpg
https://danderm-pdv.is.kkh.dk/atlas/pics/6/6-1-6.jpg
https://danderm-pdv.is.kkh.dk/atlas/pics/6/6-1-7.jpg
https://danderm-pdv.is.kkh.dk/atlas/pics/6/6-1-8.jpg
https://danderm-pdv.is.kkh.dk/atlas/pics/6/6-1-9.jpg
https://danderm-pdv.is.kkh.dk/atlas/pics/6/6-1-10.jpg
https://danderm-pdv.is.kkh.dk/atlas/pics/6/6-1-11.jpg
https://danderm-pdv.is.kkh.dk/atlas/pics/6/6-2-slor.jpg
https://danderm-pdv.is.kkh.dk/atlas/pics/6/6-3.jpg
https://danderm-pdv.is.kkh.dk/atlas/pics/6/6-4.jpg
https://danderm-pdv.is.kkh.dk/atlas/pics/6/6-5.jpg
https://danderm-pdv.is.kkh.dk/atlas/pics/6/6-6.jpg
https://danderm-pdv.is.kkh.dk/atlas/pics/6/6-6-1.jpg
https://danderm-pdv.is.kkh.dk/atlas/pics/6/6-7.jpg
https://danderm-pdv.is.kkh.dk/atlas/pics/6/6-8-slor.jpg
https://danderm-pdv.is.kkh.dk/atlas/pics/6/6-9.jpg
https://danderm-pdv.is.kkh.dk/atlas/pics/6/6-10.jpg
https://danderm-pdv.is.kkh.dk/atlas/pics/6/6-11-slor.jpg
https://danderm-pdv.is.kkh.dk/atlas/pics/6/6-12.jpg
https://danderm-pdv.is.kkh.dk/atlas/pics/6/6-13.jpg
https://danderm-pdv.is.kkh.dk/atlas/pics/6/6-13-1.jpg
https://danderm-pdv.is.kkh.dk/atlas/pics/6/6-13-2.jpg
https://danderm-pdv.is.kkh.dk/atlas/pics/6/6-14.jpg
https://danderm-pdv.is.kkh.dk/atlas/pics/6/6-14-1.jpg
https://danderm-pdv.is.kkh.dk/atlas/pics/6/6-15.jpg
https://danderm-pdv.is.kkh.dk/atlas/pics/6/6-15-1.jpg
https://danderm-pdv.is.kkh.dk/atlas/pics/6/6-15-2.jpg
https://danderm-pdv.is.kkh.dk/atlas/pics/6/6-15-3.jpg
https://danderm-pdv.is.kkh.dk/atlas/pics/6/6-16.jpg
https://danderm-pdv.is.kkh.dk/atlas/pics/6/6-17.jpg
https://danderm-pdv.is.kkh.dk/atlas/pics/6/6-17-1.jpg
https://danderm-pdv.is.kkh.dk/atlas/pics/6/6-17-2.jpg
https://danderm-pdv.is.kkh.dk/atlas/pics/6/6-18-slor.jpg
https://danderm-pdv.is.kkh.dk/atlas/pics/6/6-19.jpg
https://danderm-pdv.is.kkh.dk/atlas/pics/6/6-20-slor.jpg
https://danderm-pdv.is.kkh.dk/atlas/pics/6/6-21.jpg
https://danderm-pdv.is.kkh.dk/atlas/pics/6/6-22.jpg
https://danderm-pdv.is.kkh.dk/atlas/pics/6/6-23-slor.jpg
https://danderm-pdv.is.kkh.dk/atlas/pics/6/6-24.jpg
https://danderm-pdv.is.kkh.dk/atlas/pics/6/6-25.jpg
https://danderm-pdv.is.kkh.dk/atlas/pics/6/6-26-slor.jpg

https://danderm-pdv.is.kkh.dk/atlas/pics/6/6-26-1.jpg
https://danderm-pdv.is.kkh.dk/atlas/pics/6/6-26-2.jpg
https://danderm-pdv.is.kkh.dk/atlas/pics/6/6-26-3.jpg
https://danderm-pdv.is.kkh.dk/atlas/pics/6/6-26-4.jpg
https://danderm-pdv.is.kkh.dk/atlas/pics/6/6-26-5.jpg
https://danderm-pdv.is.kkh.dk/atlas/pics/6/6-27.jpg
https://danderm-pdv.is.kkh.dk/atlas/pics/6/6-27-1.jpg
https://danderm-pdv.is.kkh.dk/atlas/pics/6/6-28.jpg
https://danderm-pdv.is.kkh.dk/atlas/pics/6/6-29.jpg
https://danderm-pdv.is.kkh.dk/atlas/pics/6/6-30.jpg
https://danderm-pdv.is.kkh.dk/atlas/pics/6/6-31.jpg
https://danderm-pdv.is.kkh.dk/atlas/pics/6/6-31-1.jpg
https://danderm-pdv.is.kkh.dk/atlas/pics/6/6-31-2.jpg
https://danderm-pdv.is.kkh.dk/atlas/pics/6/6-31-3.jpg
https://danderm-pdv.is.kkh.dk/atlas/pics/6/6-31-4.jpg
https://danderm-pdv.is.kkh.dk/atlas/pics/6/6-31-5.jpg
https://danderm-pdv.is.kkh.dk/atlas/pics/6/6-32.jpg
https://danderm-pdv.is.kkh.dk/atlas/pics/6/6-33.jpg
https://danderm-pdv.is.kkh.dk/atlas/pics/6/6-33-1.jpg
https://danderm-pdv.is.kkh.dk/atlas/pics/6/6-34.jpg
https://danderm-pdv.is.kkh.dk/atlas/pics/6/6-34-1.jpg
https://danderm-pdv.is.kkh.dk/atlas/pics/6/6-34-2.jpg
https://danderm-pdv.is.kkh.dk/atlas/pics/6/6-35.jpg
https://danderm-pdv.is.kkh.dk/atlas/pics/6/6-36-slor.jpg
https://danderm-pdv.is.kkh.dk/atlas/pics/6/6-37.jpg
https://danderm-pdv.is.kkh.dk/atlas/pics/6/6-37-a.jpg
https://danderm-pdv.is.kkh.dk/atlas/pics/6/6-37-1.jpg
https://danderm-pdv.is.kkh.dk/atlas/pics/6/6-37-2.jpg
https://danderm-pdv.is.kkh.dk/atlas/pics/6/6-37-3.jpg
https://danderm-pdv.is.kkh.dk/atlas/pics/6/6-38.jpg
https://danderm-pdv.is.kkh.dk/atlas/pics/6/6-39.jpg
https://danderm-pdv.is.kkh.dk/atlas/pics/6/6-40.jpg
https://danderm-pdv.is.kkh.dk/atlas/pics/6/6-41.jpg
https://danderm-pdv.is.kkh.dk/atlas/pics/6/6-41-1.jpg
https://danderm-pdv.is.kkh.dk/atlas/pics/6/6-41-2.jpg
https://danderm-pdv.is.kkh.dk/atlas/pics/6/6-42.jpg
https://danderm-pdv.is.kkh.dk/atlas/pics/6/6-43.jpg
https://danderm-pdv.is.kkh.dk/atlas/pics/6/6-44.jpg
https://danderm-pdv.is.kkh.dk/atlas/pics/6/6-45.jpg
https://danderm-pdv.is.kkh.dk/atlas/pics/6/6-46.jpg
https://danderm-pdv.is.kkh.dk/atlas/pics/6/6-46-1.jpg
https://danderm-pdv.is.kkh.dk/atlas/pics/6/6-46-2.jpg
https://danderm-pdv.is.kkh.dk/atlas/pics/6/6-46-3.jpg
https://danderm-pdv.is.kkh.dk/atlas/pics/6/6-46-4.jpg
https://danderm-pdv.is.kkh.dk/atlas/pics/6/6-46-5.jpg
https://danderm-pdv.is.kkh.dk/atlas/pics/6/6-47.jpg
https://danderm-pdv.is.kkh.dk/atlas/pics/6/6-48.jpg
https://danderm-pdv.is.kkh.dk/atlas/pics/6/6-49.jpg

https://danderm-pdv.is.kkh.dk/atlas/pics/6/6-50.jpg
https://danderm-pdv.is.kkh.dk/atlas/pics/6/6-51.jpg
https://danderm-pdv.is.kkh.dk/atlas/pics/6/6-52.jpg
https://danderm-pdv.is.kkh.dk/atlas/pics/6/6-52-1.jpg
https://danderm-pdv.is.kkh.dk/atlas/pics/6/6-52-2.jpg
https://danderm-pdv.is.kkh.dk/atlas/pics/6/6-52-3.jpg
https://danderm-pdv.is.kkh.dk/atlas/pics/6/6-52-4.jpg
https://danderm-pdv.is.kkh.dk/atlas/pics/6/6-52-5.jpg
https://danderm-pdv.is.kkh.dk/atlas/pics/6/6-52-6.jpg
https://danderm-pdv.is.kkh.dk/atlas/pics/6/6-53.jpg
https://danderm-pdv.is.kkh.dk/atlas/pics/6/6-53-1.jpg
https://danderm-pdv.is.kkh.dk/atlas/pics/6/6-53-2.jpg
https://danderm-pdv.is.kkh.dk/atlas/pics/6/6-53-3.jpg
https://danderm-pdv.is.kkh.dk/atlas/pics/6/6-53-4.jpg
https://danderm-pdv.is.kkh.dk/atlas/pics/6/6-54.jpg
https://danderm-pdv.is.kkh.dk/atlas/pics/6/6-55.jpg
https://danderm-pdv.is.kkh.dk/atlas/pics/6/6-56.jpg
https://danderm-pdv.is.kkh.dk/atlas/pics/6/6-57.jpg
https://danderm-pdv.is.kkh.dk/atlas/pics/6/6-58.jpg
https://danderm-pdv.is.kkh.dk/atlas/pics/6/6-58-1.jpg
https://danderm-pdv.is.kkh.dk/atlas/pics/6/6-58-2.jpg
https://danderm-pdv.is.kkh.dk/atlas/pics/6/6-58-3.jpg
https://danderm-pdv.is.kkh.dk/atlas/pics/6/6-58-4.jpg
https://danderm-pdv.is.kkh.dk/atlas/pics/6/6-58-5.jpg
https://danderm-pdv.is.kkh.dk/atlas/pics/6/6-58-6.jpg
https://danderm-pdv.is.kkh.dk/atlas/pics/6/6-58-7.jpg
https://danderm-pdv.is.kkh.dk/atlas/pics/6/6-58-8.jpg
https://danderm-pdv.is.kkh.dk/atlas/pics/6/6-58-9.jpg
https://danderm-pdv.is.kkh.dk/atlas/pics/6/6-58-10.jpg
https://danderm-pdv.is.kkh.dk/atlas/pics/6/6-58-11.jpg
https://danderm-pdv.is.kkh.dk/atlas/pics/6/6-58-12.jpg
https://danderm-pdv.is.kkh.dk/atlas/pics/6/6-58-13.jpg
https://danderm-pdv.is.kkh.dk/atlas/pics/6/6-58-14.jpg
https://danderm-pdv.is.kkh.dk/atlas/pics/6/6-59.jpg
https://danderm-pdv.is.kkh.dk/atlas/pics/6/6-59-1.jpg
https://danderm-pdv.is.kkh.dk/atlas/pics/6/6-59-2.jpg
https://danderm-pdv.is.kkh.dk/atlas/pics/6/6-59-3.jpg
https://danderm-pdv.is.kkh.dk/atlas/pics/6/6-59-4.jpg
https://danderm-pdv.is.kkh.dk/atlas/pics/6/6-60.jpg
https://danderm-pdv.is.kkh.dk/atlas/pics/6/6-60-1.jpg
https://danderm-pdv.is.kkh.dk/atlas/pics/6/6-60-2.jpg
https://danderm-pdv.is.kkh.dk/atlas/pics/6/6-61.jpg
https://danderm-pdv.is.kkh.dk/atlas/pics/6/6-62.jpg
https://danderm-pdv.is.kkh.dk/atlas/pics/6/6-63.jpg
https://danderm-pdv.is.kkh.dk/atlas/pics/6/6-63-1.jpg
https://danderm-pdv.is.kkh.dk/atlas/pics/6/6-63-2.jpg
https://danderm-pdv.is.kkh.dk/atlas/pics/6/6-63-3.jpg
https://danderm-pdv.is.kkh.dk/atlas/pics/6/6-63-4.jpg

https://danderm-pdv.is.kkh.dk/atlas/pics/6/6-64.jpg
https://danderm-pdv.is.kkh.dk/atlas/pics/6/6-64-1.jpg
https://danderm-pdv.is.kkh.dk/atlas/pics/6/6-65.jpg
https://danderm-pdv.is.kkh.dk/atlas/pics/6/6-66.jpg
https://danderm-pdv.is.kkh.dk/atlas/pics/6/6-67.jpg
https://danderm-pdv.is.kkh.dk/atlas/pics/6/6-67-1.jpg
https://danderm-pdv.is.kkh.dk/atlas/pics/6/6-68.jpg
https://danderm-pdv.is.kkh.dk/atlas/pics/6/6-69.jpg
https://danderm-pdv.is.kkh.dk/atlas/pics/6/6-70.jpg
https://danderm-pdv.is.kkh.dk/atlas/pics/6/6-70-1.jpg
https://danderm-pdv.is.kkh.dk/atlas/pics/6/6-70-2.jpg
https://danderm-pdv.is.kkh.dk/atlas/pics/6/6-70-3.jpg
https://danderm-pdv.is.kkh.dk/atlas/pics/6/6-70-4.jpg
https://danderm-pdv.is.kkh.dk/atlas/pics/6/6-70-5.jpg
https://danderm-pdv.is.kkh.dk/atlas/pics/6/6-71.jpg
https://danderm-pdv.is.kkh.dk/atlas/pics/6/6-71-1.jpg
https://danderm-pdv.is.kkh.dk/atlas/pics/6/6-71-2.jpg
https://danderm-pdv.is.kkh.dk/atlas/pics/6/6-72.jpg
https://danderm-pdv.is.kkh.dk/atlas/pics/6/6-73.jpg
https://danderm-pdv.is.kkh.dk/atlas/pics/6/6-73-1.jpg
https://danderm-pdv.is.kkh.dk/atlas/pics/6/6-74.jpg
https://danderm-pdv.is.kkh.dk/atlas/pics/6/6-75.jpg
https://danderm-pdv.is.kkh.dk/atlas/pics/6/6-76.jpg
https://danderm-pdv.is.kkh.dk/atlas/pics/6/6-76-1.jpg
https://danderm-pdv.is.kkh.dk/atlas/pics/6/6-77.jpg
https://danderm-pdv.is.kkh.dk/atlas/pics/6/6-78.jpg
https://danderm-pdv.is.kkh.dk/atlas/pics/6/6-78-1.jpg
https://danderm-pdv.is.kkh.dk/atlas/pics/6/6-78-2.jpg
https://danderm-pdv.is.kkh.dk/atlas/pics/6/6-78-3.jpg
https://danderm-pdv.is.kkh.dk/atlas/pics/6/6-78-4.jpg
https://danderm-pdv.is.kkh.dk/atlas/pics/6/6-79.jpg
https://danderm-pdv.is.kkh.dk/atlas/pics/6/6-80.jpg
https://danderm-pdv.is.kkh.dk/atlas/pics/6/6-81.jpg
https://danderm-pdv.is.kkh.dk/atlas/pics/6/6-82.jpg
https://danderm-pdv.is.kkh.dk/atlas/pics/6/6-83.jpg
https://danderm-pdv.is.kkh.dk/atlas/pics/6/6-84.jpg
https://danderm-pdv.is.kkh.dk/atlas/pics/6/6-85.jpg
https://danderm-pdv.is.kkh.dk/atlas/pics/6/6-85-1.jpg
https://danderm-pdv.is.kkh.dk/atlas/pics/6/6-85-2.jpg
https://danderm-pdv.is.kkh.dk/atlas/pics/6/6-85-3.jpg
https://danderm-pdv.is.kkh.dk/atlas/pics/6/6-86.jpg
https://danderm-pdv.is.kkh.dk/atlas/pics/6/6-87.jpg
https://danderm-pdv.is.kkh.dk/atlas/pics/6/6-88.jpg
https://danderm-pdv.is.kkh.dk/atlas/pics/6/6-89.jpg
https://danderm-pdv.is.kkh.dk/atlas/pics/6/6-90.jpg
https://danderm-pdv.is.kkh.dk/atlas/pics/6/6-90-1.jpg
https://danderm-pdv.is.kkh.dk/atlas/pics/6/6-90-2.jpg
https://danderm-pdv.is.kkh.dk/atlas/pics/6/6-92-1.jpg

https://danderm-pdv.is.kkh.dk/atlas/pics/6/6-92-2.jpg
https://danderm-pdv.is.kkh.dk/atlas/pics/6/6-92-3.jpg
https://danderm-pdv.is.kkh.dk/atlas/pics/6/6-92-4.jpg
https://danderm-pdv.is.kkh.dk/atlas/pics/6/6-93.jpg
https://danderm-pdv.is.kkh.dk/atlas/pics/6/6-94.jpg
https://danderm-pdv.is.kkh.dk/atlas/pics/6/6-95.jpg
https://danderm-pdv.is.kkh.dk/atlas/pics/6/6-96.jpg
https://danderm-pdv.is.kkh.dk/atlas/pics/6/6-97.jpg
https://danderm-pdv.is.kkh.dk/atlas/pics/6/6-98.jpg
https://danderm-pdv.is.kkh.dk/atlas/pics/6/6-98-1.jpg
https://danderm-pdv.is.kkh.dk/atlas/pics/6/6-98-2.jpg
https://danderm-pdv.is.kkh.dk/atlas/pics/6/6-98-3.jpg
https://danderm-pdv.is.kkh.dk/atlas/pics/6/6-98-4.jpg
https://danderm-pdv.is.kkh.dk/atlas/pics/6/6-98-5.jpg
https://danderm-pdv.is.kkh.dk/atlas/pics/6/6-98-6.jpg
https://danderm-pdv.is.kkh.dk/atlas/pics/6/6-98-7.jpg
https://danderm-pdv.is.kkh.dk/atlas/pics/6/6-98-8.jpg
https://danderm-pdv.is.kkh.dk/atlas/pics/6/6-99.jpg
https://danderm-pdv.is.kkh.dk/atlas/pics/6/6-100.jpg
https://danderm-pdv.is.kkh.dk/atlas/pics/6/6-101.jpg
https://danderm-pdv.is.kkh.dk/atlas/pics/6/6-102.jpg
https://danderm-pdv.is.kkh.dk/atlas/pics/6/6-102-1.jpg
https://danderm-pdv.is.kkh.dk/atlas/pics/6/6-102-2.jpg
https://danderm-pdv.is.kkh.dk/atlas/pics/6/6-103.jpg
https://danderm-pdv.is.kkh.dk/atlas/pics/6/6-103-1.jpg
https://danderm-pdv.is.kkh.dk/atlas/pics/6/6-103-2.jpg
https://danderm-pdv.is.kkh.dk/atlas/pics/6/6-103-3.jpg
https://danderm-pdv.is.kkh.dk/atlas/pics/6/6-103-4.jpg
https://danderm-pdv.is.kkh.dk/atlas/pics/6/6-103-5.jpg
https://danderm-pdv.is.kkh.dk/atlas/pics/6/6-103-6.jpg
https://danderm-pdv.is.kkh.dk/atlas/pics/6/6-104.jpg
https://danderm-pdv.is.kkh.dk/atlas/pics/6/6-105.jpg
https://danderm-pdv.is.kkh.dk/atlas/pics/6/6-106-slor.jpg
https://danderm-pdv.is.kkh.dk/atlas/pics/6/6-106-1.jpg
https://danderm-pdv.is.kkh.dk/atlas/pics/6/6-106-2.jpg
https://danderm-pdv.is.kkh.dk/atlas/pics/6/6-106-3.jpg
https://danderm-pdv.is.kkh.dk/atlas/pics/6/6-106-4.jpg
https://danderm-pdv.is.kkh.dk/atlas/pics/6/6-106-5.jpg
https://danderm-pdv.is.kkh.dk/atlas/pics/6/6-107.jpg
https://danderm-pdv.is.kkh.dk/atlas/pics/6/6-108.jpg
https://danderm-pdv.is.kkh.dk/atlas/pics/6/6-108-1.jpg
https://danderm-pdv.is.kkh.dk/atlas/pics/6/6-108-2.jpg
https://danderm-pdv.is.kkh.dk/atlas/pics/6/6-108-3.jpg
https://danderm-pdv.is.kkh.dk/atlas/pics/6/6-109.jpg
https://danderm-pdv.is.kkh.dk/atlas/pics/6/6-110.jpg
https://danderm-pdv.is.kkh.dk/atlas/pics/6/6-110-1.jpg
https://danderm-pdv.is.kkh.dk/atlas/pics/6/6-110-2.jpg
https://danderm-pdv.is.kkh.dk/atlas/pics/6/6-110-3.jpg

https://danderm-pdv.is.kkh.dk/atlas/pics/6/6-110-4.jpg
https://danderm-pdv.is.kkh.dk/atlas/pics/6/6-110-5.jpg
https://danderm-pdv.is.kkh.dk/atlas/pics/6/6-110-6.jpg
https://danderm-pdv.is.kkh.dk/atlas/pics/6/6-110-7.jpg
https://danderm-pdv.is.kkh.dk/atlas/pics/6/6-111.jpg
https://danderm-pdv.is.kkh.dk/atlas/pics/6/6-111-1.jpg
https://danderm-pdv.is.kkh.dk/atlas/pics/6/6-111-2.jpg
https://danderm-pdv.is.kkh.dk/atlas/pics/6/6-112.jpg
https://danderm-pdv.is.kkh.dk/atlas/pics/6/6-112-1.jpg
https://danderm-pdv.is.kkh.dk/atlas/pics/6/6-113.jpg
https://danderm-pdv.is.kkh.dk/atlas/pics/6/6-114.jpg
https://danderm-pdv.is.kkh.dk/atlas/pics/6/6-114-1.jpg
https://danderm-pdv.is.kkh.dk/atlas/pics/6/6-115.jpg
https://danderm-pdv.is.kkh.dk/atlas/pics/6/6-116.jpg
https://danderm-pdv.is.kkh.dk/atlas/pics/6/6-117.jpg
https://danderm-pdv.is.kkh.dk/atlas/pics/6/6-117-1.jpg
https://danderm-pdv.is.kkh.dk/atlas/pics/6/6-118.jpg
https://danderm-pdv.is.kkh.dk/atlas/pics/6/6-119.jpg
https://danderm-pdv.is.kkh.dk/atlas/pics/6/6-120.jpg
https://danderm-pdv.is.kkh.dk/atlas/pics/6/6-121.jpg
https://danderm-pdv.is.kkh.dk/atlas/pics/6/6-121-1.jpg
https://danderm-pdv.is.kkh.dk/atlas/pics/6/6-121-2.jpg
https://danderm-pdv.is.kkh.dk/atlas/pics/6/6-121-3.jpg
https://danderm-pdv.is.kkh.dk/atlas/pics/6/6-122.jpg
https://danderm-pdv.is.kkh.dk/atlas/pics/6/6-123.jpg
https://danderm-pdv.is.kkh.dk/atlas/pics/6/6-124.jpg
https://danderm-pdv.is.kkh.dk/atlas/pics/6/6-124-1.jpg
https://danderm-pdv.is.kkh.dk/atlas/pics/6/6-125.jpg
https://danderm-pdv.is.kkh.dk/atlas/pics/6/6-126.jpg
https://danderm-pdv.is.kkh.dk/atlas/pics/6/6-126-1.jpg
https://danderm-pdv.is.kkh.dk/atlas/pics/6/6-126-2.jpg
https://danderm-pdv.is.kkh.dk/atlas/pics/6/6-127.jpg
https://danderm-pdv.is.kkh.dk/atlas/pics/6/6-128.jpg
https://danderm-pdv.is.kkh.dk/atlas/pics/6/6-129.jpg
https://danderm-pdv.is.kkh.dk/atlas/pics/6/6-129-1.jpg
https://danderm-pdv.is.kkh.dk/atlas/pics/6/6-130.jpg
https://danderm-pdv.is.kkh.dk/atlas/pics/6/6-131.jpg
https://danderm-pdv.is.kkh.dk/atlas/pics/6/6-132.jpg
https://danderm-pdv.is.kkh.dk/atlas/pics/6/6-133.jpg
https://danderm-pdv.is.kkh.dk/atlas/pics/6/6-134.jpg
https://danderm-pdv.is.kkh.dk/atlas/pics/6/6-134-1.jpg
https://danderm-pdv.is.kkh.dk/atlas/pics/6/6-134-2.jpg
https://danderm-pdv.is.kkh.dk/atlas/pics/6/6-134-3.jpg
https://danderm-pdv.is.kkh.dk/atlas/pics/6/6-134-4.jpg
https://danderm-pdv.is.kkh.dk/atlas/pics/6/6-135.jpg
https://danderm-pdv.is.kkh.dk/atlas/pics/6/6-136.jpg
https://danderm-pdv.is.kkh.dk/atlas/pics/6/6-137.jpg
https://danderm-pdv.is.kkh.dk/atlas/pics/6/6-138.jpg

https://danderm-pdv.is.kkh.dk/atlas/pics/6/6-138-1.jpg
https://danderm-pdv.is.kkh.dk/atlas/pics/6/6-138-2.jpg
https://danderm-pdv.is.kkh.dk/atlas/pics/7/7-01.jpg
https://danderm-pdv.is.kkh.dk/atlas/pics/7/7-1-2.jpg
https://danderm-pdv.is.kkh.dk/atlas/pics/7/7-1-3.jpg
https://danderm-pdv.is.kkh.dk/atlas/pics/7/7-1-4.jpg
https://danderm-pdv.is.kkh.dk/atlas/pics/7/7-1-5.jpg
https://danderm-pdv.is.kkh.dk/atlas/pics/7/7-1-6.jpg
https://danderm-pdv.is.kkh.dk/atlas/pics/7/7-1-7.jpg
https://danderm-pdv.is.kkh.dk/atlas/pics/7/7-1-1.jpg
https://danderm-pdv.is.kkh.dk/atlas/pics/7/7-02.jpg
https://danderm-pdv.is.kkh.dk/atlas/pics/7/7-03.jpg
https://danderm-pdv.is.kkh.dk/atlas/pics/7/7-3-1.jpg
https://danderm-pdv.is.kkh.dk/atlas/pics/7/7-3-2.jpg
https://danderm-pdv.is.kkh.dk/atlas/pics/7/7-3-3.jpg
https://danderm-pdv.is.kkh.dk/atlas/pics/7/7-04.jpg
https://danderm-pdv.is.kkh.dk/atlas/pics/7/7-4-1.jpg
https://danderm-pdv.is.kkh.dk/atlas/pics/7/7-4-2.jpg
https://danderm-pdv.is.kkh.dk/atlas/pics/7/7-4-3.jpg
https://danderm-pdv.is.kkh.dk/atlas/pics/7/7-4-4.jpg
https://danderm-pdv.is.kkh.dk/atlas/pics/7/7-4-5.jpg
https://danderm-pdv.is.kkh.dk/atlas/pics/7/7-4-6.jpg
https://danderm-pdv.is.kkh.dk/atlas/pics/7/7-4-7.jpg
https://danderm-pdv.is.kkh.dk/atlas/pics/7/7-4-8.jpg
https://danderm-pdv.is.kkh.dk/atlas/pics/7/7-4-9.jpg
https://danderm-pdv.is.kkh.dk/atlas/pics/7/7-4-9-1.jpg
https://danderm-pdv.is.kkh.dk/atlas/pics/7/7-4-10.jpg
https://danderm-pdv.is.kkh.dk/atlas/pics/7/7-05.jpg
https://danderm-pdv.is.kkh.dk/atlas/pics/7/7-5-1.jpg
https://danderm-pdv.is.kkh.dk/atlas/pics/7/7-5-2.jpg
https://danderm-pdv.is.kkh.dk/atlas/pics/7/7-06.jpg
https://danderm-pdv.is.kkh.dk/atlas/pics/7/7-07.jpg
https://danderm-pdv.is.kkh.dk/atlas/pics/7/7-7-1.jpg
https://danderm-pdv.is.kkh.dk/atlas/pics/7/7-7-2.jpg
https://danderm-pdv.is.kkh.dk/atlas/pics/7/7-7-3.jpg
https://danderm-pdv.is.kkh.dk/atlas/pics/7/7-7-4.jpg
https://danderm-pdv.is.kkh.dk/atlas/pics/7/7-7-5.jpg
https://danderm-pdv.is.kkh.dk/atlas/pics/7/7-7-6.jpg
https://danderm-pdv.is.kkh.dk/atlas/pics/7/7-7-7.jpg
https://danderm-pdv.is.kkh.dk/atlas/pics/7/7-7-13.jpg
https://danderm-pdv.is.kkh.dk/atlas/pics/7/7-7-8.jpg
https://danderm-pdv.is.kkh.dk/atlas/pics/7/7-08.jpg
https://danderm-pdv.is.kkh.dk/atlas/pics/7/7-8-1.jpg
https://danderm-pdv.is.kkh.dk/atlas/pics/7/7-8-2.jpg
https://danderm-pdv.is.kkh.dk/atlas/pics/7/7-8-3.jpg
https://danderm-pdv.is.kkh.dk/atlas/pics/7/7-8-4.jpg
https://danderm-pdv.is.kkh.dk/atlas/pics/7/7-8-5.jpg
https://danderm-pdv.is.kkh.dk/atlas/pics/7/7-8-6.jpg

https://danderm-pdv.is.kkh.dk/atlas/pics/7/7-8-7.jpg
https://danderm-pdv.is.kkh.dk/atlas/pics/7/7-8-10.jpg
https://danderm-pdv.is.kkh.dk/atlas/pics/7/7-8-11.jpg
https://danderm-pdv.is.kkh.dk/atlas/pics/7/7-8-12.jpg
https://danderm-pdv.is.kkh.dk/atlas/pics/7/7-09.jpg
https://danderm-pdv.is.kkh.dk/atlas/pics/7/7-10.jpg
https://danderm-pdv.is.kkh.dk/atlas/pics/7/7-10-1.jpg
https://danderm-pdv.is.kkh.dk/atlas/pics/7/7-10-2.jpg
https://danderm-pdv.is.kkh.dk/atlas/pics/7/7-10-3-.jpg
https://danderm-pdv.is.kkh.dk/atlas/pics/7/7-10-4.jpg
https://danderm-pdv.is.kkh.dk/atlas/pics/7/7-10-5.jpg
https://danderm-pdv.is.kkh.dk/atlas/pics/7/7-10-6.jpg
https://danderm-pdv.is.kkh.dk/atlas/pics/7/7-10-7.jpg
https://danderm-pdv.is.kkh.dk/atlas/pics/7/7-10-8.jpg
https://danderm-pdv.is.kkh.dk/atlas/pics/7/7-11_S.jpg
https://danderm-pdv.is.kkh.dk/atlas/pics/7/7-12.jpg
https://danderm-pdv.is.kkh.dk/atlas/pics/7/7-12-1.jpg
https://danderm-pdv.is.kkh.dk/atlas/pics/7/7-12-2.jpg
https://danderm-pdv.is.kkh.dk/atlas/pics/7/7-12-3.jpg
https://danderm-pdv.is.kkh.dk/atlas/pics/7/7-12-4.jpg
https://danderm-pdv.is.kkh.dk/atlas/pics/7/7-12-5.jpg
https://danderm-pdv.is.kkh.dk/atlas/pics/7/7-12-6.jpg
https://danderm-pdv.is.kkh.dk/atlas/pics/7/7-12-8.jpg
https://danderm-pdv.is.kkh.dk/atlas/pics/7/7-13_S.jpg
https://danderm-pdv.is.kkh.dk/atlas/pics/7/7-14.jpg
https://danderm-pdv.is.kkh.dk/atlas/pics/7/7-14-1.jpg
https://danderm-pdv.is.kkh.dk/atlas/pics/7/7-14-2.jpg
https://danderm-pdv.is.kkh.dk/atlas/pics/7/7-14-3.jpg
https://danderm-pdv.is.kkh.dk/atlas/pics/7/7-14-4.jpg
https://danderm-pdv.is.kkh.dk/atlas/pics/7/7-14-5.jpg
https://danderm-pdv.is.kkh.dk/atlas/pics/7/7-14-6.jpg
https://danderm-pdv.is.kkh.dk/atlas/pics/7/7-15_S.jpg
https://danderm-pdv.is.kkh.dk/atlas/pics/7/7-15-1.jpg
https://danderm-pdv.is.kkh.dk/atlas/pics/7/7-15-2.jpg
https://danderm-pdv.is.kkh.dk/atlas/pics/7/7-15-3.jpg
https://danderm-pdv.is.kkh.dk/atlas/pics/7/7-15-4.jpg
https://danderm-pdv.is.kkh.dk/atlas/pics/7/7-16.jpg
https://danderm-pdv.is.kkh.dk/atlas/pics/7/7-17.jpg
https://danderm-pdv.is.kkh.dk/atlas/pics/7/7-18.jpg
https://danderm-pdv.is.kkh.dk/atlas/pics/7/7-18-1.jpg
https://danderm-pdv.is.kkh.dk/atlas/pics/7/7-18-2.jpg
https://danderm-pdv.is.kkh.dk/atlas/pics/7/7-19_S.jpg
https://danderm-pdv.is.kkh.dk/atlas/pics/7/7-19-1.jpg
https://danderm-pdv.is.kkh.dk/atlas/pics/7/7-19-2.jpg
https://danderm-pdv.is.kkh.dk/atlas/pics/7/7-20.jpg
https://danderm-pdv.is.kkh.dk/atlas/pics/7/7-21.jpg
https://danderm-pdv.is.kkh.dk/atlas/pics/7/7-21-1.jpg
https://danderm-pdv.is.kkh.dk/atlas/pics/7/7-21-2.jpg

https://danderm-pdv.is.kkh.dk/atlas/pics/7/7-21-3.jpg
https://danderm-pdv.is.kkh.dk/atlas/pics/7/7-21-4.jpg
https://danderm-pdv.is.kkh.dk/atlas/pics/7/7-22.jpg
https://danderm-pdv.is.kkh.dk/atlas/pics/7/7-22-1.jpg
https://danderm-pdv.is.kkh.dk/atlas/pics/7/7-22-2.jpg
https://danderm-pdv.is.kkh.dk/atlas/pics/7/7-22-3.jpg
https://danderm-pdv.is.kkh.dk/atlas/pics/7/7-22-4.jpg
https://danderm-pdv.is.kkh.dk/atlas/pics/7/7-23-1.jpg
https://danderm-pdv.is.kkh.dk/atlas/pics/7/7-24_S.jpg
https://danderm-pdv.is.kkh.dk/atlas/pics/7/7-25_S.jpg
https://danderm-pdv.is.kkh.dk/atlas/pics/7/7-26_S.jpg
https://danderm-pdv.is.kkh.dk/atlas/pics/7/7-26-1.jpg
https://danderm-pdv.is.kkh.dk/atlas/pics/7/7-26-2.jpg
https://danderm-pdv.is.kkh.dk/atlas/pics/7/7-26-7.jpg
https://danderm-pdv.is.kkh.dk/atlas/pics/7/7-26-8.jpg
https://danderm-pdv.is.kkh.dk/atlas/pics/7/7-26-9.jpg
https://danderm-pdv.is.kkh.dk/atlas/pics/7/7-27.jpg
https://danderm-pdv.is.kkh.dk/atlas/pics/7/7-27-1.jpg
https://danderm-pdv.is.kkh.dk/atlas/pics/7/7-27-2.jpg
https://danderm-pdv.is.kkh.dk/atlas/pics/7/7-28.jpg
https://danderm-pdv.is.kkh.dk/atlas/pics/7/7-28-1.jpg
https://danderm-pdv.is.kkh.dk/atlas/pics/7/7-29.jpg
https://danderm-pdv.is.kkh.dk/atlas/pics/7/7-29-1.jpg
https://danderm-pdv.is.kkh.dk/atlas/pics/7/7-30.jpg
https://danderm-pdv.is.kkh.dk/atlas/pics/7/7-31.jpg
https://danderm-pdv.is.kkh.dk/atlas/pics/7/7-32.jpg
https://danderm-pdv.is.kkh.dk/atlas/pics/7/7-32-1.jpg
https://danderm-pdv.is.kkh.dk/atlas/pics/7/7-32-2.jpg
https://danderm-pdv.is.kkh.dk/atlas/pics/7/7-32-3.jpg
https://danderm-pdv.is.kkh.dk/atlas/pics/7/7-32-4.jpg
https://danderm-pdv.is.kkh.dk/atlas/pics/7/7-32-5.jpg
https://danderm-pdv.is.kkh.dk/atlas/pics/7/7-33.jpg
https://danderm-pdv.is.kkh.dk/atlas/pics/7/7-33-1.jpg
https://danderm-pdv.is.kkh.dk/atlas/pics/7/7-33-2.jpg
https://danderm-pdv.is.kkh.dk/atlas/pics/7/7-33-3.jpg
https://danderm-pdv.is.kkh.dk/atlas/pics/7/7-33-4.jpg
https://danderm-pdv.is.kkh.dk/atlas/pics/7/7-33-5.jpg
https://danderm-pdv.is.kkh.dk/atlas/pics/7/7-33-6.jpg
https://danderm-pdv.is.kkh.dk/atlas/pics/7/7-33-7.jpg
https://danderm-pdv.is.kkh.dk/atlas/pics/7/7-33-8.jpg
https://danderm-pdv.is.kkh.dk/atlas/pics/7/7-33-9.jpg
https://danderm-pdv.is.kkh.dk/atlas/pics/7/7-33-10.jpg
https://danderm-pdv.is.kkh.dk/atlas/pics/7/7-33-11.jpg
https://danderm-pdv.is.kkh.dk/atlas/pics/7/7-33-12.jpg
https://danderm-pdv.is.kkh.dk/atlas/pics/7/7-33-13 .jpg
https://danderm-pdv.is.kkh.dk/atlas/pics/7/7-33-14.jpg
https://danderm-pdv.is.kkh.dk/atlas/pics/7/7-33-15.jpg
https://danderm-pdv.is.kkh.dk/atlas/pics/7/7-33-16.jpg

https://danderm-pdv.is.kkh.dk/atlas/pics/7/7-33-17.jpg
https://danderm-pdv.is.kkh.dk/atlas/pics/7/7-33-18.jpg
https://danderm-pdv.is.kkh.dk/atlas/pics/7/7-33-19.jpg
https://danderm-pdv.is.kkh.dk/atlas/pics/7/7-33-20.jpg
https://danderm-pdv.is.kkh.dk/atlas/pics/7/7-33-21.jpg
https://danderm-pdv.is.kkh.dk/atlas/pics/7/7-33-22.jpg
https://danderm-pdv.is.kkh.dk/atlas/pics/7/7-33-23.jpg
https://danderm-pdv.is.kkh.dk/atlas/pics/7/7-33-24.jpg
https://danderm-pdv.is.kkh.dk/atlas/pics/7/7-34.jpg
https://danderm-pdv.is.kkh.dk/atlas/pics/7/7-34-1.jpg
https://danderm-pdv.is.kkh.dk/atlas/pics/7/7-34-2.jpg
https://danderm-pdv.is.kkh.dk/atlas/pics/7/7-34-2-1.jpg
https://danderm-pdv.is.kkh.dk/atlas/pics/7/7-34-2-2.jpg
https://danderm-pdv.is.kkh.dk/atlas/pics/7/7-34-2-3.jpg
https://danderm-pdv.is.kkh.dk/atlas/pics/7/7-34-2-4.jpg
https://danderm-pdv.is.kkh.dk/atlas/pics/7/7-34-3.jpg
https://danderm-pdv.is.kkh.dk/atlas/pics/7/7-34-4.jpg
https://danderm-pdv.is.kkh.dk/atlas/pics/7/7-34-5.jpg
https://danderm-pdv.is.kkh.dk/atlas/pics/7/7-34-6.jpg
https://danderm-pdv.is.kkh.dk/atlas/pics/7/7-34-7.jpg
https://danderm-pdv.is.kkh.dk/atlas/pics/7/7-34-8.jpg
https://danderm-pdv.is.kkh.dk/atlas/pics/7/7-35.jpg
https://danderm-pdv.is.kkh.dk/atlas/pics/7/7-35-1.jpg
https://danderm-pdv.is.kkh.dk/atlas/pics/7/7-36.jpg
https://danderm-pdv.is.kkh.dk/atlas/pics/7/7-36-1.jpg
https://danderm-pdv.is.kkh.dk/atlas/pics/7/7-36-2.jpg
https://danderm-pdv.is.kkh.dk/atlas/pics/7/7-36-3.jpg
https://danderm-pdv.is.kkh.dk/atlas/pics/7/7-36-4.jpg
https://danderm-pdv.is.kkh.dk/atlas/pics/7/7-37_S.jpg
https://danderm-pdv.is.kkh.dk/atlas/pics/7/7-38.jpg
https://danderm-pdv.is.kkh.dk/atlas/pics/7/7-39.jpg
https://danderm-pdv.is.kkh.dk/atlas/pics/7/7-39-2.jpg
https://danderm-pdv.is.kkh.dk/atlas/pics/7/7-39-3.jpg
https://danderm-pdv.is.kkh.dk/atlas/pics/7/7-39-4.jpg
https://danderm-pdv.is.kkh.dk/atlas/pics/7/7-39-5.jpg
https://danderm-pdv.is.kkh.dk/atlas/pics/7/7-39-6.jpg
https://danderm-pdv.is.kkh.dk/atlas/pics/7/7-39-7.jpg
https://danderm-pdv.is.kkh.dk/atlas/pics/7/7-39-8.jpg
https://danderm-pdv.is.kkh.dk/atlas/pics/7/7-39-9.jpg
https://danderm-pdv.is.kkh.dk/atlas/pics/7/7-39-10.jpg
https://danderm-pdv.is.kkh.dk/atlas/pics/7/7-39-11.jpg
https://danderm-pdv.is.kkh.dk/atlas/pics/7/7-39-12.jpg
https://danderm-pdv.is.kkh.dk/atlas/pics/7/7-40.jpg
https://danderm-pdv.is.kkh.dk/atlas/pics/7/7-41.jpg
https://danderm-pdv.is.kkh.dk/atlas/pics/7/7-42.jpg
https://danderm-pdv.is.kkh.dk/atlas/pics/7/7-42-1.jpg
https://danderm-pdv.is.kkh.dk/atlas/pics/7/7-43.jpg
https://danderm-pdv.is.kkh.dk/atlas/pics/7/7-43-1.jpg

https://danderm-pdv.is.kkh.dk/atlas/pics/7/7-43-2.jpg
https://danderm-pdv.is.kkh.dk/atlas/pics/7/7-44.jpg
https://danderm-pdv.is.kkh.dk/atlas/pics/7/7-45.jpg
https://danderm-pdv.is.kkh.dk/atlas/pics/7/7-45-1.jpg
https://danderm-pdv.is.kkh.dk/atlas/pics/7/7-46.jpg
https://danderm-pdv.is.kkh.dk/atlas/pics/7/7-47-1.jpg
https://danderm-pdv.is.kkh.dk/atlas/pics/7/7-47-2.jpg
https://danderm-pdv.is.kkh.dk/atlas/pics/7/7-47-3.jpg
https://danderm-pdv.is.kkh.dk/atlas/pics/7/7-47-4.jpg
https://danderm-pdv.is.kkh.dk/atlas/pics/7/7-47-5.jpg
https://danderm-pdv.is.kkh.dk/atlas/pics/7/7-47-6.jpg
https://danderm-pdv.is.kkh.dk/atlas/pics/7/7-48.jpg
https://danderm-pdv.is.kkh.dk/atlas/pics/7/7-49.jpg
https://danderm-pdv.is.kkh.dk/atlas/pics/7/7-50.jpg
https://danderm-pdv.is.kkh.dk/atlas/pics/7/7-50-1.jpg
https://danderm-pdv.is.kkh.dk/atlas/pics/7/7-50-2.jpg
https://danderm-pdv.is.kkh.dk/atlas/pics/7/7-51.jpg
https://danderm-pdv.is.kkh.dk/atlas/pics/7/7-52.jpg
https://danderm-pdv.is.kkh.dk/atlas/pics/7/7-53.jpg
https://danderm-pdv.is.kkh.dk/atlas/pics/7/7-53-1.jpg
https://danderm-pdv.is.kkh.dk/atlas/pics/7/7-53-2.jpg
https://danderm-pdv.is.kkh.dk/atlas/pics/7/7-54.jpg
https://danderm-pdv.is.kkh.dk/atlas/pics/7/7-55.jpg
https://danderm-pdv.is.kkh.dk/atlas/pics/7/7-55-1.jpg
https://danderm-pdv.is.kkh.dk/atlas/pics/7/7-55-2.jpg
https://danderm-pdv.is.kkh.dk/atlas/pics/7/7-55-3.jpg
https://danderm-pdv.is.kkh.dk/atlas/pics/7/7-56.jpg
https://danderm-pdv.is.kkh.dk/atlas/pics/7/7-56-1.jpg
https://danderm-pdv.is.kkh.dk/atlas/pics/7/7-56-2.jpg
https://danderm-pdv.is.kkh.dk/atlas/pics/7/7-57.jpg
https://danderm-pdv.is.kkh.dk/atlas/pics/7/7-58.jpg
https://danderm-pdv.is.kkh.dk/atlas/pics/7/7-58-1.jpg
https://danderm-pdv.is.kkh.dk/atlas/pics/7/7-58-2.jpg
https://danderm-pdv.is.kkh.dk/atlas/pics/7/7-58-3.jpg
https://danderm-pdv.is.kkh.dk/atlas/pics/7/7-58-4.jpg
https://danderm-pdv.is.kkh.dk/atlas/pics/7/7-58-5.jpg
https://danderm-pdv.is.kkh.dk/atlas/pics/7/7-58-6.jpg
https://danderm-pdv.is.kkh.dk/atlas/pics/7/7-61.jpg
https://danderm-pdv.is.kkh.dk/atlas/pics/7/7-62.jpg
https://danderm-pdv.is.kkh.dk/atlas/pics/7/7-63.jpg
https://danderm-pdv.is.kkh.dk/atlas/pics/7/7-64.jpg
https://danderm-pdv.is.kkh.dk/atlas/pics/7/7-65.jpg
https://danderm-pdv.is.kkh.dk/atlas/pics/7/7-66_S.jpg
https://danderm-pdv.is.kkh.dk/atlas/pics/7/7-67_S.jpg
https://danderm-pdv.is.kkh.dk/atlas/pics/7/7-68.jpg
https://danderm-pdv.is.kkh.dk/atlas/pics/7/7-68-1.jpg
https://danderm-pdv.is.kkh.dk/atlas/pics/7/7-68-2.jpg
https://danderm-pdv.is.kkh.dk/atlas/pics/7/7-68-3.jpg

https://danderm-pdv.is.kkh.dk/atlas/pics/7/7-68-4.jpg
https://danderm-pdv.is.kkh.dk/atlas/pics/7/7-68-5.jpg
https://danderm-pdv.is.kkh.dk/atlas/pics/7/7-68-6.jpg
https://danderm-pdv.is.kkh.dk/atlas/pics/7/7-69.jpg
https://danderm-pdv.is.kkh.dk/atlas/pics/7/7-69-1.jpg
https://danderm-pdv.is.kkh.dk/atlas/pics/7/7-69-2.jpg
https://danderm-pdv.is.kkh.dk/atlas/pics/7/7-69-3.jpg
https://danderm-pdv.is.kkh.dk/atlas/pics/7/7-71_S.jpg
https://danderm-pdv.is.kkh.dk/atlas/pics/7/7-71-1.jpg
https://danderm-pdv.is.kkh.dk/atlas/pics/7/7-71-2.jpg
https://danderm-pdv.is.kkh.dk/atlas/pics/7/7-71-3.jpg
https://danderm-pdv.is.kkh.dk/atlas/pics/7/7-71-4.jpg
https://danderm-pdv.is.kkh.dk/atlas/pics/7/7-72.jpg
https://danderm-pdv.is.kkh.dk/atlas/pics/7/7-73.jpg
https://danderm-pdv.is.kkh.dk/atlas/pics/7/7-74.jpg
https://danderm-pdv.is.kkh.dk/atlas/pics/7/7-74-1.jpg
https://danderm-pdv.is.kkh.dk/atlas/pics/7/7-74-2.jpg
https://danderm-pdv.is.kkh.dk/atlas/pics/7/7-74-3.jpg
https://danderm-pdv.is.kkh.dk/atlas/pics/7/7-74-4.jpg
https://danderm-pdv.is.kkh.dk/atlas/pics/7/7-74-5.jpg
https://danderm-pdv.is.kkh.dk/atlas/pics/7/7-75.jpg
https://danderm-pdv.is.kkh.dk/atlas/pics/7/7-75-1.jpg
https://danderm-pdv.is.kkh.dk/atlas/pics/7/7-75-2.jpg
https://danderm-pdv.is.kkh.dk/atlas/pics/7/7-75-3.jpg
https://danderm-pdv.is.kkh.dk/atlas/pics/7/7-75-4.jpg
https://danderm-pdv.is.kkh.dk/atlas/pics/7/7-75-5.jpg
https://danderm-pdv.is.kkh.dk/atlas/pics/7/7-75-6.jpg
https://danderm-pdv.is.kkh.dk/atlas/pics/7/7-75-7.jpg
https://danderm-pdv.is.kkh.dk/atlas/pics/7/7-75-8.jpg
https://danderm-pdv.is.kkh.dk/atlas/pics/7/7-75-9.jpg
https://danderm-pdv.is.kkh.dk/atlas/pics/7/7-76.jpg
https://danderm-pdv.is.kkh.dk/atlas/pics/7/7-77.jpg
https://danderm-pdv.is.kkh.dk/atlas/pics/7/7-78.jpg
https://danderm-pdv.is.kkh.dk/atlas/pics/7/7-79.jpg
https://danderm-pdv.is.kkh.dk/atlas/pics/7/7-80.jpg
https://danderm-pdv.is.kkh.dk/atlas/pics/7/7-81.jpg
https://danderm-pdv.is.kkh.dk/atlas/pics/7/7-81-1.jpg
https://danderm-pdv.is.kkh.dk/atlas/pics/7/7-82.jpg
https://danderm-pdv.is.kkh.dk/atlas/pics/7/7-82-1.jpg
https://danderm-pdv.is.kkh.dk/atlas/pics/7/7-82-2.jpg
https://danderm-pdv.is.kkh.dk/atlas/pics/7/7-82-3.jpg
https://danderm-pdv.is.kkh.dk/atlas/pics/7/7-82-4.jpg
https://danderm-pdv.is.kkh.dk/atlas/pics/7/7-82-5.jpg
https://danderm-pdv.is.kkh.dk/atlas/pics/7/7-82-6.jpg
https://danderm-pdv.is.kkh.dk/atlas/pics/7/7-82-7.jpg
https://danderm-pdv.is.kkh.dk/atlas/pics/7/7-82-8.jpg
https://danderm-pdv.is.kkh.dk/atlas/pics/7/7-83.jpg
https://danderm-pdv.is.kkh.dk/atlas/pics/7/7-84.jpg

https://danderm-pdv.is.kkh.dk/atlas/pics/7/7-84-1.jpg
https://danderm-pdv.is.kkh.dk/atlas/pics/7/7-84-2.jpg
https://danderm-pdv.is.kkh.dk/atlas/pics/7/7-84-3.jpg
https://danderm-pdv.is.kkh.dk/atlas/pics/7/7-84-4.jpg
https://danderm-pdv.is.kkh.dk/atlas/pics/7/7-84-5.jpg
https://danderm-pdv.is.kkh.dk/atlas/pics/7/7-85.jpg
https://danderm-pdv.is.kkh.dk/atlas/pics/7/7-86.jpg
https://danderm-pdv.is.kkh.dk/atlas/pics/7/7-87.jpg
https://danderm-pdv.is.kkh.dk/atlas/pics/7/7-88.jpg
https://danderm-pdv.is.kkh.dk/atlas/pics/7/7-89.jpg
https://danderm-pdv.is.kkh.dk/atlas/pics/7/7-90_S.jpg
https://danderm-pdv.is.kkh.dk/atlas/pics/7/7-90-1.jpg
https://danderm-pdv.is.kkh.dk/atlas/pics/7/7-90-2.jpg
https://danderm-pdv.is.kkh.dk/atlas/pics/7/7-90-3.jpg
https://danderm-pdv.is.kkh.dk/atlas/pics/7/7-90-4.jpg
https://danderm-pdv.is.kkh.dk/atlas/pics/7/7-90-5.jpg
https://danderm-pdv.is.kkh.dk/atlas/pics/7/7-90-6.jpg
https://danderm-pdv.is.kkh.dk/atlas/pics/7/7-90-7.jpg
https://danderm-pdv.is.kkh.dk/atlas/pics/7/7-91.jpg
https://danderm-pdv.is.kkh.dk/atlas/pics/7/7-91-1.jpg
https://danderm-pdv.is.kkh.dk/atlas/pics/7/7-91-2.jpg
https://danderm-pdv.is.kkh.dk/atlas/pics/7/7-91-3.jpg
https://danderm-pdv.is.kkh.dk/atlas/pics/7/7-91-4.jpg
https://danderm-pdv.is.kkh.dk/atlas/pics/7/7-91-6.jpg
https://danderm-pdv.is.kkh.dk/atlas/pics/7/7-91-7.jpg
https://danderm-pdv.is.kkh.dk/atlas/pics/7/7-91-8.jpg
https://danderm-pdv.is.kkh.dk/atlas/pics/7/7-91-9.jpg
https://danderm-pdv.is.kkh.dk/atlas/pics/7/7-91-10.jpg
https://danderm-pdv.is.kkh.dk/atlas/pics/7/7-91-11.jpg
https://danderm-pdv.is.kkh.dk/atlas/pics/7/7-92.jpg
https://danderm-pdv.is.kkh.dk/atlas/pics/7/7-92-1.jpg
https://danderm-pdv.is.kkh.dk/atlas/pics/7/7-93.jpg
https://danderm-pdv.is.kkh.dk/atlas/pics/7/7-94.jpg
https://danderm-pdv.is.kkh.dk/atlas/pics/7/7-95.jpg
https://danderm-pdv.is.kkh.dk/atlas/pics/7/7-95-1.jpg
https://danderm-pdv.is.kkh.dk/atlas/pics/7/7-95-2.jpg
https://danderm-pdv.is.kkh.dk/atlas/pics/7/7-96.jpg
https://danderm-pdv.is.kkh.dk/atlas/pics/7/7-97-1.jpg
https://danderm-pdv.is.kkh.dk/atlas/pics/7/7-98.jpg
https://danderm-pdv.is.kkh.dk/atlas/pics/7/7-98-1.jpg
https://danderm-pdv.is.kkh.dk/atlas/pics/7/7-98-2.jpg
https://danderm-pdv.is.kkh.dk/atlas/pics/7/7-98-3.jpg
https://danderm-pdv.is.kkh.dk/atlas/pics/7/7-99.jpg
https://danderm-pdv.is.kkh.dk/atlas/pics/7/7-100.jpg
https://danderm-pdv.is.kkh.dk/atlas/pics/7/7-101_S.jpg
https://danderm-pdv.is.kkh.dk/atlas/pics/7/7-100-1.jpg
https://danderm-pdv.is.kkh.dk/atlas/pics/7/7-100-2.jpg
https://danderm-pdv.is.kkh.dk/atlas/pics/7/7-100-3.jpg

https://danderm-pdv.is.kkh.dk/atlas/pics/7/7-100-4.jpg
https://danderm-pdv.is.kkh.dk/atlas/pics/7/7-101-1.jpg
https://danderm-pdv.is.kkh.dk/atlas/pics/7/7-101-2.jpg
https://danderm-pdv.is.kkh.dk/atlas/pics/7/7-101-3.jpg
https://danderm-pdv.is.kkh.dk/atlas/pics/7/7-101-4.jpg
https://danderm-pdv.is.kkh.dk/atlas/pics/7/7-101-5.jpg
https://danderm-pdv.is.kkh.dk/atlas/pics/7/7-102.jpg
https://danderm-pdv.is.kkh.dk/atlas/pics/7/7-103.jpg
https://danderm-pdv.is.kkh.dk/atlas/pics/7/7-103-1.jpg
https://danderm-pdv.is.kkh.dk/atlas/pics/7/7-103-2.jpg
https://danderm-pdv.is.kkh.dk/atlas/pics/7/7-104.jpg
https://danderm-pdv.is.kkh.dk/atlas/pics/7/7-105.jpg
https://danderm-pdv.is.kkh.dk/atlas/pics/7/7-106.jpg
https://danderm-pdv.is.kkh.dk/atlas/pics/7/7-107.jpg
https://danderm-pdv.is.kkh.dk/atlas/pics/7/7-108.jpg
https://danderm-pdv.is.kkh.dk/atlas/pics/7/7-109.jpg
https://danderm-pdv.is.kkh.dk/atlas/pics/7/7-110.jpg
https://danderm-pdv.is.kkh.dk/atlas/pics/7/7-111-1.jpg
https://danderm-pdv.is.kkh.dk/atlas/pics/7/7-111-2.jpg
https://danderm-pdv.is.kkh.dk/atlas/pics/7/7-112.jpg
https://danderm-pdv.is.kkh.dk/atlas/pics/7/7-113.jpg
https://danderm-pdv.is.kkh.dk/atlas/pics/7/7-113-1.jpg
https://danderm-pdv.is.kkh.dk/atlas/pics/7/7-114.jpg
https://danderm-pdv.is.kkh.dk/atlas/pics/7/7-115.jpg
https://danderm-pdv.is.kkh.dk/atlas/pics/7/7-115-1.jpg
https://danderm-pdv.is.kkh.dk/atlas/pics/7/7-115-2.jpg
https://danderm-pdv.is.kkh.dk/atlas/pics/7/7-116.jpg
https://danderm-pdv.is.kkh.dk/atlas/pics/7/7-117.jpg
https://danderm-pdv.is.kkh.dk/atlas/pics/7/7-118.jpg
https://danderm-pdv.is.kkh.dk/atlas/pics/7/7-118-8.jpg
https://danderm-pdv.is.kkh.dk/atlas/pics/7/7-119.jpg
https://danderm-pdv.is.kkh.dk/atlas/pics/7/7-119-2.jpg
https://danderm-pdv.is.kkh.dk/atlas/pics/7/7-119-3.jpg
https://danderm-pdv.is.kkh.dk/atlas/pics/7/7-119-4.jpg
https://danderm-pdv.is.kkh.dk/atlas/pics/7/7-119-5.jpg
https://danderm-pdv.is.kkh.dk/atlas/pics/7/7-120.jpg
https://danderm-pdv.is.kkh.dk/atlas/pics/7/7-120-1.jpg
https://danderm-pdv.is.kkh.dk/atlas/pics/7/7-121.jpg
https://danderm-pdv.is.kkh.dk/atlas/pics/7/7-122.jpg
https://danderm-pdv.is.kkh.dk/atlas/pics/7/7-122-1.jpg
https://danderm-pdv.is.kkh.dk/atlas/pics/7/7-122-2.jpg
https://danderm-pdv.is.kkh.dk/atlas/pics/7/7-123.jpg
https://danderm-pdv.is.kkh.dk/atlas/pics/7/7-124.jpg
https://danderm-pdv.is.kkh.dk/atlas/pics/7/7-124-1.jpg
https://danderm-pdv.is.kkh.dk/atlas/pics/7/7-125.jpg
https://danderm-pdv.is.kkh.dk/atlas/pics/7/7-125-1.jpg
https://danderm-pdv.is.kkh.dk/atlas/pics/7/7-125-2.jpg
https://danderm-pdv.is.kkh.dk/atlas/pics/7/7-125-3.jpg

https://danderm-pdv.is.kkh.dk/atlas/pics/7/7-125-4.jpg
https://danderm-pdv.is.kkh.dk/atlas/pics/7/7-126.jpg
https://danderm-pdv.is.kkh.dk/atlas/pics/7/7-127.jpg
https://danderm-pdv.is.kkh.dk/atlas/pics/7/7-127-1.jpg
https://danderm-pdv.is.kkh.dk/atlas/pics/7/7-127-1-1.jpg
https://danderm-pdv.is.kkh.dk/atlas/pics/7/7-128_S.jpg
https://danderm-pdv.is.kkh.dk/atlas/pics/7/7-129.jpg
https://danderm-pdv.is.kkh.dk/atlas/pics/7/7-130.jpg
https://danderm-pdv.is.kkh.dk/atlas/pics/7/7-130-1.jpg
https://danderm-pdv.is.kkh.dk/atlas/pics/7/7-130-2.jpg
https://danderm-pdv.is.kkh.dk/atlas/pics/7/7-131.jpg
https://danderm-pdv.is.kkh.dk/atlas/pics/7/7-132.jpg
https://danderm-pdv.is.kkh.dk/atlas/pics/7/7-133.jpg
https://danderm-pdv.is.kkh.dk/atlas/pics/7/7-134.jpg
https://danderm-pdv.is.kkh.dk/atlas/pics/7/7-134-1.jpg
https://danderm-pdv.is.kkh.dk/atlas/pics/7/7-134-2.jpg
https://danderm-pdv.is.kkh.dk/atlas/pics/7/7-134-3.jpg
https://danderm-pdv.is.kkh.dk/atlas/pics/7/7-134-4.jpg
https://danderm-pdv.is.kkh.dk/atlas/pics/7/7-134-5.jpg
https://danderm-pdv.is.kkh.dk/atlas/pics/7/7-134-6.jpg
https://danderm-pdv.is.kkh.dk/atlas/pics/7/7-135.jpg
https://danderm-pdv.is.kkh.dk/atlas/pics/7/7-136.jpg
https://danderm-pdv.is.kkh.dk/atlas/pics/7/7-136-1.jpg
https://danderm-pdv.is.kkh.dk/atlas/pics/7/7-136-2.jpg
https://danderm-pdv.is.kkh.dk/atlas/pics/7/7-136-3.jpg
https://danderm-pdv.is.kkh.dk/atlas/pics/7/7-136-4.jpg
https://danderm-pdv.is.kkh.dk/atlas/pics/7/7-137.jpg
https://danderm-pdv.is.kkh.dk/atlas/pics/7/7-137-1.jpg
https://danderm-pdv.is.kkh.dk/atlas/pics/7/7-137-2.jpg
https://danderm-pdv.is.kkh.dk/atlas/pics/7/7-137-3.jpg
https://danderm-pdv.is.kkh.dk/atlas/pics/7/7-138.jpg
https://danderm-pdv.is.kkh.dk/atlas/pics/7/7-139_S.jpg
https://danderm-pdv.is.kkh.dk/atlas/pics/7/7-140_S.jpg
https://danderm-pdv.is.kkh.dk/atlas/pics/7/7-141.jpg
https://danderm-pdv.is.kkh.dk/atlas/pics/7/7-142.jpg
https://danderm-pdv.is.kkh.dk/atlas/pics/7/7-143.jpg
https://danderm-pdv.is.kkh.dk/atlas/pics/7/7-143-1.jpg
https://danderm-pdv.is.kkh.dk/atlas/pics/7/7-143-2.jpg
https://danderm-pdv.is.kkh.dk/atlas/pics/7/7-143-3.jpg
https://danderm-pdv.is.kkh.dk/atlas/pics/7/7-143-4.jpg
https://danderm-pdv.is.kkh.dk/atlas/pics/7/7-143-5.jpg
https://danderm-pdv.is.kkh.dk/atlas/pics/7/7-144.jpg
https://danderm-pdv.is.kkh.dk/atlas/pics/7/7-145_S.jpg
https://danderm-pdv.is.kkh.dk/atlas/pics/7/7-146.jpg
https://danderm-pdv.is.kkh.dk/atlas/pics/7/7-146-1.jpg
https://danderm-pdv.is.kkh.dk/atlas/pics/7/7-146-2.jpg
https://danderm-pdv.is.kkh.dk/atlas/pics/7/7-146-3.jpg
https://danderm-pdv.is.kkh.dk/atlas/pics/7/7-146-4.jpg

https://danderm-pdv.is.kkh.dk/atlas/pics/7/7-147.jpg
https://danderm-pdv.is.kkh.dk/atlas/pics/7/7-147-2.jpg
https://danderm-pdv.is.kkh.dk/atlas/pics/7/7-147-3.jpg
https://danderm-pdv.is.kkh.dk/atlas/pics/7/7-147-4.jpg
https://danderm-pdv.is.kkh.dk/atlas/pics/7/7-147-5.jpg
https://danderm-pdv.is.kkh.dk/atlas/pics/7/7-148.jpg
https://danderm-pdv.is.kkh.dk/atlas/pics/7/7-148-1.jpg
https://danderm-pdv.is.kkh.dk/atlas/pics/7/7-148-2.jpg
https://danderm-pdv.is.kkh.dk/atlas/pics/7/7-150.jpg
https://danderm-pdv.is.kkh.dk/atlas/pics/7/7-150-1.jpg
https://danderm-pdv.is.kkh.dk/atlas/pics/7/7-150-2.jpg
https://danderm-pdv.is.kkh.dk/atlas/pics/7/7-151.jpg
https://danderm-pdv.is.kkh.dk/atlas/pics/7/7-151-1.jpg
https://danderm-pdv.is.kkh.dk/atlas/pics/7/7-151-2.jpg
https://danderm-pdv.is.kkh.dk/atlas/pics/7/7-152.jpg
https://danderm-pdv.is.kkh.dk/atlas/pics/7/7-152-1.jpg
https://danderm-pdv.is.kkh.dk/atlas/pics/7/7-152-2.jpg
https://danderm-pdv.is.kkh.dk/atlas/pics/7/7-152-3.jpg
https://danderm-pdv.is.kkh.dk/atlas/pics/7/7-153.jpg
https://danderm-pdv.is.kkh.dk/atlas/pics/7/7-154.jpg
https://danderm-pdv.is.kkh.dk/atlas/pics/7/7-154-1.jpg
https://danderm-pdv.is.kkh.dk/atlas/pics/7/7-154-2.jpg
https://danderm-pdv.is.kkh.dk/atlas/pics/7/7-154-3.jpg
https://danderm-pdv.is.kkh.dk/atlas/pics/7/7-154-4.jpg
https://danderm-pdv.is.kkh.dk/atlas/pics/7/7-154-5.jpg
https://danderm-pdv.is.kkh.dk/atlas/pics/7/7-155.jpg
https://danderm-pdv.is.kkh.dk/atlas/pics/7/7-156.jpg
https://danderm-pdv.is.kkh.dk/atlas/pics/7/7-156-a.jpg
https://danderm-pdv.is.kkh.dk/atlas/pics/7/7-156-b.jpg
https://danderm-pdv.is.kkh.dk/atlas/pics/7/7-156-1.jpg
https://danderm-pdv.is.kkh.dk/atlas/pics/7/7-156-2.jpg
https://danderm-pdv.is.kkh.dk/atlas/pics/7/7-157.jpg
https://danderm-pdv.is.kkh.dk/atlas/pics/7/7-158.jpg
https://danderm-pdv.is.kkh.dk/atlas/pics/7/7-159.jpg
https://danderm-pdv.is.kkh.dk/atlas/pics/7/7-159-1.jpg
https://danderm-pdv.is.kkh.dk/atlas/pics/7/7-159-2.jpg
https://danderm-pdv.is.kkh.dk/atlas/pics/7/7-159-3.jpg
https://danderm-pdv.is.kkh.dk/atlas/pics/7/7-159-4.jpg
https://danderm-pdv.is.kkh.dk/atlas/pics/7/7-159-5.jpg
https://danderm-pdv.is.kkh.dk/atlas/pics/7/7-159-6.jpg
https://danderm-pdv.is.kkh.dk/atlas/pics/7/7-159-7.jpg
https://danderm-pdv.is.kkh.dk/atlas/pics/7/7-159-8.jpg
https://danderm-pdv.is.kkh.dk/atlas/pics/7/7-159-9.jpg
https://danderm-pdv.is.kkh.dk/atlas/pics/7/7-159-10.jpg
https://danderm-pdv.is.kkh.dk/atlas/pics/7/7-159-11.jpg
https://danderm-pdv.is.kkh.dk/atlas/pics/7/7-160.jpg
https://danderm-pdv.is.kkh.dk/atlas/pics/7/7-161.jpg
https://danderm-pdv.is.kkh.dk/atlas/pics/7/7-162.jpg

https://danderm-pdv.is.kkh.dk/atlas/pics/7/7-163.jpg
https://danderm-pdv.is.kkh.dk/atlas/pics/7/7-164.jpg
https://danderm-pdv.is.kkh.dk/atlas/pics/7/7-165.jpg
https://danderm-pdv.is.kkh.dk/atlas/pics/7/7-166.jpg
https://danderm-pdv.is.kkh.dk/atlas/pics/7/7-167.jpg
https://danderm-pdv.is.kkh.dk/atlas/pics/7/7-167-1.jpg
https://danderm-pdv.is.kkh.dk/atlas/pics/7/7-167-2.jpg
https://danderm-pdv.is.kkh.dk/atlas/pics/7/7-167-3.jpg
https://danderm-pdv.is.kkh.dk/atlas/pics/7/7-167-4.jpg
https://danderm-pdv.is.kkh.dk/atlas/pics/7/7-167-5.jpg
https://danderm-pdv.is.kkh.dk/atlas/pics/7/7-167-6.jpg
https://danderm-pdv.is.kkh.dk/atlas/pics/7/7-167-7.jpg
https://danderm-pdv.is.kkh.dk/atlas/pics/7/7-167-9.jpg
https://danderm-pdv.is.kkh.dk/atlas/pics/7/7-167-10.jpg
https://danderm-pdv.is.kkh.dk/atlas/pics/7/7-168.jpg
https://danderm-pdv.is.kkh.dk/atlas/pics/7/7-168-1.jpg
https://danderm-pdv.is.kkh.dk/atlas/pics/7/7-168-2.jpg
https://danderm-pdv.is.kkh.dk/atlas/pics/7/7-168-3.jpg
https://danderm-pdv.is.kkh.dk/atlas/pics/7/7-169.jpg
https://danderm-pdv.is.kkh.dk/atlas/pics/7/7-170.jpg
https://danderm-pdv.is.kkh.dk/atlas/pics/7/7-171.jpg
https://danderm-pdv.is.kkh.dk/atlas/pics/7/7-172.jpg
https://danderm-pdv.is.kkh.dk/atlas/pics/7/7-173.jpg
https://danderm-pdv.is.kkh.dk/atlas/pics/7/7-174.jpg
https://danderm-pdv.is.kkh.dk/atlas/pics/7/7-174-1.jpg
https://danderm-pdv.is.kkh.dk/atlas/pics/7/7-174-2.jpg
https://danderm-pdv.is.kkh.dk/atlas/pics/7/7-174-3.jpg
https://danderm-pdv.is.kkh.dk/atlas/pics/7/7-174-4.jpg
https://danderm-pdv.is.kkh.dk/atlas/pics/7/7-175.jpg
https://danderm-pdv.is.kkh.dk/atlas/pics/7/7-176.jpg
https://danderm-pdv.is.kkh.dk/atlas/pics/7/7-177.jpg
https://danderm-pdv.is.kkh.dk/atlas/pics/7/7-178.jpg
https://danderm-pdv.is.kkh.dk/atlas/pics/7/7-178-1.jpg
https://danderm-pdv.is.kkh.dk/atlas/pics/7/7-178-5.jpg
https://danderm-pdv.is.kkh.dk/atlas/pics/7/7-178-6.jpg
https://danderm-pdv.is.kkh.dk/atlas/pics/7/7-179.jpg
https://danderm-pdv.is.kkh.dk/atlas/pics/7/7-180-1.jpg
https://danderm-pdv.is.kkh.dk/atlas/pics/7/7-180-2.jpg
https://danderm-pdv.is.kkh.dk/atlas/pics/7/7-181.jpg
https://danderm-pdv.is.kkh.dk/atlas/pics/7/7-182.jpg
https://danderm-pdv.is.kkh.dk/atlas/pics/7/7-183.jpg
https://danderm-pdv.is.kkh.dk/atlas/pics/7/7-183-3.jpg
https://danderm-pdv.is.kkh.dk/atlas/pics/7/7-183-4.jpg
https://danderm-pdv.is.kkh.dk/atlas/pics/7/7-183-5.jpg
https://danderm-pdv.is.kkh.dk/atlas/pics/7/7-184.jpg
https://danderm-pdv.is.kkh.dk/atlas/pics/7/7-185.jpg
https://danderm-pdv.is.kkh.dk/atlas/pics/7/7-186.jpg
https://danderm-pdv.is.kkh.dk/atlas/pics/7/7-186-1.jpg

https://danderm-pdv.is.kkh.dk/atlas/pics/7/7-186-2.jpg
https://danderm-pdv.is.kkh.dk/atlas/pics/7/7-186-3.jpg
https://danderm-pdv.is.kkh.dk/atlas/pics/7/7-186-4.jpg
https://danderm-pdv.is.kkh.dk/atlas/pics/7/7-186-8.jpg
https://danderm-pdv.is.kkh.dk/atlas/pics/7/7-186-9.jpg
https://danderm-pdv.is.kkh.dk/atlas/pics/7/7-186-10.jpg
https://danderm-pdv.is.kkh.dk/atlas/pics/7/7-187.jpg
https://danderm-pdv.is.kkh.dk/atlas/pics/7/7-188_S.jpg
https://danderm-pdv.is.kkh.dk/atlas/pics/7/7-189.jpg
https://danderm-pdv.is.kkh.dk/atlas/pics/7/7-189-1.jpg
https://danderm-pdv.is.kkh.dk/atlas/pics/7/7-189-2.jpg
https://danderm-pdv.is.kkh.dk/atlas/pics/7/7-189-3.jpg
https://danderm-pdv.is.kkh.dk/atlas/pics/7/7-189-4.jpg
https://danderm-pdv.is.kkh.dk/atlas/pics/7/7-189-5.jpg
https://danderm-pdv.is.kkh.dk/atlas/pics/7/7-189-6.jpg
https://danderm-pdv.is.kkh.dk/atlas/pics/7/7-189-7.jpg
https://danderm-pdv.is.kkh.dk/atlas/pics/7/7-189-8.jpg
https://danderm-pdv.is.kkh.dk/atlas/pics/7/7-189-9.jpg
https://danderm-pdv.is.kkh.dk/atlas/pics/7/7-189-10.jpg
https://danderm-pdv.is.kkh.dk/atlas/pics/7/7-189-11.jpg
https://danderm-pdv.is.kkh.dk/atlas/pics/7/7-190.jpg
https://danderm-pdv.is.kkh.dk/atlas/pics/7/7-191.jpg
https://danderm-pdv.is.kkh.dk/atlas/pics/7/7-192.jpg
https://danderm-pdv.is.kkh.dk/atlas/pics/7/7-192-1.jpg
https://danderm-pdv.is.kkh.dk/atlas/pics/7/7-192-2.jpg
https://danderm-pdv.is.kkh.dk/atlas/pics/7/7-193.jpg
https://danderm-pdv.is.kkh.dk/atlas/pics/7/7-193-1.jpg
https://danderm-pdv.is.kkh.dk/atlas/pics/7/7-193-2.jpg
https://danderm-pdv.is.kkh.dk/atlas/pics/7/7-193-3.jpg
https://danderm-pdv.is.kkh.dk/atlas/pics/7/7-193-4.jpg
https://danderm-pdv.is.kkh.dk/atlas/pics/7/7-193-5.jpg
https://danderm-pdv.is.kkh.dk/atlas/pics/7/7-193-6.jpg
https://danderm-pdv.is.kkh.dk/atlas/pics/7/7-193-7.jpg
https://danderm-pdv.is.kkh.dk/atlas/pics/7/7-193-8.jpg
https://danderm-pdv.is.kkh.dk/atlas/pics/7/7-194.jpg
https://danderm-pdv.is.kkh.dk/atlas/pics/7/7-195.jpg
https://danderm-pdv.is.kkh.dk/atlas/pics/7/7-195-1.jpg
https://danderm-pdv.is.kkh.dk/atlas/pics/7/7-195-2.jpg
https://danderm-pdv.is.kkh.dk/atlas/pics/7/7-195-3.jpg
https://danderm-pdv.is.kkh.dk/atlas/pics/7/7-195-4.jpg
https://danderm-pdv.is.kkh.dk/atlas/pics/7/7-195-5.jpg
https://danderm-pdv.is.kkh.dk/atlas/pics/7/7-195-6.jpg
https://danderm-pdv.is.kkh.dk/atlas/pics/7/7-195-7.jpg
https://danderm-pdv.is.kkh.dk/atlas/pics/7/7-196.jpg
https://danderm-pdv.is.kkh.dk/atlas/pics/7/7-197.jpg
https://danderm-pdv.is.kkh.dk/atlas/pics/7/7-197-1.jpg
https://danderm-pdv.is.kkh.dk/atlas/pics/7/7-197-2.jpg
https://danderm-pdv.is.kkh.dk/atlas/pics/7/7-197-3.jpg

https://danderm-pdv.is.kkh.dk/atlas/pics/7/7-198.jpg
https://danderm-pdv.is.kkh.dk/atlas/pics/7/7-198-1.jpg
https://danderm-pdv.is.kkh.dk/atlas/pics/7/7-199.jpg
https://danderm-pdv.is.kkh.dk/atlas/pics/7/7-199-1.jpg
https://danderm-pdv.is.kkh.dk/atlas/pics/7/7-199-2.jpg
https://danderm-pdv.is.kkh.dk/atlas/pics/7/7-200.jpg
https://danderm-pdv.is.kkh.dk/atlas/pics/7/7-200-1.jpg
https://danderm-pdv.is.kkh.dk/atlas/pics/7/7-200-2.jpg
https://danderm-pdv.is.kkh.dk/atlas/pics/7/7-200-3.jpg
https://danderm-pdv.is.kkh.dk/atlas/pics/7/7-201.jpg
https://danderm-pdv.is.kkh.dk/atlas/pics/7/7-201-1.jpg
https://danderm-pdv.is.kkh.dk/atlas/pics/7/7-201-2.jpg
https://danderm-pdv.is.kkh.dk/atlas/pics/7/7-201-3.jpg
https://danderm-pdv.is.kkh.dk/atlas/pics/7/7-201-4.jpg
https://danderm-pdv.is.kkh.dk/atlas/pics/7/7-202.jpg
https://danderm-pdv.is.kkh.dk/atlas/pics/7/7-203.jpg
https://danderm-pdv.is.kkh.dk/atlas/pics/8/8-01.jpg
https://danderm-pdv.is.kkh.dk/atlas/pics/8/8-02_S.jpg
https://danderm-pdv.is.kkh.dk/atlas/pics/8/8-2-1.jpg
https://danderm-pdv.is.kkh.dk/atlas/pics/8/8-03.jpg
https://danderm-pdv.is.kkh.dk/atlas/pics/8/8-3-1.jpg
https://danderm-pdv.is.kkh.dk/atlas/pics/8/8-3-2.jpg
https://danderm-pdv.is.kkh.dk/atlas/pics/8/8-3-3.jpg
https://danderm-pdv.is.kkh.dk/atlas/pics/8/8-3-4.jpg
https://danderm-pdv.is.kkh.dk/atlas/pics/8/8-3-5.jpg
https://danderm-pdv.is.kkh.dk/atlas/pics/8/8-3-7.jpg
https://danderm-pdv.is.kkh.dk/atlas/pics/8/8-3-8.jpg
https://danderm-pdv.is.kkh.dk/atlas/pics/8/8-3-9.jpg
https://danderm-pdv.is.kkh.dk/atlas/pics/8/8-3-10.jpg
https://danderm-pdv.is.kkh.dk/atlas/pics/8/8-3-11.jpg
https://danderm-pdv.is.kkh.dk/atlas/pics/8/8-3-12.jpg
https://danderm-pdv.is.kkh.dk/atlas/pics/8/8-3-13.jpg
https://danderm-pdv.is.kkh.dk/atlas/pics/8/8-3-14.jpg
https://danderm-pdv.is.kkh.dk/atlas/pics/8/8-3-15.jpg
https://danderm-pdv.is.kkh.dk/atlas/pics/8/8-3-16.jpg
https://danderm-pdv.is.kkh.dk/atlas/pics/8/8-3-17.jpg
https://danderm-pdv.is.kkh.dk/atlas/pics/8/8-3-18.jpg
https://danderm-pdv.is.kkh.dk/atlas/pics/8/8-3-19.jpg
https://danderm-pdv.is.kkh.dk/atlas/pics/8/8-3-20.jpg
https://danderm-pdv.is.kkh.dk/atlas/pics/8/8-3-21.jpg
https://danderm-pdv.is.kkh.dk/atlas/pics/8/8-04.jpg
https://danderm-pdv.is.kkh.dk/atlas/pics/8/8-05.jpg
https://danderm-pdv.is.kkh.dk/atlas/pics/8/8-5-1.jpg
https://danderm-pdv.is.kkh.dk/atlas/pics/8/8-06_S.jpg
https://danderm-pdv.is.kkh.dk/atlas/pics/8/8-07.jpg
https://danderm-pdv.is.kkh.dk/atlas/pics/8/8-08.jpg
https://danderm-pdv.is.kkh.dk/atlas/pics/8/8-09.jpg
https://danderm-pdv.is.kkh.dk/atlas/pics/8/8-9-1.jpg

https://danderm-pdv.is.kkh.dk/atlas/pics/8/8-9-2.jpg
https://danderm-pdv.is.kkh.dk/atlas/pics/8/8-10.jpg
https://danderm-pdv.is.kkh.dk/atlas/pics/8/8-11.jpg
https://danderm-pdv.is.kkh.dk/atlas/pics/8/8-12.jpg
https://danderm-pdv.is.kkh.dk/atlas/pics/8/8-13.jpg
https://danderm-pdv.is.kkh.dk/atlas/pics/8/8-14_s.jpg
https://danderm-pdv.is.kkh.dk/atlas/pics/8/8-15.jpg
https://danderm-pdv.is.kkh.dk/atlas/pics/8/8-16.jpg
https://danderm-pdv.is.kkh.dk/atlas/pics/8/8-17.jpg
https://danderm-pdv.is.kkh.dk/atlas/pics/8/8-18.jpg
https://danderm-pdv.is.kkh.dk/atlas/pics/8/8-19.jpg
https://danderm-pdv.is.kkh.dk/atlas/pics/8/8-19-1.jpg
https://danderm-pdv.is.kkh.dk/atlas/pics/8/8-20.jpg
https://danderm-pdv.is.kkh.dk/atlas/pics/8/8-21.jpg
https://danderm-pdv.is.kkh.dk/atlas/pics/8/8-22.jpg
https://danderm-pdv.is.kkh.dk/atlas/pics/8/8-23.jpg
https://danderm-pdv.is.kkh.dk/atlas/pics/8/8-24.jpg
https://danderm-pdv.is.kkh.dk/atlas/pics/8/8-24-1.jpg
https://danderm-pdv.is.kkh.dk/atlas/pics/8/8-24-2.jpg
https://danderm-pdv.is.kkh.dk/atlas/pics/8/8-25.jpg
https://danderm-pdv.is.kkh.dk/atlas/pics/8/8-26.jpg
https://danderm-pdv.is.kkh.dk/atlas/pics/8/8-26-1.jpg
https://danderm-pdv.is.kkh.dk/atlas/pics/8/8-26-2.jpg
https://danderm-pdv.is.kkh.dk/atlas/pics/8/8-26-3.jpg
https://danderm-pdv.is.kkh.dk/atlas/pics/8/8-26-4.jpg
https://danderm-pdv.is.kkh.dk/atlas/pics/8/8-27.jpg
https://danderm-pdv.is.kkh.dk/atlas/pics/8/8-28.jpg
https://danderm-pdv.is.kkh.dk/atlas/pics/8/8-29.jpg
https://danderm-pdv.is.kkh.dk/atlas/pics/8/8-30.jpg
https://danderm-pdv.is.kkh.dk/atlas/pics/8/8-30-1.jpg
https://danderm-pdv.is.kkh.dk/atlas/pics/8/8-31_S.jpg
https://danderm-pdv.is.kkh.dk/atlas/pics/8/8-31-2.jpg
https://danderm-pdv.is.kkh.dk/atlas/pics/8/8-31-1.jpg
https://danderm-pdv.is.kkh.dk/atlas/pics/8/8-31-3.jpg
https://danderm-pdv.is.kkh.dk/atlas/pics/8/8-32.jpg
https://danderm-pdv.is.kkh.dk/atlas/pics/8/8-32-1.jpg
https://danderm-pdv.is.kkh.dk/atlas/pics/8/8-32-2.jpg
https://danderm-pdv.is.kkh.dk/atlas/pics/8/8-33.jpg
https://danderm-pdv.is.kkh.dk/atlas/pics/8/8-33-1.jpg
https://danderm-pdv.is.kkh.dk/atlas/pics/8/8-33-2.jpg
https://danderm-pdv.is.kkh.dk/atlas/pics/8/8-33-3.jpg
https://danderm-pdv.is.kkh.dk/atlas/pics/8/8-33-4.jpg
https://danderm-pdv.is.kkh.dk/atlas/pics/8/8-34.jpg
https://danderm-pdv.is.kkh.dk/atlas/pics/8/8-35-1.jpg
https://danderm-pdv.is.kkh.dk/atlas/pics/8/8-35-2.jpg
https://danderm-pdv.is.kkh.dk/atlas/pics/8/8-35-3.jpg
https://danderm-pdv.is.kkh.dk/atlas/pics/8/8-35.jpg
https://danderm-pdv.is.kkh.dk/atlas/pics/8/8-36.jpg

https://danderm-pdv.is.kkh.dk/atlas/pics/8/8-38.jpg
https://danderm-pdv.is.kkh.dk/atlas/pics/8/8-37.jpg
https://danderm-pdv.is.kkh.dk/atlas/pics/8/8-40.jpg
https://danderm-pdv.is.kkh.dk/atlas/pics/8/8-40-1.jpg
https://danderm-pdv.is.kkh.dk/atlas/pics/8/8-40-2.jpg
https://danderm-pdv.is.kkh.dk/atlas/pics/8/8-40-3.jpg
https://danderm-pdv.is.kkh.dk/atlas/pics/8/8-40-4.jpg
https://danderm-pdv.is.kkh.dk/atlas/pics/8/8-40-5.jpg
https://danderm-pdv.is.kkh.dk/atlas/pics/8/8-40-6.jpg
https://danderm-pdv.is.kkh.dk/atlas/pics/8/8-40-7.jpg
https://danderm-pdv.is.kkh.dk/atlas/pics/8/8-40-8.jpg
https://danderm-pdv.is.kkh.dk/atlas/pics/8/8-40-9.jpg
https://danderm-pdv.is.kkh.dk/atlas/pics/8/8-41.jpg
https://danderm-pdv.is.kkh.dk/atlas/pics/8/8-42.jpg
https://danderm-pdv.is.kkh.dk/atlas/pics/8/8-42-1.jpg
https://danderm-pdv.is.kkh.dk/atlas/pics/8/8-42-2.jpg
https://danderm-pdv.is.kkh.dk/atlas/pics/8/8-42-3.jpg
https://danderm-pdv.is.kkh.dk/atlas/pics/8/8-42-4.jpg
https://danderm-pdv.is.kkh.dk/atlas/pics/8/8-43.jpg
https://danderm-pdv.is.kkh.dk/atlas/pics/8/8-43-2.jpg
https://danderm-pdv.is.kkh.dk/atlas/pics/8/8-43-3.jpg
https://danderm-pdv.is.kkh.dk/atlas/pics/8/8-43-1.jpg
https://danderm-pdv.is.kkh.dk/atlas/pics/8/8-44.jpg
https://danderm-pdv.is.kkh.dk/atlas/pics/8/8-45.jpg
https://danderm-pdv.is.kkh.dk/atlas/pics/8/8-46.jpg
https://danderm-pdv.is.kkh.dk/atlas/pics/8/8-46-1.jpg
https://danderm-pdv.is.kkh.dk/atlas/pics/8/8-46-2.jpg
https://danderm-pdv.is.kkh.dk/atlas/pics/8/8-46-3.jpg
https://danderm-pdv.is.kkh.dk/atlas/pics/8/8-46-4.jpg
https://danderm-pdv.is.kkh.dk/atlas/pics/8/8-47.jpg
https://danderm-pdv.is.kkh.dk/atlas/pics/8/8-48.jpg
https://danderm-pdv.is.kkh.dk/atlas/pics/8/8-48-1.jpg
https://danderm-pdv.is.kkh.dk/atlas/pics/8/8-49.jpg
https://danderm-pdv.is.kkh.dk/atlas/pics/8/8-50.jpg
https://danderm-pdv.is.kkh.dk/atlas/pics/8/8-50-1.jpg
https://danderm-pdv.is.kkh.dk/atlas/pics/8/8-51.jpg
https://danderm-pdv.is.kkh.dk/atlas/pics/8/8-51-1.jpg
https://danderm-pdv.is.kkh.dk/atlas/pics/8/8-51-2.jpg
https://danderm-pdv.is.kkh.dk/atlas/pics/8/8-51-3.jpg
https://danderm-pdv.is.kkh.dk/atlas/pics/8/8-52.jpg
https://danderm-pdv.is.kkh.dk/atlas/pics/8/8-52-1.jpg
https://danderm-pdv.is.kkh.dk/atlas/pics/8/8-52-2.jpg
https://danderm-pdv.is.kkh.dk/atlas/pics/8/8-52-3.jpg
https://danderm-pdv.is.kkh.dk/atlas/pics/8/8-53.jpg
https://danderm-pdv.is.kkh.dk/atlas/pics/8/8-54.jpg
https://danderm-pdv.is.kkh.dk/atlas/pics/8/8-55.jpg
https://danderm-pdv.is.kkh.dk/atlas/pics/8/8-55-1.jpg
https://danderm-pdv.is.kkh.dk/atlas/pics/8/8-55-2.jpg

https://danderm-pdv.is.kkh.dk/atlas/pics/8/8-55-3.jpg
https://danderm-pdv.is.kkh.dk/atlas/pics/8/8-56.jpg
https://danderm-pdv.is.kkh.dk/atlas/pics/8/8-56-1.jpg
https://danderm-pdv.is.kkh.dk/atlas/pics/8/8-57.jpg
https://danderm-pdv.is.kkh.dk/atlas/pics/8/8-57-1.jpg
https://danderm-pdv.is.kkh.dk/atlas/pics/8/8-58.jpg
https://danderm-pdv.is.kkh.dk/atlas/pics/8/8-59.jpg
https://danderm-pdv.is.kkh.dk/atlas/pics/8/8-59-1.jpg
https://danderm-pdv.is.kkh.dk/atlas/pics/8/8-59-2.jpg
https://danderm-pdv.is.kkh.dk/atlas/pics/8/8-59-3.jpg
https://danderm-pdv.is.kkh.dk/atlas/pics/8/8-59-4.jpg
https://danderm-pdv.is.kkh.dk/atlas/pics/8/8-60.jpg
https://danderm-pdv.is.kkh.dk/atlas/pics/8/8-61.jpg
https://danderm-pdv.is.kkh.dk/atlas/pics/8/8-62.jpg
https://danderm-pdv.is.kkh.dk/atlas/pics/8/8-63.jpg
https://danderm-pdv.is.kkh.dk/atlas/pics/8/8-63-1.jpg
https://danderm-pdv.is.kkh.dk/atlas/pics/8/8-63-2.jpg
https://danderm-pdv.is.kkh.dk/atlas/pics/8/8-64.jpg
https://danderm-pdv.is.kkh.dk/atlas/pics/8/8-65.jpg
https://danderm-pdv.is.kkh.dk/atlas/pics/8/8-66.jpg
https://danderm-pdv.is.kkh.dk/atlas/pics/8/8-67.jpg
https://danderm-pdv.is.kkh.dk/atlas/pics/8/8-67-1.jpg
https://danderm-pdv.is.kkh.dk/atlas/pics/8/8-67-2.jpg
https://danderm-pdv.is.kkh.dk/atlas/pics/8/8-67-3.jpg
https://danderm-pdv.is.kkh.dk/atlas/pics/8/8-67-4.jpg
https://danderm-pdv.is.kkh.dk/atlas/pics/8/8-67-5.jpg
https://danderm-pdv.is.kkh.dk/atlas/pics/8/8-67-6.jpg
https://danderm-pdv.is.kkh.dk/atlas/pics/8/8-67-7.jpg
https://danderm-pdv.is.kkh.dk/atlas/pics/8/8-68.jpg
https://danderm-pdv.is.kkh.dk/atlas/pics/8/8-69.jpg
https://danderm-pdv.is.kkh.dk/atlas/pics/8/8-69-1.jpg
https://danderm-pdv.is.kkh.dk/atlas/pics/8/8-69-2.jpg
https://danderm-pdv.is.kkh.dk/atlas/pics/8/8-70.jpg
https://danderm-pdv.is.kkh.dk/atlas/pics/8/8-71.jpg
https://danderm-pdv.is.kkh.dk/atlas/pics/8/8-71-1.jpg
https://danderm-pdv.is.kkh.dk/atlas/pics/8/8-71-2.jpg
https://danderm-pdv.is.kkh.dk/atlas/pics/8/8-71-3.jpg
https://danderm-pdv.is.kkh.dk/atlas/pics/8/8-71-4.jpg
https://danderm-pdv.is.kkh.dk/atlas/pics/8/8-71-5.jpg
https://danderm-pdv.is.kkh.dk/atlas/pics/8/8-72_S.jpg
https://danderm-pdv.is.kkh.dk/atlas/pics/8/8-73.jpg
https://danderm-pdv.is.kkh.dk/atlas/pics/8/8-73-1.jpg
https://danderm-pdv.is.kkh.dk/atlas/pics/8/8-73-2.jpg
https://danderm-pdv.is.kkh.dk/atlas/pics/8/8-73-3.jpg
https://danderm-pdv.is.kkh.dk/atlas/pics/8/8-73-4.jpg
https://danderm-pdv.is.kkh.dk/atlas/pics/8/8-73-5.jpg
https://danderm-pdv.is.kkh.dk/atlas/pics/8/8-73-6.jpg
https://danderm-pdv.is.kkh.dk/atlas/pics/8/8-73-7.jpg

https://danderm-pdv.is.kkh.dk/atlas/pics/8/8-73-8.jpg
https://danderm-pdv.is.kkh.dk/atlas/pics/8/8-73-9.jpg
https://danderm-pdv.is.kkh.dk/atlas/pics/8/8-73-10.jpg
https://danderm-pdv.is.kkh.dk/atlas/pics/8/8-74_S.jpg
https://danderm-pdv.is.kkh.dk/atlas/pics/8/8-75_S.jpg
https://danderm-pdv.is.kkh.dk/atlas/pics/8/8-76.jpg
https://danderm-pdv.is.kkh.dk/atlas/pics/8/8-77.jpg
https://danderm-pdv.is.kkh.dk/atlas/pics/8/8-77-1.jpg
https://danderm-pdv.is.kkh.dk/atlas/pics/8/8-77-2.jpg
https://danderm-pdv.is.kkh.dk/atlas/pics/8/8-77-3.jpg
https://danderm-pdv.is.kkh.dk/atlas/pics/8/8-77-4.jpg
https://danderm-pdv.is.kkh.dk/atlas/pics/8/8-77-5.jpg
https://danderm-pdv.is.kkh.dk/atlas/pics/8/8-77-6.jpg
https://danderm-pdv.is.kkh.dk/atlas/pics/8/8-77-7.jpg
https://danderm-pdv.is.kkh.dk/atlas/pics/8/8-77-8.jpg
https://danderm-pdv.is.kkh.dk/atlas/pics/8/8-77-9.jpg
https://danderm-pdv.is.kkh.dk/atlas/pics/8/8-77-10.jpg
https://danderm-pdv.is.kkh.dk/atlas/pics/8/8-77-11.jpg
https://danderm-pdv.is.kkh.dk/atlas/pics/8/8-77-12.jpg
https://danderm-pdv.is.kkh.dk/atlas/pics/8/8-78.jpg
https://danderm-pdv.is.kkh.dk/atlas/pics/8/8-79.jpg
https://danderm-pdv.is.kkh.dk/atlas/pics/8/8-79-1.jpg
https://danderm-pdv.is.kkh.dk/atlas/pics/8/8-79-2.jpg
https://danderm-pdv.is.kkh.dk/atlas/pics/8/8-79-3.jpg
https://danderm-pdv.is.kkh.dk/atlas/pics/8/8-79-4.jpg
https://danderm-pdv.is.kkh.dk/atlas/pics/8/8-80.jpg
https://danderm-pdv.is.kkh.dk/atlas/pics/8/8-81.jpg
https://danderm-pdv.is.kkh.dk/atlas/pics/8/8-81-1.jpg
https://danderm-pdv.is.kkh.dk/atlas/pics/8/8-81-2.jpg
https://danderm-pdv.is.kkh.dk/atlas/pics/8/8-81-3.jpg
https://danderm-pdv.is.kkh.dk/atlas/pics/8/8-81-4.jpg
https://danderm-pdv.is.kkh.dk/atlas/pics/8/8-81-5.jpg
https://danderm-pdv.is.kkh.dk/atlas/pics/8/8-81-6.jpg
https://danderm-pdv.is.kkh.dk/atlas/pics/8/8-81-7.jpg
https://danderm-pdv.is.kkh.dk/atlas/pics/8/8-81-8.jpg
https://danderm-pdv.is.kkh.dk/atlas/pics/8/8-81-9.jpg
https://danderm-pdv.is.kkh.dk/atlas/pics/8/8-81-10.jpg
https://danderm-pdv.is.kkh.dk/atlas/pics/8/8-82.jpg
https://danderm-pdv.is.kkh.dk/atlas/pics/8/8-83.jpg
https://danderm-pdv.is.kkh.dk/atlas/pics/8/8-83-1.jpg
https://danderm-pdv.is.kkh.dk/atlas/pics/8/8-83-2.jpg
https://danderm-pdv.is.kkh.dk/atlas/pics/8/8-84.jpg
https://danderm-pdv.is.kkh.dk/atlas/pics/8/8-84-1.jpg
https://danderm-pdv.is.kkh.dk/atlas/pics/8/8-84-2.jpg
https://danderm-pdv.is.kkh.dk/atlas/pics/8/8-84-3.jpg
https://danderm-pdv.is.kkh.dk/atlas/pics/8/8-84-4.jpg
https://danderm-pdv.is.kkh.dk/atlas/pics/8/8-85.jpg
https://danderm-pdv.is.kkh.dk/atlas/pics/8/8-85-1.jpg

https://danderm-pdv.is.kkh.dk/atlas/pics/8/8-85-2.jpg
https://danderm-pdv.is.kkh.dk/atlas/pics/8/8-85-3.jpg
https://danderm-pdv.is.kkh.dk/atlas/pics/8/8-85-4.jpg
https://danderm-pdv.is.kkh.dk/atlas/pics/8/8-85-5.jpg
https://danderm-pdv.is.kkh.dk/atlas/pics/8/8-85-6.jpg
https://danderm-pdv.is.kkh.dk/atlas/pics/8/8-85-7.jpg
https://danderm-pdv.is.kkh.dk/atlas/pics/8/8-85-8.jpg
https://danderm-pdv.is.kkh.dk/atlas/pics/8/8-86.jpg
https://danderm-pdv.is.kkh.dk/atlas/pics/8/8-86-1.jpg
https://danderm-pdv.is.kkh.dk/atlas/pics/8/8-86-2.jpg
https://danderm-pdv.is.kkh.dk/atlas/pics/8/8-87.jpg
https://danderm-pdv.is.kkh.dk/atlas/pics/8/8-88.jpg
https://danderm-pdv.is.kkh.dk/atlas/pics/8/8-88-1.jpg
https://danderm-pdv.is.kkh.dk/atlas/pics/8/8-88-2.jpg
https://danderm-pdv.is.kkh.dk/atlas/pics/8/8-88-3.jpg
https://danderm-pdv.is.kkh.dk/atlas/pics/8/8-88-4.jpg
https://danderm-pdv.is.kkh.dk/atlas/pics/8/8-88-6.jpg
https://danderm-pdv.is.kkh.dk/atlas/pics/8/8-88-7.jpg
https://danderm-pdv.is.kkh.dk/atlas/pics/8/8-89.jpg
https://danderm-pdv.is.kkh.dk/atlas/pics/8/8-89-1.jpg
https://danderm-pdv.is.kkh.dk/atlas/pics/8/8-89-2.jpg
https://danderm-pdv.is.kkh.dk/atlas/pics/8/8-90.jpg
https://danderm-pdv.is.kkh.dk/atlas/pics/8/8-91.jpg
https://danderm-pdv.is.kkh.dk/atlas/pics/8/8-92.jpg
https://danderm-pdv.is.kkh.dk/atlas/pics/8/8-93.jpg
https://danderm-pdv.is.kkh.dk/atlas/pics/8/8-93-1.jpg
https://danderm-pdv.is.kkh.dk/atlas/pics/8/8-93-2.jpg
https://danderm-pdv.is.kkh.dk/atlas/pics/8/8-93-3.jpg
https://danderm-pdv.is.kkh.dk/atlas/pics/8/8-94.jpg
https://danderm-pdv.is.kkh.dk/atlas/pics/8/8-94-1.jpg
https://danderm-pdv.is.kkh.dk/atlas/pics/8/8-94-2.jpg
https://danderm-pdv.is.kkh.dk/atlas/pics/8/8-94-3.jpg
https://danderm-pdv.is.kkh.dk/atlas/pics/8/8-94-4.jpg
https://danderm-pdv.is.kkh.dk/atlas/pics/8/8-95.jpg
https://danderm-pdv.is.kkh.dk/atlas/pics/8/8-95-1.jpg
https://danderm-pdv.is.kkh.dk/atlas/pics/8/8-95-2.jpg
https://danderm-pdv.is.kkh.dk/atlas/pics/8/8-95-2-1.jpg
https://danderm-pdv.is.kkh.dk/atlas/pics/8/8-95-3.jpg
https://danderm-pdv.is.kkh.dk/atlas/pics/8/8-96.jpg
https://danderm-pdv.is.kkh.dk/atlas/pics/8/8-97.jpg
https://danderm-pdv.is.kkh.dk/atlas/pics/8/8-97-1.jpg
https://danderm-pdv.is.kkh.dk/atlas/pics/8/8-97-2.jpg
https://danderm-pdv.is.kkh.dk/atlas/pics/8/8-97-3.jpg
https://danderm-pdv.is.kkh.dk/atlas/pics/8/8-97-4.jpg
https://danderm-pdv.is.kkh.dk/atlas/pics/8/8-97-5.jpg
https://danderm-pdv.is.kkh.dk/atlas/pics/8/8-97-6.jpg
https://danderm-pdv.is.kkh.dk/atlas/pics/8/8-99-1.jpg
https://danderm-pdv.is.kkh.dk/atlas/pics/8/8-99-2.jpg

https://danderm-pdv.is.kkh.dk/atlas/pics/8/8-99-3.jpg
https://danderm-pdv.is.kkh.dk/atlas/pics/8/8-99-4.jpg
https://danderm-pdv.is.kkh.dk/atlas/pics/8/8-99-5.jpg
https://danderm-pdv.is.kkh.dk/atlas/pics/8/8-100.jpg
https://danderm-pdv.is.kkh.dk/atlas/pics/8/8-101.jpg
https://danderm-pdv.is.kkh.dk/atlas/pics/8/8-102.jpg
https://danderm-pdv.is.kkh.dk/atlas/pics/8/8-103.jpg
https://danderm-pdv.is.kkh.dk/atlas/pics/8/8-103-1.jpg
https://danderm-pdv.is.kkh.dk/atlas/pics/8/8-103-2.jpg
https://danderm-pdv.is.kkh.dk/atlas/pics/8/8-103-3.jpg
https://danderm-pdv.is.kkh.dk/atlas/pics/8/8-103-4.jpg
https://danderm-pdv.is.kkh.dk/atlas/pics/8/8-103-5.jpg
https://danderm-pdv.is.kkh.dk/atlas/pics/8/8-103-6.jpg
https://danderm-pdv.is.kkh.dk/atlas/pics/8/8-104.jpg
https://danderm-pdv.is.kkh.dk/atlas/pics/8/8-104-1.jpg
https://danderm-pdv.is.kkh.dk/atlas/pics/8/8-105.jpg
https://danderm-pdv.is.kkh.dk/atlas/pics/8/8-106.jpg
https://danderm-pdv.is.kkh.dk/atlas/pics/8/8-107-1.jpg
https://danderm-pdv.is.kkh.dk/atlas/pics/8/8-107-2.jpg
https://danderm-pdv.is.kkh.dk/atlas/pics/8/8-107-3.jpg
https://danderm-pdv.is.kkh.dk/atlas/pics/8/8-107-4.jpg
https://danderm-pdv.is.kkh.dk/atlas/pics/8/8-107-5.jpg
https://danderm-pdv.is.kkh.dk/atlas/pics/8/8-107-6.jpg
https://danderm-pdv.is.kkh.dk/atlas/pics/8/8-107-7.jpg
https://danderm-pdv.is.kkh.dk/atlas/pics/8/8-107-8.jpg
https://danderm-pdv.is.kkh.dk/atlas/pics/8/8-107-9.jpg
https://danderm-pdv.is.kkh.dk/atlas/pics/8/8-108_S.jpg
https://danderm-pdv.is.kkh.dk/atlas/pics/8/8-108-1.jpg
https://danderm-pdv.is.kkh.dk/atlas/pics/8/8-108-2.jpg
https://danderm-pdv.is.kkh.dk/atlas/pics/8/8-108-3.jpg
https://danderm-pdv.is.kkh.dk/atlas/pics/8/8-108-4.jpg
https://danderm-pdv.is.kkh.dk/atlas/pics/8/8-108-5.jpg
https://danderm-pdv.is.kkh.dk/atlas/pics/8/8-109.jpg
https://danderm-pdv.is.kkh.dk/atlas/pics/8/8-109-1.jpg
https://danderm-pdv.is.kkh.dk/atlas/pics/8/8-109-2.jpg
https://danderm-pdv.is.kkh.dk/atlas/pics/8/8-109-3.jpg
https://danderm-pdv.is.kkh.dk/atlas/pics/8/8-109-4.jpg
https://danderm-pdv.is.kkh.dk/atlas/pics/8/8-109-5.jpg
https://danderm-pdv.is.kkh.dk/atlas/pics/8/8-110.jpg
https://danderm-pdv.is.kkh.dk/atlas/pics/8/8-110-1.jpg
https://danderm-pdv.is.kkh.dk/atlas/pics/8/8-110-2.jpg
https://danderm-pdv.is.kkh.dk/atlas/pics/8/8-110-3.jpg
https://danderm-pdv.is.kkh.dk/atlas/pics/8/8-110-4.jpg
https://danderm-pdv.is.kkh.dk/atlas/pics/8/8-110-5.jpg
https://danderm-pdv.is.kkh.dk/atlas/pics/8/8-110-6.jpg
https://danderm-pdv.is.kkh.dk/atlas/pics/8/8-110-7.jpg
https://danderm-pdv.is.kkh.dk/atlas/pics/8/8-110-8.jpg
https://danderm-pdv.is.kkh.dk/atlas/pics/8/8-110-9.jpg

https://danderm-pdv.is.kkh.dk/atlas/pics/8/8-110-10.jpg
https://danderm-pdv.is.kkh.dk/atlas/pics/8/8-110-11.jpg
https://danderm-pdv.is.kkh.dk/atlas/pics/8/8-110-12.jpg
https://danderm-pdv.is.kkh.dk/atlas/pics/8/8-110-13.jpg
https://danderm-pdv.is.kkh.dk/atlas/pics/8/8-110-14.jpg
https://danderm-pdv.is.kkh.dk/atlas/pics/8/8-111.jpg
https://danderm-pdv.is.kkh.dk/atlas/pics/8/8-112.jpg
https://danderm-pdv.is.kkh.dk/atlas/pics/8/8-113.jpg
https://danderm-pdv.is.kkh.dk/atlas/pics/8/8-114.jpg
https://danderm-pdv.is.kkh.dk/atlas/pics/8/8-115.jpg
https://danderm-pdv.is.kkh.dk/atlas/pics/8/8-116.jpg
https://danderm-pdv.is.kkh.dk/atlas/pics/8/8-116-1.jpg
https://danderm-pdv.is.kkh.dk/atlas/pics/8/8-117.jpg
https://danderm-pdv.is.kkh.dk/atlas/pics/8/8-117-1.jpg
https://danderm-pdv.is.kkh.dk/atlas/pics/8/8-117-2.jpg
https://danderm-pdv.is.kkh.dk/atlas/pics/8/8-117-3.jpg
https://danderm-pdv.is.kkh.dk/atlas/pics/8/8-117-4.jpg
https://danderm-pdv.is.kkh.dk/atlas/pics/8/8-117-5.jpg
https://danderm-pdv.is.kkh.dk/atlas/pics/8/8-117-6.jpg
https://danderm-pdv.is.kkh.dk/atlas/pics/8/8-118.jpg
https://danderm-pdv.is.kkh.dk/atlas/pics/8/8-119.jpg
https://danderm-pdv.is.kkh.dk/atlas/pics/8/8-119-1.jpg
https://danderm-pdv.is.kkh.dk/atlas/pics/8/8-119-2.jpg
https://danderm-pdv.is.kkh.dk/atlas/pics/8/8-120.jpg
https://danderm-pdv.is.kkh.dk/atlas/pics/8/8-120-1.jpg
https://danderm-pdv.is.kkh.dk/atlas/pics/8/8-120-2.jpg
https://danderm-pdv.is.kkh.dk/atlas/pics/8/8-120-3.jpg
https://danderm-pdv.is.kkh.dk/atlas/pics/8/8-120-4.jpg
https://danderm-pdv.is.kkh.dk/atlas/pics/8/8-121.jpg
https://danderm-pdv.is.kkh.dk/atlas/pics/8/8-122.jpg
https://danderm-pdv.is.kkh.dk/atlas/pics/8/8-123.jpg
https://danderm-pdv.is.kkh.dk/atlas/pics/8/8-123-1.jpg
https://danderm-pdv.is.kkh.dk/atlas/pics/8/8-123-2.jpg
https://danderm-pdv.is.kkh.dk/atlas/pics/8/8-123-3.jpg
https://danderm-pdv.is.kkh.dk/atlas/pics/8/8-123-4.jpg
https://danderm-pdv.is.kkh.dk/atlas/pics/8/8-123-5.jpg
https://danderm-pdv.is.kkh.dk/atlas/pics/8/8-123-6.jpg
https://danderm-pdv.is.kkh.dk/atlas/pics/8/8-123-7.jpg
https://danderm-pdv.is.kkh.dk/atlas/pics/8/8-123-8.jpg
https://danderm-pdv.is.kkh.dk/atlas/pics/8/8-123-9.jpg
https://danderm-pdv.is.kkh.dk/atlas/pics/8/8-123-10.jpg
https://danderm-pdv.is.kkh.dk/atlas/pics/8/8-123-11.jpg
https://danderm-pdv.is.kkh.dk/atlas/pics/8/8-123-12.jpg
https://danderm-pdv.is.kkh.dk/atlas/pics/8/8-123-13.jpg
https://danderm-pdv.is.kkh.dk/atlas/pics/8/8-124.jpg
https://danderm-pdv.is.kkh.dk/atlas/pics/8/8-124-1.jpg
https://danderm-pdv.is.kkh.dk/atlas/pics/8/8-125.jpg
https://danderm-pdv.is.kkh.dk/atlas/pics/8/8-126.jpg

https://danderm-pdv.is.kkh.dk/atlas/pics/8/8-127.jpg
https://danderm-pdv.is.kkh.dk/atlas/pics/8/8-128_S.jpg
https://danderm-pdv.is.kkh.dk/atlas/pics/8/8-129.jpg
https://danderm-pdv.is.kkh.dk/atlas/pics/8/8-130.jpg
https://danderm-pdv.is.kkh.dk/atlas/pics/8/8-131.jpg
https://danderm-pdv.is.kkh.dk/atlas/pics/8/8-131-1.jpg
https://danderm-pdv.is.kkh.dk/atlas/pics/8/8-132.jpg
https://danderm-pdv.is.kkh.dk/atlas/pics/8/8-133.jpg
https://danderm-pdv.is.kkh.dk/atlas/pics/8/8-134.jpg
https://danderm-pdv.is.kkh.dk/atlas/pics/8/8-135.jpg
https://danderm-pdv.is.kkh.dk/atlas/pics/8/8-136.jpg
https://danderm-pdv.is.kkh.dk/atlas/pics/8/8-137.jpg
https://danderm-pdv.is.kkh.dk/atlas/pics/8/8-138.jpg
https://danderm-pdv.is.kkh.dk/atlas/pics/8/8-139.jpg
https://danderm-pdv.is.kkh.dk/atlas/pics/8/8-140.jpg
https://danderm-pdv.is.kkh.dk/atlas/pics/8/8-140-1.jpg
https://danderm-pdv.is.kkh.dk/atlas/pics/8/8-140-2.jpg
https://danderm-pdv.is.kkh.dk/atlas/pics/8/8-140-3.jpg
https://danderm-pdv.is.kkh.dk/atlas/pics/8/8-140-4.jpg
https://danderm-pdv.is.kkh.dk/atlas/pics/8/8-140-5.jpg
https://danderm-pdv.is.kkh.dk/atlas/pics/8/8-140-6.jpg
https://danderm-pdv.is.kkh.dk/atlas/pics/8/8-140-7.jpg
https://danderm-pdv.is.kkh.dk/atlas/pics/8/8-141.jpg
https://danderm-pdv.is.kkh.dk/atlas/pics/8/8-142.jpg
https://danderm-pdv.is.kkh.dk/atlas/pics/8/8-143.jpg
https://danderm-pdv.is.kkh.dk/atlas/pics/8/8-143-1.jpg
https://danderm-pdv.is.kkh.dk/atlas/pics/8/8-143-2.jpg
https://danderm-pdv.is.kkh.dk/atlas/pics/8/8-144.jpg
https://danderm-pdv.is.kkh.dk/atlas/pics/8/8-145.jpg
https://danderm-pdv.is.kkh.dk/atlas/pics/8/8-146.jpg
https://danderm-pdv.is.kkh.dk/atlas/pics/8/8-146-1.jpg
https://danderm-pdv.is.kkh.dk/atlas/pics/8/8-147.jpg
https://danderm-pdv.is.kkh.dk/atlas/pics/8/8-148.jpg
https://danderm-pdv.is.kkh.dk/atlas/pics/8/8-149.jpg
https://danderm-pdv.is.kkh.dk/atlas/pics/8/8-150.jpg
https://danderm-pdv.is.kkh.dk/atlas/pics/8/8-151.jpg
https://danderm-pdv.is.kkh.dk/atlas/pics/8/8-152.jpg
https://danderm-pdv.is.kkh.dk/atlas/pics/8/8-153.jpg
https://danderm-pdv.is.kkh.dk/atlas/pics/8/8-153-1.jpg
https://danderm-pdv.is.kkh.dk/atlas/pics/8/8-154.jpg
https://danderm-pdv.is.kkh.dk/atlas/pics/8/8-155.jpg
https://danderm-pdv.is.kkh.dk/atlas/pics/8/8-155-1.jpg
https://danderm-pdv.is.kkh.dk/atlas/pics/8/8-156.jpg
https://danderm-pdv.is.kkh.dk/atlas/pics/8/8-157.jpg
https://danderm-pdv.is.kkh.dk/atlas/pics/8/8-158.jpg
https://danderm-pdv.is.kkh.dk/atlas/pics/8/8-159.jpg
https://danderm-pdv.is.kkh.dk/atlas/pics/8/8-160.jpg
https://danderm-pdv.is.kkh.dk/atlas/pics/8/8-160-1.jpg

https://danderm-pdv.is.kkh.dk/atlas/pics/8/8-160-2.jpg
https://danderm-pdv.is.kkh.dk/atlas/pics/8/8-160-3.jpg
https://danderm-pdv.is.kkh.dk/atlas/pics/8/8-161.jpg
https://danderm-pdv.is.kkh.dk/atlas/pics/8/8-162.jpg
https://danderm-pdv.is.kkh.dk/atlas/pics/8/8-163.jpg
https://danderm-pdv.is.kkh.dk/atlas/pics/8/8-163-1.jpg
https://danderm-pdv.is.kkh.dk/atlas/pics/8/8-164.jpg
https://danderm-pdv.is.kkh.dk/atlas/pics/8/8-165.jpg
https://danderm-pdv.is.kkh.dk/atlas/pics/8/8-166.jpg
https://danderm-pdv.is.kkh.dk/atlas/pics/8/8-167.jpg
https://danderm-pdv.is.kkh.dk/atlas/pics/8/8-168.jpg
https://danderm-pdv.is.kkh.dk/atlas/pics/8/8-168-1.jpg
https://danderm-pdv.is.kkh.dk/atlas/pics/8/8-169.jpg
https://danderm-pdv.is.kkh.dk/atlas/pics/8/8-169-1.jpg
https://danderm-pdv.is.kkh.dk/atlas/pics/8/8-169-2.jpg
https://danderm-pdv.is.kkh.dk/atlas/pics/8/8-170.jpg
https://danderm-pdv.is.kkh.dk/atlas/pics/8/8-170-1.jpg
https://danderm-pdv.is.kkh.dk/atlas/pics/8/8-171.jpg
https://danderm-pdv.is.kkh.dk/atlas/pics/8/8-172.jpg
https://danderm-pdv.is.kkh.dk/atlas/pics/8/8-173.jpg
https://danderm-pdv.is.kkh.dk/atlas/pics/8/8-174.jpg
https://danderm-pdv.is.kkh.dk/atlas/pics/8/8-174-1.jpg
https://danderm-pdv.is.kkh.dk/atlas/pics/8/8-174-2.jpg
https://danderm-pdv.is.kkh.dk/atlas/pics/8/8-175.jpg
https://danderm-pdv.is.kkh.dk/atlas/pics/8/8-176.jpg
https://danderm-pdv.is.kkh.dk/atlas/pics/8/8-177.jpg
https://danderm-pdv.is.kkh.dk/atlas/pics/8/8-179.jpg
https://danderm-pdv.is.kkh.dk/atlas/pics/8/8-180.jpg
https://danderm-pdv.is.kkh.dk/atlas/pics/8/8-181.jpg
https://danderm-pdv.is.kkh.dk/atlas/pics/8/8-182-1.jpg
https://danderm-pdv.is.kkh.dk/atlas/pics/8/8-183.jpg
https://danderm-pdv.is.kkh.dk/atlas/pics/8/8-184.jpg
https://danderm-pdv.is.kkh.dk/atlas/pics/8/8-185.jpg
https://danderm-pdv.is.kkh.dk/atlas/pics/8/8-186.jpg
https://danderm-pdv.is.kkh.dk/atlas/pics/8/8-187.jpg
https://danderm-pdv.is.kkh.dk/atlas/pics/8/8-188.jpg
https://danderm-pdv.is.kkh.dk/atlas/pics/8/8-189.jpg
https://danderm-pdv.is.kkh.dk/atlas/pics/8/8-190.jpg
https://danderm-pdv.is.kkh.dk/atlas/pics/8/8-191.jpg
https://danderm-pdv.is.kkh.dk/atlas/pics/8/8-192.jpg
https://danderm-pdv.is.kkh.dk/atlas/pics/8/8-192-1.jpg
https://danderm-pdv.is.kkh.dk/atlas/pics/8/8-192-2.jpg
https://danderm-pdv.is.kkh.dk/atlas/pics/8/8-193.jpg
https://danderm-pdv.is.kkh.dk/atlas/pics/8/8-194.jpg
https://danderm-pdv.is.kkh.dk/atlas/pics/8/8-195.jpg
https://danderm-pdv.is.kkh.dk/atlas/pics/8/8-195-1.jpg
https://danderm-pdv.is.kkh.dk/atlas/pics/8/8-195-2.jpg
https://danderm-pdv.is.kkh.dk/atlas/pics/8/8-196-1.jpg

https://danderm-pdv.is.kkh.dk/atlas/pics/8/8-196-2.jpg
https://danderm-pdv.is.kkh.dk/atlas/pics/8/8-196-3.jpg
https://danderm-pdv.is.kkh.dk/atlas/pics/8/8-196_S.jpg
https://danderm-pdv.is.kkh.dk/atlas/pics/8/8-197_S.jpg
https://danderm-pdv.is.kkh.dk/atlas/pics/8/8-198_S.jpg
https://danderm-pdv.is.kkh.dk/atlas/pics/8/8-199.jpg
https://danderm-pdv.is.kkh.dk/atlas/pics/8/8-199-1.jpg
https://danderm-pdv.is.kkh.dk/atlas/pics/8/8-199-2.jpg
https://danderm-pdv.is.kkh.dk/atlas/pics/8/8-199-3.jpg
https://danderm-pdv.is.kkh.dk/atlas/pics/8/8-199-4.jpg
https://danderm-pdv.is.kkh.dk/atlas/pics/8/8-199-5.jpg
https://danderm-pdv.is.kkh.dk/atlas/pics/9/9-1.jpg
https://danderm-pdv.is.kkh.dk/atlas/pics/9/9-1-1.jpg
https://danderm-pdv.is.kkh.dk/atlas/pics/9/9-1-2.jpg
https://danderm-pdv.is.kkh.dk/atlas/pics/9/9-1-3.jpg
https://danderm-pdv.is.kkh.dk/atlas/pics/9/9-2.jpg
https://danderm-pdv.is.kkh.dk/atlas/pics/9/9-3.jpg
https://danderm-pdv.is.kkh.dk/atlas/pics/9/9-3-1.jpg
https://danderm-pdv.is.kkh.dk/atlas/pics/9/9-3-2.jpg
https://danderm-pdv.is.kkh.dk/atlas/pics/9/9-3-3.jpg
https://danderm-pdv.is.kkh.dk/atlas/pics/9/9-3-4.jpg
https://danderm-pdv.is.kkh.dk/atlas/pics/9/9-3-5.jpg
https://danderm-pdv.is.kkh.dk/atlas/pics/9/9-3-6.jpg
https://danderm-pdv.is.kkh.dk/atlas/pics/9/9-4.jpg
https://danderm-pdv.is.kkh.dk/atlas/pics/9/9-4-1.jpg
https://danderm-pdv.is.kkh.dk/atlas/pics/9/9-4-1-1.jpg
https://danderm-pdv.is.kkh.dk/atlas/pics/9/9-4-1-2.jpg
https://danderm-pdv.is.kkh.dk/atlas/pics/9/9-4-2.jpg
https://danderm-pdv.is.kkh.dk/atlas/pics/9/9-4-3.jpg
https://danderm-pdv.is.kkh.dk/atlas/pics/9/9-4-4.jpg
https://danderm-pdv.is.kkh.dk/atlas/pics/9/9-5-0.jpg
https://danderm-pdv.is.kkh.dk/atlas/pics/9/9-5.jpg
https://danderm-pdv.is.kkh.dk/atlas/pics/9/9-6.jpg
https://danderm-pdv.is.kkh.dk/atlas/pics/9/9-7.jpg
https://danderm-pdv.is.kkh.dk/atlas/pics/9/9-7-1.jpg
https://danderm-pdv.is.kkh.dk/atlas/pics/9/9-7-2.jpg
https://danderm-pdv.is.kkh.dk/atlas/pics/9/9-7-3.jpg
https://danderm-pdv.is.kkh.dk/atlas/pics/9/9-7-4.jpg
https://danderm-pdv.is.kkh.dk/atlas/pics/9/9-7-5.jpg
https://danderm-pdv.is.kkh.dk/atlas/pics/9/9-7-6.jpg
https://danderm-pdv.is.kkh.dk/atlas/pics/9/9-8.jpg
https://danderm-pdv.is.kkh.dk/atlas/pics/9/9-9.jpg
https://danderm-pdv.is.kkh.dk/atlas/pics/9/9-10.jpg
https://danderm-pdv.is.kkh.dk/atlas/pics/9/9-10-1.jpg
https://danderm-pdv.is.kkh.dk/atlas/pics/9/9-10-1-1.jpg
https://danderm-pdv.is.kkh.dk/atlas/pics/9/9-10-1-2.jpg
https://danderm-pdv.is.kkh.dk/atlas/pics/9/9-10-1-3.jpg
https://danderm-pdv.is.kkh.dk/atlas/pics/9/9-10-1-5.jpg

https://danderm-pdv.is.kkh.dk/atlas/pics/9/9-10-1-6.jpg
https://danderm-pdv.is.kkh.dk/atlas/pics/9/9-10-1-7.jpg
https://danderm-pdv.is.kkh.dk/atlas/pics/9/9-10-1-8.jpg
https://danderm-pdv.is.kkh.dk/atlas/pics/9/9-10-1-9.jpg
https://danderm-pdv.is.kkh.dk/atlas/pics/9/9-10-1-10.jpg
https://danderm-pdv.is.kkh.dk/atlas/pics/9/9-10-2.jpg
https://danderm-pdv.is.kkh.dk/atlas/pics/9/9-10-3.jpg
https://danderm-pdv.is.kkh.dk/atlas/pics/9/9-10-4.jpg
https://danderm-pdv.is.kkh.dk/atlas/pics/9/9-10-5.jpg
https://danderm-pdv.is.kkh.dk/atlas/pics/9/9-10-6.jpg
https://danderm-pdv.is.kkh.dk/atlas/pics/9/9-10-7.jpg
https://danderm-pdv.is.kkh.dk/atlas/pics/9/9-10-7-1.jpg
https://danderm-pdv.is.kkh.dk/atlas/pics/9/9-10-7-2.jpg
https://danderm-pdv.is.kkh.dk/atlas/pics/9/9-10-7-3.jpg
https://danderm-pdv.is.kkh.dk/atlas/pics/9/9-10-7-4.jpg
https://danderm-pdv.is.kkh.dk/atlas/pics/9/9-10-7-5.jpg
https://danderm-pdv.is.kkh.dk/atlas/pics/9/9-10-8.jpg
https://danderm-pdv.is.kkh.dk/atlas/pics/9/9-10-9.jpg
https://danderm-pdv.is.kkh.dk/atlas/pics/9/9-11.jpg
https://danderm-pdv.is.kkh.dk/atlas/pics/9/9-12.jpg
https://danderm-pdv.is.kkh.dk/atlas/pics/9/9-13.jpg
https://danderm-pdv.is.kkh.dk/atlas/pics/9/9-13-1.jpg
https://danderm-pdv.is.kkh.dk/atlas/pics/9/9-13-2.jpg
https://danderm-pdv.is.kkh.dk/atlas/pics/9/9-14.jpg
https://danderm-pdv.is.kkh.dk/atlas/pics/9/9-14-1.jpg
https://danderm-pdv.is.kkh.dk/atlas/pics/9/9-14-2.jpg
https://danderm-pdv.is.kkh.dk/atlas/pics/9/9-14-3.jpg
https://danderm-pdv.is.kkh.dk/atlas/pics/9/9-15.jpg
https://danderm-pdv.is.kkh.dk/atlas/pics/9/9-16.jpg
https://danderm-pdv.is.kkh.dk/atlas/pics/9/9-16-1.jpg
https://danderm-pdv.is.kkh.dk/atlas/pics/9/9-16-1-1.jpg
https://danderm-pdv.is.kkh.dk/atlas/pics/9/9-16-1-2.jpg
https://danderm-pdv.is.kkh.dk/atlas/pics/9/9-16-1-4.jpg
https://danderm-pdv.is.kkh.dk/atlas/pics/9/9-18.jpg
https://danderm-pdv.is.kkh.dk/atlas/pics/9/9-19.jpg
https://danderm-pdv.is.kkh.dk/atlas/pics/9/9-19-1.jpg
https://danderm-pdv.is.kkh.dk/atlas/pics/9/9-19-2.jpg
https://danderm-pdv.is.kkh.dk/atlas/pics/9/9-19-3.jpg
https://danderm-pdv.is.kkh.dk/atlas/pics/9/9-19-4.jpg
https://danderm-pdv.is.kkh.dk/atlas/pics/9/9-19-5.jpg
https://danderm-pdv.is.kkh.dk/atlas/pics/9/9-20.jpg
https://danderm-pdv.is.kkh.dk/atlas/pics/9/9-20-1.jpg
https://danderm-pdv.is.kkh.dk/atlas/pics/9/9-20-2.jpg
https://danderm-pdv.is.kkh.dk/atlas/pics/9/9-20-3.jpg
https://danderm-pdv.is.kkh.dk/atlas/pics/9/9-21.jpg
https://danderm-pdv.is.kkh.dk/atlas/pics/9/9-21-2.jpg
https://danderm-pdv.is.kkh.dk/atlas/pics/9/9-21-3.jpg
https://danderm-pdv.is.kkh.dk/atlas/pics/9/9-21-4.jpg

https://danderm-pdv.is.kkh.dk/atlas/pics/9/9-22.jpg
https://danderm-pdv.is.kkh.dk/atlas/pics/9/9-22-2.jpg
https://danderm-pdv.is.kkh.dk/atlas/pics/9/9-22-3.jpg
https://danderm-pdv.is.kkh.dk/atlas/pics/9/9-22-4.jpg
https://danderm-pdv.is.kkh.dk/atlas/pics/9/9-23.jpg
https://danderm-pdv.is.kkh.dk/atlas/pics/9/9-23-1.jpg
https://danderm-pdv.is.kkh.dk/atlas/pics/9/9-23-2.jpg
https://danderm-pdv.is.kkh.dk/atlas/pics/9/9-23-3.jpg
https://danderm-pdv.is.kkh.dk/atlas/pics/1/1-1.jpg
https://danderm-pdv.is.kkh.dk/atlas/pics/1/1-10.jpg
https://danderm-pdv.is.kkh.dk/atlas/pics/1/1-2.jpg
https://danderm-pdv.is.kkh.dk/atlas/pics/1/1-3_s.jpg
https://danderm-pdv.is.kkh.dk/atlas/pics/1/1-4.jpg
https://danderm-pdv.is.kkh.dk/atlas/pics/1/1-5-1.jpg
https://danderm-pdv.is.kkh.dk/atlas/pics/1/1-5-2.jpg
https://danderm-pdv.is.kkh.dk/atlas/pics/1/1-5-3.jpg
https://danderm-pdv.is.kkh.dk/atlas/pics/1/1-5-4.jpg
https://danderm-pdv.is.kkh.dk/atlas/pics/1/1-5-5.jpg
https://danderm-pdv.is.kkh.dk/atlas/pics/1/1-5-6.jpg
https://danderm-pdv.is.kkh.dk/atlas/pics/1/1-5-7.jpg
https://danderm-pdv.is.kkh.dk/atlas/pics/1/1-5.jpg
https://danderm-pdv.is.kkh.dk/atlas/pics/1/1-6.jpg
https://danderm-pdv.is.kkh.dk/atlas/pics/1/1-7.jpg
https://danderm-pdv.is.kkh.dk/atlas/pics/1/1-8-1.jpg
https://danderm-pdv.is.kkh.dk/atlas/pics/1/1-8.jpg
https://danderm-pdv.is.kkh.dk/atlas/pics/1/1-9.jpg
https://danderm-pdv.is.kkh.dk/atlas/pics/2/2-103.jpg
https://danderm-pdv.is.kkh.dk/atlas/pics/2/2-108-5.jpg
https://danderm-pdv.is.kkh.dk/atlas/pics/2/2-12.jpg
https://danderm-pdv.is.kkh.dk/atlas/pics/2/2-13.jpg
https://danderm-pdv.is.kkh.dk/atlas/pics/2/2-138.jpg
https://danderm-pdv.is.kkh.dk/atlas/pics/2/2-145.jpg
https://danderm-pdv.is.kkh.dk/atlas/pics/2/2-27-6.jpg
https://danderm-pdv.is.kkh.dk/atlas/pics/2/2-27-7.jpg
https://danderm-pdv.is.kkh.dk/atlas/pics/2/2-29-1.jpg
https://danderm-pdv.is.kkh.dk/atlas/pics/2/2-33.jpg
https://danderm-pdv.is.kkh.dk/atlas/pics/2/2-58-1.jpg
https://danderm-pdv.is.kkh.dk/atlas/pics/2/2-60.jpg
https://danderm-pdv.is.kkh.dk/atlas/pics/2/2-90.jpg
https://danderm-pdv.is.kkh.dk/atlas/pics/2/2-91-1.jpg
https://danderm-pdv.is.kkh.dk/atlas/pics/2/2-91-2.jpg
https://danderm-pdv.is.kkh.dk/atlas/pics/2/2-91-3.jpg
https://danderm-pdv.is.kkh.dk/atlas/pics/2/2-91.jpg
https://danderm-pdv.is.kkh.dk/atlas/pics/2/2-92.jpg
https://danderm-pdv.is.kkh.dk/atlas/pics/2/2-93.jpg
https://danderm-pdv.is.kkh.dk/atlas/pics/2/2-94.jpg
https://danderm-pdv.is.kkh.dk/atlas/pics/2/2-95-1.jpg
https://danderm-pdv.is.kkh.dk/atlas/pics/2/2-95-2.jpg

https://danderm-pdv.is.kkh.dk/atlas/pics/2/2-95.jpg
https://danderm-pdv.is.kkh.dk/atlas/pics/3/3-103.jpg
https://danderm-pdv.is.kkh.dk/atlas/pics/3/3-104.jpg
https://danderm-pdv.is.kkh.dk/atlas/pics/3/3-105.jpg
https://danderm-pdv.is.kkh.dk/atlas/pics/3/3-119-5.jpg
https://danderm-pdv.is.kkh.dk/atlas/pics/3/3-119-7a.jpg
https://danderm-pdv.is.kkh.dk/atlas/pics/3/3-210.jpg
https://danderm-pdv.is.kkh.dk/atlas/pics/3/3-211.jpg
https://danderm-pdv.is.kkh.dk/atlas/pics/3/3-212.jpg
https://danderm-pdv.is.kkh.dk/atlas/pics/3/3-213.jpg
https://danderm-pdv.is.kkh.dk/atlas/pics/3/3-214.jpg
https://danderm-pdv.is.kkh.dk/atlas/pics/3/3-215.jpg
https://danderm-pdv.is.kkh.dk/atlas/pics/3/3-216.jpg
https://danderm-pdv.is.kkh.dk/atlas/pics/3/3-218.jpg
https://danderm-pdv.is.kkh.dk/atlas/pics/3/3-219.jpg
https://danderm-pdv.is.kkh.dk/atlas/pics/3/3-220.jpg
https://danderm-pdv.is.kkh.dk/atlas/pics/3/3-221.jpg
https://danderm-pdv.is.kkh.dk/atlas/pics/3/3-222.jpg
https://danderm-pdv.is.kkh.dk/atlas/pics/3/3-223.jpg
https://danderm-pdv.is.kkh.dk/atlas/pics/3/3-224.jpg
https://danderm-pdv.is.kkh.dk/atlas/pics/3/3-225.jpg
https://danderm-pdv.is.kkh.dk/atlas/pics/3/3-226.jpg
https://danderm-pdv.is.kkh.dk/atlas/pics/3/3-227.jpg
https://danderm-pdv.is.kkh.dk/atlas/pics/3/3-228.jpg
https://danderm-pdv.is.kkh.dk/atlas/pics/3/3-229.jpg
https://danderm-pdv.is.kkh.dk/atlas/pics/3/3-230.jpg
https://danderm-pdv.is.kkh.dk/atlas/pics/3/3-231.jpg
https://danderm-pdv.is.kkh.dk/atlas/pics/3/3-232.jpg
https://danderm-pdv.is.kkh.dk/atlas/pics/3/3-233.jpg
https://danderm-pdv.is.kkh.dk/atlas/pics/3/3-234.jpg
https://danderm-pdv.is.kkh.dk/atlas/pics/3/3-235.jpg
https://danderm-pdv.is.kkh.dk/atlas/pics/3/3-236.jpg
https://danderm-pdv.is.kkh.dk/atlas/pics/3/3-237.jpg
https://danderm-pdv.is.kkh.dk/atlas/pics/3/3-238.jpg
https://danderm-pdv.is.kkh.dk/atlas/pics/3/3-239.jpg
https://danderm-pdv.is.kkh.dk/atlas/pics/3/3-240.jpg
https://danderm-pdv.is.kkh.dk/atlas/pics/3/3-241.jpg
https://danderm-pdv.is.kkh.dk/atlas/pics/3/3-242.jpg
https://danderm-pdv.is.kkh.dk/atlas/pics/3/3-243.jpg
https://danderm-pdv.is.kkh.dk/atlas/pics/3/3-244.jpg
https://danderm-pdv.is.kkh.dk/atlas/pics/3/3-245.jpg
https://danderm-pdv.is.kkh.dk/atlas/pics/3/3-246.jpg
https://danderm-pdv.is.kkh.dk/atlas/pics/3/3-247.jpg
https://danderm-pdv.is.kkh.dk/atlas/pics/3/3-248.jpg
https://danderm-pdv.is.kkh.dk/atlas/pics/3/3-249.jpg
https://danderm-pdv.is.kkh.dk/atlas/pics/3/3-250.jpg
https://danderm-pdv.is.kkh.dk/atlas/pics/3/3-251.jpg
https://danderm-pdv.is.kkh.dk/atlas/pics/3/3-252.jpg

https://danderm-pdv.is.kkh.dk/atlas/pics/3/3-253.jpg
https://danderm-pdv.is.kkh.dk/atlas/pics/4/4-11.jpg
https://danderm-pdv.is.kkh.dk/atlas/pics/4/4-120.jpg
https://danderm-pdv.is.kkh.dk/atlas/pics/4/4-127-1.jpg
https://danderm-pdv.is.kkh.dk/atlas/pics/4/4-127-2.jpg
https://danderm-pdv.is.kkh.dk/atlas/pics/4/4-127-3.jpg
https://danderm-pdv.is.kkh.dk/atlas/pics/4/4-127-4.jpg
https://danderm-pdv.is.kkh.dk/atlas/pics/4/4-127-5.jpg
https://danderm-pdv.is.kkh.dk/atlas/pics/4/4-127-6.jpg
https://danderm-pdv.is.kkh.dk/atlas/pics/4/4-127-7.jpg
https://danderm-pdv.is.kkh.dk/atlas/pics/4/4-127-8.jpg
https://danderm-pdv.is.kkh.dk/atlas/pics/4/4-127.jpg
https://danderm-pdv.is.kkh.dk/atlas/pics/4/4-132_S.jpg
https://danderm-pdv.is.kkh.dk/atlas/pics/4/4-167.jpg
https://danderm-pdv.is.kkh.dk/atlas/pics/4/4-168-1.jpg
https://danderm-pdv.is.kkh.dk/atlas/pics/4/4-168.jpg
https://danderm-pdv.is.kkh.dk/atlas/pics/4/4-20.jpg
https://danderm-pdv.is.kkh.dk/atlas/pics/4/4-69-1.jpg
https://danderm-pdv.is.kkh.dk/atlas/pics/4/4-69-2.jpg
https://danderm-pdv.is.kkh.dk/atlas/pics/4/4-69-3.jpg
https://danderm-pdv.is.kkh.dk/atlas/pics/4/4-69-4.jpg
https://danderm-pdv.is.kkh.dk/atlas/pics/4/4-72.jpg
https://danderm-pdv.is.kkh.dk/atlas/pics/4/4-73.jpg
https://danderm-pdv.is.kkh.dk/atlas/pics/4/4-74.jpg
https://danderm-pdv.is.kkh.dk/atlas/pics/4/4-75.jpg
https://danderm-pdv.is.kkh.dk/atlas/pics/4/4-76.jpg
https://danderm-pdv.is.kkh.dk/atlas/pics/5/5-115.jpg
https://danderm-pdv.is.kkh.dk/atlas/pics/5/5-116.jpg
https://danderm-pdv.is.kkh.dk/atlas/pics/5/5-119.jpg
https://danderm-pdv.is.kkh.dk/atlas/pics/5/5-120.jpg
https://danderm-pdv.is.kkh.dk/atlas/pics/5/5-139-1.jpg
https://danderm-pdv.is.kkh.dk/atlas/pics/5/5-139-2.jpg
https://danderm-pdv.is.kkh.dk/atlas/pics/5/5-139-3.jpg
https://danderm-pdv.is.kkh.dk/atlas/pics/5/5-139-4.jpg
https://danderm-pdv.is.kkh.dk/atlas/pics/5/5-139-5.jpg
https://danderm-pdv.is.kkh.dk/atlas/pics/5/5-139-6.jpg
https://danderm-pdv.is.kkh.dk/atlas/pics/5/5-139-7.jpg
https://danderm-pdv.is.kkh.dk/atlas/pics/5/5-38.jpg
https://danderm-pdv.is.kkh.dk/atlas/pics/5/5-39.jpg
https://danderm-pdv.is.kkh.dk/atlas/pics/5/5-40.jpg
https://danderm-pdv.is.kkh.dk/atlas/pics/5/5-42-1.jpg
https://danderm-pdv.is.kkh.dk/atlas/pics/5/5-69-5.jpg
https://danderm-pdv.is.kkh.dk/atlas/pics/5/5-69-6.jpg
https://danderm-pdv.is.kkh.dk/atlas/pics/5/5-86-2.jpg
https://danderm-pdv.is.kkh.dk/atlas/pics/5/5-88-1.jpg
https://danderm-pdv.is.kkh.dk/atlas/pics/5/5-88-2.jpg
https://danderm-pdv.is.kkh.dk/atlas/pics/5/5-88-3.jpg
https://danderm-pdv.is.kkh.dk/atlas/pics/5/5-88-4.jpg

https://danderm-pdv.is.kkh.dk/atlas/pics/5/5-88-5.jpg
https://danderm-pdv.is.kkh.dk/atlas/pics/5/5-88-6.jpg
https://danderm-pdv.is.kkh.dk/atlas/pics/5/5-88-7.jpg
https://danderm-pdv.is.kkh.dk/atlas/pics/5/5-88-8.jpg
https://danderm-pdv.is.kkh.dk/atlas/pics/6/6-52-1-1.jpg
https://danderm-pdv.is.kkh.dk/atlas/pics/6/6-52-1-2.jpg
https://danderm-pdv.is.kkh.dk/atlas/pics/6/6-52-1-3.jpg
https://danderm-pdv.is.kkh.dk/atlas/pics/6/6-91.jpg
https://danderm-pdv.is.kkh.dk/atlas/pics/6/6-92.jpg
https://danderm-pdv.is.kkh.dk/atlas/pics/7/7-111.jpg
https://danderm-pdv.is.kkh.dk/atlas/pics/7/7-123-1.jpg
https://danderm-pdv.is.kkh.dk/atlas/pics/7/7-140-1.jpg
https://danderm-pdv.is.kkh.dk/atlas/pics/7/7-140-2.jpg
https://danderm-pdv.is.kkh.dk/atlas/pics/7/7-149.jpg
https://danderm-pdv.is.kkh.dk/atlas/pics/7/7-167-8.jpg
https://danderm-pdv.is.kkh.dk/atlas/pics/7/7-180.jpg
https://danderm-pdv.is.kkh.dk/atlas/pics/7/7-23.jpg
https://danderm-pdv.is.kkh.dk/atlas/pics/7/7-47.jpg
https://danderm-pdv.is.kkh.dk/atlas/pics/7/7-59.jpg
https://danderm-pdv.is.kkh.dk/atlas/pics/7/7-60.jpg
https://danderm-pdv.is.kkh.dk/atlas/pics/7/7-70.jpg
https://danderm-pdv.is.kkh.dk/atlas/pics/7/7-97.jpg
https://danderm-pdv.is.kkh.dk/atlas/pics/8/8-107.jpg
https://danderm-pdv.is.kkh.dk/atlas/pics/8/8-182.jpg
https://danderm-pdv.is.kkh.dk/atlas/pics/8/8-39.jpg
https://danderm-pdv.is.kkh.dk/atlas/pics/8/8-98.jpg
https://danderm-pdv.is.kkh.dk/atlas/pics/8/8-99.jpg
https://danderm-pdv.is.kkh.dk/atlas/pics/9/9-17.jpg
https://www.dermis.net/bilder/CD044/550px/img0082.jpg
https://www.dermis.net/bilder/CD014/550px/img0028.jpg
https://www.dermis.net/bilder/CD039/550px/img0029.jpg
https://www.dermis.net/bilder/CD071/550px/img0057.jpg
https://www.dermis.net/bilder/CD042/550px/img0079.jpg
https://www.dermis.net/bilder/CD040/550px/img0068.jpg
https://www.dermis.net/bilder/CD054/550px/img0016.jpg
https://www.dermis.net/bilder/CD050/550px/img0093.jpg
https://www.dermis.net/bilder/CD050/550px/img0094.jpg
https://www.dermis.net/bilder/CD050/550px/img0095.jpg
https://www.dermis.net/bilder/CD070/550px/img0090.jpg
https://www.dermis.net/bilder/CD069/550px/img0116.jpg
https://www.dermis.net/bilder/CD044/550px/img0085.jpg
https://www.dermis.net/bilder/CD071/550px/img0039.jpg
https://www.dermis.net/bilder/CD071/550px/img0056.jpg
https://www.dermis.net/bilder/CD025/550px/img0044.jpg
https://www.dermis.net/bilder/CD049/550px/img0101.jpg
https://www.dermis.net/bilder/CD049/550px/img0102.jpg
https://www.dermis.net/bilder/CD070/550px/img0089.jpg
https://www.dermis.net/bilder/CD069/550px/img0114.jpg

https://www.dermis.net/bilder/CD005/550px/img0103.jpg
https://www.dermis.net/bilder/CD071/550px/img0113.jpg
https://www.dermis.net/bilder/CD071/550px/img0052.jpg
https://www.dermis.net/bilder/CD054/550px/img0063.jpg
https://www.dermis.net/bilder/CD054/550px/img0064.jpg
https://www.dermis.net/bilder/CD001/550px/img0090.jpg
https://www.dermis.net/bilder/CD054/550px/img0062.jpg
https://www.dermis.net/bilder/CD070/550px/img0017.jpg
https://www.dermis.net/bilder/CD018/550px/img0003.jpg
https://www.dermis.net/bilder/CD001/550px/img0042.jpg
https://www.dermis.net/bilder/CD070/550px/img0018.jpg
https://www.dermis.net/bilder/CD071/550px/img0025.jpg
https://www.dermis.net/bilder/CD069/550px/img0036.jpg
https://www.dermis.net/bilder/CD071/550px/img0099.jpg
https://www.dermis.net/bilder/CD041/550px/img0010.jpg
https://www.dermis.net/bilder/CD018/550px/img0047.jpg
https://www.dermis.net/bilder/CD071/550px/img0073.jpg
https://www.dermis.net/bilder/CD001/550px/img0051.jpg
https://www.dermis.net/bilder/CD040/550px/img0087.jpg
https://www.dermis.net/bilder/CD040/550px/img0088.jpg
https://www.dermis.net/bilder/CD070/550px/img0021.jpg
https://www.dermis.net/bilder/CD020/550px/img0004.jpg
https://www.dermis.net/bilder/CD070/550px/img0015.jpg
https://www.dermis.net/bilder/CD070/550px/img0016.jpg
https://www.dermis.net/bilder/CD020/550px/img0003.jpg
https://www.dermis.net/bilder/CD044/550px/img0064.jpg
https://www.dermis.net/bilder/CD014/550px/img0085.jpg
https://www.dermis.net/bilder/CD018/550px/img0106.jpg
https://www.dermis.net/bilder/CD056/550px/img0025.jpg
https://www.dermis.net/bilder/CD025/550px/img0050.jpg
https://www.dermis.net/bilder/CD040/550px/img0095.jpg
https://www.dermis.net/bilder/CD040/550px/img0096.jpg
https://www.dermis.net/bilder/CD014/550px/img0065.jpg
https://www.dermis.net/bilder/CD025/550px/img0051.jpg
https://www.dermis.net/bilder/CD039/550px/img0039.jpg
https://www.dermis.net/bilder/CD018/550px/img0069.jpg
https://www.dermis.net/bilder/CD034/550px/img0017.jpg
https://www.dermis.net/bilder/CD070/550px/img0029.jpg
https://www.dermis.net/bilder/CD006/550px/img0076.jpg
https://www.dermis.net/bilder/CD017/550px/img0087.jpg
https://www.dermis.net/bilder/CD070/550px/img0028.jpg
https://www.dermis.net/bilder/CD070/550px/img0026.jpg
https://www.dermis.net/bilder/CD070/550px/img0027.jpg
https://www.dermis.net/bilder/CD070/550px/img0033.jpg
https://www.dermis.net/bilder/CD034/550px/img0014.jpg
https://www.dermis.net/bilder/CD034/550px/img0015.jpg
https://www.dermis.net/bilder/CD047/550px/img0094.jpg
https://www.dermis.net/bilder/CD047/550px/img0095.jpg

https://www.dermis.net/bilder/CD047/550px/img0096.jpg
https://www.dermis.net/bilder/CD069/550px/img0115.jpg
https://www.dermis.net/bilder/CD016/550px/img0024.jpg
https://www.dermis.net/bilder/CD071/550px/img0083.jpg
https://www.dermis.net/bilder/CD071/550px/img0084.jpg
https://www.dermis.net/bilder/CD071/550px/img0085.jpg
https://www.dermis.net/bilder/CD070/550px/img0024.jpg
https://www.dermis.net/bilder/CD070/550px/img0025.jpg
https://www.dermis.net/bilder/CD071/550px/img0098.jpg
https://www.dermis.net/bilder/CD071/550px/img0080.jpg
https://www.dermis.net/bilder/CD071/550px/img0082.jpg
https://www.dermis.net/bilder/CD070/550px/img0031.jpg
https://www.dermis.net/bilder/CD071/550px/img0100.jpg
https://www.dermis.net/bilder/CD070/550px/img0030.jpg
https://www.dermis.net/bilder/CD071/550px/img0070.jpg
https://www.dermis.net/bilder/CD071/550px/img0027.jpg
https://www.dermis.net/bilder/CD071/550px/img0026.jpg
https://www.dermis.net/bilder/CD014/550px/img0050.jpg
https://www.dermis.net/bilder/CD071/550px/img0060.jpg
https://www.dermis.net/bilder/CD071/550px/img0061.jpg
https://www.dermis.net/bilder/CD004/550px/img0028.jpg
https://www.dermis.net/bilder/CD071/550px/img0096.jpg
https://www.dermis.net/bilder/CD071/550px/img0095.jpg
https://www.dermis.net/bilder/CD071/550px/img0111.jpg
https://www.dermis.net/bilder/CD057/550px/img0016.jpg
https://www.dermis.net/bilder/CD071/550px/img0046.jpg
https://www.dermis.net/bilder/CD018/550px/img0026.jpg
https://www.dermis.net/bilder/CD049/550px/img0085.jpg
https://www.dermis.net/bilder/CD018/550px/img0110.jpg
https://www.dermis.net/bilder/CD071/550px/img0109.jpg
https://www.dermis.net/bilder/CD069/550px/img0079.jpg
https://www.dermis.net/bilder/CD071/550px/img0071.jpg
https://www.dermis.net/bilder/CD071/550px/img0040.jpg
https://www.dermis.net/bilder/CD014/550px/img0052.jpg
https://www.dermis.net/bilder/CD015/550px/img0033.jpg
https://www.dermis.net/bilder/CD051/550px/img0041.jpg
https://www.dermis.net/bilder/CD037/550px/img0067.jpg
https://www.dermis.net/bilder/CD037/550px/img0068.jpg
https://www.dermis.net/bilder/CD037/550px/img0069.jpg
https://www.dermis.net/bilder/CD070/550px/img0004.jpg
https://www.dermis.net/bilder/CD018/550px/img0063.jpg
https://www.dermis.net/bilder/CD014/550px/img0077.jpg
https://www.dermis.net/bilder/CD071/550px/img0106.jpg
https://www.dermis.net/bilder/CD018/550px/img0033.jpg
https://www.dermis.net/bilder/CD055/550px/img0094.jpg
https://www.dermis.net/bilder/CD021/550px/img0084.jpg
https://www.dermis.net/bilder/CD069/550px/img0006.jpg
https://www.dermis.net/bilder/CD070/550px/img0095.jpg

https://www.dermis.net/bilder/CD026/550px/img0098.jpg
https://www.dermis.net/bilder/CD070/550px/img0001.jpg
https://www.dermis.net/bilder/CD026/550px/img0100.jpg
https://www.dermis.net/bilder/CD032/550px/img0051.jpg
https://www.dermis.net/bilder/CD032/550px/img0052.jpg
https://www.dermis.net/bilder/CD071/550px/img0090.jpg
https://www.dermis.net/bilder/CD054/550px/img0049.jpg
https://www.dermis.net/bilder/CD052/550px/img0064.jpg
https://www.dermis.net/bilder/CD054/550px/img0048.jpg
https://www.dermis.net/bilder/CD071/550px/img0089.jpg
https://www.dermis.net/bilder/CD071/550px/img0091.jpg
https://www.dermis.net/bilder/CD001/550px/img0085.jpg
https://www.dermis.net/bilder/CD037/550px/img0057.jpg
https://www.dermis.net/bilder/CD069/550px/img0049.jpg
https://www.dermis.net/bilder/CD070/550px/img0006.jpg
https://www.dermis.net/bilder/CD018/550px/img0050.jpg
https://www.dermis.net/bilder/CD071/550px/img0078.jpg
https://www.dermis.net/bilder/CD050/550px/img0019.jpg
https://www.dermis.net/bilder/CD050/550px/img0020.jpg
https://www.dermis.net/bilder/CD071/550px/img0112.jpg
https://www.dermis.net/bilder/CD036/550px/img0099.jpg
https://www.dermis.net/bilder/CD014/550px/img0081.jpg
https://www.dermis.net/bilder/CD014/550px/img0082.jpg
https://www.dermis.net/bilder/CD040/550px/img0052.jpg
https://www.dermis.net/bilder/CD071/550px/img0054.jpg
https://www.dermis.net/bilder/CD025/550px/img0094.jpg
https://www.dermis.net/bilder/CD071/550px/img0107.jpg
https://www.dermis.net/bilder/CD069/550px/img0041.jpg
https://www.dermis.net/bilder/CD069/550px/img0040.jpg
https://www.dermis.net/bilder/CD003/550px/img0064.jpg
https://www.dermis.net/bilder/CD016/550px/img0009.jpg
https://www.dermis.net/bilder/CD071/550px/img0105.jpg
https://www.dermis.net/bilder/CD071/550px/img0086.jpg
https://www.dermis.net/bilder/CD071/550px/img0087.jpg
https://www.dermis.net/bilder/CD018/550px/img0064.jpg
https://www.dermis.net/bilder/CD016/550px/img0055.jpg
https://www.dermis.net/bilder/CD050/550px/img0092.jpg
https://www.dermis.net/bilder/CD020/550px/img0045.jpg
https://www.dermis.net/bilder/CD056/550px/img0037.jpg
https://www.dermis.net/bilder/CD056/550px/img0038.jpg
https://www.dermis.net/bilder/CD018/550px/img0053.jpg
https://www.dermis.net/bilder/CD001/550px/img0068.jpg
https://www.dermis.net/bilder/CD047/550px/img0032.jpg
https://www.dermis.net/bilder/CD071/550px/img0007.jpg
https://www.dermis.net/bilder/CD003/550px/img0081.jpg
https://www.dermis.net/bilder/CD070/550px/img0032.jpg
https://www.dermis.net/bilder/CD014/550px/img0040.jpg
https://www.dermis.net/bilder/CD071/550px/img0094.jpg

https://www.dermis.net/bilder/CD073/550px/img0009.jpg
https://www.dermis.net/bilder/CD050/550px/img0068.jpg
https://www.dermis.net/bilder/CD048/550px/img0050.jpg
https://www.dermis.net/bilder/CD008/550px/img0041.jpg
https://www.dermis.net/bilder/CD024/550px/img0017.jpg
https://www.dermis.net/bilder/CD071/550px/img0051.jpg
https://www.dermis.net/bilder/CD071/550px/img0062.jpg
https://www.dermis.net/bilder/CD071/550px/img0042.jpg
https://www.dermis.net/bilder/CD014/550px/img0047.jpg
https://www.dermis.net/bilder/CD071/550px/img0043.jpg
https://www.dermis.net/bilder/CD015/550px/img0026.jpg
https://www.dermis.net/bilder/CD005/550px/img0066.jpg
https://www.dermis.net/bilder/CD046/550px/img0013.jpg
https://www.dermis.net/bilder/CD046/550px/img0020.jpg
https://www.dermis.net/bilder/CD005/550px/img0065.jpg
https://www.dermis.net/bilder/CD071/550px/img0077.jpg
https://www.dermis.net/bilder/CD036/550px/img0105.jpg
https://www.dermis.net/bilder/CD071/550px/img0103.jpg
https://www.dermis.net/bilder/CD036/550px/img0090.jpg
https://www.dermis.net/bilder/CD014/550px/img0080.jpg
https://www.dermis.net/bilder/CD015/550px/img0031.jpg
https://www.dermis.net/bilder/CD014/550px/img0076.jpg
https://www.dermis.net/bilder/CD041/550px/img0076.jpg
https://www.dermis.net/bilder/CD001/550px/img0009.jpg
https://www.dermis.net/bilder/CD056/550px/img0031.jpg
https://www.dermis.net/bilder/CD071/550px/img0088.jpg
https://www.dermis.net/bilder/CD014/550px/img0057.jpg
https://www.dermis.net/bilder/CD036/550px/img0086.jpg
https://www.dermis.net/bilder/CD046/550px/img0079.jpg
https://www.dermis.net/bilder/CD046/550px/img0080.jpg
https://www.dermis.net/bilder/CD071/550px/img0075.jpg
https://www.dermis.net/bilder/CD071/550px/img0076.jpg
https://www.dermis.net/bilder/CD071/550px/img0101.jpg
https://www.dermis.net/bilder/CD071/550px/img0102.jpg
https://www.dermis.net/bilder/CD028/550px/img0079.jpg
https://www.dermis.net/bilder/CD020/550px/img0074.jpg
https://www.dermis.net/bilder/CD020/550px/img0075.jpg
https://www.dermis.net/bilder/CD020/550px/img0072.jpg
https://www.dermis.net/bilder/CD020/550px/img0073.jpg
https://www.dermis.net/bilder/CD069/550px/img0044.jpg
https://www.dermis.net/bilder/CD069/550px/img0045.jpg
https://www.dermis.net/bilder/CD048/550px/img0083.jpg
https://www.dermis.net/bilder/CD001/550px/img0054.jpg
https://www.dermis.net/bilder/CD021/550px/img0076.jpg
https://www.dermis.net/bilder/CD023/550px/img0055.jpg
https://www.dermis.net/bilder/CD023/550px/img0056.jpg
https://www.dermis.net/bilder/CD006/550px/img0055.jpg
https://www.dermis.net/bilder/CD005/550px/img0040.jpg

https://www.dermis.net/bilder/CD023/550px/img0057.jpg
https://www.dermis.net/bilder/CD018/550px/img0102.jpg
https://www.dermis.net/bilder/CD004/550px/img0032.jpg
https://www.dermis.net/bilder/CD001/550px/img0004.jpg
https://www.dermis.net/bilder/CD018/550px/img0057.jpg
https://www.dermis.net/bilder/CD052/550px/img0100.jpg
https://www.dermis.net/bilder/CD006/550px/img0036.jpg
https://www.dermis.net/bilder/CD024/550px/img0007.jpg
https://www.dermis.net/bilder/CD024/550px/img0008.jpg
https://www.dermis.net/bilder/CD019/550px/img0005.jpg
https://www.dermis.net/bilder/CD071/550px/img0079.jpg
https://www.dermis.net/bilder/CD054/550px/img0100.jpg
https://www.dermis.net/bilder/CD016/550px/img0010.jpg
https://www.dermis.net/bilder/CD056/550px/img0036.jpg
https://www.dermis.net/bilder/CD012/550px/img0084.jpg
https://www.dermis.net/bilder/CD027/550px/img0078.jpg
https://www.dermis.net/bilder/CD036/550px/img0104.jpg
https://www.dermis.net/bilder/CD071/550px/img0104.jpg
https://www.dermis.net/bilder/CD027/550px/img0079.jpg
https://www.dermis.net/bilder/CD027/550px/img0080.jpg
https://www.dermis.net/bilder/CD070/550px/img0081.jpg
https://www.dermis.net/bilder/CD070/550px/img0082.jpg
https://www.dermis.net/bilder/CD048/550px/img0081.jpg
https://www.dermis.net/bilder/CD048/550px/img0082.jpg
https://www.dermis.net/bilder/CD021/550px/img0067.jpg
https://www.dermis.net/bilder/CD008/550px/img0063.jpg
https://www.dermis.net/bilder/CD014/550px/img0038.jpg
https://www.dermis.net/bilder/CD014/550px/img0048.jpg
https://www.dermis.net/bilder/CD034/550px/img0040.jpg
https://www.dermis.net/bilder/CD071/550px/img0058.jpg
https://www.dermis.net/bilder/CD040/550px/img0083.jpg
https://www.dermis.net/bilder/CD071/550px/img0059.jpg
https://www.dermis.net/bilder/CD049/550px/img0024.jpg
https://www.dermis.net/bilder/CD015/550px/img0046.jpg
https://www.dermis.net/bilder/CD033/550px/img0078.jpg
https://www.dermis.net/bilder/CD006/550px/img0031.jpg
https://www.dermis.net/bilder/CD035/550px/img0058.jpg
https://www.dermis.net/bilder/CD035/550px/img0059.jpg
https://www.dermis.net/bilder/CD039/550px/img0007.jpg
https://www.dermis.net/bilder/CD049/550px/img0038.jpg
https://www.dermis.net/bilder/CD049/550px/img0039.jpg
https://www.dermis.net/bilder/CD035/550px/img0089.jpg
https://www.dermis.net/bilder/CD037/550px/img0028.jpg
https://www.dermis.net/bilder/CD058/550px/img0017.jpg
https://www.dermis.net/bilder/CD058/550px/img0018.jpg
https://www.dermis.net/bilder/CD041/550px/img0004.jpg
https://www.dermis.net/bilder/CD041/550px/img0005.jpg
https://www.dermis.net/bilder/CD041/550px/img0006.jpg

https://www.dermis.net/bilder/CD039/550px/img0010.jpg
https://www.dermis.net/bilder/CD004/550px/img0016.jpg
https://www.dermis.net/bilder/CD004/550px/img0017.jpg
https://www.dermis.net/bilder/CD025/550px/img0040.jpg
https://www.dermis.net/bilder/CD049/550px/img0040.jpg
https://www.dermis.net/bilder/CD049/550px/img0041.jpg
https://www.dermis.net/bilder/CD016/550px/img0014.jpg
https://www.dermis.net/bilder/CD006/550px/img0079.jpg
https://www.dermis.net/bilder/CD026/550px/img0052.jpg
https://www.dermis.net/bilder/CD014/550px/img0075.jpg
https://www.dermis.net/bilder/CD026/550px/img0051.jpg
https://www.dermis.net/bilder/CD071/550px/img0114.jpg
https://www.dermis.net/bilder/CD071/550px/img0115.jpg
https://www.dermis.net/bilder/CD023/550px/img0048.jpg
https://www.dermis.net/bilder/CD071/550px/img0022.jpg
https://www.dermis.net/bilder/CD071/550px/img0041.jpg
https://www.dermis.net/bilder/CD016/550px/img0006.jpg
https://www.dermis.net/bilder/CD055/550px/img0085.jpg
https://www.dermis.net/bilder/CD051/550px/img0058.jpg
https://www.dermis.net/bilder/CD037/550px/img0031.jpg
https://www.dermis.net/bilder/CD048/550px/img0090.jpg
https://www.dermis.net/bilder/CD048/550px/img0091.jpg
https://www.dermis.net/bilder/CD036/550px/img0058.jpg
https://www.dermis.net/bilder/CD049/550px/img0025.jpg
https://www.dermis.net/bilder/CD049/550px/img0026.jpg
https://www.dermis.net/bilder/CD049/550px/img0027.jpg
https://www.dermis.net/bilder/CD049/550px/img0028.jpg
https://www.dermis.net/bilder/CD049/550px/img0031.jpg
https://www.dermis.net/bilder/CD049/550px/img0032.jpg
https://www.dermis.net/bilder/CD049/550px/img0033.jpg
https://www.dermis.net/bilder/CD049/550px/img0034.jpg
https://www.dermis.net/bilder/CD049/550px/img0035.jpg
https://www.dermis.net/bilder/CD049/550px/img0036.jpg
https://www.dermis.net/bilder/CD049/550px/img0037.jpg
https://www.dermis.net/bilder/CD050/550px/img0043.jpg
https://www.dermis.net/bilder/CD050/550px/img0042.jpg
https://www.dermis.net/bilder/CD001/550px/img0067.jpg
https://www.dermis.net/bilder/CD006/550px/img0099.jpg
https://www.dermis.net/bilder/CD036/550px/img0037.jpg
https://www.dermis.net/bilder/CD005/550px/img0080.jpg
https://www.dermis.net/bilder/CD020/550px/img0091.jpg
https://www.dermis.net/bilder/CD002/550px/img0083.jpg
https://www.dermis.net/bilder/CD005/550px/img0016.jpg
https://www.dermis.net/bilder/CD002/550px/img0084.jpg
https://www.dermis.net/bilder/CD048/550px/img0077.jpg
https://www.dermis.net/bilder/CD048/550px/img0078.jpg
https://www.dermis.net/bilder/CD023/550px/img0016.jpg
https://www.dermis.net/bilder/CD036/550px/img0039.jpg

https://www.dermis.net/bilder/CD024/550px/img0013.jpg
https://www.dermis.net/bilder/CD036/550px/img0102.jpg
https://www.dermis.net/bilder/CD034/550px/img0036.jpg
https://www.dermis.net/bilder/CD027/550px/img0095.jpg
https://www.dermis.net/bilder/CD049/550px/img0092.jpg
https://www.dermis.net/bilder/CD015/550px/img0024.jpg
https://www.dermis.net/bilder/CD034/550px/img0002.jpg
https://www.dermis.net/bilder/CD018/550px/img0079.jpg
https://www.dermis.net/bilder/CD018/550px/img0038.jpg
https://www.dermis.net/bilder/CD027/550px/img0096.jpg
https://www.dermis.net/bilder/CD024/550px/img0014.jpg
https://www.dermis.net/bilder/CD026/550px/img0040.jpg
https://www.dermis.net/bilder/CD028/550px/img0047.jpg
https://www.dermis.net/bilder/CD028/550px/img0048.jpg
https://www.dermis.net/bilder/CD028/550px/img0049.jpg
https://www.dermis.net/bilder/CD028/550px/img0050.jpg
https://www.dermis.net/bilder/CD071/550px/img0074.jpg
https://www.dermis.net/bilder/CD034/550px/img0001.jpg
https://www.dermis.net/bilder/CD037/550px/img0026.jpg
https://www.dermis.net/bilder/CD024/550px/img0089.jpg
https://www.dermis.net/bilder/CD026/550px/img0059.jpg
https://www.dermis.net/bilder/CD026/550px/img0058.jpg
https://www.dermis.net/bilder/CD026/550px/img0060.jpg
https://www.dermis.net/bilder/CD050/550px/img0076.jpg
https://www.dermis.net/bilder/CD037/550px/img0027.jpg
https://www.dermis.net/bilder/CD018/550px/img0112.jpg
https://www.dermis.net/bilder/CD037/550px/img0025.jpg
https://www.dermis.net/bilder/CD021/550px/img0093.jpg
https://www.dermis.net/bilder/CD015/550px/img0044.jpg
https://www.dermis.net/bilder/CD016/550px/img0013.jpg
https://www.dermis.net/bilder/CD016/550px/img0047.jpg
https://www.dermis.net/bilder/CD054/550px/img0092.jpg
https://www.dermis.net/bilder/CD005/550px/img0034.jpg
https://www.dermis.net/bilder/CD032/550px/img0092.jpg
https://www.dermis.net/bilder/CD046/550px/img0040.jpg
https://www.dermis.net/bilder/CD032/550px/img0093.jpg
https://www.dermis.net/bilder/CD002/550px/img0011.jpg
https://www.dermis.net/bilder/CD034/550px/img0100.jpg
https://www.dermis.net/bilder/CD046/550px/img0033.jpg
https://www.dermis.net/bilder/CD032/550px/img0100.jpg
https://www.dermis.net/bilder/CD046/550px/img0034.jpg
https://www.dermis.net/bilder/CD015/550px/img0029.jpg
https://www.dermis.net/bilder/CD025/550px/img0077.jpg
https://www.dermis.net/bilder/CD042/550px/img0028.jpg
https://www.dermis.net/bilder/CD026/550px/img0047.jpg
https://www.dermis.net/bilder/CD026/550px/img0048.jpg
https://www.dermis.net/bilder/CD014/550px/img0049.jpg
https://www.dermis.net/bilder/CD047/550px/img0049.jpg

https://www.dermis.net/bilder/CD049/550px/img0084.jpg
https://www.dermis.net/bilder/CD047/550px/img0050.jpg
https://www.dermis.net/bilder/CD047/550px/img0051.jpg
https://www.dermis.net/bilder/CD047/550px/img0052.jpg
https://www.dermis.net/bilder/CD049/550px/img0076.jpg
https://www.dermis.net/bilder/CD047/550px/img0018.jpg
https://www.dermis.net/bilder/CD039/550px/img0082.jpg
https://www.dermis.net/bilder/CD037/550px/img0040.jpg
https://www.dermis.net/bilder/CD026/550px/img0075.jpg
https://www.dermis.net/bilder/CD058/550px/img0063.jpg
https://www.dermis.net/bilder/CD036/550px/img0056.jpg
https://www.dermis.net/bilder/CD058/550px/img0061.jpg
https://www.dermis.net/bilder/CD058/550px/img0062.jpg
https://www.dermis.net/bilder/CD015/550px/img0032.jpg
https://www.dermis.net/bilder/CD040/550px/img0050.jpg
https://www.dermis.net/bilder/CD050/550px/img0101.jpg
https://www.dermis.net/bilder/CD032/550px/img0060.jpg
https://www.dermis.net/bilder/CD050/550px/img0102.jpg
https://www.dermis.net/bilder/CD050/550px/img0100.jpg
https://www.dermis.net/bilder/CD025/550px/img0026.jpg
https://www.dermis.net/bilder/CD025/550px/img0027.jpg
https://www.dermis.net/bilder/CD025/550px/img0028.jpg
https://www.dermis.net/bilder/CD049/550px/img0088.jpg
https://www.dermis.net/bilder/CD027/550px/img0066.jpg
https://www.dermis.net/bilder/CD046/550px/img0095.jpg
https://www.dermis.net/bilder/CD046/550px/img0096.jpg
https://www.dermis.net/bilder/CD054/550px/img0045.jpg
https://www.dermis.net/bilder/CD016/550px/img0016.jpg
https://www.dermis.net/bilder/CD018/550px/img0032.jpg
https://www.dermis.net/bilder/CD020/550px/img0052.jpg
https://www.dermis.net/bilder/CD018/550px/img0090.jpg
https://www.dermis.net/bilder/CD035/550px/img0004.jpg
https://www.dermis.net/bilder/CD035/550px/img0008.jpg
https://www.dermis.net/bilder/CD007/550px/img0029.jpg
https://www.dermis.net/bilder/CD015/550px/img0030.jpg
https://www.dermis.net/bilder/CD006/550px/img0040.jpg
https://www.dermis.net/bilder/CD044/550px/img0012.jpg
https://www.dermis.net/bilder/CD040/550px/img0073.jpg
https://www.dermis.net/bilder/CD035/550px/img0002.jpg
https://www.dermis.net/bilder/CD015/550px/img0036.jpg
https://www.dermis.net/bilder/CD049/550px/img0090.jpg
https://www.dermis.net/bilder/CD049/550px/img0091.jpg
https://www.dermis.net/bilder/CD024/550px/img0021.jpg
https://www.dermis.net/bilder/CD046/550px/img0052.jpg
https://www.dermis.net/bilder/CD023/550px/img0023.jpg
https://www.dermis.net/bilder/CD023/550px/img0024.jpg
https://www.dermis.net/bilder/CD023/550px/img0026.jpg
https://www.dermis.net/bilder/CD023/550px/img0027.jpg

https://www.dermis.net/bilder/CD023/550px/img0028.jpg
https://www.dermis.net/bilder/CD023/550px/img0025.jpg
https://www.dermis.net/bilder/CD023/550px/img0029.jpg
https://www.dermis.net/bilder/CD019/550px/img0009.jpg
https://www.dermis.net/bilder/CD025/550px/img0031.jpg
https://www.dermis.net/bilder/CD015/550px/img0047.jpg
https://www.dermis.net/bilder/CD047/550px/img0009.jpg
https://www.dermis.net/bilder/CD048/550px/img0058.jpg
https://www.dermis.net/bilder/CD048/550px/img0059.jpg
https://www.dermis.net/bilder/CD025/550px/img0030.jpg
https://www.dermis.net/bilder/CD049/550px/img0055.jpg
https://www.dermis.net/bilder/CD049/550px/img0056.jpg
https://www.dermis.net/bilder/CD025/550px/img0033.jpg
https://www.dermis.net/bilder/CD036/550px/img0106.jpg
https://www.dermis.net/bilder/CD015/550px/img0023.jpg
https://www.dermis.net/bilder/CD036/550px/img0019.jpg
https://www.dermis.net/bilder/CD020/550px/img0054.jpg
https://www.dermis.net/bilder/CD020/550px/img0055.jpg
https://www.dermis.net/bilder/CD020/550px/img0058.jpg
https://www.dermis.net/bilder/CD020/550px/img0053.jpg
https://www.dermis.net/bilder/CD047/550px/img0085.jpg
https://www.dermis.net/bilder/CD047/550px/img0086.jpg
https://www.dermis.net/bilder/CD007/550px/img0009.jpg
https://www.dermis.net/bilder/CD046/550px/img0025.jpg
https://www.dermis.net/bilder/CD046/550px/img0026.jpg
https://www.dermis.net/bilder/CD044/550px/img0047.jpg
https://www.dermis.net/bilder/CD048/550px/img0080.jpg
https://www.dermis.net/bilder/CD046/550px/img0027.jpg
https://www.dermis.net/bilder/CD046/550px/img0028.jpg
https://www.dermis.net/bilder/CD036/550px/img0062.jpg
https://www.dermis.net/bilder/CD051/550px/img0105.jpg
https://www.dermis.net/bilder/CD007/550px/img0048.jpg
https://www.dermis.net/bilder/CD044/550px/img0019.jpg
https://www.dermis.net/bilder/CD024/550px/img0048.jpg
https://www.dermis.net/bilder/CD049/550px/img0053.jpg
https://www.dermis.net/bilder/CD047/550px/img0022.jpg
https://www.dermis.net/bilder/CD004/550px/img0014.jpg
https://www.dermis.net/bilder/CD016/550px/img0017.jpg
https://www.dermis.net/bilder/CD002/550px/img0035.jpg
https://www.dermis.net/bilder/CD046/550px/img0077.jpg
https://www.dermis.net/bilder/CD046/550px/img0078.jpg
https://www.dermis.net/bilder/CD007/550px/img0038.jpg
https://www.dermis.net/bilder/CD040/550px/img0082.jpg
https://www.dermis.net/bilder/CD032/550px/img0013.jpg
https://www.dermis.net/bilder/CD032/550px/img0014.jpg
https://www.dermis.net/bilder/CD032/550px/img0018.jpg
https://www.dermis.net/bilder/CD035/550px/img0037.jpg
https://www.dermis.net/bilder/CD035/550px/img0036.jpg

https://www.dermis.net/bilder/CD007/550px/img0037.jpg
https://www.dermis.net/bilder/CD026/550px/img0087.jpg
https://www.dermis.net/bilder/CD039/550px/img0100.jpg
https://www.dermis.net/bilder/CD039/550px/img0054.jpg
https://www.dermis.net/bilder/CD044/550px/img0048.jpg
https://www.dermis.net/bilder/CD016/550px/img0018.jpg
https://www.dermis.net/bilder/CD020/550px/img0019.jpg
https://www.dermis.net/bilder/CD025/550px/img0100.jpg
https://www.dermis.net/bilder/CD020/550px/img0020.jpg
https://www.dermis.net/bilder/CD035/550px/img0021.jpg
https://www.dermis.net/bilder/CD021/550px/img0079.jpg
https://www.dermis.net/bilder/CD024/550px/img0015.jpg
https://www.dermis.net/bilder/CD048/550px/img0101.jpg
https://www.dermis.net/bilder/CD025/550px/img0041.jpg
https://www.dermis.net/bilder/CD032/550px/img0073.jpg
https://www.dermis.net/bilder/CD032/550px/img0074.jpg
https://www.dermis.net/bilder/CD032/550px/img0091.jpg
https://www.dermis.net/bilder/CD046/550px/img0029.jpg
https://www.dermis.net/bilder/CD046/550px/img0036.jpg
https://www.dermis.net/bilder/CD049/550px/img0074.jpg
https://www.dermis.net/bilder/CD033/550px/img0081.jpg
https://www.dermis.net/bilder/CD026/550px/img0019.jpg
https://www.dermis.net/bilder/CD014/550px/img0013.jpg
https://www.dermis.net/bilder/CD023/550px/img0042.jpg
https://www.dermis.net/bilder/CD023/550px/img0043.jpg
https://www.dermis.net/bilder/CD026/550px/img0018.jpg
https://www.dermis.net/bilder/CD027/550px/img0008.jpg
https://www.dermis.net/bilder/CD046/550px/img0069.jpg
https://www.dermis.net/bilder/CD002/550px/img0037.jpg
https://www.dermis.net/bilder/CD046/550px/img0070.jpg
https://www.dermis.net/bilder/CD023/550px/img0041.jpg
https://www.dermis.net/bilder/CD046/550px/img0098.jpg
https://www.dermis.net/bilder/CD058/550px/img0053.jpg
https://www.dermis.net/bilder/CD058/550px/img0059.jpg
https://www.dermis.net/bilder/CD015/550px/img0008.jpg
https://www.dermis.net/bilder/CD003/550px/img0012.jpg
https://www.dermis.net/bilder/CD049/550px/img0078.jpg
https://www.dermis.net/bilder/CD023/550px/img0018.jpg
https://www.dermis.net/bilder/CD044/550px/img0053.jpg
https://www.dermis.net/bilder/CD037/550px/img0021.jpg
https://www.dermis.net/bilder/CD024/550px/img0010.jpg
https://www.dermis.net/bilder/CD036/550px/img0012.jpg
https://www.dermis.net/bilder/CD006/550px/img0064.jpg
https://www.dermis.net/bilder/CD047/550px/img0008.jpg
https://www.dermis.net/bilder/CD023/550px/img0014.jpg
https://www.dermis.net/bilder/CD023/550px/img0015.jpg
https://www.dermis.net/bilder/CD048/550px/img0092.jpg
https://www.dermis.net/bilder/CD018/550px/img0034.jpg

https://www.dermis.net/bilder/CD018/550px/img0029.jpg
https://www.dermis.net/bilder/CD023/550px/img0077.jpg
https://www.dermis.net/bilder/CD071/550px/img0065.jpg
https://www.dermis.net/bilder/CD023/550px/img0078.jpg
https://www.dermis.net/bilder/CD023/550px/img0079.jpg
https://www.dermis.net/bilder/CD035/550px/img0062.jpg
https://www.dermis.net/bilder/CD035/550px/img0063.jpg
https://www.dermis.net/bilder/CD007/550px/img0011.jpg
https://www.dermis.net/bilder/CD047/550px/img0090.jpg
https://www.dermis.net/bilder/CD052/550px/img0030.jpg
https://www.dermis.net/bilder/CD052/550px/img0062.jpg
https://www.dermis.net/bilder/CD040/550px/img0021.jpg
https://www.dermis.net/bilder/CD036/550px/img0083.jpg
https://www.dermis.net/bilder/CD018/550px/img0030.jpg
https://www.dermis.net/bilder/CD002/550px/img0032.jpg
https://www.dermis.net/bilder/CD046/550px/img0021.jpg
https://www.dermis.net/bilder/CD032/550px/img0085.jpg
https://www.dermis.net/bilder/CD032/550px/img0087.jpg
https://www.dermis.net/bilder/CD032/550px/img0076.jpg
https://www.dermis.net/bilder/CD024/550px/img0003.jpg
https://www.dermis.net/bilder/CD025/550px/img0093.jpg
https://www.dermis.net/bilder/CD039/550px/img0003.jpg
https://www.dermis.net/bilder/CD032/550px/img0025.jpg
https://www.dermis.net/bilder/CD032/550px/img0029.jpg
https://www.dermis.net/bilder/CD032/550px/img0030.jpg
https://www.dermis.net/bilder/CD057/550px/img0040.jpg
https://www.dermis.net/bilder/CD054/550px/img0046.jpg
https://www.dermis.net/bilder/CD032/550px/img0024.jpg
https://www.dermis.net/bilder/CD027/550px/img0084.jpg
https://www.dermis.net/bilder/CD023/550px/img0074.jpg
https://www.dermis.net/bilder/CD027/550px/img0004.jpg
https://www.dermis.net/bilder/CD048/550px/img0097.jpg
https://www.dermis.net/bilder/CD037/550px/img0020.jpg
https://www.dermis.net/bilder/CD057/550px/img0001.jpg
https://www.dermis.net/bilder/CD023/550px/img0094.jpg
https://www.dermis.net/bilder/CD023/550px/img0095.jpg
https://www.dermis.net/bilder/CD040/550px/img0091.jpg
https://www.dermis.net/bilder/CD047/550px/img0023.jpg
https://www.dermis.net/bilder/CD047/550px/img0024.jpg
https://www.dermis.net/bilder/CD047/550px/img0011.jpg
https://www.dermis.net/bilder/CD014/550px/img0032.jpg
https://www.dermis.net/bilder/CD042/550px/img0017.jpg
https://www.dermis.net/bilder/CD042/550px/img0018.jpg
https://www.dermis.net/bilder/CD006/550px/img0043.jpg
https://www.dermis.net/bilder/CD039/550px/img0027.jpg
https://www.dermis.net/bilder/CD032/550px/img0057.jpg
https://www.dermis.net/bilder/CD032/550px/img0077.jpg
https://www.dermis.net/bilder/CD020/550px/img0013.jpg

https://www.dermis.net/bilder/CD002/550px/img0063.jpg
https://www.dermis.net/bilder/CD040/550px/img0101.jpg
https://www.dermis.net/bilder/CD040/550px/img0102.jpg
https://www.dermis.net/bilder/CD020/550px/img0012.jpg
https://www.dermis.net/bilder/CD020/550px/img0014.jpg
https://www.dermis.net/bilder/CD020/550px/img0015.jpg
https://www.dermis.net/bilder/CD033/550px/img0089.jpg
https://www.dermis.net/bilder/CD025/550px/img0075.jpg
https://www.dermis.net/bilder/CD039/550px/img0075.jpg
https://www.dermis.net/bilder/CD039/550px/img0074.jpg
https://www.dermis.net/bilder/CD039/550px/img0076.jpg
https://www.dermis.net/bilder/CD039/550px/img0077.jpg
https://www.dermis.net/bilder/CD023/550px/img0090.jpg
https://www.dermis.net/bilder/CD049/550px/img0089.jpg
https://www.dermis.net/bilder/CD018/550px/img0082.jpg
https://www.dermis.net/bilder/CD027/550px/img0100.jpg
https://www.dermis.net/bilder/CD034/550px/img0082.jpg
https://www.dermis.net/bilder/CD039/550px/img0017.jpg
https://www.dermis.net/bilder/CD047/550px/img0012.jpg
https://www.dermis.net/bilder/CD047/550px/img0013.jpg
https://www.dermis.net/bilder/CD032/550px/img0079.jpg
https://www.dermis.net/bilder/CD032/550px/img0080.jpg
https://www.dermis.net/bilder/CD057/550px/img0032.jpg
https://www.dermis.net/bilder/CD057/550px/img0019.jpg
https://www.dermis.net/bilder/CD044/550px/img0018.jpg
https://www.dermis.net/bilder/CD004/550px/img0009.jpg
https://www.dermis.net/bilder/CD039/550px/img0047.jpg
https://www.dermis.net/bilder/CD033/550px/img0074.jpg
https://www.dermis.net/bilder/CD039/550px/img0048.jpg
https://www.dermis.net/bilder/CD047/550px/img0062.jpg
https://www.dermis.net/bilder/CD047/550px/img0063.jpg
https://www.dermis.net/bilder/CD050/550px/img0045.jpg
https://www.dermis.net/bilder/CD058/550px/img0097.jpg
https://www.dermis.net/bilder/CD044/550px/img0033.jpg
https://www.dermis.net/bilder/CD002/550px/img0051.jpg
https://www.dermis.net/bilder/CD046/550px/img0097.jpg
https://www.dermis.net/bilder/CD047/550px/img0072.jpg
https://www.dermis.net/bilder/CD032/550px/img0097.jpg
https://www.dermis.net/bilder/CD044/550px/img0070.jpg
https://www.dermis.net/bilder/CD044/550px/img0071.jpg
https://www.dermis.net/bilder/CD035/550px/img0042.jpg
https://www.dermis.net/bilder/CD035/550px/img0043.jpg
https://www.dermis.net/bilder/CD048/550px/img0075.jpg
https://www.dermis.net/bilder/CD035/550px/img0044.jpg
https://www.dermis.net/bilder/CD035/550px/img0045.jpg
https://www.dermis.net/bilder/CD014/550px/img0083.jpg
https://www.dermis.net/bilder/CD008/550px/img0045.jpg
https://www.dermis.net/bilder/CD005/550px/img0051.jpg

https://www.dermis.net/bilder/CD026/550px/img0041.jpg
https://www.dermis.net/bilder/CD046/550px/img0030.jpg
https://www.dermis.net/bilder/CD046/550px/img0031.jpg
https://www.dermis.net/bilder/CD016/550px/img0004.jpg
https://www.dermis.net/bilder/CD005/550px/img0091.jpg
https://www.dermis.net/bilder/CD039/550px/img0063.jpg
https://www.dermis.net/bilder/CD039/550px/img0064.jpg
https://www.dermis.net/bilder/CD039/550px/img0065.jpg
https://www.dermis.net/bilder/CD018/550px/img0031.jpg
https://www.dermis.net/bilder/CD039/550px/img0061.jpg
https://www.dermis.net/bilder/CD039/550px/img0062.jpg
https://www.dermis.net/bilder/CD052/550px/img0011.jpg
https://www.dermis.net/bilder/CD018/550px/img0049.jpg
https://www.dermis.net/bilder/CD006/550px/img0067.jpg
https://www.dermis.net/bilder/CD034/550px/img0071.jpg
https://www.dermis.net/bilder/CD046/550px/img0089.jpg
https://www.dermis.net/bilder/CD046/550px/img0090.jpg
https://www.dermis.net/bilder/CD057/550px/img0017.jpg
https://www.dermis.net/bilder/CD026/550px/img0028.jpg
https://www.dermis.net/bilder/CD026/550px/img0029.jpg
https://www.dermis.net/bilder/CD005/550px/img0057.jpg
https://www.dermis.net/bilder/CD020/550px/img0032.jpg
https://www.dermis.net/bilder/CD020/550px/img0033.jpg
https://www.dermis.net/bilder/CD023/550px/img0051.jpg
https://www.dermis.net/bilder/CD023/550px/img0052.jpg
https://www.dermis.net/bilder/CD033/550px/img0085.jpg
https://www.dermis.net/bilder/CD025/550px/img0001.jpg
https://www.dermis.net/bilder/CD023/550px/img0050.jpg
https://www.dermis.net/bilder/CD033/550px/img0086.jpg
https://www.dermis.net/bilder/CD037/550px/img0009.jpg
https://www.dermis.net/bilder/CD007/550px/img0008.jpg
https://www.dermis.net/bilder/CD042/550px/img0092.jpg
https://www.dermis.net/bilder/CD037/550px/img0010.jpg
https://www.dermis.net/bilder/CD039/550px/img0030.jpg
https://www.dermis.net/bilder/CD033/550px/img0091.jpg
https://www.dermis.net/bilder/CD033/550px/img0092.jpg
https://www.dermis.net/bilder/CD034/550px/img0079.jpg
https://www.dermis.net/bilder/CD034/550px/img0080.jpg
https://www.dermis.net/bilder/CD052/550px/img0063.jpg
https://www.dermis.net/bilder/CD057/550px/img0041.jpg
https://www.dermis.net/bilder/CD046/550px/img0087.jpg
https://www.dermis.net/bilder/CD049/550px/img0069.jpg
https://www.dermis.net/bilder/CD049/550px/img0070.jpg
https://www.dermis.net/bilder/CD046/550px/img0088.jpg
https://www.dermis.net/bilder/CD046/550px/img0056.jpg
https://www.dermis.net/bilder/CD005/550px/img0052.jpg
https://www.dermis.net/bilder/CD035/550px/img0079.jpg
https://www.dermis.net/bilder/CD036/550px/img0087.jpg

https://www.dermis.net/bilder/CD212/550px/img0026.jpg
https://www.dermis.net/bilder/CD212/550px/img0027.jpg
https://www.dermis.net/bilder/CD042/550px/img0087.jpg
https://www.dermis.net/bilder/CD212/550px/img0021.jpg
https://www.dermis.net/bilder/CD212/550px/img0022.jpg
https://www.dermis.net/bilder/CD212/550px/img0018.jpg
https://www.dermis.net/bilder/CD183/550px/img0044.jpg
https://www.dermis.net/bilder/CD193/550px/img0020.jpg
https://www.dermis.net/bilder/CD194/550px/img0102.jpg
https://www.dermis.net/bilder/CD212/550px/img0003.jpg
https://www.dermis.net/bilder/CD196/550px/img0035.jpg
https://www.dermis.net/bilder/CD196/550px/img0037.jpg
https://www.dermis.net/bilder/CD018/550px/img0023.jpg
https://www.dermis.net/bilder/CD055/550px/img0069.jpg
https://www.dermis.net/bilder/CD198/550px/img0112.jpg
https://www.dermis.net/bilder/CD001/550px/img0081.jpg
https://www.dermis.net/bilder/CD001/550px/img0082.jpg
https://www.dermis.net/bilder/CD189/550px/img0100.jpg
https://www.dermis.net/bilder/CD185/550px/img0016.jpg
https://www.dermis.net/bilder/CD194/550px/img0103.jpg
https://www.dermis.net/bilder/CD187/550px/img0016.jpg
https://www.dermis.net/bilder/CD002/550px/img0013.jpg
https://www.dermis.net/bilder/CD002/550px/img0038.jpg
https://www.dermis.net/bilder/CD193/550px/img0018.jpg
https://www.dermis.net/bilder/CD193/550px/img0019.jpg
https://www.dermis.net/bilder/CD001/550px/img0044.jpg
https://www.dermis.net/bilder/CD044/550px/img0041.jpg
https://www.dermis.net/bilder/CD035/550px/img0087.jpg
https://www.dermis.net/bilder/CD037/550px/img0029.jpg
https://www.dermis.net/bilder/CD035/550px/img0065.jpg
https://www.dermis.net/bilder/CD035/550px/img0066.jpg
https://www.dermis.net/bilder/CD183/550px/img0085.jpg
https://www.dermis.net/bilder/CD042/550px/img0054.jpg
https://www.dermis.net/bilder/CD199/550px/img0036.jpg
https://www.dermis.net/bilder/CD189/550px/img0045.jpg
https://www.dermis.net/bilder/CD178/550px/img0040.jpg
https://www.dermis.net/bilder/CD178/550px/img0041.jpg
https://www.dermis.net/bilder/CD188/550px/img0001.jpg
https://www.dermis.net/bilder/CD188/550px/img0002.jpg
https://www.dermis.net/bilder/CD188/550px/img0003.jpg
https://www.dermis.net/bilder/CD188/550px/img0004.jpg
https://www.dermis.net/bilder/CD188/550px/img0005.jpg
https://www.dermis.net/bilder/CD187/550px/img0075.jpg
https://www.dermis.net/bilder/CD199/550px/img0029.jpg
https://www.dermis.net/bilder/CD199/550px/img0128.jpg
https://www.dermis.net/bilder/CD193/550px/img0096.jpg
https://www.dermis.net/bilder/CD191/550px/img0043.jpg
https://www.dermis.net/bilder/CD029/550px/img0015.jpg

https://www.dermis.net/bilder/CD180/550px/img0038.jpg
https://www.dermis.net/bilder/CD041/550px/img0063.jpg
https://www.dermis.net/bilder/CD002/550px/img0046.jpg
https://www.dermis.net/bilder/CD002/550px/img0007.jpg
https://www.dermis.net/bilder/CD002/550px/img0041.jpg
https://www.dermis.net/bilder/CD002/550px/img0016.jpg
https://www.dermis.net/bilder/CD002/550px/img0002.jpg
https://www.dermis.net/bilder/CD002/550px/img0028.jpg
https://www.dermis.net/bilder/CD002/550px/img0043.jpg
https://www.dermis.net/bilder/CD027/550px/img0032.jpg
https://www.dermis.net/bilder/CD195/550px/img0031.jpg
https://www.dermis.net/bilder/CD212/550px/img0004.jpg
https://www.dermis.net/bilder/CD195/550px/img0115.jpg
https://www.dermis.net/bilder/CD195/550px/img0116.jpg
https://www.dermis.net/bilder/CD198/550px/img0051.jpg
https://www.dermis.net/bilder/CD179/550px/img0031.jpg
https://www.dermis.net/bilder/CD183/550px/img0084.jpg
https://www.dermis.net/bilder/CD193/550px/img0183.jpg
https://www.dermis.net/bilder/CD193/550px/img0184.jpg
https://www.dermis.net/bilder/CD196/550px/img0049.jpg
https://www.dermis.net/bilder/CD196/550px/img0050.jpg
https://www.dermis.net/bilder/CD197/550px/img0053.jpg
https://www.dermis.net/bilder/CD212/550px/img0028.jpg
https://www.dermis.net/bilder/CD192/550px/img0064.jpg
https://www.dermis.net/bilder/CD192/550px/img0065.jpg
https://www.dermis.net/bilder/CD058/550px/img0093.jpg
https://www.dermis.net/bilder/CD006/550px/img0052.jpg
https://www.dermis.net/bilder/CD057/550px/img0059.jpg
https://www.dermis.net/bilder/CD189/550px/img0044.jpg
https://www.dermis.net/bilder/CD178/550px/img0038.jpg
https://www.dermis.net/bilder/CD178/550px/img0039.jpg
https://www.dermis.net/bilder/CD183/550px/img0058.jpg
https://www.dermis.net/bilder/CD018/550px/img0067.jpg
https://www.dermis.net/bilder/CD190/550px/img0063.jpg
https://www.dermis.net/bilder/CD190/550px/img0065.jpg
https://www.dermis.net/bilder/CD198/550px/img0087.jpg
https://www.dermis.net/bilder/CD033/550px/img0011.jpg
https://www.dermis.net/bilder/CD212/550px/img0078.jpg
https://www.dermis.net/bilder/CD212/550px/img0079.jpg
https://www.dermis.net/bilder/CD012/550px/img0086.jpg
https://www.dermis.net/bilder/CD211/550px/img0010.jpg
https://www.dermis.net/bilder/CD210/550px/img0029.jpg
https://www.dermis.net/bilder/CD025/550px/img0067.jpg
https://www.dermis.net/bilder/CD183/550px/img0041.jpg
https://www.dermis.net/bilder/CD183/550px/img0042.jpg
https://www.dermis.net/bilder/CD183/550px/img0043.jpg
https://www.dermis.net/bilder/CD193/550px/img0109.jpg
https://www.dermis.net/bilder/CD193/550px/img0110.jpg

https://www.dermis.net/bilder/CD016/550px/img0005.jpg
https://www.dermis.net/bilder/CD042/550px/img0048.jpg
https://www.dermis.net/bilder/CD042/550px/img0050.jpg
https://www.dermis.net/bilder/CD035/550px/img0085.jpg
https://www.dermis.net/bilder/CD181/550px/img0026.jpg
https://www.dermis.net/bilder/CD181/550px/img0025.jpg
https://www.dermis.net/bilder/CD191/550px/img0058.jpg
https://www.dermis.net/bilder/CD191/550px/img0059.jpg
https://www.dermis.net/bilder/CD013/550px/img0029.jpg
https://www.dermis.net/bilder/CD212/550px/img0085.jpg
https://www.dermis.net/bilder/CD035/550px/img0067.jpg
https://www.dermis.net/bilder/CD070/550px/img0085.jpg
https://www.dermis.net/bilder/CD036/550px/img0015.jpg
https://www.dermis.net/bilder/CD194/550px/img0110.jpg
https://www.dermis.net/bilder/CD197/550px/img0045.jpg
https://www.dermis.net/bilder/CD212/550px/img0048.jpg
https://www.dermis.net/bilder/CD212/550px/img0050.jpg
https://www.dermis.net/bilder/CD187/550px/img0074.jpg
https://www.dermis.net/bilder/CD055/550px/img0060.jpg
https://www.dermis.net/bilder/CD055/550px/img0061.jpg
https://www.dermis.net/bilder/CD192/550px/img0048.jpg
https://www.dermis.net/bilder/CD192/550px/img0049.jpg
https://www.dermis.net/bilder/CD193/550px/img0185.jpg
https://www.dermis.net/bilder/CD198/550px/img0122.jpg
https://www.dermis.net/bilder/CD210/550px/img0090.jpg
https://www.dermis.net/bilder/CD210/550px/img0092.jpg
https://www.dermis.net/bilder/CD210/550px/img0093.jpg
https://www.dermis.net/bilder/CD008/550px/img0012.jpg
https://www.dermis.net/bilder/CD193/550px/img0078.jpg
https://www.dermis.net/bilder/CD189/550px/img0051.jpg
https://www.dermis.net/bilder/CD189/550px/img0052.jpg
https://www.dermis.net/bilder/CD193/550px/img0143.jpg
https://www.dermis.net/bilder/CD193/550px/img0144.jpg
https://www.dermis.net/bilder/CD042/550px/img0011.jpg
https://www.dermis.net/bilder/CD042/550px/img0012.jpg
https://www.dermis.net/bilder/CD199/550px/img0113.jpg
https://www.dermis.net/bilder/CD002/550px/img0070.jpg
https://www.dermis.net/bilder/CD212/550px/img0104.jpg
https://www.dermis.net/bilder/CD196/550px/img0138.jpg
https://www.dermis.net/bilder/CD041/550px/img0058.jpg
https://www.dermis.net/bilder/CD189/550px/img0096.jpg
https://www.dermis.net/bilder/CD184/550px/img0055.jpg
https://www.dermis.net/bilder/CD189/550px/img0043.jpg
https://www.dermis.net/bilder/CD201/550px/img0031.jpg
https://www.dermis.net/bilder/CD183/550px/img0065.jpg
https://www.dermis.net/bilder/CD193/550px/img0157.jpg
https://www.dermis.net/bilder/CD190/550px/img0012.jpg
https://www.dermis.net/bilder/CD190/550px/img0013.jpg

https://www.dermis.net/bilder/CD011/550px/img0096.jpg
https://www.dermis.net/bilder/CD042/550px/img0030.jpg
https://www.dermis.net/bilder/CD042/550px/img0013.jpg
https://www.dermis.net/bilder/CD196/550px/img0005.jpg
https://www.dermis.net/bilder/CD194/550px/img0105.jpg
https://www.dermis.net/bilder/CD055/550px/img0042.jpg
https://www.dermis.net/bilder/CD057/550px/img0066.jpg
https://www.dermis.net/bilder/CD040/550px/img0084.jpg
https://www.dermis.net/bilder/CD181/550px/img0027.jpg
https://www.dermis.net/bilder/CD055/550px/img0059.jpg
https://www.dermis.net/bilder/CD184/550px/img0083.jpg
https://www.dermis.net/bilder/CD183/550px/img0018.jpg
https://www.dermis.net/bilder/CD025/550px/img0013.jpg
https://www.dermis.net/bilder/CD033/550px/img0006.jpg
https://www.dermis.net/bilder/CD195/550px/img0064.jpg
https://www.dermis.net/bilder/CD195/550px/img0065.jpg
https://www.dermis.net/bilder/CD210/550px/img0048.jpg
https://www.dermis.net/bilder/CD193/550px/img0098.jpg
https://www.dermis.net/bilder/CD196/550px/img0120.jpg
https://www.dermis.net/bilder/CD042/550px/img0069.jpg
https://www.dermis.net/bilder/CD198/550px/img0001.jpg
https://www.dermis.net/bilder/CD180/550px/img0052.jpg
https://www.dermis.net/bilder/CD194/550px/img0162.jpg
https://www.dermis.net/bilder/CD027/550px/img0021.jpg
https://www.dermis.net/bilder/CD001/550px/img0056.jpg
https://www.dermis.net/bilder/CD178/550px/img0001.jpg
https://www.dermis.net/bilder/CD184/550px/img0042.jpg
https://www.dermis.net/bilder/CD194/550px/img0020.jpg
https://www.dermis.net/bilder/CD194/550px/img0021.jpg
https://www.dermis.net/bilder/CD194/550px/img0022.jpg
https://www.dermis.net/bilder/CD035/550px/img0093.jpg
https://www.dermis.net/bilder/CD056/550px/img0077.jpg
https://www.dermis.net/bilder/CD192/550px/img0039.jpg
https://www.dermis.net/bilder/CD055/550px/img0082.jpg
https://www.dermis.net/bilder/CD198/550px/img0145.jpg
https://www.dermis.net/bilder/CD198/550px/img0146.jpg
https://www.dermis.net/bilder/CD041/550px/img0066.jpg
https://www.dermis.net/bilder/CD008/550px/img0073.jpg
https://www.dermis.net/bilder/CD013/550px/img0045.jpg
https://www.dermis.net/bilder/CD042/550px/img0052.jpg
https://www.dermis.net/bilder/CD070/550px/img0011.jpg
https://www.dermis.net/bilder/CD070/550px/img0010.jpg
https://www.dermis.net/bilder/CD070/550px/img0002.jpg
https://www.dermis.net/bilder/CD194/550px/img0027.jpg
https://www.dermis.net/bilder/CD042/550px/img0016.jpg
https://www.dermis.net/bilder/CD001/550px/img0053.jpg
https://www.dermis.net/bilder/CD006/550px/img0077.jpg
https://www.dermis.net/bilder/CD182/550px/img0079.jpg

https://www.dermis.net/bilder/CD185/550px/img0024.jpg
https://www.dermis.net/bilder/CD015/550px/img0001.jpg
https://www.dermis.net/bilder/CD008/550px/img0020.jpg
https://www.dermis.net/bilder/CD189/550px/img0041.jpg
https://www.dermis.net/bilder/CD189/550px/img0042.jpg
https://www.dermis.net/bilder/CD178/550px/img0037.jpg
https://www.dermis.net/bilder/CD183/550px/img0045.jpg
https://www.dermis.net/bilder/CD184/550px/img0081.jpg
https://www.dermis.net/bilder/CD184/550px/img0082.jpg
https://www.dermis.net/bilder/CD184/550px/img0061.jpg
https://www.dermis.net/bilder/CD183/550px/img0056.jpg
https://www.dermis.net/bilder/CD183/550px/img0057.jpg
https://www.dermis.net/bilder/CD183/550px/img0060.jpg
https://www.dermis.net/bilder/CD183/550px/img0061.jpg
https://www.dermis.net/bilder/CD195/550px/img0028.jpg
https://www.dermis.net/bilder/CD186/550px/img0006.jpg
https://www.dermis.net/bilder/CD070/550px/img0083.jpg
https://www.dermis.net/bilder/CD190/550px/img0062.jpg
https://www.dermis.net/bilder/CD198/550px/img0086.jpg
https://www.dermis.net/bilder/CD013/550px/img0031.jpg
https://www.dermis.net/bilder/CD193/550px/img0013.jpg
https://www.dermis.net/bilder/CD185/550px/img0044.jpg
https://www.dermis.net/bilder/CD185/550px/img0045.jpg
https://www.dermis.net/bilder/CD193/550px/img0033.jpg
https://www.dermis.net/bilder/CD193/550px/img0034.jpg
https://www.dermis.net/bilder/CD212/550px/img0100.jpg
https://www.dermis.net/bilder/CD085/550px/img0072.jpg
https://www.dermis.net/bilder/CD054/550px/img0018.jpg
https://www.dermis.net/bilder/CD183/550px/img0062.jpg
https://www.dermis.net/bilder/CD041/550px/img0087.jpg
https://www.dermis.net/bilder/CD056/550px/img0060.jpg
https://www.dermis.net/bilder/CD183/550px/img0040.jpg
https://www.dermis.net/bilder/CD036/550px/img0011.jpg
https://www.dermis.net/bilder/CD193/550px/img0101.jpg
https://www.dermis.net/bilder/CD193/550px/img0155.jpg
https://www.dermis.net/bilder/CD196/550px/img0027.jpg
https://www.dermis.net/bilder/CD196/550px/img0145.jpg
https://www.dermis.net/bilder/CD196/550px/img0146.jpg
https://www.dermis.net/bilder/CD196/550px/img0147.jpg
https://www.dermis.net/bilder/CD196/550px/img0148.jpg
https://www.dermis.net/bilder/CD008/550px/img0002.jpg
https://www.dermis.net/bilder/CD185/550px/img0080.jpg
https://www.dermis.net/bilder/CD193/550px/img0070.jpg
https://www.dermis.net/bilder/CD193/550px/img0121.jpg
https://www.dermis.net/bilder/CD193/550px/img0122.jpg
https://www.dermis.net/bilder/CD198/550px/img0116.jpg
https://www.dermis.net/bilder/CD198/550px/img0117.jpg
https://www.dermis.net/bilder/CD055/550px/img0044.jpg

https://www.dermis.net/bilder/CD178/550px/img0090.jpg
https://www.dermis.net/bilder/CD178/550px/img0091.jpg
https://www.dermis.net/bilder/CD184/550px/img0015.jpg
https://www.dermis.net/bilder/CD184/550px/img0017.jpg
https://www.dermis.net/bilder/CD013/550px/img0009.jpg
https://www.dermis.net/bilder/CD007/550px/img0100.jpg
https://www.dermis.net/bilder/CD196/550px/img0115.jpg
https://www.dermis.net/bilder/CD196/550px/img0116.jpg
https://www.dermis.net/bilder/CD042/550px/img0034.jpg
https://www.dermis.net/bilder/CD194/550px/img0109.jpg
https://www.dermis.net/bilder/CD198/550px/img0113.jpg
https://www.dermis.net/bilder/CD191/550px/img0032.jpg
https://www.dermis.net/bilder/CD192/550px/img0087.jpg
https://www.dermis.net/bilder/CD192/550px/img0086.jpg
https://www.dermis.net/bilder/CD189/550px/img0050.jpg
https://www.dermis.net/bilder/CD193/550px/img0142.jpg
https://www.dermis.net/bilder/CD007/550px/img0118.jpg
https://www.dermis.net/bilder/CD210/550px/img0050.jpg
https://www.dermis.net/bilder/CD002/550px/img0058.jpg
https://www.dermis.net/bilder/CD005/550px/img0064.jpg
https://www.dermis.net/bilder/CD005/550px/img0076.jpg
https://www.dermis.net/bilder/CD002/550px/img0059.jpg
https://www.dermis.net/bilder/CD001/550px/img0023.jpg
https://www.dermis.net/bilder/CD001/550px/img0021.jpg
https://www.dermis.net/bilder/CD001/550px/img0022.jpg
https://www.dermis.net/bilder/CD016/550px/img0032.jpg
https://www.dermis.net/bilder/CD021/550px/img0097.jpg
https://www.dermis.net/bilder/CD002/550px/img0034.jpg
https://www.dermis.net/bilder/CD190/550px/img0049.jpg
https://www.dermis.net/bilder/CD070/550px/img0012.jpg
https://www.dermis.net/bilder/CD035/550px/img0088.jpg
https://www.dermis.net/bilder/CD194/550px/img0083.jpg
https://www.dermis.net/bilder/CD194/550px/img0084.jpg
https://www.dermis.net/bilder/CD194/550px/img0085.jpg
https://www.dermis.net/bilder/CD180/550px/img0003.jpg
https://www.dermis.net/bilder/CD185/550px/img0003.jpg
https://www.dermis.net/bilder/CD055/550px/img0065.jpg
https://www.dermis.net/bilder/CD178/550px/img0083.jpg
https://www.dermis.net/bilder/CD189/550px/img0026.jpg
https://www.dermis.net/bilder/CD189/550px/img0027.jpg
https://www.dermis.net/bilder/CD189/550px/img0028.jpg
https://www.dermis.net/bilder/CD187/550px/img0072.jpg
https://www.dermis.net/bilder/CD199/550px/img0123.jpg
https://www.dermis.net/bilder/CD212/550px/img0064.jpg
https://www.dermis.net/bilder/CD013/550px/img0033.jpg
https://www.dermis.net/bilder/CD210/550px/img0057.jpg
https://www.dermis.net/bilder/CD199/550px/img0075.jpg
https://www.dermis.net/bilder/CD212/550px/img0087.jpg

https://www.dermis.net/bilder/CD044/550px/img0034.jpg
https://www.dermis.net/bilder/CD190/550px/img0041.jpg
https://www.dermis.net/bilder/CD198/550px/img0055.jpg
https://www.dermis.net/bilder/CD212/550px/img0029.jpg
https://www.dermis.net/bilder/CD191/550px/img0050.jpg
https://www.dermis.net/bilder/CD199/550px/img0120.jpg
https://www.dermis.net/bilder/CD212/550px/img0016.jpg
https://www.dermis.net/bilder/CD212/550px/img0017.jpg
https://www.dermis.net/bilder/CD194/550px/img0134.jpg
https://www.dermis.net/bilder/CD055/550px/img0056.jpg
https://www.dermis.net/bilder/CD055/550px/img0057.jpg
https://www.dermis.net/bilder/CD196/550px/img0006.jpg
https://www.dermis.net/bilder/CD196/550px/img0009.jpg
https://www.dermis.net/bilder/CD196/550px/img0010.jpg
https://www.dermis.net/bilder/CD012/550px/img0008.jpg
https://www.dermis.net/bilder/CD012/550px/img0009.jpg
https://www.dermis.net/bilder/CD035/550px/img0081.jpg
https://www.dermis.net/bilder/CD026/550px/img0007.jpg
https://www.dermis.net/bilder/CD026/550px/img0015.jpg
https://www.dermis.net/bilder/CD199/550px/img0031.jpg
https://www.dermis.net/bilder/CD199/550px/img0032.jpg
https://www.dermis.net/bilder/CD189/550px/img0040.jpg
https://www.dermis.net/bilder/CD184/550px/img0041.jpg
https://www.dermis.net/bilder/CD002/550px/img0082.jpg
https://www.dermis.net/bilder/CD004/550px/img0013.jpg
https://www.dermis.net/bilder/CD182/550px/img0009.jpg
https://www.dermis.net/bilder/CD182/550px/img0011.jpg
https://www.dermis.net/bilder/CD193/550px/img0136.jpg
https://www.dermis.net/bilder/CD182/550px/img0008.jpg
https://www.dermis.net/bilder/CD182/550px/img0007.jpg
https://www.dermis.net/bilder/CD187/550px/img0024.jpg
https://www.dermis.net/bilder/CD197/550px/img0063.jpg
https://www.dermis.net/bilder/CD197/550px/img0064.jpg
https://www.dermis.net/bilder/CD197/550px/img0047.jpg
https://www.dermis.net/bilder/CD198/550px/img0137.jpg
https://www.dermis.net/bilder/CD042/550px/img0029.jpg
https://www.dermis.net/bilder/CD210/550px/img0049.jpg
https://www.dermis.net/bilder/CD013/550px/img0026.jpg
https://www.dermis.net/bilder/CD001/550px/img0095.jpg
https://www.dermis.net/bilder/CD032/550px/img0089.jpg
https://www.dermis.net/bilder/CD041/550px/img0077.jpg
https://www.dermis.net/bilder/CD012/550px/img0076.jpg
https://www.dermis.net/bilder/CD178/550px/img0101.jpg
https://www.dermis.net/bilder/CD197/550px/img0046.jpg
https://www.dermis.net/bilder/CD011/550px/img0034.jpg
https://www.dermis.net/bilder/CD011/550px/img0044.jpg
https://www.dermis.net/bilder/CD011/550px/img0046.jpg
https://www.dermis.net/bilder/CD011/550px/img0047.jpg

https://www.dermis.net/bilder/CD025/550px/img0014.jpg
https://www.dermis.net/bilder/CD193/550px/img0057.jpg
https://www.dermis.net/bilder/CD002/550px/img0025.jpg
https://www.dermis.net/bilder/CD008/550px/img0078.jpg
https://www.dermis.net/bilder/CD037/550px/img0048.jpg
https://www.dermis.net/bilder/CD194/550px/img0086.jpg
https://www.dermis.net/bilder/CD196/550px/img0026.jpg
https://www.dermis.net/bilder/CD002/550px/img0081.jpg
https://www.dermis.net/bilder/CD181/550px/img0013.jpg
https://www.dermis.net/bilder/CD033/550px/img0019.jpg
https://www.dermis.net/bilder/CD002/550px/img0024.jpg
https://www.dermis.net/bilder/CD033/550px/img0025.jpg
https://www.dermis.net/bilder/CD033/550px/img0030.jpg
https://www.dermis.net/bilder/CD055/550px/img0052.jpg
https://www.dermis.net/bilder/CD041/550px/img0011.jpg
https://www.dermis.net/bilder/CD027/550px/img0016.jpg
https://www.dermis.net/bilder/CD036/550px/img0084.jpg
https://www.dermis.net/bilder/CD198/550px/img0124.jpg
https://www.dermis.net/bilder/CD199/550px/img0034.jpg
https://www.dermis.net/bilder/CD054/550px/img0009.jpg
https://www.dermis.net/bilder/CD054/550px/img0010.jpg
https://www.dermis.net/bilder/CD016/550px/img0015.jpg
https://www.dermis.net/bilder/CD012/550px/img0081.jpg
https://www.dermis.net/bilder/CD025/550px/img0010.jpg
https://www.dermis.net/bilder/CD193/550px/img0099.jpg
https://www.dermis.net/bilder/CD026/550px/img0009.jpg
https://www.dermis.net/bilder/CD186/550px/img0018.jpg
https://www.dermis.net/bilder/CD017/550px/img0081.jpg
https://www.dermis.net/bilder/CD013/550px/img0006.jpg
https://www.dermis.net/bilder/CD013/550px/img0010.jpg
https://www.dermis.net/bilder/CD193/550px/img0017.jpg
https://www.dermis.net/bilder/CD002/550px/img0048.jpg
https://www.dermis.net/bilder/CD193/550px/img0003.jpg
https://www.dermis.net/bilder/CD180/550px/img0028.jpg
https://www.dermis.net/bilder/CD002/550px/img0001.jpg
https://www.dermis.net/bilder/CD002/550px/img0039.jpg
https://www.dermis.net/bilder/CD042/550px/img0019.jpg
https://www.dermis.net/bilder/CD042/550px/img0020.jpg
https://www.dermis.net/bilder/CD199/550px/img0106.jpg
https://www.dermis.net/bilder/CD013/550px/img0011.jpg
https://www.dermis.net/bilder/CD189/550px/img0101.jpg
https://www.dermis.net/bilder/CD193/550px/img0164.jpg
https://www.dermis.net/bilder/CD193/550px/img0165.jpg
https://www.dermis.net/bilder/CD201/550px/img0003.jpg
https://www.dermis.net/bilder/CD002/550px/img0042.jpg
https://www.dermis.net/bilder/CD194/550px/img0165.jpg
https://www.dermis.net/bilder/CD042/550px/img0035.jpg
https://www.dermis.net/bilder/CD184/550px/img0009.jpg

https://www.dermis.net/bilder/CD187/550px/img0001.jpg
https://www.dermis.net/bilder/CD013/550px/img0012.jpg
https://www.dermis.net/bilder/CD196/550px/img0012.jpg
https://www.dermis.net/bilder/CD057/550px/img0069.jpg
https://www.dermis.net/bilder/CD184/550px/img0010.jpg
https://www.dermis.net/bilder/CD183/550px/img0070.jpg
https://www.dermis.net/bilder/CD017/550px/img0041.jpg
https://www.dermis.net/bilder/CD001/550px/img0083.jpg
https://www.dermis.net/bilder/CD001/550px/img0099.jpg
https://www.dermis.net/bilder/CD183/550px/img0002.jpg
https://www.dermis.net/bilder/CD183/550px/img0016.jpg
https://www.dermis.net/bilder/CD183/550px/img0017.jpg
https://www.dermis.net/bilder/CD183/550px/img0020.jpg
https://www.dermis.net/bilder/CD181/550px/img0070.jpg
https://www.dermis.net/bilder/CD181/550px/img0069.jpg
https://www.dermis.net/bilder/CD181/550px/img0068.jpg
https://www.dermis.net/bilder/CD192/550px/img0099.jpg
https://www.dermis.net/bilder/CD211/550px/img0076.jpg
https://www.dermis.net/bilder/CD211/550px/img0012.jpg
https://www.dermis.net/bilder/CD197/550px/img0031.jpg
https://www.dermis.net/bilder/CD194/550px/img0107.jpg
https://www.dermis.net/bilder/CD196/550px/img0034.jpg
https://www.dermis.net/bilder/CD194/550px/img0106.jpg
https://www.dermis.net/bilder/CD198/550px/img0028.jpg
https://www.dermis.net/bilder/CD198/550px/img0030.jpg
https://www.dermis.net/bilder/CD198/550px/img0142.jpg
https://www.dermis.net/bilder/CD198/550px/img0143.jpg
https://www.dermis.net/bilder/CD198/550px/img0096.jpg
https://www.dermis.net/bilder/CD055/550px/img0073.jpg
https://www.dermis.net/bilder/CD184/550px/img0111.jpg
https://www.dermis.net/bilder/CD184/550px/img0112.jpg
https://www.dermis.net/bilder/CD002/550px/img0009.jpg
https://www.dermis.net/bilder/CD002/550px/img0036.jpg
https://www.dermis.net/bilder/CD055/550px/img0009.jpg
https://www.dermis.net/bilder/CD181/550px/img0060.jpg
https://www.dermis.net/bilder/CD181/550px/img0057.jpg
https://www.dermis.net/bilder/CD182/550px/img0005.jpg
https://www.dermis.net/bilder/CD187/550px/img0002.jpg
https://www.dermis.net/bilder/CD055/550px/img0003.jpg
https://www.dermis.net/bilder/CD201/550px/img0001.jpg
https://www.dermis.net/bilder/CD193/550px/img0115.jpg
https://www.dermis.net/bilder/CD036/550px/img0013.jpg
https://www.dermis.net/bilder/CD195/550px/img0104.jpg
https://www.dermis.net/bilder/CD196/550px/img0051.jpg
https://www.dermis.net/bilder/CD196/550px/img0052.jpg
https://www.dermis.net/bilder/CD185/550px/img0017.jpg
https://www.dermis.net/bilder/CD197/550px/img0059.jpg
https://www.dermis.net/bilder/CD178/550px/img0019.jpg

https://www.dermis.net/bilder/CD178/550px/img0020.jpg
https://www.dermis.net/bilder/CD179/550px/img0019.jpg
https://www.dermis.net/bilder/CD040/550px/img0100.jpg
https://www.dermis.net/bilder/CD187/550px/img0061.jpg
https://www.dermis.net/bilder/CD184/550px/img0069.jpg
https://www.dermis.net/bilder/CD195/550px/img0105.jpg
https://www.dermis.net/bilder/CD198/550px/img0079.jpg
https://www.dermis.net/bilder/CD194/550px/img0113.jpg
https://www.dermis.net/bilder/CD196/550px/img0104.jpg
https://www.dermis.net/bilder/CD183/550px/img0069.jpg
https://www.dermis.net/bilder/CD196/550px/img0028.jpg
https://www.dermis.net/bilder/CD212/550px/img0101.jpg
https://www.dermis.net/bilder/CD196/550px/img0103.jpg
https://www.dermis.net/bilder/CD196/550px/img0154.jpg
https://www.dermis.net/bilder/CD191/550px/img0071.jpg
https://www.dermis.net/bilder/CD071/550px/img0092.jpg
https://www.dermis.net/bilder/CD040/550px/img0099.jpg
https://www.dermis.net/bilder/CD012/550px/img0065.jpg
https://www.dermis.net/bilder/CD033/550px/img0031.jpg
https://www.dermis.net/bilder/CD054/550px/img0005.jpg
https://www.dermis.net/bilder/CD007/550px/img0092.jpg
https://www.dermis.net/bilder/CD002/550px/img0026.jpg
https://www.dermis.net/bilder/CD191/550px/img0018.jpg
https://www.dermis.net/bilder/CD002/550px/img0023.jpg
https://www.dermis.net/bilder/CD022/550px/img0008.jpg
https://www.dermis.net/bilder/CD193/550px/img0065.jpg
https://www.dermis.net/bilder/CD192/550px/img0081.jpg
https://www.dermis.net/bilder/CD184/550px/img0033.jpg
https://www.dermis.net/bilder/CD002/550px/img0021.jpg
https://www.dermis.net/bilder/CD191/550px/img0051.jpg
https://www.dermis.net/bilder/CD181/550px/img0015.jpg
https://www.dermis.net/bilder/CD055/550px/img0071.jpg
https://www.dermis.net/bilder/CD002/550px/img0012.jpg
https://www.dermis.net/bilder/CD192/550px/img0082.jpg
https://www.dermis.net/bilder/CD007/550px/img0024.jpg
https://www.dermis.net/bilder/CD194/550px/img0081.jpg
https://www.dermis.net/bilder/CD194/550px/img0082.jpg
https://www.dermis.net/bilder/CD195/550px/img0011.jpg
https://www.dermis.net/bilder/CD197/550px/img0032.jpg
https://www.dermis.net/bilder/CD197/550px/img0033.jpg
https://www.dermis.net/bilder/CD190/550px/img0060.jpg
https://www.dermis.net/bilder/CD190/550px/img0061.jpg
https://www.dermis.net/bilder/CD187/550px/img0062.jpg
https://www.dermis.net/bilder/CD187/550px/img0063.jpg
https://www.dermis.net/bilder/CD179/550px/img0010.jpg
https://www.dermis.net/bilder/CD195/550px/img0026.jpg
https://www.dermis.net/bilder/CD199/550px/img0077.jpg
https://www.dermis.net/bilder/CD184/550px/img0072.jpg

https://www.dermis.net/bilder/CD182/550px/img0076.jpg
https://www.dermis.net/bilder/CD002/550px/img0018.jpg
https://www.dermis.net/bilder/CD178/550px/img0021.jpg
https://www.dermis.net/bilder/CD183/550px/img0082.jpg
https://www.dermis.net/bilder/CD196/550px/img0030.jpg
https://www.dermis.net/bilder/CD196/550px/img0031.jpg
https://www.dermis.net/bilder/CD196/550px/img0158.jpg
https://www.dermis.net/bilder/CD196/550px/img0157.jpg
https://www.dermis.net/bilder/CD183/550px/img0068.jpg
https://www.dermis.net/bilder/CD183/550px/img0071.jpg
https://www.dermis.net/bilder/CD183/550px/img0072.jpg
https://www.dermis.net/bilder/CD008/550px/img0044.jpg
https://www.dermis.net/bilder/CD008/550px/img0074.jpg
https://www.dermis.net/bilder/CD006/550px/img0015.jpg
https://www.dermis.net/bilder/CD183/550px/img0081.jpg
https://www.dermis.net/bilder/CD191/550px/img0048.jpg
https://www.dermis.net/bilder/CD180/550px/img0005.jpg
https://www.dermis.net/bilder/CD189/550px/img0095.jpg
https://www.dermis.net/bilder/CD212/550px/img0088.jpg
https://www.dermis.net/bilder/CD018/550px/img0004.jpg
https://www.dermis.net/bilder/CD039/550px/img0019.jpg
https://www.dermis.net/bilder/CD198/550px/img0084.jpg
https://www.dermis.net/bilder/CD026/550px/img0011.jpg
https://www.dermis.net/bilder/CD026/550px/img0012.jpg
https://www.dermis.net/bilder/CD026/550px/img0008.jpg
https://www.dermis.net/bilder/CD026/550px/img0010.jpg
https://www.dermis.net/bilder/CD195/550px/img0036.jpg
https://www.dermis.net/bilder/CD055/550px/img0041.jpg
https://www.dermis.net/bilder/CD057/550px/img0065.jpg
https://www.dermis.net/bilder/CD057/550px/img0018.jpg
https://www.dermis.net/bilder/CD189/550px/img0104.jpg
https://www.dermis.net/bilder/CD194/550px/img0144.jpg
https://www.dermis.net/bilder/CD198/550px/img0018.jpg
https://www.dermis.net/bilder/CD210/550px/img0140.jpg
https://www.dermis.net/bilder/CD013/550px/img0041.jpg
https://www.dermis.net/bilder/CD182/550px/img0068.jpg
https://www.dermis.net/bilder/CD180/550px/img0055.jpg
https://www.dermis.net/bilder/CD180/550px/img0009.jpg
https://www.dermis.net/bilder/CD184/550px/img0032.jpg
https://www.dermis.net/bilder/CD184/550px/img0113.jpg
https://www.dermis.net/bilder/CD191/550px/img0010.jpg
https://www.dermis.net/bilder/CD191/550px/img0081.jpg
https://www.dermis.net/bilder/CD194/550px/img0097.jpg
https://www.dermis.net/bilder/CD196/550px/img0128.jpg
https://www.dermis.net/bilder/CD196/550px/img0129.jpg
https://www.dermis.net/bilder/CD198/550px/img0125.jpg
https://www.dermis.net/bilder/CD190/550px/img0040.jpg
https://www.dermis.net/bilder/CD181/550px/img0061.jpg

https://www.dermis.net/bilder/CD183/550px/img0052.jpg
https://www.dermis.net/bilder/CD183/550px/img0053.jpg
https://www.dermis.net/bilder/CD183/550px/img0054.jpg
https://www.dermis.net/bilder/CD183/550px/img0055.jpg
https://www.dermis.net/bilder/CD192/550px/img0041.jpg
https://www.dermis.net/bilder/CD195/550px/img0006.jpg
https://www.dermis.net/bilder/CD195/550px/img0007.jpg
https://www.dermis.net/bilder/CD044/550px/img0078.jpg
https://www.dermis.net/bilder/CD026/550px/img0013.jpg
https://www.dermis.net/bilder/CD026/550px/img0014.jpg
https://www.dermis.net/bilder/CD004/550px/img0015.jpg
https://www.dermis.net/bilder/CD035/550px/img0054.jpg
https://www.dermis.net/bilder/CD036/550px/img0089.jpg
https://www.dermis.net/bilder/CD198/550px/img0114.jpg
https://www.dermis.net/bilder/CD191/550px/img0090.jpg
https://www.dermis.net/bilder/CD191/550px/img0091.jpg
https://www.dermis.net/bilder/CD191/550px/img0092.jpg
https://www.dermis.net/bilder/CD178/550px/img0095.jpg
https://www.dermis.net/bilder/CD184/550px/img0057.jpg
https://www.dermis.net/bilder/CD197/550px/img0022.jpg
https://www.dermis.net/bilder/CD035/550px/img0056.jpg
https://www.dermis.net/bilder/CD212/550px/img0108.jpg
https://www.dermis.net/bilder/CD180/550px/img0020.jpg
https://www.dermis.net/bilder/CD180/550px/img0019.jpg
https://www.dermis.net/bilder/CD184/550px/img0045.jpg
https://www.dermis.net/bilder/CD012/550px/img0075.jpg
https://www.dermis.net/bilder/CD189/550px/img0053.jpg
https://www.dermis.net/bilder/CD191/550px/img0019.jpg
https://www.dermis.net/bilder/CD191/550px/img0020.jpg
https://www.dermis.net/bilder/CD195/550px/img0025.jpg
https://www.dermis.net/bilder/CD196/550px/img0032.jpg
https://www.dermis.net/bilder/CD196/550px/img0156.jpg
https://www.dermis.net/bilder/CD193/550px/img0004.jpg
https://www.dermis.net/bilder/CD193/550px/img0008.jpg
https://www.dermis.net/bilder/CD198/550px/img0026.jpg
https://www.dermis.net/bilder/CD198/550px/img0027.jpg
https://www.dermis.net/bilder/CD012/550px/img0067.jpg
https://www.dermis.net/bilder/CD194/550px/img0168.jpg
https://www.dermis.net/bilder/CD180/550px/img0097.jpg
https://www.dermis.net/bilder/CD210/550px/img0099.jpg
https://www.dermis.net/bilder/CD025/550px/img0016.jpg
https://www.dermis.net/bilder/CD011/550px/img0020.jpg
https://www.dermis.net/bilder/CD011/550px/img0021.jpg
https://www.dermis.net/bilder/CD013/550px/img0004.jpg
https://www.dermis.net/bilder/CD194/550px/img0125.jpg
https://www.dermis.net/bilder/CD194/550px/img0126.jpg
https://www.dermis.net/bilder/CD186/550px/img0008.jpg
https://www.dermis.net/bilder/CD184/550px/img0075.jpg

https://www.dermis.net/bilder/CD195/550px/img0024.jpg
https://www.dermis.net/bilder/CD193/550px/img0156.jpg
https://www.dermis.net/bilder/CD190/550px/img0048.jpg
https://www.dermis.net/bilder/CD178/550px/img0067.jpg
https://www.dermis.net/bilder/CD197/550px/img0038.jpg
https://www.dermis.net/bilder/CD183/550px/img0028.jpg
https://www.dermis.net/bilder/CD193/550px/img0071.jpg
https://www.dermis.net/bilder/CD001/550px/img0041.jpg
https://www.dermis.net/bilder/CD015/550px/img0012.jpg
https://www.dermis.net/bilder/CD185/550px/img0021.jpg
https://www.dermis.net/bilder/CD196/550px/img0094.jpg
https://www.dermis.net/bilder/CD184/550px/img0043.jpg
https://www.dermis.net/bilder/CD211/550px/img0102.jpg
https://www.dermis.net/bilder/CD184/550px/img0044.jpg
https://www.dermis.net/bilder/CD070/550px/img0013.jpg
https://www.dermis.net/bilder/CD181/550px/img0088.jpg
https://www.dermis.net/bilder/CD183/550px/img0001.jpg
https://www.dermis.net/bilder/CD198/550px/img0022.jpg
https://www.dermis.net/bilder/CD179/550px/img0004.jpg
https://www.dermis.net/bilder/CD184/550px/img0027.jpg
https://www.dermis.net/bilder/CD044/550px/img0081.jpg
https://www.dermis.net/bilder/CD198/550px/img0077.jpg
https://www.dermis.net/bilder/CD198/550px/img0078.jpg
https://www.dermis.net/bilder/CD021/550px/img0082.jpg
https://www.dermis.net/bilder/CD193/550px/img0137.jpg
https://www.dermis.net/bilder/CD193/550px/img0138.jpg
https://www.dermis.net/bilder/CD187/550px/img0076.jpg
https://www.dermis.net/bilder/CD039/550px/img0032.jpg
https://www.dermis.net/bilder/CD199/550px/img0035.jpg
https://www.dermis.net/bilder/CD210/550px/img0089.jpg
https://www.dermis.net/bilder/CD198/550px/img0080.jpg
https://www.dermis.net/bilder/CD001/550px/img0008.jpg
https://www.dermis.net/bilder/CD001/550px/img0086.jpg
https://www.dermis.net/bilder/CD212/550px/img0113.jpg
https://www.dermis.net/bilder/CD198/550px/img0003.jpg
https://www.dermis.net/bilder/CD197/550px/img0012.jpg
https://www.dermis.net/bilder/CD211/550px/img0037.jpg
https://www.dermis.net/bilder/CD193/550px/img0128.jpg
https://www.dermis.net/bilder/CD183/550px/img0064.jpg
https://www.dermis.net/bilder/CD194/550px/img0076.jpg
https://www.dermis.net/bilder/CD193/550px/img0097.jpg
https://www.dermis.net/bilder/CD182/550px/img0078.jpg
https://www.dermis.net/bilder/CD197/550px/img0065.jpg
https://www.dermis.net/bilder/CD197/550px/img0066.jpg
https://www.dermis.net/bilder/CD193/550px/img0063.jpg
https://www.dermis.net/bilder/CD194/550px/img0011.jpg
https://www.dermis.net/bilder/CD196/550px/img0046.jpg
https://www.dermis.net/bilder/CD196/550px/img0047.jpg

https://www.dermis.net/bilder/CD196/550px/img0048.jpg
https://www.dermis.net/bilder/CD070/550px/img0023.jpg
https://www.dermis.net/bilder/CD193/550px/img0167.jpg
https://www.dermis.net/bilder/CD193/550px/img0168.jpg
https://www.dermis.net/bilder/CD212/550px/img0107.jpg
https://www.dermis.net/bilder/CD035/550px/img0090.jpg
https://www.dermis.net/bilder/CD035/550px/img0091.jpg
https://www.dermis.net/bilder/CD005/550px/img0001.jpg
https://www.dermis.net/bilder/CD184/550px/img0028.jpg
https://www.dermis.net/bilder/CD184/550px/img0029.jpg
https://www.dermis.net/bilder/CD182/550px/img0057.jpg
https://www.dermis.net/bilder/CD182/550px/img0058.jpg
https://www.dermis.net/bilder/CD194/550px/img0054.jpg
https://www.dermis.net/bilder/CD194/550px/img0055.jpg
https://www.dermis.net/bilder/CD198/550px/img0023.jpg
https://www.dermis.net/bilder/CD211/550px/img0054.jpg
https://www.dermis.net/bilder/CD198/550px/img0024.jpg
https://www.dermis.net/bilder/CD198/550px/img0025.jpg
https://www.dermis.net/bilder/CD182/550px/img0016.jpg
https://www.dermis.net/bilder/CD182/550px/img0017.jpg
https://www.dermis.net/bilder/CD008/550px/img0021.jpg
https://www.dermis.net/bilder/CD210/550px/img0043.jpg
https://www.dermis.net/bilder/CD035/550px/img0094.jpg
https://www.dermis.net/bilder/CD212/550px/img0013.jpg
https://www.dermis.net/bilder/CD212/550px/img0014.jpg
https://www.dermis.net/bilder/CD021/550px/img0058.jpg
https://www.dermis.net/bilder/CD185/550px/img0001.jpg
https://www.dermis.net/bilder/CD185/550px/img0002.jpg
https://www.dermis.net/bilder/CD182/550px/img0034.jpg
https://www.dermis.net/bilder/CD182/550px/img0033.jpg
https://www.dermis.net/bilder/CD211/550px/img0025.jpg
https://www.dermis.net/bilder/CD191/550px/img0011.jpg
https://www.dermis.net/bilder/CD191/550px/img0012.jpg
https://www.dermis.net/bilder/CD198/550px/img0085.jpg
https://www.dermis.net/bilder/CD210/550px/img0023.jpg
https://www.dermis.net/bilder/CD008/550px/img0038.jpg
https://www.dermis.net/bilder/CD008/550px/img0043.jpg
https://www.dermis.net/bilder/CD187/550px/img0039.jpg
https://www.dermis.net/bilder/CD187/550px/img0040.jpg
https://www.dermis.net/bilder/CD187/550px/img0041.jpg
https://www.dermis.net/bilder/CD187/550px/img0042.jpg
https://www.dermis.net/bilder/CD187/550px/img0043.jpg
https://www.dermis.net/bilder/CD189/550px/img0103.jpg
https://www.dermis.net/bilder/CD199/550px/img0025.jpg
https://www.dermis.net/bilder/CD194/550px/img0031.jpg
https://www.dermis.net/bilder/CD194/550px/img0032.jpg
https://www.dermis.net/bilder/CD194/550px/img0033.jpg
https://www.dermis.net/bilder/CD194/550px/img0166.jpg

https://www.dermis.net/bilder/CD198/550px/img0056.jpg
https://www.dermis.net/bilder/CD198/550px/img0057.jpg
https://www.dermis.net/bilder/CD210/550px/img0018.jpg
https://www.dermis.net/bilder/CD198/550px/img0159.jpg
https://www.dermis.net/bilder/CD190/550px/img0021.jpg
https://www.dermis.net/bilder/CD190/550px/img0022.jpg
https://www.dermis.net/bilder/CD194/550px/img0127.jpg
https://www.dermis.net/bilder/CD194/550px/img0128.jpg
https://www.dermis.net/bilder/CD198/550px/img0097.jpg
https://www.dermis.net/bilder/CD198/550px/img0098.jpg
https://www.dermis.net/bilder/CD198/550px/img0101.jpg
https://www.dermis.net/bilder/CD196/550px/img0102.jpg
https://www.dermis.net/bilder/CD036/550px/img0085.jpg
https://www.dermis.net/bilder/CD054/550px/img0022.jpg
https://www.dermis.net/bilder/CD054/550px/img0014.jpg
https://www.dermis.net/bilder/CD026/550px/img0031.jpg
https://www.dermis.net/bilder/CD042/550px/img0081.jpg
https://www.dermis.net/bilder/CD178/550px/img0068.jpg
https://www.dermis.net/bilder/CD198/550px/img0054.jpg
https://www.dermis.net/bilder/CD211/550px/img0101.jpg
https://www.dermis.net/bilder/CD025/550px/img0072.jpg
https://www.dermis.net/bilder/CD002/550px/img0062.jpg
https://www.dermis.net/bilder/CD194/550px/img0163.jpg
https://www.dermis.net/bilder/CD212/550px/img0110.jpg
https://www.dermis.net/bilder/CD032/550px/img0088.jpg
https://www.dermis.net/bilder/CD032/550px/img0090.jpg
https://www.dermis.net/bilder/CD182/550px/img0004.jpg
https://www.dermis.net/bilder/CD182/550px/img0003.jpg
https://www.dermis.net/bilder/CD180/550px/img0027.jpg
https://www.dermis.net/bilder/CD178/550px/img0066.jpg
https://www.dermis.net/bilder/CD196/550px/img0079.jpg
https://www.dermis.net/bilder/CD196/550px/img0080.jpg
https://www.dermis.net/bilder/CD178/550px/img0008.jpg
https://www.dermis.net/bilder/CD211/550px/img0096.jpg
https://www.dermis.net/bilder/CD198/550px/img0040.jpg
https://www.dermis.net/bilder/CD198/550px/img0041.jpg
https://www.dermis.net/bilder/CD055/550px/img0072.jpg
https://www.dermis.net/bilder/CD196/550px/img0007.jpg
https://www.dermis.net/bilder/CD196/550px/img0008.jpg
https://www.dermis.net/bilder/CD181/550px/img0090.jpg
https://www.dermis.net/bilder/CD179/550px/img0030.jpg
https://www.dermis.net/bilder/CD211/550px/img0027.jpg
https://www.dermis.net/bilder/CD211/550px/img0028.jpg
https://www.dermis.net/bilder/CD040/550px/img0074.jpg
https://www.dermis.net/bilder/CD194/550px/img0161.jpg
https://www.dermis.net/bilder/CD189/550px/img0105.jpg
https://www.dermis.net/bilder/CD194/550px/img0119.jpg
https://www.dermis.net/bilder/CD197/550px/img0074.jpg

https://www.dermis.net/bilder/CD194/550px/img0049.jpg
https://www.dermis.net/bilder/CD198/550px/img0081.jpg
https://www.dermis.net/bilder/CD198/550px/img0072.jpg
https://www.dermis.net/bilder/CD198/550px/img0009.jpg
https://www.dermis.net/bilder/CD211/550px/img0056.jpg
https://www.dermis.net/bilder/CD012/550px/img0074.jpg
https://www.dermis.net/bilder/CD029/550px/img0013.jpg
https://www.dermis.net/bilder/CD182/550px/img0021.jpg
https://www.dermis.net/bilder/CD182/550px/img0020.jpg
https://www.dermis.net/bilder/CD182/550px/img0019.jpg
https://www.dermis.net/bilder/CD182/550px/img0018.jpg
https://www.dermis.net/bilder/CD055/550px/img0074.jpg
https://www.dermis.net/bilder/CD035/550px/img0078.jpg
https://www.dermis.net/bilder/CD002/550px/img0022.jpg
https://www.dermis.net/bilder/CD002/550px/img0047.jpg
https://www.dermis.net/bilder/CD193/550px/img0066.jpg
https://www.dermis.net/bilder/CD194/550px/img0135.jpg
https://www.dermis.net/bilder/CD057/550px/img0015.jpg
https://www.dermis.net/bilder/CD002/550px/img0004.jpg
https://www.dermis.net/bilder/CD013/550px/img0035.jpg
https://www.dermis.net/bilder/CD013/550px/img0036.jpg
https://www.dermis.net/bilder/CD013/550px/img0037.jpg
https://www.dermis.net/bilder/CD018/550px/img0114.jpg
https://www.dermis.net/bilder/CD002/550px/img0029.jpg
https://www.dermis.net/bilder/CD184/550px/img0053.jpg
https://www.dermis.net/bilder/CD002/550px/img0027.jpg
https://www.dermis.net/bilder/CD033/550px/img0039.jpg
https://www.dermis.net/bilder/CD186/550px/img0007.jpg
https://www.dermis.net/bilder/CD194/550px/img0167.jpg
https://www.dermis.net/bilder/CD033/550px/img0022.jpg
https://www.dermis.net/bilder/CD193/550px/img0014.jpg
https://www.dermis.net/bilder/CD193/550px/img0015.jpg
https://www.dermis.net/bilder/CD193/550px/img0016.jpg
https://www.dermis.net/bilder/CD181/550px/img0014.jpg
https://www.dermis.net/bilder/CD002/550px/img0033.jpg
https://www.dermis.net/bilder/CD033/550px/img0003.jpg
https://www.dermis.net/bilder/CD194/550px/img0079.jpg
https://www.dermis.net/bilder/CD194/550px/img0080.jpg
https://www.dermis.net/bilder/CD196/550px/img0112.jpg
https://www.dermis.net/bilder/CD196/550px/img0114.jpg
https://www.dermis.net/bilder/CD197/550px/img0058.jpg
https://www.dermis.net/bilder/CD193/550px/img0053.jpg
https://www.dermis.net/bilder/CD193/550px/img0054.jpg
https://www.dermis.net/bilder/CD002/550px/img0003.jpg
https://www.dermis.net/bilder/CD033/550px/img0004.jpg
https://www.dermis.net/bilder/CD033/550px/img0016.jpg
https://www.dermis.net/bilder/CD210/550px/img0028.jpg
https://www.dermis.net/bilder/CD196/550px/img0033.jpg

https://www.dermis.net/bilder/CD196/550px/img0155.jpg
https://www.dermis.net/bilder/CD193/550px/img0001.jpg
https://www.dermis.net/bilder/CD193/550px/img0002.jpg
https://www.dermis.net/bilder/CD193/550px/img0006.jpg
https://www.dermis.net/bilder/CD007/550px/img0090.jpg
https://www.dermis.net/bilder/CD194/550px/img0111.jpg
https://www.dermis.net/bilder/CD194/550px/img0112.jpg
https://www.dermis.net/bilder/CD191/550px/img0005.jpg
https://www.dermis.net/bilder/CD002/550px/img0008.jpg
https://www.dermis.net/bilder/CD055/550px/img0075.jpg
https://www.dermis.net/bilder/CD002/550px/img0005.jpg
https://www.dermis.net/bilder/CD196/550px/img0029.jpg
https://www.dermis.net/bilder/CD033/550px/img0015.jpg
https://www.dermis.net/bilder/CD211/550px/img0009.jpg
https://www.dermis.net/bilder/CD029/550px/img0008.jpg
https://www.dermis.net/bilder/CD041/550px/img0093.jpg
https://www.dermis.net/bilder/CD198/550px/img0075.jpg
https://www.dermis.net/bilder/CD196/550px/img0095.jpg
https://www.dermis.net/bilder/CD196/550px/img0096.jpg
https://www.dermis.net/bilder/CD195/550px/img0085.jpg
https://www.dermis.net/bilder/CD195/550px/img0086.jpg
https://www.dermis.net/bilder/CD212/550px/img0082.jpg
https://www.dermis.net/bilder/CD035/550px/img0083.jpg
https://www.dermis.net/bilder/CD187/550px/img0044.jpg
https://www.dermis.net/bilder/CD187/550px/img0045.jpg
https://www.dermis.net/bilder/CD187/550px/img0046.jpg
https://www.dermis.net/bilder/CD197/550px/img0029.jpg
https://www.dermis.net/bilder/CD197/550px/img0030.jpg
https://www.dermis.net/bilder/CD002/550px/img0045.jpg
https://www.dermis.net/bilder/CD199/550px/img0126.jpg
https://www.dermis.net/bilder/CD055/550px/img0064.jpg
https://www.dermis.net/bilder/CD002/550px/img0053.jpg
https://www.dermis.net/bilder/CD041/550px/img0056.jpg
https://www.dermis.net/bilder/CD183/550px/img0046.jpg
https://www.dermis.net/bilder/CD212/550px/img0010.jpg
https://www.dermis.net/bilder/CD195/550px/img0032.jpg
https://www.dermis.net/bilder/CD025/550px/img0018.jpg
https://www.dermis.net/bilder/CD002/550px/img0055.jpg
https://www.dermis.net/bilder/CD013/550px/img0024.jpg
https://www.dermis.net/bilder/CD001/550px/img0093.jpg
https://www.dermis.net/bilder/CD034/550px/img0056.jpg
https://www.dermis.net/bilder/CD056/550px/img0019.jpg
https://www.dermis.net/bilder/CD069/550px/img0113.jpg
https://www.dermis.net/bilder/CD070/550px/img0086.jpg
https://www.dermis.net/bilder/CD051/550px/img0061.jpg
https://www.dermis.net/bilder/CD051/550px/img0062.jpg
https://www.dermis.net/bilder/CD036/550px/img0052.jpg
https://www.dermis.net/bilder/CD025/550px/img0052.jpg

https://www.dermis.net/bilder/CD001/550px/img0007.jpg
https://www.dermis.net/bilder/CD071/550px/img0067.jpg
https://www.dermis.net/bilder/CD050/550px/img0009.jpg
https://www.dermis.net/bilder/CD004/550px/img0003.jpg
https://www.dermis.net/bilder/CD054/550px/img0008.jpg
https://www.dermis.net/bilder/CD069/550px/img0087.jpg
https://www.dermis.net/bilder/CD071/550px/img0036.jpg
https://www.dermis.net/bilder/CD050/550px/img0070.jpg
https://www.dermis.net/bilder/CD006/550px/img0048.jpg
https://www.dermis.net/bilder/CD014/550px/img0091.jpg
https://www.dermis.net/bilder/CD056/550px/img0033.jpg
https://www.dermis.net/bilder/CD041/550px/img0020.jpg
https://www.dermis.net/bilder/CD005/550px/img0072.jpg
https://www.dermis.net/bilder/CD037/550px/img0052.jpg
https://www.dermis.net/bilder/CD006/550px/img0075.jpg
https://www.dermis.net/bilder/CD026/550px/img0034.jpg
https://www.dermis.net/bilder/CD071/550px/img0028.jpg
https://www.dermis.net/bilder/CD044/550px/img0024.jpg
https://www.dermis.net/bilder/CD044/550px/img0037.jpg
https://www.dermis.net/bilder/CD057/550px/img0036.jpg
https://www.dermis.net/bilder/CD007/550px/img0031.jpg
https://www.dermis.net/bilder/CD025/550px/img0034.jpg
https://www.dermis.net/bilder/CD025/550px/img0036.jpg
https://www.dermis.net/bilder/CD006/550px/img0065.jpg
https://www.dermis.net/bilder/CD026/550px/img0016.jpg
https://www.dermis.net/bilder/CD040/550px/img0041.jpg
https://www.dermis.net/bilder/CD057/550px/img0023.jpg
https://www.dermis.net/bilder/CD002/550px/img0050.jpg
https://www.dermis.net/bilder/CD026/550px/img0090.jpg
https://www.dermis.net/bilder/CD026/550px/img0091.jpg
https://www.dermis.net/bilder/CD027/550px/img0002.jpg
https://www.dermis.net/bilder/CD027/550px/img0001.jpg
https://www.dermis.net/bilder/CD002/550px/img0049.jpg
https://www.dermis.net/bilder/CD044/550px/img0013.jpg
https://www.dermis.net/bilder/CD044/550px/img0014.jpg
https://www.dermis.net/bilder/CD006/550px/img0060.jpg
https://www.dermis.net/bilder/CD046/550px/img0011.jpg
https://www.dermis.net/bilder/CD026/550px/img0042.jpg
https://www.dermis.net/bilder/CD051/550px/img0016.jpg
https://www.dermis.net/bilder/CD005/550px/img0055.jpg
https://www.dermis.net/bilder/CD191/550px/img0014.jpg
https://www.dermis.net/bilder/CD026/550px/img0089.jpg
https://www.dermis.net/bilder/CD044/550px/img0011.jpg
https://www.dermis.net/bilder/CD184/550px/img0093.jpg
https://www.dermis.net/bilder/CD190/550px/img0067.jpg
https://www.dermis.net/bilder/CD041/550px/img0057.jpg
https://www.dermis.net/bilder/CD040/550px/img0030.jpg
https://www.dermis.net/bilder/CD013/550px/img0001.jpg

https://www.dermis.net/bilder/CD042/550px/img0068.jpg
https://www.dermis.net/bilder/CD193/550px/img0145.jpg
https://www.dermis.net/bilder/CD003/550px/img0062.jpg
https://www.dermis.net/bilder/CD001/550px/img0030.jpg
https://www.dermis.net/bilder/CD001/550px/img0059.jpg
https://www.dermis.net/bilder/CD195/550px/img0040.jpg
https://www.dermis.net/bilder/CD183/550px/img0092.jpg
https://www.dermis.net/bilder/CD198/550px/img0131.jpg
https://www.dermis.net/bilder/CD198/550px/img0132.jpg
https://www.dermis.net/bilder/CD198/550px/img0160.jpg
https://www.dermis.net/bilder/CD025/550px/img0019.jpg
https://www.dermis.net/bilder/CD012/550px/img0027.jpg
https://www.dermis.net/bilder/CD212/550px/img0011.jpg
https://www.dermis.net/bilder/CD051/550px/img0006.jpg
https://www.dermis.net/bilder/CD085/550px/img0080.jpg
https://www.dermis.net/bilder/CD184/550px/img0038.jpg
https://www.dermis.net/bilder/CD184/550px/img0039.jpg
https://www.dermis.net/bilder/CD178/550px/img0092.jpg
https://www.dermis.net/bilder/CD181/550px/img0039.jpg
https://www.dermis.net/bilder/CD181/550px/img0040.jpg
https://www.dermis.net/bilder/CD001/550px/img0024.jpg
https://www.dermis.net/bilder/CD192/550px/img0071.jpg
https://www.dermis.net/bilder/CD192/550px/img0072.jpg
https://www.dermis.net/bilder/CD189/550px/img0035.jpg
https://www.dermis.net/bilder/CD189/550px/img0036.jpg
https://www.dermis.net/bilder/CD191/550px/img0064.jpg
https://www.dermis.net/bilder/CD198/550px/img0042.jpg
https://www.dermis.net/bilder/CD198/550px/img0043.jpg
https://www.dermis.net/bilder/CD198/550px/img0044.jpg
https://www.dermis.net/bilder/CD198/550px/img0045.jpg
https://www.dermis.net/bilder/CD021/550px/img0098.jpg
https://www.dermis.net/bilder/CD035/550px/img0072.jpg
https://www.dermis.net/bilder/CD043/550px/img0004.jpg
https://www.dermis.net/bilder/CD184/550px/img0014.jpg
https://www.dermis.net/bilder/CD190/550px/img0055.jpg
https://www.dermis.net/bilder/CD190/550px/img0056.jpg
https://www.dermis.net/bilder/CD193/550px/img0117.jpg
https://www.dermis.net/bilder/CD181/550px/img0017.jpg
https://www.dermis.net/bilder/CD185/550px/img0025.jpg
https://www.dermis.net/bilder/CD013/550px/img0007.jpg
https://www.dermis.net/bilder/CD054/550px/img0020.jpg
https://www.dermis.net/bilder/CD051/550px/img0060.jpg
https://www.dermis.net/bilder/CD018/550px/img0072.jpg
https://www.dermis.net/bilder/CD016/550px/img0022.jpg
https://www.dermis.net/bilder/CD018/550px/img0013.jpg
https://www.dermis.net/bilder/CD037/550px/img0002.jpg
https://www.dermis.net/bilder/CD005/550px/img0045.jpg
https://www.dermis.net/bilder/CD035/550px/img0046.jpg

https://www.dermis.net/bilder/CD052/550px/img0067.jpg
https://www.dermis.net/bilder/CD003/550px/img0074.jpg
https://www.dermis.net/bilder/CD001/550px/img0096.jpg
https://www.dermis.net/bilder/CD069/550px/img0002.jpg
https://www.dermis.net/bilder/CD069/550px/img0003.jpg
https://www.dermis.net/bilder/CD018/550px/img0016.jpg
https://www.dermis.net/bilder/CD071/550px/img0018.jpg
https://www.dermis.net/bilder/CD071/550px/img0019.jpg
https://www.dermis.net/bilder/CD071/550px/img0020.jpg
https://www.dermis.net/bilder/CD069/550px/img0089.jpg
https://www.dermis.net/bilder/CD034/550px/img0055.jpg
https://www.dermis.net/bilder/CD040/550px/img0065.jpg
https://www.dermis.net/bilder/CD054/550px/img0021.jpg
https://www.dermis.net/bilder/CD056/550px/img0052.jpg
https://www.dermis.net/bilder/CD069/550px/img0094.jpg
https://www.dermis.net/bilder/CD069/550px/img0095.jpg
https://www.dermis.net/bilder/CD036/550px/img0072.jpg
https://www.dermis.net/bilder/CD056/550px/img0053.jpg
https://www.dermis.net/bilder/CD040/550px/img0020.jpg
https://www.dermis.net/bilder/CD040/550px/img0017.jpg
https://www.dermis.net/bilder/CD003/550px/img0032.jpg
https://www.dermis.net/bilder/CD007/550px/img0012.jpg
https://www.dermis.net/bilder/CD020/550px/img0002.jpg
https://www.dermis.net/bilder/CD001/550px/img0060.jpg
https://www.dermis.net/bilder/CD020/550px/img0001.jpg
https://www.dermis.net/bilder/CD056/550px/img0061.jpg
https://www.dermis.net/bilder/CD069/550px/img0106.jpg
https://www.dermis.net/bilder/CD016/550px/img0050.jpg
https://www.dermis.net/bilder/CD034/550px/img0062.jpg
https://www.dermis.net/bilder/CD034/550px/img0063.jpg
https://www.dermis.net/bilder/CD001/550px/img0074.jpg
https://www.dermis.net/bilder/CD070/550px/img0003.jpg
https://www.dermis.net/bilder/CD071/550px/img0048.jpg
https://www.dermis.net/bilder/CD006/550px/img0041.jpg
https://www.dermis.net/bilder/CD035/550px/img0009.jpg
https://www.dermis.net/bilder/CD069/550px/img0081.jpg
https://www.dermis.net/bilder/CD014/550px/img0044.jpg
https://www.dermis.net/bilder/CD018/550px/img0046.jpg
https://www.dermis.net/bilder/CD036/550px/img0069.jpg
https://www.dermis.net/bilder/CD050/550px/img0067.jpg
https://www.dermis.net/bilder/CD056/550px/img0040.jpg
https://www.dermis.net/bilder/CD036/550px/img0007.jpg
https://www.dermis.net/bilder/CD056/550px/img0041.jpg
https://www.dermis.net/bilder/CD071/550px/img0072.jpg
https://www.dermis.net/bilder/CD036/550px/img0006.jpg
https://www.dermis.net/bilder/CD014/550px/img0067.jpg
https://www.dermis.net/bilder/CD035/550px/img0040.jpg
https://www.dermis.net/bilder/CD035/550px/img0041.jpg

https://www.dermis.net/bilder/CD006/550px/img0068.jpg
https://www.dermis.net/bilder/CD014/550px/img0068.jpg
https://www.dermis.net/bilder/CD070/550px/img0014.jpg
https://www.dermis.net/bilder/CD003/550px/img0097.jpg
https://www.dermis.net/bilder/CD056/550px/img0003.jpg
https://www.dermis.net/bilder/CD035/550px/img0038.jpg
https://www.dermis.net/bilder/CD035/550px/img0039.jpg
https://www.dermis.net/bilder/CD070/550px/img0005.jpg
https://www.dermis.net/bilder/CD069/550px/img0100.jpg
https://www.dermis.net/bilder/CD004/550px/img0084.jpg
https://www.dermis.net/bilder/CD036/550px/img0033.jpg
https://www.dermis.net/bilder/CD047/550px/img0029.jpg
https://www.dermis.net/bilder/CD005/550px/img0070.jpg
https://www.dermis.net/bilder/CD006/550px/img0013.jpg
https://www.dermis.net/bilder/CD005/550px/img0058.jpg
https://www.dermis.net/bilder/CD051/550px/img0075.jpg
https://www.dermis.net/bilder/CD004/550px/img0079.jpg
https://www.dermis.net/bilder/CD069/550px/img0088.jpg
https://www.dermis.net/bilder/CD018/550px/img0059.jpg
https://www.dermis.net/bilder/CD051/550px/img0074.jpg
https://www.dermis.net/bilder/CD004/550px/img0005.jpg
https://www.dermis.net/bilder/CD043/550px/img0012.jpg
https://www.dermis.net/bilder/CD024/550px/img0018.jpg
https://www.dermis.net/bilder/CD070/550px/img0007.jpg
https://www.dermis.net/bilder/CD043/550px/img0011.jpg
https://www.dermis.net/bilder/CD004/550px/img0078.jpg
https://www.dermis.net/bilder/CD014/550px/img0022.jpg
https://www.dermis.net/bilder/CD018/550px/img0070.jpg
https://www.dermis.net/bilder/CD002/550px/img0060.jpg
https://www.dermis.net/bilder/CD015/550px/img0052.jpg
https://www.dermis.net/bilder/CD025/550px/img0059.jpg
https://www.dermis.net/bilder/CD025/550px/img0060.jpg
https://www.dermis.net/bilder/CD028/550px/img0061.jpg
https://www.dermis.net/bilder/CD028/550px/img0063.jpg
https://www.dermis.net/bilder/CD021/550px/img0069.jpg
https://www.dermis.net/bilder/CD006/550px/img0053.jpg
https://www.dermis.net/bilder/CD016/550px/img0045.jpg
https://www.dermis.net/bilder/CD023/550px/img0019.jpg
https://www.dermis.net/bilder/CD023/550px/img0020.jpg
https://www.dermis.net/bilder/CD028/550px/img0062.jpg
https://www.dermis.net/bilder/CD018/550px/img0045.jpg
https://www.dermis.net/bilder/CD003/550px/img0086.jpg
https://www.dermis.net/bilder/CD054/550px/img0013.jpg
https://www.dermis.net/bilder/CD041/550px/img0074.jpg
https://www.dermis.net/bilder/CD041/550px/img0075.jpg
https://www.dermis.net/bilder/CD014/550px/img0041.jpg
https://www.dermis.net/bilder/CD052/550px/img0058.jpg
https://www.dermis.net/bilder/CD003/550px/img0039.jpg

https://www.dermis.net/bilder/CD014/550px/img0039.jpg
https://www.dermis.net/bilder/CD016/550px/img0048.jpg
https://www.dermis.net/bilder/CD026/550px/img0054.jpg
https://www.dermis.net/bilder/CD026/550px/img0053.jpg
https://www.dermis.net/bilder/CD071/550px/img0005.jpg
https://www.dermis.net/bilder/CD026/550px/img0055.jpg
https://www.dermis.net/bilder/CD003/550px/img0035.jpg
https://www.dermis.net/bilder/CD014/550px/img0030.jpg
https://www.dermis.net/bilder/CD003/550px/img0045.jpg
https://www.dermis.net/bilder/CD003/550px/img0090.jpg
https://www.dermis.net/bilder/CD004/550px/img0082.jpg
https://www.dermis.net/bilder/CD005/550px/img0044.jpg
https://www.dermis.net/bilder/CD004/550px/img0063.jpg
https://www.dermis.net/bilder/CD016/550px/img0020.jpg
https://www.dermis.net/bilder/CD037/550px/img0022.jpg
https://www.dermis.net/bilder/CD036/550px/img0028.jpg
https://www.dermis.net/bilder/CD024/550px/img0035.jpg
https://www.dermis.net/bilder/CD024/550px/img0036.jpg
https://www.dermis.net/bilder/CD026/550px/img0003.jpg
https://www.dermis.net/bilder/CD026/550px/img0001.jpg
https://www.dermis.net/bilder/CD026/550px/img0002.jpg
https://www.dermis.net/bilder/CD004/550px/img0056.jpg
https://www.dermis.net/bilder/CD018/550px/img0099.jpg
https://www.dermis.net/bilder/CD027/550px/img0046.jpg
https://www.dermis.net/bilder/CD003/550px/img0040.jpg
https://www.dermis.net/bilder/CD008/550px/img0049.jpg
https://www.dermis.net/bilder/CD014/550px/img0061.jpg
https://www.dermis.net/bilder/CD014/550px/img0062.jpg
https://www.dermis.net/bilder/CD044/550px/img0079.jpg
https://www.dermis.net/bilder/CD018/550px/img0001.jpg
https://www.dermis.net/bilder/CD056/550px/img0004.jpg
https://www.dermis.net/bilder/CD018/550px/img0076.jpg
https://www.dermis.net/bilder/CD014/550px/img0021.jpg
https://www.dermis.net/bilder/CD006/550px/img0071.jpg
https://www.dermis.net/bilder/CD014/550px/img0037.jpg
https://www.dermis.net/bilder/CD042/550px/img0076.jpg
https://www.dermis.net/bilder/CD008/550px/img0032.jpg
https://www.dermis.net/bilder/CD033/550px/img0083.jpg
https://www.dermis.net/bilder/CD034/550px/img0022.jpg
https://www.dermis.net/bilder/CD034/550px/img0023.jpg
https://www.dermis.net/bilder/CD034/550px/img0024.jpg
https://www.dermis.net/bilder/CD041/550px/img0021.jpg
https://www.dermis.net/bilder/CD040/550px/img0039.jpg
https://www.dermis.net/bilder/CD040/550px/img0040.jpg
https://www.dermis.net/bilder/CD055/550px/img0089.jpg
https://www.dermis.net/bilder/CD020/550px/img0094.jpg
https://www.dermis.net/bilder/CD050/550px/img0001.jpg
https://www.dermis.net/bilder/CD049/550px/img0049.jpg

https://www.dermis.net/bilder/CD050/550px/img0006.jpg
https://www.dermis.net/bilder/CD047/550px/img0076.jpg
https://www.dermis.net/bilder/CD037/550px/img0049.jpg
https://www.dermis.net/bilder/CD024/550px/img0090.jpg
https://www.dermis.net/bilder/CD049/550px/img0080.jpg
https://www.dermis.net/bilder/CD047/550px/img0074.jpg
https://www.dermis.net/bilder/CD047/550px/img0075.jpg
https://www.dermis.net/bilder/CD024/550px/img0020.jpg
https://www.dermis.net/bilder/CD037/550px/img0051.jpg
https://www.dermis.net/bilder/CD024/550px/img0095.jpg
https://www.dermis.net/bilder/CD028/550px/img0038.jpg
https://www.dermis.net/bilder/CD028/550px/img0040.jpg
https://www.dermis.net/bilder/CD036/550px/img0057.jpg
https://www.dermis.net/bilder/CD028/550px/img0042.jpg
https://www.dermis.net/bilder/CD028/550px/img0043.jpg
https://www.dermis.net/bilder/CD019/550px/img0006.jpg
https://www.dermis.net/bilder/CD019/550px/img0007.jpg
https://www.dermis.net/bilder/CD028/550px/img0041.jpg
https://www.dermis.net/bilder/CD026/550px/img0035.jpg
https://www.dermis.net/bilder/CD039/550px/img0078.jpg
https://www.dermis.net/bilder/CD039/550px/img0079.jpg
https://www.dermis.net/bilder/CD051/550px/img0023.jpg
https://www.dermis.net/bilder/CD042/550px/img0021.jpg
https://www.dermis.net/bilder/CD024/550px/img0100.jpg
https://www.dermis.net/bilder/CD034/550px/img0090.jpg
https://www.dermis.net/bilder/CD019/550px/img0001.jpg
https://www.dermis.net/bilder/CD019/550px/img0002.jpg
https://www.dermis.net/bilder/CD058/550px/img0022.jpg
https://www.dermis.net/bilder/CD005/550px/img0073.jpg
https://www.dermis.net/bilder/CD003/550px/img0023.jpg
https://www.dermis.net/bilder/CD003/550px/img0024.jpg
https://www.dermis.net/bilder/CD014/550px/img0058.jpg
https://www.dermis.net/bilder/CD021/550px/img0073.jpg
https://www.dermis.net/bilder/CD032/550px/img0094.jpg
https://www.dermis.net/bilder/CD032/550px/img0095.jpg
https://www.dermis.net/bilder/CD004/550px/img0019.jpg
https://www.dermis.net/bilder/CD004/550px/img0020.jpg
https://www.dermis.net/bilder/CD018/550px/img0006.jpg
https://www.dermis.net/bilder/CD004/550px/img0018.jpg
https://www.dermis.net/bilder/CD014/550px/img0060.jpg
https://www.dermis.net/bilder/CD018/550px/img0087.jpg
https://www.dermis.net/bilder/CD018/550px/img0011.jpg
https://www.dermis.net/bilder/CD039/550px/img0013.jpg
https://www.dermis.net/bilder/CD039/550px/img0014.jpg
https://www.dermis.net/bilder/CD039/550px/img0015.jpg
https://www.dermis.net/bilder/CD004/550px/img0061.jpg
https://www.dermis.net/bilder/CD039/550px/img0058.jpg
https://www.dermis.net/bilder/CD004/550px/img0062.jpg

https://www.dermis.net/bilder/CD007/550px/img0050.jpg
https://www.dermis.net/bilder/CD049/550px/img0057.jpg
https://www.dermis.net/bilder/CD007/550px/img0055.jpg
https://www.dermis.net/bilder/CD054/550px/img0058.jpg
https://www.dermis.net/bilder/CD036/550px/img0100.jpg
https://www.dermis.net/bilder/CD044/550px/img0030.jpg
https://www.dermis.net/bilder/CD054/550px/img0071.jpg
https://www.dermis.net/bilder/CD054/550px/img0072.jpg
https://www.dermis.net/bilder/CD006/550px/img0059.jpg
https://www.dermis.net/bilder/CD015/550px/img0007.jpg
https://www.dermis.net/bilder/CD047/550px/img0088.jpg
https://www.dermis.net/bilder/CD005/550px/img0079.jpg
https://www.dermis.net/bilder/CD018/550px/img0089.jpg
https://www.dermis.net/bilder/CD018/550px/img0088.jpg
https://www.dermis.net/bilder/CD032/550px/img0019.jpg
https://www.dermis.net/bilder/CD032/550px/img0020.jpg
https://www.dermis.net/bilder/CD032/550px/img0021.jpg
https://www.dermis.net/bilder/CD046/550px/img0061.jpg
https://www.dermis.net/bilder/CD046/550px/img0067.jpg
https://www.dermis.net/bilder/CD046/550px/img0068.jpg
https://www.dermis.net/bilder/CD018/550px/img0077.jpg
https://www.dermis.net/bilder/CD023/550px/img0081.jpg
https://www.dermis.net/bilder/CD023/550px/img0082.jpg
https://www.dermis.net/bilder/CD024/550px/img0028.jpg
https://www.dermis.net/bilder/CD008/550px/img0039.jpg
https://www.dermis.net/bilder/CD026/550px/img0085.jpg
https://www.dermis.net/bilder/CD056/550px/img0032.jpg
https://www.dermis.net/bilder/CD044/550px/img0068.jpg
https://www.dermis.net/bilder/CD044/550px/img0050.jpg
https://www.dermis.net/bilder/CD020/550px/img0006.jpg
https://www.dermis.net/bilder/CD020/550px/img0007.jpg
https://www.dermis.net/bilder/CD050/550px/img0038.jpg
https://www.dermis.net/bilder/CD050/550px/img0039.jpg
https://www.dermis.net/bilder/CD032/550px/img0016.jpg
https://www.dermis.net/bilder/CD032/550px/img0017.jpg
https://www.dermis.net/bilder/CD052/550px/img0059.jpg
https://www.dermis.net/bilder/CD051/550px/img0079.jpg
https://www.dermis.net/bilder/CD005/550px/img0086.jpg
https://www.dermis.net/bilder/CD005/550px/img0096.jpg
https://www.dermis.net/bilder/CD004/550px/img0051.jpg
https://www.dermis.net/bilder/CD005/550px/img0047.jpg
https://www.dermis.net/bilder/CD005/550px/img0049.jpg
https://www.dermis.net/bilder/CD023/550px/img0068.jpg
https://www.dermis.net/bilder/CD004/550px/img0097.jpg
https://www.dermis.net/bilder/CD023/550px/img0085.jpg
https://www.dermis.net/bilder/CD023/550px/img0086.jpg
https://www.dermis.net/bilder/CD017/550px/img0083.jpg
https://www.dermis.net/bilder/CD039/550px/img0071.jpg

https://www.dermis.net/bilder/CD039/550px/img0072.jpg
https://www.dermis.net/bilder/CD042/550px/img0072.jpg
https://www.dermis.net/bilder/CD032/550px/img0098.jpg
https://www.dermis.net/bilder/CD032/550px/img0099.jpg
https://www.dermis.net/bilder/CD039/550px/img0073.jpg
https://www.dermis.net/bilder/CD035/550px/img0049.jpg
https://www.dermis.net/bilder/CD035/550px/img0050.jpg
https://www.dermis.net/bilder/CD036/550px/img0078.jpg
https://www.dermis.net/bilder/CD051/550px/img0080.jpg
https://www.dermis.net/bilder/CD033/550px/img0097.jpg
https://www.dermis.net/bilder/CD036/550px/img0075.jpg
https://www.dermis.net/bilder/CD036/550px/img0076.jpg
https://www.dermis.net/bilder/CD036/550px/img0077.jpg
https://www.dermis.net/bilder/CD015/550px/img0039.jpg
https://www.dermis.net/bilder/CD004/550px/img0022.jpg
https://www.dermis.net/bilder/CD015/550px/img0040.jpg
https://www.dermis.net/bilder/CD044/550px/img0054.jpg
https://www.dermis.net/bilder/CD027/550px/img0007.jpg
https://www.dermis.net/bilder/CD024/550px/img0053.jpg
https://www.dermis.net/bilder/CD024/550px/img0054.jpg
https://www.dermis.net/bilder/CD024/550px/img0055.jpg
https://www.dermis.net/bilder/CD024/550px/img0057.jpg
https://www.dermis.net/bilder/CD027/550px/img0009.jpg
https://www.dermis.net/bilder/CD007/550px/img0006.jpg
https://www.dermis.net/bilder/CD046/550px/img0076.jpg
https://www.dermis.net/bilder/CD034/550px/img0051.jpg
https://www.dermis.net/bilder/CD032/550px/img0034.jpg
https://www.dermis.net/bilder/CD032/550px/img0035.jpg
https://www.dermis.net/bilder/CD003/550px/img0036.jpg
https://www.dermis.net/bilder/CD054/550px/img0019.jpg
https://www.dermis.net/bilder/CD048/550px/img0085.jpg
https://www.dermis.net/bilder/CD032/550px/img0033.jpg
https://www.dermis.net/bilder/CD023/550px/img0005.jpg
https://www.dermis.net/bilder/CD023/550px/img0006.jpg
https://www.dermis.net/bilder/CD026/550px/img0095.jpg
https://www.dermis.net/bilder/CD026/550px/img0096.jpg
https://www.dermis.net/bilder/CD020/550px/img0081.jpg
https://www.dermis.net/bilder/CD020/550px/img0082.jpg
https://www.dermis.net/bilder/CD025/550px/img0024.jpg
https://www.dermis.net/bilder/CD008/550px/img0066.jpg
https://www.dermis.net/bilder/CD034/550px/img0041.jpg
https://www.dermis.net/bilder/CD047/550px/img0068.jpg
https://www.dermis.net/bilder/CD041/550px/img0016.jpg
https://www.dermis.net/bilder/CD039/550px/img0066.jpg
https://www.dermis.net/bilder/CD039/550px/img0067.jpg
https://www.dermis.net/bilder/CD039/550px/img0068.jpg
https://www.dermis.net/bilder/CD039/550px/img0069.jpg
https://www.dermis.net/bilder/CD039/550px/img0070.jpg

https://www.dermis.net/bilder/CD034/550px/img0042.jpg
https://www.dermis.net/bilder/CD050/550px/img0098.jpg
https://www.dermis.net/bilder/CD050/550px/img0099.jpg
https://www.dermis.net/bilder/CD047/550px/img0067.jpg
https://www.dermis.net/bilder/CD037/550px/img0007.jpg
https://www.dermis.net/bilder/CD007/550px/img0007.jpg
https://www.dermis.net/bilder/CD020/550px/img0046.jpg
https://www.dermis.net/bilder/CD020/550px/img0050.jpg
https://www.dermis.net/bilder/CD020/550px/img0049.jpg
https://www.dermis.net/bilder/CD047/550px/img0100.jpg
https://www.dermis.net/bilder/CD032/550px/img0081.jpg
https://www.dermis.net/bilder/CD032/550px/img0082.jpg
https://www.dermis.net/bilder/CD040/550px/img0038.jpg
https://www.dermis.net/bilder/CD008/550px/img0046.jpg
https://www.dermis.net/bilder/CD006/550px/img0028.jpg
https://www.dermis.net/bilder/CD058/550px/img0081.jpg
https://www.dermis.net/bilder/CD024/550px/img0031.jpg
https://www.dermis.net/bilder/CD014/550px/img0015.jpg
https://www.dermis.net/bilder/CD027/550px/img0059.jpg
https://www.dermis.net/bilder/CD014/550px/img0012.jpg
https://www.dermis.net/bilder/CD044/550px/img0066.jpg
https://www.dermis.net/bilder/CD015/550px/img0005.jpg
https://www.dermis.net/bilder/CD049/550px/img0103.jpg
https://www.dermis.net/bilder/CD049/550px/img0104.jpg
https://www.dermis.net/bilder/CD005/550px/img0038.jpg
https://www.dermis.net/bilder/CD043/550px/img0041.jpg
https://www.dermis.net/bilder/CD044/550px/img0059.jpg
https://www.dermis.net/bilder/CD017/550px/img0091.jpg
https://www.dermis.net/bilder/CD005/550px/img0089.jpg
https://www.dermis.net/bilder/CD007/550px/img0047.jpg
https://www.dermis.net/bilder/CD008/550px/img0035.jpg
https://www.dermis.net/bilder/CD035/550px/img0019.jpg
https://www.dermis.net/bilder/CD018/550px/img0037.jpg
https://www.dermis.net/bilder/CD047/550px/img0060.jpg
https://www.dermis.net/bilder/CD047/550px/img0061.jpg
https://www.dermis.net/bilder/CD007/550px/img0005.jpg
https://www.dermis.net/bilder/CD047/550px/img0058.jpg
https://www.dermis.net/bilder/CD058/550px/img0003.jpg
https://www.dermis.net/bilder/CD058/550px/img0004.jpg
https://www.dermis.net/bilder/CD035/550px/img0060.jpg
https://www.dermis.net/bilder/CD035/550px/img0061.jpg
https://www.dermis.net/bilder/CD036/550px/img0066.jpg
https://www.dermis.net/bilder/CD020/550px/img0095.jpg
https://www.dermis.net/bilder/CD039/550px/img0001.jpg
https://www.dermis.net/bilder/CD058/550px/img0054.jpg
https://www.dermis.net/bilder/CD052/550px/img0032.jpg
https://www.dermis.net/bilder/CD052/550px/img0022.jpg
https://www.dermis.net/bilder/CD034/550px/img0078.jpg

https://www.dermis.net/bilder/CD026/550px/img0063.jpg
https://www.dermis.net/bilder/CD026/550px/img0064.jpg
https://www.dermis.net/bilder/CD026/550px/img0043.jpg
https://www.dermis.net/bilder/CD032/550px/img0070.jpg
https://www.dermis.net/bilder/CD018/550px/img0093.jpg
https://www.dermis.net/bilder/CD050/550px/img0037.jpg
https://www.dermis.net/bilder/CD050/550px/img0044.jpg
https://www.dermis.net/bilder/CD044/550px/img0062.jpg
https://www.dermis.net/bilder/CD049/550px/img0067.jpg
https://www.dermis.net/bilder/CD049/550px/img0068.jpg
https://www.dermis.net/bilder/CD003/550px/img0021.jpg
https://www.dermis.net/bilder/CD037/550px/img0024.jpg
https://www.dermis.net/bilder/CD098/550px/img0063.jpg
https://www.dermis.net/bilder/CD002/550px/img0064.jpg
https://www.dermis.net/bilder/CD016/550px/img0019.jpg
https://www.dermis.net/bilder/CD036/550px/img0050.jpg
https://www.dermis.net/bilder/CD212/550px/img0119.jpg
https://www.dermis.net/bilder/CD212/550px/img0019.jpg
https://www.dermis.net/bilder/CD054/550px/img0042.jpg
https://www.dermis.net/bilder/CD054/550px/img0043.jpg
https://www.dermis.net/bilder/CD033/550px/img0038.jpg
https://www.dermis.net/bilder/CD041/550px/img0064.jpg
https://www.dermis.net/bilder/CD042/550px/img0073.jpg
https://www.dermis.net/bilder/CD195/550px/img0054.jpg
https://www.dermis.net/bilder/CD011/550px/img0004.jpg
https://www.dermis.net/bilder/CD011/550px/img0005.jpg
https://www.dermis.net/bilder/CD012/550px/img0024.jpg
https://www.dermis.net/bilder/CD210/550px/img0026.jpg
https://www.dermis.net/bilder/CD182/550px/img0013.jpg
https://www.dermis.net/bilder/CD182/550px/img0012.jpg
https://www.dermis.net/bilder/CD192/550px/img0095.jpg
https://www.dermis.net/bilder/CD055/550px/img0050.jpg
https://www.dermis.net/bilder/CD190/550px/img0043.jpg
https://www.dermis.net/bilder/CD202/550px/img0016.jpg
https://www.dermis.net/bilder/CD192/550px/img0010.jpg
https://www.dermis.net/bilder/CD210/550px/img0120.jpg
https://www.dermis.net/bilder/CD193/550px/img0010.jpg
https://www.dermis.net/bilder/CD198/550px/img0180.jpg
https://www.dermis.net/bilder/CD198/550px/img0181.jpg
https://www.dermis.net/bilder/CD041/550px/img0055.jpg
https://www.dermis.net/bilder/CD012/550px/img0078.jpg
https://www.dermis.net/bilder/CD184/550px/img0080.jpg
https://www.dermis.net/bilder/CD194/550px/img0157.jpg
https://www.dermis.net/bilder/CD194/550px/img0158.jpg
https://www.dermis.net/bilder/CD194/550px/img0159.jpg
https://www.dermis.net/bilder/CD194/550px/img0160.jpg
https://www.dermis.net/bilder/CD025/550px/img0023.jpg
https://www.dermis.net/bilder/CD017/550px/img0040.jpg

https://www.dermis.net/bilder/CD178/550px/img0062.jpg
https://www.dermis.net/bilder/CD035/550px/img0076.jpg
https://www.dermis.net/bilder/CD027/550px/img0017.jpg
https://www.dermis.net/bilder/CD007/550px/img0019.jpg
https://www.dermis.net/bilder/CD185/550px/img0012.jpg
https://www.dermis.net/bilder/CD185/550px/img0013.jpg
https://www.dermis.net/bilder/CD211/550px/img0066.jpg
https://www.dermis.net/bilder/CD027/550px/img0024.jpg
https://www.dermis.net/bilder/CD192/550px/img0016.jpg
https://www.dermis.net/bilder/CD055/550px/img0076.jpg
https://www.dermis.net/bilder/CD055/550px/img0077.jpg
https://www.dermis.net/bilder/CD185/550px/img0098.jpg
https://www.dermis.net/bilder/CD193/550px/img0011.jpg
https://www.dermis.net/bilder/CD196/550px/img0151.jpg
https://www.dermis.net/bilder/CD196/550px/img0152.jpg
https://www.dermis.net/bilder/CD210/550px/img0053.jpg
https://www.dermis.net/bilder/CD186/550px/img0022.jpg
https://www.dermis.net/bilder/CD195/550px/img0109.jpg
https://www.dermis.net/bilder/CD196/550px/img0126.jpg
https://www.dermis.net/bilder/CD193/550px/img0028.jpg
https://www.dermis.net/bilder/CD070/550px/img0009.jpg
https://www.dermis.net/bilder/CD003/550px/img0066.jpg
https://www.dermis.net/bilder/CD002/550px/img0054.jpg
https://www.dermis.net/bilder/CD185/550px/img0015.jpg
https://www.dermis.net/bilder/CD193/550px/img0026.jpg
https://www.dermis.net/bilder/CD184/550px/img0008.jpg
https://www.dermis.net/bilder/CD184/550px/img0095.jpg
https://www.dermis.net/bilder/CD051/550px/img0002.jpg
https://www.dermis.net/bilder/CD197/550px/img0056.jpg
https://www.dermis.net/bilder/CD197/550px/img0057.jpg
https://www.dermis.net/bilder/CD193/550px/img0022.jpg
https://www.dermis.net/bilder/CD043/550px/img0043.jpg
https://www.dermis.net/bilder/CD180/550px/img0053.jpg
https://www.dermis.net/bilder/CD194/550px/img0145.jpg
https://www.dermis.net/bilder/CD202/550px/img0027.jpg
https://www.dermis.net/bilder/CD199/550px/img0109.jpg
https://www.dermis.net/bilder/CD199/550px/img0110.jpg
https://www.dermis.net/bilder/CD193/550px/img0102.jpg
https://www.dermis.net/bilder/CD193/550px/img0103.jpg
https://www.dermis.net/bilder/CD191/550px/img0053.jpg
https://www.dermis.net/bilder/CD191/550px/img0054.jpg
https://www.dermis.net/bilder/CD191/550px/img0055.jpg
https://www.dermis.net/bilder/CD021/550px/img0053.jpg
https://www.dermis.net/bilder/CD192/550px/img0094.jpg
https://www.dermis.net/bilder/CD007/550px/img0112.jpg
https://www.dermis.net/bilder/CD055/550px/img0079.jpg
https://www.dermis.net/bilder/CD055/550px/img0080.jpg
https://www.dermis.net/bilder/CD187/550px/img0018.jpg

https://www.dermis.net/bilder/CD187/550px/img0019.jpg
https://www.dermis.net/bilder/CD187/550px/img0020.jpg
https://www.dermis.net/bilder/CD040/550px/img0025.jpg
https://www.dermis.net/bilder/CD182/550px/img0060.jpg
https://www.dermis.net/bilder/CD196/550px/img0091.jpg
https://www.dermis.net/bilder/CD195/550px/img0013.jpg
https://www.dermis.net/bilder/CD071/550px/img0002.jpg
https://www.dermis.net/bilder/CD035/550px/img0084.jpg
https://www.dermis.net/bilder/CD189/550px/img0031.jpg
https://www.dermis.net/bilder/CD189/550px/img0032.jpg
https://www.dermis.net/bilder/CD187/550px/img0089.jpg
https://www.dermis.net/bilder/CD187/550px/img0090.jpg
https://www.dermis.net/bilder/CD008/550px/img0042.jpg
https://www.dermis.net/bilder/CD193/550px/img0007.jpg
https://www.dermis.net/bilder/CD003/550px/img0080.jpg
https://www.dermis.net/bilder/CD033/550px/img0012.jpg
https://www.dermis.net/bilder/CD003/550px/img0048.jpg
https://www.dermis.net/bilder/CD025/550px/img0022.jpg
https://www.dermis.net/bilder/CD002/550px/img0057.jpg
https://www.dermis.net/bilder/CD021/550px/img0020.jpg
https://www.dermis.net/bilder/CD021/550px/img0021.jpg
https://www.dermis.net/bilder/CD021/550px/img0040.jpg
https://www.dermis.net/bilder/CD021/550px/img0047.jpg
https://www.dermis.net/bilder/CD194/550px/img0149.jpg
https://www.dermis.net/bilder/CD021/550px/img0003.jpg
https://www.dermis.net/bilder/CD191/550px/img0067.jpg
https://www.dermis.net/bilder/CD193/550px/img0140.jpg
https://www.dermis.net/bilder/CD002/550px/img0067.jpg
https://www.dermis.net/bilder/CD004/550px/img0073.jpg
https://www.dermis.net/bilder/CD008/550px/img0007.jpg
https://www.dermis.net/bilder/CD182/550px/img0037.jpg
https://www.dermis.net/bilder/CD182/550px/img0038.jpg
https://www.dermis.net/bilder/CD178/550px/img0069.jpg
https://www.dermis.net/bilder/CD191/550px/img0114.jpg
https://www.dermis.net/bilder/CD191/550px/img0115.jpg
https://www.dermis.net/bilder/CD191/550px/img0118.jpg
https://www.dermis.net/bilder/CD193/550px/img0118.jpg
https://www.dermis.net/bilder/CD193/550px/img0123.jpg
https://www.dermis.net/bilder/CD193/550px/img0124.jpg
https://www.dermis.net/bilder/CD199/550px/img0105.jpg
https://www.dermis.net/bilder/CD180/550px/img0054.jpg
https://www.dermis.net/bilder/CD195/550px/img0014.jpg
https://www.dermis.net/bilder/CD195/550px/img0015.jpg
https://www.dermis.net/bilder/CD210/550px/img0096.jpg
https://www.dermis.net/bilder/CD210/550px/img0097.jpg
https://www.dermis.net/bilder/CD186/550px/img0015.jpg
https://www.dermis.net/bilder/CD055/550px/img0067.jpg
https://www.dermis.net/bilder/CD033/550px/img0009.jpg

https://www.dermis.net/bilder/CD041/550px/img0042.jpg
https://www.dermis.net/bilder/CD040/550px/img0103.jpg
https://www.dermis.net/bilder/CD036/550px/img0101.jpg
https://www.dermis.net/bilder/CD052/550px/img0070.jpg
https://www.dermis.net/bilder/CD211/550px/img0017.jpg
https://www.dermis.net/bilder/CD199/550px/img0037.jpg
https://www.dermis.net/bilder/CD180/550px/img0047.jpg
https://www.dermis.net/bilder/CD040/550px/img0071.jpg
https://www.dermis.net/bilder/CD040/550px/img0072.jpg
https://www.dermis.net/bilder/CD210/550px/img0024.jpg
https://www.dermis.net/bilder/CD040/550px/img0075.jpg
https://www.dermis.net/bilder/CD185/550px/img0073.jpg
https://www.dermis.net/bilder/CD185/550px/img0074.jpg
https://www.dermis.net/bilder/CD185/550px/img0075.jpg
https://www.dermis.net/bilder/CD185/550px/img0076.jpg
https://www.dermis.net/bilder/CD201/550px/img0036.jpg
https://www.dermis.net/bilder/CD196/550px/img0022.jpg
https://www.dermis.net/bilder/CD199/550px/img0108.jpg
https://www.dermis.net/bilder/CD180/550px/img0058.jpg
https://www.dermis.net/bilder/CD180/550px/img0057.jpg
https://www.dermis.net/bilder/CD212/550px/img0057.jpg
https://www.dermis.net/bilder/CD044/550px/img0080.jpg
https://www.dermis.net/bilder/CD196/550px/img0092.jpg
https://www.dermis.net/bilder/CD196/550px/img0093.jpg
https://www.dermis.net/bilder/CD196/550px/img0149.jpg
https://www.dermis.net/bilder/CD017/550px/img0096.jpg
https://www.dermis.net/bilder/CD194/550px/img0073.jpg
https://www.dermis.net/bilder/CD194/550px/img0074.jpg
https://www.dermis.net/bilder/CD041/550px/img0054.jpg
https://www.dermis.net/bilder/CD193/550px/img0082.jpg
https://www.dermis.net/bilder/CD189/550px/img0089.jpg
https://www.dermis.net/bilder/CD196/550px/img0063.jpg
https://www.dermis.net/bilder/CD189/550px/img0098.jpg
https://www.dermis.net/bilder/CD189/550px/img0099.jpg
https://www.dermis.net/bilder/CD199/550px/img0111.jpg
https://www.dermis.net/bilder/CD183/550px/img0077.jpg
https://www.dermis.net/bilder/CD193/550px/img0072.jpg
https://www.dermis.net/bilder/CD036/550px/img0070.jpg
https://www.dermis.net/bilder/CD002/550px/img0085.jpg
https://www.dermis.net/bilder/CD033/550px/img0026.jpg
https://www.dermis.net/bilder/CD181/550px/img0066.jpg
https://www.dermis.net/bilder/CD036/550px/img0048.jpg
https://www.dermis.net/bilder/CD191/550px/img0015.jpg
https://www.dermis.net/bilder/CD184/550px/img0094.jpg
https://www.dermis.net/bilder/CD183/550px/img0047.jpg
https://www.dermis.net/bilder/CD180/550px/img0046.jpg
https://www.dermis.net/bilder/CD180/550px/img0077.jpg
https://www.dermis.net/bilder/CD180/550px/img0078.jpg

https://www.dermis.net/bilder/CD180/550px/img0076.jpg
https://www.dermis.net/bilder/CD212/550px/img0036.jpg
https://www.dermis.net/bilder/CD212/550px/img0037.jpg
https://www.dermis.net/bilder/CD055/550px/img0049.jpg
https://www.dermis.net/bilder/CD183/550px/img0089.jpg
https://www.dermis.net/bilder/CD013/550px/img0002.jpg
https://www.dermis.net/bilder/CD188/550px/img0006.jpg
https://www.dermis.net/bilder/CD188/550px/img0007.jpg
https://www.dermis.net/bilder/CD188/550px/img0008.jpg
https://www.dermis.net/bilder/CD021/550px/img0037.jpg
https://www.dermis.net/bilder/CD182/550px/img0036.jpg
https://www.dermis.net/bilder/CD182/550px/img0035.jpg
https://www.dermis.net/bilder/CD190/550px/img0057.jpg
https://www.dermis.net/bilder/CD193/550px/img0116.jpg
https://www.dermis.net/bilder/CD181/550px/img0024.jpg
https://www.dermis.net/bilder/CD181/550px/img0023.jpg
https://www.dermis.net/bilder/CD186/550px/img0020.jpg
https://www.dermis.net/bilder/CD186/550px/img0021.jpg
https://www.dermis.net/bilder/CD041/550px/img0068.jpg
https://www.dermis.net/bilder/CD016/550px/img0021.jpg
https://www.dermis.net/bilder/CD211/550px/img0082.jpg
https://www.dermis.net/bilder/CD192/550px/img0030.jpg
https://www.dermis.net/bilder/CD199/550px/img0041.jpg
https://www.dermis.net/bilder/CD199/550px/img0043.jpg
https://www.dermis.net/bilder/CD184/550px/img0085.jpg
https://www.dermis.net/bilder/CD184/550px/img0063.jpg
https://www.dermis.net/bilder/CD005/550px/img0002.jpg
https://www.dermis.net/bilder/CD193/550px/img0023.jpg
https://www.dermis.net/bilder/CD196/550px/img0023.jpg
https://www.dermis.net/bilder/CD199/550px/img0137.jpg
https://www.dermis.net/bilder/CD199/550px/img0138.jpg
https://www.dermis.net/bilder/CD008/550px/img0077.jpg
https://www.dermis.net/bilder/CD179/550px/img0021.jpg
https://www.dermis.net/bilder/CD179/550px/img0022.jpg
https://www.dermis.net/bilder/CD191/550px/img0096.jpg
https://www.dermis.net/bilder/CD017/550px/img0101.jpg
https://www.dermis.net/bilder/CD198/550px/img0177.jpg
https://www.dermis.net/bilder/CD008/550px/img0036.jpg
https://www.dermis.net/bilder/CD017/550px/img0085.jpg
https://www.dermis.net/bilder/CD211/550px/img0081.jpg
https://www.dermis.net/bilder/CD192/550px/img0075.jpg
https://www.dermis.net/bilder/CD181/550px/img0082.jpg
https://www.dermis.net/bilder/CD008/550px/img0080.jpg
https://www.dermis.net/bilder/CD002/550px/img0086.jpg
https://www.dermis.net/bilder/CD071/550px/img0037.jpg
https://www.dermis.net/bilder/CD199/550px/img0132.jpg
https://www.dermis.net/bilder/CD041/550px/img0041.jpg
https://www.dermis.net/bilder/CD199/550px/img0133.jpg

https://www.dermis.net/bilder/CD199/550px/img0042.jpg
https://www.dermis.net/bilder/CD021/550px/img0042.jpg
https://www.dermis.net/bilder/CD191/550px/img0100.jpg
https://www.dermis.net/bilder/CD191/550px/img0101.jpg
https://www.dermis.net/bilder/CD193/550px/img0107.jpg
https://www.dermis.net/bilder/CD211/550px/img0026.jpg
https://www.dermis.net/bilder/CD193/550px/img0030.jpg
https://www.dermis.net/bilder/CD193/550px/img0031.jpg
https://www.dermis.net/bilder/CD055/550px/img0095.jpg
https://www.dermis.net/bilder/CD198/550px/img0082.jpg
https://www.dermis.net/bilder/CD195/550px/img0022.jpg
https://www.dermis.net/bilder/CD210/550px/img0052.jpg
https://www.dermis.net/bilder/CD196/550px/img0122.jpg
https://www.dermis.net/bilder/CD198/550px/img0073.jpg
https://www.dermis.net/bilder/CD199/550px/img0134.jpg
https://www.dermis.net/bilder/CD184/550px/img0030.jpg
https://www.dermis.net/bilder/CD184/550px/img0031.jpg
https://www.dermis.net/bilder/CD210/550px/img0121.jpg
https://www.dermis.net/bilder/CD041/550px/img0070.jpg
https://www.dermis.net/bilder/CD189/550px/img0047.jpg
https://www.dermis.net/bilder/CD210/550px/img0123.jpg
https://www.dermis.net/bilder/CD054/550px/img0044.jpg
https://www.dermis.net/bilder/CD196/550px/img0144.jpg
https://www.dermis.net/bilder/CD041/550px/img0045.jpg
https://www.dermis.net/bilder/CD193/550px/img0193.jpg
https://www.dermis.net/bilder/CD012/550px/img0089.jpg
https://www.dermis.net/bilder/CD012/550px/img0090.jpg
https://www.dermis.net/bilder/CD198/550px/img0148.jpg
https://www.dermis.net/bilder/CD198/550px/img0150.jpg
https://www.dermis.net/bilder/CD001/550px/img0014.jpg
https://www.dermis.net/bilder/CD199/550px/img0078.jpg
https://www.dermis.net/bilder/CD199/550px/img0079.jpg
https://www.dermis.net/bilder/CD199/550px/img0080.jpg
https://www.dermis.net/bilder/CD040/550px/img0036.jpg
https://www.dermis.net/bilder/CD055/550px/img0083.jpg
https://www.dermis.net/bilder/CD195/550px/img0051.jpg
https://www.dermis.net/bilder/CD055/550px/img0084.jpg
https://www.dermis.net/bilder/CD001/550px/img0061.jpg
https://www.dermis.net/bilder/CD014/550px/img0073.jpg
https://www.dermis.net/bilder/CD199/550px/img0130.jpg
https://www.dermis.net/bilder/CD199/550px/img0131.jpg
https://www.dermis.net/bilder/CD210/550px/img0058.jpg
https://www.dermis.net/bilder/CD210/550px/img0059.jpg
https://www.dermis.net/bilder/CD179/550px/img0024.jpg
https://www.dermis.net/bilder/CD195/550px/img0090.jpg
https://www.dermis.net/bilder/CD055/550px/img0081.jpg
https://www.dermis.net/bilder/CD210/550px/img0054.jpg
https://www.dermis.net/bilder/CD182/550px/img0031.jpg

https://www.dermis.net/bilder/CD035/550px/img0069.jpg
https://www.dermis.net/bilder/CD199/550px/img0066.jpg
https://www.dermis.net/bilder/CD199/550px/img0067.jpg
https://www.dermis.net/bilder/CD199/550px/img0068.jpg
https://www.dermis.net/bilder/CD181/550px/img0041.jpg
https://www.dermis.net/bilder/CD181/550px/img0042.jpg
https://www.dermis.net/bilder/CD184/550px/img0096.jpg
https://www.dermis.net/bilder/CD040/550px/img0076.jpg
https://www.dermis.net/bilder/CD014/550px/img0027.jpg
https://www.dermis.net/bilder/CD054/550px/img0017.jpg
https://www.dermis.net/bilder/CD192/550px/img0068.jpg
https://www.dermis.net/bilder/CD196/550px/img0150.jpg
https://www.dermis.net/bilder/CD026/550px/img0084.jpg
https://www.dermis.net/bilder/CD188/550px/img0016.jpg
https://www.dermis.net/bilder/CD188/550px/img0017.jpg
https://www.dermis.net/bilder/CD188/550px/img0018.jpg
https://www.dermis.net/bilder/CD187/550px/img0048.jpg
https://www.dermis.net/bilder/CD187/550px/img0049.jpg
https://www.dermis.net/bilder/CD187/550px/img0050.jpg
https://www.dermis.net/bilder/CD187/550px/img0051.jpg
https://www.dermis.net/bilder/CD187/550px/img0052.jpg
https://www.dermis.net/bilder/CD191/550px/img0002.jpg
https://www.dermis.net/bilder/CD021/550px/img0090.jpg
https://www.dermis.net/bilder/CD055/550px/img0014.jpg
https://www.dermis.net/bilder/CD181/550px/img0021.jpg
https://www.dermis.net/bilder/CD181/550px/img0018.jpg
https://www.dermis.net/bilder/CD056/550px/img0023.jpg
https://www.dermis.net/bilder/CD185/550px/img0097.jpg
https://www.dermis.net/bilder/CD187/550px/img0047.jpg
https://www.dermis.net/bilder/CD212/550px/img0047.jpg
https://www.dermis.net/bilder/CD008/550px/img0055.jpg
https://www.dermis.net/bilder/CD181/550px/img0067.jpg
https://www.dermis.net/bilder/CD185/550px/img0010.jpg
https://www.dermis.net/bilder/CD193/550px/img0027.jpg
https://www.dermis.net/bilder/CD212/550px/img0042.jpg
https://www.dermis.net/bilder/CD212/550px/img0129.jpg
https://www.dermis.net/bilder/CD201/550px/img0023.jpg
https://www.dermis.net/bilder/CD199/550px/img0039.jpg
https://www.dermis.net/bilder/CD199/550px/img0040.jpg
https://www.dermis.net/bilder/CD182/550px/img0043.jpg
https://www.dermis.net/bilder/CD184/550px/img0062.jpg
https://www.dermis.net/bilder/CD194/550px/img0146.jpg
https://www.dermis.net/bilder/CD194/550px/img0147.jpg
https://www.dermis.net/bilder/CD193/550px/img0100.jpg
https://www.dermis.net/bilder/CD191/550px/img0052.jpg
https://www.dermis.net/bilder/CD055/550px/img0046.jpg
https://www.dermis.net/bilder/CD183/550px/img0091.jpg
https://www.dermis.net/bilder/CD196/550px/img0090.jpg

https://www.dermis.net/bilder/CD199/550px/img0002.jpg
https://www.dermis.net/bilder/CD199/550px/img0003.jpg
https://www.dermis.net/bilder/CD187/550px/img0091.jpg
https://www.dermis.net/bilder/CD181/550px/img0080.jpg
https://www.dermis.net/bilder/CD211/550px/img0045.jpg
https://www.dermis.net/bilder/CD012/550px/img0080.jpg
https://www.dermis.net/bilder/CD055/550px/img0048.jpg
https://www.dermis.net/bilder/CD190/550px/img0029.jpg
https://www.dermis.net/bilder/CD190/550px/img0030.jpg
https://www.dermis.net/bilder/CD210/550px/img0065.jpg
https://www.dermis.net/bilder/CD210/550px/img0066.jpg
https://www.dermis.net/bilder/CD017/550px/img0089.jpg
https://www.dermis.net/bilder/CD180/550px/img0056.jpg
https://www.dermis.net/bilder/CD056/550px/img0024.jpg
https://www.dermis.net/bilder/CD033/550px/img0032.jpg
https://www.dermis.net/bilder/CD025/550px/img0005.jpg
https://www.dermis.net/bilder/CD041/550px/img0052.jpg
https://www.dermis.net/bilder/CD007/550px/img0111.jpg
https://www.dermis.net/bilder/CD193/550px/img0196.jpg
https://www.dermis.net/bilder/CD183/550px/img0078.jpg
https://www.dermis.net/bilder/CD032/550px/img0047.jpg
https://www.dermis.net/bilder/CD183/550px/img0048.jpg
https://www.dermis.net/bilder/CD183/550px/img0049.jpg
https://www.dermis.net/bilder/CD196/550px/img0066.jpg
https://www.dermis.net/bilder/CD196/550px/img0067.jpg
https://www.dermis.net/bilder/CD196/550px/img0068.jpg
https://www.dermis.net/bilder/CD196/550px/img0024.jpg
https://www.dermis.net/bilder/CD041/550px/img0085.jpg
https://www.dermis.net/bilder/CD212/550px/img0074.jpg
https://www.dermis.net/bilder/CD007/550px/img0115.jpg
https://www.dermis.net/bilder/CD196/550px/img0059.jpg
https://www.dermis.net/bilder/CD196/550px/img0060.jpg
https://www.dermis.net/bilder/CD195/550px/img0110.jpg
https://www.dermis.net/bilder/CD033/550px/img0027.jpg
https://www.dermis.net/bilder/CD008/550px/img0006.jpg
https://www.dermis.net/bilder/CD195/550px/img0108.jpg
https://www.dermis.net/bilder/CD018/550px/img0042.jpg
https://www.dermis.net/bilder/CD040/550px/img0067.jpg
https://www.dermis.net/bilder/CD001/550px/img0052.jpg
https://www.dermis.net/bilder/CD071/550px/img0029.jpg
https://www.dermis.net/bilder/CD056/550px/img0039.jpg
https://www.dermis.net/bilder/CD069/550px/img0107.jpg
https://www.dermis.net/bilder/CD054/550px/img0002.jpg
https://www.dermis.net/bilder/CD003/550px/img0043.jpg
https://www.dermis.net/bilder/CD021/550px/img0068.jpg
https://www.dermis.net/bilder/CD001/550px/img0043.jpg
https://www.dermis.net/bilder/CD005/550px/img0102.jpg
https://www.dermis.net/bilder/CD069/550px/img0102.jpg

https://www.dermis.net/bilder/CD069/550px/img0103.jpg
https://www.dermis.net/bilder/CD003/550px/img0014.jpg
https://www.dermis.net/bilder/CD032/550px/img0072.jpg
https://www.dermis.net/bilder/CD017/550px/img0042.jpg
https://www.dermis.net/bilder/CD003/550px/img0075.jpg
https://www.dermis.net/bilder/CD212/550px/img0045.jpg
https://www.dermis.net/bilder/CD193/550px/img0024.jpg
https://www.dermis.net/bilder/CD210/550px/img0025.jpg
https://www.dermis.net/bilder/CD189/550px/img0008.jpg
https://www.dermis.net/bilder/CD054/550px/img0007.jpg
https://www.dermis.net/bilder/CD194/550px/img0099.jpg
https://www.dermis.net/bilder/CD004/550px/img0060.jpg
https://www.dermis.net/bilder/CD042/550px/img0067.jpg
https://www.dermis.net/bilder/CD199/550px/img0038.jpg
https://www.dermis.net/bilder/CD211/550px/img0084.jpg
https://www.dermis.net/bilder/CD022/550px/img0001.jpg
https://www.dermis.net/bilder/CD003/550px/img0083.jpg
https://www.dermis.net/bilder/CD040/550px/img0033.jpg
https://www.dermis.net/bilder/CD044/550px/img0058.jpg
https://www.dermis.net/bilder/CD017/550px/img0100.jpg
https://www.dermis.net/bilder/CD054/550px/img0066.jpg
https://www.dermis.net/bilder/CD014/550px/img0024.jpg
https://www.dermis.net/bilder/CD197/550px/img0008.jpg
https://www.dermis.net/bilder/CD197/550px/img0009.jpg
https://www.dermis.net/bilder/CD198/550px/img0173.jpg
https://www.dermis.net/bilder/CD211/550px/img0078.jpg
https://www.dermis.net/bilder/CD210/550px/img0122.jpg
https://www.dermis.net/bilder/CD014/550px/img0036.jpg
https://www.dermis.net/bilder/CD002/550px/img0061.jpg
https://www.dermis.net/bilder/CD004/550px/img0053.jpg
https://www.dermis.net/bilder/CD048/550px/img0086.jpg
https://www.dermis.net/bilder/CD002/550px/img0078.jpg
https://www.dermis.net/bilder/CD054/550px/img0067.jpg
https://www.dermis.net/bilder/CD041/550px/img0083.jpg
https://www.dermis.net/bilder/CD040/550px/img0056.jpg
https://www.dermis.net/bilder/CD071/550px/img0021.jpg
https://www.dermis.net/bilder/CD050/550px/img0096.jpg
https://www.dermis.net/bilder/CD005/550px/img0097.jpg
https://www.dermis.net/bilder/CD069/550px/img0093.jpg
https://www.dermis.net/bilder/CD025/550px/img0045.jpg
https://www.dermis.net/bilder/CD070/550px/img0096.jpg
https://www.dermis.net/bilder/CD071/550px/img0015.jpg
https://www.dermis.net/bilder/CD070/550px/img0046.jpg
https://www.dermis.net/bilder/CD070/550px/img0047.jpg
https://www.dermis.net/bilder/CD069/550px/img0111.jpg
https://www.dermis.net/bilder/CD025/550px/img0046.jpg
https://www.dermis.net/bilder/CD070/550px/img0045.jpg
https://www.dermis.net/bilder/CD021/550px/img0089.jpg

https://www.dermis.net/bilder/CD040/550px/img0018.jpg
https://www.dermis.net/bilder/CD040/550px/img0019.jpg
https://www.dermis.net/bilder/CD005/550px/img0048.jpg
https://www.dermis.net/bilder/CD004/550px/img0030.jpg
https://www.dermis.net/bilder/CD006/550px/img0037.jpg
https://www.dermis.net/bilder/CD056/550px/img0051.jpg
https://www.dermis.net/bilder/CD026/550px/img0069.jpg
https://www.dermis.net/bilder/CD026/550px/img0068.jpg
https://www.dermis.net/bilder/CD026/550px/img0070.jpg
https://www.dermis.net/bilder/CD054/550px/img0103.jpg
https://www.dermis.net/bilder/CD054/550px/img0104.jpg
https://www.dermis.net/bilder/CD001/550px/img0035.jpg
https://www.dermis.net/bilder/CD020/550px/img0037.jpg
https://www.dermis.net/bilder/CD020/550px/img0039.jpg
https://www.dermis.net/bilder/CD054/550px/img0069.jpg
https://www.dermis.net/bilder/CD054/550px/img0070.jpg
https://www.dermis.net/bilder/CD028/550px/img0003.jpg
https://www.dermis.net/bilder/CD028/550px/img0004.jpg
https://www.dermis.net/bilder/CD014/550px/img0088.jpg
https://www.dermis.net/bilder/CD014/550px/img0087.jpg
https://www.dermis.net/bilder/CD018/550px/img0066.jpg
https://www.dermis.net/bilder/CD070/550px/img0084.jpg
https://www.dermis.net/bilder/CD028/550px/img0010.jpg
https://www.dermis.net/bilder/CD028/550px/img0009.jpg
https://www.dermis.net/bilder/CD004/550px/img0049.jpg
https://www.dermis.net/bilder/CD070/550px/img0042.jpg
https://www.dermis.net/bilder/CD070/550px/img0043.jpg
https://www.dermis.net/bilder/CD050/550px/img0029.jpg
https://www.dermis.net/bilder/CD050/550px/img0035.jpg
https://www.dermis.net/bilder/CD050/550px/img0036.jpg
https://www.dermis.net/bilder/CD035/550px/img0025.jpg
https://www.dermis.net/bilder/CD028/550px/img0011.jpg
https://www.dermis.net/bilder/CD047/550px/img0017.jpg
https://www.dermis.net/bilder/CD001/550px/img0026.jpg
https://www.dermis.net/bilder/CD056/550px/img0058.jpg
https://www.dermis.net/bilder/CD071/550px/img0011.jpg
https://www.dermis.net/bilder/CD057/550px/img0009.jpg
https://www.dermis.net/bilder/CD057/550px/img0010.jpg
https://www.dermis.net/bilder/CD055/550px/img0096.jpg
https://www.dermis.net/bilder/CD016/550px/img0003.jpg
https://www.dermis.net/bilder/CD006/550px/img0006.jpg
https://www.dermis.net/bilder/CD033/550px/img0062.jpg
https://www.dermis.net/bilder/CD071/550px/img0069.jpg
https://www.dermis.net/bilder/CD016/550px/img0027.jpg
https://www.dermis.net/bilder/CD036/550px/img0059.jpg
https://www.dermis.net/bilder/CD051/550px/img0032.jpg
https://www.dermis.net/bilder/CD005/550px/img0084.jpg
https://www.dermis.net/bilder/CD015/550px/img0020.jpg

https://www.dermis.net/bilder/CD070/550px/img0034.jpg
https://www.dermis.net/bilder/CD008/550px/img0040.jpg
https://www.dermis.net/bilder/CD035/550px/img0011.jpg
https://www.dermis.net/bilder/CD070/550px/img0100.jpg
https://www.dermis.net/bilder/CD070/550px/img0099.jpg
https://www.dermis.net/bilder/CD006/550px/img0074.jpg
https://www.dermis.net/bilder/CD073/550px/img0003.jpg
https://www.dermis.net/bilder/CD055/550px/img0092.jpg
https://www.dermis.net/bilder/CD055/550px/img0093.jpg
https://www.dermis.net/bilder/CD016/550px/img0035.jpg
https://www.dermis.net/bilder/CD026/550px/img0021.jpg
https://www.dermis.net/bilder/CD026/550px/img0022.jpg
https://www.dermis.net/bilder/CD001/550px/img0049.jpg
https://www.dermis.net/bilder/CD069/550px/img0099.jpg
https://www.dermis.net/bilder/CD006/550px/img0008.jpg
https://www.dermis.net/bilder/CD037/550px/img0039.jpg
https://www.dermis.net/bilder/CD069/550px/img0108.jpg
https://www.dermis.net/bilder/CD001/550px/img0092.jpg
https://www.dermis.net/bilder/CD069/550px/img0030.jpg
https://www.dermis.net/bilder/CD056/550px/img0055.jpg
https://www.dermis.net/bilder/CD006/550px/img0080.jpg
https://www.dermis.net/bilder/CD043/550px/img0010.jpg
https://www.dermis.net/bilder/CD025/550px/img0057.jpg
https://www.dermis.net/bilder/CD014/550px/img0005.jpg
https://www.dermis.net/bilder/CD002/550px/img0069.jpg
https://www.dermis.net/bilder/CD025/550px/img0058.jpg
https://www.dermis.net/bilder/CD052/550px/img0101.jpg
https://www.dermis.net/bilder/CD004/550px/img0038.jpg
https://www.dermis.net/bilder/CD056/550px/img0001.jpg
https://www.dermis.net/bilder/CD001/550px/img0088.jpg
https://www.dermis.net/bilder/CD001/550px/img0103.jpg
https://www.dermis.net/bilder/CD015/550px/img0025.jpg
https://www.dermis.net/bilder/CD018/550px/img0111.jpg
https://www.dermis.net/bilder/CD006/550px/img0082.jpg
https://www.dermis.net/bilder/CD018/550px/img0109.jpg
https://www.dermis.net/bilder/CD050/550px/img0065.jpg
https://www.dermis.net/bilder/CD020/550px/img0044.jpg
https://www.dermis.net/bilder/CD004/550px/img0095.jpg
https://www.dermis.net/bilder/CD073/550px/img0007.jpg
https://www.dermis.net/bilder/CD054/550px/img0003.jpg
https://www.dermis.net/bilder/CD052/550px/img0009.jpg
https://www.dermis.net/bilder/CD069/550px/img0042.jpg
https://www.dermis.net/bilder/CD069/550px/img0043.jpg
https://www.dermis.net/bilder/CD003/550px/img0084.jpg
https://www.dermis.net/bilder/CD069/550px/img0046.jpg
https://www.dermis.net/bilder/CD018/550px/img0108.jpg
https://www.dermis.net/bilder/CD069/550px/img0047.jpg
https://www.dermis.net/bilder/CD069/550px/img0048.jpg

https://www.dermis.net/bilder/CD018/550px/img0052.jpg
https://www.dermis.net/bilder/CD051/550px/img0072.jpg
https://www.dermis.net/bilder/CD015/550px/img0003.jpg
https://www.dermis.net/bilder/CD042/550px/img0038.jpg
https://www.dermis.net/bilder/CD020/550px/img0078.jpg
https://www.dermis.net/bilder/CD025/550px/img0053.jpg
https://www.dermis.net/bilder/CD025/550px/img0054.jpg
https://www.dermis.net/bilder/CD025/550px/img0055.jpg
https://www.dermis.net/bilder/CD025/550px/img0056.jpg
https://www.dermis.net/bilder/CD003/550px/img0054.jpg
https://www.dermis.net/bilder/CD005/550px/img0009.jpg
https://www.dermis.net/bilder/CD044/550px/img0046.jpg
https://www.dermis.net/bilder/CD046/550px/img0051.jpg
https://www.dermis.net/bilder/CD048/550px/img0071.jpg
https://www.dermis.net/bilder/CD054/550px/img0030.jpg
https://www.dermis.net/bilder/CD044/550px/img0040.jpg
https://www.dermis.net/bilder/CD048/550px/img0070.jpg
https://www.dermis.net/bilder/CD034/550px/img0092.jpg
https://www.dermis.net/bilder/CD008/550px/img0056.jpg
https://www.dermis.net/bilder/CD016/550px/img0002.jpg
https://www.dermis.net/bilder/CD043/550px/img0022.jpg
https://www.dermis.net/bilder/CD036/550px/img0064.jpg
https://www.dermis.net/bilder/CD006/550px/img0089.jpg
https://www.dermis.net/bilder/CD016/550px/img0054.jpg
https://www.dermis.net/bilder/CD001/550px/img0011.jpg
https://www.dermis.net/bilder/CD007/550px/img0042.jpg
https://www.dermis.net/bilder/CD052/550px/img0099.jpg
https://www.dermis.net/bilder/CD018/550px/img0104.jpg
https://www.dermis.net/bilder/CD004/550px/img0088.jpg
https://www.dermis.net/bilder/CD004/550px/img0046.jpg
https://www.dermis.net/bilder/CD003/550px/img0009.jpg
https://www.dermis.net/bilder/CD041/550px/img0025.jpg
https://www.dermis.net/bilder/CD001/550px/img0002.jpg
https://www.dermis.net/bilder/CD021/550px/img0074.jpg
https://www.dermis.net/bilder/CD004/550px/img0057.jpg
https://www.dermis.net/bilder/CD005/550px/img0037.jpg
https://www.dermis.net/bilder/CD028/550px/img0037.jpg
https://www.dermis.net/bilder/CD035/550px/img0057.jpg
https://www.dermis.net/bilder/CD015/550px/img0045.jpg
https://www.dermis.net/bilder/CD050/550px/img0071.jpg
https://www.dermis.net/bilder/CD071/550px/img0049.jpg
https://www.dermis.net/bilder/CD018/550px/img0081.jpg
https://www.dermis.net/bilder/CD005/550px/img0093.jpg
https://www.dermis.net/bilder/CD042/550px/img0003.jpg
https://www.dermis.net/bilder/CD005/550px/img0105.jpg
https://www.dermis.net/bilder/CD007/550px/img0052.jpg
https://www.dermis.net/bilder/CD020/550px/img0067.jpg
https://www.dermis.net/bilder/CD046/550px/img0037.jpg

https://www.dermis.net/bilder/CD046/550px/img0038.jpg
https://www.dermis.net/bilder/CD004/550px/img0083.jpg
https://www.dermis.net/bilder/CD004/550px/img0058.jpg
https://www.dermis.net/bilder/CD021/550px/img0094.jpg
https://www.dermis.net/bilder/CD004/550px/img0090.jpg
https://www.dermis.net/bilder/CD004/550px/img0092.jpg
https://www.dermis.net/bilder/CD004/550px/img0089.jpg
https://www.dermis.net/bilder/CD004/550px/img0041.jpg
https://www.dermis.net/bilder/CD020/550px/img0090.jpg
https://www.dermis.net/bilder/CD008/550px/img0068.jpg
https://www.dermis.net/bilder/CD049/550px/img0051.jpg
https://www.dermis.net/bilder/CD034/550px/img0004.jpg
https://www.dermis.net/bilder/CD005/550px/img0018.jpg
https://www.dermis.net/bilder/CD020/550px/img0010.jpg
https://www.dermis.net/bilder/CD034/550px/img0003.jpg
https://www.dermis.net/bilder/CD024/550px/img0085.jpg
https://www.dermis.net/bilder/CD024/550px/img0086.jpg
https://www.dermis.net/bilder/CD024/550px/img0092.jpg
https://www.dermis.net/bilder/CD024/550px/img0093.jpg
https://www.dermis.net/bilder/CD033/550px/img0057.jpg
https://www.dermis.net/bilder/CD069/550px/img0101.jpg
https://www.dermis.net/bilder/CD034/550px/img0073.jpg
https://www.dermis.net/bilder/CD006/550px/img0027.jpg
https://www.dermis.net/bilder/CD034/550px/img0072.jpg
https://www.dermis.net/bilder/CD017/550px/img0079.jpg
https://www.dermis.net/bilder/CD026/550px/img0038.jpg
https://www.dermis.net/bilder/CD023/550px/img0098.jpg
https://www.dermis.net/bilder/CD034/550px/img0099.jpg
https://www.dermis.net/bilder/CD052/550px/img0031.jpg
https://www.dermis.net/bilder/CD023/550px/img0096.jpg
https://www.dermis.net/bilder/CD034/550px/img0039.jpg
https://www.dermis.net/bilder/CD047/550px/img0027.jpg
https://www.dermis.net/bilder/CD047/550px/img0028.jpg
https://www.dermis.net/bilder/CD023/550px/img0100.jpg
https://www.dermis.net/bilder/CD034/550px/img0087.jpg
https://www.dermis.net/bilder/CD027/550px/img0075.jpg
https://www.dermis.net/bilder/CD044/550px/img0008.jpg
https://www.dermis.net/bilder/CD026/550px/img0046.jpg
https://www.dermis.net/bilder/CD003/550px/img0060.jpg
https://www.dermis.net/bilder/CD055/550px/img0004.jpg
https://www.dermis.net/bilder/CD026/550px/img0049.jpg
https://www.dermis.net/bilder/CD034/550px/img0088.jpg
https://www.dermis.net/bilder/CD034/550px/img0089.jpg
https://www.dermis.net/bilder/CD019/550px/img0010.jpg
https://www.dermis.net/bilder/CD033/550px/img0060.jpg
https://www.dermis.net/bilder/CD001/550px/img0036.jpg
https://www.dermis.net/bilder/CD039/550px/img0084.jpg
https://www.dermis.net/bilder/CD039/550px/img0085.jpg

https://www.dermis.net/bilder/CD039/550px/img0086.jpg
https://www.dermis.net/bilder/CD039/550px/img0087.jpg
https://www.dermis.net/bilder/CD039/550px/img0088.jpg
https://www.dermis.net/bilder/CD033/550px/img0061.jpg
https://www.dermis.net/bilder/CD001/550px/img0038.jpg
https://www.dermis.net/bilder/CD001/550px/img0039.jpg
https://www.dermis.net/bilder/CD039/550px/img0089.jpg
https://www.dermis.net/bilder/CD039/550px/img0090.jpg
https://www.dermis.net/bilder/CD005/550px/img0067.jpg
https://www.dermis.net/bilder/CD004/550px/img0034.jpg
https://www.dermis.net/bilder/CD033/550px/img0050.jpg
https://www.dermis.net/bilder/CD044/550px/img0027.jpg
https://www.dermis.net/bilder/CD044/550px/img0028.jpg
https://www.dermis.net/bilder/CD041/550px/img0014.jpg
https://www.dermis.net/bilder/CD006/550px/img0011.jpg
https://www.dermis.net/bilder/CD057/550px/img0037.jpg
https://www.dermis.net/bilder/CD057/550px/img0006.jpg
https://www.dermis.net/bilder/CD019/550px/img0003.jpg
https://www.dermis.net/bilder/CD034/550px/img0028.jpg
https://www.dermis.net/bilder/CD004/550px/img0075.jpg
https://www.dermis.net/bilder/CD023/550px/img0038.jpg
https://www.dermis.net/bilder/CD048/550px/img0094.jpg
https://www.dermis.net/bilder/CD048/550px/img0095.jpg
https://www.dermis.net/bilder/CD023/550px/img0040.jpg
https://www.dermis.net/bilder/CD018/550px/img0103.jpg
https://www.dermis.net/bilder/CD026/550px/img0092.jpg
https://www.dermis.net/bilder/CD026/550px/img0093.jpg
https://www.dermis.net/bilder/CD054/550px/img0074.jpg
https://www.dermis.net/bilder/CD016/550px/img0026.jpg
https://www.dermis.net/bilder/CD044/550px/img0005.jpg
https://www.dermis.net/bilder/CD014/550px/img0097.jpg
https://www.dermis.net/bilder/CD016/550px/img0030.jpg
https://www.dermis.net/bilder/CD035/550px/img0007.jpg
https://www.dermis.net/bilder/CD046/550px/img0003.jpg
https://www.dermis.net/bilder/CD035/550px/img0005.jpg
https://www.dermis.net/bilder/CD035/550px/img0006.jpg
https://www.dermis.net/bilder/CD037/550px/img0059.jpg
https://www.dermis.net/bilder/CD037/550px/img0058.jpg
https://www.dermis.net/bilder/CD046/550px/img0001.jpg
https://www.dermis.net/bilder/CD046/550px/img0002.jpg
https://www.dermis.net/bilder/CD046/550px/img0004.jpg
https://www.dermis.net/bilder/CD003/550px/img0055.jpg
https://www.dermis.net/bilder/CD035/550px/img0001.jpg
https://www.dermis.net/bilder/CD003/550px/img0006.jpg
https://www.dermis.net/bilder/CD044/550px/img0015.jpg
https://www.dermis.net/bilder/CD050/550px/img0021.jpg
https://www.dermis.net/bilder/CD021/550px/img0091.jpg
https://www.dermis.net/bilder/CD037/550px/img0014.jpg

https://www.dermis.net/bilder/CD037/550px/img0015.jpg
https://www.dermis.net/bilder/CD027/550px/img0090.jpg
https://www.dermis.net/bilder/CD027/550px/img0091.jpg
https://www.dermis.net/bilder/CD020/550px/img0085.jpg
https://www.dermis.net/bilder/CD037/550px/img0013.jpg
https://www.dermis.net/bilder/CD027/550px/img0089.jpg
https://www.dermis.net/bilder/CD001/550px/img0010.jpg
https://www.dermis.net/bilder/CD005/550px/img0069.jpg
https://www.dermis.net/bilder/CD037/550px/img0046.jpg
https://www.dermis.net/bilder/CD027/550px/img0056.jpg
https://www.dermis.net/bilder/CD055/550px/img0001.jpg
https://www.dermis.net/bilder/CD044/550px/img0049.jpg
https://www.dermis.net/bilder/CD004/550px/img0047.jpg
https://www.dermis.net/bilder/CD004/550px/img0087.jpg
https://www.dermis.net/bilder/CD027/550px/img0055.jpg
https://www.dermis.net/bilder/CD004/550px/img0037.jpg
https://www.dermis.net/bilder/CD014/550px/img0084.jpg
https://www.dermis.net/bilder/CD047/550px/img0033.jpg
https://www.dermis.net/bilder/CD047/550px/img0038.jpg
https://www.dermis.net/bilder/CD007/550px/img0035.jpg
https://www.dermis.net/bilder/CD014/550px/img0001.jpg
https://www.dermis.net/bilder/CD056/550px/img0022.jpg
https://www.dermis.net/bilder/CD007/550px/img0004.jpg
https://www.dermis.net/bilder/CD004/550px/img0035.jpg
https://www.dermis.net/bilder/CD007/550px/img0023.jpg
https://www.dermis.net/bilder/CD025/550px/img0038.jpg
https://www.dermis.net/bilder/CD034/550px/img0008.jpg
https://www.dermis.net/bilder/CD034/550px/img0010.jpg
https://www.dermis.net/bilder/CD034/550px/img0011.jpg
https://www.dermis.net/bilder/CD034/550px/img0012.jpg
https://www.dermis.net/bilder/CD034/550px/img0013.jpg
https://www.dermis.net/bilder/CD027/550px/img0070.jpg
https://www.dermis.net/bilder/CD057/550px/img0038.jpg
https://www.dermis.net/bilder/CD057/550px/img0039.jpg
https://www.dermis.net/bilder/CD005/550px/img0062.jpg
https://www.dermis.net/bilder/CD034/550px/img0007.jpg
https://www.dermis.net/bilder/CD025/550px/img0039.jpg
https://www.dermis.net/bilder/CD006/550px/img0047.jpg
https://www.dermis.net/bilder/CD055/550px/img0028.jpg
https://www.dermis.net/bilder/CD055/550px/img0034.jpg
https://www.dermis.net/bilder/CD044/550px/img0025.jpg
https://www.dermis.net/bilder/CD044/550px/img0026.jpg
https://www.dermis.net/bilder/CD056/550px/img0018.jpg
https://www.dermis.net/bilder/CD004/550px/img0033.jpg
https://www.dermis.net/bilder/CD032/550px/img0022.jpg
https://www.dermis.net/bilder/CD037/550px/img0042.jpg
https://www.dermis.net/bilder/CD036/550px/img0021.jpg
https://www.dermis.net/bilder/CD036/550px/img0022.jpg

https://www.dermis.net/bilder/CD032/550px/img0023.jpg
https://www.dermis.net/bilder/CD006/550px/img0087.jpg
https://www.dermis.net/bilder/CD006/550px/img0088.jpg
https://www.dermis.net/bilder/CD015/550px/img0010.jpg
https://www.dermis.net/bilder/CD005/550px/img0039.jpg
https://www.dermis.net/bilder/CD040/550px/img0014.jpg
https://www.dermis.net/bilder/CD002/550px/img0080.jpg
https://www.dermis.net/bilder/CD037/550px/img0038.jpg
https://www.dermis.net/bilder/CD003/550px/img0061.jpg
https://www.dermis.net/bilder/CD004/550px/img0040.jpg
https://www.dermis.net/bilder/CD040/550px/img0015.jpg
https://www.dermis.net/bilder/CD055/550px/img0029.jpg
https://www.dermis.net/bilder/CD040/550px/img0016.jpg
https://www.dermis.net/bilder/CD005/550px/img0028.jpg
https://www.dermis.net/bilder/CD005/550px/img0030.jpg
https://www.dermis.net/bilder/CD050/550px/img0061.jpg
https://www.dermis.net/bilder/CD004/550px/img0042.jpg
https://www.dermis.net/bilder/CD020/550px/img0005.jpg
https://www.dermis.net/bilder/CD044/550px/img0051.jpg
https://www.dermis.net/bilder/CD052/550px/img0097.jpg
https://www.dermis.net/bilder/CD054/550px/img0050.jpg
https://www.dermis.net/bilder/CD025/550px/img0063.jpg
https://www.dermis.net/bilder/CD044/550px/img0009.jpg
https://www.dermis.net/bilder/CD025/550px/img0061.jpg
https://www.dermis.net/bilder/CD033/550px/img0096.jpg
https://www.dermis.net/bilder/CD033/550px/img0064.jpg
https://www.dermis.net/bilder/CD054/550px/img0051.jpg
https://www.dermis.net/bilder/CD035/550px/img0016.jpg
https://www.dermis.net/bilder/CD036/550px/img0036.jpg
https://www.dermis.net/bilder/CD026/550px/img0088.jpg
https://www.dermis.net/bilder/CD039/550px/img0016.jpg
https://www.dermis.net/bilder/CD007/550px/img0036.jpg
https://www.dermis.net/bilder/CD066/550px/img0002.jpg
https://www.dermis.net/bilder/CD006/550px/img0049.jpg
https://www.dermis.net/bilder/CD048/550px/img0020.jpg
https://www.dermis.net/bilder/CD001/550px/img0055.jpg
https://www.dermis.net/bilder/CD004/550px/img0071.jpg
https://www.dermis.net/bilder/CD049/550px/img0045.jpg
https://www.dermis.net/bilder/CD001/550px/img0100.jpg
https://www.dermis.net/bilder/CD001/550px/img0066.jpg
https://www.dermis.net/bilder/CD001/550px/img0078.jpg
https://www.dermis.net/bilder/CD049/550px/img0047.jpg
https://www.dermis.net/bilder/CD049/550px/img0048.jpg
https://www.dermis.net/bilder/CD048/550px/img0019.jpg
https://www.dermis.net/bilder/CD048/550px/img0021.jpg
https://www.dermis.net/bilder/CD048/550px/img0028.jpg
https://www.dermis.net/bilder/CD004/550px/img0099.jpg
https://www.dermis.net/bilder/CD046/550px/img0084.jpg

https://www.dermis.net/bilder/CD046/550px/img0083.jpg
https://www.dermis.net/bilder/CD024/550px/img0037.jpg
https://www.dermis.net/bilder/CD024/550px/img0038.jpg
https://www.dermis.net/bilder/CD035/550px/img0051.jpg
https://www.dermis.net/bilder/CD035/550px/img0052.jpg
https://www.dermis.net/bilder/CD005/550px/img0012.jpg
https://www.dermis.net/bilder/CD018/550px/img0025.jpg
https://www.dermis.net/bilder/CD004/550px/img0085.jpg
https://www.dermis.net/bilder/CD020/550px/img0027.jpg
https://www.dermis.net/bilder/CD005/550px/img0035.jpg
https://www.dermis.net/bilder/CD020/550px/img0026.jpg
https://www.dermis.net/bilder/CD057/550px/img0005.jpg
https://www.dermis.net/bilder/CD015/550px/img0011.jpg
https://www.dermis.net/bilder/CD001/550px/img0028.jpg
https://www.dermis.net/bilder/CD005/550px/img0036.jpg
https://www.dermis.net/bilder/CD050/550px/img0026.jpg
https://www.dermis.net/bilder/CD050/550px/img0025.jpg
https://www.dermis.net/bilder/CD005/550px/img0107.jpg
https://www.dermis.net/bilder/CD014/550px/img0031.jpg
https://www.dermis.net/bilder/CD046/550px/img0065.jpg
https://www.dermis.net/bilder/CD046/550px/img0066.jpg
https://www.dermis.net/bilder/CD003/550px/img0011.jpg
https://www.dermis.net/bilder/CD002/550px/img0066.jpg
https://www.dermis.net/bilder/CD004/550px/img0039.jpg
https://www.dermis.net/bilder/CD052/550px/img0026.jpg
https://www.dermis.net/bilder/CD047/550px/img0092.jpg
https://www.dermis.net/bilder/CD023/550px/img0084.jpg
https://www.dermis.net/bilder/CD047/550px/img0069.jpg
https://www.dermis.net/bilder/CD047/550px/img0070.jpg
https://www.dermis.net/bilder/CD034/550px/img0045.jpg
https://www.dermis.net/bilder/CD014/550px/img0078.jpg
https://www.dermis.net/bilder/CD037/550px/img0005.jpg
https://www.dermis.net/bilder/CD002/550px/img0089.jpg
https://www.dermis.net/bilder/CD051/550px/img0066.jpg
https://www.dermis.net/bilder/CD035/550px/img0031.jpg
https://www.dermis.net/bilder/CD034/550px/img0044.jpg
https://www.dermis.net/bilder/CD034/550px/img0046.jpg
https://www.dermis.net/bilder/CD044/550px/img0004.jpg
https://www.dermis.net/bilder/CD044/550px/img0002.jpg
https://www.dermis.net/bilder/CD044/550px/img0003.jpg
https://www.dermis.net/bilder/CD050/550px/img0053.jpg
https://www.dermis.net/bilder/CD050/550px/img0054.jpg
https://www.dermis.net/bilder/CD050/550px/img0056.jpg
https://www.dermis.net/bilder/CD044/550px/img0006.jpg
https://www.dermis.net/bilder/CD046/550px/img0094.jpg
https://www.dermis.net/bilder/CD048/550px/img0023.jpg
https://www.dermis.net/bilder/CD048/550px/img0024.jpg
https://www.dermis.net/bilder/CD015/550px/img0017.jpg

https://www.dermis.net/bilder/CD037/550px/img0017.jpg
https://www.dermis.net/bilder/CD024/550px/img0044.jpg
https://www.dermis.net/bilder/CD024/550px/img0046.jpg
https://www.dermis.net/bilder/CD027/550px/img0006.jpg
https://www.dermis.net/bilder/CD044/550px/img0075.jpg
https://www.dermis.net/bilder/CD044/550px/img0073.jpg
https://www.dermis.net/bilder/CD058/550px/img0078.jpg
https://www.dermis.net/bilder/CD058/550px/img0079.jpg
https://www.dermis.net/bilder/CD052/550px/img0004.jpg
https://www.dermis.net/bilder/CD037/550px/img0019.jpg
https://www.dermis.net/bilder/CD020/550px/img0099.jpg
https://www.dermis.net/bilder/CD027/550px/img0050.jpg
https://www.dermis.net/bilder/CD027/550px/img0053.jpg
https://www.dermis.net/bilder/CD027/550px/img0048.jpg
https://www.dermis.net/bilder/CD020/550px/img0098.jpg
https://www.dermis.net/bilder/CD027/550px/img0049.jpg
https://www.dermis.net/bilder/CD054/550px/img0001.jpg
https://www.dermis.net/bilder/CD020/550px/img0097.jpg
https://www.dermis.net/bilder/CD032/550px/img0031.jpg
https://www.dermis.net/bilder/CD006/550px/img0084.jpg
https://www.dermis.net/bilder/CD014/550px/img0019.jpg
https://www.dermis.net/bilder/CD054/550px/img0088.jpg
https://www.dermis.net/bilder/CD004/550px/img0059.jpg
https://www.dermis.net/bilder/CD026/550px/img0094.jpg
https://www.dermis.net/bilder/CD006/550px/img0066.jpg
https://www.dermis.net/bilder/CD003/550px/img0052.jpg
https://www.dermis.net/bilder/CD003/550px/img0053.jpg
https://www.dermis.net/bilder/CD040/550px/img0098.jpg
https://www.dermis.net/bilder/CD005/550px/img0029.jpg
https://www.dermis.net/bilder/CD023/550px/img0061.jpg
https://www.dermis.net/bilder/CD044/550px/img0007.jpg
https://www.dermis.net/bilder/CD020/550px/img0008.jpg
https://www.dermis.net/bilder/CD020/550px/img0009.jpg
https://www.dermis.net/bilder/CD004/550px/img0036.jpg
https://www.dermis.net/bilder/CD041/550px/img0027.jpg
https://www.dermis.net/bilder/CD014/550px/img0014.jpg
https://www.dermis.net/bilder/CD016/550px/img0051.jpg
https://www.dermis.net/bilder/CD033/550px/img0087.jpg
https://www.dermis.net/bilder/CD058/550px/img0090.jpg
https://www.dermis.net/bilder/CD003/550px/img0093.jpg
https://www.dermis.net/bilder/CD039/550px/img0050.jpg
https://www.dermis.net/bilder/CD046/550px/img0009.jpg
https://www.dermis.net/bilder/CD046/550px/img0010.jpg
https://www.dermis.net/bilder/CD039/550px/img0041.jpg
https://www.dermis.net/bilder/CD039/550px/img0042.jpg
https://www.dermis.net/bilder/CD046/550px/img0015.jpg
https://www.dermis.net/bilder/CD046/550px/img0016.jpg
https://www.dermis.net/bilder/CD037/550px/img0060.jpg

https://www.dermis.net/bilder/CD046/550px/img0093.jpg
https://www.dermis.net/bilder/CD026/550px/img0044.jpg
https://www.dermis.net/bilder/CD032/550px/img0071.jpg
https://www.dermis.net/bilder/CD051/550px/img0017.jpg
https://www.dermis.net/bilder/CD044/550px/img0063.jpg
https://www.dermis.net/bilder/CD001/550px/img0062.jpg
https://www.dermis.net/bilder/CD005/550px/img0027.jpg
https://www.dermis.net/bilder/CD002/550px/img0052.jpg
https://www.dermis.net/bilder/CD003/550px/img0096.jpg
https://www.dermis.net/bilder/CD003/550px/img0019.jpg
https://www.dermis.net/bilder/CD034/550px/img0031.jpg
https://www.dermis.net/bilder/CD034/550px/img0032.jpg
https://www.dermis.net/bilder/CD005/550px/img0092.jpg
https://www.dermis.net/bilder/CD048/550px/img0088.jpg
https://www.dermis.net/bilder/CD048/550px/img0089.jpg
https://www.dermis.net/bilder/CD006/550px/img0078.jpg
https://www.dermis.net/bilder/CD055/550px/img0036.jpg
https://www.dermis.net/bilder/CD087/550px/img0009.jpg
https://www.dermis.net/bilder/CD042/550px/img0089.jpg
https://www.dermis.net/bilder/CD212/550px/img0033.jpg
https://www.dermis.net/bilder/CD212/550px/img0034.jpg
https://www.dermis.net/bilder/CD212/550px/img0116.jpg
https://www.dermis.net/bilder/CD212/550px/img0117.jpg
https://www.dermis.net/bilder/CD212/550px/img0030.jpg
https://www.dermis.net/bilder/CD212/550px/img0031.jpg
https://www.dermis.net/bilder/CD212/550px/img0032.jpg
https://www.dermis.net/bilder/CD002/550px/img0072.jpg
https://www.dermis.net/bilder/CD194/550px/img0077.jpg
https://www.dermis.net/bilder/CD013/550px/img0020.jpg
https://www.dermis.net/bilder/CD179/550px/img0005.jpg
https://www.dermis.net/bilder/CD195/550px/img0069.jpg
https://www.dermis.net/bilder/CD195/550px/img0070.jpg
https://www.dermis.net/bilder/CD199/550px/img0044.jpg
https://www.dermis.net/bilder/CD007/550px/img0109.jpg
https://www.dermis.net/bilder/CD014/550px/img0089.jpg
https://www.dermis.net/bilder/CD056/550px/img0064.jpg
https://www.dermis.net/bilder/CD187/550px/img0028.jpg
https://www.dermis.net/bilder/CD210/550px/img0015.jpg
https://www.dermis.net/bilder/CD210/550px/img0016.jpg
https://www.dermis.net/bilder/CD198/550px/img0161.jpg
https://www.dermis.net/bilder/CD198/550px/img0162.jpg
https://www.dermis.net/bilder/CD054/550px/img0027.jpg
https://www.dermis.net/bilder/CD056/550px/img0079.jpg
https://www.dermis.net/bilder/CD056/550px/img0080.jpg
https://www.dermis.net/bilder/CD056/550px/img0081.jpg
https://www.dermis.net/bilder/CD040/550px/img0042.jpg
https://www.dermis.net/bilder/CD014/550px/img0018.jpg
https://www.dermis.net/bilder/CD040/550px/img0032.jpg

https://www.dermis.net/bilder/CD182/550px/img0062.jpg
https://www.dermis.net/bilder/CD185/550px/img0047.jpg
https://www.dermis.net/bilder/CD185/550px/img0048.jpg
https://www.dermis.net/bilder/CD178/550px/img0009.jpg
https://www.dermis.net/bilder/CD199/550px/img0004.jpg
https://www.dermis.net/bilder/CD070/550px/img0087.jpg
https://www.dermis.net/bilder/CD070/550px/img0088.jpg
https://www.dermis.net/bilder/CD001/550px/img0091.jpg
https://www.dermis.net/bilder/CD192/550px/img0076.jpg
https://www.dermis.net/bilder/CD021/550px/img0014.jpg
https://www.dermis.net/bilder/CD193/550px/img0035.jpg
https://www.dermis.net/bilder/CD193/550px/img0036.jpg
https://www.dermis.net/bilder/CD008/550px/img0003.jpg
https://www.dermis.net/bilder/CD182/550px/img0075.jpg
https://www.dermis.net/bilder/CD196/550px/img0173.jpg
https://www.dermis.net/bilder/CD018/550px/img0054.jpg
https://www.dermis.net/bilder/CD013/550px/img0016.jpg
https://www.dermis.net/bilder/CD003/550px/img0059.jpg
https://www.dermis.net/bilder/CD196/550px/img0142.jpg
https://www.dermis.net/bilder/CD001/550px/img0073.jpg
https://www.dermis.net/bilder/CD189/550px/img0069.jpg
https://www.dermis.net/bilder/CD189/550px/img0070.jpg
https://www.dermis.net/bilder/CD001/550px/img0069.jpg
https://www.dermis.net/bilder/CD041/550px/img0065.jpg
https://www.dermis.net/bilder/CD201/550px/img0006.jpg
https://www.dermis.net/bilder/CD195/550px/img0071.jpg
https://www.dermis.net/bilder/CD211/550px/img0089.jpg
https://www.dermis.net/bilder/CD033/550px/img0008.jpg
https://www.dermis.net/bilder/CD033/550px/img0024.jpg
https://www.dermis.net/bilder/CD036/550px/img0060.jpg
https://www.dermis.net/bilder/CD182/550px/img0001.jpg
https://www.dermis.net/bilder/CD032/550px/img0040.jpg
https://www.dermis.net/bilder/CD180/550px/img0065.jpg
https://www.dermis.net/bilder/CD180/550px/img0064.jpg
https://www.dermis.net/bilder/CD178/550px/img0093.jpg
https://www.dermis.net/bilder/CD001/550px/img0027.jpg
https://www.dermis.net/bilder/CD184/550px/img0021.jpg
https://www.dermis.net/bilder/CD184/550px/img0022.jpg
https://www.dermis.net/bilder/CD033/550px/img0001.jpg
https://www.dermis.net/bilder/CD178/550px/img0072.jpg
https://www.dermis.net/bilder/CD184/550px/img0058.jpg
https://www.dermis.net/bilder/CD194/550px/img0143.jpg
https://www.dermis.net/bilder/CD021/550px/img0005.jpg
https://www.dermis.net/bilder/CD033/550px/img0065.jpg
https://www.dermis.net/bilder/CD187/550px/img0008.jpg
https://www.dermis.net/bilder/CD196/550px/img0086.jpg
https://www.dermis.net/bilder/CD210/550px/img0094.jpg
https://www.dermis.net/bilder/CD011/550px/img0081.jpg

https://www.dermis.net/bilder/CD033/550px/img0007.jpg
https://www.dermis.net/bilder/CD017/550px/img0003.jpg
https://www.dermis.net/bilder/CD211/550px/img0088.jpg
https://www.dermis.net/bilder/CD178/550px/img0073.jpg
https://www.dermis.net/bilder/CD198/550px/img0187.jpg
https://www.dermis.net/bilder/CD198/550px/img0188.jpg
https://www.dermis.net/bilder/CD198/550px/img0120.jpg
https://www.dermis.net/bilder/CD187/550px/img0029.jpg
https://www.dermis.net/bilder/CD187/550px/img0030.jpg
https://www.dermis.net/bilder/CD180/550px/img0063.jpg
https://www.dermis.net/bilder/CD187/550px/img0032.jpg
https://www.dermis.net/bilder/CD008/550px/img0014.jpg
https://www.dermis.net/bilder/CD041/550px/img0023.jpg
https://www.dermis.net/bilder/CD041/550px/img0024.jpg
https://www.dermis.net/bilder/CD004/550px/img0091.jpg
https://www.dermis.net/bilder/CD210/550px/img0109.jpg
https://www.dermis.net/bilder/CD042/550px/img0083.jpg
https://www.dermis.net/bilder/CD042/550px/img0084.jpg
https://www.dermis.net/bilder/CD008/550px/img0075.jpg
https://www.dermis.net/bilder/CD008/550px/img0028.jpg
https://www.dermis.net/bilder/CD037/550px/img0047.jpg
https://www.dermis.net/bilder/CD001/550px/img0013.jpg
https://www.dermis.net/bilder/CD032/550px/img0050.jpg
https://www.dermis.net/bilder/CD181/550px/img0063.jpg
https://www.dermis.net/bilder/CD027/550px/img0023.jpg
https://www.dermis.net/bilder/CD198/550px/img0047.jpg
https://www.dermis.net/bilder/CD199/550px/img0020.jpg
https://www.dermis.net/bilder/CD212/550px/img0125.jpg
https://www.dermis.net/bilder/CD185/550px/img0066.jpg
https://www.dermis.net/bilder/CD196/550px/img0042.jpg
https://www.dermis.net/bilder/CD181/550px/img0020.jpg
https://www.dermis.net/bilder/CD181/550px/img0019.jpg
https://www.dermis.net/bilder/CD181/550px/img0016.jpg
https://www.dermis.net/bilder/CD180/550px/img0070.jpg
https://www.dermis.net/bilder/CD041/550px/img0060.jpg
https://www.dermis.net/bilder/CD199/550px/img0136.jpg
https://www.dermis.net/bilder/CD193/550px/img0046.jpg
https://www.dermis.net/bilder/CD210/550px/img0113.jpg
https://www.dermis.net/bilder/CD057/550px/img0067.jpg
https://www.dermis.net/bilder/CD057/550px/img0068.jpg
https://www.dermis.net/bilder/CD195/550px/img0044.jpg
https://www.dermis.net/bilder/CD212/550px/img0126.jpg
https://www.dermis.net/bilder/CD212/550px/img0127.jpg
https://www.dermis.net/bilder/CD212/550px/img0128.jpg
https://www.dermis.net/bilder/CD007/550px/img0117.jpg
https://www.dermis.net/bilder/CD008/550px/img0004.jpg
https://www.dermis.net/bilder/CD195/550px/img0056.jpg
https://www.dermis.net/bilder/CD195/550px/img0058.jpg

https://www.dermis.net/bilder/CD195/550px/img0059.jpg
https://www.dermis.net/bilder/CD195/550px/img0060.jpg
https://www.dermis.net/bilder/CD015/550px/img0002.jpg
https://www.dermis.net/bilder/CD198/550px/img0166.jpg
https://www.dermis.net/bilder/CD056/550px/img0044.jpg
https://www.dermis.net/bilder/CD211/550px/img0001.jpg
https://www.dermis.net/bilder/CD191/550px/img0038.jpg
https://www.dermis.net/bilder/CD191/550px/img0039.jpg
https://www.dermis.net/bilder/CD192/550px/img0043.jpg
https://www.dermis.net/bilder/CD042/550px/img0044.jpg
https://www.dermis.net/bilder/CD042/550px/img0045.jpg
https://www.dermis.net/bilder/CD180/550px/img0012.jpg
https://www.dermis.net/bilder/CD180/550px/img0013.jpg
https://www.dermis.net/bilder/CD180/550px/img0011.jpg
https://www.dermis.net/bilder/CD180/550px/img0010.jpg
https://www.dermis.net/bilder/CD033/550px/img0021.jpg
https://www.dermis.net/bilder/CD210/550px/img0103.jpg
https://www.dermis.net/bilder/CD181/550px/img0044.jpg
https://www.dermis.net/bilder/CD014/550px/img0070.jpg
https://www.dermis.net/bilder/CD180/550px/img0024.jpg
https://www.dermis.net/bilder/CD180/550px/img0025.jpg
https://www.dermis.net/bilder/CD041/550px/img0008.jpg
https://www.dermis.net/bilder/CD184/550px/img0074.jpg
https://www.dermis.net/bilder/CD181/550px/img0077.jpg
https://www.dermis.net/bilder/CD181/550px/img0075.jpg
https://www.dermis.net/bilder/CD198/550px/img0012.jpg
https://www.dermis.net/bilder/CD198/550px/img0013.jpg
https://www.dermis.net/bilder/CD198/550px/img0015.jpg
https://www.dermis.net/bilder/CD007/550px/img0091.jpg
https://www.dermis.net/bilder/CD007/550px/img0093.jpg
https://www.dermis.net/bilder/CD007/550px/img0097.jpg
https://www.dermis.net/bilder/CD007/550px/img0099.jpg
https://www.dermis.net/bilder/CD007/550px/img0094.jpg
https://www.dermis.net/bilder/CD007/550px/img0101.jpg
https://www.dermis.net/bilder/CD184/550px/img0059.jpg
https://www.dermis.net/bilder/CD184/550px/img0060.jpg
https://www.dermis.net/bilder/CD197/550px/img0018.jpg
https://www.dermis.net/bilder/CD197/550px/img0019.jpg
https://www.dermis.net/bilder/CD197/550px/img0020.jpg
https://www.dermis.net/bilder/CD197/550px/img0021.jpg
https://www.dermis.net/bilder/CD202/550px/img0030.jpg
https://www.dermis.net/bilder/CD012/550px/img0079.jpg
https://www.dermis.net/bilder/CD181/550px/img0054.jpg
https://www.dermis.net/bilder/CD011/550px/img0098.jpg
https://www.dermis.net/bilder/CD191/550px/img0029.jpg
https://www.dermis.net/bilder/CD191/550px/img0030.jpg
https://www.dermis.net/bilder/CD041/550px/img0092.jpg
https://www.dermis.net/bilder/CD043/550px/img0030.jpg

https://www.dermis.net/bilder/CD197/550px/img0002.jpg
https://www.dermis.net/bilder/CD196/550px/img0123.jpg
https://www.dermis.net/bilder/CD041/550px/img0062.jpg
https://www.dermis.net/bilder/CD056/550px/img0043.jpg
https://www.dermis.net/bilder/CD004/550px/img0100.jpg
https://www.dermis.net/bilder/CD042/550px/img0070.jpg
https://www.dermis.net/bilder/CD001/550px/img0065.jpg
https://www.dermis.net/bilder/CD001/550px/img0057.jpg
https://www.dermis.net/bilder/CD027/550px/img0019.jpg
https://www.dermis.net/bilder/CD085/550px/img0075.jpg
https://www.dermis.net/bilder/CD033/550px/img0063.jpg
https://www.dermis.net/bilder/CD021/550px/img0077.jpg
https://www.dermis.net/bilder/CD193/550px/img0186.jpg
https://www.dermis.net/bilder/CD193/550px/img0187.jpg
https://www.dermis.net/bilder/CD193/550px/img0189.jpg
https://www.dermis.net/bilder/CD193/550px/img0190.jpg
https://www.dermis.net/bilder/CD199/550px/img0125.jpg
https://www.dermis.net/bilder/CD211/550px/img0038.jpg
https://www.dermis.net/bilder/CD040/550px/img0085.jpg
https://www.dermis.net/bilder/CD040/550px/img0086.jpg
https://www.dermis.net/bilder/CD033/550px/img0052.jpg
https://www.dermis.net/bilder/CD002/550px/img0087.jpg
https://www.dermis.net/bilder/CD002/550px/img0088.jpg
https://www.dermis.net/bilder/CD002/550px/img0091.jpg
https://www.dermis.net/bilder/CD003/550px/img0003.jpg
https://www.dermis.net/bilder/CD013/550px/img0039.jpg
https://www.dermis.net/bilder/CD184/550px/img0105.jpg
https://www.dermis.net/bilder/CD184/550px/img0106.jpg
https://www.dermis.net/bilder/CD184/550px/img0107.jpg
https://www.dermis.net/bilder/CD184/550px/img0108.jpg
https://www.dermis.net/bilder/CD193/550px/img0198.jpg
https://www.dermis.net/bilder/CD193/550px/img0199.jpg
https://www.dermis.net/bilder/CD198/550px/img0093.jpg
https://www.dermis.net/bilder/CD198/550px/img0094.jpg
https://www.dermis.net/bilder/CD212/550px/img0134.jpg
https://www.dermis.net/bilder/CD212/550px/img0135.jpg
https://www.dermis.net/bilder/CD055/550px/img0023.jpg
https://www.dermis.net/bilder/CD041/550px/img0034.jpg
https://www.dermis.net/bilder/CD180/550px/img0079.jpg
https://www.dermis.net/bilder/CD180/550px/img0080.jpg
https://www.dermis.net/bilder/CD006/550px/img0086.jpg
https://www.dermis.net/bilder/CD001/550px/img0101.jpg
https://www.dermis.net/bilder/CD195/550px/img0117.jpg
https://www.dermis.net/bilder/CD195/550px/img0118.jpg
https://www.dermis.net/bilder/CD033/550px/img0066.jpg
https://www.dermis.net/bilder/CD202/550px/img0033.jpg
https://www.dermis.net/bilder/CD007/550px/img0025.jpg
https://www.dermis.net/bilder/CD025/550px/img0069.jpg

https://www.dermis.net/bilder/CD025/550px/img0071.jpg
https://www.dermis.net/bilder/CD180/550px/img0062.jpg
https://www.dermis.net/bilder/CD036/550px/img0080.jpg
https://www.dermis.net/bilder/CD189/550px/img0037.jpg
https://www.dermis.net/bilder/CD198/550px/img0011.jpg
https://www.dermis.net/bilder/CD007/550px/img0096.jpg
https://www.dermis.net/bilder/CD042/550px/img0093.jpg
https://www.dermis.net/bilder/CD021/550px/img0018.jpg
https://www.dermis.net/bilder/CD042/550px/img0032.jpg
https://www.dermis.net/bilder/CD056/550px/img0066.jpg
https://www.dermis.net/bilder/CD056/550px/img0068.jpg
https://www.dermis.net/bilder/CD189/550px/img0010.jpg
https://www.dermis.net/bilder/CD193/550px/img0141.jpg
https://www.dermis.net/bilder/CD197/550px/img0078.jpg
https://www.dermis.net/bilder/CD197/550px/img0079.jpg
https://www.dermis.net/bilder/CD197/550px/img0001.jpg
https://www.dermis.net/bilder/CD193/550px/img0073.jpg
https://www.dermis.net/bilder/CD013/550px/img0005.jpg
https://www.dermis.net/bilder/CD042/550px/img0082.jpg
https://www.dermis.net/bilder/CD042/550px/img0053.jpg
https://www.dermis.net/bilder/CD021/550px/img0006.jpg
https://www.dermis.net/bilder/CD197/550px/img0003.jpg
https://www.dermis.net/bilder/CD193/550px/img0131.jpg
https://www.dermis.net/bilder/CD193/550px/img0132.jpg
https://www.dermis.net/bilder/CD196/550px/img0143.jpg
https://www.dermis.net/bilder/CD195/550px/img0047.jpg
https://www.dermis.net/bilder/CD178/550px/img0076.jpg
https://www.dermis.net/bilder/CD178/550px/img0077.jpg
https://www.dermis.net/bilder/CD178/550px/img0011.jpg
https://www.dermis.net/bilder/CD178/550px/img0012.jpg
https://www.dermis.net/bilder/CD198/550px/img0074.jpg
https://www.dermis.net/bilder/CD185/550px/img0026.jpg
https://www.dermis.net/bilder/CD196/550px/img0071.jpg
https://www.dermis.net/bilder/CD196/550px/img0070.jpg
https://www.dermis.net/bilder/CD008/550px/img0050.jpg
https://www.dermis.net/bilder/CD184/550px/img0035.jpg
https://www.dermis.net/bilder/CD178/550px/img0058.jpg
https://www.dermis.net/bilder/CD185/550px/img0059.jpg
https://www.dermis.net/bilder/CD196/550px/img0041.jpg
https://www.dermis.net/bilder/CD212/550px/img0123.jpg
https://www.dermis.net/bilder/CD212/550px/img0124.jpg
https://www.dermis.net/bilder/CD187/550px/img0026.jpg
https://www.dermis.net/bilder/CD187/550px/img0027.jpg
https://www.dermis.net/bilder/CD196/550px/img0109.jpg
https://www.dermis.net/bilder/CD036/550px/img0071.jpg
https://www.dermis.net/bilder/CD033/550px/img0041.jpg
https://www.dermis.net/bilder/CD021/550px/img0059.jpg
https://www.dermis.net/bilder/CD195/550px/img0092.jpg

https://www.dermis.net/bilder/CD040/550px/img0044.jpg
https://www.dermis.net/bilder/CD180/550px/img0067.jpg
https://www.dermis.net/bilder/CD180/550px/img0069.jpg
https://www.dermis.net/bilder/CD021/550px/img0095.jpg
https://www.dermis.net/bilder/CD193/550px/img0079.jpg
https://www.dermis.net/bilder/CD198/550px/img0019.jpg
https://www.dermis.net/bilder/CD198/550px/img0020.jpg
https://www.dermis.net/bilder/CD198/550px/img0021.jpg
https://www.dermis.net/bilder/CD001/550px/img0094.jpg
https://www.dermis.net/bilder/CD001/550px/img0033.jpg
https://www.dermis.net/bilder/CD041/550px/img0036.jpg
https://www.dermis.net/bilder/CD002/550px/img0092.jpg
https://www.dermis.net/bilder/CD021/550px/img0045.jpg
https://www.dermis.net/bilder/CD193/550px/img0194.jpg
https://www.dermis.net/bilder/CD041/550px/img0001.jpg
https://www.dermis.net/bilder/CD041/550px/img0002.jpg
https://www.dermis.net/bilder/CD041/550px/img0003.jpg
https://www.dermis.net/bilder/CD194/550px/img0101.jpg
https://www.dermis.net/bilder/CD196/550px/img0083.jpg
https://www.dermis.net/bilder/CD196/550px/img0084.jpg
https://www.dermis.net/bilder/CD180/550px/img0093.jpg
https://www.dermis.net/bilder/CD197/550px/img0011.jpg
https://www.dermis.net/bilder/CD183/550px/img0074.jpg
https://www.dermis.net/bilder/CD211/550px/img0032.jpg
https://www.dermis.net/bilder/CD211/550px/img0033.jpg
https://www.dermis.net/bilder/CD197/550px/img0075.jpg
https://www.dermis.net/bilder/CD011/550px/img0080.jpg
https://www.dermis.net/bilder/CD196/550px/img0174.jpg
https://www.dermis.net/bilder/CD197/550px/img0004.jpg
https://www.dermis.net/bilder/CD196/550px/img0127.jpg
https://www.dermis.net/bilder/CD008/550px/img0054.jpg
https://www.dermis.net/bilder/CD041/550px/img0059.jpg
https://www.dermis.net/bilder/CD002/550px/img0071.jpg
https://www.dermis.net/bilder/CD180/550px/img0066.jpg
https://www.dermis.net/bilder/CD033/550px/img0059.jpg
https://www.dermis.net/bilder/CD055/550px/img0043.jpg
https://www.dermis.net/bilder/CD191/550px/img0088.jpg
https://www.dermis.net/bilder/CD040/550px/img0045.jpg
https://www.dermis.net/bilder/CD040/550px/img0046.jpg
https://www.dermis.net/bilder/CD040/550px/img0047.jpg
https://www.dermis.net/bilder/CD033/550px/img0053.jpg
https://www.dermis.net/bilder/CD050/550px/img0027.jpg
https://www.dermis.net/bilder/CD050/550px/img0028.jpg
https://www.dermis.net/bilder/CD033/550px/img0054.jpg
https://www.dermis.net/bilder/CD185/550px/img0022.jpg
https://www.dermis.net/bilder/CD185/550px/img0023.jpg
https://www.dermis.net/bilder/CD210/550px/img0107.jpg
https://www.dermis.net/bilder/CD210/550px/img0110.jpg

https://www.dermis.net/bilder/CD210/550px/img0111.jpg
https://www.dermis.net/bilder/CD043/550px/img0001.jpg
https://www.dermis.net/bilder/CD001/550px/img0016.jpg
https://www.dermis.net/bilder/CD001/550px/img0089.jpg
https://www.dermis.net/bilder/CD045/550px/img0049.jpg
https://www.dermis.net/bilder/CD056/550px/img0042.jpg
https://www.dermis.net/bilder/CD192/550px/img0044.jpg
https://www.dermis.net/bilder/CD192/550px/img0045.jpg
https://www.dermis.net/bilder/CD192/550px/img0046.jpg
https://www.dermis.net/bilder/CD192/550px/img0047.jpg
https://www.dermis.net/bilder/CD018/550px/img0115.jpg
https://www.dermis.net/bilder/CD193/550px/img0108.jpg
https://www.dermis.net/bilder/CD027/550px/img0025.jpg
https://www.dermis.net/bilder/CD027/550px/img0026.jpg
https://www.dermis.net/bilder/CD021/550px/img0066.jpg
https://www.dermis.net/bilder/CD210/550px/img0101.jpg
https://www.dermis.net/bilder/CD181/550px/img0045.jpg
https://www.dermis.net/bilder/CD193/550px/img0048.jpg
https://www.dermis.net/bilder/CD193/550px/img0049.jpg
https://www.dermis.net/bilder/CD193/550px/img0087.jpg
https://www.dermis.net/bilder/CD181/550px/img0078.jpg
https://www.dermis.net/bilder/CD181/550px/img0076.jpg
https://www.dermis.net/bilder/CD184/550px/img0018.jpg
https://www.dermis.net/bilder/CD184/550px/img0019.jpg
https://www.dermis.net/bilder/CD184/550px/img0020.jpg
https://www.dermis.net/bilder/CD013/550px/img0003.jpg
https://www.dermis.net/bilder/CD178/550px/img0071.jpg
https://www.dermis.net/bilder/CD021/550px/img0001.jpg
https://www.dermis.net/bilder/CD189/550px/img0063.jpg
https://www.dermis.net/bilder/CD189/550px/img0064.jpg
https://www.dermis.net/bilder/CD042/550px/img0094.jpg
https://www.dermis.net/bilder/CD181/550px/img0051.jpg
https://www.dermis.net/bilder/CD181/550px/img0053.jpg
https://www.dermis.net/bilder/CD181/550px/img0050.jpg
https://www.dermis.net/bilder/CD190/550px/img0031.jpg
https://www.dermis.net/bilder/CD190/550px/img0032.jpg
https://www.dermis.net/bilder/CD191/550px/img0006.jpg
https://www.dermis.net/bilder/CD191/550px/img0008.jpg
https://www.dermis.net/bilder/CD185/550px/img0039.jpg
https://www.dermis.net/bilder/CD185/550px/img0040.jpg
https://www.dermis.net/bilder/CD042/550px/img0014.jpg
https://www.dermis.net/bilder/CD021/550px/img0063.jpg
https://www.dermis.net/bilder/CD021/550px/img0065.jpg
https://www.dermis.net/bilder/CD193/550px/img0074.jpg
https://www.dermis.net/bilder/CD192/550px/img0091.jpg
https://www.dermis.net/bilder/CD192/550px/img0090.jpg
https://www.dermis.net/bilder/CD008/550px/img0029.jpg
https://www.dermis.net/bilder/CD198/550px/img0167.jpg

https://www.dermis.net/bilder/CD008/550px/img0005.jpg
https://www.dermis.net/bilder/CD055/550px/img0032.jpg
https://www.dermis.net/bilder/CD055/550px/img0039.jpg
https://www.dermis.net/bilder/CD197/550px/img0071.jpg
https://www.dermis.net/bilder/CD055/550px/img0025.jpg
https://www.dermis.net/bilder/CD033/550px/img0049.jpg
https://www.dermis.net/bilder/CD191/550px/img0107.jpg
https://www.dermis.net/bilder/CD191/550px/img0106.jpg
https://www.dermis.net/bilder/CD005/550px/img0104.jpg
https://www.dermis.net/bilder/CD033/550px/img0058.jpg
https://www.dermis.net/bilder/CD036/550px/img0001.jpg
https://www.dermis.net/bilder/CD198/550px/img0129.jpg
https://www.dermis.net/bilder/CD210/550px/img0126.jpg
https://www.dermis.net/bilder/CD210/550px/img0087.jpg
https://www.dermis.net/bilder/CD001/550px/img0077.jpg
https://www.dermis.net/bilder/CD184/550px/img0073.jpg
https://www.dermis.net/bilder/CD190/550px/img0050.jpg
https://www.dermis.net/bilder/CD190/550px/img0051.jpg
https://www.dermis.net/bilder/CD199/550px/img0017.jpg
https://www.dermis.net/bilder/CD199/550px/img0018.jpg
https://www.dermis.net/bilder/CD199/550px/img0063.jpg
https://www.dermis.net/bilder/CD199/550px/img0064.jpg
https://www.dermis.net/bilder/CD051/550px/img0001.jpg
https://www.dermis.net/bilder/CD001/550px/img0012.jpg
https://www.dermis.net/bilder/CD036/550px/img0047.jpg
https://www.dermis.net/bilder/CD043/550px/img0002.jpg
https://www.dermis.net/bilder/CD181/550px/img0030.jpg
https://www.dermis.net/bilder/CD181/550px/img0033.jpg
https://www.dermis.net/bilder/CD181/550px/img0035.jpg
https://www.dermis.net/bilder/CD181/550px/img0034.jpg
https://www.dermis.net/bilder/CD181/550px/img0032.jpg
https://www.dermis.net/bilder/CD181/550px/img0031.jpg
https://www.dermis.net/bilder/CD179/550px/img0006.jpg
https://www.dermis.net/bilder/CD185/550px/img0027.jpg
https://www.dermis.net/bilder/CD185/550px/img0028.jpg
https://www.dermis.net/bilder/CD185/550px/img0030.jpg
https://www.dermis.net/bilder/CD185/550px/img0031.jpg
https://www.dermis.net/bilder/CD210/550px/img0021.jpg
https://www.dermis.net/bilder/CD184/550px/img0097.jpg
https://www.dermis.net/bilder/CD183/550px/img0050.jpg
https://www.dermis.net/bilder/CD183/550px/img0051.jpg
https://www.dermis.net/bilder/CD185/550px/img0060.jpg
https://www.dermis.net/bilder/CD185/550px/img0061.jpg
https://www.dermis.net/bilder/CD182/550px/img0042.jpg
https://www.dermis.net/bilder/CD182/550px/img0041.jpg
https://www.dermis.net/bilder/CD180/550px/img0045.jpg
https://www.dermis.net/bilder/CD195/550px/img0004.jpg
https://www.dermis.net/bilder/CD210/550px/img0014.jpg

https://www.dermis.net/bilder/CD001/550px/img0018.jpg
https://www.dermis.net/bilder/CD021/550px/img0038.jpg
https://www.dermis.net/bilder/CD179/550px/img0023.jpg
https://www.dermis.net/bilder/CD189/550px/img0058.jpg
https://www.dermis.net/bilder/CD189/550px/img0061.jpg
https://www.dermis.net/bilder/CD194/550px/img0063.jpg
https://www.dermis.net/bilder/CD192/550px/img0009.jpg
https://www.dermis.net/bilder/CD211/550px/img0058.jpg
https://www.dermis.net/bilder/CD185/550px/img0096.jpg
https://www.dermis.net/bilder/CD197/550px/img0068.jpg
https://www.dermis.net/bilder/CD197/550px/img0069.jpg
https://www.dermis.net/bilder/CD187/550px/img0093.jpg
https://www.dermis.net/bilder/CD187/550px/img0094.jpg
https://www.dermis.net/bilder/CD185/550px/img0099.jpg
https://www.dermis.net/bilder/CD193/550px/img0009.jpg
https://www.dermis.net/bilder/CD192/550px/img0073.jpg
https://www.dermis.net/bilder/CD001/550px/img0032.jpg
https://www.dermis.net/bilder/CD198/550px/img0178.jpg
https://www.dermis.net/bilder/CD198/550px/img0179.jpg
https://www.dermis.net/bilder/CD188/550px/img0013.jpg
https://www.dermis.net/bilder/CD188/550px/img0014.jpg
https://www.dermis.net/bilder/CD188/550px/img0015.jpg
https://www.dermis.net/bilder/CD189/550px/img0001.jpg
https://www.dermis.net/bilder/CD008/550px/img0008.jpg
https://www.dermis.net/bilder/CD178/550px/img0070.jpg
https://www.dermis.net/bilder/CD199/550px/img0047.jpg
https://www.dermis.net/bilder/CD190/550px/img0019.jpg
https://www.dermis.net/bilder/CD190/550px/img0020.jpg
https://www.dermis.net/bilder/CD194/550px/img0108.jpg
https://www.dermis.net/bilder/CD197/550px/img0077.jpg
https://www.dermis.net/bilder/CD195/550px/img0111.jpg
https://www.dermis.net/bilder/CD181/550px/img0064.jpg
https://www.dermis.net/bilder/CD194/550px/img0100.jpg
https://www.dermis.net/bilder/CD198/550px/img0006.jpg
https://www.dermis.net/bilder/CD196/550px/img0085.jpg
https://www.dermis.net/bilder/CD021/550px/img0072.jpg
https://www.dermis.net/bilder/CD033/550px/img0042.jpg
https://www.dermis.net/bilder/CD002/550px/img0096.jpg
https://www.dermis.net/bilder/CD014/550px/img0025.jpg
https://www.dermis.net/bilder/CD007/550px/img0044.jpg
https://www.dermis.net/bilder/CD021/550px/img0013.jpg
https://www.dermis.net/bilder/CD041/550px/img0022.jpg
https://www.dermis.net/bilder/CD071/550px/img0044.jpg
https://www.dermis.net/bilder/CD056/550px/img0006.jpg
https://www.dermis.net/bilder/CD071/550px/img0045.jpg
https://www.dermis.net/bilder/CD034/550px/img0075.jpg
https://www.dermis.net/bilder/CD056/550px/img0007.jpg
https://www.dermis.net/bilder/CD056/550px/img0047.jpg

https://www.dermis.net/bilder/CD014/550px/img0055.jpg
https://www.dermis.net/bilder/CD070/550px/img0038.jpg
https://www.dermis.net/bilder/CD069/550px/img0090.jpg
https://www.dermis.net/bilder/CD040/550px/img0066.jpg
https://www.dermis.net/bilder/CD069/550px/img0091.jpg
https://www.dermis.net/bilder/CD056/550px/img0020.jpg
https://www.dermis.net/bilder/CD034/550px/img0059.jpg
https://www.dermis.net/bilder/CD042/550px/img0077.jpg
https://www.dermis.net/bilder/CD069/550px/img0092.jpg
https://www.dermis.net/bilder/CD069/550px/img0109.jpg
https://www.dermis.net/bilder/CD069/550px/img0112.jpg
https://www.dermis.net/bilder/CD071/550px/img0014.jpg
https://www.dermis.net/bilder/CD069/550px/img0110.jpg
https://www.dermis.net/bilder/CD025/550px/img0047.jpg
https://www.dermis.net/bilder/CD070/550px/img0044.jpg
https://www.dermis.net/bilder/CD071/550px/img0110.jpg
https://www.dermis.net/bilder/CD071/550px/img0033.jpg
https://www.dermis.net/bilder/CD071/550px/img0034.jpg
https://www.dermis.net/bilder/CD018/550px/img0116.jpg
https://www.dermis.net/bilder/CD070/550px/img0040.jpg
https://www.dermis.net/bilder/CD020/550px/img0038.jpg
https://www.dermis.net/bilder/CD020/550px/img0040.jpg
https://www.dermis.net/bilder/CD070/550px/img0094.jpg
https://www.dermis.net/bilder/CD056/550px/img0012.jpg
https://www.dermis.net/bilder/CD056/550px/img0026.jpg
https://www.dermis.net/bilder/CD050/550px/img0030.jpg
https://www.dermis.net/bilder/CD050/550px/img0031.jpg
https://www.dermis.net/bilder/CD071/550px/img0012.jpg
https://www.dermis.net/bilder/CD057/550px/img0011.jpg
https://www.dermis.net/bilder/CD006/550px/img0005.jpg
https://www.dermis.net/bilder/CD018/550px/img0041.jpg
https://www.dermis.net/bilder/CD071/550px/img0030.jpg
https://www.dermis.net/bilder/CD048/550px/img0036.jpg
https://www.dermis.net/bilder/CD048/550px/img0037.jpg
https://www.dermis.net/bilder/CD005/550px/img0003.jpg
https://www.dermis.net/bilder/CD071/550px/img0047.jpg
https://www.dermis.net/bilder/CD018/550px/img0065.jpg
https://www.dermis.net/bilder/CD069/550px/img0083.jpg
https://www.dermis.net/bilder/CD035/550px/img0012.jpg
https://www.dermis.net/bilder/CD037/550px/img0070.jpg
https://www.dermis.net/bilder/CD006/550px/img0081.jpg
https://www.dermis.net/bilder/CD020/550px/img0079.jpg
https://www.dermis.net/bilder/CD020/550px/img0080.jpg
https://www.dermis.net/bilder/CD043/550px/img0009.jpg
https://www.dermis.net/bilder/CD035/550px/img0010.jpg
https://www.dermis.net/bilder/CD069/550px/img0080.jpg
https://www.dermis.net/bilder/CD058/550px/img0075.jpg
https://www.dermis.net/bilder/CD026/550px/img0024.jpg

https://www.dermis.net/bilder/CD069/550px/img0013.jpg
https://www.dermis.net/bilder/CD069/550px/img0014.jpg
https://www.dermis.net/bilder/CD069/550px/img0015.jpg
https://www.dermis.net/bilder/CD069/550px/img0016.jpg
https://www.dermis.net/bilder/CD058/550px/img0076.jpg
https://www.dermis.net/bilder/CD006/550px/img0034.jpg
https://www.dermis.net/bilder/CD069/550px/img0019.jpg
https://www.dermis.net/bilder/CD069/550px/img0020.jpg
https://www.dermis.net/bilder/CD004/550px/img0067.jpg
https://www.dermis.net/bilder/CD070/550px/img0035.jpg
https://www.dermis.net/bilder/CD015/550px/img0018.jpg
https://www.dermis.net/bilder/CD047/550px/img0047.jpg
https://www.dermis.net/bilder/CD069/550px/img0010.jpg
https://www.dermis.net/bilder/CD005/550px/img0032.jpg
https://www.dermis.net/bilder/CD018/550px/img0107.jpg
https://www.dermis.net/bilder/CD069/550px/img0011.jpg
https://www.dermis.net/bilder/CD052/550px/img0015.jpg
https://www.dermis.net/bilder/CD056/550px/img0056.jpg
https://www.dermis.net/bilder/CD018/550px/img0058.jpg
https://www.dermis.net/bilder/CD051/550px/img0078.jpg
https://www.dermis.net/bilder/CD021/550px/img0085.jpg
https://www.dermis.net/bilder/CD069/550px/img0077.jpg
https://www.dermis.net/bilder/CD001/550px/img0045.jpg
https://www.dermis.net/bilder/CD016/550px/img0061.jpg
https://www.dermis.net/bilder/CD069/550px/img0076.jpg
https://www.dermis.net/bilder/CD071/550px/img0035.jpg
https://www.dermis.net/bilder/CD023/550px/img0046.jpg
https://www.dermis.net/bilder/CD051/550px/img0031.jpg
https://www.dermis.net/bilder/CD006/550px/img0050.jpg
https://www.dermis.net/bilder/CD020/550px/img0041.jpg
https://www.dermis.net/bilder/CD020/550px/img0042.jpg
https://www.dermis.net/bilder/CD054/550px/img0012.jpg
https://www.dermis.net/bilder/CD005/550px/img0021.jpg
https://www.dermis.net/bilder/CD016/550px/img0029.jpg
https://www.dermis.net/bilder/CD006/550px/img0026.jpg
https://www.dermis.net/bilder/CD002/550px/img0077.jpg
https://www.dermis.net/bilder/CD071/550px/img0006.jpg
https://www.dermis.net/bilder/CD005/550px/img0095.jpg
https://www.dermis.net/bilder/CD048/550px/img0065.jpg
https://www.dermis.net/bilder/CD007/550px/img0026.jpg
https://www.dermis.net/bilder/CD041/550px/img0026.jpg
https://www.dermis.net/bilder/CD018/550px/img0085.jpg
https://www.dermis.net/bilder/CD036/550px/img0082.jpg
https://www.dermis.net/bilder/CD006/550px/img0097.jpg
https://www.dermis.net/bilder/CD034/550px/img0093.jpg
https://www.dermis.net/bilder/CD034/550px/img0096.jpg
https://www.dermis.net/bilder/CD006/550px/img0096.jpg
https://www.dermis.net/bilder/CD007/550px/img0030.jpg

https://www.dermis.net/bilder/CD056/550px/img0013.jpg
https://www.dermis.net/bilder/CD007/550px/img0028.jpg
https://www.dermis.net/bilder/CD020/550px/img0029.jpg
https://www.dermis.net/bilder/CD020/550px/img0030.jpg
https://www.dermis.net/bilder/CD020/550px/img0031.jpg
https://www.dermis.net/bilder/CD028/550px/img0058.jpg
https://www.dermis.net/bilder/CD028/550px/img0059.jpg
https://www.dermis.net/bilder/CD044/550px/img0039.jpg
https://www.dermis.net/bilder/CD051/550px/img0021.jpg
https://www.dermis.net/bilder/CD043/550px/img0021.jpg
https://www.dermis.net/bilder/CD023/550px/img0034.jpg
https://www.dermis.net/bilder/CD071/550px/img0023.jpg
https://www.dermis.net/bilder/CD007/550px/img0045.jpg
https://www.dermis.net/bilder/CD034/550px/img0026.jpg
https://www.dermis.net/bilder/CD034/550px/img0025.jpg
https://www.dermis.net/bilder/CD023/550px/img0030.jpg
https://www.dermis.net/bilder/CD071/550px/img0024.jpg
https://www.dermis.net/bilder/CD046/550px/img0048.jpg
https://www.dermis.net/bilder/CD052/550px/img0089.jpg
https://www.dermis.net/bilder/CD052/550px/img0090.jpg
https://www.dermis.net/bilder/CD052/550px/img0091.jpg
https://www.dermis.net/bilder/CD046/550px/img0047.jpg
https://www.dermis.net/bilder/CD017/550px/img0088.jpg
https://www.dermis.net/bilder/CD020/550px/img0025.jpg
https://www.dermis.net/bilder/CD070/550px/img0068.jpg
https://www.dermis.net/bilder/CD014/550px/img0008.jpg
https://www.dermis.net/bilder/CD006/550px/img0019.jpg
https://www.dermis.net/bilder/CD007/550px/img0034.jpg
https://www.dermis.net/bilder/CD056/550px/img0049.jpg
https://www.dermis.net/bilder/CD018/550px/img0074.jpg
https://www.dermis.net/bilder/CD018/550px/img0075.jpg
https://www.dermis.net/bilder/CD052/550px/img0056.jpg
https://www.dermis.net/bilder/CD052/550px/img0049.jpg
https://www.dermis.net/bilder/CD023/550px/img0071.jpg
https://www.dermis.net/bilder/CD023/550px/img0073.jpg
https://www.dermis.net/bilder/CD042/550px/img0007.jpg
https://www.dermis.net/bilder/CD023/550px/img0072.jpg
https://www.dermis.net/bilder/CD034/550px/img0066.jpg
https://www.dermis.net/bilder/CD034/550px/img0067.jpg
https://www.dermis.net/bilder/CD042/550px/img0006.jpg
https://www.dermis.net/bilder/CD020/550px/img0069.jpg
https://www.dermis.net/bilder/CD005/550px/img0050.jpg
https://www.dermis.net/bilder/CD004/550px/img0068.jpg
https://www.dermis.net/bilder/CD069/550px/img0017.jpg
https://www.dermis.net/bilder/CD008/550px/img0033.jpg
https://www.dermis.net/bilder/CD058/550px/img0015.jpg
https://www.dermis.net/bilder/CD058/550px/img0020.jpg
https://www.dermis.net/bilder/CD034/550px/img0064.jpg

https://www.dermis.net/bilder/CD044/550px/img0089.jpg
https://www.dermis.net/bilder/CD044/550px/img0090.jpg
https://www.dermis.net/bilder/CD052/550px/img0034.jpg
https://www.dermis.net/bilder/CD020/550px/img0093.jpg
https://www.dermis.net/bilder/CD040/550px/img0034.jpg
https://www.dermis.net/bilder/CD029/550px/img0018.jpg
https://www.dermis.net/bilder/CD029/550px/img0025.jpg
https://www.dermis.net/bilder/CD029/550px/img0027.jpg
https://www.dermis.net/bilder/CD029/550px/img0028.jpg
https://www.dermis.net/bilder/CD003/550px/img0005.jpg
https://www.dermis.net/bilder/CD006/550px/img0056.jpg
https://www.dermis.net/bilder/CD047/550px/img0078.jpg
https://www.dermis.net/bilder/CD017/550px/img0099.jpg
https://www.dermis.net/bilder/CD005/550px/img0017.jpg
https://www.dermis.net/bilder/CD047/550px/img0077.jpg
https://www.dermis.net/bilder/CD025/550px/img0086.jpg
https://www.dermis.net/bilder/CD025/550px/img0087.jpg
https://www.dermis.net/bilder/CD025/550px/img0088.jpg
https://www.dermis.net/bilder/CD024/550px/img0058.jpg
https://www.dermis.net/bilder/CD024/550px/img0059.jpg
https://www.dermis.net/bilder/CD007/550px/img0040.jpg
https://www.dermis.net/bilder/CD036/550px/img0063.jpg
https://www.dermis.net/bilder/CD006/550px/img0057.jpg
https://www.dermis.net/bilder/CD040/550px/img0005.jpg
https://www.dermis.net/bilder/CD040/550px/img0006.jpg
https://www.dermis.net/bilder/CD040/550px/img0007.jpg
https://www.dermis.net/bilder/CD040/550px/img0012.jpg
https://www.dermis.net/bilder/CD046/550px/img0099.jpg
https://www.dermis.net/bilder/CD027/550px/img0061.jpg
https://www.dermis.net/bilder/CD027/550px/img0062.jpg
https://www.dermis.net/bilder/CD027/550px/img0063.jpg
https://www.dermis.net/bilder/CD019/550px/img0008.jpg
https://www.dermis.net/bilder/CD003/550px/img0008.jpg
https://www.dermis.net/bilder/CD008/550px/img0037.jpg
https://www.dermis.net/bilder/CD024/550px/img0097.jpg
https://www.dermis.net/bilder/CD015/550px/img0048.jpg
https://www.dermis.net/bilder/CD006/550px/img0017.jpg
https://www.dermis.net/bilder/CD016/550px/img0058.jpg
https://www.dermis.net/bilder/CD041/550px/img0028.jpg
https://www.dermis.net/bilder/CD052/550px/img0019.jpg
https://www.dermis.net/bilder/CD054/550px/img0059.jpg
https://www.dermis.net/bilder/CD054/550px/img0060.jpg
https://www.dermis.net/bilder/CD054/550px/img0061.jpg
https://www.dermis.net/bilder/CD006/550px/img0018.jpg
https://www.dermis.net/bilder/CD006/550px/img0021.jpg
https://www.dermis.net/bilder/CD006/550px/img0042.jpg
https://www.dermis.net/bilder/CD005/550px/img0108.jpg
https://www.dermis.net/bilder/CD034/550px/img0049.jpg

https://www.dermis.net/bilder/CD023/550px/img0075.jpg
https://www.dermis.net/bilder/CD023/550px/img0076.jpg
https://www.dermis.net/bilder/CD008/550px/img0051.jpg
https://www.dermis.net/bilder/CD048/550px/img0096.jpg
https://www.dermis.net/bilder/CD043/550px/img0028.jpg
https://www.dermis.net/bilder/CD040/550px/img0049.jpg
https://www.dermis.net/bilder/CD034/550px/img0050.jpg
https://www.dermis.net/bilder/CD054/550px/img0053.jpg
https://www.dermis.net/bilder/CD054/550px/img0054.jpg
https://www.dermis.net/bilder/CD037/550px/img0036.jpg
https://www.dermis.net/bilder/CD046/550px/img0006.jpg
https://www.dermis.net/bilder/CD046/550px/img0007.jpg
https://www.dermis.net/bilder/CD046/550px/img0008.jpg
https://www.dermis.net/bilder/CD020/550px/img0084.jpg
https://www.dermis.net/bilder/CD049/550px/img0043.jpg
https://www.dermis.net/bilder/CD049/550px/img0044.jpg
https://www.dermis.net/bilder/CD046/550px/img0081.jpg
https://www.dermis.net/bilder/CD046/550px/img0082.jpg
https://www.dermis.net/bilder/CD049/550px/img0042.jpg
https://www.dermis.net/bilder/CD027/550px/img0013.jpg
https://www.dermis.net/bilder/CD027/550px/img0014.jpg
https://www.dermis.net/bilder/CD003/550px/img0001.jpg
https://www.dermis.net/bilder/CD050/550px/img0032.jpg
https://www.dermis.net/bilder/CD037/550px/img0045.jpg
https://www.dermis.net/bilder/CD005/550px/img0085.jpg
https://www.dermis.net/bilder/CD006/550px/img0094.jpg
https://www.dermis.net/bilder/CD004/550px/img0086.jpg
https://www.dermis.net/bilder/CD005/550px/img0022.jpg
https://www.dermis.net/bilder/CD055/550px/img0037.jpg
https://www.dermis.net/bilder/CD023/550px/img0008.jpg
https://www.dermis.net/bilder/CD018/550px/img0094.jpg
https://www.dermis.net/bilder/CD026/550px/img0079.jpg
https://www.dermis.net/bilder/CD026/550px/img0080.jpg
https://www.dermis.net/bilder/CD017/550px/img0092.jpg
https://www.dermis.net/bilder/CD051/550px/img0012.jpg
https://www.dermis.net/bilder/CD051/550px/img0013.jpg
https://www.dermis.net/bilder/CD034/550px/img0020.jpg
https://www.dermis.net/bilder/CD034/550px/img0019.jpg
https://www.dermis.net/bilder/CD051/550px/img0106.jpg
https://www.dermis.net/bilder/CD050/550px/img0081.jpg
https://www.dermis.net/bilder/CD021/550px/img0086.jpg
https://www.dermis.net/bilder/CD056/550px/img0015.jpg
https://www.dermis.net/bilder/CD047/550px/img0084.jpg
https://www.dermis.net/bilder/CD042/550px/img0036.jpg
https://www.dermis.net/bilder/CD042/550px/img0037.jpg
https://www.dermis.net/bilder/CD006/550px/img0092.jpg
https://www.dermis.net/bilder/CD024/550px/img0051.jpg
https://www.dermis.net/bilder/CD004/550px/img0050.jpg

https://www.dermis.net/bilder/CD018/550px/img0012.jpg
https://www.dermis.net/bilder/CD052/550px/img0071.jpg
https://www.dermis.net/bilder/CD052/550px/img0072.jpg
https://www.dermis.net/bilder/CD024/550px/img0002.jpg
https://www.dermis.net/bilder/CD042/550px/img0040.jpg
https://www.dermis.net/bilder/CD036/550px/img0046.jpg
https://www.dermis.net/bilder/CD042/550px/img0041.jpg
https://www.dermis.net/bilder/CD039/550px/img0008.jpg
https://www.dermis.net/bilder/CD039/550px/img0009.jpg
https://www.dermis.net/bilder/CD018/550px/img0105.jpg
https://www.dermis.net/bilder/CD044/550px/img0052.jpg
https://www.dermis.net/bilder/CD015/550px/img0004.jpg
https://www.dermis.net/bilder/CD036/550px/img0035.jpg
https://www.dermis.net/bilder/CD035/550px/img0015.jpg
https://www.dermis.net/bilder/CD035/550px/img0014.jpg
https://www.dermis.net/bilder/CD006/550px/img0023.jpg
https://www.dermis.net/bilder/CD007/550px/img0053.jpg
https://www.dermis.net/bilder/CD008/550px/img0052.jpg
https://www.dermis.net/bilder/CD020/550px/img0017.jpg
https://www.dermis.net/bilder/CD033/550px/img0051.jpg
https://www.dermis.net/bilder/CD048/550px/img0022.jpg
https://www.dermis.net/bilder/CD033/550px/img0069.jpg
https://www.dermis.net/bilder/CD033/550px/img0070.jpg
https://www.dermis.net/bilder/CD018/550px/img0097.jpg
https://www.dermis.net/bilder/CD032/550px/img0065.jpg
https://www.dermis.net/bilder/CD032/550px/img0066.jpg
https://www.dermis.net/bilder/CD032/550px/img0067.jpg
https://www.dermis.net/bilder/CD048/550px/img0031.jpg
https://www.dermis.net/bilder/CD048/550px/img0032.jpg
https://www.dermis.net/bilder/CD048/550px/img0033.jpg
https://www.dermis.net/bilder/CD041/550px/img0030.jpg
https://www.dermis.net/bilder/CD024/550px/img0040.jpg
https://www.dermis.net/bilder/CD024/550px/img0039.jpg
https://www.dermis.net/bilder/CD057/550px/img0034.jpg
https://www.dermis.net/bilder/CD039/550px/img0099.jpg
https://www.dermis.net/bilder/CD016/550px/img0052.jpg
https://www.dermis.net/bilder/CD021/550px/img0088.jpg
https://www.dermis.net/bilder/CD005/550px/img0101.jpg
https://www.dermis.net/bilder/CD005/550px/img0083.jpg
https://www.dermis.net/bilder/CD046/550px/img0041.jpg
https://www.dermis.net/bilder/CD046/550px/img0042.jpg
https://www.dermis.net/bilder/CD046/550px/img0043.jpg
https://www.dermis.net/bilder/CD046/550px/img0044.jpg
https://www.dermis.net/bilder/CD039/550px/img0005.jpg
https://www.dermis.net/bilder/CD039/550px/img0006.jpg
https://www.dermis.net/bilder/CD017/550px/img0084.jpg
https://www.dermis.net/bilder/CD034/550px/img0054.jpg
https://www.dermis.net/bilder/CD051/550px/img0100.jpg

https://www.dermis.net/bilder/CD051/550px/img0101.jpg
https://www.dermis.net/bilder/CD020/550px/img0028.jpg
https://www.dermis.net/bilder/CD034/550px/img0052.jpg
https://www.dermis.net/bilder/CD034/550px/img0053.jpg
https://www.dermis.net/bilder/CD003/550px/img0094.jpg
https://www.dermis.net/bilder/CD026/550px/img0081.jpg
https://www.dermis.net/bilder/CD005/550px/img0060.jpg
https://www.dermis.net/bilder/CD034/550px/img0083.jpg
https://www.dermis.net/bilder/CD034/550px/img0084.jpg
https://www.dermis.net/bilder/CD032/550px/img0036.jpg
https://www.dermis.net/bilder/CD032/550px/img0037.jpg
https://www.dermis.net/bilder/CD024/550px/img0076.jpg
https://www.dermis.net/bilder/CD024/550px/img0077.jpg
https://www.dermis.net/bilder/CD024/550px/img0078.jpg
https://www.dermis.net/bilder/CD024/550px/img0079.jpg
https://www.dermis.net/bilder/CD024/550px/img0080.jpg
https://www.dermis.net/bilder/CD027/550px/img0098.jpg
https://www.dermis.net/bilder/CD006/550px/img0091.jpg
https://www.dermis.net/bilder/CD003/550px/img0010.jpg
https://www.dermis.net/bilder/CD020/550px/img0021.jpg
https://www.dermis.net/bilder/CD020/550px/img0022.jpg
https://www.dermis.net/bilder/CD049/550px/img0098.jpg
https://www.dermis.net/bilder/CD020/550px/img0023.jpg
https://www.dermis.net/bilder/CD058/550px/img0086.jpg
https://www.dermis.net/bilder/CD058/550px/img0087.jpg
https://www.dermis.net/bilder/CD008/550px/img0053.jpg
https://www.dermis.net/bilder/CD024/550px/img0011.jpg
https://www.dermis.net/bilder/CD024/550px/img0012.jpg
https://www.dermis.net/bilder/CD049/550px/img0081.jpg
https://www.dermis.net/bilder/CD049/550px/img0082.jpg
https://www.dermis.net/bilder/CD015/550px/img0006.jpg
https://www.dermis.net/bilder/CD003/550px/img0037.jpg
https://www.dermis.net/bilder/CD039/550px/img0045.jpg
https://www.dermis.net/bilder/CD039/550px/img0046.jpg
https://www.dermis.net/bilder/CD049/550px/img0072.jpg
https://www.dermis.net/bilder/CD033/550px/img0084.jpg
https://www.dermis.net/bilder/CD047/550px/img0002.jpg
https://www.dermis.net/bilder/CD047/550px/img0003.jpg
https://www.dermis.net/bilder/CD007/550px/img0051.jpg
https://www.dermis.net/bilder/CD049/550px/img0071.jpg
https://www.dermis.net/bilder/CD036/550px/img0043.jpg
https://www.dermis.net/bilder/CD041/550px/img0007.jpg
https://www.dermis.net/bilder/CD041/550px/img0009.jpg
https://www.dermis.net/bilder/CD037/550px/img0016.jpg
https://www.dermis.net/bilder/CD004/550px/img0077.jpg
https://www.dermis.net/bilder/CD050/550px/img0059.jpg
https://www.dermis.net/bilder/CD050/550px/img0060.jpg
https://www.dermis.net/bilder/CD003/550px/img0050.jpg

https://www.dermis.net/bilder/CD004/550px/img0066.jpg
https://www.dermis.net/bilder/CD037/550px/img0061.jpg
https://www.dermis.net/bilder/CD024/550px/img0045.jpg
https://www.dermis.net/bilder/CD027/550px/img0058.jpg
https://www.dermis.net/bilder/CD034/550px/img0086.jpg
https://www.dermis.net/bilder/CD034/550px/img0085.jpg
https://www.dermis.net/bilder/CD052/550px/img0038.jpg
https://www.dermis.net/bilder/CD050/550px/img0097.jpg
https://www.dermis.net/bilder/CD054/550px/img0087.jpg
https://www.dermis.net/bilder/CD051/550px/img0044.jpg
https://www.dermis.net/bilder/CD051/550px/img0045.jpg
https://www.dermis.net/bilder/CD051/550px/img0046.jpg
https://www.dermis.net/bilder/CD051/550px/img0047.jpg
https://www.dermis.net/bilder/CD051/550px/img0048.jpg
https://www.dermis.net/bilder/CD051/550px/img0030.jpg
https://www.dermis.net/bilder/CD023/550px/img0012.jpg
https://www.dermis.net/bilder/CD032/550px/img0078.jpg
https://www.dermis.net/bilder/CD051/550px/img0029.jpg
https://www.dermis.net/bilder/CD056/550px/img0014.jpg
https://www.dermis.net/bilder/CD003/550px/img0038.jpg
https://www.dermis.net/bilder/CD036/550px/img0002.jpg
https://www.dermis.net/bilder/CD004/550px/img0065.jpg
https://www.dermis.net/bilder/CD046/550px/img0064.jpg
https://www.dermis.net/bilder/CD017/550px/img0090.jpg
https://www.dermis.net/bilder/CD036/550px/img0003.jpg
https://www.dermis.net/bilder/CD016/550px/img0001.jpg
https://www.dermis.net/bilder/CD055/550px/img0031.jpg
https://www.dermis.net/bilder/CD049/550px/img0066.jpg
https://www.dermis.net/bilder/CD048/550px/img0066.jpg
https://www.dermis.net/bilder/CD048/550px/img0068.jpg
https://www.dermis.net/bilder/CD054/550px/img0056.jpg
https://www.dermis.net/bilder/CD054/550px/img0057.jpg
https://www.dermis.net/bilder/CD001/550px/img0040.jpg
https://www.dermis.net/bilder/CD041/550px/img0072.jpg
https://www.dermis.net/bilder/CD052/550px/img0037.jpg
https://www.dermis.net/bilder/CD050/550px/img0087.jpg
https://www.dermis.net/bilder/CD050/550px/img0088.jpg
https://www.dermis.net/bilder/CD048/550px/img0104.jpg
https://www.dermis.net/bilder/CD044/550px/img0001.jpg
https://www.dermis.net/bilder/CD003/550px/img0099.jpg
https://www.dermis.net/bilder/CD040/550px/img0063.jpg
https://www.dermis.net/bilder/CD036/550px/img0041.jpg
https://www.dermis.net/bilder/CD026/550px/img0071.jpg
https://www.dermis.net/bilder/CD026/550px/img0072.jpg
https://www.dermis.net/bilder/CD027/550px/img0073.jpg
https://www.dermis.net/bilder/CD027/550px/img0074.jpg
https://www.dermis.net/bilder/CD026/550px/img0077.jpg
https://www.dermis.net/bilder/CD028/550px/img0068.jpg

https://www.dermis.net/bilder/CD020/550px/img0034.jpg
https://www.dermis.net/bilder/CD020/550px/img0035.jpg
https://www.dermis.net/bilder/CD020/550px/img0036.jpg
https://www.dermis.net/bilder/CD033/550px/img0040.jpg
https://www.dermis.net/bilder/CD051/550px/img0038.jpg
https://www.dermis.net/bilder/CD051/550px/img0037.jpg
https://www.dermis.net/bilder/CD052/550px/img0043.jpg
https://www.dermis.net/bilder/CD051/550px/img0103.jpg
https://www.dermis.net/bilder/CD017/550px/img0102.jpg
https://www.dermis.net/bilder/CD003/550px/img0042.jpg
https://www.dermis.net/bilder/CD005/550px/img0026.jpg
https://www.dermis.net/bilder/CD003/550px/img0095.jpg
https://www.dermis.net/bilder/CD003/550px/img0020.jpg
https://www.dermis.net/bilder/CD042/550px/img0086.jpg
https://www.dermis.net/bilder/CD042/550px/img0088.jpg
https://www.dermis.net/bilder/CD087/550px/img0019.jpg
https://www.dermis.net/bilder/CD042/550px/img0091.jpg
https://www.dermis.net/bilder/CD212/550px/img0025.jpg
https://www.dermis.net/bilder/CD212/550px/img0114.jpg
https://www.dermis.net/bilder/CD211/550px/img0004.jpg
https://www.dermis.net/bilder/CD198/550px/img0071.jpg
https://www.dermis.net/bilder/CD036/550px/img0017.jpg
https://www.dermis.net/bilder/CD180/550px/img0072.jpg
https://www.dermis.net/bilder/CD197/550px/img0017.jpg
https://www.dermis.net/bilder/CD195/550px/img0098.jpg
https://www.dermis.net/bilder/CD195/550px/img0099.jpg
https://www.dermis.net/bilder/CD197/550px/img0036.jpg
https://www.dermis.net/bilder/CD042/550px/img0022.jpg
https://www.dermis.net/bilder/CD179/550px/img0028.jpg
https://www.dermis.net/bilder/CD180/550px/img0034.jpg
https://www.dermis.net/bilder/CD032/550px/img0039.jpg
https://www.dermis.net/bilder/CD197/550px/img0024.jpg
https://www.dermis.net/bilder/CD025/550px/img0073.jpg
https://www.dermis.net/bilder/CD179/550px/img0027.jpg
https://www.dermis.net/bilder/CD180/550px/img0071.jpg
https://www.dermis.net/bilder/CD007/550px/img0110.jpg
https://www.dermis.net/bilder/CD178/550px/img0036.jpg
https://www.dermis.net/bilder/CD193/550px/img0149.jpg
https://www.dermis.net/bilder/CD193/550px/img0150.jpg
https://www.dermis.net/bilder/CD193/550px/img0151.jpg
https://www.dermis.net/bilder/CD043/550px/img0026.jpg
https://www.dermis.net/bilder/CD191/550px/img0113.jpg
https://www.dermis.net/bilder/CD184/550px/img0091.jpg
https://www.dermis.net/bilder/CD197/550px/img0014.jpg
https://www.dermis.net/bilder/CD016/550px/img0057.jpg
https://www.dermis.net/bilder/CD041/550px/img0037.jpg
https://www.dermis.net/bilder/CD041/550px/img0033.jpg
https://www.dermis.net/bilder/CD184/550px/img0002.jpg

https://www.dermis.net/bilder/CD211/550px/img0003.jpg
https://www.dermis.net/bilder/CD033/550px/img0071.jpg
https://www.dermis.net/bilder/CD196/550px/img0141.jpg
https://www.dermis.net/bilder/CD017/550px/img0082.jpg
https://www.dermis.net/bilder/CD042/550px/img0085.jpg
https://www.dermis.net/bilder/CD035/550px/img0080.jpg
https://www.dermis.net/bilder/CD197/550px/img0013.jpg
https://www.dermis.net/bilder/CD040/550px/img0070.jpg
https://www.dermis.net/bilder/CD041/550px/img0061.jpg
https://www.dermis.net/bilder/CD178/550px/img0088.jpg
https://www.dermis.net/bilder/CD178/550px/img0089.jpg
https://www.dermis.net/bilder/CD196/550px/img0045.jpg
https://www.dermis.net/bilder/CD054/550px/img0004.jpg
https://www.dermis.net/bilder/CD008/550px/img0076.jpg
https://www.dermis.net/bilder/CD199/550px/img0143.jpg
https://www.dermis.net/bilder/CD197/550px/img0023.jpg
https://www.dermis.net/bilder/CD197/550px/img0026.jpg
https://www.dermis.net/bilder/CD052/550px/img0093.jpg
https://www.dermis.net/bilder/CD025/550px/img0068.jpg
https://www.dermis.net/bilder/CD055/550px/img0045.jpg
https://www.dermis.net/bilder/CD179/550px/img0026.jpg
https://www.dermis.net/bilder/CD195/550px/img0010.jpg
https://www.dermis.net/bilder/CD197/550px/img0016.jpg
https://www.dermis.net/bilder/CD043/550px/img0027.jpg
https://www.dermis.net/bilder/CD184/550px/img0024.jpg
https://www.dermis.net/bilder/CD184/550px/img0025.jpg
https://www.dermis.net/bilder/CD041/550px/img0043.jpg
https://www.dermis.net/bilder/CD041/550px/img0044.jpg
https://www.dermis.net/bilder/CD189/550px/img0067.jpg
https://www.dermis.net/bilder/CD042/550px/img0031.jpg
https://www.dermis.net/bilder/CD011/550px/img0100.jpg
https://www.dermis.net/bilder/CD021/550px/img0099.jpg
https://www.dermis.net/bilder/CD199/550px/img0145.jpg
https://www.dermis.net/bilder/CD199/550px/img0146.jpg
https://www.dermis.net/bilder/CD199/550px/img0122.jpg
https://www.dermis.net/bilder/CD199/550px/img0061.jpg
https://www.dermis.net/bilder/CD001/550px/img0025.jpg
https://www.dermis.net/bilder/CD196/550px/img0117.jpg
https://www.dermis.net/bilder/CD199/550px/img0144.jpg
https://www.dermis.net/bilder/CD012/550px/img0082.jpg
https://www.dermis.net/bilder/CD187/550px/img0013.jpg
https://www.dermis.net/bilder/CD187/550px/img0014.jpg
https://www.dermis.net/bilder/CD196/550px/img0119.jpg
https://www.dermis.net/bilder/CD041/550px/img0067.jpg
https://www.dermis.net/bilder/CD180/550px/img0082.jpg
https://www.dermis.net/bilder/CD040/550px/img0069.jpg
https://www.dermis.net/bilder/CD191/550px/img0040.jpg
https://www.dermis.net/bilder/CD191/550px/img0041.jpg

https://www.dermis.net/bilder/CD210/550px/img0114.jpg
https://www.dermis.net/bilder/CD193/550px/img0093.jpg
https://www.dermis.net/bilder/CD199/550px/img0001.jpg
https://www.dermis.net/bilder/CD199/550px/img0129.jpg
https://www.dermis.net/bilder/CD192/550px/img0026.jpg
https://www.dermis.net/bilder/CD032/550px/img0049.jpg
https://www.dermis.net/bilder/CD181/550px/img0062.jpg
https://www.dermis.net/bilder/CD012/550px/img0028.jpg
https://www.dermis.net/bilder/CD027/550px/img0031.jpg
https://www.dermis.net/bilder/CD021/550px/img0081.jpg
https://www.dermis.net/bilder/CD181/550px/img0087.jpg
https://www.dermis.net/bilder/CD041/550px/img0046.jpg
https://www.dermis.net/bilder/CD196/550px/img0044.jpg
https://www.dermis.net/bilder/CD195/550px/img0073.jpg
https://www.dermis.net/bilder/CD196/550px/img0038.jpg
https://www.dermis.net/bilder/CD196/550px/img0039.jpg
https://www.dermis.net/bilder/CD054/550px/img0006.jpg
https://www.dermis.net/bilder/CD008/550px/img0083.jpg
https://www.dermis.net/bilder/CD182/550px/img0064.jpg
https://www.dermis.net/bilder/CD002/550px/img0065.jpg
https://www.dermis.net/bilder/CD041/550px/img0048.jpg
https://www.dermis.net/bilder/CD197/550px/img0081.jpg
https://www.dermis.net/bilder/CD197/550px/img0082.jpg
https://www.dermis.net/bilder/CD199/550px/img0135.jpg
https://www.dermis.net/bilder/CD041/550px/img0047.jpg
https://www.dermis.net/bilder/CD040/550px/img0011.jpg
https://www.dermis.net/bilder/CD194/550px/img0154.jpg
https://www.dermis.net/bilder/CD189/550px/img0093.jpg
https://www.dermis.net/bilder/CD027/550px/img0039.jpg
https://www.dermis.net/bilder/CD005/550px/img0090.jpg
https://www.dermis.net/bilder/CD191/550px/img0016.jpg
https://www.dermis.net/bilder/CD191/550px/img0017.jpg
https://www.dermis.net/bilder/CD212/550px/img0049.jpg
https://www.dermis.net/bilder/CD178/550px/img0014.jpg
https://www.dermis.net/bilder/CD042/550px/img0010.jpg
https://www.dermis.net/bilder/CD182/550px/img0040.jpg
https://www.dermis.net/bilder/CD006/550px/img0061.jpg
https://www.dermis.net/bilder/CD056/550px/img0063.jpg
https://www.dermis.net/bilder/CD187/550px/img0033.jpg
https://www.dermis.net/bilder/CD196/550px/img0107.jpg
https://www.dermis.net/bilder/CD196/550px/img0110.jpg
https://www.dermis.net/bilder/CD027/550px/img0028.jpg
https://www.dermis.net/bilder/CD027/550px/img0029.jpg
https://www.dermis.net/bilder/CD032/550px/img0038.jpg
https://www.dermis.net/bilder/CD180/550px/img0029.jpg
https://www.dermis.net/bilder/CD041/550px/img0086.jpg
https://www.dermis.net/bilder/CD197/550px/img0039.jpg
https://www.dermis.net/bilder/CD197/550px/img0040.jpg

https://www.dermis.net/bilder/CD197/550px/img0041.jpg
https://www.dermis.net/bilder/CD194/550px/img0060.jpg
https://www.dermis.net/bilder/CD197/550px/img0007.jpg
https://www.dermis.net/bilder/CD185/550px/img0054.jpg
https://www.dermis.net/bilder/CD202/550px/img0021.jpg
https://www.dermis.net/bilder/CD181/550px/img0086.jpg
https://www.dermis.net/bilder/CD003/550px/img0100.jpg
https://www.dermis.net/bilder/CD041/550px/img0038.jpg
https://www.dermis.net/bilder/CD041/550px/img0039.jpg
https://www.dermis.net/bilder/CD041/550px/img0040.jpg
https://www.dermis.net/bilder/CD021/550px/img0036.jpg
https://www.dermis.net/bilder/CD198/550px/img0185.jpg
https://www.dermis.net/bilder/CD008/550px/img0079.jpg
https://www.dermis.net/bilder/CD193/550px/img0042.jpg
https://www.dermis.net/bilder/CD189/550px/img0004.jpg
https://www.dermis.net/bilder/CD189/550px/img0005.jpg
https://www.dermis.net/bilder/CD197/550px/img0080.jpg
https://www.dermis.net/bilder/CD194/550px/img0116.jpg
https://www.dermis.net/bilder/CD196/550px/img0170.jpg
https://www.dermis.net/bilder/CD196/550px/img0171.jpg
https://www.dermis.net/bilder/CD195/550px/img0112.jpg
https://www.dermis.net/bilder/CD195/550px/img0113.jpg
https://www.dermis.net/bilder/CD014/550px/img0079.jpg
https://www.dermis.net/bilder/CD013/550px/img0017.jpg
https://www.dermis.net/bilder/CD022/550px/img0003.jpg
https://www.dermis.net/bilder/CD042/550px/img0023.jpg
https://www.dermis.net/bilder/CD178/550px/img0096.jpg
https://www.dermis.net/bilder/CD178/550px/img0097.jpg
https://www.dermis.net/bilder/CD178/550px/img0098.jpg
https://www.dermis.net/bilder/CD178/550px/img0099.jpg
https://www.dermis.net/bilder/CD003/550px/img0079.jpg
https://www.dermis.net/bilder/CD181/550px/img0007.jpg
https://www.dermis.net/bilder/CD181/550px/img0006.jpg
https://www.dermis.net/bilder/CD041/550px/img0084.jpg
https://www.dermis.net/bilder/CD194/550px/img0153.jpg
https://www.dermis.net/bilder/CD196/550px/img0139.jpg
https://www.dermis.net/bilder/CD196/550px/img0140.jpg
https://www.dermis.net/bilder/CD197/550px/img0037.jpg
https://www.dermis.net/bilder/CD198/550px/img0139.jpg
https://www.dermis.net/bilder/CD036/550px/img0074.jpg
https://www.dermis.net/bilder/CD212/550px/img0056.jpg
https://www.dermis.net/bilder/CD210/550px/img0020.jpg
https://www.dermis.net/bilder/CD035/550px/img0030.jpg
https://www.dermis.net/bilder/CD035/550px/img0032.jpg
https://www.dermis.net/bilder/CD016/550px/img0056.jpg
https://www.dermis.net/bilder/CD027/550px/img0038.jpg
https://www.dermis.net/bilder/CD027/550px/img0040.jpg
https://www.dermis.net/bilder/CD180/550px/img0036.jpg

https://www.dermis.net/bilder/CD180/550px/img0035.jpg
https://www.dermis.net/bilder/CD180/550px/img0037.jpg
https://www.dermis.net/bilder/CD194/550px/img0041.jpg
https://www.dermis.net/bilder/CD194/550px/img0042.jpg
https://www.dermis.net/bilder/CD194/550px/img0043.jpg
https://www.dermis.net/bilder/CD195/550px/img0094.jpg
https://www.dermis.net/bilder/CD043/550px/img0040.jpg
https://www.dermis.net/bilder/CD184/550px/img0087.jpg
https://www.dermis.net/bilder/CD210/550px/img0152.jpg
https://www.dermis.net/bilder/CD210/550px/img0153.jpg
https://www.dermis.net/bilder/CD201/550px/img0012.jpg
https://www.dermis.net/bilder/CD201/550px/img0013.jpg
https://www.dermis.net/bilder/CD195/550px/img0030.jpg
https://www.dermis.net/bilder/CD036/550px/img0051.jpg
https://www.dermis.net/bilder/CD185/550px/img0068.jpg
https://www.dermis.net/bilder/CD185/550px/img0069.jpg
https://www.dermis.net/bilder/CD185/550px/img0072.jpg
https://www.dermis.net/bilder/CD194/550px/img0046.jpg
https://www.dermis.net/bilder/CD194/550px/img0047.jpg
https://www.dermis.net/bilder/CD194/550px/img0048.jpg
https://www.dermis.net/bilder/CD027/550px/img0027.jpg
https://www.dermis.net/bilder/CD032/550px/img0042.jpg
https://www.dermis.net/bilder/CD197/550px/img0027.jpg
https://www.dermis.net/bilder/CD180/550px/img0032.jpg
https://www.dermis.net/bilder/CD033/550px/img0017.jpg
https://www.dermis.net/bilder/CD021/550px/img0060.jpg
https://www.dermis.net/bilder/CD192/550px/img0097.jpg
https://www.dermis.net/bilder/CD192/550px/img0096.jpg
https://www.dermis.net/bilder/CD183/550px/img0009.jpg
https://www.dermis.net/bilder/CD183/550px/img0010.jpg
https://www.dermis.net/bilder/CD194/550px/img0089.jpg
https://www.dermis.net/bilder/CD194/550px/img0090.jpg
https://www.dermis.net/bilder/CD194/550px/img0091.jpg
https://www.dermis.net/bilder/CD179/550px/img0014.jpg
https://www.dermis.net/bilder/CD190/550px/img0024.jpg
https://www.dermis.net/bilder/CD190/550px/img0026.jpg
https://www.dermis.net/bilder/CD190/550px/img0027.jpg
https://www.dermis.net/bilder/CD190/550px/img0008.jpg
https://www.dermis.net/bilder/CD190/550px/img0009.jpg
https://www.dermis.net/bilder/CD190/550px/img0010.jpg
https://www.dermis.net/bilder/CD212/550px/img0067.jpg
https://www.dermis.net/bilder/CD193/550px/img0174.jpg
https://www.dermis.net/bilder/CD193/550px/img0175.jpg
https://www.dermis.net/bilder/CD212/550px/img0007.jpg
https://www.dermis.net/bilder/CD198/550px/img0174.jpg
https://www.dermis.net/bilder/CD178/550px/img0034.jpg
https://www.dermis.net/bilder/CD178/550px/img0035.jpg
https://www.dermis.net/bilder/CD193/550px/img0051.jpg

https://www.dermis.net/bilder/CD201/550px/img0024.jpg
https://www.dermis.net/bilder/CD199/550px/img0015.jpg
https://www.dermis.net/bilder/CD199/550px/img0016.jpg
https://www.dermis.net/bilder/CD193/550px/img0130.jpg
https://www.dermis.net/bilder/CD005/550px/img0011.jpg
https://www.dermis.net/bilder/CD033/550px/img0047.jpg
https://www.dermis.net/bilder/CD035/550px/img0074.jpg
https://www.dermis.net/bilder/CD042/550px/img0008.jpg
https://www.dermis.net/bilder/CD042/550px/img0001.jpg
https://www.dermis.net/bilder/CD042/550px/img0002.jpg
https://www.dermis.net/bilder/CD021/550px/img0041.jpg
https://www.dermis.net/bilder/CD193/550px/img0195.jpg
https://www.dermis.net/bilder/CD194/550px/img0152.jpg
https://www.dermis.net/bilder/CD194/550px/img0151.jpg
https://www.dermis.net/bilder/CD012/550px/img0044.jpg
https://www.dermis.net/bilder/CD187/550px/img0011.jpg
https://www.dermis.net/bilder/CD187/550px/img0012.jpg
https://www.dermis.net/bilder/CD196/550px/img0054.jpg
https://www.dermis.net/bilder/CD196/550px/img0055.jpg
https://www.dermis.net/bilder/CD196/550px/img0056.jpg
https://www.dermis.net/bilder/CD196/550px/img0166.jpg
https://www.dermis.net/bilder/CD198/550px/img0118.jpg
https://www.dermis.net/bilder/CD183/550px/img0032.jpg
https://www.dermis.net/bilder/CD183/550px/img0033.jpg
https://www.dermis.net/bilder/CD193/550px/img0040.jpg
https://www.dermis.net/bilder/CD193/550px/img0041.jpg
https://www.dermis.net/bilder/CD193/550px/img0043.jpg
https://www.dermis.net/bilder/CD021/550px/img0083.jpg
https://www.dermis.net/bilder/CD195/550px/img0023.jpg
https://www.dermis.net/bilder/CD193/550px/img0169.jpg
https://www.dermis.net/bilder/CD193/550px/img0171.jpg
https://www.dermis.net/bilder/CD201/550px/img0004.jpg
https://www.dermis.net/bilder/CD188/550px/img0012.jpg
https://www.dermis.net/bilder/CD194/550px/img0130.jpg
https://www.dermis.net/bilder/CD195/550px/img0095.jpg
https://www.dermis.net/bilder/CD210/550px/img0044.jpg
https://www.dermis.net/bilder/CD051/550px/img0003.jpg
https://www.dermis.net/bilder/CD194/550px/img0118.jpg
https://www.dermis.net/bilder/CD192/550px/img0050.jpg
https://www.dermis.net/bilder/CD043/550px/img0025.jpg
https://www.dermis.net/bilder/CD195/550px/img0076.jpg
https://www.dermis.net/bilder/CD195/550px/img0077.jpg
https://www.dermis.net/bilder/CD195/550px/img0078.jpg
https://www.dermis.net/bilder/CD195/550px/img0079.jpg
https://www.dermis.net/bilder/CD199/550px/img0089.jpg
https://www.dermis.net/bilder/CD041/550px/img0069.jpg
https://www.dermis.net/bilder/CD003/550px/img0098.jpg
https://www.dermis.net/bilder/CD042/550px/img0046.jpg

https://www.dermis.net/bilder/CD055/550px/img0021.jpg
https://www.dermis.net/bilder/CD055/550px/img0022.jpg
https://www.dermis.net/bilder/CD191/550px/img0034.jpg
https://www.dermis.net/bilder/CD191/550px/img0035.jpg
https://www.dermis.net/bilder/CD212/550px/img0120.jpg
https://www.dermis.net/bilder/CD042/550px/img0015.jpg
https://www.dermis.net/bilder/CD199/550px/img0062.jpg
https://www.dermis.net/bilder/CD045/550px/img0050.jpg
https://www.dermis.net/bilder/CD042/550px/img0047.jpg
https://www.dermis.net/bilder/CD197/550px/img0015.jpg
https://www.dermis.net/bilder/CD193/550px/img0061.jpg
https://www.dermis.net/bilder/CD193/550px/img0062.jpg
https://www.dermis.net/bilder/CD027/550px/img0030.jpg
https://www.dermis.net/bilder/CD197/550px/img0025.jpg
https://www.dermis.net/bilder/CD021/550px/img0064.jpg
https://www.dermis.net/bilder/CD181/550px/img0046.jpg
https://www.dermis.net/bilder/CD181/550px/img0047.jpg
https://www.dermis.net/bilder/CD181/550px/img0048.jpg
https://www.dermis.net/bilder/CD193/550px/img0181.jpg
https://www.dermis.net/bilder/CD193/550px/img0180.jpg
https://www.dermis.net/bilder/CD052/550px/img0052.jpg
https://www.dermis.net/bilder/CD036/550px/img0079.jpg
https://www.dermis.net/bilder/CD193/550px/img0088.jpg
https://www.dermis.net/bilder/CD193/550px/img0089.jpg
https://www.dermis.net/bilder/CD193/550px/img0090.jpg
https://www.dermis.net/bilder/CD193/550px/img0092.jpg
https://www.dermis.net/bilder/CD199/550px/img0107.jpg
https://www.dermis.net/bilder/CD211/550px/img0016.jpg
https://www.dermis.net/bilder/CD184/550px/img0023.jpg
https://www.dermis.net/bilder/CD002/550px/img0097.jpg
https://www.dermis.net/bilder/CD021/550px/img0004.jpg
https://www.dermis.net/bilder/CD189/550px/img0065.jpg
https://www.dermis.net/bilder/CD189/550px/img0068.jpg
https://www.dermis.net/bilder/CD055/550px/img0040.jpg
https://www.dermis.net/bilder/CD180/550px/img0018.jpg
https://www.dermis.net/bilder/CD012/550px/img0066.jpg
https://www.dermis.net/bilder/CD190/550px/img0035.jpg
https://www.dermis.net/bilder/CD191/550px/img0009.jpg
https://www.dermis.net/bilder/CD198/550px/img0135.jpg
https://www.dermis.net/bilder/CD198/550px/img0136.jpg
https://www.dermis.net/bilder/CD004/550px/img0072.jpg
https://www.dermis.net/bilder/CD044/550px/img0084.jpg
https://www.dermis.net/bilder/CD021/550px/img0080.jpg
https://www.dermis.net/bilder/CD017/550px/img0093.jpg
https://www.dermis.net/bilder/CD195/550px/img0088.jpg
https://www.dermis.net/bilder/CD069/550px/img0104.jpg
https://www.dermis.net/bilder/CD033/550px/img0018.jpg
https://www.dermis.net/bilder/CD194/550px/img0087.jpg

https://www.dermis.net/bilder/CD194/550px/img0088.jpg
https://www.dermis.net/bilder/CD188/550px/img0024.jpg
https://www.dermis.net/bilder/CD191/550px/img0036.jpg
https://www.dermis.net/bilder/CD191/550px/img0037.jpg
https://www.dermis.net/bilder/CD196/550px/img0118.jpg
https://www.dermis.net/bilder/CD189/550px/img0107.jpg
https://www.dermis.net/bilder/CD189/550px/img0108.jpg
https://www.dermis.net/bilder/CD189/550px/img0109.jpg
https://www.dermis.net/bilder/CD033/550px/img0045.jpg
https://www.dermis.net/bilder/CD184/550px/img0026.jpg
https://www.dermis.net/bilder/CD039/550px/img0096.jpg
https://www.dermis.net/bilder/CD182/550px/img0070.jpg
https://www.dermis.net/bilder/CD182/550px/img0074.jpg
https://www.dermis.net/bilder/CD182/550px/img0073.jpg
https://www.dermis.net/bilder/CD182/550px/img0072.jpg
https://www.dermis.net/bilder/CD182/550px/img0071.jpg
https://www.dermis.net/bilder/CD182/550px/img0069.jpg
https://www.dermis.net/bilder/CD043/550px/img0029.jpg
https://www.dermis.net/bilder/CD043/550px/img0031.jpg
https://www.dermis.net/bilder/CD192/550px/img0058.jpg
https://www.dermis.net/bilder/CD040/550px/img0055.jpg
https://www.dermis.net/bilder/CD193/550px/img0075.jpg
https://www.dermis.net/bilder/CD194/550px/img0001.jpg
https://www.dermis.net/bilder/CD194/550px/img0002.jpg
https://www.dermis.net/bilder/CD180/550px/img0073.jpg
https://www.dermis.net/bilder/CD180/550px/img0074.jpg
https://www.dermis.net/bilder/CD004/550px/img0044.jpg
https://www.dermis.net/bilder/CD021/550px/img0039.jpg
https://www.dermis.net/bilder/CD192/550px/img0027.jpg
https://www.dermis.net/bilder/CD192/550px/img0028.jpg
https://www.dermis.net/bilder/CD212/550px/img0131.jpg
https://www.dermis.net/bilder/CD189/550px/img0091.jpg
https://www.dermis.net/bilder/CD027/550px/img0036.jpg
https://www.dermis.net/bilder/CD027/550px/img0037.jpg
https://www.dermis.net/bilder/CD013/550px/img0019.jpg
https://www.dermis.net/bilder/CD179/550px/img0007.jpg
https://www.dermis.net/bilder/CD179/550px/img0008.jpg
https://www.dermis.net/bilder/CD193/550px/img0029.jpg
https://www.dermis.net/bilder/CD042/550px/img0066.jpg
https://www.dermis.net/bilder/CD184/550px/img0086.jpg
https://www.dermis.net/bilder/CD184/550px/img0064.jpg
https://www.dermis.net/bilder/CD180/550px/img0007.jpg
https://www.dermis.net/bilder/CD199/550px/img0023.jpg
https://www.dermis.net/bilder/CD195/550px/img0002.jpg
https://www.dermis.net/bilder/CD195/550px/img0005.jpg
https://www.dermis.net/bilder/CD198/550px/img0110.jpg
https://www.dermis.net/bilder/CD212/550px/img0122.jpg
https://www.dermis.net/bilder/CD024/550px/img0083.jpg

https://www.dermis.net/bilder/CD024/550px/img0084.jpg
https://www.dermis.net/bilder/CD199/550px/img0096.jpg
https://www.dermis.net/bilder/CD210/550px/img0027.jpg
https://www.dermis.net/bilder/CD033/550px/img0035.jpg
https://www.dermis.net/bilder/CD180/550px/img0033.jpg
https://www.dermis.net/bilder/CD211/550px/img0002.jpg
https://www.dermis.net/bilder/CD189/550px/img0060.jpg
https://www.dermis.net/bilder/CD189/550px/img0062.jpg
https://www.dermis.net/bilder/CD035/550px/img0071.jpg
https://www.dermis.net/bilder/CD012/550px/img0093.jpg
https://www.dermis.net/bilder/CD198/550px/img0168.jpg
https://www.dermis.net/bilder/CD198/550px/img0170.jpg
https://www.dermis.net/bilder/CD198/550px/img0171.jpg
https://www.dermis.net/bilder/CD199/550px/img0069.jpg
https://www.dermis.net/bilder/CD199/550px/img0070.jpg
https://www.dermis.net/bilder/CD199/550px/img0074.jpg
https://www.dermis.net/bilder/CD182/550px/img0066.jpg
https://www.dermis.net/bilder/CD178/550px/img0033.jpg
https://www.dermis.net/bilder/CD185/550px/img0050.jpg
https://www.dermis.net/bilder/CD185/550px/img0051.jpg
https://www.dermis.net/bilder/CD211/550px/img0062.jpg
https://www.dermis.net/bilder/CD211/550px/img0042.jpg
https://www.dermis.net/bilder/CD042/550px/img0058.jpg
https://www.dermis.net/bilder/CD042/550px/img0059.jpg
https://www.dermis.net/bilder/CD193/550px/img0044.jpg
https://www.dermis.net/bilder/CD193/550px/img0045.jpg
https://www.dermis.net/bilder/CD192/550px/img0074.jpg
https://www.dermis.net/bilder/CD181/550px/img0084.jpg
https://www.dermis.net/bilder/CD025/550px/img0025.jpg
https://www.dermis.net/bilder/CD198/550px/img0184.jpg
https://www.dermis.net/bilder/CD056/550px/img0092.jpg
https://www.dermis.net/bilder/CD195/550px/img0020.jpg
https://www.dermis.net/bilder/CD195/550px/img0021.jpg
https://www.dermis.net/bilder/CD185/550px/img0042.jpg
https://www.dermis.net/bilder/CD185/550px/img0043.jpg
https://www.dermis.net/bilder/CD193/550px/img0146.jpg
https://www.dermis.net/bilder/CD194/550px/img0117.jpg
https://www.dermis.net/bilder/CD195/550px/img0114.jpg
https://www.dermis.net/bilder/CD041/550px/img0071.jpg
https://www.dermis.net/bilder/CD041/550px/img0081.jpg
https://www.dermis.net/bilder/CD036/550px/img0034.jpg
https://www.dermis.net/bilder/CD046/550px/img0045.jpg
https://www.dermis.net/bilder/CD004/550px/img0029.jpg
https://www.dermis.net/bilder/CD024/550px/img0033.jpg
https://www.dermis.net/bilder/CD006/550px/img0030.jpg
https://www.dermis.net/bilder/CD017/550px/img0094.jpg
https://www.dermis.net/bilder/CD036/550px/img0004.jpg
https://www.dermis.net/bilder/CD073/550px/img0002.jpg

https://www.dermis.net/bilder/CD211/550px/img0080.jpg
https://www.dermis.net/bilder/CD002/550px/img0095.jpg
https://www.dermis.net/bilder/CD012/550px/img0083.jpg
https://www.dermis.net/bilder/CD034/550px/img0057.jpg
https://www.dermis.net/bilder/CD034/550px/img0058.jpg
https://www.dermis.net/bilder/CD028/550px/img0002.jpg
https://www.dermis.net/bilder/CD014/550px/img0086.jpg
https://www.dermis.net/bilder/CD041/550px/img0050.jpg
https://www.dermis.net/bilder/CD016/550px/img0037.jpg
https://www.dermis.net/bilder/CD003/550px/img0031.jpg
https://www.dermis.net/bilder/CD069/550px/img0098.jpg
https://www.dermis.net/bilder/CD016/550px/img0033.jpg
https://www.dermis.net/bilder/CD025/550px/img0095.jpg
https://www.dermis.net/bilder/CD018/550px/img0043.jpg
https://www.dermis.net/bilder/CD018/550px/img0019.jpg
https://www.dermis.net/bilder/CD054/550px/img0011.jpg
https://www.dermis.net/bilder/CD016/550px/img0041.jpg
https://www.dermis.net/bilder/CD014/550px/img0092.jpg
https://www.dermis.net/bilder/CD054/550px/img0031.jpg
https://www.dermis.net/bilder/CD016/550px/img0062.jpg
https://www.dermis.net/bilder/CD034/550px/img0095.jpg
https://www.dermis.net/bilder/CD055/550px/img0090.jpg
https://www.dermis.net/bilder/CD055/550px/img0091.jpg
https://www.dermis.net/bilder/CD023/550px/img0033.jpg
https://www.dermis.net/bilder/CD027/550px/img0092.jpg
https://www.dermis.net/bilder/CD018/550px/img0044.jpg
https://www.dermis.net/bilder/CD023/550px/img0080.jpg
https://www.dermis.net/bilder/CD046/550px/img0046.jpg
https://www.dermis.net/bilder/CD039/550px/img0052.jpg
https://www.dermis.net/bilder/CD026/550px/img0006.jpg
https://www.dermis.net/bilder/CD023/550px/img0062.jpg
https://www.dermis.net/bilder/CD023/550px/img0063.jpg
https://www.dermis.net/bilder/CD033/550px/img0099.jpg
https://www.dermis.net/bilder/CD033/550px/img0100.jpg
https://www.dermis.net/bilder/CD036/550px/img0091.jpg
https://www.dermis.net/bilder/CD052/550px/img0042.jpg
https://www.dermis.net/bilder/CD003/550px/img0015.jpg
https://www.dermis.net/bilder/CD025/550px/img0085.jpg
https://www.dermis.net/bilder/CD037/550px/img0063.jpg
https://www.dermis.net/bilder/CD037/550px/img0064.jpg
https://www.dermis.net/bilder/CD014/550px/img0093.jpg
https://www.dermis.net/bilder/CD051/550px/img0085.jpg
https://www.dermis.net/bilder/CD018/550px/img0086.jpg
https://www.dermis.net/bilder/CD040/550px/img0035.jpg
https://www.dermis.net/bilder/CD052/550px/img0066.jpg
https://www.dermis.net/bilder/CD055/550px/img0088.jpg
https://www.dermis.net/bilder/CD052/550px/img0085.jpg
https://www.dermis.net/bilder/CD015/550px/img0038.jpg

https://www.dermis.net/bilder/CD016/550px/img0040.jpg
https://www.dermis.net/bilder/CD037/550px/img0050.jpg
https://www.dermis.net/bilder/CD054/550px/img0107.jpg
https://www.dermis.net/bilder/CD018/550px/img0056.jpg
https://www.dermis.net/bilder/CD024/550px/img0099.jpg
https://www.dermis.net/bilder/CD054/550px/img0093.jpg
https://www.dermis.net/bilder/CD005/550px/img0043.jpg
https://www.dermis.net/bilder/CD034/550px/img0030.jpg
https://www.dermis.net/bilder/CD016/550px/img0044.jpg
https://www.dermis.net/bilder/CD007/550px/img0056.jpg
https://www.dermis.net/bilder/CD007/550px/img0021.jpg
https://www.dermis.net/bilder/CD025/550px/img0090.jpg
https://www.dermis.net/bilder/CD007/550px/img0020.jpg
https://www.dermis.net/bilder/CD057/550px/img0014.jpg
https://www.dermis.net/bilder/CD017/550px/img0080.jpg
https://www.dermis.net/bilder/CD016/550px/img0036.jpg
https://www.dermis.net/bilder/CD014/550px/img0053.jpg
https://www.dermis.net/bilder/CD006/550px/img0025.jpg
https://www.dermis.net/bilder/CD047/550px/img0016.jpg
https://www.dermis.net/bilder/CD039/550px/img0021.jpg
https://www.dermis.net/bilder/CD050/550px/img0004.jpg
https://www.dermis.net/bilder/CD052/550px/img0082.jpg
https://www.dermis.net/bilder/CD047/550px/img0081.jpg
https://www.dermis.net/bilder/CD016/550px/img0038.jpg
https://www.dermis.net/bilder/CD050/550px/img0086.jpg
https://www.dermis.net/bilder/CD048/550px/img0013.jpg
https://www.dermis.net/bilder/CD040/550px/img0089.jpg
https://www.dermis.net/bilder/CD040/550px/img0090.jpg
https://www.dermis.net/bilder/CD052/550px/img0081.jpg
https://www.dermis.net/bilder/CD024/550px/img0056.jpg
https://www.dermis.net/bilder/CD047/550px/img0007.jpg
https://www.dermis.net/bilder/CD040/550px/img0078.jpg
https://www.dermis.net/bilder/CD047/550px/img0014.jpg
https://www.dermis.net/bilder/CD047/550px/img0015.jpg
https://www.dermis.net/bilder/CD057/550px/img0004.jpg
https://www.dermis.net/bilder/CD015/550px/img0035.jpg
https://www.dermis.net/bilder/CD039/550px/img0056.jpg
https://www.dermis.net/bilder/CD005/550px/img0094.jpg
https://www.dermis.net/bilder/CD020/550px/img0048.jpg
https://www.dermis.net/bilder/CD006/550px/img0095.jpg
https://www.dermis.net/bilder/CD043/550px/img0042.jpg
https://www.dermis.net/bilder/CD023/550px/img0011.jpg
https://www.dermis.net/bilder/CD007/550px/img0013.jpg
https://www.dermis.net/bilder/CD048/550px/img0044.jpg
https://www.dermis.net/bilder/CD048/550px/img0045.jpg
https://www.dermis.net/bilder/CD048/550px/img0046.jpg
https://www.dermis.net/bilder/CD046/550px/img0059.jpg
https://www.dermis.net/bilder/CD026/550px/img0073.jpg

https://www.dermis.net/bilder/CD026/550px/img0074.jpg
https://www.dermis.net/bilder/CD025/550px/img0080.jpg
https://www.dermis.net/bilder/CD006/550px/img0063.jpg
https://www.dermis.net/bilder/CD052/550px/img0039.jpg
https://www.dermis.net/bilder/CD052/550px/img0040.jpg
https://www.dermis.net/bilder/CD016/550px/img0042.jpg
https://www.dermis.net/bilder/CD178/550px/img0107.jpg
https://www.dermis.net/bilder/CD178/550px/img0018.jpg
https://www.dermis.net/bilder/CD056/550px/img0062.jpg
https://www.dermis.net/bilder/CD181/550px/img0036.jpg
https://www.dermis.net/bilder/CD196/550px/img0097.jpg
https://www.dermis.net/bilder/CD196/550px/img0098.jpg
https://www.dermis.net/bilder/CD199/550px/img0095.jpg
https://www.dermis.net/bilder/CD212/550px/img0038.jpg
https://www.dermis.net/bilder/CD212/550px/img0040.jpg
https://www.dermis.net/bilder/CD193/550px/img0021.jpg
https://www.dermis.net/bilder/CD035/550px/img0077.jpg
https://www.dermis.net/bilder/CD036/550px/img0049.jpg
https://www.dermis.net/bilder/CD189/550px/img0072.jpg
https://www.dermis.net/bilder/CD189/550px/img0073.jpg
https://www.dermis.net/bilder/CD184/550px/img0084.jpg
https://www.dermis.net/bilder/CD036/550px/img0094.jpg
https://www.dermis.net/bilder/CD195/550px/img0029.jpg
https://www.dermis.net/bilder/CD021/550px/img0092.jpg
https://www.dermis.net/bilder/CD024/550px/img0082.jpg
https://www.dermis.net/bilder/CD007/550px/img0016.jpg
https://www.dermis.net/bilder/CD189/550px/img0059.jpg
https://www.dermis.net/bilder/CD190/550px/img0045.jpg
https://www.dermis.net/bilder/CD190/550px/img0046.jpg
https://www.dermis.net/bilder/CD194/550px/img0051.jpg
https://www.dermis.net/bilder/CD216/550px/img0001.jpg
https://www.dermis.net/bilder/CD007/550px/img0116.jpg
https://www.dermis.net/bilder/CD035/550px/img0070.jpg
https://www.dermis.net/bilder/CD192/550px/img0012.jpg
https://www.dermis.net/bilder/CD210/550px/img0124.jpg
https://www.dermis.net/bilder/CD211/550px/img0059.jpg
https://www.dermis.net/bilder/CD212/550px/img0001.jpg
https://www.dermis.net/bilder/CD036/550px/img0005.jpg
https://www.dermis.net/bilder/CD056/550px/img0067.jpg
https://www.dermis.net/bilder/CD180/550px/img0094.jpg
https://www.dermis.net/bilder/CD012/550px/img0068.jpg
https://www.dermis.net/bilder/CD199/550px/img0140.jpg
https://www.dermis.net/bilder/CD033/550px/img0072.jpg
https://www.dermis.net/bilder/CD033/550px/img0079.jpg
https://www.dermis.net/bilder/CD033/550px/img0080.jpg
https://www.dermis.net/bilder/CD199/550px/img0021.jpg
https://www.dermis.net/bilder/CD013/550px/img0018.jpg
https://www.dermis.net/bilder/CD018/550px/img0078.jpg

https://www.dermis.net/bilder/CD179/550px/img0001.jpg
https://www.dermis.net/bilder/CD179/550px/img0002.jpg
https://www.dermis.net/bilder/CD194/550px/img0131.jpg
https://www.dermis.net/bilder/CD194/550px/img0132.jpg
https://www.dermis.net/bilder/CD179/550px/img0025.jpg
https://www.dermis.net/bilder/CD016/550px/img0039.jpg
https://www.dermis.net/bilder/CD040/550px/img0093.jpg
https://www.dermis.net/bilder/CD040/550px/img0094.jpg
https://www.dermis.net/bilder/CD212/550px/img0069.jpg
https://www.dermis.net/bilder/CD039/550px/img0095.jpg
https://www.dermis.net/bilder/CD187/550px/img0036.jpg
https://www.dermis.net/bilder/CD012/550px/img0072.jpg
https://www.dermis.net/bilder/CD193/550px/img0094.jpg
https://www.dermis.net/bilder/CD193/550px/img0095.jpg
https://www.dermis.net/bilder/CD198/550px/img0083.jpg
https://www.dermis.net/bilder/CD192/550px/img0032.jpg
https://www.dermis.net/bilder/CD195/550px/img0068.jpg
https://www.dermis.net/bilder/CD198/550px/img0091.jpg
https://www.dermis.net/bilder/CD198/550px/img0092.jpg
https://www.dermis.net/bilder/CD193/550px/img0105.jpg
https://www.dermis.net/bilder/CD194/550px/img0061.jpg
https://www.dermis.net/bilder/CD194/550px/img0062.jpg
https://www.dermis.net/bilder/CD192/550px/img0011.jpg
https://www.dermis.net/bilder/CD192/550px/img0019.jpg
https://www.dermis.net/bilder/CD212/550px/img0075.jpg
https://www.dermis.net/bilder/CD211/550px/img0030.jpg
https://www.dermis.net/bilder/CD033/550px/img0043.jpg
https://www.dermis.net/bilder/CD196/550px/img0087.jpg
https://www.dermis.net/bilder/CD189/550px/img0077.jpg
https://www.dermis.net/bilder/CD025/550px/img0020.jpg
https://www.dermis.net/bilder/CD021/550px/img0056.jpg
https://www.dermis.net/bilder/CD183/550px/img0003.jpg
https://www.dermis.net/bilder/CD183/550px/img0004.jpg
https://www.dermis.net/bilder/CD199/550px/img0028.jpg
https://www.dermis.net/bilder/CD212/550px/img0071.jpg
https://www.dermis.net/bilder/CD199/550px/img0026.jpg
https://www.dermis.net/bilder/CD033/550px/img0013.jpg
https://www.dermis.net/bilder/CD012/550px/img0069.jpg
https://www.dermis.net/bilder/CD055/550px/img0047.jpg
https://www.dermis.net/bilder/CD188/550px/img0022.jpg
https://www.dermis.net/bilder/CD188/550px/img0023.jpg
https://www.dermis.net/bilder/CD195/550px/img0074.jpg
https://www.dermis.net/bilder/CD195/550px/img0075.jpg
https://www.dermis.net/bilder/CD184/550px/img0047.jpg
https://www.dermis.net/bilder/CD195/550px/img0019.jpg
https://www.dermis.net/bilder/CD179/550px/img0003.jpg
https://www.dermis.net/bilder/CD182/550px/img0032.jpg
https://www.dermis.net/bilder/CD199/550px/img0065.jpg

https://www.dermis.net/bilder/CD191/550px/img0105.jpg
https://www.dermis.net/bilder/CD178/550px/img0081.jpg
https://www.dermis.net/bilder/CD178/550px/img0082.jpg
https://www.dermis.net/bilder/CD012/550px/img0064.jpg
https://www.dermis.net/bilder/CD192/550px/img0083.jpg
https://www.dermis.net/bilder/CD056/550px/img0089.jpg
https://www.dermis.net/bilder/CD056/550px/img0090.jpg
https://www.dermis.net/bilder/CD201/550px/img0010.jpg
https://www.dermis.net/bilder/CD192/550px/img0077.jpg
https://www.dermis.net/bilder/CD180/550px/img0021.jpg
https://www.dermis.net/bilder/CD180/550px/img0022.jpg
https://www.dermis.net/bilder/CD186/550px/img0001.jpg
https://www.dermis.net/bilder/CD185/550px/img0100.jpg
https://www.dermis.net/bilder/CD186/550px/img0002.jpg
https://www.dermis.net/bilder/CD196/550px/img0168.jpg
https://www.dermis.net/bilder/CD192/550px/img0059.jpg
https://www.dermis.net/bilder/CD198/550px/img0095.jpg
https://www.dermis.net/bilder/CD184/550px/img0048.jpg
https://www.dermis.net/bilder/CD192/550px/img0084.jpg
https://www.dermis.net/bilder/CD033/550px/img0020.jpg
https://www.dermis.net/bilder/CD195/550px/img0066.jpg
https://www.dermis.net/bilder/CD195/550px/img0067.jpg
https://www.dermis.net/bilder/CD021/550px/img0075.jpg
https://www.dermis.net/bilder/CD012/550px/img0063.jpg
https://www.dermis.net/bilder/CD029/550px/img0014.jpg
https://www.dermis.net/bilder/CD033/550px/img0044.jpg
https://www.dermis.net/bilder/CD210/550px/img0061.jpg
https://www.dermis.net/bilder/CD210/550px/img0062.jpg
https://www.dermis.net/bilder/CD025/550px/img0015.jpg
https://www.dermis.net/bilder/CD194/550px/img0122.jpg
https://www.dermis.net/bilder/CD199/550px/img0094.jpg
https://www.dermis.net/bilder/CD212/550px/img0039.jpg
https://www.dermis.net/bilder/CD195/550px/img0103.jpg
https://www.dermis.net/bilder/CD013/550px/img0008.jpg
https://www.dermis.net/bilder/CD028/550px/img0001.jpg
https://www.dermis.net/bilder/CD001/550px/img0046.jpg
https://www.dermis.net/bilder/CD178/550px/img0080.jpg
https://www.dermis.net/bilder/CD041/550px/img0082.jpg
https://www.dermis.net/bilder/CD040/550px/img0031.jpg
https://www.dermis.net/bilder/CD071/550px/img0004.jpg
https://www.dermis.net/bilder/CD028/550px/img0008.jpg
https://www.dermis.net/bilder/CD069/550px/img0096.jpg
https://www.dermis.net/bilder/CD193/550px/img0191.jpg
https://www.dermis.net/bilder/CD193/550px/img0192.jpg
https://www.dermis.net/bilder/CD006/550px/img0004.jpg
https://www.dermis.net/bilder/CD006/550px/img0009.jpg
https://www.dermis.net/bilder/CD054/550px/img0065.jpg
https://www.dermis.net/bilder/CD073/550px/img0001.jpg

https://www.dermis.net/bilder/CD069/550px/img0001.jpg
https://www.dermis.net/bilder/CD070/550px/img0061.jpg
https://www.dermis.net/bilder/CD070/550px/img0062.jpg
https://www.dermis.net/bilder/CD182/550px/img0039.jpg
https://www.dermis.net/bilder/CD056/550px/img0054.jpg
https://www.dermis.net/bilder/CD001/550px/img0079.jpg
https://www.dermis.net/bilder/CD070/550px/img0008.jpg
https://www.dermis.net/bilder/CD006/550px/img0058.jpg
https://www.dermis.net/bilder/CD005/550px/img0019.jpg
https://www.dermis.net/bilder/CD041/550px/img0053.jpg
https://www.dermis.net/bilder/CD042/550px/img0042.jpg
https://www.dermis.net/bilder/CD040/550px/img0077.jpg
https://www.dermis.net/bilder/CD013/550px/img0027.jpg
https://www.dermis.net/bilder/CD181/550px/img0081.jpg
https://www.dermis.net/bilder/CD067/550px/img0035.jpg
https://www.dermis.net/bilder/CD067/550px/img0036.jpg
https://www.dermis.net/bilder/CD067/550px/img0037.jpg
https://www.dermis.net/bilder/CD039/550px/img0028.jpg
https://www.dermis.net/bilder/CD047/550px/img0098.jpg
https://www.dermis.net/bilder/CD067/550px/img0033.jpg
https://www.dermis.net/bilder/CD067/550px/img0034.jpg
https://www.dermis.net/bilder/CD042/550px/img0080.jpg
https://www.dermis.net/bilder/CD071/550px/img0001.jpg
https://www.dermis.net/bilder/CD018/550px/img0022.jpg
https://www.dermis.net/bilder/CD050/550px/img0089.jpg
https://www.dermis.net/bilder/CD044/550px/img0087.jpg
https://www.dermis.net/bilder/CD051/550px/img0052.jpg
https://www.dermis.net/bilder/CD065/550px/img0026.jpg
https://www.dermis.net/bilder/CD065/550px/img0027.jpg
https://www.dermis.net/bilder/CD039/550px/img0024.jpg
https://www.dermis.net/bilder/CD039/550px/img0025.jpg
https://www.dermis.net/bilder/CD040/550px/img0013.jpg
https://www.dermis.net/bilder/CD059/550px/img0057.jpg
https://www.dermis.net/bilder/CD059/550px/img0058.jpg
https://www.dermis.net/bilder/CD059/550px/img0059.jpg
https://www.dermis.net/bilder/CD059/550px/img0060.jpg
https://www.dermis.net/bilder/CD059/550px/img0061.jpg
https://www.dermis.net/bilder/CD023/550px/img0065.jpg
https://www.dermis.net/bilder/CD023/550px/img0066.jpg
https://www.dermis.net/bilder/CD064/550px/img0056.jpg
https://www.dermis.net/bilder/CD064/550px/img0057.jpg
https://www.dermis.net/bilder/CD051/550px/img0018.jpg
https://www.dermis.net/bilder/CD051/550px/img0019.jpg
https://www.dermis.net/bilder/CD073/550px/img0012.jpg
https://www.dermis.net/bilder/CD073/550px/img0013.jpg
https://www.dermis.net/bilder/CD073/550px/img0014.jpg
https://www.dermis.net/bilder/CD073/550px/img0015.jpg
https://www.dermis.net/bilder/CD051/550px/img0055.jpg

https://www.dermis.net/bilder/CD014/550px/img0046.jpg
https://www.dermis.net/bilder/CD073/550px/img0030.jpg
https://www.dermis.net/bilder/CD073/550px/img0031.jpg
https://www.dermis.net/bilder/CD073/550px/img0032.jpg
https://www.dermis.net/bilder/CD039/550px/img0040.jpg
https://www.dermis.net/bilder/CD051/550px/img0069.jpg
https://www.dermis.net/bilder/CD064/550px/img0101.jpg
https://www.dermis.net/bilder/CD064/550px/img0102.jpg
https://www.dermis.net/bilder/CD067/550px/img0082.jpg
https://www.dermis.net/bilder/CD067/550px/img0083.jpg
https://www.dermis.net/bilder/CD056/550px/img0057.jpg
https://www.dermis.net/bilder/CD005/550px/img0061.jpg
https://www.dermis.net/bilder/CD071/550px/img0068.jpg
https://www.dermis.net/bilder/CD067/550px/img0001.jpg
https://www.dermis.net/bilder/CD067/550px/img0005.jpg
https://www.dermis.net/bilder/CD048/550px/img0035.jpg
https://www.dermis.net/bilder/CD067/550px/img0002.jpg
https://www.dermis.net/bilder/CD067/550px/img0004.jpg
https://www.dermis.net/bilder/CD064/550px/img0073.jpg
https://www.dermis.net/bilder/CD064/550px/img0074.jpg
https://www.dermis.net/bilder/CD064/550px/img0075.jpg
https://www.dermis.net/bilder/CD064/550px/img0076.jpg
https://www.dermis.net/bilder/CD043/550px/img0003.jpg
https://www.dermis.net/bilder/CD018/550px/img0014.jpg
https://www.dermis.net/bilder/CD064/550px/img0049.jpg
https://www.dermis.net/bilder/CD064/550px/img0050.jpg
https://www.dermis.net/bilder/CD064/550px/img0051.jpg
https://www.dermis.net/bilder/CD064/550px/img0052.jpg
https://www.dermis.net/bilder/CD064/550px/img0053.jpg
https://www.dermis.net/bilder/CD064/550px/img0054.jpg
https://www.dermis.net/bilder/CD064/550px/img0055.jpg
https://www.dermis.net/bilder/CD026/550px/img0099.jpg
https://www.dermis.net/bilder/CD066/550px/img0034.jpg
https://www.dermis.net/bilder/CD066/550px/img0035.jpg
https://www.dermis.net/bilder/CD066/550px/img0036.jpg
https://www.dermis.net/bilder/CD066/550px/img0037.jpg
https://www.dermis.net/bilder/CD005/550px/img0033.jpg
https://www.dermis.net/bilder/CD050/550px/img0010.jpg
https://www.dermis.net/bilder/CD050/550px/img0016.jpg
https://www.dermis.net/bilder/CD050/550px/img0011.jpg
https://www.dermis.net/bilder/CD050/550px/img0013.jpg
https://www.dermis.net/bilder/CD050/550px/img0014.jpg
https://www.dermis.net/bilder/CD050/550px/img0018.jpg
https://www.dermis.net/bilder/CD067/550px/img0026.jpg
https://www.dermis.net/bilder/CD067/550px/img0027.jpg
https://www.dermis.net/bilder/CD067/550px/img0028.jpg
https://www.dermis.net/bilder/CD004/550px/img0007.jpg
https://www.dermis.net/bilder/CD067/550px/img0006.jpg

https://www.dermis.net/bilder/CD016/550px/img0046.jpg
https://www.dermis.net/bilder/CD067/550px/img0007.jpg
https://www.dermis.net/bilder/CD067/550px/img0009.jpg
https://www.dermis.net/bilder/CD067/550px/img0010.jpg
https://www.dermis.net/bilder/CD067/550px/img0011.jpg
https://www.dermis.net/bilder/CD067/550px/img0012.jpg
https://www.dermis.net/bilder/CD047/550px/img0048.jpg
https://www.dermis.net/bilder/CD050/550px/img0062.jpg
https://www.dermis.net/bilder/CD048/550px/img0052.jpg
https://www.dermis.net/bilder/CD006/550px/img0070.jpg
https://www.dermis.net/bilder/CD071/550px/img0010.jpg
https://www.dermis.net/bilder/CD073/550px/img0016.jpg
https://www.dermis.net/bilder/CD073/550px/img0017.jpg
https://www.dermis.net/bilder/CD073/550px/img0018.jpg
https://www.dermis.net/bilder/CD003/550px/img0004.jpg
https://www.dermis.net/bilder/CD018/550px/img0062.jpg
https://www.dermis.net/bilder/CD063/550px/img0085.jpg
https://www.dermis.net/bilder/CD014/550px/img0066.jpg
https://www.dermis.net/bilder/CD006/550px/img0069.jpg
https://www.dermis.net/bilder/CD012/550px/img0091.jpg
https://www.dermis.net/bilder/CD065/550px/img0094.jpg
https://www.dermis.net/bilder/CD065/550px/img0096.jpg
https://www.dermis.net/bilder/CD018/550px/img0068.jpg
https://www.dermis.net/bilder/CD056/550px/img0016.jpg
https://www.dermis.net/bilder/CD063/550px/img0001.jpg
https://www.dermis.net/bilder/CD063/550px/img0002.jpg
https://www.dermis.net/bilder/CD063/550px/img0005.jpg
https://www.dermis.net/bilder/CD059/550px/img0045.jpg
https://www.dermis.net/bilder/CD059/550px/img0046.jpg
https://www.dermis.net/bilder/CD059/550px/img0047.jpg
https://www.dermis.net/bilder/CD014/550px/img0045.jpg
https://www.dermis.net/bilder/CD063/550px/img0003.jpg
https://www.dermis.net/bilder/CD046/550px/img0017.jpg
https://www.dermis.net/bilder/CD046/550px/img0018.jpg
https://www.dermis.net/bilder/CD046/550px/img0024.jpg
https://www.dermis.net/bilder/CD065/550px/img0024.jpg
https://www.dermis.net/bilder/CD056/550px/img0045.jpg
https://www.dermis.net/bilder/CD066/550px/img0097.jpg
https://www.dermis.net/bilder/CD066/550px/img0098.jpg
https://www.dermis.net/bilder/CD063/550px/img0091.jpg
https://www.dermis.net/bilder/CD063/550px/img0092.jpg
https://www.dermis.net/bilder/CD063/550px/img0080.jpg
https://www.dermis.net/bilder/CD069/550px/img0085.jpg
https://www.dermis.net/bilder/CD014/550px/img0042.jpg
https://www.dermis.net/bilder/CD042/550px/img0027.jpg
https://www.dermis.net/bilder/CD052/550px/img0057.jpg
https://www.dermis.net/bilder/CD058/550px/img0026.jpg
https://www.dermis.net/bilder/CD048/550px/img0063.jpg

https://www.dermis.net/bilder/CD048/550px/img0064.jpg
https://www.dermis.net/bilder/CD058/550px/img0025.jpg
https://www.dermis.net/bilder/CD054/550px/img0101.jpg
https://www.dermis.net/bilder/CD054/550px/img0102.jpg
https://www.dermis.net/bilder/CD049/550px/img0093.jpg
https://www.dermis.net/bilder/CD003/550px/img0082.jpg
https://www.dermis.net/bilder/CD003/550px/img0047.jpg
https://www.dermis.net/bilder/CD048/550px/img0084.jpg
https://www.dermis.net/bilder/CD026/550px/img0057.jpg
https://www.dermis.net/bilder/CD008/550px/img0062.jpg
https://www.dermis.net/bilder/CD058/550px/img0027.jpg
https://www.dermis.net/bilder/CD042/550px/img0039.jpg
https://www.dermis.net/bilder/CD052/550px/img0048.jpg
https://www.dermis.net/bilder/CD023/550px/img0053.jpg
https://www.dermis.net/bilder/CD023/550px/img0054.jpg
https://www.dermis.net/bilder/CD056/550px/img0048.jpg
https://www.dermis.net/bilder/CD048/550px/img0069.jpg
https://www.dermis.net/bilder/CD006/550px/img0033.jpg
https://www.dermis.net/bilder/CD006/550px/img0022.jpg
https://www.dermis.net/bilder/CD044/550px/img0038.jpg
https://www.dermis.net/bilder/CD036/550px/img0081.jpg
https://www.dermis.net/bilder/CD050/550px/img0069.jpg
https://www.dermis.net/bilder/CD036/550px/img0068.jpg
https://www.dermis.net/bilder/CD059/550px/img0042.jpg
https://www.dermis.net/bilder/CD059/550px/img0043.jpg
https://www.dermis.net/bilder/CD059/550px/img0044.jpg
https://www.dermis.net/bilder/CD005/550px/img0098.jpg
https://www.dermis.net/bilder/CD014/550px/img0035.jpg
https://www.dermis.net/bilder/CD003/550px/img0044.jpg
https://www.dermis.net/bilder/CD059/550px/img0029.jpg
https://www.dermis.net/bilder/CD059/550px/img0030.jpg
https://www.dermis.net/bilder/CD023/550px/img0031.jpg
https://www.dermis.net/bilder/CD058/550px/img0028.jpg
https://www.dermis.net/bilder/CD004/550px/img0002.jpg
https://www.dermis.net/bilder/CD018/550px/img0073.jpg
https://www.dermis.net/bilder/CD014/550px/img0090.jpg
https://www.dermis.net/bilder/CD039/550px/img0053.jpg
https://www.dermis.net/bilder/CD026/550px/img0065.jpg
https://www.dermis.net/bilder/CD026/550px/img0066.jpg
https://www.dermis.net/bilder/CD044/550px/img0045.jpg
https://www.dermis.net/bilder/CD066/550px/img0101.jpg
https://www.dermis.net/bilder/CD066/550px/img0102.jpg
https://www.dermis.net/bilder/CD063/550px/img0036.jpg
https://www.dermis.net/bilder/CD063/550px/img0034.jpg
https://www.dermis.net/bilder/CD063/550px/img0035.jpg
https://www.dermis.net/bilder/CD025/550px/img0098.jpg
https://www.dermis.net/bilder/CD025/550px/img0099.jpg
https://www.dermis.net/bilder/CD067/550px/img0023.jpg

https://www.dermis.net/bilder/CD054/550px/img0096.jpg
https://www.dermis.net/bilder/CD054/550px/img0098.jpg
https://www.dermis.net/bilder/CD014/550px/img0007.jpg
https://www.dermis.net/bilder/CD067/550px/img0065.jpg
https://www.dermis.net/bilder/CD063/550px/img0009.jpg
https://www.dermis.net/bilder/CD063/550px/img0011.jpg
https://www.dermis.net/bilder/CD063/550px/img0012.jpg
https://www.dermis.net/bilder/CD063/550px/img0010.jpg
https://www.dermis.net/bilder/CD063/550px/img0054.jpg
https://www.dermis.net/bilder/CD036/550px/img0092.jpg
https://www.dermis.net/bilder/CD007/550px/img0003.jpg
https://www.dermis.net/bilder/CD052/550px/img0095.jpg
https://www.dermis.net/bilder/CD001/550px/img0102.jpg
https://www.dermis.net/bilder/CD028/550px/img0026.jpg
https://www.dermis.net/bilder/CD028/550px/img0027.jpg
https://www.dermis.net/bilder/CD028/550px/img0029.jpg
https://www.dermis.net/bilder/CD028/550px/img0030.jpg
https://www.dermis.net/bilder/CD028/550px/img0032.jpg
https://www.dermis.net/bilder/CD028/550px/img0036.jpg
https://www.dermis.net/bilder/CD027/550px/img0041.jpg
https://www.dermis.net/bilder/CD027/550px/img0045.jpg
https://www.dermis.net/bilder/CD054/550px/img0095.jpg
https://www.dermis.net/bilder/CD003/550px/img0017.jpg
https://www.dermis.net/bilder/CD064/550px/img0094.jpg
https://www.dermis.net/bilder/CD064/550px/img0095.jpg
https://www.dermis.net/bilder/CD064/550px/img0096.jpg
https://www.dermis.net/bilder/CD059/550px/img0037.jpg
https://www.dermis.net/bilder/CD067/550px/img0020.jpg
https://www.dermis.net/bilder/CD016/550px/img0011.jpg
https://www.dermis.net/bilder/CD067/550px/img0018.jpg
https://www.dermis.net/bilder/CD067/550px/img0019.jpg
https://www.dermis.net/bilder/CD067/550px/img0021.jpg
https://www.dermis.net/bilder/CD067/550px/img0022.jpg
https://www.dermis.net/bilder/CD023/550px/img0002.jpg
https://www.dermis.net/bilder/CD023/550px/img0004.jpg
https://www.dermis.net/bilder/CD023/550px/img0058.jpg
https://www.dermis.net/bilder/CD058/550px/img0006.jpg
https://www.dermis.net/bilder/CD058/550px/img0001.jpg
https://www.dermis.net/bilder/CD058/550px/img0002.jpg
https://www.dermis.net/bilder/CD058/550px/img0035.jpg
https://www.dermis.net/bilder/CD058/550px/img0099.jpg
https://www.dermis.net/bilder/CD049/550px/img0007.jpg
https://www.dermis.net/bilder/CD049/550px/img0008.jpg
https://www.dermis.net/bilder/CD049/550px/img0009.jpg
https://www.dermis.net/bilder/CD049/550px/img0010.jpg
https://www.dermis.net/bilder/CD049/550px/img0013.jpg
https://www.dermis.net/bilder/CD049/550px/img0014.jpg
https://www.dermis.net/bilder/CD049/550px/img0015.jpg

https://www.dermis.net/bilder/CD049/550px/img0017.jpg
https://www.dermis.net/bilder/CD049/550px/img0018.jpg
https://www.dermis.net/bilder/CD049/550px/img0019.jpg
https://www.dermis.net/bilder/CD060/550px/img0021.jpg
https://www.dermis.net/bilder/CD060/550px/img0020.jpg
https://www.dermis.net/bilder/CD020/550px/img0068.jpg
https://www.dermis.net/bilder/CD018/550px/img0083.jpg
https://www.dermis.net/bilder/CD057/550px/img0048.jpg
https://www.dermis.net/bilder/CD055/550px/img0086.jpg
https://www.dermis.net/bilder/CD060/550px/img0093.jpg
https://www.dermis.net/bilder/CD060/550px/img0094.jpg
https://www.dermis.net/bilder/CD060/550px/img0095.jpg
https://www.dermis.net/bilder/CD042/550px/img0074.jpg
https://www.dermis.net/bilder/CD042/550px/img0075.jpg
https://www.dermis.net/bilder/CD036/550px/img0061.jpg
https://www.dermis.net/bilder/CD049/550px/img0029.jpg
https://www.dermis.net/bilder/CD032/550px/img0063.jpg
https://www.dermis.net/bilder/CD032/550px/img0064.jpg
https://www.dermis.net/bilder/CD003/550px/img0089.jpg
https://www.dermis.net/bilder/CD067/550px/img0072.jpg
https://www.dermis.net/bilder/CD065/550px/img0088.jpg
https://www.dermis.net/bilder/CD065/550px/img0090.jpg
https://www.dermis.net/bilder/CD065/550px/img0091.jpg
https://www.dermis.net/bilder/CD065/550px/img0093.jpg
https://www.dermis.net/bilder/CD051/550px/img0086.jpg
https://www.dermis.net/bilder/CD060/550px/img0097.jpg
https://www.dermis.net/bilder/CD060/550px/img0099.jpg
https://www.dermis.net/bilder/CD060/550px/img0100.jpg
https://www.dermis.net/bilder/CD060/550px/img0101.jpg
https://www.dermis.net/bilder/CD060/550px/img0102.jpg
https://www.dermis.net/bilder/CD059/550px/img0025.jpg
https://www.dermis.net/bilder/CD059/550px/img0026.jpg
https://www.dermis.net/bilder/CD058/550px/img0009.jpg
https://www.dermis.net/bilder/CD058/550px/img0016.jpg
https://www.dermis.net/bilder/CD058/550px/img0014.jpg
https://www.dermis.net/bilder/CD058/550px/img0019.jpg
https://www.dermis.net/bilder/CD067/550px/img0042.jpg
https://www.dermis.net/bilder/CD067/550px/img0043.jpg
https://www.dermis.net/bilder/CD067/550px/img0044.jpg
https://www.dermis.net/bilder/CD067/550px/img0045.jpg
https://www.dermis.net/bilder/CD020/550px/img0092.jpg
https://www.dermis.net/bilder/CD049/550px/img0001.jpg
https://www.dermis.net/bilder/CD049/550px/img0002.jpg
https://www.dermis.net/bilder/CD049/550px/img0003.jpg
https://www.dermis.net/bilder/CD049/550px/img0004.jpg
https://www.dermis.net/bilder/CD049/550px/img0005.jpg
https://www.dermis.net/bilder/CD049/550px/img0006.jpg
https://www.dermis.net/bilder/CD007/550px/img0015.jpg

https://www.dermis.net/bilder/CD049/550px/img0062.jpg
https://www.dermis.net/bilder/CD049/550px/img0063.jpg
https://www.dermis.net/bilder/CD065/550px/img0100.jpg
https://www.dermis.net/bilder/CD060/550px/img0074.jpg
https://www.dermis.net/bilder/CD060/550px/img0075.jpg
https://www.dermis.net/bilder/CD020/550px/img0011.jpg
https://www.dermis.net/bilder/CD048/550px/img0061.jpg
https://www.dermis.net/bilder/CD048/550px/img0062.jpg
https://www.dermis.net/bilder/CD067/550px/img0093.jpg
https://www.dermis.net/bilder/CD067/550px/img0094.jpg
https://www.dermis.net/bilder/CD018/550px/img0096.jpg
https://www.dermis.net/bilder/CD052/550px/img0065.jpg
https://www.dermis.net/bilder/CD055/550px/img0087.jpg
https://www.dermis.net/bilder/CD046/550px/img0053.jpg
https://www.dermis.net/bilder/CD029/550px/img0019.jpg
https://www.dermis.net/bilder/CD049/550px/img0050.jpg
https://www.dermis.net/bilder/CD065/550px/img0056.jpg
https://www.dermis.net/bilder/CD063/550px/img0029.jpg
https://www.dermis.net/bilder/CD049/550px/img0083.jpg
https://www.dermis.net/bilder/CD024/550px/img0019.jpg
https://www.dermis.net/bilder/CD035/550px/img0023.jpg
https://www.dermis.net/bilder/CD035/550px/img0024.jpg
https://www.dermis.net/bilder/CD014/550px/img0003.jpg
https://www.dermis.net/bilder/CD024/550px/img0094.jpg
https://www.dermis.net/bilder/CD049/550px/img0079.jpg
https://www.dermis.net/bilder/CD065/550px/img0010.jpg
https://www.dermis.net/bilder/CD058/550px/img0077.jpg
https://www.dermis.net/bilder/CD058/550px/img0084.jpg
https://www.dermis.net/bilder/CD154/550px/img0088.jpg
https://www.dermis.net/bilder/CD154/550px/img0089.jpg
https://www.dermis.net/bilder/CD063/550px/img0065.jpg
https://www.dermis.net/bilder/CD026/550px/img0033.jpg
https://www.dermis.net/bilder/CD026/550px/img0036.jpg
https://www.dermis.net/bilder/CD026/550px/img0037.jpg
https://www.dermis.net/bilder/CD042/550px/img0043.jpg
https://www.dermis.net/bilder/CD036/550px/img0020.jpg
https://www.dermis.net/bilder/CD065/550px/img0074.jpg
https://www.dermis.net/bilder/CD065/550px/img0075.jpg
https://www.dermis.net/bilder/CD036/550px/img0026.jpg
https://www.dermis.net/bilder/CD060/550px/img0002.jpg
https://www.dermis.net/bilder/CD066/550px/img0064.jpg
https://www.dermis.net/bilder/CD066/550px/img0065.jpg
https://www.dermis.net/bilder/CD066/550px/img0066.jpg
https://www.dermis.net/bilder/CD066/550px/img0067.jpg
https://www.dermis.net/bilder/CD058/550px/img0065.jpg
https://www.dermis.net/bilder/CD058/550px/img0069.jpg
https://www.dermis.net/bilder/CD058/550px/img0070.jpg
https://www.dermis.net/bilder/CD058/550px/img0071.jpg

https://www.dermis.net/bilder/CD058/550px/img0072.jpg
https://www.dermis.net/bilder/CD060/550px/img0001.jpg
https://www.dermis.net/bilder/CD047/550px/img0025.jpg
https://www.dermis.net/bilder/CD044/550px/img0022.jpg
https://www.dermis.net/bilder/CD015/550px/img0041.jpg
https://www.dermis.net/bilder/CD059/550px/img0071.jpg
https://www.dermis.net/bilder/CD059/550px/img0072.jpg
https://www.dermis.net/bilder/CD037/550px/img0001.jpg
https://www.dermis.net/bilder/CD063/550px/img0084.jpg
https://www.dermis.net/bilder/CD056/550px/img0008.jpg
https://www.dermis.net/bilder/CD057/550px/img0024.jpg
https://www.dermis.net/bilder/CD040/550px/img0008.jpg
https://www.dermis.net/bilder/CD027/550px/img0077.jpg
https://www.dermis.net/bilder/CD057/550px/img0064.jpg
https://www.dermis.net/bilder/CD024/550px/img0098.jpg
https://www.dermis.net/bilder/CD014/550px/img0023.jpg
https://www.dermis.net/bilder/CD067/550px/img0024.jpg
https://www.dermis.net/bilder/CD026/550px/img0076.jpg
https://www.dermis.net/bilder/CD058/550px/img0100.jpg
https://www.dermis.net/bilder/CD066/550px/img0090.jpg
https://www.dermis.net/bilder/CD066/550px/img0091.jpg
https://www.dermis.net/bilder/CD058/550px/img0041.jpg
https://www.dermis.net/bilder/CD058/550px/img0047.jpg
https://www.dermis.net/bilder/CD058/550px/img0048.jpg
https://www.dermis.net/bilder/CD014/550px/img0033.jpg
https://www.dermis.net/bilder/CD058/550px/img0045.jpg
https://www.dermis.net/bilder/CD004/550px/img0080.jpg
https://www.dermis.net/bilder/CD018/550px/img0091.jpg
https://www.dermis.net/bilder/CD039/550px/img0081.jpg
https://www.dermis.net/bilder/CD039/550px/img0083.jpg
https://www.dermis.net/bilder/CD042/550px/img0055.jpg
https://www.dermis.net/bilder/CD042/550px/img0056.jpg
https://www.dermis.net/bilder/CD042/550px/img0057.jpg
https://www.dermis.net/bilder/CD058/550px/img0021.jpg
https://www.dermis.net/bilder/CD058/550px/img0046.jpg
https://www.dermis.net/bilder/CD064/550px/img0066.jpg
https://www.dermis.net/bilder/CD064/550px/img0068.jpg
https://www.dermis.net/bilder/CD061/550px/img0009.jpg
https://www.dermis.net/bilder/CD061/550px/img0010.jpg
https://www.dermis.net/bilder/CD063/550px/img0030.jpg
https://www.dermis.net/bilder/CD063/550px/img0031.jpg
https://www.dermis.net/bilder/CD018/550px/img0018.jpg
https://www.dermis.net/bilder/CD057/550px/img0035.jpg
https://www.dermis.net/bilder/CD067/550px/img0100.jpg
https://www.dermis.net/bilder/CD033/550px/img0090.jpg
https://www.dermis.net/bilder/CD058/550px/img0064.jpg
https://www.dermis.net/bilder/CD047/550px/img0053.jpg
https://www.dermis.net/bilder/CD047/550px/img0056.jpg

https://www.dermis.net/bilder/CD063/550px/img0078.jpg
https://www.dermis.net/bilder/CD063/550px/img0070.jpg
https://www.dermis.net/bilder/CD063/550px/img0071.jpg
https://www.dermis.net/bilder/CD032/550px/img0061.jpg
https://www.dermis.net/bilder/CD052/550px/img0036.jpg
https://www.dermis.net/bilder/CD035/550px/img0092.jpg
https://www.dermis.net/bilder/CD064/550px/img0012.jpg
https://www.dermis.net/bilder/CD064/550px/img0013.jpg
https://www.dermis.net/bilder/CD064/550px/img0014.jpg
https://www.dermis.net/bilder/CD040/550px/img0051.jpg
https://www.dermis.net/bilder/CD064/550px/img0015.jpg
https://www.dermis.net/bilder/CD064/550px/img0016.jpg
https://www.dermis.net/bilder/CD064/550px/img0018.jpg
https://www.dermis.net/bilder/CD064/550px/img0019.jpg
https://www.dermis.net/bilder/CD064/550px/img0020.jpg
https://www.dermis.net/bilder/CD064/550px/img0021.jpg
https://www.dermis.net/bilder/CD067/550px/img0084.jpg
https://www.dermis.net/bilder/CD067/550px/img0085.jpg
https://www.dermis.net/bilder/CD065/550px/img0017.jpg
https://www.dermis.net/bilder/CD023/550px/img0039.jpg
https://www.dermis.net/bilder/CD047/550px/img0046.jpg
https://www.dermis.net/bilder/CD025/550px/img0029.jpg
https://www.dermis.net/bilder/CD065/550px/img0106.jpg
https://www.dermis.net/bilder/CD064/550px/img0089.jpg
https://www.dermis.net/bilder/CD064/550px/img0090.jpg
https://www.dermis.net/bilder/CD064/550px/img0091.jpg
https://www.dermis.net/bilder/CD064/550px/img0092.jpg
https://www.dermis.net/bilder/CD007/550px/img0041.jpg
https://www.dermis.net/bilder/CD037/550px/img0053.jpg
https://www.dermis.net/bilder/CD027/550px/img0067.jpg
https://www.dermis.net/bilder/CD027/550px/img0068.jpg
https://www.dermis.net/bilder/CD054/550px/img0055.jpg
https://www.dermis.net/bilder/CD064/550px/img0083.jpg
https://www.dermis.net/bilder/CD064/550px/img0084.jpg
https://www.dermis.net/bilder/CD064/550px/img0085.jpg
https://www.dermis.net/bilder/CD064/550px/img0086.jpg
https://www.dermis.net/bilder/CD064/550px/img0087.jpg
https://www.dermis.net/bilder/CD064/550px/img0088.jpg
https://www.dermis.net/bilder/CD059/550px/img0020.jpg
https://www.dermis.net/bilder/CD059/550px/img0021.jpg
https://www.dermis.net/bilder/CD059/550px/img0022.jpg
https://www.dermis.net/bilder/CD059/550px/img0023.jpg
https://www.dermis.net/bilder/CD063/550px/img0038.jpg
https://www.dermis.net/bilder/CD063/550px/img0039.jpg
https://www.dermis.net/bilder/CD063/550px/img0040.jpg
https://www.dermis.net/bilder/CD046/550px/img0085.jpg
https://www.dermis.net/bilder/CD046/550px/img0086.jpg
https://www.dermis.net/bilder/CD065/550px/img0030.jpg

https://www.dermis.net/bilder/CD065/550px/img0032.jpg
https://www.dermis.net/bilder/CD065/550px/img0033.jpg
https://www.dermis.net/bilder/CD050/550px/img0023.jpg
https://www.dermis.net/bilder/CD050/550px/img0024.jpg
https://www.dermis.net/bilder/CD047/550px/img0010.jpg
https://www.dermis.net/bilder/CD033/550px/img0075.jpg
https://www.dermis.net/bilder/CD066/550px/img0073.jpg
https://www.dermis.net/bilder/CD066/550px/img0074.jpg
https://www.dermis.net/bilder/CD066/550px/img0075.jpg
https://www.dermis.net/bilder/CD018/550px/img0009.jpg
https://www.dermis.net/bilder/CD067/550px/img0103.jpg
https://www.dermis.net/bilder/CD027/550px/img0012.jpg
https://www.dermis.net/bilder/CD027/550px/img0011.jpg
https://www.dermis.net/bilder/CD050/550px/img0046.jpg
https://www.dermis.net/bilder/CD050/550px/img0047.jpg
https://www.dermis.net/bilder/CD039/550px/img0002.jpg
https://www.dermis.net/bilder/CD054/550px/img0073.jpg
https://www.dermis.net/bilder/CD058/550px/img0052.jpg
https://www.dermis.net/bilder/CD060/550px/img0069.jpg
https://www.dermis.net/bilder/CD064/550px/img0105.jpg
https://www.dermis.net/bilder/CD064/550px/img0106.jpg
https://www.dermis.net/bilder/CD005/550px/img0015.jpg
https://www.dermis.net/bilder/CD004/550px/img0076.jpg
https://www.dermis.net/bilder/CD063/550px/img0049.jpg
https://www.dermis.net/bilder/CD063/550px/img0050.jpg
https://www.dermis.net/bilder/CD063/550px/img0051.jpg
https://www.dermis.net/bilder/CD067/550px/img0108.jpg
https://www.dermis.net/bilder/CD051/550px/img0020.jpg
https://www.dermis.net/bilder/CD039/550px/img0057.jpg
https://www.dermis.net/bilder/CD064/550px/img0079.jpg
https://www.dermis.net/bilder/CD064/550px/img0082.jpg
https://www.dermis.net/bilder/CD066/550px/img0104.jpg
https://www.dermis.net/bilder/CD057/550px/img0042.jpg
https://www.dermis.net/bilder/CD052/550px/img0035.jpg
https://www.dermis.net/bilder/CD065/550px/img0084.jpg
https://www.dermis.net/bilder/CD065/550px/img0085.jpg
https://www.dermis.net/bilder/CD065/550px/img0086.jpg
https://www.dermis.net/bilder/CD066/550px/img0024.jpg
https://www.dermis.net/bilder/CD066/550px/img0023.jpg
https://www.dermis.net/bilder/CD044/550px/img0031.jpg
https://www.dermis.net/bilder/CD057/550px/img0043.jpg
https://www.dermis.net/bilder/CD057/550px/img0044.jpg
https://www.dermis.net/bilder/CD048/550px/img0057.jpg
https://www.dermis.net/bilder/CD059/550px/img0031.jpg
https://www.dermis.net/bilder/CD059/550px/img0032.jpg
https://www.dermis.net/bilder/CD063/550px/img0024.jpg
https://www.dermis.net/bilder/CD065/550px/img0098.jpg
https://www.dermis.net/bilder/CD025/550px/img0083.jpg

https://www.dermis.net/bilder/CD025/550px/img0084.jpg
https://www.dermis.net/bilder/CD047/550px/img0034.jpg
https://www.dermis.net/bilder/CD047/550px/img0035.jpg
https://www.dermis.net/bilder/CD047/550px/img0036.jpg
https://www.dermis.net/bilder/CD047/550px/img0037.jpg
https://www.dermis.net/bilder/CD065/550px/img0061.jpg
https://www.dermis.net/bilder/CD007/550px/img0032.jpg
https://www.dermis.net/bilder/CD033/550px/img0088.jpg
https://www.dermis.net/bilder/CD063/550px/img0055.jpg
https://www.dermis.net/bilder/CD063/550px/img0056.jpg
https://www.dermis.net/bilder/CD049/550px/img0087.jpg
https://www.dermis.net/bilder/CD049/550px/img0094.jpg
https://www.dermis.net/bilder/CD049/550px/img0095.jpg
https://www.dermis.net/bilder/CD063/550px/img0044.jpg
https://www.dermis.net/bilder/CD063/550px/img0045.jpg
https://www.dermis.net/bilder/CD063/550px/img0046.jpg
https://www.dermis.net/bilder/CD007/550px/img0001.jpg
https://www.dermis.net/bilder/CD025/550px/img0035.jpg
https://www.dermis.net/bilder/CD034/550px/img0009.jpg
https://www.dermis.net/bilder/CD024/550px/img0062.jpg
https://www.dermis.net/bilder/CD024/550px/img0063.jpg
https://www.dermis.net/bilder/CD024/550px/img0064.jpg
https://www.dermis.net/bilder/CD024/550px/img0067.jpg
https://www.dermis.net/bilder/CD024/550px/img0072.jpg
https://www.dermis.net/bilder/CD060/550px/img0048.jpg
https://www.dermis.net/bilder/CD043/550px/img0048.jpg
https://www.dermis.net/bilder/CD048/550px/img0079.jpg
https://www.dermis.net/bilder/CD027/550px/img0071.jpg
https://www.dermis.net/bilder/CD067/550px/img0090.jpg
https://www.dermis.net/bilder/CD051/550px/img0063.jpg
https://www.dermis.net/bilder/CD056/550px/img0034.jpg
https://www.dermis.net/bilder/CD004/550px/img0096.jpg
https://www.dermis.net/bilder/CD064/550px/img0097.jpg
https://www.dermis.net/bilder/CD064/550px/img0098.jpg
https://www.dermis.net/bilder/CD065/550px/img0052.jpg
https://www.dermis.net/bilder/CD067/550px/img0059.jpg
https://www.dermis.net/bilder/CD024/550px/img0049.jpg
https://www.dermis.net/bilder/CD064/550px/img0004.jpg
https://www.dermis.net/bilder/CD064/550px/img0005.jpg
https://www.dermis.net/bilder/CD064/550px/img0006.jpg
https://www.dermis.net/bilder/CD064/550px/img0007.jpg
https://www.dermis.net/bilder/CD064/550px/img0008.jpg
https://www.dermis.net/bilder/CD064/550px/img0009.jpg
https://www.dermis.net/bilder/CD064/550px/img0010.jpg
https://www.dermis.net/bilder/CD024/550px/img0025.jpg
https://www.dermis.net/bilder/CD024/550px/img0026.jpg
https://www.dermis.net/bilder/CD024/550px/img0027.jpg
https://www.dermis.net/bilder/CD040/550px/img0009.jpg

https://www.dermis.net/bilder/CD036/550px/img0096.jpg
https://www.dermis.net/bilder/CD047/550px/img0079.jpg
https://www.dermis.net/bilder/CD047/550px/img0080.jpg
https://www.dermis.net/bilder/CD002/550px/img0074.jpg
https://www.dermis.net/bilder/CD047/550px/img0082.jpg
https://www.dermis.net/bilder/CD047/550px/img0083.jpg
https://www.dermis.net/bilder/CD026/550px/img0086.jpg
https://www.dermis.net/bilder/CD067/550px/img0057.jpg
https://www.dermis.net/bilder/CD067/550px/img0058.jpg
https://www.dermis.net/bilder/CD024/550px/img0050.jpg
https://www.dermis.net/bilder/CD007/550px/img0033.jpg
https://www.dermis.net/bilder/CD065/550px/img0104.jpg
https://www.dermis.net/bilder/CD014/550px/img0010.jpg
https://www.dermis.net/bilder/CD059/550px/img0001.jpg
https://www.dermis.net/bilder/CD059/550px/img0002.jpg
https://www.dermis.net/bilder/CD059/550px/img0007.jpg
https://www.dermis.net/bilder/CD014/550px/img0011.jpg
https://www.dermis.net/bilder/CD060/550px/img0104.jpg
https://www.dermis.net/bilder/CD060/550px/img0105.jpg
https://www.dermis.net/bilder/CD064/550px/img0060.jpg
https://www.dermis.net/bilder/CD064/550px/img0061.jpg
https://www.dermis.net/bilder/CD064/550px/img0062.jpg
https://www.dermis.net/bilder/CD064/550px/img0063.jpg
https://www.dermis.net/bilder/CD064/550px/img0064.jpg
https://www.dermis.net/bilder/CD049/550px/img0052.jpg
https://www.dermis.net/bilder/CD049/550px/img0054.jpg
https://www.dermis.net/bilder/CD067/550px/img0060.jpg
https://www.dermis.net/bilder/CD067/550px/img0061.jpg
https://www.dermis.net/bilder/CD019/550px/img0011.jpg
https://www.dermis.net/bilder/CD059/550px/img0003.jpg
https://www.dermis.net/bilder/CD059/550px/img0005.jpg
https://www.dermis.net/bilder/CD059/550px/img0006.jpg
https://www.dermis.net/bilder/CD059/550px/img0052.jpg
https://www.dermis.net/bilder/CD059/550px/img0053.jpg
https://www.dermis.net/bilder/CD057/550px/img0003.jpg
https://www.dermis.net/bilder/CD040/550px/img0081.jpg
https://www.dermis.net/bilder/CD048/550px/img0008.jpg
https://www.dermis.net/bilder/CD048/550px/img0011.jpg
https://www.dermis.net/bilder/CD036/550px/img0045.jpg
https://www.dermis.net/bilder/CD018/550px/img0101.jpg
https://www.dermis.net/bilder/CD035/550px/img0033.jpg
https://www.dermis.net/bilder/CD035/550px/img0034.jpg
https://www.dermis.net/bilder/CD037/550px/img0011.jpg
https://www.dermis.net/bilder/CD020/550px/img0059.jpg
https://www.dermis.net/bilder/CD020/550px/img0060.jpg
https://www.dermis.net/bilder/CD018/550px/img0092.jpg
https://www.dermis.net/bilder/CD005/550px/img0046.jpg
https://www.dermis.net/bilder/CD063/550px/img0105.jpg

https://www.dermis.net/bilder/CD066/550px/img0086.jpg
https://www.dermis.net/bilder/CD066/550px/img0087.jpg
https://www.dermis.net/bilder/CD052/550px/img0088.jpg
https://www.dermis.net/bilder/CD007/550px/img0022.jpg
https://www.dermis.net/bilder/CD006/550px/img0090.jpg
https://www.dermis.net/bilder/CD046/550px/img0073.jpg
https://www.dermis.net/bilder/CD046/550px/img0074.jpg
https://www.dermis.net/bilder/CD006/550px/img0024.jpg
https://www.dermis.net/bilder/CD007/550px/img0054.jpg
https://www.dermis.net/bilder/CD061/550px/img0013.jpg
https://www.dermis.net/bilder/CD065/550px/img0034.jpg
https://www.dermis.net/bilder/CD004/550px/img0098.jpg
https://www.dermis.net/bilder/CD033/550px/img0095.jpg
https://www.dermis.net/bilder/CD066/550px/img0105.jpg
https://www.dermis.net/bilder/CD066/550px/img0106.jpg
https://www.dermis.net/bilder/CD067/550px/img0095.jpg
https://www.dermis.net/bilder/CD067/550px/img0096.jpg
https://www.dermis.net/bilder/CD066/550px/img0001.jpg
https://www.dermis.net/bilder/CD066/550px/img0003.jpg
https://www.dermis.net/bilder/CD066/550px/img0004.jpg
https://www.dermis.net/bilder/CD066/550px/img0005.jpg
https://www.dermis.net/bilder/CD066/550px/img0006.jpg
https://www.dermis.net/bilder/CD004/550px/img0001.jpg
https://www.dermis.net/bilder/CD048/550px/img0030.jpg
https://www.dermis.net/bilder/CD002/550px/img0090.jpg
https://www.dermis.net/bilder/CD049/550px/img0046.jpg
https://www.dermis.net/bilder/CD065/550px/img0103.jpg
https://www.dermis.net/bilder/CD058/550px/img0074.jpg
https://www.dermis.net/bilder/CD057/550px/img0033.jpg
https://www.dermis.net/bilder/CD057/550px/img0008.jpg
https://www.dermis.net/bilder/CD057/550px/img0012.jpg
https://www.dermis.net/bilder/CD048/550px/img0106.jpg
https://www.dermis.net/bilder/CD049/550px/img0075.jpg
https://www.dermis.net/bilder/CD051/550px/img0034.jpg
https://www.dermis.net/bilder/CD051/550px/img0035.jpg
https://www.dermis.net/bilder/CD039/550px/img0098.jpg
https://www.dermis.net/bilder/CD067/550px/img0106.jpg
https://www.dermis.net/bilder/CD067/550px/img0107.jpg
https://www.dermis.net/bilder/CD048/550px/img0040.jpg
https://www.dermis.net/bilder/CD048/550px/img0041.jpg
https://www.dermis.net/bilder/CD048/550px/img0042.jpg
https://www.dermis.net/bilder/CD048/550px/img0043.jpg
https://www.dermis.net/bilder/CD066/550px/img0084.jpg
https://www.dermis.net/bilder/CD066/550px/img0085.jpg
https://www.dermis.net/bilder/CD066/550px/img0100.jpg
https://www.dermis.net/bilder/CD041/550px/img0032.jpg
https://www.dermis.net/bilder/CD067/550px/img0049.jpg
https://www.dermis.net/bilder/CD067/550px/img0050.jpg

https://www.dermis.net/bilder/CD067/550px/img0051.jpg
https://www.dermis.net/bilder/CD067/550px/img0052.jpg
https://www.dermis.net/bilder/CD058/550px/img0037.jpg
https://www.dermis.net/bilder/CD058/550px/img0042.jpg
https://www.dermis.net/bilder/CD058/550px/img0043.jpg
https://www.dermis.net/bilder/CD058/550px/img0044.jpg
https://www.dermis.net/bilder/CD058/550px/img0073.jpg
https://www.dermis.net/bilder/CD058/550px/img0080.jpg
https://www.dermis.net/bilder/CD060/550px/img0044.jpg
https://www.dermis.net/bilder/CD060/550px/img0045.jpg
https://www.dermis.net/bilder/CD047/550px/img0097.jpg
https://www.dermis.net/bilder/CD048/550px/img0105.jpg
https://www.dermis.net/bilder/CD047/550px/img0040.jpg
https://www.dermis.net/bilder/CD066/550px/img0016.jpg
https://www.dermis.net/bilder/CD066/550px/img0017.jpg
https://www.dermis.net/bilder/CD066/550px/img0018.jpg
https://www.dermis.net/bilder/CD066/550px/img0019.jpg
https://www.dermis.net/bilder/CD066/550px/img0042.jpg
https://www.dermis.net/bilder/CD066/550px/img0043.jpg
https://www.dermis.net/bilder/CD066/550px/img0044.jpg
https://www.dermis.net/bilder/CD066/550px/img0020.jpg
https://www.dermis.net/bilder/CD014/550px/img0017.jpg
https://www.dermis.net/bilder/CD040/550px/img0058.jpg
https://www.dermis.net/bilder/CD050/550px/img0103.jpg
https://www.dermis.net/bilder/CD046/550px/img0071.jpg
https://www.dermis.net/bilder/CD060/550px/img0088.jpg
https://www.dermis.net/bilder/CD060/550px/img0089.jpg
https://www.dermis.net/bilder/CD067/550px/img0075.jpg
https://www.dermis.net/bilder/CD067/550px/img0076.jpg
https://www.dermis.net/bilder/CD049/550px/img0073.jpg
https://www.dermis.net/bilder/CD049/550px/img0077.jpg
https://www.dermis.net/bilder/CD054/550px/img0047.jpg
https://www.dermis.net/bilder/CD048/550px/img0102.jpg
https://www.dermis.net/bilder/CD051/550px/img0099.jpg
https://www.dermis.net/bilder/CD050/550px/img0049.jpg
https://www.dermis.net/bilder/CD050/550px/img0050.jpg
https://www.dermis.net/bilder/CD050/550px/img0051.jpg
https://www.dermis.net/bilder/CD065/550px/img0004.jpg
https://www.dermis.net/bilder/CD067/550px/img0105.jpg
https://www.dermis.net/bilder/CD014/550px/img0094.jpg
https://www.dermis.net/bilder/CD058/550px/img0060.jpg
https://www.dermis.net/bilder/CD065/550px/img0022.jpg
https://www.dermis.net/bilder/CD046/550px/img0075.jpg
https://www.dermis.net/bilder/CD027/550px/img0099.jpg
https://www.dermis.net/bilder/CD067/550px/img0078.jpg
https://www.dermis.net/bilder/CD067/550px/img0079.jpg
https://www.dermis.net/bilder/CD036/550px/img0014.jpg
https://www.dermis.net/bilder/CD023/550px/img0017.jpg

https://www.dermis.net/bilder/CD049/550px/img0099.jpg
https://www.dermis.net/bilder/CD052/550px/img0001.jpg
https://www.dermis.net/bilder/CD052/550px/img0002.jpg
https://www.dermis.net/bilder/CD018/550px/img0048.jpg
https://www.dermis.net/bilder/CD036/550px/img0044.jpg
https://www.dermis.net/bilder/CD014/550px/img0064.jpg
https://www.dermis.net/bilder/CD003/550px/img0051.jpg
https://www.dermis.net/bilder/CD046/550px/img0072.jpg
https://www.dermis.net/bilder/CD018/550px/img0095.jpg
https://www.dermis.net/bilder/CD065/550px/img0054.jpg
https://www.dermis.net/bilder/CD154/550px/img0091.jpg
https://www.dermis.net/bilder/CD018/550px/img0008.jpg
https://www.dermis.net/bilder/CD058/550px/img0058.jpg
https://www.dermis.net/bilder/CD049/550px/img0097.jpg
https://www.dermis.net/bilder/CD037/550px/img0003.jpg
https://www.dermis.net/bilder/CD058/550px/img0023.jpg
https://www.dermis.net/bilder/CD058/550px/img0024.jpg
https://www.dermis.net/bilder/CD067/550px/img0013.jpg
https://www.dermis.net/bilder/CD067/550px/img0014.jpg
https://www.dermis.net/bilder/CD067/550px/img0016.jpg
https://www.dermis.net/bilder/CD067/550px/img0067.jpg
https://www.dermis.net/bilder/CD067/550px/img0068.jpg
https://www.dermis.net/bilder/CD052/550px/img0020.jpg
https://www.dermis.net/bilder/CD058/550px/img0057.jpg
https://www.dermis.net/bilder/CD065/550px/img0021.jpg
https://www.dermis.net/bilder/CD057/550px/img0020.jpg
https://www.dermis.net/bilder/CD066/550px/img0077.jpg
https://www.dermis.net/bilder/CD066/550px/img0078.jpg
https://www.dermis.net/bilder/CD060/550px/img0054.jpg
https://www.dermis.net/bilder/CD058/550px/img0088.jpg
https://www.dermis.net/bilder/CD060/550px/img0055.jpg
https://www.dermis.net/bilder/CD060/550px/img0056.jpg
https://www.dermis.net/bilder/CD060/550px/img0053.jpg
https://www.dermis.net/bilder/CD047/550px/img0091.jpg
https://www.dermis.net/bilder/CD047/550px/img0093.jpg
https://www.dermis.net/bilder/CD066/550px/img0027.jpg
https://www.dermis.net/bilder/CD066/550px/img0029.jpg
https://www.dermis.net/bilder/CD066/550px/img0030.jpg
https://www.dermis.net/bilder/CD066/550px/img0031.jpg
https://www.dermis.net/bilder/CD059/550px/img0073.jpg
https://www.dermis.net/bilder/CD059/550px/img0074.jpg
https://www.dermis.net/bilder/CD059/550px/img0075.jpg
https://www.dermis.net/bilder/CD059/550px/img0076.jpg
https://www.dermis.net/bilder/CD024/550px/img0004.jpg
https://www.dermis.net/bilder/CD052/550px/img0013.jpg
https://www.dermis.net/bilder/CD063/550px/img0074.jpg
https://www.dermis.net/bilder/CD039/550px/img0044.jpg
https://www.dermis.net/bilder/CD060/550px/img0057.jpg

https://www.dermis.net/bilder/CD051/550px/img0065.jpg
https://www.dermis.net/bilder/CD051/550px/img0067.jpg
https://www.dermis.net/bilder/CD051/550px/img0068.jpg
https://www.dermis.net/bilder/CD060/550px/img0081.jpg
https://www.dermis.net/bilder/CD041/550px/img0015.jpg
https://www.dermis.net/bilder/CD014/550px/img0096.jpg
https://www.dermis.net/bilder/CD040/550px/img0022.jpg
https://www.dermis.net/bilder/CD060/550px/img0059.jpg
https://www.dermis.net/bilder/CD060/550px/img0060.jpg
https://www.dermis.net/bilder/CD040/550px/img0079.jpg
https://www.dermis.net/bilder/CD067/550px/img0081.jpg
https://www.dermis.net/bilder/CD025/550px/img0082.jpg
https://www.dermis.net/bilder/CD032/550px/img0086.jpg
https://www.dermis.net/bilder/CD039/550px/img0055.jpg
https://www.dermis.net/bilder/CD050/550px/img0055.jpg
https://www.dermis.net/bilder/CD064/550px/img0071.jpg
https://www.dermis.net/bilder/CD064/550px/img0072.jpg
https://www.dermis.net/bilder/CD007/550px/img0010.jpg
https://www.dermis.net/bilder/CD067/550px/img0030.jpg
https://www.dermis.net/bilder/CD067/550px/img0031.jpg
https://www.dermis.net/bilder/CD067/550px/img0032.jpg
https://www.dermis.net/bilder/CD064/550px/img0099.jpg
https://www.dermis.net/bilder/CD064/550px/img0100.jpg
https://www.dermis.net/bilder/CD063/550px/img0072.jpg
https://www.dermis.net/bilder/CD066/550px/img0069.jpg
https://www.dermis.net/bilder/CD057/550px/img0027.jpg
https://www.dermis.net/bilder/CD060/550px/img0049.jpg
https://www.dermis.net/bilder/CD060/550px/img0050.jpg
https://www.dermis.net/bilder/CD005/550px/img0100.jpg
https://www.dermis.net/bilder/CD020/550px/img0087.jpg
https://www.dermis.net/bilder/CD032/550px/img0027.jpg
https://www.dermis.net/bilder/CD032/550px/img0028.jpg
https://www.dermis.net/bilder/CD027/550px/img0083.jpg
https://www.dermis.net/bilder/CD026/550px/img0097.jpg
https://www.dermis.net/bilder/CD063/550px/img0089.jpg
https://www.dermis.net/bilder/CD048/550px/img0054.jpg
https://www.dermis.net/bilder/CD048/550px/img0055.jpg
https://www.dermis.net/bilder/CD048/550px/img0056.jpg
https://www.dermis.net/bilder/CD060/550px/img0070.jpg
https://www.dermis.net/bilder/CD027/550px/img0085.jpg
https://www.dermis.net/bilder/CD048/550px/img0103.jpg
https://www.dermis.net/bilder/CD057/550px/img0028.jpg
https://www.dermis.net/bilder/CD067/550px/img0097.jpg
https://www.dermis.net/bilder/CD024/550px/img0029.jpg
https://www.dermis.net/bilder/CD064/550px/img0104.jpg
https://www.dermis.net/bilder/CD005/550px/img0088.jpg
https://www.dermis.net/bilder/CD032/550px/img0032.jpg
https://www.dermis.net/bilder/CD154/550px/img0084.jpg

https://www.dermis.net/bilder/CD154/550px/img0085.jpg
https://www.dermis.net/bilder/CD154/550px/img0086.jpg
https://www.dermis.net/bilder/CD154/550px/img0087.jpg
https://www.dermis.net/bilder/CD057/550px/img0045.jpg
https://www.dermis.net/bilder/CD065/550px/img0102.jpg
https://www.dermis.net/bilder/CD067/550px/img0040.jpg
https://www.dermis.net/bilder/CD067/550px/img0041.jpg
https://www.dermis.net/bilder/CD040/550px/img0092.jpg
https://www.dermis.net/bilder/CD067/550px/img0071.jpg
https://www.dermis.net/bilder/CD067/550px/img0069.jpg
https://www.dermis.net/bilder/CD040/550px/img0026.jpg
https://www.dermis.net/bilder/CD040/550px/img0027.jpg
https://www.dermis.net/bilder/CD040/550px/img0028.jpg
https://www.dermis.net/bilder/CD061/550px/img0011.jpg
https://www.dermis.net/bilder/CD067/550px/img0038.jpg
https://www.dermis.net/bilder/CD067/550px/img0039.jpg
https://www.dermis.net/bilder/CD066/550px/img0088.jpg
https://www.dermis.net/bilder/CD066/550px/img0089.jpg
https://www.dermis.net/bilder/CD027/550px/img0057.jpg
https://www.dermis.net/bilder/CD027/550px/img0060.jpg
https://www.dermis.net/bilder/CD039/550px/img0026.jpg
https://www.dermis.net/bilder/CD065/550px/img0045.jpg
https://www.dermis.net/bilder/CD065/550px/img0046.jpg
https://www.dermis.net/bilder/CD054/550px/img0083.jpg
https://www.dermis.net/bilder/CD060/550px/img0082.jpg
https://www.dermis.net/bilder/CD060/550px/img0083.jpg
https://www.dermis.net/bilder/CD005/550px/img0106.jpg
https://www.dermis.net/bilder/CD044/550px/img0065.jpg
https://www.dermis.net/bilder/CD065/550px/img0068.jpg
https://www.dermis.net/bilder/CD065/550px/img0069.jpg
https://www.dermis.net/bilder/CD065/550px/img0070.jpg
https://www.dermis.net/bilder/CD003/550px/img0013.jpg
https://www.dermis.net/bilder/CD040/550px/img0097.jpg
https://www.dermis.net/bilder/CD051/550px/img0027.jpg
https://www.dermis.net/bilder/CD051/550px/img0028.jpg
https://www.dermis.net/bilder/CD006/550px/img0029.jpg
https://www.dermis.net/bilder/CD059/550px/img0024.jpg
https://www.dermis.net/bilder/CD067/550px/img0104.jpg
https://www.dermis.net/bilder/CD007/550px/img0014.jpg
https://www.dermis.net/bilder/CD064/550px/img0108.jpg
https://www.dermis.net/bilder/CD059/550px/img0033.jpg
https://www.dermis.net/bilder/CD065/550px/img0101.jpg
https://www.dermis.net/bilder/CD032/550px/img0056.jpg
https://www.dermis.net/bilder/CD060/550px/img0090.jpg
https://www.dermis.net/bilder/CD060/550px/img0091.jpg
https://www.dermis.net/bilder/CD060/550px/img0092.jpg
https://www.dermis.net/bilder/CD067/550px/img0088.jpg
https://www.dermis.net/bilder/CD051/550px/img0024.jpg

https://www.dermis.net/bilder/CD046/550px/img0100.jpg
https://www.dermis.net/bilder/CD060/550px/img0096.jpg
https://www.dermis.net/bilder/CD018/550px/img0005.jpg
https://www.dermis.net/bilder/CD049/550px/img0059.jpg
https://www.dermis.net/bilder/CD049/550px/img0060.jpg
https://www.dermis.net/bilder/CD060/550px/img0013.jpg
https://www.dermis.net/bilder/CD060/550px/img0014.jpg
https://www.dermis.net/bilder/CD060/550px/img0015.jpg
https://www.dermis.net/bilder/CD063/550px/img0093.jpg
https://www.dermis.net/bilder/CD006/550px/img0062.jpg
https://www.dermis.net/bilder/CD067/550px/img0091.jpg
https://www.dermis.net/bilder/CD067/550px/img0092.jpg
https://www.dermis.net/bilder/CD048/550px/img0047.jpg
https://www.dermis.net/bilder/CD067/550px/img0086.jpg
https://www.dermis.net/bilder/CD067/550px/img0087.jpg
https://www.dermis.net/bilder/CD047/550px/img0042.jpg
https://www.dermis.net/bilder/CD047/550px/img0043.jpg
https://www.dermis.net/bilder/CD025/550px/img0076.jpg
https://www.dermis.net/bilder/CD047/550px/img0020.jpg
https://www.dermis.net/bilder/CD067/550px/img0053.jpg
https://www.dermis.net/bilder/CD067/550px/img0054.jpg
https://www.dermis.net/bilder/CD067/550px/img0055.jpg
https://www.dermis.net/bilder/CD067/550px/img0056.jpg
https://www.dermis.net/bilder/CD059/550px/img0077.jpg
https://www.dermis.net/bilder/CD059/550px/img0078.jpg
https://www.dermis.net/bilder/CD059/550px/img0080.jpg
https://www.dermis.net/bilder/CD063/550px/img0097.jpg
https://www.dermis.net/bilder/CD063/550px/img0059.jpg
https://www.dermis.net/bilder/CD063/550px/img0060.jpg
https://www.dermis.net/bilder/CD067/550px/img0074.jpg
https://www.dermis.net/bilder/CD048/550px/img0067.jpg
https://www.dermis.net/bilder/CD052/550px/img0045.jpg
https://www.dermis.net/bilder/CD035/550px/img0017.jpg
https://www.dermis.net/bilder/CD027/550px/img0081.jpg
https://www.dermis.net/bilder/CD027/550px/img0082.jpg
https://www.dermis.net/bilder/CD034/550px/img0061.jpg
https://www.dermis.net/bilder/CD033/550px/img0056.jpg
https://www.dermis.net/bilder/CD041/550px/img0031.jpg
https://www.dermis.net/bilder/CD063/550px/img0088.jpg
https://www.dermis.net/bilder/CD063/550px/img0075.jpg
https://www.dermis.net/bilder/CD063/550px/img0076.jpg
https://www.dermis.net/bilder/CD005/550px/img0025.jpg
https://www.dermis.net/bilder/CD047/550px/img0019.jpg
https://www.dermis.net/bilder/CD039/550px/img0018.jpg
https://www.dermis.net/bilder/CD047/550px/img0057.jpg
https://www.dermis.net/bilder/CD047/550px/img0059.jpg
https://www.dermis.net/bilder/CD037/550px/img0072.jpg
https://www.dermis.net/bilder/CD057/550px/img0031.jpg

https://www.dermis.net/bilder/CD066/550px/img0092.jpg
https://www.dermis.net/bilder/CD066/550px/img0093.jpg
https://www.dermis.net/bilder/CD034/550px/img0081.jpg
https://www.dermis.net/bilder/CD063/550px/img0102.jpg
https://www.dermis.net/bilder/CD063/550px/img0103.jpg
https://www.dermis.net/bilder/CD060/550px/img0066.jpg
https://www.dermis.net/bilder/CD060/550px/img0067.jpg
https://www.dermis.net/bilder/CD060/550px/img0068.jpg
https://www.dermis.net/bilder/CD060/550px/img0072.jpg
https://www.dermis.net/bilder/CD058/550px/img0005.jpg
https://www.dermis.net/bilder/CD058/550px/img0011.jpg
https://www.dermis.net/bilder/CD058/550px/img0012.jpg
https://www.dermis.net/bilder/CD052/550px/img0017.jpg
https://www.dermis.net/bilder/CD065/550px/img0019.jpg
https://www.dermis.net/bilder/CD057/550px/img0030.jpg
https://www.dermis.net/bilder/CD007/550px/img0017.jpg
https://www.dermis.net/bilder/CD040/550px/img0061.jpg
https://www.dermis.net/bilder/CD032/550px/img0096.jpg
https://www.dermis.net/bilder/CD066/550px/img0046.jpg
https://www.dermis.net/bilder/CD066/550px/img0047.jpg
https://www.dermis.net/bilder/CD066/550px/img0048.jpg
https://www.dermis.net/bilder/CD154/550px/img0036.jpg
https://www.dermis.net/bilder/CD020/550px/img0096.jpg
https://www.dermis.net/bilder/CD066/550px/img0082.jpg
https://www.dermis.net/bilder/CD066/550px/img0083.jpg
https://www.dermis.net/bilder/CD016/550px/img0060.jpg
https://www.dermis.net/bilder/CD058/550px/img0082.jpg
https://www.dermis.net/bilder/CD058/550px/img0056.jpg
https://www.dermis.net/bilder/CD058/550px/img0083.jpg
https://www.dermis.net/bilder/CD066/550px/img0094.jpg
https://www.dermis.net/bilder/CD066/550px/img0095.jpg
https://www.dermis.net/bilder/CD052/550px/img0025.jpg
https://www.dermis.net/bilder/CD049/550px/img0086.jpg
https://www.dermis.net/bilder/CD058/550px/img0055.jpg
https://www.dermis.net/bilder/CD059/550px/img0054.jpg
https://www.dermis.net/bilder/CD059/550px/img0055.jpg
https://www.dermis.net/bilder/CD059/550px/img0056.jpg
https://www.dermis.net/bilder/CD048/550px/img0076.jpg
https://www.dermis.net/bilder/CD054/550px/img0080.jpg
https://www.dermis.net/bilder/CD054/550px/img0081.jpg
https://www.dermis.net/bilder/CD054/550px/img0082.jpg
https://www.dermis.net/bilder/CD028/550px/img0069.jpg
https://www.dermis.net/bilder/CD028/550px/img0070.jpg
https://www.dermis.net/bilder/CD063/550px/img0018.jpg
https://www.dermis.net/bilder/CD063/550px/img0019.jpg
https://www.dermis.net/bilder/CD063/550px/img0020.jpg
https://www.dermis.net/bilder/CD063/550px/img0021.jpg
https://www.dermis.net/bilder/CD037/550px/img0012.jpg

https://www.dermis.net/bilder/CD032/550px/img0075.jpg
https://www.dermis.net/bilder/CD060/550px/img0106.jpg
https://www.dermis.net/bilder/CD026/550px/img0045.jpg
https://www.dermis.net/bilder/CD018/550px/img0098.jpg
https://www.dermis.net/bilder/CD051/550px/img0036.jpg
https://www.dermis.net/bilder/CD018/550px/img0010.jpg
https://www.dermis.net/bilder/CD003/550px/img0085.jpg
https://www.dermis.net/bilder/CD057/550px/img0022.jpg
https://www.dermis.net/bilder/CD059/550px/img0027.jpg
https://www.dermis.net/bilder/CD059/550px/img0028.jpg
https://www.dermis.net/bilder/CD060/550px/img0038.jpg
https://www.dermis.net/bilder/CD046/550px/img0032.jpg
https://www.dermis.net/bilder/CD060/550px/img0084.jpg
https://www.dermis.net/bilder/CD066/550px/img0049.jpg
https://www.dermis.net/bilder/CD066/550px/img0050.jpg
https://www.dermis.net/bilder/CD014/550px/img0051.jpg
https://www.dermis.net/bilder/CD066/550px/img0058.jpg
https://www.dermis.net/bilder/CD066/550px/img0059.jpg
https://www.dermis.net/bilder/CD042/550px/img0051.jpg
https://www.dermis.net/bilder/CD060/550px/img0037.jpg
https://www.dermis.net/bilder/CD066/550px/img0051.jpg
https://www.dermis.net/bilder/CD066/550px/img0052.jpg
https://www.dermis.net/bilder/CD060/550px/img0040.jpg
https://www.dermis.net/bilder/CD060/550px/img0041.jpg
https://www.dermis.net/bilder/CD058/550px/img0050.jpg
https://www.dermis.net/bilder/CD066/550px/img0071.jpg
https://www.dermis.net/bilder/CD066/550px/img0072.jpg
https://www.dermis.net/bilder/CD018/550px/img0017.jpg
https://www.dermis.net/bilder/CD057/550px/img0025.jpg
https://www.dermis.net/bilder/CD057/550px/img0026.jpg
https://www.dermis.net/bilder/CD059/550px/img0064.jpg
https://www.dermis.net/bilder/CD059/550px/img0065.jpg
https://www.dermis.net/bilder/CD059/550px/img0066.jpg
https://www.dermis.net/bilder/CD063/550px/img0041.jpg
https://www.dermis.net/bilder/CD063/550px/img0042.jpg
https://www.dermis.net/bilder/CD063/550px/img0043.jpg
https://www.dermis.net/bilder/CD059/550px/img0067.jpg
https://www.dermis.net/bilder/CD039/550px/img0031.jpg
https://www.dermis.net/bilder/CD057/550px/img0046.jpg
https://www.dermis.net/bilder/CD057/550px/img0047.jpg
https://www.dermis.net/bilder/CD067/550px/img0101.jpg
https://www.dermis.net/bilder/CD067/550px/img0102.jpg
https://www.dermis.net/bilder/CD060/550px/img0071.jpg
https://www.dermis.net/bilder/CD026/550px/img0026.jpg
https://www.dermis.net/bilder/CD026/550px/img0027.jpg
https://www.dermis.net/bilder/CD064/550px/img0107.jpg
https://www.dermis.net/bilder/CD065/550px/img0071.jpg
https://www.dermis.net/bilder/CD066/550px/img0008.jpg

https://www.dermis.net/bilder/CD066/550px/img0009.jpg
https://www.dermis.net/bilder/CD058/550px/img0066.jpg
https://www.dermis.net/bilder/CD058/550px/img0067.jpg
https://www.dermis.net/bilder/CD058/550px/img0068.jpg
https://www.dermis.net/bilder/CD035/550px/img0053.jpg
https://www.dermis.net/bilder/CD046/550px/img0049.jpg
https://www.dermis.net/bilder/CD058/550px/img0039.jpg
https://www.dermis.net/bilder/CD036/550px/img0095.jpg
https://www.dermis.net/bilder/CD003/550px/img0022.jpg
https://www.dermis.net/bilder/CD052/550px/img0027.jpg
https://www.dermis.net/bilder/CD052/550px/img0028.jpg
https://www.dermis.net/bilder/CD087/550px/img0068.jpg
https://www.dermis.net/bilder/CD085/550px/img0100.jpg
https://www.dermis.net/bilder/CD088/550px/img0024.jpg
https://www.dermis.net/bilder/CD088/550px/img0025.jpg
https://www.dermis.net/bilder/CD093/550px/img0001.jpg
https://www.dermis.net/bilder/CD093/550px/img0002.jpg
https://www.dermis.net/bilder/CD093/550px/img0003.jpg
https://www.dermis.net/bilder/CD093/550px/img0004.jpg
https://www.dermis.net/bilder/CD093/550px/img0005.jpg
https://www.dermis.net/bilder/CD093/550px/img0006.jpg
https://www.dermis.net/bilder/CD082/550px/img0001.jpg
https://www.dermis.net/bilder/CD082/550px/img0041.jpg
https://www.dermis.net/bilder/CD095/550px/img0043.jpg
https://www.dermis.net/bilder/CD103/550px/img0010.jpg
https://www.dermis.net/bilder/CD106/550px/img0059.jpg
https://www.dermis.net/bilder/CD106/550px/img0062.jpg
https://www.dermis.net/bilder/CD106/550px/img0064.jpg
https://www.dermis.net/bilder/CD099/550px/img0007.jpg
https://www.dermis.net/bilder/CD085/550px/img0008.jpg
https://www.dermis.net/bilder/CD085/550px/img0009.jpg
https://www.dermis.net/bilder/CD085/550px/img0011.jpg
https://www.dermis.net/bilder/CD106/550px/img0071.jpg
https://www.dermis.net/bilder/CD093/550px/img0041.jpg
https://www.dermis.net/bilder/CD098/550px/img0013.jpg
https://www.dermis.net/bilder/CD099/550px/img0027.jpg
https://www.dermis.net/bilder/CD099/550px/img0029.jpg
https://www.dermis.net/bilder/CD086/550px/img0044.jpg
https://www.dermis.net/bilder/CD087/550px/img0080.jpg
https://www.dermis.net/bilder/CD085/550px/img0082.jpg
https://www.dermis.net/bilder/CD085/550px/img0083.jpg
https://www.dermis.net/bilder/CD085/550px/img0084.jpg
https://www.dermis.net/bilder/CD085/550px/img0085.jpg
https://www.dermis.net/bilder/CD097/550px/img0091.jpg
https://www.dermis.net/bilder/CD085/550px/img0040.jpg
https://www.dermis.net/bilder/CD085/550px/img0042.jpg
https://www.dermis.net/bilder/CD085/550px/img0043.jpg
https://www.dermis.net/bilder/CD107/550px/img0016.jpg

https://www.dermis.net/bilder/CD107/550px/img0018.jpg
https://www.dermis.net/bilder/CD088/550px/img0022.jpg
https://www.dermis.net/bilder/CD106/550px/img0072.jpg
https://www.dermis.net/bilder/CD106/550px/img0073.jpg
https://www.dermis.net/bilder/CD106/550px/img0074.jpg
https://www.dermis.net/bilder/CD087/550px/img0010.jpg
https://www.dermis.net/bilder/CD087/550px/img0011.jpg
https://www.dermis.net/bilder/CD097/550px/img0032.jpg
https://www.dermis.net/bilder/CD097/550px/img0033.jpg
https://www.dermis.net/bilder/CD093/550px/img0059.jpg
https://www.dermis.net/bilder/CD093/550px/img0088.jpg
https://www.dermis.net/bilder/CD093/550px/img0089.jpg
https://www.dermis.net/bilder/CD093/550px/img0090.jpg
https://www.dermis.net/bilder/CD098/550px/img0059.jpg
https://www.dermis.net/bilder/CD098/550px/img0060.jpg
https://www.dermis.net/bilder/CD098/550px/img0061.jpg
https://www.dermis.net/bilder/CD096/550px/img0001.jpg
https://www.dermis.net/bilder/CD098/550px/img0021.jpg
https://www.dermis.net/bilder/CD085/550px/img0044.jpg
https://www.dermis.net/bilder/CD085/550px/img0096.jpg
https://www.dermis.net/bilder/CD085/550px/img0097.jpg
https://www.dermis.net/bilder/CD085/550px/img0099.jpg
https://www.dermis.net/bilder/CD098/550px/img0025.jpg
https://www.dermis.net/bilder/CD107/550px/img0054.jpg
https://www.dermis.net/bilder/CD107/550px/img0055.jpg
https://www.dermis.net/bilder/CD107/550px/img0056.jpg
https://www.dermis.net/bilder/CD097/550px/img0055.jpg
https://www.dermis.net/bilder/CD085/550px/img0033.jpg
https://www.dermis.net/bilder/CD085/550px/img0034.jpg
https://www.dermis.net/bilder/CD085/550px/img0035.jpg
https://www.dermis.net/bilder/CD087/550px/img0090.jpg
https://www.dermis.net/bilder/CD088/550px/img0001.jpg
https://www.dermis.net/bilder/CD088/550px/img0003.jpg
https://www.dermis.net/bilder/CD085/550px/img0052.jpg
https://www.dermis.net/bilder/CD085/550px/img0053.jpg
https://www.dermis.net/bilder/CD085/550px/img0054.jpg
https://www.dermis.net/bilder/CD085/550px/img0055.jpg
https://www.dermis.net/bilder/CD087/550px/img0099.jpg
https://www.dermis.net/bilder/CD087/550px/img0100.jpg
https://www.dermis.net/bilder/CD086/550px/img0096.jpg
https://www.dermis.net/bilder/CD085/550px/img0089.jpg
https://www.dermis.net/bilder/CD107/550px/img0040.jpg
https://www.dermis.net/bilder/CD107/550px/img0041.jpg
https://www.dermis.net/bilder/CD107/550px/img0042.jpg
https://www.dermis.net/bilder/CD088/550px/img0093.jpg
https://www.dermis.net/bilder/CD088/550px/img0094.jpg
https://www.dermis.net/bilder/CD088/550px/img0095.jpg
https://www.dermis.net/bilder/CD099/550px/img0017.jpg

https://www.dermis.net/bilder/CD099/550px/img0025.jpg
https://www.dermis.net/bilder/CD099/550px/img0026.jpg
https://www.dermis.net/bilder/CD085/550px/img0037.jpg
https://www.dermis.net/bilder/CD085/550px/img0039.jpg
https://www.dermis.net/bilder/CD088/550px/img0031.jpg
https://www.dermis.net/bilder/CD088/550px/img0032.jpg
https://www.dermis.net/bilder/CD088/550px/img0033.jpg
https://www.dermis.net/bilder/CD088/550px/img0034.jpg
https://www.dermis.net/bilder/CD088/550px/img0035.jpg
https://www.dermis.net/bilder/CD099/550px/img0028.jpg
https://www.dermis.net/bilder/CD099/550px/img0030.jpg
https://www.dermis.net/bilder/CD099/550px/img0032.jpg
https://www.dermis.net/bilder/CD098/550px/img0072.jpg
https://www.dermis.net/bilder/CD097/550px/img0090.jpg
https://www.dermis.net/bilder/CD085/550px/img0001.jpg
https://www.dermis.net/bilder/CD085/550px/img0003.jpg
https://www.dermis.net/bilder/CD085/550px/img0004.jpg
https://www.dermis.net/bilder/CD098/550px/img0032.jpg
https://www.dermis.net/bilder/CD098/550px/img0037.jpg
https://www.dermis.net/bilder/CD098/550px/img0038.jpg
https://www.dermis.net/bilder/CD098/550px/img0039.jpg
https://www.dermis.net/bilder/CD098/550px/img0040.jpg
https://www.dermis.net/bilder/CD097/550px/img0073.jpg
https://www.dermis.net/bilder/CD097/550px/img0074.jpg
https://www.dermis.net/bilder/CD096/550px/img0051.jpg
https://www.dermis.net/bilder/CD085/550px/img0090.jpg
https://www.dermis.net/bilder/CD085/550px/img0091.jpg
https://www.dermis.net/bilder/CD085/550px/img0092.jpg
https://www.dermis.net/bilder/CD085/550px/img0093.jpg
https://www.dermis.net/bilder/CD085/550px/img0094.jpg
https://www.dermis.net/bilder/CD085/550px/img0095.jpg
https://www.dermis.net/bilder/CD093/550px/img0080.jpg
https://www.dermis.net/bilder/CD093/550px/img0081.jpg
https://www.dermis.net/bilder/CD093/550px/img0082.jpg
https://www.dermis.net/bilder/CD106/550px/img0010.jpg
https://www.dermis.net/bilder/CD093/550px/img0058.jpg
https://www.dermis.net/bilder/CD093/550px/img0060.jpg
https://www.dermis.net/bilder/CD093/550px/img0087.jpg
https://www.dermis.net/bilder/CD097/550px/img0081.jpg
https://www.dermis.net/bilder/CD086/550px/img0019.jpg
https://www.dermis.net/bilder/CD086/550px/img0022.jpg
https://www.dermis.net/bilder/CD086/550px/img0023.jpg
https://www.dermis.net/bilder/CD088/550px/img0057.jpg
https://www.dermis.net/bilder/CD098/550px/img0018.jpg
https://www.dermis.net/bilder/CD098/550px/img0019.jpg
https://www.dermis.net/bilder/CD088/550px/img0028.jpg
https://www.dermis.net/bilder/CD106/550px/img0075.jpg
https://www.dermis.net/bilder/CD107/550px/img0028.jpg

https://www.dermis.net/bilder/CD085/550px/img0018.jpg
https://www.dermis.net/bilder/CD085/550px/img0019.jpg
https://www.dermis.net/bilder/CD088/550px/img0036.jpg
https://www.dermis.net/bilder/CD085/550px/img0038.jpg
https://www.dermis.net/bilder/CD087/550px/img0091.jpg
https://www.dermis.net/bilder/CD087/550px/img0092.jpg
https://www.dermis.net/bilder/CD097/550px/img0061.jpg
https://www.dermis.net/bilder/CD097/550px/img0062.jpg
https://www.dermis.net/bilder/CD097/550px/img0063.jpg
https://www.dermis.net/bilder/CD097/550px/img0064.jpg
https://www.dermis.net/bilder/CD085/550px/img0045.jpg
https://www.dermis.net/bilder/CD085/550px/img0046.jpg
https://www.dermis.net/bilder/CD085/550px/img0047.jpg
https://www.dermis.net/bilder/CD085/550px/img0048.jpg
https://www.dermis.net/bilder/CD085/550px/img0049.jpg
https://www.dermis.net/bilder/CD099/550px/img0013.jpg
https://www.dermis.net/bilder/CD099/550px/img0061.jpg
https://www.dermis.net/bilder/CD099/550px/img0062.jpg
https://www.dermis.net/bilder/CD088/550px/img0064.jpg
https://www.dermis.net/bilder/CD088/550px/img0065.jpg
https://www.dermis.net/bilder/CD088/550px/img0023.jpg
https://www.dermis.net/bilder/CD087/550px/img0013.jpg
https://www.dermis.net/bilder/CD087/550px/img0014.jpg
https://www.dermis.net/bilder/CD087/550px/img0072.jpg
https://www.dermis.net/bilder/CD087/550px/img0073.jpg
https://www.dermis.net/bilder/CD093/550px/img0025.jpg
https://www.dermis.net/bilder/CD087/550px/img0042.jpg
https://www.dermis.net/bilder/CD087/550px/img0044.jpg
https://www.dermis.net/bilder/CD087/550px/img0045.jpg
https://www.dermis.net/bilder/CD086/550px/img0099.jpg
https://www.dermis.net/bilder/CD085/550px/img0056.jpg
https://www.dermis.net/bilder/CD085/550px/img0057.jpg
https://www.dermis.net/bilder/CD085/550px/img0058.jpg
https://www.dermis.net/bilder/CD099/550px/img0075.jpg
https://www.dermis.net/bilder/CD099/550px/img0076.jpg
https://www.dermis.net/bilder/CD097/550px/img0021.jpg
https://www.dermis.net/bilder/CD097/550px/img0022.jpg
https://www.dermis.net/bilder/CD097/550px/img0023.jpg
https://www.dermis.net/bilder/CD097/550px/img0030.jpg
https://www.dermis.net/bilder/CD104/550px/img0038.jpg
https://www.dermis.net/bilder/CD107/550px/img0015.jpg
https://www.dermis.net/bilder/CD109/550px/img0068.jpg
https://www.dermis.net/bilder/CD103/550px/img0026.jpg
https://www.dermis.net/bilder/CD103/550px/img0031.jpg
https://www.dermis.net/bilder/CD104/550px/img0066.jpg
https://www.dermis.net/bilder/CD105/550px/img0014.jpg
https://www.dermis.net/bilder/CD106/550px/img0058.jpg
https://www.dermis.net/bilder/CD106/550px/img0065.jpg

https://www.dermis.net/bilder/CD106/550px/img0068.jpg
https://www.dermis.net/bilder/CD109/550px/img0013.jpg
https://www.dermis.net/bilder/CD098/550px/img0041.jpg
https://www.dermis.net/bilder/CD099/550px/img0098.jpg
https://www.dermis.net/bilder/CD098/550px/img0014.jpg
https://www.dermis.net/bilder/CD098/550px/img0015.jpg
https://www.dermis.net/bilder/CD098/550px/img0016.jpg
https://www.dermis.net/bilder/CD099/550px/img0084.jpg
https://www.dermis.net/bilder/CD087/550px/img0074.jpg
https://www.dermis.net/bilder/CD086/550px/img0045.jpg
https://www.dermis.net/bilder/CD098/550px/img0053.jpg
https://www.dermis.net/bilder/CD085/550px/img0010.jpg
https://www.dermis.net/bilder/CD099/550px/img0016.jpg
https://www.dermis.net/bilder/CD099/550px/img0066.jpg
https://www.dermis.net/bilder/CD087/550px/img0060.jpg
https://www.dermis.net/bilder/CD087/550px/img0061.jpg
https://www.dermis.net/bilder/CD086/550px/img0100.jpg
https://www.dermis.net/bilder/CD088/550px/img0013.jpg
https://www.dermis.net/bilder/CD088/550px/img0014.jpg
https://www.dermis.net/bilder/CD087/550px/img0064.jpg
https://www.dermis.net/bilder/CD087/550px/img0065.jpg
https://www.dermis.net/bilder/CD087/550px/img0066.jpg
https://www.dermis.net/bilder/CD093/550px/img0040.jpg
https://www.dermis.net/bilder/CD093/550px/img0042.jpg
https://www.dermis.net/bilder/CD093/550px/img0043.jpg
https://www.dermis.net/bilder/CD106/550px/img0033.jpg
https://www.dermis.net/bilder/CD107/550px/img0022.jpg
https://www.dermis.net/bilder/CD110/550px/img0091.jpg
https://www.dermis.net/bilder/CD088/550px/img0008.jpg
https://www.dermis.net/bilder/CD107/550px/img0057.jpg
https://www.dermis.net/bilder/CD107/550px/img0058.jpg
https://www.dermis.net/bilder/CD094/550px/img0020.jpg
https://www.dermis.net/bilder/CD088/550px/img0083.jpg
https://www.dermis.net/bilder/CD088/550px/img0084.jpg
https://www.dermis.net/bilder/CD097/550px/img0014.jpg
https://www.dermis.net/bilder/CD097/550px/img0015.jpg
https://www.dermis.net/bilder/CD097/550px/img0069.jpg
https://www.dermis.net/bilder/CD097/550px/img0070.jpg
https://www.dermis.net/bilder/CD106/550px/img0024.jpg
https://www.dermis.net/bilder/CD087/550px/img0015.jpg
https://www.dermis.net/bilder/CD087/550px/img0012.jpg
https://www.dermis.net/bilder/CD098/550px/img0043.jpg
https://www.dermis.net/bilder/CD099/550px/img0078.jpg
https://www.dermis.net/bilder/CD086/550px/img0027.jpg
https://www.dermis.net/bilder/CD088/550px/img0029.jpg
https://www.dermis.net/bilder/CD099/550px/img0067.jpg
https://www.dermis.net/bilder/CD097/550px/img0045.jpg
https://www.dermis.net/bilder/CD097/550px/img0046.jpg

https://www.dermis.net/bilder/CD097/550px/img0047.jpg
https://www.dermis.net/bilder/CD098/550px/img0008.jpg
https://www.dermis.net/bilder/CD098/550px/img0009.jpg
https://www.dermis.net/bilder/CD086/550px/img0012.jpg
https://www.dermis.net/bilder/CD086/550px/img0013.jpg
https://www.dermis.net/bilder/CD086/550px/img0014.jpg
https://www.dermis.net/bilder/CD086/550px/img0015.jpg
https://www.dermis.net/bilder/CD086/550px/img0016.jpg
https://www.dermis.net/bilder/CD087/550px/img0067.jpg
https://www.dermis.net/bilder/CD099/550px/img0034.jpg
https://www.dermis.net/bilder/CD099/550px/img0035.jpg
https://www.dermis.net/bilder/CD099/550px/img0037.jpg
https://www.dermis.net/bilder/CD099/550px/img0048.jpg
https://www.dermis.net/bilder/CD099/550px/img0058.jpg
https://www.dermis.net/bilder/CD086/550px/img0052.jpg
https://www.dermis.net/bilder/CD086/550px/img0053.jpg
https://www.dermis.net/bilder/CD086/550px/img0003.jpg
https://www.dermis.net/bilder/CD086/550px/img0004.jpg
https://www.dermis.net/bilder/CD086/550px/img0005.jpg
https://www.dermis.net/bilder/CD086/550px/img0006.jpg
https://www.dermis.net/bilder/CD088/550px/img0006.jpg
https://www.dermis.net/bilder/CD086/550px/img0017.jpg
https://www.dermis.net/bilder/CD087/550px/img0056.jpg
https://www.dermis.net/bilder/CD087/550px/img0057.jpg
https://www.dermis.net/bilder/CD087/550px/img0058.jpg
https://www.dermis.net/bilder/CD097/550px/img0018.jpg
https://www.dermis.net/bilder/CD097/550px/img0019.jpg
https://www.dermis.net/bilder/CD097/550px/img0020.jpg
https://www.dermis.net/bilder/CD099/550px/img0063.jpg
https://www.dermis.net/bilder/CD097/550px/img0028.jpg
https://www.dermis.net/bilder/CD097/550px/img0093.jpg
https://www.dermis.net/bilder/CD097/550px/img0094.jpg
https://www.dermis.net/bilder/CD097/550px/img0095.jpg
https://www.dermis.net/bilder/CD097/550px/img0096.jpg
https://www.dermis.net/bilder/CD097/550px/img0056.jpg
https://www.dermis.net/bilder/CD098/550px/img0067.jpg
https://www.dermis.net/bilder/CD099/550px/img0065.jpg
https://www.dermis.net/bilder/CD093/550px/img0078.jpg
https://www.dermis.net/bilder/CD099/550px/img0097.jpg
https://www.dermis.net/bilder/CD099/550px/img0085.jpg
https://www.dermis.net/bilder/CD099/550px/img0086.jpg
https://www.dermis.net/bilder/CD099/550px/img0087.jpg
https://www.dermis.net/bilder/CD099/550px/img0088.jpg
https://www.dermis.net/bilder/CD099/550px/img0089.jpg
https://www.dermis.net/bilder/CD099/550px/img0020.jpg
https://www.dermis.net/bilder/CD099/550px/img0021.jpg
https://www.dermis.net/bilder/CD099/550px/img0022.jpg
https://www.dermis.net/bilder/CD099/550px/img0023.jpg

https://www.dermis.net/bilder/CD099/550px/img0024.jpg
https://www.dermis.net/bilder/CD086/550px/img0089.jpg
https://www.dermis.net/bilder/CD086/550px/img0090.jpg
https://www.dermis.net/bilder/CD099/550px/img0010.jpg
https://www.dermis.net/bilder/CD099/550px/img0011.jpg
https://www.dermis.net/bilder/CD088/550px/img0080.jpg
https://www.dermis.net/bilder/CD099/550px/img0090.jpg
https://www.dermis.net/bilder/CD099/550px/img0092.jpg
https://www.dermis.net/bilder/CD099/550px/img0093.jpg
https://www.dermis.net/bilder/CD099/550px/img0031.jpg
https://www.dermis.net/bilder/CD098/550px/img0075.jpg
https://www.dermis.net/bilder/CD087/550px/img0007.jpg
https://www.dermis.net/bilder/CD087/550px/img0008.jpg
https://www.dermis.net/bilder/CD097/550px/img0097.jpg
https://www.dermis.net/bilder/CD097/550px/img0098.jpg
https://www.dermis.net/bilder/CD097/550px/img0099.jpg
https://www.dermis.net/bilder/CD097/550px/img0100.jpg
https://www.dermis.net/bilder/CD085/550px/img0002.jpg
https://www.dermis.net/bilder/CD091/550px/img0067.jpg
https://www.dermis.net/bilder/CD099/550px/img0091.jpg
https://www.dermis.net/bilder/CD102/550px/img0030.jpg
https://www.dermis.net/bilder/CD098/550px/img0052.jpg
https://www.dermis.net/bilder/CD098/550px/img0054.jpg
https://www.dermis.net/bilder/CD098/550px/img0056.jpg
https://www.dermis.net/bilder/CD098/550px/img0057.jpg
https://www.dermis.net/bilder/CD098/550px/img0055.jpg
https://www.dermis.net/bilder/CD098/550px/img0058.jpg
https://www.dermis.net/bilder/CD098/550px/img0073.jpg
https://www.dermis.net/bilder/CD098/550px/img0074.jpg
https://www.dermis.net/bilder/CD097/550px/img0066.jpg
https://www.dermis.net/bilder/CD093/550px/img0093.jpg
https://www.dermis.net/bilder/CD096/550px/img0086.jpg
https://www.dermis.net/bilder/CD086/550px/img0029.jpg
https://www.dermis.net/bilder/CD093/550px/img0071.jpg
https://www.dermis.net/bilder/CD093/550px/img0072.jpg
https://www.dermis.net/bilder/CD093/550px/img0075.jpg
https://www.dermis.net/bilder/CD093/550px/img0076.jpg
https://www.dermis.net/bilder/CD093/550px/img0077.jpg
https://www.dermis.net/bilder/CD093/550px/img0079.jpg
https://www.dermis.net/bilder/CD087/550px/img0025.jpg
https://www.dermis.net/bilder/CD086/550px/img0020.jpg
https://www.dermis.net/bilder/CD086/550px/img0021.jpg
https://www.dermis.net/bilder/CD099/550px/img0080.jpg
https://www.dermis.net/bilder/CD098/550px/img0011.jpg
https://www.dermis.net/bilder/CD098/550px/img0012.jpg
https://www.dermis.net/bilder/CD099/550px/img0054.jpg
https://www.dermis.net/bilder/CD097/550px/img0003.jpg
https://www.dermis.net/bilder/CD097/550px/img0004.jpg

https://www.dermis.net/bilder/CD087/550px/img0050.jpg
https://www.dermis.net/bilder/CD088/550px/img0062.jpg
https://www.dermis.net/bilder/CD087/550px/img0048.jpg
https://www.dermis.net/bilder/CD087/550px/img0049.jpg
https://www.dermis.net/bilder/CD088/550px/img0061.jpg
https://www.dermis.net/bilder/CD107/550px/img0043.jpg
https://www.dermis.net/bilder/CD101/550px/img0091.jpg
https://www.dermis.net/bilder/CD101/550px/img0092.jpg
https://www.dermis.net/bilder/CD086/550px/img0085.jpg
https://www.dermis.net/bilder/CD086/550px/img0060.jpg
https://www.dermis.net/bilder/CD086/550px/img0061.jpg
https://www.dermis.net/bilder/CD086/550px/img0062.jpg
https://www.dermis.net/bilder/CD086/550px/img0064.jpg
https://www.dermis.net/bilder/CD086/550px/img0065.jpg
https://www.dermis.net/bilder/CD086/550px/img0091.jpg
https://www.dermis.net/bilder/CD086/550px/img0092.jpg
https://www.dermis.net/bilder/CD086/550px/img0093.jpg
https://www.dermis.net/bilder/CD086/550px/img0094.jpg
https://www.dermis.net/bilder/CD088/550px/img0055.jpg
https://www.dermis.net/bilder/CD088/550px/img0056.jpg
https://www.dermis.net/bilder/CD086/550px/img0033.jpg
https://www.dermis.net/bilder/CD086/550px/img0034.jpg
https://www.dermis.net/bilder/CD088/550px/img0037.jpg
https://www.dermis.net/bilder/CD098/550px/img0002.jpg
https://www.dermis.net/bilder/CD098/550px/img0003.jpg
https://www.dermis.net/bilder/CD098/550px/img0004.jpg
https://www.dermis.net/bilder/CD098/550px/img0005.jpg
https://www.dermis.net/bilder/CD098/550px/img0006.jpg
https://www.dermis.net/bilder/CD098/550px/img0042.jpg
https://www.dermis.net/bilder/CD086/550px/img0086.jpg
https://www.dermis.net/bilder/CD107/550px/img0023.jpg
https://www.dermis.net/bilder/CD107/550px/img0024.jpg
https://www.dermis.net/bilder/CD086/550px/img0057.jpg
https://www.dermis.net/bilder/CD099/550px/img0068.jpg
https://www.dermis.net/bilder/CD099/550px/img0069.jpg
https://www.dermis.net/bilder/CD092/550px/img0037.jpg
https://www.dermis.net/bilder/CD092/550px/img0038.jpg
https://www.dermis.net/bilder/CD092/550px/img0005.jpg
https://www.dermis.net/bilder/CD092/550px/img0025.jpg
https://www.dermis.net/bilder/CD092/550px/img0034.jpg
https://www.dermis.net/bilder/CD092/550px/img0021.jpg
https://www.dermis.net/bilder/CD092/550px/img0036.jpg
https://www.dermis.net/bilder/CD087/550px/img0038.jpg
https://www.dermis.net/bilder/CD087/550px/img0039.jpg
https://www.dermis.net/bilder/CD099/550px/img0073.jpg
https://www.dermis.net/bilder/CD099/550px/img0074.jpg
https://www.dermis.net/bilder/CD086/550px/img0046.jpg
https://www.dermis.net/bilder/CD086/550px/img0047.jpg

https://www.dermis.net/bilder/CD098/550px/img0068.jpg
https://www.dermis.net/bilder/CD093/550px/img0046.jpg
https://www.dermis.net/bilder/CD097/550px/img0026.jpg
https://www.dermis.net/bilder/CD097/550px/img0027.jpg
https://www.dermis.net/bilder/CD097/550px/img0029.jpg
https://www.dermis.net/bilder/CD087/550px/img0022.jpg
https://www.dermis.net/bilder/CD087/550px/img0001.jpg
https://www.dermis.net/bilder/CD087/550px/img0002.jpg
https://www.dermis.net/bilder/CD087/550px/img0003.jpg
https://www.dermis.net/bilder/CD107/550px/img0013.jpg
https://www.dermis.net/bilder/CD088/550px/img0058.jpg
https://www.dermis.net/bilder/CD086/550px/img0025.jpg
https://www.dermis.net/bilder/CD086/550px/img0074.jpg
https://www.dermis.net/bilder/CD098/550px/img0010.jpg
https://www.dermis.net/bilder/CD098/550px/img0062.jpg
https://www.dermis.net/bilder/CD098/550px/img0017.jpg
https://www.dermis.net/bilder/CD086/550px/img0007.jpg
https://www.dermis.net/bilder/CD098/550px/img0066.jpg
https://www.dermis.net/bilder/CD106/550px/img0045.jpg
https://www.dermis.net/bilder/CD086/550px/img0097.jpg
https://www.dermis.net/bilder/CD099/550px/img0064.jpg
https://www.dermis.net/bilder/CD097/550px/img0084.jpg
https://www.dermis.net/bilder/CD086/550px/img0055.jpg
https://www.dermis.net/bilder/CD097/550px/img0087.jpg
https://www.dermis.net/bilder/CD097/550px/img0049.jpg
https://www.dermis.net/bilder/CD097/550px/img0050.jpg
https://www.dermis.net/bilder/CD093/550px/img0030.jpg
https://www.dermis.net/bilder/CD093/550px/img0031.jpg
https://www.dermis.net/bilder/CD093/550px/img0032.jpg
https://www.dermis.net/bilder/CD093/550px/img0033.jpg
https://www.dermis.net/bilder/CD098/550px/img0064.jpg
https://www.dermis.net/bilder/CD099/550px/img0083.jpg
https://www.dermis.net/bilder/CD086/550px/img0048.jpg
https://www.dermis.net/bilder/CD098/550px/img0071.jpg
https://www.dermis.net/bilder/CD089/550px/img0008.jpg
https://www.dermis.net/bilder/CD086/550px/img0077.jpg
https://www.dermis.net/bilder/CD093/550px/img0094.jpg
https://www.dermis.net/bilder/CD093/550px/img0095.jpg
https://www.dermis.net/bilder/CD089/550px/img0004.jpg
https://www.dermis.net/bilder/CD097/550px/img0010.jpg
https://www.dermis.net/bilder/CD088/550px/img0099.jpg
https://www.dermis.net/bilder/CD099/550px/img0094.jpg
https://www.dermis.net/bilder/CD099/550px/img0095.jpg
https://www.dermis.net/bilder/CD099/550px/img0096.jpg
https://www.dermis.net/bilder/CD088/550px/img0091.jpg
https://www.dermis.net/bilder/CD088/550px/img0092.jpg
https://www.dermis.net/bilder/CD097/550px/img0008.jpg
https://www.dermis.net/bilder/CD097/550px/img0009.jpg

https://www.dermis.net/bilder/CD088/550px/img0100.jpg
https://www.dermis.net/bilder/CD098/550px/img0050.jpg
https://www.dermis.net/bilder/CD098/550px/img0051.jpg
https://www.dermis.net/bilder/CD098/550px/img0049.jpg
https://www.dermis.net/bilder/CD086/550px/img0070.jpg
https://www.dermis.net/bilder/CD086/550px/img0071.jpg
https://www.dermis.net/bilder/CD086/550px/img0072.jpg
https://www.dermis.net/bilder/CD086/550px/img0073.jpg
https://www.dermis.net/bilder/CD106/550px/img0042.jpg
https://www.dermis.net/bilder/CD106/550px/img0044.jpg
https://www.dermis.net/bilder/CD098/550px/img0029.jpg
https://www.dermis.net/bilder/CD098/550px/img0030.jpg
https://www.dermis.net/bilder/CD055/550px/img0035.jpg
https://www.dermis.net/bilder/CD093/550px/img0029.jpg
https://www.dermis.net/bilder/CD088/550px/img0053.jpg
https://www.dermis.net/bilder/CD087/550px/img0041.jpg
https://www.dermis.net/bilder/CD087/550px/img0043.jpg
https://www.dermis.net/bilder/CD085/550px/img0059.jpg
https://www.dermis.net/bilder/CD085/550px/img0060.jpg
https://www.dermis.net/bilder/CD103/550px/img0025.jpg
https://www.dermis.net/bilder/CD103/550px/img0045.jpg
https://www.dermis.net/bilder/CD103/550px/img0065.jpg
https://www.dermis.net/bilder/CD106/550px/img0054.jpg
https://www.dermis.net/bilder/CD106/550px/img0055.jpg
https://www.dermis.net/bilder/CD106/550px/img0056.jpg
https://www.dermis.net/bilder/CD106/550px/img0057.jpg
https://www.dermis.net/bilder/CD106/550px/img0061.jpg
https://www.dermis.net/bilder/CD106/550px/img0066.jpg
https://www.dermis.net/bilder/CD106/550px/img0067.jpg
https://www.dermis.net/bilder/CD096/550px/img0068.jpg
https://www.dermis.net/bilder/CD087/550px/img0018.jpg
https://www.dermis.net/bilder/CD087/550px/img0020.jpg
https://www.dermis.net/bilder/CD087/550px/img0021.jpg
https://www.dermis.net/bilder/CD087/550px/img0023.jpg
https://www.dermis.net/bilder/CD106/550px/img0076.jpg
https://www.dermis.net/bilder/CD106/550px/img0077.jpg
https://www.dermis.net/bilder/CD106/550px/img0078.jpg
https://www.dermis.net/bilder/CD106/550px/img0079.jpg
https://www.dermis.net/bilder/CD106/550px/img0080.jpg
https://www.dermis.net/bilder/CD107/550px/img0020.jpg
https://www.dermis.net/bilder/CD086/550px/img0031.jpg
https://www.dermis.net/bilder/CD088/550px/img0030.jpg
https://www.dermis.net/bilder/CD086/550px/img0011.jpg
https://www.dermis.net/bilder/CD099/550px/img0071.jpg
https://www.dermis.net/bilder/CD099/550px/img0072.jpg
https://www.dermis.net/bilder/CD106/550px/img0025.jpg
https://www.dermis.net/bilder/CD106/550px/img0026.jpg
https://www.dermis.net/bilder/CD106/550px/img0027.jpg

https://www.dermis.net/bilder/CD110/550px/img0082.jpg
https://www.dermis.net/bilder/CD087/550px/img0032.jpg
https://www.dermis.net/bilder/CD087/550px/img0033.jpg
https://www.dermis.net/bilder/CD087/550px/img0034.jpg
https://www.dermis.net/bilder/CD087/550px/img0035.jpg
https://www.dermis.net/bilder/CD086/550px/img0026.jpg
https://www.dermis.net/bilder/CD086/550px/img0028.jpg
https://www.dermis.net/bilder/CD088/550px/img0088.jpg
https://www.dermis.net/bilder/CD088/550px/img0090.jpg
https://www.dermis.net/bilder/CD086/550px/img0075.jpg
https://www.dermis.net/bilder/CD088/550px/img0005.jpg
https://www.dermis.net/bilder/CD086/550px/img0008.jpg
https://www.dermis.net/bilder/CD086/550px/img0009.jpg
https://www.dermis.net/bilder/CD086/550px/img0010.jpg
https://www.dermis.net/bilder/CD089/550px/img0010.jpg
https://www.dermis.net/bilder/CD089/550px/img0011.jpg
https://www.dermis.net/bilder/CD089/550px/img0012.jpg
https://www.dermis.net/bilder/CD106/550px/img0053.jpg
https://www.dermis.net/bilder/CD106/550px/img0028.jpg
https://www.dermis.net/bilder/CD085/550px/img0036.jpg
https://www.dermis.net/bilder/CD086/550px/img0098.jpg
https://www.dermis.net/bilder/CD087/550px/img0055.jpg
https://www.dermis.net/bilder/CD107/550px/img0031.jpg
https://www.dermis.net/bilder/CD096/550px/img0070.jpg
https://www.dermis.net/bilder/CD093/550px/img0023.jpg
https://www.dermis.net/bilder/CD107/550px/img0027.jpg
https://www.dermis.net/bilder/CD086/550px/img0066.jpg
https://www.dermis.net/bilder/CD086/550px/img0067.jpg
https://www.dermis.net/bilder/CD086/550px/img0068.jpg
https://www.dermis.net/bilder/CD086/550px/img0069.jpg
https://www.dermis.net/bilder/CD088/550px/img0015.jpg
https://www.dermis.net/bilder/CD088/550px/img0016.jpg
https://www.dermis.net/bilder/CD088/550px/img0017.jpg
https://www.dermis.net/bilder/CD088/550px/img0018.jpg
https://www.dermis.net/bilder/CD087/550px/img0006.jpg
https://www.dermis.net/bilder/CD098/550px/img0001.jpg
https://www.dermis.net/bilder/CD091/550px/img0060.jpg
https://www.dermis.net/bilder/CD091/550px/img0061.jpg
https://www.dermis.net/bilder/CD093/550px/img0034.jpg
https://www.dermis.net/bilder/CD093/550px/img0035.jpg
https://www.dermis.net/bilder/CD093/550px/img0036.jpg
https://www.dermis.net/bilder/CD093/550px/img0037.jpg
https://www.dermis.net/bilder/CD093/550px/img0039.jpg
https://www.dermis.net/bilder/CD097/550px/img0048.jpg
https://www.dermis.net/bilder/CD098/550px/img0065.jpg
https://www.dermis.net/bilder/CD089/550px/img0002.jpg
https://www.dermis.net/bilder/CD089/550px/img0003.jpg
https://www.dermis.net/bilder/CD097/550px/img0067.jpg

https://www.dermis.net/bilder/CD107/550px/img0017.jpg
https://www.dermis.net/bilder/CD106/550px/img0092.jpg
https://www.dermis.net/bilder/CD106/550px/img0093.jpg
https://www.dermis.net/bilder/CD089/550px/img0005.jpg
https://www.dermis.net/bilder/CD089/550px/img0006.jpg
https://www.dermis.net/bilder/CD089/550px/img0007.jpg
https://www.dermis.net/bilder/CD086/550px/img0095.jpg
https://www.dermis.net/bilder/CD093/550px/img0069.jpg
https://www.dermis.net/bilder/CD093/550px/img0070.jpg
https://www.dermis.net/bilder/CD093/550px/img0055.jpg
https://www.dermis.net/bilder/CD093/550px/img0061.jpg
https://www.dermis.net/bilder/CD093/550px/img0062.jpg
https://www.dermis.net/bilder/CD106/550px/img0004.jpg
https://www.dermis.net/bilder/CD106/550px/img0005.jpg
https://www.dermis.net/bilder/CD106/550px/img0018.jpg
https://www.dermis.net/bilder/CD106/550px/img0019.jpg
https://www.dermis.net/bilder/CD106/550px/img0002.jpg
https://www.dermis.net/bilder/CD106/550px/img0006.jpg
https://www.dermis.net/bilder/CD106/550px/img0021.jpg
https://www.dermis.net/bilder/CD087/550px/img0024.jpg
https://www.dermis.net/bilder/CD093/550px/img0083.jpg
https://www.dermis.net/bilder/CD093/550px/img0084.jpg
https://www.dermis.net/bilder/CD093/550px/img0085.jpg
https://www.dermis.net/bilder/CD093/550px/img0086.jpg
https://www.dermis.net/bilder/CD087/550px/img0031.jpg
https://www.dermis.net/bilder/CD086/550px/img0024.jpg
https://www.dermis.net/bilder/CD087/550px/img0004.jpg
https://www.dermis.net/bilder/CD087/550px/img0005.jpg
https://www.dermis.net/bilder/CD099/550px/img0079.jpg
https://www.dermis.net/bilder/CD087/550px/img0047.jpg
https://www.dermis.net/bilder/CD107/550px/img0044.jpg
https://www.dermis.net/bilder/CD107/550px/img0045.jpg
https://www.dermis.net/bilder/CD107/550px/img0048.jpg
https://www.dermis.net/bilder/CD107/550px/img0050.jpg
https://www.dermis.net/bilder/CD107/550px/img0051.jpg
https://www.dermis.net/bilder/CD107/550px/img0053.jpg
https://www.dermis.net/bilder/CD093/550px/img0028.jpg
https://www.dermis.net/bilder/CD086/550px/img0063.jpg
https://www.dermis.net/bilder/CD097/550px/img0059.jpg
https://www.dermis.net/bilder/CD097/550px/img0060.jpg
https://www.dermis.net/bilder/CD088/550px/img0054.jpg
https://www.dermis.net/bilder/CD098/550px/img0007.jpg
https://www.dermis.net/bilder/CD088/550px/img0052.jpg
https://www.dermis.net/bilder/CD106/550px/img0034.jpg
https://www.dermis.net/bilder/CD106/550px/img0035.jpg
https://www.dermis.net/bilder/CD106/550px/img0037.jpg
https://www.dermis.net/bilder/CD106/550px/img0038.jpg
https://www.dermis.net/bilder/CD106/550px/img0040.jpg

https://www.dermis.net/bilder/CD106/550px/img0041.jpg
https://www.dermis.net/bilder/CD106/550px/img0046.jpg
https://www.dermis.net/bilder/CD106/550px/img0048.jpg
https://www.dermis.net/bilder/CD086/550px/img0058.jpg
https://www.dermis.net/bilder/CD086/550px/img0059.jpg
https://www.dermis.net/bilder/CD056/550px/img0017.jpg
https://www.dermis.net/bilder/CD057/550px/img0070.jpg
https://www.dermis.net/bilder/CD057/550px/img0071.jpg
https://www.dermis.net/bilder/CD079/550px/img0015.jpg
https://www.dermis.net/bilder/CD079/550px/img0016.jpg
https://www.dermis.net/bilder/CD079/550px/img0017.jpg
https://www.dermis.net/bilder/CD150/550px/img0070.jpg
https://www.dermis.net/bilder/CD150/550px/img0072.jpg
https://www.dermis.net/bilder/CD150/550px/img0073.jpg
https://www.dermis.net/bilder/CD076/550px/img0066.jpg
https://www.dermis.net/bilder/CD079/550px/img0041.jpg
https://www.dermis.net/bilder/CD079/550px/img0042.jpg
https://www.dermis.net/bilder/CD079/550px/img0043.jpg
https://www.dermis.net/bilder/CD079/550px/img0045.jpg
https://www.dermis.net/bilder/CD077/550px/img0029.jpg
https://www.dermis.net/bilder/CD077/550px/img0030.jpg
https://www.dermis.net/bilder/CD077/550px/img0031.jpg
https://www.dermis.net/bilder/CD077/550px/img0009.jpg
https://www.dermis.net/bilder/CD077/550px/img0010.jpg
https://www.dermis.net/bilder/CD077/550px/img0014.jpg
https://www.dermis.net/bilder/CD078/550px/img0079.jpg
https://www.dermis.net/bilder/CD078/550px/img0081.jpg
https://www.dermis.net/bilder/CD152/550px/img0057.jpg
https://www.dermis.net/bilder/CD152/550px/img0060.jpg
https://www.dermis.net/bilder/CD078/550px/img0047.jpg
https://www.dermis.net/bilder/CD078/550px/img0048.jpg
https://www.dermis.net/bilder/CD078/550px/img0049.jpg
https://www.dermis.net/bilder/CD150/550px/img0018.jpg
https://www.dermis.net/bilder/CD150/550px/img0019.jpg
https://www.dermis.net/bilder/CD150/550px/img0021.jpg
https://www.dermis.net/bilder/CD055/550px/img0038.jpg
https://www.dermis.net/bilder/CD150/550px/img0063.jpg
https://www.dermis.net/bilder/CD152/550px/img0070.jpg
https://www.dermis.net/bilder/CD153/550px/img0005.jpg
https://www.dermis.net/bilder/CD154/550px/img0099.jpg
https://www.dermis.net/bilder/CD184/550px/img0003.jpg
https://www.dermis.net/bilder/CD076/550px/img0076.jpg
https://www.dermis.net/bilder/CD078/550px/img0061.jpg
https://www.dermis.net/bilder/CD078/550px/img0092.jpg
https://www.dermis.net/bilder/CD055/550px/img0012.jpg
https://www.dermis.net/bilder/CD077/550px/img0098.jpg
https://www.dermis.net/bilder/CD077/550px/img0100.jpg
https://www.dermis.net/bilder/CD078/550px/img0068.jpg

https://www.dermis.net/bilder/CD052/550px/img0096.jpg
https://www.dermis.net/bilder/CD153/550px/img0063.jpg
https://www.dermis.net/bilder/CD153/550px/img0064.jpg
https://www.dermis.net/bilder/CD153/550px/img0065.jpg
https://www.dermis.net/bilder/CD153/550px/img0066.jpg
https://www.dermis.net/bilder/CD033/550px/img0036.jpg
https://www.dermis.net/bilder/CD003/550px/img0063.jpg
https://www.dermis.net/bilder/CD003/550px/img0070.jpg
https://www.dermis.net/bilder/CD003/550px/img0071.jpg
https://www.dermis.net/bilder/CD003/550px/img0072.jpg
https://www.dermis.net/bilder/CD017/550px/img0038.jpg
https://www.dermis.net/bilder/CD077/550px/img0058.jpg
https://www.dermis.net/bilder/CD077/550px/img0059.jpg
https://www.dermis.net/bilder/CD079/550px/img0008.jpg
https://www.dermis.net/bilder/CD079/550px/img0009.jpg
https://www.dermis.net/bilder/CD079/550px/img0010.jpg
https://www.dermis.net/bilder/CD151/550px/img0086.jpg
https://www.dermis.net/bilder/CD151/550px/img0088.jpg
https://www.dermis.net/bilder/CD152/550px/img0076.jpg
https://www.dermis.net/bilder/CD152/550px/img0077.jpg
https://www.dermis.net/bilder/CD152/550px/img0078.jpg
https://www.dermis.net/bilder/CD152/550px/img0080.jpg
https://www.dermis.net/bilder/CD152/550px/img0081.jpg
https://www.dermis.net/bilder/CD079/550px/img0064.jpg
https://www.dermis.net/bilder/CD029/550px/img0010.jpg
https://www.dermis.net/bilder/CD152/550px/img0001.jpg
https://www.dermis.net/bilder/CD056/550px/img0091.jpg
https://www.dermis.net/bilder/CD085/550px/img0061.jpg
https://www.dermis.net/bilder/CD085/550px/img0062.jpg
https://www.dermis.net/bilder/CD085/550px/img0063.jpg
https://www.dermis.net/bilder/CD055/550px/img0055.jpg
https://www.dermis.net/bilder/CD001/550px/img0015.jpg
https://www.dermis.net/bilder/CD001/550px/img0063.jpg
https://www.dermis.net/bilder/CD001/550px/img0087.jpg
https://www.dermis.net/bilder/CD001/550px/img0098.jpg
https://www.dermis.net/bilder/CD076/550px/img0107.jpg
https://www.dermis.net/bilder/CD076/550px/img0108.jpg
https://www.dermis.net/bilder/CD153/550px/img0007.jpg
https://www.dermis.net/bilder/CD153/550px/img0008.jpg
https://www.dermis.net/bilder/CD153/550px/img0009.jpg
https://www.dermis.net/bilder/CD153/550px/img0010.jpg
https://www.dermis.net/bilder/CD153/550px/img0019.jpg
https://www.dermis.net/bilder/CD153/550px/img0020.jpg
https://www.dermis.net/bilder/CD153/550px/img0021.jpg
https://www.dermis.net/bilder/CD078/550px/img0078.jpg
https://www.dermis.net/bilder/CD079/550px/img0046.jpg
https://www.dermis.net/bilder/CD079/550px/img0095.jpg
https://www.dermis.net/bilder/CD151/550px/img0048.jpg

https://www.dermis.net/bilder/CD035/550px/img0028.jpg
https://www.dermis.net/bilder/CD076/550px/img0058.jpg
https://www.dermis.net/bilder/CD078/550px/img0026.jpg
https://www.dermis.net/bilder/CD079/550px/img0047.jpg
https://www.dermis.net/bilder/CD153/550px/img0049.jpg
https://www.dermis.net/bilder/CD061/550px/img0004.jpg
https://www.dermis.net/bilder/CD061/550px/img0005.jpg
https://www.dermis.net/bilder/CD178/550px/img0010.jpg
https://www.dermis.net/bilder/CD189/550px/img0112.jpg
https://www.dermis.net/bilder/CD187/550px/img0022.jpg
https://www.dermis.net/bilder/CD191/550px/img0083.jpg
https://www.dermis.net/bilder/CD191/550px/img0084.jpg
https://www.dermis.net/bilder/CD212/550px/img0083.jpg
https://www.dermis.net/bilder/CD033/550px/img0014.jpg
https://www.dermis.net/bilder/CD152/550px/img0010.jpg
https://www.dermis.net/bilder/CD152/550px/img0012.jpg
https://www.dermis.net/bilder/CD152/550px/img0013.jpg
https://www.dermis.net/bilder/CD152/550px/img0014.jpg
https://www.dermis.net/bilder/CD154/550px/img0020.jpg
https://www.dermis.net/bilder/CD079/550px/img0048.jpg
https://www.dermis.net/bilder/CD151/550px/img0103.jpg
https://www.dermis.net/bilder/CD077/550px/img0003.jpg
https://www.dermis.net/bilder/CD077/550px/img0004.jpg
https://www.dermis.net/bilder/CD150/550px/img0013.jpg
https://www.dermis.net/bilder/CD058/550px/img0095.jpg
https://www.dermis.net/bilder/CD077/550px/img0001.jpg
https://www.dermis.net/bilder/CD153/550px/img0053.jpg
https://www.dermis.net/bilder/CD078/550px/img0025.jpg
https://www.dermis.net/bilder/CD079/550px/img0011.jpg
https://www.dermis.net/bilder/CD079/550px/img0012.jpg
https://www.dermis.net/bilder/CD008/550px/img0047.jpg
https://www.dermis.net/bilder/CD078/550px/img0072.jpg
https://www.dermis.net/bilder/CD078/550px/img0073.jpg
https://www.dermis.net/bilder/CD078/550px/img0074.jpg
https://www.dermis.net/bilder/CD078/550px/img0075.jpg
https://www.dermis.net/bilder/CD078/550px/img0076.jpg
https://www.dermis.net/bilder/CD150/550px/img0038.jpg
https://www.dermis.net/bilder/CD150/550px/img0039.jpg
https://www.dermis.net/bilder/CD150/550px/img0040.jpg
https://www.dermis.net/bilder/CD150/550px/img0041.jpg
https://www.dermis.net/bilder/CD151/550px/img0050.jpg
https://www.dermis.net/bilder/CD151/550px/img0051.jpg
https://www.dermis.net/bilder/CD151/550px/img0052.jpg
https://www.dermis.net/bilder/CD152/550px/img0071.jpg
https://www.dermis.net/bilder/CD152/550px/img0074.jpg
https://www.dermis.net/bilder/CD152/550px/img0075.jpg
https://www.dermis.net/bilder/CD056/550px/img0028.jpg
https://www.dermis.net/bilder/CD056/550px/img0029.jpg

https://www.dermis.net/bilder/CD056/550px/img0065.jpg
https://www.dermis.net/bilder/CD154/550px/img0055.jpg
https://www.dermis.net/bilder/CD015/550px/img0019.jpg
https://www.dermis.net/bilder/CD050/550px/img0040.jpg
https://www.dermis.net/bilder/CD154/550px/img0100.jpg
https://www.dermis.net/bilder/CD150/550px/img0074.jpg
https://www.dermis.net/bilder/CD150/550px/img0075.jpg
https://www.dermis.net/bilder/CD002/550px/img0010.jpg
https://www.dermis.net/bilder/CD076/550px/img0075.jpg
https://www.dermis.net/bilder/CD036/550px/img0016.jpg
https://www.dermis.net/bilder/CD191/550px/img0072.jpg
https://www.dermis.net/bilder/CD191/550px/img0073.jpg
https://www.dermis.net/bilder/CD154/550px/img0062.jpg
https://www.dermis.net/bilder/CD154/550px/img0063.jpg
https://www.dermis.net/bilder/CD154/550px/img0064.jpg
https://www.dermis.net/bilder/CD154/550px/img0065.jpg
https://www.dermis.net/bilder/CD058/550px/img0094.jpg
https://www.dermis.net/bilder/CD001/550px/img0070.jpg
https://www.dermis.net/bilder/CD153/550px/img0077.jpg
https://www.dermis.net/bilder/CD153/550px/img0078.jpg
https://www.dermis.net/bilder/CD085/550px/img0076.jpg
https://www.dermis.net/bilder/CD085/550px/img0077.jpg
https://www.dermis.net/bilder/CD088/550px/img0007.jpg
https://www.dermis.net/bilder/CD106/550px/img0051.jpg
https://www.dermis.net/bilder/CD106/550px/img0052.jpg
https://www.dermis.net/bilder/CD110/550px/img0025.jpg
https://www.dermis.net/bilder/CD079/550px/img0070.jpg
https://www.dermis.net/bilder/CD076/550px/img0031.jpg
https://www.dermis.net/bilder/CD076/550px/img0032.jpg
https://www.dermis.net/bilder/CD076/550px/img0044.jpg
https://www.dermis.net/bilder/CD150/550px/img0101.jpg
https://www.dermis.net/bilder/CD192/550px/img0029.jpg
https://www.dermis.net/bilder/CD060/550px/img0008.jpg
https://www.dermis.net/bilder/CD060/550px/img0009.jpg
https://www.dermis.net/bilder/CD061/550px/img0006.jpg
https://www.dermis.net/bilder/CD041/550px/img0094.jpg
https://www.dermis.net/bilder/CD084/550px/img0095.jpg
https://www.dermis.net/bilder/CD005/550px/img0059.jpg
https://www.dermis.net/bilder/CD185/550px/img0019.jpg
https://www.dermis.net/bilder/CD185/550px/img0020.jpg
https://www.dermis.net/bilder/CD033/550px/img0033.jpg
https://www.dermis.net/bilder/CD033/550px/img0034.jpg
https://www.dermis.net/bilder/CD008/550px/img0001.jpg
https://www.dermis.net/bilder/CD078/550px/img0062.jpg
https://www.dermis.net/bilder/CD078/550px/img0063.jpg
https://www.dermis.net/bilder/CD152/550px/img0100.jpg
https://www.dermis.net/bilder/CD042/550px/img0009.jpg
https://www.dermis.net/bilder/CD040/550px/img0062.jpg

https://www.dermis.net/bilder/CD040/550px/img0064.jpg
https://www.dermis.net/bilder/CD079/550px/img0089.jpg
https://www.dermis.net/bilder/CD079/550px/img0090.jpg
https://www.dermis.net/bilder/CD004/550px/img0055.jpg
https://www.dermis.net/bilder/CD153/550px/img0030.jpg
https://www.dermis.net/bilder/CD056/550px/img0035.jpg
https://www.dermis.net/bilder/CD077/550px/img0041.jpg
https://www.dermis.net/bilder/CD077/550px/img0042.jpg
https://www.dermis.net/bilder/CD077/550px/img0044.jpg
https://www.dermis.net/bilder/CD151/550px/img0026.jpg
https://www.dermis.net/bilder/CD151/550px/img0027.jpg
https://www.dermis.net/bilder/CD153/550px/img0041.jpg
https://www.dermis.net/bilder/CD153/550px/img0042.jpg
https://www.dermis.net/bilder/CD153/550px/img0043.jpg
https://www.dermis.net/bilder/CD153/550px/img0046.jpg
https://www.dermis.net/bilder/CD032/550px/img0003.jpg
https://www.dermis.net/bilder/CD032/550px/img0004.jpg
https://www.dermis.net/bilder/CD078/550px/img0003.jpg
https://www.dermis.net/bilder/CD078/550px/img0058.jpg
https://www.dermis.net/bilder/CD078/550px/img0059.jpg
https://www.dermis.net/bilder/CD079/550px/img0093.jpg
https://www.dermis.net/bilder/CD150/550px/img0009.jpg
https://www.dermis.net/bilder/CD056/550px/img0002.jpg
https://www.dermis.net/bilder/CD013/550px/img0028.jpg
https://www.dermis.net/bilder/CD154/550px/img0012.jpg
https://www.dermis.net/bilder/CD154/550px/img0013.jpg
https://www.dermis.net/bilder/CD035/550px/img0073.jpg
https://www.dermis.net/bilder/CD061/550px/img0001.jpg
https://www.dermis.net/bilder/CD064/550px/img0001.jpg
https://www.dermis.net/bilder/CD064/550px/img0025.jpg
https://www.dermis.net/bilder/CD064/550px/img0026.jpg
https://www.dermis.net/bilder/CD064/550px/img0027.jpg
https://www.dermis.net/bilder/CD064/550px/img0028.jpg
https://www.dermis.net/bilder/CD064/550px/img0029.jpg
https://www.dermis.net/bilder/CD064/550px/img0030.jpg
https://www.dermis.net/bilder/CD064/550px/img0031.jpg
https://www.dermis.net/bilder/CD064/550px/img0032.jpg
https://www.dermis.net/bilder/CD064/550px/img0033.jpg
https://www.dermis.net/bilder/CD064/550px/img0034.jpg
https://www.dermis.net/bilder/CD064/550px/img0035.jpg
https://www.dermis.net/bilder/CD064/550px/img0036.jpg
https://www.dermis.net/bilder/CD064/550px/img0037.jpg
https://www.dermis.net/bilder/CD064/550px/img0038.jpg
https://www.dermis.net/bilder/CD064/550px/img0039.jpg
https://www.dermis.net/bilder/CD064/550px/img0040.jpg
https://www.dermis.net/bilder/CD064/550px/img0041.jpg
https://www.dermis.net/bilder/CD064/550px/img0042.jpg
https://www.dermis.net/bilder/CD064/550px/img0043.jpg

https://www.dermis.net/bilder/CD064/550px/img0044.jpg
https://www.dermis.net/bilder/CD064/550px/img0046.jpg
https://www.dermis.net/bilder/CD077/550px/img0025.jpg
https://www.dermis.net/bilder/CD077/550px/img0026.jpg
https://www.dermis.net/bilder/CD077/550px/img0027.jpg
https://www.dermis.net/bilder/CD079/550px/img0059.jpg
https://www.dermis.net/bilder/CD040/550px/img0053.jpg
https://www.dermis.net/bilder/CD040/550px/img0060.jpg
https://www.dermis.net/bilder/CD151/550px/img0098.jpg
https://www.dermis.net/bilder/CD151/550px/img0099.jpg
https://www.dermis.net/bilder/CD154/550px/img0059.jpg
https://www.dermis.net/bilder/CD154/550px/img0060.jpg
https://www.dermis.net/bilder/CD154/550px/img0061.jpg
https://www.dermis.net/bilder/CD055/550px/img0017.jpg
https://www.dermis.net/bilder/CD016/550px/img0063.jpg
https://www.dermis.net/bilder/CD079/550px/img0082.jpg
https://www.dermis.net/bilder/CD021/550px/img0012.jpg
https://www.dermis.net/bilder/CD021/550px/img0015.jpg
https://www.dermis.net/bilder/CD078/550px/img0001.jpg
https://www.dermis.net/bilder/CD076/550px/img0046.jpg
https://www.dermis.net/bilder/CD076/550px/img0047.jpg
https://www.dermis.net/bilder/CD078/550px/img0053.jpg
https://www.dermis.net/bilder/CD078/550px/img0055.jpg
https://www.dermis.net/bilder/CD078/550px/img0056.jpg
https://www.dermis.net/bilder/CD150/550px/img0025.jpg
https://www.dermis.net/bilder/CD150/550px/img0026.jpg
https://www.dermis.net/bilder/CD018/550px/img0027.jpg
https://www.dermis.net/bilder/CD018/550px/img0028.jpg
https://www.dermis.net/bilder/CD153/550px/img0038.jpg
https://www.dermis.net/bilder/CD056/550px/img0046.jpg
https://www.dermis.net/bilder/CD055/550px/img0008.jpg
https://www.dermis.net/bilder/CD079/550px/img0087.jpg
https://www.dermis.net/bilder/CD085/550px/img0013.jpg
https://www.dermis.net/bilder/CD152/550px/img0017.jpg
https://www.dermis.net/bilder/CD153/550px/img0025.jpg
https://www.dermis.net/bilder/CD153/550px/img0026.jpg
https://www.dermis.net/bilder/CD154/550px/img0016.jpg
https://www.dermis.net/bilder/CD085/550px/img0021.jpg
https://www.dermis.net/bilder/CD079/550px/img0065.jpg
https://www.dermis.net/bilder/CD079/550px/img0066.jpg
https://www.dermis.net/bilder/CD079/550px/img0058.jpg
https://www.dermis.net/bilder/CD151/550px/img0062.jpg
https://www.dermis.net/bilder/CD151/550px/img0105.jpg
https://www.dermis.net/bilder/CD153/550px/img0061.jpg
https://www.dermis.net/bilder/CD151/550px/img0025.jpg
https://www.dermis.net/bilder/CD025/550px/img0006.jpg
https://www.dermis.net/bilder/CD150/550px/img0017.jpg
https://www.dermis.net/bilder/CD151/550px/img0090.jpg

https://www.dermis.net/bilder/CD079/550px/img0029.jpg
https://www.dermis.net/bilder/CD079/550px/img0030.jpg
https://www.dermis.net/bilder/CD079/550px/img0031.jpg
https://www.dermis.net/bilder/CD079/550px/img0032.jpg
https://www.dermis.net/bilder/CD012/550px/img0025.jpg
https://www.dermis.net/bilder/CD054/550px/img0028.jpg
https://www.dermis.net/bilder/CD073/550px/img0025.jpg
https://www.dermis.net/bilder/CD073/550px/img0026.jpg
https://www.dermis.net/bilder/CD073/550px/img0027.jpg
https://www.dermis.net/bilder/CD073/550px/img0028.jpg
https://www.dermis.net/bilder/CD073/550px/img0029.jpg
https://www.dermis.net/bilder/CD154/550px/img0015.jpg
https://www.dermis.net/bilder/CD008/550px/img0081.jpg
https://www.dermis.net/bilder/CD197/550px/img0044.jpg
https://www.dermis.net/bilder/CD001/550px/img0075.jpg
https://www.dermis.net/bilder/CD001/550px/img0076.jpg
https://www.dermis.net/bilder/CD151/550px/img0044.jpg
https://www.dermis.net/bilder/CD151/550px/img0047.jpg
https://www.dermis.net/bilder/CD154/550px/img0023.jpg
https://www.dermis.net/bilder/CD154/550px/img0030.jpg
https://www.dermis.net/bilder/CD039/550px/img0011.jpg
https://www.dermis.net/bilder/CD039/550px/img0012.jpg
https://www.dermis.net/bilder/CD078/550px/img0035.jpg
https://www.dermis.net/bilder/CD078/550px/img0036.jpg
https://www.dermis.net/bilder/CD150/550px/img0024.jpg
https://www.dermis.net/bilder/CD178/550px/img0060.jpg
https://www.dermis.net/bilder/CD006/550px/img0032.jpg
https://www.dermis.net/bilder/CD059/550px/img0081.jpg
https://www.dermis.net/bilder/CD154/550px/img0019.jpg
https://www.dermis.net/bilder/CD154/550px/img0081.jpg
https://www.dermis.net/bilder/CD055/550px/img0053.jpg
https://www.dermis.net/bilder/CD013/550px/img0015.jpg
https://www.dermis.net/bilder/CD011/550px/img0065.jpg
https://www.dermis.net/bilder/CD011/550px/img0066.jpg
https://www.dermis.net/bilder/CD197/550px/img0043.jpg
https://www.dermis.net/bilder/CD055/550px/img0051.jpg
https://www.dermis.net/bilder/CD151/550px/img0112.jpg
https://www.dermis.net/bilder/CD151/550px/img0113.jpg
https://www.dermis.net/bilder/CD151/550px/img0114.jpg
https://www.dermis.net/bilder/CD151/550px/img0115.jpg
https://www.dermis.net/bilder/CD151/550px/img0116.jpg
https://www.dermis.net/bilder/CD192/550px/img0093.jpg
https://www.dermis.net/bilder/CD192/550px/img0092.jpg
https://www.dermis.net/bilder/CD055/550px/img0070.jpg
https://www.dermis.net/bilder/CD076/550px/img0090.jpg
https://www.dermis.net/bilder/CD077/550px/img0090.jpg
https://www.dermis.net/bilder/CD151/550px/img0017.jpg
https://www.dermis.net/bilder/CD154/550px/img0098.jpg

https://www.dermis.net/bilder/CD150/550px/img0086.jpg
https://www.dermis.net/bilder/CD150/550px/img0091.jpg
https://www.dermis.net/bilder/CD154/550px/img0097.jpg
https://www.dermis.net/bilder/CD150/550px/img0011.jpg
https://www.dermis.net/bilder/CD150/550px/img0087.jpg
https://www.dermis.net/bilder/CD151/550px/img0011.jpg
https://www.dermis.net/bilder/CD151/550px/img0012.jpg
https://www.dermis.net/bilder/CD040/550px/img0043.jpg
https://www.dermis.net/bilder/CD077/550px/img0088.jpg
https://www.dermis.net/bilder/CD151/550px/img0015.jpg
https://www.dermis.net/bilder/CD032/550px/img0083.jpg
https://www.dermis.net/bilder/CD041/550px/img0018.jpg
https://www.dermis.net/bilder/CD041/550px/img0019.jpg
https://www.dermis.net/bilder/CD076/550px/img0110.jpg
https://www.dermis.net/bilder/CD076/550px/img0111.jpg
https://www.dermis.net/bilder/CD076/550px/img0112.jpg
https://www.dermis.net/bilder/CD150/550px/img0007.jpg
https://www.dermis.net/bilder/CD150/550px/img0008.jpg
https://www.dermis.net/bilder/CD151/550px/img0020.jpg
https://www.dermis.net/bilder/CD151/550px/img0021.jpg
https://www.dermis.net/bilder/CD151/550px/img0022.jpg
https://www.dermis.net/bilder/CD187/550px/img0059.jpg
https://www.dermis.net/bilder/CD078/550px/img0095.jpg
https://www.dermis.net/bilder/CD152/550px/img0029.jpg
https://www.dermis.net/bilder/CD056/550px/img0059.jpg
https://www.dermis.net/bilder/CD154/550px/img0003.jpg
https://www.dermis.net/bilder/CD154/550px/img0004.jpg
https://www.dermis.net/bilder/CD077/550px/img0072.jpg
https://www.dermis.net/bilder/CD012/550px/img0006.jpg
https://www.dermis.net/bilder/CD012/550px/img0018.jpg
https://www.dermis.net/bilder/CD012/550px/img0019.jpg
https://www.dermis.net/bilder/CD021/550px/img0096.jpg
https://www.dermis.net/bilder/CD192/550px/img0007.jpg
https://www.dermis.net/bilder/CD192/550px/img0008.jpg
https://www.dermis.net/bilder/CD042/550px/img0049.jpg
https://www.dermis.net/bilder/CD152/550px/img0023.jpg
https://www.dermis.net/bilder/CD079/550px/img0076.jpg
https://www.dermis.net/bilder/CD153/550px/img0070.jpg
https://www.dermis.net/bilder/CD011/550px/img0050.jpg
https://www.dermis.net/bilder/CD011/550px/img0051.jpg
https://www.dermis.net/bilder/CD011/550px/img0052.jpg
https://www.dermis.net/bilder/CD011/550px/img0053.jpg
https://www.dermis.net/bilder/CD076/550px/img0067.jpg
https://www.dermis.net/bilder/CD076/550px/img0068.jpg
https://www.dermis.net/bilder/CD076/550px/img0069.jpg
https://www.dermis.net/bilder/CD008/550px/img0084.jpg
https://www.dermis.net/bilder/CD077/550px/img0018.jpg
https://www.dermis.net/bilder/CD151/550px/img0043.jpg

https://www.dermis.net/bilder/CD085/550px/img0005.jpg
https://www.dermis.net/bilder/CD085/550px/img0006.jpg
https://www.dermis.net/bilder/CD085/550px/img0078.jpg
https://www.dermis.net/bilder/CD085/550px/img0079.jpg
https://www.dermis.net/bilder/CD154/550px/img0082.jpg
https://www.dermis.net/bilder/CD154/550px/img0083.jpg
https://www.dermis.net/bilder/CD152/550px/img0031.jpg
https://www.dermis.net/bilder/CD152/550px/img0032.jpg
https://www.dermis.net/bilder/CD077/550px/img0056.jpg
https://www.dermis.net/bilder/CD077/550px/img0048.jpg
https://www.dermis.net/bilder/CD077/550px/img0049.jpg
https://www.dermis.net/bilder/CD040/550px/img0029.jpg
https://www.dermis.net/bilder/CD151/550px/img0039.jpg
https://www.dermis.net/bilder/CD151/550px/img0041.jpg
https://www.dermis.net/bilder/CD151/550px/img0042.jpg
https://www.dermis.net/bilder/CD078/550px/img0037.jpg
https://www.dermis.net/bilder/CD078/550px/img0038.jpg
https://www.dermis.net/bilder/CD078/550px/img0039.jpg
https://www.dermis.net/bilder/CD154/550px/img0053.jpg
https://www.dermis.net/bilder/CD153/550px/img0033.jpg
https://www.dermis.net/bilder/CD153/550px/img0034.jpg
https://www.dermis.net/bilder/CD039/550px/img0037.jpg
https://www.dermis.net/bilder/CD039/550px/img0038.jpg
https://www.dermis.net/bilder/CD077/550px/img0061.jpg
https://www.dermis.net/bilder/CD014/550px/img0029.jpg
https://www.dermis.net/bilder/CD076/550px/img0045.jpg
https://www.dermis.net/bilder/CD078/550px/img0060.jpg
https://www.dermis.net/bilder/CD078/550px/img0094.jpg
https://www.dermis.net/bilder/CD150/550px/img0078.jpg
https://www.dermis.net/bilder/CD151/550px/img0078.jpg
https://www.dermis.net/bilder/CD153/550px/img0055.jpg
https://www.dermis.net/bilder/CD181/550px/img0071.jpg
https://www.dermis.net/bilder/CD181/550px/img0072.jpg
https://www.dermis.net/bilder/CD193/550px/img0134.jpg
https://www.dermis.net/bilder/CD195/550px/img0084.jpg
https://www.dermis.net/bilder/CD196/550px/img0001.jpg
https://www.dermis.net/bilder/CD197/550px/img0034.jpg
https://www.dermis.net/bilder/CD197/550px/img0035.jpg
https://www.dermis.net/bilder/CD196/550px/img0077.jpg
https://www.dermis.net/bilder/CD183/550px/img0030.jpg
https://www.dermis.net/bilder/CD014/550px/img0034.jpg
https://www.dermis.net/bilder/CD079/550px/img0068.jpg
https://www.dermis.net/bilder/CD151/550px/img0034.jpg
https://www.dermis.net/bilder/CD152/550px/img0069.jpg
https://www.dermis.net/bilder/CD046/550px/img0057.jpg
https://www.dermis.net/bilder/CD055/550px/img0005.jpg
https://www.dermis.net/bilder/CD188/550px/img0020.jpg
https://www.dermis.net/bilder/CD198/550px/img0106.jpg

https://www.dermis.net/bilder/CD078/550px/img0099.jpg
https://www.dermis.net/bilder/CD078/550px/img0101.jpg
https://www.dermis.net/bilder/CD078/550px/img0102.jpg
https://www.dermis.net/bilder/CD151/550px/img0060.jpg
https://www.dermis.net/bilder/CD151/550px/img0061.jpg
https://www.dermis.net/bilder/CD011/550px/img0018.jpg
https://www.dermis.net/bilder/CD011/550px/img0019.jpg
https://www.dermis.net/bilder/CD152/550px/img0034.jpg
https://www.dermis.net/bilder/CD152/550px/img0035.jpg
https://www.dermis.net/bilder/CD152/550px/img0036.jpg
https://www.dermis.net/bilder/CD152/550px/img0040.jpg
https://www.dermis.net/bilder/CD152/550px/img0041.jpg
https://www.dermis.net/bilder/CD152/550px/img0042.jpg
https://www.dermis.net/bilder/CD152/550px/img0016.jpg
https://www.dermis.net/bilder/CD043/550px/img0038.jpg
https://www.dermis.net/bilder/CD043/550px/img0039.jpg
https://www.dermis.net/bilder/CD037/550px/img0023.jpg
https://www.dermis.net/bilder/CD076/550px/img0001.jpg
https://www.dermis.net/bilder/CD076/550px/img0002.jpg
https://www.dermis.net/bilder/CD076/550px/img0004.jpg
https://www.dermis.net/bilder/CD076/550px/img0005.jpg
https://www.dermis.net/bilder/CD076/550px/img0006.jpg
https://www.dermis.net/bilder/CD076/550px/img0008.jpg
https://www.dermis.net/bilder/CD151/550px/img0002.jpg
https://www.dermis.net/bilder/CD151/550px/img0003.jpg
https://www.dermis.net/bilder/CD151/550px/img0004.jpg
https://www.dermis.net/bilder/CD152/550px/img0093.jpg
https://www.dermis.net/bilder/CD152/550px/img0096.jpg
https://www.dermis.net/bilder/CD154/550px/img0101.jpg
https://www.dermis.net/bilder/CD154/550px/img0102.jpg
https://www.dermis.net/bilder/CD154/550px/img0103.jpg
https://www.dermis.net/bilder/CD154/550px/img0104.jpg
https://www.dermis.net/bilder/CD002/550px/img0017.jpg
https://www.dermis.net/bilder/CD152/550px/img0098.jpg
https://www.dermis.net/bilder/CD153/550px/img0001.jpg
https://www.dermis.net/bilder/CD153/550px/img0002.jpg
https://www.dermis.net/bilder/CD153/550px/img0003.jpg
https://www.dermis.net/bilder/CD153/550px/img0048.jpg
https://www.dermis.net/bilder/CD193/550px/img0005.jpg
https://www.dermis.net/bilder/CD151/550px/img0091.jpg
https://www.dermis.net/bilder/CD076/550px/img0035.jpg
https://www.dermis.net/bilder/CD078/550px/img0084.jpg
https://www.dermis.net/bilder/CD078/550px/img0086.jpg
https://www.dermis.net/bilder/CD079/550px/img0050.jpg
https://www.dermis.net/bilder/CD055/550px/img0019.jpg
https://www.dermis.net/bilder/CD055/550px/img0020.jpg
https://www.dermis.net/bilder/CD003/550px/img0073.jpg
https://www.dermis.net/bilder/CD057/550px/img0002.jpg

https://www.dermis.net/bilder/CD055/550px/img0007.jpg
https://www.dermis.net/bilder/CD191/550px/img0022.jpg
https://www.dermis.net/bilder/CD079/550px/img0078.jpg
https://www.dermis.net/bilder/CD018/550px/img0002.jpg
https://www.dermis.net/bilder/CD152/550px/img0048.jpg
https://www.dermis.net/bilder/CD021/550px/img0070.jpg
https://www.dermis.net/bilder/CD151/550px/img0074.jpg
https://www.dermis.net/bilder/CD151/550px/img0075.jpg
https://www.dermis.net/bilder/CD154/550px/img0005.jpg
https://www.dermis.net/bilder/CD154/550px/img0008.jpg
https://www.dermis.net/bilder/CD003/550px/img0025.jpg
https://www.dermis.net/bilder/CD077/550px/img0008.jpg
https://www.dermis.net/bilder/CD003/550px/img0076.jpg
https://www.dermis.net/bilder/CD003/550px/img0077.jpg
https://www.dermis.net/bilder/CD043/550px/img0044.jpg
https://www.dermis.net/bilder/CD043/550px/img0045.jpg
https://www.dermis.net/bilder/CD043/550px/img0046.jpg
https://www.dermis.net/bilder/CD043/550px/img0047.jpg
https://www.dermis.net/bilder/CD021/550px/img0071.jpg
https://www.dermis.net/bilder/CD039/550px/img0091.jpg
https://www.dermis.net/bilder/CD039/550px/img0093.jpg
https://www.dermis.net/bilder/CD039/550px/img0094.jpg
https://www.dermis.net/bilder/CD039/550px/img0097.jpg
https://www.dermis.net/bilder/CD035/550px/img0068.jpg
https://www.dermis.net/bilder/CD154/550px/img0073.jpg
https://www.dermis.net/bilder/CD057/550px/img0013.jpg
https://www.dermis.net/bilder/CD077/550px/img0054.jpg
https://www.dermis.net/bilder/CD077/550px/img0062.jpg
https://www.dermis.net/bilder/CD153/550px/img0051.jpg
https://www.dermis.net/bilder/CD152/550px/img0085.jpg
https://www.dermis.net/bilder/CD152/550px/img0086.jpg
https://www.dermis.net/bilder/CD191/550px/img0065.jpg
https://www.dermis.net/bilder/CD106/550px/img0008.jpg
https://www.dermis.net/bilder/CD002/550px/img0098.jpg
https://www.dermis.net/bilder/CD002/550px/img0075.jpg
https://www.dermis.net/bilder/CD021/550px/img0008.jpg
https://www.dermis.net/bilder/CD021/550px/img0043.jpg
https://www.dermis.net/bilder/CD002/550px/img0073.jpg
https://www.dermis.net/bilder/CD013/550px/img0049.jpg
https://www.dermis.net/bilder/CD150/550px/img0106.jpg
https://www.dermis.net/bilder/CD150/550px/img0107.jpg
https://www.dermis.net/bilder/CD002/550px/img0099.jpg
https://www.dermis.net/bilder/CD021/550px/img0002.jpg
https://www.dermis.net/bilder/CD150/550px/img0001.jpg
https://www.dermis.net/bilder/CD150/550px/img0002.jpg
https://www.dermis.net/bilder/CD150/550px/img0044.jpg
https://www.dermis.net/bilder/CD150/550px/img0045.jpg
https://www.dermis.net/bilder/CD154/550px/img0056.jpg

https://www.dermis.net/bilder/CD154/550px/img0057.jpg
https://www.dermis.net/bilder/CD154/550px/img0058.jpg
https://www.dermis.net/bilder/CD012/550px/img0046.jpg
https://www.dermis.net/bilder/CD012/550px/img0057.jpg
https://www.dermis.net/bilder/CD012/550px/img0058.jpg
https://www.dermis.net/bilder/CD012/550px/img0059.jpg
https://www.dermis.net/bilder/CD012/550px/img0060.jpg
https://www.dermis.net/bilder/CD076/550px/img0049.jpg
https://www.dermis.net/bilder/CD076/550px/img0050.jpg
https://www.dermis.net/bilder/CD079/550px/img0084.jpg
https://www.dermis.net/bilder/CD077/550px/img0052.jpg
https://www.dermis.net/bilder/CD061/550px/img0002.jpg
https://www.dermis.net/bilder/CD061/550px/img0003.jpg
https://www.dermis.net/bilder/CD067/550px/img0017.jpg
https://www.dermis.net/bilder/CD191/550px/img0108.jpg
https://www.dermis.net/bilder/CD191/550px/img0111.jpg
https://www.dermis.net/bilder/CD191/550px/img0112.jpg
https://www.dermis.net/bilder/CD151/550px/img0106.jpg
https://www.dermis.net/bilder/CD151/550px/img0107.jpg
https://www.dermis.net/bilder/CD151/550px/img0108.jpg
https://www.dermis.net/bilder/CD151/550px/img0110.jpg
https://www.dermis.net/bilder/CD042/550px/img0033.jpg
https://www.dermis.net/bilder/CD187/550px/img0078.jpg
https://www.dermis.net/bilder/CD187/550px/img0079.jpg
https://www.dermis.net/bilder/CD187/550px/img0080.jpg
https://www.dermis.net/bilder/CD187/550px/img0081.jpg
https://www.dermis.net/bilder/CD187/550px/img0082.jpg
https://www.dermis.net/bilder/CD152/550px/img0018.jpg
https://www.dermis.net/bilder/CD152/550px/img0019.jpg
https://www.dermis.net/bilder/CD152/550px/img0020.jpg
https://www.dermis.net/bilder/CD151/550px/img0097.jpg
https://www.dermis.net/bilder/CD183/550px/img0022.jpg
https://www.dermis.net/bilder/CD183/550px/img0024.jpg
https://www.dermis.net/bilder/CD183/550px/img0025.jpg
https://www.dermis.net/bilder/CD076/550px/img0099.jpg
https://www.dermis.net/bilder/CD076/550px/img0100.jpg
https://www.dermis.net/bilder/CD076/550px/img0101.jpg
https://www.dermis.net/bilder/CD077/550px/img0022.jpg
https://www.dermis.net/bilder/CD079/550px/img0035.jpg
https://www.dermis.net/bilder/CD077/550px/img0023.jpg
https://www.dermis.net/bilder/CD077/550px/img0024.jpg
https://www.dermis.net/bilder/CD079/550px/img0034.jpg
https://www.dermis.net/bilder/CD154/550px/img0011.jpg
https://www.dermis.net/bilder/CD079/550px/img0037.jpg
https://www.dermis.net/bilder/CD079/550px/img0039.jpg
https://www.dermis.net/bilder/CD185/550px/img0006.jpg
https://www.dermis.net/bilder/CD185/550px/img0007.jpg
https://www.dermis.net/bilder/CD185/550px/img0008.jpg

https://www.dermis.net/bilder/CD150/550px/img0079.jpg
https://www.dermis.net/bilder/CD011/550px/img0097.jpg
https://www.dermis.net/bilder/CD151/550px/img0054.jpg
https://www.dermis.net/bilder/CD151/550px/img0055.jpg
https://www.dermis.net/bilder/CD061/550px/img0007.jpg
https://www.dermis.net/bilder/CD079/550px/img0056.jpg
https://www.dermis.net/bilder/CD055/550px/img0002.jpg
https://www.dermis.net/bilder/CD055/550px/img0013.jpg
https://www.dermis.net/bilder/CD076/550px/img0077.jpg
https://www.dermis.net/bilder/CD076/550px/img0078.jpg
https://www.dermis.net/bilder/CD077/550px/img0068.jpg
https://www.dermis.net/bilder/CD153/550px/img0039.jpg
https://www.dermis.net/bilder/CD154/550px/img0068.jpg
https://www.dermis.net/bilder/CD210/550px/img0031.jpg
https://www.dermis.net/bilder/CD002/550px/img0014.jpg
https://www.dermis.net/bilder/CD076/550px/img0029.jpg
https://www.dermis.net/bilder/CD043/550px/img0033.jpg
https://www.dermis.net/bilder/CD078/550px/img0010.jpg
https://www.dermis.net/bilder/CD078/550px/img0011.jpg
https://www.dermis.net/bilder/CD078/550px/img0012.jpg
https://www.dermis.net/bilder/CD078/550px/img0013.jpg
https://www.dermis.net/bilder/CD002/550px/img0056.jpg
https://www.dermis.net/bilder/CD039/550px/img0033.jpg
https://www.dermis.net/bilder/CD077/550px/img0006.jpg
https://www.dermis.net/bilder/CD078/550px/img0027.jpg
https://www.dermis.net/bilder/CD079/550px/img0062.jpg
https://www.dermis.net/bilder/CD079/550px/img0086.jpg
https://www.dermis.net/bilder/CD079/550px/img0099.jpg
https://www.dermis.net/bilder/CD150/550px/img0062.jpg
https://www.dermis.net/bilder/CD150/550px/img0097.jpg
https://www.dermis.net/bilder/CD150/550px/img0098.jpg
https://www.dermis.net/bilder/CD151/550px/img0024.jpg
https://www.dermis.net/bilder/CD151/550px/img0057.jpg
https://www.dermis.net/bilder/CD151/550px/img0102.jpg
https://www.dermis.net/bilder/CD152/550px/img0065.jpg
https://www.dermis.net/bilder/CD153/550px/img0084.jpg
https://www.dermis.net/bilder/CD153/550px/img0086.jpg
https://www.dermis.net/bilder/CD154/550px/img0002.jpg
https://www.dermis.net/bilder/CD150/550px/img0077.jpg
https://www.dermis.net/bilder/CD153/550px/img0103.jpg
https://www.dermis.net/bilder/CD187/550px/img0035.jpg
https://www.dermis.net/bilder/CD085/550px/img0015.jpg
https://www.dermis.net/bilder/CD085/550px/img0016.jpg
https://www.dermis.net/bilder/CD085/550px/img0017.jpg
https://www.dermis.net/bilder/CD085/550px/img0032.jpg
https://www.dermis.net/bilder/CD184/550px/img0013.jpg
https://www.dermis.net/bilder/CD151/550px/img0079.jpg
https://www.dermis.net/bilder/CD151/550px/img0080.jpg

https://www.dermis.net/bilder/CD079/550px/img0019.jpg
https://www.dermis.net/bilder/CD079/550px/img0021.jpg
https://www.dermis.net/bilder/CD079/550px/img0022.jpg
https://www.dermis.net/bilder/CD190/550px/img0058.jpg
https://www.dermis.net/bilder/CD028/550px/img0090.jpg
https://www.dermis.net/bilder/CD028/550px/img0092.jpg
https://www.dermis.net/bilder/CD028/550px/img0093.jpg
https://www.dermis.net/bilder/CD028/550px/img0096.jpg
https://www.dermis.net/bilder/CD152/550px/img0083.jpg
https://www.dermis.net/bilder/CD210/550px/img0156.jpg
https://www.dermis.net/bilder/CD013/550px/img0025.jpg
https://www.dermis.net/bilder/CD040/550px/img0048.jpg
https://www.dermis.net/bilder/CD055/550px/img0010.jpg
https://www.dermis.net/bilder/CD055/550px/img0011.jpg
https://www.dermis.net/bilder/CD055/550px/img0015.jpg
https://www.dermis.net/bilder/CD055/550px/img0016.jpg
https://www.dermis.net/bilder/CD055/550px/img0030.jpg
https://www.dermis.net/bilder/CD076/550px/img0052.jpg
https://www.dermis.net/bilder/CD076/550px/img0054.jpg
https://www.dermis.net/bilder/CD076/550px/img0056.jpg
https://www.dermis.net/bilder/CD076/550px/img0064.jpg
https://www.dermis.net/bilder/CD077/550px/img0033.jpg
https://www.dermis.net/bilder/CD077/550px/img0034.jpg
https://www.dermis.net/bilder/CD078/550px/img0071.jpg
https://www.dermis.net/bilder/CD079/550px/img0024.jpg
https://www.dermis.net/bilder/CD079/550px/img0092.jpg
https://www.dermis.net/bilder/CD079/550px/img0097.jpg
https://www.dermis.net/bilder/CD079/550px/img0098.jpg
https://www.dermis.net/bilder/CD150/550px/img0057.jpg
https://www.dermis.net/bilder/CD150/550px/img0068.jpg
https://www.dermis.net/bilder/CD151/550px/img0059.jpg
https://www.dermis.net/bilder/CD153/550px/img0040.jpg
https://www.dermis.net/bilder/CD153/550px/img0060.jpg
https://www.dermis.net/bilder/CD153/550px/img0087.jpg
https://www.dermis.net/bilder/CD153/550px/img0088.jpg
https://www.dermis.net/bilder/CD154/550px/img0042.jpg
https://www.dermis.net/bilder/CD190/550px/img0053.jpg
https://www.dermis.net/bilder/CD185/550px/img0018.jpg
https://www.dermis.net/bilder/CD184/550px/img0078.jpg
https://www.dermis.net/bilder/CD183/550px/img0015.jpg
https://www.dermis.net/bilder/CD193/550px/img0162.jpg
https://www.dermis.net/bilder/CD193/550px/img0163.jpg
https://www.dermis.net/bilder/CD194/550px/img0064.jpg
https://www.dermis.net/bilder/CD195/550px/img0080.jpg
https://www.dermis.net/bilder/CD195/550px/img0081.jpg
https://www.dermis.net/bilder/CD196/550px/img0130.jpg
https://www.dermis.net/bilder/CD196/550px/img0131.jpg
https://www.dermis.net/bilder/CD196/550px/img0132.jpg

https://www.dermis.net/bilder/CD196/550px/img0134.jpg
https://www.dermis.net/bilder/CD196/550px/img0136.jpg
https://www.dermis.net/bilder/CD193/550px/img0172.jpg
https://www.dermis.net/bilder/CD193/550px/img0173.jpg
https://www.dermis.net/bilder/CD210/550px/img0022.jpg
https://www.dermis.net/bilder/CD211/550px/img0071.jpg
https://www.dermis.net/bilder/CD211/550px/img0072.jpg
https://www.dermis.net/bilder/CD076/550px/img0037.jpg
https://www.dermis.net/bilder/CD079/550px/img0001.jpg
https://www.dermis.net/bilder/CD079/550px/img0002.jpg
https://www.dermis.net/bilder/CD079/550px/img0003.jpg
https://www.dermis.net/bilder/CD079/550px/img0004.jpg
https://www.dermis.net/bilder/CD153/550px/img0071.jpg
https://www.dermis.net/bilder/CD153/550px/img0072.jpg
https://www.dermis.net/bilder/CD153/550px/img0073.jpg
https://www.dermis.net/bilder/CD153/550px/img0074.jpg
https://www.dermis.net/bilder/CD033/550px/img0037.jpg
https://www.dermis.net/bilder/CD077/550px/img0070.jpg
https://www.dermis.net/bilder/CD077/550px/img0075.jpg
https://www.dermis.net/bilder/CD077/550px/img0077.jpg
https://www.dermis.net/bilder/CD153/550px/img0092.jpg
https://www.dermis.net/bilder/CD153/550px/img0093.jpg
https://www.dermis.net/bilder/CD042/550px/img0024.jpg
https://www.dermis.net/bilder/CD042/550px/img0025.jpg
https://www.dermis.net/bilder/CD042/550px/img0026.jpg
https://www.dermis.net/bilder/CD016/550px/img0049.jpg
https://www.dermis.net/bilder/CD077/550px/img0016.jpg
https://www.dermis.net/bilder/CD079/550px/img0072.jpg
https://www.dermis.net/bilder/CD052/550px/img0094.jpg
https://www.dermis.net/bilder/CD036/550px/img0065.jpg
https://www.dermis.net/bilder/CD056/550px/img0030.jpg
https://www.dermis.net/bilder/CD185/550px/img0046.jpg
https://www.dermis.net/bilder/CD056/550px/img0078.jpg
https://www.dermis.net/bilder/CD152/550px/img0021.jpg
https://www.dermis.net/bilder/CD152/550px/img0022.jpg
https://www.dermis.net/bilder/CD011/550px/img0082.jpg
https://www.dermis.net/bilder/CD011/550px/img0084.jpg
https://www.dermis.net/bilder/CD065/550px/img0063.jpg
https://www.dermis.net/bilder/CD036/550px/img0027.jpg
https://www.dermis.net/bilder/CD152/550px/img0051.jpg
https://www.dermis.net/bilder/CD152/550px/img0052.jpg
https://www.dermis.net/bilder/CD152/550px/img0053.jpg
https://www.dermis.net/bilder/CD152/550px/img0055.jpg
https://www.dermis.net/bilder/CD154/550px/img0092.jpg
https://www.dermis.net/bilder/CD154/550px/img0093.jpg
https://www.dermis.net/bilder/CD078/550px/img0041.jpg
https://www.dermis.net/bilder/CD078/550px/img0042.jpg
https://www.dermis.net/bilder/CD078/550px/img0043.jpg

https://www.dermis.net/bilder/CD078/550px/img0045.jpg
https://www.dermis.net/bilder/CD150/550px/img0083.jpg
https://www.dermis.net/bilder/CD152/550px/img0003.jpg
https://www.dermis.net/bilder/CD152/550px/img0004.jpg
https://www.dermis.net/bilder/CD152/550px/img0005.jpg
https://www.dermis.net/bilder/CD152/550px/img0006.jpg
https://www.dermis.net/bilder/CD152/550px/img0092.jpg
https://www.dermis.net/bilder/CD077/550px/img0096.jpg
https://www.dermis.net/bilder/CD076/550px/img0073.jpg
https://www.dermis.net/bilder/CD076/550px/img0074.jpg
https://www.dermis.net/bilder/CD151/550px/img0104.jpg
https://www.dermis.net/bilder/CD001/550px/img0031.jpg
https://www.dermis.net/bilder/CD003/550px/img0078.jpg
https://www.dermis.net/bilder/CD006/550px/img0054.jpg
https://www.dermis.net/bilder/CD026/550px/img0062.jpg
https://www.dermis.net/bilder/CD058/550px/img0085.jpg
https://www.dermis.net/bilder/CD058/550px/img0091.jpg
https://www.dermis.net/bilder/CD058/550px/img0092.jpg
https://www.dermis.net/bilder/CD063/550px/img0067.jpg
https://www.dermis.net/bilder/CD063/550px/img0068.jpg
https://www.dermis.net/bilder/CD178/550px/img0028.jpg
https://www.dermis.net/bilder/CD178/550px/img0030.jpg
https://www.dermis.net/bilder/CD181/550px/img0001.jpg
https://www.dermis.net/bilder/CD183/550px/img0013.jpg
https://www.dermis.net/bilder/CD183/550px/img0014.jpg
https://www.dermis.net/bilder/CD184/550px/img0050.jpg
https://www.dermis.net/bilder/CD184/550px/img0052.jpg
https://www.dermis.net/bilder/CD185/550px/img0078.jpg
https://www.dermis.net/bilder/CD185/550px/img0081.jpg
https://www.dermis.net/bilder/CD185/550px/img0082.jpg
https://www.dermis.net/bilder/CD187/550px/img0053.jpg
https://www.dermis.net/bilder/CD187/550px/img0054.jpg
https://www.dermis.net/bilder/CD187/550px/img0055.jpg
https://www.dermis.net/bilder/CD187/550px/img0056.jpg
https://www.dermis.net/bilder/CD187/550px/img0057.jpg
https://www.dermis.net/bilder/CD187/550px/img0070.jpg
https://www.dermis.net/bilder/CD191/550px/img0044.jpg
https://www.dermis.net/bilder/CD191/550px/img0045.jpg
https://www.dermis.net/bilder/CD191/550px/img0046.jpg
https://www.dermis.net/bilder/CD192/550px/img0069.jpg
https://www.dermis.net/bilder/CD194/550px/img0129.jpg
http://www.hellenicdermatlas.com/photos/0000/0001/00000001_standalone.jpg
http://www.hellenicdermatlas.com/photos/0000/0002/00000002_standalone.jpg
http://www.hellenicdermatlas.com/photos/0000/0003/00000003_standalone.jpg
http://www.hellenicdermatlas.com/photos/0000/0004/00000004_standalone.jpg
http://www.hellenicdermatlas.com/photos/0000/0005/00000005_standalone.jpg
http://www.hellenicdermatlas.com/photos/0000/0006/00000006_standalone.jpg
http://www.hellenicdermatlas.com/photos/0000/0007/00000007_standalone.jpg

http://www.hellenicdermatlas.com/photos/0000/0008/00000008_standalone.jpg
http://www.hellenicdermatlas.com/photos/0000/0009/00000009_standalone.jpg
http://www.hellenicdermatlas.com/photos/0000/0010/00000010_standalone.jpg
http://www.hellenicdermatlas.com/photos/0000/0011/00000011_standalone.jpg
http://www.hellenicdermatlas.com/photos/0000/0012/00000012_standalone.jpg
http://www.hellenicdermatlas.com/photos/0000/0013/00000013_standalone.jpg
http://www.hellenicdermatlas.com/photos/0000/0014/00000014_standalone.jpg
http://www.hellenicdermatlas.com/photos/0000/0015/00000015_standalone.jpg
http://www.hellenicdermatlas.com/photos/0000/0016/00000016_standalone.jpg
http://www.hellenicdermatlas.com/photos/0000/0018/00000018_standalone.jpg
http://www.hellenicdermatlas.com/photos/0000/0019/00000019_standalone.jpg
http://www.hellenicdermatlas.com/photos/0000/0020/00000020_standalone.jpg
http://www.hellenicdermatlas.com/photos/0000/0021/00000021_standalone.jpg
http://www.hellenicdermatlas.com/photos/0000/0022/00000022_standalone.jpg
http://www.hellenicdermatlas.com/photos/0000/0023/00000023_standalone.jpg
http://www.hellenicdermatlas.com/photos/0000/0024/00000024_standalone.jpg
http://www.hellenicdermatlas.com/photos/0000/0025/00000025_standalone.jpg
http://www.hellenicdermatlas.com/photos/0000/0026/00000026_standalone.jpg
http://www.hellenicdermatlas.com/photos/0000/0027/00000027_standalone.jpg
http://www.hellenicdermatlas.com/photos/0000/0028/00000028_standalone.jpg
http://www.hellenicdermatlas.com/photos/0000/0029/00000029_standalone.jpg
http://www.hellenicdermatlas.com/photos/0000/0030/00000030_standalone.jpg
http://www.hellenicdermatlas.com/photos/0000/0031/00000031_standalone.jpg
http://www.hellenicdermatlas.com/photos/0000/0032/00000032_standalone.jpg
http://www.hellenicdermatlas.com/photos/0000/0033/00000033_standalone.jpg
http://www.hellenicdermatlas.com/photos/0000/0034/00000034_standalone.jpg
http://www.hellenicdermatlas.com/photos/0000/0035/00000035_standalone.jpg
http://www.hellenicdermatlas.com/photos/0000/0036/00000036_standalone.jpg
http://www.hellenicdermatlas.com/photos/0000/0037/00000037_standalone.jpg
http://www.hellenicdermatlas.com/photos/0000/0038/00000038_standalone.jpg
http://www.hellenicdermatlas.com/photos/0000/0039/00000039_standalone.jpg
http://www.hellenicdermatlas.com/photos/0000/0040/00000040_standalone.jpg
http://www.hellenicdermatlas.com/photos/0000/0041/00000041_standalone.jpg
http://www.hellenicdermatlas.com/photos/0000/0042/00000042_standalone.jpg
http://www.hellenicdermatlas.com/photos/0000/0043/00000043_standalone.jpg
http://www.hellenicdermatlas.com/photos/0000/0044/00000044_standalone.jpg
http://www.hellenicdermatlas.com/photos/0000/0045/00000045_standalone.jpg
http://www.hellenicdermatlas.com/photos/0000/0046/00000046_standalone.jpg
http://www.hellenicdermatlas.com/photos/0000/0047/00000047_standalone.jpg
http://www.hellenicdermatlas.com/photos/0000/0048/00000048_standalone.jpg
http://www.hellenicdermatlas.com/photos/0000/0049/00000049_standalone.jpg
http://www.hellenicdermatlas.com/photos/0000/0050/00000050_standalone.jpg
http://www.hellenicdermatlas.com/photos/0000/0051/00000051_standalone.jpg
http://www.hellenicdermatlas.com/photos/0000/0052/00000052_standalone.jpg
http://www.hellenicdermatlas.com/photos/0000/0053/00000053_standalone.jpg
http://www.hellenicdermatlas.com/photos/0000/0054/00000054_standalone.jpg
http://www.hellenicdermatlas.com/photos/0000/0055/00000055_standalone.jpg
http://www.hellenicdermatlas.com/photos/0000/0056/00000056_standalone.jpg

http://www.hellenicdermatlas.com/photos/0000/0057/00000057_standalone.jpg
http://www.hellenicdermatlas.com/photos/0000/0058/00000058_standalone.jpg
http://www.hellenicdermatlas.com/photos/0000/0059/00000059_standalone.jpg
http://www.hellenicdermatlas.com/photos/0000/0060/00000060_standalone.jpg
http://www.hellenicdermatlas.com/photos/0000/0061/00000061_standalone.jpg
http://www.hellenicdermatlas.com/photos/0000/0062/00000062_standalone.jpg
http://www.hellenicdermatlas.com/photos/0000/0063/00000063_standalone.jpg
http://www.hellenicdermatlas.com/photos/0000/0064/00000064_standalone.jpg
http://www.hellenicdermatlas.com/photos/0000/0065/00000065_standalone.jpg
http://www.hellenicdermatlas.com/photos/0000/0066/00000066_standalone.jpg
http://www.hellenicdermatlas.com/photos/0000/0067/00000067_standalone.jpg
http://www.hellenicdermatlas.com/photos/0000/0068/00000068_standalone.jpg
http://www.hellenicdermatlas.com/photos/0000/0069/00000069_standalone.jpg
http://www.hellenicdermatlas.com/photos/0000/0070/00000070_standalone.jpg
http://www.hellenicdermatlas.com/photos/0000/0071/00000071_standalone.jpg
http://www.hellenicdermatlas.com/photos/0000/0072/00000072_standalone.jpg
http://www.hellenicdermatlas.com/photos/0000/0073/00000073_standalone.jpg
http://www.hellenicdermatlas.com/photos/0000/0074/00000074_standalone.jpg
http://www.hellenicdermatlas.com/photos/0000/0075/00000075_standalone.jpg
http://www.hellenicdermatlas.com/photos/0000/0076/00000076_standalone.jpg
http://www.hellenicdermatlas.com/photos/0000/0077/00000077_standalone.jpg
http://www.hellenicdermatlas.com/photos/0000/0078/00000078_standalone.jpg
http://www.hellenicdermatlas.com/photos/0000/0079/00000079_standalone.jpg
http://www.hellenicdermatlas.com/photos/0000/0080/00000080_standalone.jpg
http://www.hellenicdermatlas.com/photos/0000/0081/00000081_standalone.jpg
http://www.hellenicdermatlas.com/photos/0000/0082/00000082_standalone.jpg
http://www.hellenicdermatlas.com/photos/0000/0083/00000083_standalone.jpg
http://www.hellenicdermatlas.com/photos/0000/0084/00000084_standalone.jpg
http://www.hellenicdermatlas.com/photos/0000/0085/00000085_standalone.jpg
http://www.hellenicdermatlas.com/photos/0000/0086/00000086_standalone.jpg
http://www.hellenicdermatlas.com/photos/0000/0087/00000087_standalone.jpg
http://www.hellenicdermatlas.com/photos/0000/0088/00000088_standalone.jpg
http://www.hellenicdermatlas.com/photos/0000/0089/00000089_standalone.jpg
http://www.hellenicdermatlas.com/photos/0000/0090/00000090_standalone.jpg
http://www.hellenicdermatlas.com/photos/0000/0091/00000091_standalone.jpg
http://www.hellenicdermatlas.com/photos/0000/0092/00000092_standalone.jpg
http://www.hellenicdermatlas.com/photos/0000/0093/00000093_standalone.jpg
http://www.hellenicdermatlas.com/photos/0000/0094/00000094_standalone.jpg
http://www.hellenicdermatlas.com/photos/0000/0095/00000095_standalone.jpg
http://www.hellenicdermatlas.com/photos/0000/0096/00000096_standalone.jpg
http://www.hellenicdermatlas.com/photos/0000/0097/00000097_standalone.jpg
http://www.hellenicdermatlas.com/photos/0000/0098/00000098_standalone.jpg
http://www.hellenicdermatlas.com/photos/0000/0099/00000099_standalone.jpg
http://www.hellenicdermatlas.com/photos/0000/0100/00000100_standalone.jpg
http://www.hellenicdermatlas.com/photos/0000/0101/00000101_standalone.jpg
http://www.hellenicdermatlas.com/photos/0000/0102/00000102_standalone.jpg
http://www.hellenicdermatlas.com/photos/0000/0103/00000103_standalone.jpg
http://www.hellenicdermatlas.com/photos/0000/0104/00000104_standalone.jpg

http://www.hellenicdermatlas.com/photos/0000/0105/00000105_standalone.jpg
http://www.hellenicdermatlas.com/photos/0000/0106/00000106_standalone.jpg
http://www.hellenicdermatlas.com/photos/0000/0107/00000107_standalone.jpg
http://www.hellenicdermatlas.com/photos/0000/0108/00000108_standalone.jpg
http://www.hellenicdermatlas.com/photos/0000/0109/00000109_standalone.jpg
http://www.hellenicdermatlas.com/photos/0000/0110/00000110_standalone.jpg
http://www.hellenicdermatlas.com/photos/0000/0111/00000111_standalone.jpg
http://www.hellenicdermatlas.com/photos/0000/0112/00000112_standalone.jpg
http://www.hellenicdermatlas.com/photos/0000/0113/00000113_standalone.jpg
http://www.hellenicdermatlas.com/photos/0000/0114/00000114_standalone.jpg
http://www.hellenicdermatlas.com/photos/0000/0115/00000115_standalone.jpg
http://www.hellenicdermatlas.com/photos/0000/0116/00000116_standalone.jpg
http://www.hellenicdermatlas.com/photos/0000/0117/00000117_standalone.jpg
http://www.hellenicdermatlas.com/photos/0000/0118/00000118_standalone.jpg
http://www.hellenicdermatlas.com/photos/0000/0119/00000119_standalone.jpg
http://www.hellenicdermatlas.com/photos/0000/0120/00000120_standalone.jpg
http://www.hellenicdermatlas.com/photos/0000/0121/00000121_standalone.jpg
http://www.hellenicdermatlas.com/photos/0000/0122/00000122_standalone.jpg
http://www.hellenicdermatlas.com/photos/0000/0123/00000123_standalone.jpg
http://www.hellenicdermatlas.com/photos/0000/0124/00000124_standalone.jpg
http://www.hellenicdermatlas.com/photos/0000/0125/00000125_standalone.jpg
http://www.hellenicdermatlas.com/photos/0000/0126/00000126_standalone.jpg
http://www.hellenicdermatlas.com/photos/0000/0127/00000127_standalone.jpg
http://www.hellenicdermatlas.com/photos/0000/0128/00000128_standalone.jpg
http://www.hellenicdermatlas.com/photos/0000/0129/00000129_standalone.jpg
http://www.hellenicdermatlas.com/photos/0000/0130/00000130_standalone.jpg
http://www.hellenicdermatlas.com/photos/0000/0131/00000131_standalone.jpg
http://www.hellenicdermatlas.com/photos/0000/0132/00000132_standalone.jpg
http://www.hellenicdermatlas.com/photos/0000/0133/00000133_standalone.jpg
http://www.hellenicdermatlas.com/photos/0000/0134/00000134_standalone.jpg
http://www.hellenicdermatlas.com/photos/0000/0135/00000135_standalone.jpg
http://www.hellenicdermatlas.com/photos/0000/0136/00000136_standalone.jpg
http://www.hellenicdermatlas.com/photos/0000/0137/00000137_standalone.jpg
http://www.hellenicdermatlas.com/photos/0000/0138/00000138_standalone.jpg
http://www.hellenicdermatlas.com/photos/0000/0139/00000139_standalone.jpg
http://www.hellenicdermatlas.com/photos/0000/0140/00000140_standalone.jpg
http://www.hellenicdermatlas.com/photos/0000/0141/00000141_standalone.jpg
http://www.hellenicdermatlas.com/photos/0000/0142/00000142_standalone.jpg
http://www.hellenicdermatlas.com/photos/0000/0143/00000143_standalone.jpg
http://www.hellenicdermatlas.com/photos/0000/0144/00000144_standalone.jpg
http://www.hellenicdermatlas.com/photos/0000/0145/00000145_standalone.jpg
http://www.hellenicdermatlas.com/photos/0000/0146/00000146_standalone.jpg
http://www.hellenicdermatlas.com/photos/0000/0147/00000147_standalone.jpg
http://www.hellenicdermatlas.com/photos/0000/0148/00000148_standalone.jpg
http://www.hellenicdermatlas.com/photos/0000/0149/00000149_standalone.jpg
http://www.hellenicdermatlas.com/photos/0000/0150/00000150_standalone.jpg
http://www.hellenicdermatlas.com/photos/0000/0151/00000151_standalone.jpg
http://www.hellenicdermatlas.com/photos/0000/0152/00000152_standalone.jpg

http://www.hellenicdermatlas.com/photos/0000/0153/00000153_standalone.jpg
http://www.hellenicdermatlas.com/photos/0000/0154/00000154_standalone.jpg
http://www.hellenicdermatlas.com/photos/0000/0155/00000155_standalone.jpg
http://www.hellenicdermatlas.com/photos/0000/0156/00000156_standalone.jpg
http://www.hellenicdermatlas.com/photos/0000/0157/00000157_standalone.jpg
http://www.hellenicdermatlas.com/photos/0000/0158/00000158_standalone.jpg
http://www.hellenicdermatlas.com/photos/0000/0159/00000159_standalone.jpg
http://www.hellenicdermatlas.com/photos/0000/0160/00000160_standalone.jpg
http://www.hellenicdermatlas.com/photos/0000/0161/00000161_standalone.jpg
http://www.hellenicdermatlas.com/photos/0000/0162/00000162_standalone.jpg
http://www.hellenicdermatlas.com/photos/0000/0163/00000163_standalone.jpg
http://www.hellenicdermatlas.com/photos/0000/0164/00000164_standalone.jpg
http://www.hellenicdermatlas.com/photos/0000/0165/00000165_standalone.jpg
http://www.hellenicdermatlas.com/photos/0000/0166/00000166_standalone.jpg
http://www.hellenicdermatlas.com/photos/0000/0167/00000167_standalone.jpg
http://www.hellenicdermatlas.com/photos/0000/0168/00000168_standalone.jpg
http://www.hellenicdermatlas.com/photos/0000/0169/00000169_standalone.jpg
http://www.hellenicdermatlas.com/photos/0000/0170/00000170_standalone.jpg
http://www.hellenicdermatlas.com/photos/0000/0171/00000171_standalone.jpg
http://www.hellenicdermatlas.com/photos/0000/0172/00000172_standalone.jpg
http://www.hellenicdermatlas.com/photos/0000/0173/00000173_standalone.jpg
http://www.hellenicdermatlas.com/photos/0000/0174/00000174_standalone.jpg
http://www.hellenicdermatlas.com/photos/0000/0175/00000175_standalone.jpg
http://www.hellenicdermatlas.com/photos/0000/0176/00000176_standalone.jpg
http://www.hellenicdermatlas.com/photos/0000/0177/00000177_standalone.jpg
http://www.hellenicdermatlas.com/photos/0000/0178/00000178_standalone.jpg
http://www.hellenicdermatlas.com/photos/0000/0179/00000179_standalone.jpg
http://www.hellenicdermatlas.com/photos/0000/0181/00000181_standalone.jpg
http://www.hellenicdermatlas.com/photos/0000/0182/00000182_standalone.jpg
http://www.hellenicdermatlas.com/photos/0000/0183/00000183_standalone.jpg
http://www.hellenicdermatlas.com/photos/0000/0184/00000184_standalone.jpg
http://www.hellenicdermatlas.com/photos/0000/0185/00000185_standalone.jpg
http://www.hellenicdermatlas.com/photos/0000/0186/00000186_standalone.jpg
http://www.hellenicdermatlas.com/photos/0000/0187/00000187_standalone.jpg
http://www.hellenicdermatlas.com/photos/0000/0188/00000188_standalone.jpg
http://www.hellenicdermatlas.com/photos/0000/0189/00000189_standalone.jpg
http://www.hellenicdermatlas.com/photos/0000/0190/00000190_standalone.jpg
http://www.hellenicdermatlas.com/photos/0000/0191/00000191_standalone.jpg
http://www.hellenicdermatlas.com/photos/0000/0192/00000192_standalone.jpg
http://www.hellenicdermatlas.com/photos/0000/0193/00000193_standalone.jpg
http://www.hellenicdermatlas.com/photos/0000/0194/00000194_standalone.jpg
http://www.hellenicdermatlas.com/photos/0000/0195/00000195_standalone.jpg
http://www.hellenicdermatlas.com/photos/0000/0196/00000196_standalone.jpg
http://www.hellenicdermatlas.com/photos/0000/0197/00000197_standalone.jpg
http://www.hellenicdermatlas.com/photos/0000/0198/00000198_standalone.jpg
http://www.hellenicdermatlas.com/photos/0000/0199/00000199_standalone.jpg
http://www.hellenicdermatlas.com/photos/0000/0200/00000200_standalone.jpg
http://www.hellenicdermatlas.com/photos/0000/0201/00000201_standalone.jpg

http://www.hellenicdermatlas.com/photos/0000/0202/00000202_standalone.jpg
http://www.hellenicdermatlas.com/photos/0000/0203/00000203_standalone.jpg
http://www.hellenicdermatlas.com/photos/0000/0204/00000204_standalone.jpg
http://www.hellenicdermatlas.com/photos/0000/0205/00000205_standalone.jpg
http://www.hellenicdermatlas.com/photos/0000/0206/00000206_standalone.jpg
http://www.hellenicdermatlas.com/photos/0000/0207/00000207_standalone.jpg
http://www.hellenicdermatlas.com/photos/0000/0208/00000208_standalone.jpg
http://www.hellenicdermatlas.com/photos/0000/0209/00000209_standalone.jpg
http://www.hellenicdermatlas.com/photos/0000/0210/00000210_standalone.jpg
http://www.hellenicdermatlas.com/photos/0000/0211/00000211_standalone.jpg
http://www.hellenicdermatlas.com/photos/0000/0212/00000212_standalone.jpg
http://www.hellenicdermatlas.com/photos/0000/0213/00000213_standalone.jpg
http://www.hellenicdermatlas.com/photos/0000/0214/00000214_standalone.jpg
http://www.hellenicdermatlas.com/photos/0000/0215/00000215_standalone.jpg
http://www.hellenicdermatlas.com/photos/0000/0216/00000216_standalone.jpg
http://www.hellenicdermatlas.com/photos/0000/0217/00000217_standalone.jpg
http://www.hellenicdermatlas.com/photos/0000/0218/00000218_standalone.jpg
http://www.hellenicdermatlas.com/photos/0000/0219/00000219_standalone.jpg
http://www.hellenicdermatlas.com/photos/0000/0220/00000220_standalone.jpg
http://www.hellenicdermatlas.com/photos/0000/0221/00000221_standalone.jpg
http://www.hellenicdermatlas.com/photos/0000/0222/00000222_standalone.jpg
http://www.hellenicdermatlas.com/photos/0000/0223/00000223_standalone.jpg
http://www.hellenicdermatlas.com/photos/0000/0224/00000224_standalone.jpg
http://www.hellenicdermatlas.com/photos/0000/0225/00000225_standalone.jpg
http://www.hellenicdermatlas.com/photos/0000/0226/00000226_standalone.jpg
http://www.hellenicdermatlas.com/photos/0000/0227/00000227_standalone.jpg
http://www.hellenicdermatlas.com/photos/0000/0228/00000228_standalone.jpg
http://www.hellenicdermatlas.com/photos/0000/0229/00000229_standalone.jpg
http://www.hellenicdermatlas.com/photos/0000/0230/00000230_standalone.jpg
http://www.hellenicdermatlas.com/photos/0000/0231/00000231_standalone.jpg
http://www.hellenicdermatlas.com/photos/0000/0232/00000232_standalone.jpg
http://www.hellenicdermatlas.com/photos/0000/0233/00000233_standalone.jpg
http://www.hellenicdermatlas.com/photos/0000/0234/00000234_standalone.jpg
http://www.hellenicdermatlas.com/photos/0000/0235/00000235_standalone.jpg
http://www.hellenicdermatlas.com/photos/0000/0236/00000236_standalone.jpg
http://www.hellenicdermatlas.com/photos/0000/0237/00000237_standalone.jpg
http://www.hellenicdermatlas.com/photos/0000/0238/00000238_standalone.jpg
http://www.hellenicdermatlas.com/photos/0000/0239/00000239_standalone.jpg
http://www.hellenicdermatlas.com/photos/0000/0240/00000240_standalone.jpg
http://www.hellenicdermatlas.com/photos/0000/0241/00000241_standalone.jpg
http://www.hellenicdermatlas.com/photos/0000/0242/00000242_standalone.jpg
http://www.hellenicdermatlas.com/photos/0000/0243/00000243_standalone.jpg
http://www.hellenicdermatlas.com/photos/0000/0244/00000244_standalone.jpg
http://www.hellenicdermatlas.com/photos/0000/0245/00000245_standalone.jpg
http://www.hellenicdermatlas.com/photos/0000/0246/00000246_standalone.jpg
http://www.hellenicdermatlas.com/photos/0000/0247/00000247_standalone.jpg
http://www.hellenicdermatlas.com/photos/0000/0248/00000248_standalone.jpg
http://www.hellenicdermatlas.com/photos/0000/0249/00000249_standalone.jpg

http://www.hellenicdermatlas.com/photos/0000/0250/00000250_standalone.jpg
http://www.hellenicdermatlas.com/photos/0000/0251/00000251_standalone.jpg
http://www.hellenicdermatlas.com/photos/0000/0252/00000252_standalone.jpg
http://www.hellenicdermatlas.com/photos/0000/0253/00000253_standalone.jpg
http://www.hellenicdermatlas.com/photos/0000/0254/00000254_standalone.jpg
http://www.hellenicdermatlas.com/photos/0000/0255/00000255_standalone.jpg
http://www.hellenicdermatlas.com/photos/0000/0256/00000256_standalone.jpg
http://www.hellenicdermatlas.com/photos/0000/0257/00000257_standalone.jpg
http://www.hellenicdermatlas.com/photos/0000/0258/00000258_standalone.jpg
http://www.hellenicdermatlas.com/photos/0000/0259/00000259_standalone.jpg
http://www.hellenicdermatlas.com/photos/0000/0260/00000260_standalone.jpg
http://www.hellenicdermatlas.com/photos/0000/0261/00000261_standalone.jpg
http://www.hellenicdermatlas.com/photos/0000/0262/00000262_standalone.jpg
http://www.hellenicdermatlas.com/photos/0000/0263/00000263_standalone.jpg
http://www.hellenicdermatlas.com/photos/0000/0264/00000264_standalone.jpg
http://www.hellenicdermatlas.com/photos/0000/0265/00000265_standalone.jpg
http://www.hellenicdermatlas.com/photos/0000/0266/00000266_standalone.jpg
http://www.hellenicdermatlas.com/photos/0000/0267/00000267_standalone.jpg
http://www.hellenicdermatlas.com/photos/0000/0268/00000268_standalone.jpg
http://www.hellenicdermatlas.com/photos/0000/0269/00000269_standalone.jpg
http://www.hellenicdermatlas.com/photos/0000/0270/00000270_standalone.jpg
http://www.hellenicdermatlas.com/photos/0000/0271/00000271_standalone.jpg
http://www.hellenicdermatlas.com/photos/0000/0272/00000272_standalone.jpg
http://www.hellenicdermatlas.com/photos/0000/0273/00000273_standalone.jpg
http://www.hellenicdermatlas.com/photos/0000/0274/00000274_standalone.jpg
http://www.hellenicdermatlas.com/photos/0000/0275/00000275_standalone.jpg
http://www.hellenicdermatlas.com/photos/0000/0276/00000276_standalone.jpg
http://www.hellenicdermatlas.com/photos/0000/0277/00000277_standalone.jpg
http://www.hellenicdermatlas.com/photos/0000/0278/00000278_standalone.jpg
http://www.hellenicdermatlas.com/photos/0000/0279/00000279_standalone.jpg
http://www.hellenicdermatlas.com/photos/0000/0280/00000280_standalone.jpg
http://www.hellenicdermatlas.com/photos/0000/0281/00000281_standalone.jpg
http://www.hellenicdermatlas.com/photos/0000/0282/00000282_standalone.jpg
http://www.hellenicdermatlas.com/photos/0000/0283/00000283_standalone.jpg
http://www.hellenicdermatlas.com/photos/0000/0284/00000284_standalone.jpg
http://www.hellenicdermatlas.com/photos/0000/0285/00000285_standalone.jpg
http://www.hellenicdermatlas.com/photos/0000/0286/00000286_standalone.jpg
http://www.hellenicdermatlas.com/photos/0000/0287/00000287_standalone.jpg
http://www.hellenicdermatlas.com/photos/0000/0288/00000288_standalone.jpg
http://www.hellenicdermatlas.com/photos/0000/0289/00000289_standalone.jpg
http://www.hellenicdermatlas.com/photos/0000/0290/00000290_standalone.jpg
http://www.hellenicdermatlas.com/photos/0000/0291/00000291_standalone.jpg
http://www.hellenicdermatlas.com/photos/0000/0292/00000292_standalone.jpg
http://www.hellenicdermatlas.com/photos/0000/0293/00000293_standalone.jpg
http://www.hellenicdermatlas.com/photos/0000/0294/00000294_standalone.jpg
http://www.hellenicdermatlas.com/photos/0000/0295/00000295_standalone.jpg
http://www.hellenicdermatlas.com/photos/0000/0296/00000296_standalone.jpg
http://www.hellenicdermatlas.com/photos/0000/0297/00000297_standalone.jpg

http://www.hellenicdermatlas.com/photos/0000/0298/00000298_standalone.jpg
http://www.hellenicdermatlas.com/photos/0000/0299/00000299_standalone.jpg
http://www.hellenicdermatlas.com/photos/0000/0300/00000300_standalone.jpg
http://www.hellenicdermatlas.com/photos/0000/0301/00000301_standalone.jpg
http://www.hellenicdermatlas.com/photos/0000/0302/00000302_standalone.jpg
http://www.hellenicdermatlas.com/photos/0000/0303/00000303_standalone.jpg
http://www.hellenicdermatlas.com/photos/0000/0304/00000304_standalone.jpg
http://www.hellenicdermatlas.com/photos/0000/0305/00000305_standalone.jpg
http://www.hellenicdermatlas.com/photos/0000/0306/00000306_standalone.jpg
http://www.hellenicdermatlas.com/photos/0000/0307/00000307_standalone.jpg
http://www.hellenicdermatlas.com/photos/0000/0308/00000308_standalone.jpg
http://www.hellenicdermatlas.com/photos/0000/0309/00000309_standalone.jpg
http://www.hellenicdermatlas.com/photos/0000/0310/00000310_standalone.jpg
http://www.hellenicdermatlas.com/photos/0000/0311/00000311_standalone.jpg
http://www.hellenicdermatlas.com/photos/0000/0312/00000312_standalone.jpg
http://www.hellenicdermatlas.com/photos/0000/0313/00000313_standalone.jpg
http://www.hellenicdermatlas.com/photos/0000/0314/00000314_standalone.jpg
http://www.hellenicdermatlas.com/photos/0000/0315/00000315_standalone.jpg
http://www.hellenicdermatlas.com/photos/0000/0316/00000316_standalone.jpg
http://www.hellenicdermatlas.com/photos/0000/0317/00000317_standalone.jpg
http://www.hellenicdermatlas.com/photos/0000/0318/00000318_standalone.jpg
http://www.hellenicdermatlas.com/photos/0000/0319/00000319_standalone.jpg
http://www.hellenicdermatlas.com/photos/0000/0320/00000320_standalone.jpg
http://www.hellenicdermatlas.com/photos/0000/0321/00000321_standalone.jpg
http://www.hellenicdermatlas.com/photos/0000/0322/00000322_standalone.jpg
http://www.hellenicdermatlas.com/photos/0000/0323/00000323_standalone.jpg
http://www.hellenicdermatlas.com/photos/0000/0324/00000324_standalone.jpg
http://www.hellenicdermatlas.com/photos/0000/0325/00000325_standalone.jpg
http://www.hellenicdermatlas.com/photos/0000/0326/00000326_standalone.jpg
http://www.hellenicdermatlas.com/photos/0000/0327/00000327_standalone.jpg
http://www.hellenicdermatlas.com/photos/0000/0328/00000328_standalone.jpg
http://www.hellenicdermatlas.com/photos/0000/0329/00000329_standalone.jpg
http://www.hellenicdermatlas.com/photos/0000/0330/00000330_standalone.jpg
http://www.hellenicdermatlas.com/photos/0000/0331/00000331_standalone.jpg
http://www.hellenicdermatlas.com/photos/0000/0332/00000332_standalone.jpg
http://www.hellenicdermatlas.com/photos/0000/0333/00000333_standalone.jpg
http://www.hellenicdermatlas.com/photos/0000/0334/00000334_standalone.jpg
http://www.hellenicdermatlas.com/photos/0000/0335/00000335_standalone.jpg
http://www.hellenicdermatlas.com/photos/0000/0336/00000336_standalone.jpg
http://www.hellenicdermatlas.com/photos/0000/0337/00000337_standalone.jpg
http://www.hellenicdermatlas.com/photos/0000/0338/00000338_standalone.jpg
http://www.hellenicdermatlas.com/photos/0000/0339/00000339_standalone.jpg
http://www.hellenicdermatlas.com/photos/0000/0340/00000340_standalone.jpg
http://www.hellenicdermatlas.com/photos/0000/0341/00000341_standalone.jpg
http://www.hellenicdermatlas.com/photos/0000/0342/00000342_standalone.jpg
http://www.hellenicdermatlas.com/photos/0000/0343/00000343_standalone.jpg
http://www.hellenicdermatlas.com/photos/0000/0344/00000344_standalone.jpg
http://www.hellenicdermatlas.com/photos/0000/0345/00000345_standalone.jpg

http://www.hellenicdermatlas.com/photos/0000/0346/00000346_standalone.jpg
http://www.hellenicdermatlas.com/photos/0000/0347/00000347_standalone.jpg
http://www.hellenicdermatlas.com/photos/0000/0348/00000348_standalone.jpg
http://www.hellenicdermatlas.com/photos/0000/0349/00000349_standalone.jpg
http://www.hellenicdermatlas.com/photos/0000/0350/00000350_standalone.jpg
http://www.hellenicdermatlas.com/photos/0000/0351/00000351_standalone.jpg
http://www.hellenicdermatlas.com/photos/0000/0352/00000352_standalone.jpg
http://www.hellenicdermatlas.com/photos/0000/0353/00000353_standalone.jpg
http://www.hellenicdermatlas.com/photos/0000/0354/00000354_standalone.jpg
http://www.hellenicdermatlas.com/photos/0000/0355/00000355_standalone.jpg
http://www.hellenicdermatlas.com/photos/0000/0356/00000356_standalone.jpg
http://www.hellenicdermatlas.com/photos/0000/0357/00000357_standalone.jpg
http://www.hellenicdermatlas.com/photos/0000/0358/00000358_standalone.jpg
http://www.hellenicdermatlas.com/photos/0000/0359/00000359_standalone.jpg
http://www.hellenicdermatlas.com/photos/0000/0360/00000360_standalone.jpg
http://www.hellenicdermatlas.com/photos/0000/0361/00000361_standalone.jpg
http://www.hellenicdermatlas.com/photos/0000/0362/00000362_standalone.jpg
http://www.hellenicdermatlas.com/photos/0000/0363/00000363_standalone.jpg
http://www.hellenicdermatlas.com/photos/0000/0364/00000364_standalone.jpg
http://www.hellenicdermatlas.com/photos/0000/0365/00000365_standalone.jpg
http://www.hellenicdermatlas.com/photos/0000/0366/00000366_standalone.jpg
http://www.hellenicdermatlas.com/photos/0000/0367/00000367_standalone.jpg
http://www.hellenicdermatlas.com/photos/0000/0368/00000368_standalone.jpg
http://www.hellenicdermatlas.com/photos/0000/0369/00000369_standalone.jpg
http://www.hellenicdermatlas.com/photos/0000/0370/00000370_standalone.jpg
http://www.hellenicdermatlas.com/photos/0000/0371/00000371_standalone.jpg
http://www.hellenicdermatlas.com/photos/0000/0372/00000372_standalone.jpg
http://www.hellenicdermatlas.com/photos/0000/0373/00000373_standalone.jpg
http://www.hellenicdermatlas.com/photos/0000/0374/00000374_standalone.jpg
http://www.hellenicdermatlas.com/photos/0000/0375/00000375_standalone.jpg
http://www.hellenicdermatlas.com/photos/0000/0376/00000376_standalone.jpg
http://www.hellenicdermatlas.com/photos/0000/0377/00000377_standalone.jpg
http://www.hellenicdermatlas.com/photos/0000/0378/00000378_standalone.jpg
http://www.hellenicdermatlas.com/photos/0000/0379/00000379_standalone.jpg
http://www.hellenicdermatlas.com/photos/0000/0380/00000380_standalone.jpg
http://www.hellenicdermatlas.com/photos/0000/0381/00000381_standalone.jpg
http://www.hellenicdermatlas.com/photos/0000/0382/00000382_standalone.jpg
http://www.hellenicdermatlas.com/photos/0000/0383/00000383_standalone.jpg
http://www.hellenicdermatlas.com/photos/0000/0384/00000384_standalone.jpg
http://www.hellenicdermatlas.com/photos/0000/0385/00000385_standalone.jpg
http://www.hellenicdermatlas.com/photos/0000/0386/00000386_standalone.jpg
http://www.hellenicdermatlas.com/photos/0000/0387/00000387_standalone.jpg
http://www.hellenicdermatlas.com/photos/0000/0388/00000388_standalone.jpg
http://www.hellenicdermatlas.com/photos/0000/0389/00000389_standalone.jpg
http://www.hellenicdermatlas.com/photos/0000/0390/00000390_standalone.jpg
http://www.hellenicdermatlas.com/photos/0000/0391/00000391_standalone.jpg
http://www.hellenicdermatlas.com/photos/0000/0392/00000392_standalone.jpg
http://www.hellenicdermatlas.com/photos/0000/0393/00000393_standalone.jpg

http://www.hellenicdermatlas.com/photos/0000/0394/00000394_standalone.jpg
http://www.hellenicdermatlas.com/photos/0000/0395/00000395_standalone.jpg
http://www.hellenicdermatlas.com/photos/0000/0396/00000396_standalone.jpg
http://www.hellenicdermatlas.com/photos/0000/0397/00000397_standalone.jpg
http://www.hellenicdermatlas.com/photos/0000/0398/00000398_standalone.jpg
http://www.hellenicdermatlas.com/photos/0000/0399/00000399_standalone.jpg
http://www.hellenicdermatlas.com/photos/0000/0400/00000400_standalone.jpg
http://www.hellenicdermatlas.com/photos/0000/0401/00000401_standalone.jpg
http://www.hellenicdermatlas.com/photos/0000/0402/00000402_standalone.jpg
http://www.hellenicdermatlas.com/photos/0000/0403/00000403_standalone.jpg
http://www.hellenicdermatlas.com/photos/0000/0404/00000404_standalone.jpg
http://www.hellenicdermatlas.com/photos/0000/0405/00000405_standalone.jpg
http://www.hellenicdermatlas.com/photos/0000/0406/00000406_standalone.jpg
http://www.hellenicdermatlas.com/photos/0000/0407/00000407_standalone.jpg
http://www.hellenicdermatlas.com/photos/0000/0408/00000408_standalone.jpg
http://www.hellenicdermatlas.com/photos/0000/0409/00000409_standalone.jpg
http://www.hellenicdermatlas.com/photos/0000/0410/00000410_standalone.jpg
http://www.hellenicdermatlas.com/photos/0000/0411/00000411_standalone.jpg
http://www.hellenicdermatlas.com/photos/0000/0412/00000412_standalone.jpg
http://www.hellenicdermatlas.com/photos/0000/0413/00000413_standalone.jpg
http://www.hellenicdermatlas.com/photos/0000/0414/00000414_standalone.jpg
http://www.hellenicdermatlas.com/photos/0000/0415/00000415_standalone.jpg
http://www.hellenicdermatlas.com/photos/0000/0416/00000416_standalone.jpg
http://www.hellenicdermatlas.com/photos/0000/0417/00000417_standalone.jpg
http://www.hellenicdermatlas.com/photos/0000/0418/00000418_standalone.jpg
http://www.hellenicdermatlas.com/photos/0000/0419/00000419_standalone.jpg
http://www.hellenicdermatlas.com/photos/0000/0420/00000420_standalone.jpg
http://www.hellenicdermatlas.com/photos/0000/0421/00000421_standalone.jpg
http://www.hellenicdermatlas.com/photos/0000/0422/00000422_standalone.jpg
http://www.hellenicdermatlas.com/photos/0000/0423/00000423_standalone.jpg
http://www.hellenicdermatlas.com/photos/0000/0424/00000424_standalone.jpg
http://www.hellenicdermatlas.com/photos/0000/0425/00000425_standalone.jpg
http://www.hellenicdermatlas.com/photos/0000/0426/00000426_standalone.jpg
http://www.hellenicdermatlas.com/photos/0000/0427/00000427_standalone.jpg
http://www.hellenicdermatlas.com/photos/0000/0428/00000428_standalone.jpg
http://www.hellenicdermatlas.com/photos/0000/0429/00000429_standalone.jpg
http://www.hellenicdermatlas.com/photos/0000/0430/00000430_standalone.jpg
http://www.hellenicdermatlas.com/photos/0000/0431/00000431_standalone.jpg
http://www.hellenicdermatlas.com/photos/0000/0432/00000432_standalone.jpg
http://www.hellenicdermatlas.com/photos/0000/0433/00000433_standalone.jpg
http://www.hellenicdermatlas.com/photos/0000/0434/00000434_standalone.jpg
http://www.hellenicdermatlas.com/photos/0000/0435/00000435_standalone.jpg
http://www.hellenicdermatlas.com/photos/0000/0436/00000436_standalone.jpg
http://www.hellenicdermatlas.com/photos/0000/0437/00000437_standalone.jpg
http://www.hellenicdermatlas.com/photos/0000/0438/00000438_standalone.jpg
http://www.hellenicdermatlas.com/photos/0000/0439/00000439_standalone.jpg
http://www.hellenicdermatlas.com/photos/0000/0440/00000440_standalone.jpg
http://www.hellenicdermatlas.com/photos/0000/0441/00000441_standalone.jpg

http://www.hellenicdermatlas.com/photos/0000/0442/00000442_standalone.jpg
http://www.hellenicdermatlas.com/photos/0000/0443/00000443_standalone.jpg
http://www.hellenicdermatlas.com/photos/0000/0444/00000444_standalone.jpg
http://www.hellenicdermatlas.com/photos/0000/0445/00000445_standalone.jpg
http://www.hellenicdermatlas.com/photos/0000/0446/00000446_standalone.jpg
http://www.hellenicdermatlas.com/photos/0000/0447/00000447_standalone.jpg
http://www.hellenicdermatlas.com/photos/0000/0448/00000448_standalone.jpg
http://www.hellenicdermatlas.com/photos/0000/0449/00000449_standalone.jpg
http://www.hellenicdermatlas.com/photos/0000/0450/00000450_standalone.jpg
http://www.hellenicdermatlas.com/photos/0000/0451/00000451_standalone.jpg
http://www.hellenicdermatlas.com/photos/0000/0452/00000452_standalone.jpg
http://www.hellenicdermatlas.com/photos/0000/0453/00000453_standalone.jpg
http://www.hellenicdermatlas.com/photos/0000/0454/00000454_standalone.jpg
http://www.hellenicdermatlas.com/photos/0000/0455/00000455_standalone.jpg
http://www.hellenicdermatlas.com/photos/0000/0456/00000456_standalone.jpg
http://www.hellenicdermatlas.com/photos/0000/0457/00000457_standalone.jpg
http://www.hellenicdermatlas.com/photos/0000/0458/00000458_standalone.jpg
http://www.hellenicdermatlas.com/photos/0000/0459/00000459_standalone.jpg
http://www.hellenicdermatlas.com/photos/0000/0460/00000460_standalone.jpg
http://www.hellenicdermatlas.com/photos/0000/0461/00000461_standalone.jpg
http://www.hellenicdermatlas.com/photos/0000/0462/00000462_standalone.jpg
http://www.hellenicdermatlas.com/photos/0000/0463/00000463_standalone.jpg
http://www.hellenicdermatlas.com/photos/0000/0464/00000464_standalone.jpg
http://www.hellenicdermatlas.com/photos/0000/0465/00000465_standalone.jpg
http://www.hellenicdermatlas.com/photos/0000/0466/00000466_standalone.jpg
http://www.hellenicdermatlas.com/photos/0000/0467/00000467_standalone.jpg
http://www.hellenicdermatlas.com/photos/0000/0468/00000468_standalone.jpg
http://www.hellenicdermatlas.com/photos/0000/0469/00000469_standalone.jpg
http://www.hellenicdermatlas.com/photos/0000/0470/00000470_standalone.jpg
http://www.hellenicdermatlas.com/photos/0000/0471/00000471_standalone.jpg
http://www.hellenicdermatlas.com/photos/0000/0472/00000472_standalone.jpg
http://www.hellenicdermatlas.com/photos/0000/0473/00000473_standalone.jpg
http://www.hellenicdermatlas.com/photos/0000/0474/00000474_standalone.jpg
http://www.hellenicdermatlas.com/photos/0000/0475/00000475_standalone.jpg
http://www.hellenicdermatlas.com/photos/0000/0476/00000476_standalone.jpg
http://www.hellenicdermatlas.com/photos/0000/0477/00000477_standalone.jpg
http://www.hellenicdermatlas.com/photos/0000/0478/00000478_standalone.jpg
http://www.hellenicdermatlas.com/photos/0000/0479/00000479_standalone.jpg
http://www.hellenicdermatlas.com/photos/0000/0480/00000480_standalone.jpg
http://www.hellenicdermatlas.com/photos/0000/0481/00000481_standalone.jpg
http://www.hellenicdermatlas.com/photos/0000/0482/00000482_standalone.jpg
http://www.hellenicdermatlas.com/photos/0000/0483/00000483_standalone.jpg
http://www.hellenicdermatlas.com/photos/0000/0484/00000484_standalone.jpg
http://www.hellenicdermatlas.com/photos/0000/0485/00000485_standalone.jpg
http://www.hellenicdermatlas.com/photos/0000/0486/00000486_standalone.jpg
http://www.hellenicdermatlas.com/photos/0000/0487/00000487_standalone.jpg
http://www.hellenicdermatlas.com/photos/0000/0488/00000488_standalone.jpg
http://www.hellenicdermatlas.com/photos/0000/0489/00000489_standalone.jpg

http://www.hellenicdermatlas.com/photos/0000/0490/00000490_standalone.jpg
http://www.hellenicdermatlas.com/photos/0000/0491/00000491_standalone.jpg
http://www.hellenicdermatlas.com/photos/0000/0492/00000492_standalone.jpg
http://www.hellenicdermatlas.com/photos/0000/0493/00000493_standalone.jpg
http://www.hellenicdermatlas.com/photos/0000/0494/00000494_standalone.jpg
http://www.hellenicdermatlas.com/photos/0000/0495/00000495_standalone.jpg
http://www.hellenicdermatlas.com/photos/0000/0496/00000496_standalone.jpg
http://www.hellenicdermatlas.com/photos/0000/0497/00000497_standalone.jpg
http://www.hellenicdermatlas.com/photos/0000/0498/00000498_standalone.jpg
http://www.hellenicdermatlas.com/photos/0000/0499/00000499_standalone.jpg
http://www.hellenicdermatlas.com/photos/0000/0500/00000500_standalone.jpg
http://www.hellenicdermatlas.com/photos/0000/0501/00000501_standalone.jpg
http://www.hellenicdermatlas.com/photos/0000/0502/00000502_standalone.jpg
http://www.hellenicdermatlas.com/photos/0000/0503/00000503_standalone.jpg
http://www.hellenicdermatlas.com/photos/0000/0504/00000504_standalone.jpg
http://www.hellenicdermatlas.com/photos/0000/0505/00000505_standalone.jpg
http://www.hellenicdermatlas.com/photos/0000/0506/00000506_standalone.jpg
http://www.hellenicdermatlas.com/photos/0000/0507/00000507_standalone.jpg
http://www.hellenicdermatlas.com/photos/0000/0508/00000508_standalone.jpg
http://www.hellenicdermatlas.com/photos/0000/0509/00000509_standalone.jpg
http://www.hellenicdermatlas.com/photos/0000/0510/00000510_standalone.jpg
http://www.hellenicdermatlas.com/photos/0000/0511/00000511_standalone.jpg
http://www.hellenicdermatlas.com/photos/0000/0512/00000512_standalone.jpg
http://www.hellenicdermatlas.com/photos/0000/0513/00000513_standalone.jpg
http://www.hellenicdermatlas.com/photos/0000/0514/00000514_standalone.jpg
http://www.hellenicdermatlas.com/photos/0000/0515/00000515_standalone.jpg
http://www.hellenicdermatlas.com/photos/0000/0517/00000517_standalone.jpg
http://www.hellenicdermatlas.com/photos/0000/0518/00000518_standalone.jpg
http://www.hellenicdermatlas.com/photos/0000/0519/00000519_standalone.jpg
http://www.hellenicdermatlas.com/photos/0000/0520/00000520_standalone.jpg
http://www.hellenicdermatlas.com/photos/0000/0521/00000521_standalone.jpg
http://www.hellenicdermatlas.com/photos/0000/0522/00000522_standalone.jpg
http://www.hellenicdermatlas.com/photos/0000/0523/00000523_standalone.jpg
http://www.hellenicdermatlas.com/photos/0000/0524/00000524_standalone.jpg
http://www.hellenicdermatlas.com/photos/0000/0525/00000525_standalone.jpg
http://www.hellenicdermatlas.com/photos/0000/0526/00000526_standalone.jpg
http://www.hellenicdermatlas.com/photos/0000/0527/00000527_standalone.jpg
http://www.hellenicdermatlas.com/photos/0000/0528/00000528_standalone.jpg
http://www.hellenicdermatlas.com/photos/0000/0529/00000529_standalone.jpg
http://www.hellenicdermatlas.com/photos/0000/0530/00000530_standalone.jpg
http://www.hellenicdermatlas.com/photos/0000/0531/00000531_standalone.jpg
http://www.hellenicdermatlas.com/photos/0000/0532/00000532_standalone.jpg
http://www.hellenicdermatlas.com/photos/0000/0533/00000533_standalone.jpg
http://www.hellenicdermatlas.com/photos/0000/0534/00000534_standalone.jpg
http://www.hellenicdermatlas.com/photos/0000/0535/00000535_standalone.jpg
http://www.hellenicdermatlas.com/photos/0000/0536/00000536_standalone.jpg
http://www.hellenicdermatlas.com/photos/0000/0537/00000537_standalone.jpg
http://www.hellenicdermatlas.com/photos/0000/0538/00000538_standalone.jpg

http://www.hellenicdermatlas.com/photos/0000/0539/00000539_standalone.jpg
http://www.hellenicdermatlas.com/photos/0000/0540/00000540_standalone.jpg
http://www.hellenicdermatlas.com/photos/0000/0541/00000541_standalone.jpg
http://www.hellenicdermatlas.com/photos/0000/0542/00000542_standalone.jpg
http://www.hellenicdermatlas.com/photos/0000/0543/00000543_standalone.jpg
http://www.hellenicdermatlas.com/photos/0000/0544/00000544_standalone.jpg
http://www.hellenicdermatlas.com/photos/0000/0545/00000545_standalone.jpg
http://www.hellenicdermatlas.com/photos/0000/0546/00000546_standalone.jpg
http://www.hellenicdermatlas.com/photos/0000/0547/00000547_standalone.jpg
http://www.hellenicdermatlas.com/photos/0000/0548/00000548_standalone.jpg
http://www.hellenicdermatlas.com/photos/0000/0549/00000549_standalone.jpg
http://www.hellenicdermatlas.com/photos/0000/0550/00000550_standalone.jpg
http://www.hellenicdermatlas.com/photos/0000/0551/00000551_standalone.jpg
http://www.hellenicdermatlas.com/photos/0000/0552/00000552_standalone.jpg
http://www.hellenicdermatlas.com/photos/0000/0553/00000553_standalone.jpg
http://www.hellenicdermatlas.com/photos/0000/0554/00000554_standalone.jpg
http://www.hellenicdermatlas.com/photos/0000/0555/00000555_standalone.jpg
http://www.hellenicdermatlas.com/photos/0000/0556/00000556_standalone.jpg
http://www.hellenicdermatlas.com/photos/0000/0557/00000557_standalone.jpg
http://www.hellenicdermatlas.com/photos/0000/0558/00000558_standalone.jpg
http://www.hellenicdermatlas.com/photos/0000/0559/00000559_standalone.jpg
http://www.hellenicdermatlas.com/photos/0000/0560/00000560_standalone.jpg
http://www.hellenicdermatlas.com/photos/0000/0561/00000561_standalone.jpg
http://www.hellenicdermatlas.com/photos/0000/0562/00000562_standalone.jpg
http://www.hellenicdermatlas.com/photos/0000/0563/00000563_standalone.jpg
http://www.hellenicdermatlas.com/photos/0000/0564/00000564_standalone.jpg
http://www.hellenicdermatlas.com/photos/0000/0565/00000565_standalone.jpg
http://www.hellenicdermatlas.com/photos/0000/0566/00000566_standalone.jpg
http://www.hellenicdermatlas.com/photos/0000/0567/00000567_standalone.jpg
http://www.hellenicdermatlas.com/photos/0000/0568/00000568_standalone.jpg
http://www.hellenicdermatlas.com/photos/0000/0569/00000569_standalone.jpg
http://www.hellenicdermatlas.com/photos/0000/0570/00000570_standalone.jpg
http://www.hellenicdermatlas.com/photos/0000/0571/00000571_standalone.jpg
http://www.hellenicdermatlas.com/photos/0000/0572/00000572_standalone.jpg
http://www.hellenicdermatlas.com/photos/0000/0573/00000573_standalone.jpg
http://www.hellenicdermatlas.com/photos/0000/0574/00000574_standalone.jpg
http://www.hellenicdermatlas.com/photos/0000/0575/00000575_standalone.jpg
http://www.hellenicdermatlas.com/photos/0000/0576/00000576_standalone.jpg
http://www.hellenicdermatlas.com/photos/0000/0577/00000577_standalone.jpg
http://www.hellenicdermatlas.com/photos/0000/0578/00000578_standalone.jpg
http://www.hellenicdermatlas.com/photos/0000/0579/00000579_standalone.jpg
http://www.hellenicdermatlas.com/photos/0000/0580/00000580_standalone.jpg
http://www.hellenicdermatlas.com/photos/0000/0581/00000581_standalone.jpg
http://www.hellenicdermatlas.com/photos/0000/0582/00000582_standalone.jpg
http://www.hellenicdermatlas.com/photos/0000/0583/00000583_standalone.jpg
http://www.hellenicdermatlas.com/photos/0000/0584/00000584_standalone.jpg
http://www.hellenicdermatlas.com/photos/0000/0585/00000585_standalone.jpg
http://www.hellenicdermatlas.com/photos/0000/0586/00000586_standalone.jpg

http://www.hellenicdermatlas.com/photos/0000/0587/00000587_standalone.jpg
http://www.hellenicdermatlas.com/photos/0000/0588/00000588_standalone.jpg
http://www.hellenicdermatlas.com/photos/0000/0589/00000589_standalone.jpg
http://www.hellenicdermatlas.com/photos/0000/0590/00000590_standalone.jpg
http://www.hellenicdermatlas.com/photos/0000/0591/00000591_standalone.jpg
http://www.hellenicdermatlas.com/photos/0000/0592/00000592_standalone.jpg
http://www.hellenicdermatlas.com/photos/0000/0593/00000593_standalone.jpg
http://www.hellenicdermatlas.com/photos/0000/0594/00000594_standalone.jpg
http://www.hellenicdermatlas.com/photos/0000/0595/00000595_standalone.jpg
http://www.hellenicdermatlas.com/photos/0000/0596/00000596_standalone.jpg
http://www.hellenicdermatlas.com/photos/0000/0597/00000597_standalone.jpg
http://www.hellenicdermatlas.com/photos/0000/0598/00000598_standalone.jpg
http://www.hellenicdermatlas.com/photos/0000/0599/00000599_standalone.jpg
http://www.hellenicdermatlas.com/photos/0000/0600/00000600_standalone.jpg
http://www.hellenicdermatlas.com/photos/0000/0601/00000601_standalone.jpg
http://www.hellenicdermatlas.com/photos/0000/0602/00000602_standalone.jpg
http://www.hellenicdermatlas.com/photos/0000/0603/00000603_standalone.jpg
http://www.hellenicdermatlas.com/photos/0000/0604/00000604_standalone.jpg
http://www.hellenicdermatlas.com/photos/0000/0605/00000605_standalone.jpg
http://www.hellenicdermatlas.com/photos/0000/0606/00000606_standalone.jpg
http://www.hellenicdermatlas.com/photos/0000/0607/00000607_standalone.jpg
http://www.hellenicdermatlas.com/photos/0000/0608/00000608_standalone.jpg
http://www.hellenicdermatlas.com/photos/0000/0609/00000609_standalone.jpg
http://www.hellenicdermatlas.com/photos/0000/0610/00000610_standalone.jpg
http://www.hellenicdermatlas.com/photos/0000/0611/00000611_standalone.jpg
http://www.hellenicdermatlas.com/photos/0000/0612/00000612_standalone.jpg
http://www.hellenicdermatlas.com/photos/0000/0613/00000613_standalone.jpg
http://www.hellenicdermatlas.com/photos/0000/0614/00000614_standalone.jpg
http://www.hellenicdermatlas.com/photos/0000/0615/00000615_standalone.jpg
http://www.hellenicdermatlas.com/photos/0000/0616/00000616_standalone.jpg
http://www.hellenicdermatlas.com/photos/0000/0617/00000617_standalone.jpg
http://www.hellenicdermatlas.com/photos/0000/0618/00000618_standalone.jpg
http://www.hellenicdermatlas.com/photos/0000/0619/00000619_standalone.jpg
http://www.hellenicdermatlas.com/photos/0000/0620/00000620_standalone.jpg
http://www.hellenicdermatlas.com/photos/0000/0621/00000621_standalone.jpg
http://www.hellenicdermatlas.com/photos/0000/0622/00000622_standalone.jpg
http://www.hellenicdermatlas.com/photos/0000/0623/00000623_standalone.jpg
http://www.hellenicdermatlas.com/photos/0000/0624/00000624_standalone.jpg
http://www.hellenicdermatlas.com/photos/0000/0625/00000625_standalone.jpg
http://www.hellenicdermatlas.com/photos/0000/0626/00000626_standalone.jpg
http://www.hellenicdermatlas.com/photos/0000/0627/00000627_standalone.jpg
http://www.hellenicdermatlas.com/photos/0000/0628/00000628_standalone.jpg
http://www.hellenicdermatlas.com/photos/0000/0629/00000629_standalone.jpg
http://www.hellenicdermatlas.com/photos/0000/0630/00000630_standalone.jpg
http://www.hellenicdermatlas.com/photos/0000/0631/00000631_standalone.jpg
http://www.hellenicdermatlas.com/photos/0000/0632/00000632_standalone.jpg
http://www.hellenicdermatlas.com/photos/0000/0633/00000633_standalone.jpg
http://www.hellenicdermatlas.com/photos/0000/0634/00000634_standalone.jpg

http://www.hellenicdermatlas.com/photos/0000/0635/00000635_standalone.jpg
http://www.hellenicdermatlas.com/photos/0000/0636/00000636_standalone.jpg
http://www.hellenicdermatlas.com/photos/0000/0637/00000637_standalone.jpg
http://www.hellenicdermatlas.com/photos/0000/0638/00000638_standalone.jpg
http://www.hellenicdermatlas.com/photos/0000/0639/00000639_standalone.jpg
http://www.hellenicdermatlas.com/photos/0000/0640/00000640_standalone.jpg
http://www.hellenicdermatlas.com/photos/0000/0641/00000641_standalone.jpg
http://www.hellenicdermatlas.com/photos/0000/0642/00000642_standalone.jpg
http://www.hellenicdermatlas.com/photos/0000/0643/00000643_standalone.jpg
http://www.hellenicdermatlas.com/photos/0000/0644/00000644_standalone.jpg
http://www.hellenicdermatlas.com/photos/0000/0645/00000645_standalone.jpg
http://www.hellenicdermatlas.com/photos/0000/0646/00000646_standalone.jpg
http://www.hellenicdermatlas.com/photos/0000/0647/00000647_standalone.jpg
http://www.hellenicdermatlas.com/photos/0000/0648/00000648_standalone.jpg
http://www.hellenicdermatlas.com/photos/0000/0649/00000649_standalone.jpg
http://www.hellenicdermatlas.com/photos/0000/0650/00000650_standalone.jpg
http://www.hellenicdermatlas.com/photos/0000/0651/00000651_standalone.jpg
http://www.hellenicdermatlas.com/photos/0000/0652/00000652_standalone.jpg
http://www.hellenicdermatlas.com/photos/0000/0653/00000653_standalone.jpg
http://www.hellenicdermatlas.com/photos/0000/0654/00000654_standalone.jpg
http://www.hellenicdermatlas.com/photos/0000/0655/00000655_standalone.jpg
http://www.hellenicdermatlas.com/photos/0000/0656/00000656_standalone.jpg
http://www.hellenicdermatlas.com/photos/0000/0657/00000657_standalone.jpg
http://www.hellenicdermatlas.com/photos/0000/0658/00000658_standalone.jpg
http://www.hellenicdermatlas.com/photos/0000/0659/00000659_standalone.jpg
http://www.hellenicdermatlas.com/photos/0000/0660/00000660_standalone.jpg
http://www.hellenicdermatlas.com/photos/0000/0661/00000661_standalone.jpg
http://www.hellenicdermatlas.com/photos/0000/0662/00000662_standalone.jpg
http://www.hellenicdermatlas.com/photos/0000/0663/00000663_standalone.jpg
http://www.hellenicdermatlas.com/photos/0000/0664/00000664_standalone.jpg
http://www.hellenicdermatlas.com/photos/0000/0665/00000665_standalone.jpg
http://www.hellenicdermatlas.com/photos/0000/0666/00000666_standalone.jpg
http://www.hellenicdermatlas.com/photos/0000/0667/00000667_standalone.jpg
http://www.hellenicdermatlas.com/photos/0000/0668/00000668_standalone.jpg
http://www.hellenicdermatlas.com/photos/0000/0669/00000669_standalone.jpg
http://www.hellenicdermatlas.com/photos/0000/0670/00000670_standalone.jpg
http://www.hellenicdermatlas.com/photos/0000/0671/00000671_standalone.jpg
http://www.hellenicdermatlas.com/photos/0000/0672/00000672_standalone.jpg
http://www.hellenicdermatlas.com/photos/0000/0673/00000673_standalone.jpg
http://www.hellenicdermatlas.com/photos/0000/0674/00000674_standalone.jpg
http://www.hellenicdermatlas.com/photos/0000/0675/00000675_standalone.jpg
http://www.hellenicdermatlas.com/photos/0000/0676/00000676_standalone.jpg
http://www.hellenicdermatlas.com/photos/0000/0677/00000677_standalone.jpg
http://www.hellenicdermatlas.com/photos/0000/0678/00000678_standalone.jpg
http://www.hellenicdermatlas.com/photos/0000/0679/00000679_standalone.jpg
http://www.hellenicdermatlas.com/photos/0000/0680/00000680_standalone.jpg
http://www.hellenicdermatlas.com/photos/0000/0681/00000681_standalone.jpg
http://www.hellenicdermatlas.com/photos/0000/0682/00000682_standalone.jpg

http://www.hellenicdermatlas.com/photos/0000/0683/00000683_standalone.jpg
http://www.hellenicdermatlas.com/photos/0000/0684/00000684_standalone.jpg
http://www.hellenicdermatlas.com/photos/0000/0685/00000685_standalone.jpg
http://www.hellenicdermatlas.com/photos/0000/0686/00000686_standalone.jpg
http://www.hellenicdermatlas.com/photos/0000/0687/00000687_standalone.jpg
http://www.hellenicdermatlas.com/photos/0000/0688/00000688_standalone.jpg
http://www.hellenicdermatlas.com/photos/0000/0689/00000689_standalone.jpg
http://www.hellenicdermatlas.com/photos/0000/0690/00000690_standalone.jpg
http://www.hellenicdermatlas.com/photos/0000/0691/00000691_standalone.jpg
http://www.hellenicdermatlas.com/photos/0000/0692/00000692_standalone.jpg
http://www.hellenicdermatlas.com/photos/0000/0693/00000693_standalone.jpg
http://www.hellenicdermatlas.com/photos/0000/0694/00000694_standalone.jpg
http://www.hellenicdermatlas.com/photos/0000/0695/00000695_standalone.jpg
http://www.hellenicdermatlas.com/photos/0000/0696/00000696_standalone.jpg
http://www.hellenicdermatlas.com/photos/0000/0697/00000697_standalone.jpg
http://www.hellenicdermatlas.com/photos/0000/0698/00000698_standalone.jpg
http://www.hellenicdermatlas.com/photos/0000/0699/00000699_standalone.jpg
http://www.hellenicdermatlas.com/photos/0000/0700/00000700_standalone.jpg
http://www.hellenicdermatlas.com/photos/0000/0701/00000701_standalone.jpg
http://www.hellenicdermatlas.com/photos/0000/0702/00000702_standalone.jpg
http://www.hellenicdermatlas.com/photos/0000/0703/00000703_standalone.jpg
http://www.hellenicdermatlas.com/photos/0000/0704/00000704_standalone.jpg
http://www.hellenicdermatlas.com/photos/0000/0705/00000705_standalone.jpg
http://www.hellenicdermatlas.com/photos/0000/0706/00000706_standalone.jpg
http://www.hellenicdermatlas.com/photos/0000/0707/00000707_standalone.jpg
http://www.hellenicdermatlas.com/photos/0000/0708/00000708_standalone.jpg
http://www.hellenicdermatlas.com/photos/0000/0709/00000709_standalone.jpg
http://www.hellenicdermatlas.com/photos/0000/0710/00000710_standalone.jpg
http://www.hellenicdermatlas.com/photos/0000/0711/00000711_standalone.jpg
http://www.hellenicdermatlas.com/photos/0000/0712/00000712_standalone.jpg
http://www.hellenicdermatlas.com/photos/0000/0713/00000713_standalone.jpg
http://www.hellenicdermatlas.com/photos/0000/0714/00000714_standalone.jpg
http://www.hellenicdermatlas.com/photos/0000/0715/00000715_standalone.jpg
http://www.hellenicdermatlas.com/photos/0000/0716/00000716_standalone.jpg
http://www.hellenicdermatlas.com/photos/0000/0717/00000717_standalone.jpg
http://www.hellenicdermatlas.com/photos/0000/0718/00000718_standalone.jpg
http://www.hellenicdermatlas.com/photos/0000/0719/00000719_standalone.jpg
http://www.hellenicdermatlas.com/photos/0000/0720/00000720_standalone.jpg
http://www.hellenicdermatlas.com/photos/0000/0721/00000721_standalone.jpg
http://www.hellenicdermatlas.com/photos/0000/0722/00000722_standalone.jpg
http://www.hellenicdermatlas.com/photos/0000/0723/00000723_standalone.jpg
http://www.hellenicdermatlas.com/photos/0000/0724/00000724_standalone.jpg
http://www.hellenicdermatlas.com/photos/0000/0725/00000725_standalone.jpg
http://www.hellenicdermatlas.com/photos/0000/0726/00000726_standalone.jpg
http://www.hellenicdermatlas.com/photos/0000/0727/00000727_standalone.jpg
http://www.hellenicdermatlas.com/photos/0000/0728/00000728_standalone.jpg
http://www.hellenicdermatlas.com/photos/0000/0729/00000729_standalone.jpg
http://www.hellenicdermatlas.com/photos/0000/0730/00000730_standalone.jpg

http://www.hellenicdermatlas.com/photos/0000/0731/00000731_standalone.jpg
http://www.hellenicdermatlas.com/photos/0000/0732/00000732_standalone.jpg
http://www.hellenicdermatlas.com/photos/0000/0733/00000733_standalone.jpg
http://www.hellenicdermatlas.com/photos/0000/0734/00000734_standalone.jpg
http://www.hellenicdermatlas.com/photos/0000/0735/00000735_standalone.jpg
http://www.hellenicdermatlas.com/photos/0000/0736/00000736_standalone.jpg
http://www.hellenicdermatlas.com/photos/0000/0737/00000737_standalone.jpg
http://www.hellenicdermatlas.com/photos/0000/0738/00000738_standalone.jpg
http://www.hellenicdermatlas.com/photos/0000/0739/00000739_standalone.jpg
http://www.hellenicdermatlas.com/photos/0000/0740/00000740_standalone.jpg
http://www.hellenicdermatlas.com/photos/0000/0741/00000741_standalone.jpg
http://www.hellenicdermatlas.com/photos/0000/0742/00000742_standalone.jpg
http://www.hellenicdermatlas.com/photos/0000/0743/00000743_standalone.jpg
http://www.hellenicdermatlas.com/photos/0000/0744/00000744_standalone.jpg
http://www.hellenicdermatlas.com/photos/0000/0745/00000745_standalone.jpg
http://www.hellenicdermatlas.com/photos/0000/0746/00000746_standalone.jpg
http://www.hellenicdermatlas.com/photos/0000/0747/00000747_standalone.jpg
http://www.hellenicdermatlas.com/photos/0000/0748/00000748_standalone.jpg
http://www.hellenicdermatlas.com/photos/0000/0749/00000749_standalone.jpg
http://www.hellenicdermatlas.com/photos/0000/0750/00000750_standalone.jpg
http://www.hellenicdermatlas.com/photos/0000/0751/00000751_standalone.jpg
http://www.hellenicdermatlas.com/photos/0000/0752/00000752_standalone.jpg
http://www.hellenicdermatlas.com/photos/0000/0753/00000753_standalone.jpg
http://www.hellenicdermatlas.com/photos/0000/0754/00000754_standalone.jpg
http://www.hellenicdermatlas.com/photos/0000/0755/00000755_standalone.jpg
http://www.hellenicdermatlas.com/photos/0000/0756/00000756_standalone.jpg
http://www.hellenicdermatlas.com/photos/0000/0757/00000757_standalone.jpg
http://www.hellenicdermatlas.com/photos/0000/0758/00000758_standalone.jpg
http://www.hellenicdermatlas.com/photos/0000/0759/00000759_standalone.jpg
http://www.hellenicdermatlas.com/photos/0000/0760/00000760_standalone.jpg
http://www.hellenicdermatlas.com/photos/0000/0761/00000761_standalone.jpg
http://www.hellenicdermatlas.com/photos/0000/0762/00000762_standalone.jpg
http://www.hellenicdermatlas.com/photos/0000/0763/00000763_standalone.jpg
http://www.hellenicdermatlas.com/photos/0000/0764/00000764_standalone.jpg
http://www.hellenicdermatlas.com/photos/0000/0765/00000765_standalone.jpg
http://www.hellenicdermatlas.com/photos/0000/0766/00000766_standalone.jpg
http://www.hellenicdermatlas.com/photos/0000/0767/00000767_standalone.jpg
http://www.hellenicdermatlas.com/photos/0000/0768/00000768_standalone.jpg
http://www.hellenicdermatlas.com/photos/0000/0769/00000769_standalone.jpg
http://www.hellenicdermatlas.com/photos/0000/0770/00000770_standalone.jpg
http://www.hellenicdermatlas.com/photos/0000/0771/00000771_standalone.jpg
http://www.hellenicdermatlas.com/photos/0000/0772/00000772_standalone.jpg
http://www.hellenicdermatlas.com/photos/0000/0773/00000773_standalone.jpg
http://www.hellenicdermatlas.com/photos/0000/0774/00000774_standalone.jpg
http://www.hellenicdermatlas.com/photos/0000/0775/00000775_standalone.jpg
http://www.hellenicdermatlas.com/photos/0000/0776/00000776_standalone.jpg
http://www.hellenicdermatlas.com/photos/0000/0777/00000777_standalone.jpg
http://www.hellenicdermatlas.com/photos/0000/0778/00000778_standalone.jpg

http://www.hellenicdermatlas.com/photos/0000/0779/00000779_standalone.jpg
http://www.hellenicdermatlas.com/photos/0000/0780/00000780_standalone.jpg
http://www.hellenicdermatlas.com/photos/0000/0781/00000781_standalone.jpg
http://www.hellenicdermatlas.com/photos/0000/0782/00000782_standalone.jpg
http://www.hellenicdermatlas.com/photos/0000/0783/00000783_standalone.jpg
http://www.hellenicdermatlas.com/photos/0000/0784/00000784_standalone.jpg
http://www.hellenicdermatlas.com/photos/0000/0785/00000785_standalone.jpg
http://www.hellenicdermatlas.com/photos/0000/0786/00000786_standalone.jpg
http://www.hellenicdermatlas.com/photos/0000/0787/00000787_standalone.jpg
http://www.hellenicdermatlas.com/photos/0000/0788/00000788_standalone.jpg
http://www.hellenicdermatlas.com/photos/0000/0789/00000789_standalone.jpg
http://www.hellenicdermatlas.com/photos/0000/0790/00000790_standalone.jpg
http://www.hellenicdermatlas.com/photos/0000/0791/00000791_standalone.jpg
http://www.hellenicdermatlas.com/photos/0000/0792/00000792_standalone.jpg
http://www.hellenicdermatlas.com/photos/0000/0793/00000793_standalone.jpg
http://www.hellenicdermatlas.com/photos/0000/0794/00000794_standalone.jpg
http://www.hellenicdermatlas.com/photos/0000/0795/00000795_standalone.jpg
http://www.hellenicdermatlas.com/photos/0000/0796/00000796_standalone.jpg
http://www.hellenicdermatlas.com/photos/0000/0797/00000797_standalone.jpg
http://www.hellenicdermatlas.com/photos/0000/0798/00000798_standalone.jpg
http://www.hellenicdermatlas.com/photos/0000/0799/00000799_standalone.jpg
http://www.hellenicdermatlas.com/photos/0000/0800/00000800_standalone.jpg
http://www.hellenicdermatlas.com/photos/0000/0801/00000801_standalone.jpg
http://www.hellenicdermatlas.com/photos/0000/0802/00000802_standalone.jpg
http://www.hellenicdermatlas.com/photos/0000/0803/00000803_standalone.jpg
http://www.hellenicdermatlas.com/photos/0000/0804/00000804_standalone.jpg
http://www.hellenicdermatlas.com/photos/0000/0805/00000805_standalone.jpg
http://www.hellenicdermatlas.com/photos/0000/0806/00000806_standalone.jpg
http://www.hellenicdermatlas.com/photos/0000/0807/00000807_standalone.jpg
http://www.hellenicdermatlas.com/photos/0000/0808/00000808_standalone.jpg
http://www.hellenicdermatlas.com/photos/0000/0809/00000809_standalone.jpg
http://www.hellenicdermatlas.com/photos/0000/0810/00000810_standalone.jpg
http://www.hellenicdermatlas.com/photos/0000/0811/00000811_standalone.jpg
http://www.hellenicdermatlas.com/photos/0000/0812/00000812_standalone.jpg
http://www.hellenicdermatlas.com/photos/0000/0813/00000813_standalone.jpg
http://www.hellenicdermatlas.com/photos/0000/0814/00000814_standalone.jpg
http://www.hellenicdermatlas.com/photos/0000/0815/00000815_standalone.jpg
http://www.hellenicdermatlas.com/photos/0000/0816/00000816_standalone.jpg
http://www.hellenicdermatlas.com/photos/0000/0817/00000817_standalone.jpg
http://www.hellenicdermatlas.com/photos/0000/0818/00000818_standalone.jpg
http://www.hellenicdermatlas.com/photos/0000/0819/00000819_standalone.jpg
http://www.hellenicdermatlas.com/photos/0000/0820/00000820_standalone.jpg
http://www.hellenicdermatlas.com/photos/0000/0821/00000821_standalone.jpg
http://www.hellenicdermatlas.com/photos/0000/0822/00000822_standalone.jpg
http://www.hellenicdermatlas.com/photos/0000/0823/00000823_standalone.jpg
http://www.hellenicdermatlas.com/photos/0000/0824/00000824_standalone.jpg
http://www.hellenicdermatlas.com/photos/0000/0825/00000825_standalone.jpg
http://www.hellenicdermatlas.com/photos/0000/0826/00000826_standalone.jpg

http://www.hellenicdermatlas.com/photos/0000/0827/00000827_standalone.jpg
http://www.hellenicdermatlas.com/photos/0000/0828/00000828_standalone.jpg
http://www.hellenicdermatlas.com/photos/0000/0829/00000829_standalone.jpg
http://www.hellenicdermatlas.com/photos/0000/0830/00000830_standalone.jpg
http://www.hellenicdermatlas.com/photos/0000/0831/00000831_standalone.jpg
http://www.hellenicdermatlas.com/photos/0000/0832/00000832_standalone.jpg
http://www.hellenicdermatlas.com/photos/0000/0833/00000833_standalone.jpg
http://www.hellenicdermatlas.com/photos/0000/0834/00000834_standalone.jpg
http://www.hellenicdermatlas.com/photos/0000/0835/00000835_standalone.jpg
http://www.hellenicdermatlas.com/photos/0000/0836/00000836_standalone.jpg
http://www.hellenicdermatlas.com/photos/0000/0837/00000837_standalone.jpg
http://www.hellenicdermatlas.com/photos/0000/0838/00000838_standalone.jpg
http://www.hellenicdermatlas.com/photos/0000/0839/00000839_standalone.jpg
http://www.hellenicdermatlas.com/photos/0000/0840/00000840_standalone.jpg
http://www.hellenicdermatlas.com/photos/0000/0841/00000841_standalone.jpg
http://www.hellenicdermatlas.com/photos/0000/0842/00000842_standalone.jpg
http://www.hellenicdermatlas.com/photos/0000/0843/00000843_standalone.jpg
http://www.hellenicdermatlas.com/photos/0000/0844/00000844_standalone.jpg
http://www.hellenicdermatlas.com/photos/0000/0845/00000845_standalone.jpg
http://www.hellenicdermatlas.com/photos/0000/0846/00000846_standalone.jpg
http://www.hellenicdermatlas.com/photos/0000/0847/00000847_standalone.jpg
http://www.hellenicdermatlas.com/photos/0000/0848/00000848_standalone.jpg
http://www.hellenicdermatlas.com/photos/0000/0849/00000849_standalone.jpg
http://www.hellenicdermatlas.com/photos/0000/0850/00000850_standalone.jpg
http://www.hellenicdermatlas.com/photos/0000/0851/00000851_standalone.jpg
http://www.hellenicdermatlas.com/photos/0000/0852/00000852_standalone.jpg
http://www.hellenicdermatlas.com/photos/0000/0853/00000853_standalone.jpg
http://www.hellenicdermatlas.com/photos/0000/0854/00000854_standalone.jpg
http://www.hellenicdermatlas.com/photos/0000/0855/00000855_standalone.jpg
http://www.hellenicdermatlas.com/photos/0000/0856/00000856_standalone.jpg
http://www.hellenicdermatlas.com/photos/0000/0857/00000857_standalone.jpg
http://www.hellenicdermatlas.com/photos/0000/0858/00000858_standalone.jpg
http://www.hellenicdermatlas.com/photos/0000/0859/00000859_standalone.jpg
http://www.hellenicdermatlas.com/photos/0000/0860/00000860_standalone.jpg
http://www.hellenicdermatlas.com/photos/0000/0861/00000861_standalone.jpg
http://www.hellenicdermatlas.com/photos/0000/0862/00000862_standalone.jpg
http://www.hellenicdermatlas.com/photos/0000/0863/00000863_standalone.jpg
http://www.hellenicdermatlas.com/photos/0000/0864/00000864_standalone.jpg
http://www.hellenicdermatlas.com/photos/0000/0865/00000865_standalone.jpg
http://www.hellenicdermatlas.com/photos/0000/0866/00000866_standalone.jpg
http://www.hellenicdermatlas.com/photos/0000/0867/00000867_standalone.jpg
http://www.hellenicdermatlas.com/photos/0000/0868/00000868_standalone.jpg
http://www.hellenicdermatlas.com/photos/0000/0869/00000869_standalone.jpg
http://www.hellenicdermatlas.com/photos/0000/0870/00000870_standalone.jpg
http://www.hellenicdermatlas.com/photos/0000/0871/00000871_standalone.jpg
http://www.hellenicdermatlas.com/photos/0000/0872/00000872_standalone.jpg
http://www.hellenicdermatlas.com/photos/0000/0873/00000873_standalone.jpg
http://www.hellenicdermatlas.com/photos/0000/0874/00000874_standalone.jpg

http://www.hellenicdermatlas.com/photos/0000/0875/00000875_standalone.jpg
http://www.hellenicdermatlas.com/photos/0000/0876/00000876_standalone.jpg
http://www.hellenicdermatlas.com/photos/0000/0877/00000877_standalone.jpg
http://www.hellenicdermatlas.com/photos/0000/0878/00000878_standalone.jpg
http://www.hellenicdermatlas.com/photos/0000/0879/00000879_standalone.jpg
http://www.hellenicdermatlas.com/photos/0000/0880/00000880_standalone.jpg
http://www.hellenicdermatlas.com/photos/0000/0881/00000881_standalone.jpg
http://www.hellenicdermatlas.com/photos/0000/0882/00000882_standalone.jpg
http://www.hellenicdermatlas.com/photos/0000/0883/00000883_standalone.jpg
http://www.hellenicdermatlas.com/photos/0000/0884/00000884_standalone.jpg
http://www.hellenicdermatlas.com/photos/0000/0885/00000885_standalone.jpg
http://www.hellenicdermatlas.com/photos/0000/0886/00000886_standalone.jpg
http://www.hellenicdermatlas.com/photos/0000/0887/00000887_standalone.jpg
http://www.hellenicdermatlas.com/photos/0000/0888/00000888_standalone.jpg
http://www.hellenicdermatlas.com/photos/0000/0889/00000889_standalone.jpg
http://www.hellenicdermatlas.com/photos/0000/0890/00000890_standalone.jpg
http://www.hellenicdermatlas.com/photos/0000/0891/00000891_standalone.jpg
http://www.hellenicdermatlas.com/photos/0000/0892/00000892_standalone.jpg
http://www.hellenicdermatlas.com/photos/0000/0893/00000893_standalone.jpg
http://www.hellenicdermatlas.com/photos/0000/0894/00000894_standalone.jpg
http://www.hellenicdermatlas.com/photos/0000/0895/00000895_standalone.jpg
http://www.hellenicdermatlas.com/photos/0000/0896/00000896_standalone.jpg
http://www.hellenicdermatlas.com/photos/0000/0897/00000897_standalone.jpg
http://www.hellenicdermatlas.com/photos/0000/0898/00000898_standalone.jpg
http://www.hellenicdermatlas.com/photos/0000/0899/00000899_standalone.jpg
http://www.hellenicdermatlas.com/photos/0000/0900/00000900_standalone.jpg
http://www.hellenicdermatlas.com/photos/0000/0901/00000901_standalone.jpg
http://www.hellenicdermatlas.com/photos/0000/0902/00000902_standalone.jpg
http://www.hellenicdermatlas.com/photos/0000/0903/00000903_standalone.jpg
http://www.hellenicdermatlas.com/photos/0000/0904/00000904_standalone.jpg
http://www.hellenicdermatlas.com/photos/0000/0905/00000905_standalone.jpg
http://www.hellenicdermatlas.com/photos/0000/0906/00000906_standalone.jpg
http://www.hellenicdermatlas.com/photos/0000/0907/00000907_standalone.jpg
http://www.hellenicdermatlas.com/photos/0000/0908/00000908_standalone.jpg
http://www.hellenicdermatlas.com/photos/0000/0909/00000909_standalone.jpg
http://www.hellenicdermatlas.com/photos/0000/0910/00000910_standalone.jpg
http://www.hellenicdermatlas.com/photos/0000/0911/00000911_standalone.jpg
http://www.hellenicdermatlas.com/photos/0000/0912/00000912_standalone.jpg
http://www.hellenicdermatlas.com/photos/0000/0913/00000913_standalone.jpg
http://www.hellenicdermatlas.com/photos/0000/0914/00000914_standalone.jpg
http://www.hellenicdermatlas.com/photos/0000/0915/00000915_standalone.jpg
http://www.hellenicdermatlas.com/photos/0000/0916/00000916_standalone.jpg
http://www.hellenicdermatlas.com/photos/0000/0917/00000917_standalone.jpg
http://www.hellenicdermatlas.com/photos/0000/0918/00000918_standalone.jpg
http://www.hellenicdermatlas.com/photos/0000/0919/00000919_standalone.jpg
http://www.hellenicdermatlas.com/photos/0000/0920/00000920_standalone.jpg
http://www.hellenicdermatlas.com/photos/0000/0921/00000921_standalone.jpg
http://www.hellenicdermatlas.com/photos/0000/0922/00000922_standalone.jpg

http://www.hellenicdermatlas.com/photos/0000/0923/00000923_standalone.jpg
http://www.hellenicdermatlas.com/photos/0000/0924/00000924_standalone.jpg
http://www.hellenicdermatlas.com/photos/0000/0925/00000925_standalone.jpg
http://www.hellenicdermatlas.com/photos/0000/0926/00000926_standalone.jpg
http://www.hellenicdermatlas.com/photos/0000/0927/00000927_standalone.jpg
http://www.hellenicdermatlas.com/photos/0000/0928/00000928_standalone.jpg
http://www.hellenicdermatlas.com/photos/0000/0929/00000929_standalone.jpg
http://www.hellenicdermatlas.com/photos/0000/0930/00000930_standalone.jpg
http://www.hellenicdermatlas.com/photos/0000/0931/00000931_standalone.jpg
http://www.hellenicdermatlas.com/photos/0000/0932/00000932_standalone.jpg
http://www.hellenicdermatlas.com/photos/0000/0933/00000933_standalone.jpg
http://www.hellenicdermatlas.com/photos/0000/0934/00000934_standalone.jpg
http://www.hellenicdermatlas.com/photos/0000/0935/00000935_standalone.jpg
http://www.hellenicdermatlas.com/photos/0000/0936/00000936_standalone.jpg
http://www.hellenicdermatlas.com/photos/0000/0937/00000937_standalone.jpg
http://www.hellenicdermatlas.com/photos/0000/0938/00000938_standalone.jpg
http://www.hellenicdermatlas.com/photos/0000/0939/00000939_standalone.jpg
http://www.hellenicdermatlas.com/photos/0000/0940/00000940_standalone.jpg
http://www.hellenicdermatlas.com/photos/0000/0941/00000941_standalone.jpg
http://www.hellenicdermatlas.com/photos/0000/0942/00000942_standalone.jpg
http://www.hellenicdermatlas.com/photos/0000/0943/00000943_standalone.jpg
http://www.hellenicdermatlas.com/photos/0000/0944/00000944_standalone.jpg
http://www.hellenicdermatlas.com/photos/0000/0945/00000945_standalone.jpg
http://www.hellenicdermatlas.com/photos/0000/0946/00000946_standalone.jpg
http://www.hellenicdermatlas.com/photos/0000/0947/00000947_standalone.jpg
http://www.hellenicdermatlas.com/photos/0000/0948/00000948_standalone.jpg
http://www.hellenicdermatlas.com/photos/0000/0949/00000949_standalone.jpg
http://www.hellenicdermatlas.com/photos/0000/0950/00000950_standalone.jpg
http://www.hellenicdermatlas.com/photos/0000/0951/00000951_standalone.jpg
http://www.hellenicdermatlas.com/photos/0000/0952/00000952_standalone.jpg
http://www.hellenicdermatlas.com/photos/0000/0953/00000953_standalone.jpg
http://www.hellenicdermatlas.com/photos/0000/0954/00000954_standalone.jpg
http://www.hellenicdermatlas.com/photos/0000/0955/00000955_standalone.jpg
http://www.hellenicdermatlas.com/photos/0000/0956/00000956_standalone.jpg
http://www.hellenicdermatlas.com/photos/0000/0957/00000957_standalone.jpg
http://www.hellenicdermatlas.com/photos/0000/0958/00000958_standalone.jpg
http://www.hellenicdermatlas.com/photos/0000/0959/00000959_standalone.jpg
http://www.hellenicdermatlas.com/photos/0000/0960/00000960_standalone.jpg
http://www.hellenicdermatlas.com/photos/0000/0961/00000961_standalone.jpg
http://www.hellenicdermatlas.com/photos/0000/0962/00000962_standalone.jpg
http://www.hellenicdermatlas.com/photos/0000/0963/00000963_standalone.jpg
http://www.hellenicdermatlas.com/photos/0000/0964/00000964_standalone.jpg
http://www.hellenicdermatlas.com/photos/0000/0965/00000965_standalone.jpg
http://www.hellenicdermatlas.com/photos/0000/0966/00000966_standalone.jpg
http://www.hellenicdermatlas.com/photos/0000/0967/00000967_standalone.jpg
http://www.hellenicdermatlas.com/photos/0000/0968/00000968_standalone.jpg
http://www.hellenicdermatlas.com/photos/0000/0969/00000969_standalone.jpg
http://www.hellenicdermatlas.com/photos/0000/0970/00000970_standalone.jpg

http://www.hellenicdermatlas.com/photos/0000/0971/00000971_standalone.jpg
http://www.hellenicdermatlas.com/photos/0000/0972/00000972_standalone.jpg
http://www.hellenicdermatlas.com/photos/0000/0973/00000973_standalone.jpg
http://www.hellenicdermatlas.com/photos/0000/0974/00000974_standalone.jpg
http://www.hellenicdermatlas.com/photos/0000/0975/00000975_standalone.jpg
http://www.hellenicdermatlas.com/photos/0000/0976/00000976_standalone.jpg
http://www.hellenicdermatlas.com/photos/0000/0977/00000977_standalone.jpg
http://www.hellenicdermatlas.com/photos/0000/0978/00000978_standalone.jpg
http://www.hellenicdermatlas.com/photos/0000/0979/00000979_standalone.jpg
http://www.hellenicdermatlas.com/photos/0000/0980/00000980_standalone.jpg
http://www.hellenicdermatlas.com/photos/0000/0981/00000981_standalone.jpg
http://www.hellenicdermatlas.com/photos/0000/0982/00000982_standalone.jpg
http://www.hellenicdermatlas.com/photos/0000/0983/00000983_standalone.jpg
http://www.hellenicdermatlas.com/photos/0000/0984/00000984_standalone.jpg
http://www.hellenicdermatlas.com/photos/0000/0985/00000985_standalone.jpg
http://www.hellenicdermatlas.com/photos/0000/0986/00000986_standalone.jpg
http://www.hellenicdermatlas.com/photos/0000/0987/00000987_standalone.jpg
http://www.hellenicdermatlas.com/photos/0000/0988/00000988_standalone.jpg
http://www.hellenicdermatlas.com/photos/0000/0989/00000989_standalone.jpg
http://www.hellenicdermatlas.com/photos/0000/0990/00000990_standalone.jpg
http://www.hellenicdermatlas.com/photos/0000/0991/00000991_standalone.jpg
http://www.hellenicdermatlas.com/photos/0000/0992/00000992_standalone.jpg
http://www.hellenicdermatlas.com/photos/0000/0993/00000993_standalone.jpg
http://www.hellenicdermatlas.com/photos/0000/0994/00000994_standalone.jpg
http://www.hellenicdermatlas.com/photos/0000/0995/00000995_standalone.jpg
http://www.hellenicdermatlas.com/photos/0000/0996/00000996_standalone.jpg
http://www.hellenicdermatlas.com/photos/0000/0998/00000998_standalone.jpg
http://www.hellenicdermatlas.com/photos/0000/0999/00000999_standalone.jpg
http://www.hellenicdermatlas.com/photos/000/01000/00001000_standalone.jpg
http://www.hellenicdermatlas.com/photos/000/01001/00001001_standalone.jpg
http://www.hellenicdermatlas.com/photos/000/01002/00001002_standalone.jpg
http://www.hellenicdermatlas.com/photos/0000/1003/00001003_standalone.jpg
http://www.hellenicdermatlas.com/photos/0000/1004/00001004_standalone.jpg
http://www.hellenicdermatlas.com/photos/0000/1005/00001005_standalone.jpg
http://www.hellenicdermatlas.com/photos/0000/1006/00001006_standalone.jpg
http://www.hellenicdermatlas.com/photos/0000/1007/00001007_standalone.jpg
http://www.hellenicdermatlas.com/photos/0000/1008/00001008_standalone.jpg
http://www.hellenicdermatlas.com/photos/0000/1009/00001009_standalone.jpg
http://www.hellenicdermatlas.com/photos/0000/1010/00001010_standalone.jpg
http://www.hellenicdermatlas.com/photos/0000/1011/00001011_standalone.jpg
http://www.hellenicdermatlas.com/photos/0000/1012/00001012_standalone.jpg
http://www.hellenicdermatlas.com/photos/0000/1013/00001013_standalone.jpg
http://www.hellenicdermatlas.com/photos/0000/1014/00001014_standalone.jpg
http://www.hellenicdermatlas.com/photos/0000/1015/00001015_standalone.jpg
http://www.hellenicdermatlas.com/photos/0000/1016/00001016_standalone.jpg
http://www.hellenicdermatlas.com/photos/0000/1017/00001017_standalone.jpg
http://www.hellenicdermatlas.com/photos/0000/1018/00001018_standalone.jpg
http://www.hellenicdermatlas.com/photos/0000/1019/00001019_standalone.jpg

http://www.hellenicdermatlas.com/photos/0000/1020/00001020_standalone.jpg
http://www.hellenicdermatlas.com/photos/0000/1021/00001021_standalone.jpg
http://www.hellenicdermatlas.com/photos/0000/1022/00001022_standalone.jpg
http://www.hellenicdermatlas.com/photos/0000/1023/00001023_standalone.jpg
http://www.hellenicdermatlas.com/photos/0000/1024/00001024_standalone.jpg
http://www.hellenicdermatlas.com/photos/0000/1025/00001025_standalone.jpg
http://www.hellenicdermatlas.com/photos/0000/1026/00001026_standalone.jpg
http://www.hellenicdermatlas.com/photos/0000/1027/00001027_standalone.jpg
http://www.hellenicdermatlas.com/photos/0000/1028/00001028_standalone.jpg
http://www.hellenicdermatlas.com/photos/0000/1029/00001029_standalone.jpg
http://www.hellenicdermatlas.com/photos/0000/1030/00001030_standalone.jpg
http://www.hellenicdermatlas.com/photos/0000/1031/00001031_standalone.jpg
http://www.hellenicdermatlas.com/photos/0000/1032/00001032_standalone.jpg
http://www.hellenicdermatlas.com/photos/0000/1033/00001033_standalone.jpg
http://www.hellenicdermatlas.com/photos/0000/1034/00001034_standalone.jpg
http://www.hellenicdermatlas.com/photos/0000/1035/00001035_standalone.jpg
http://www.hellenicdermatlas.com/photos/0000/1036/00001036_standalone.jpg
http://www.hellenicdermatlas.com/photos/0000/1037/00001037_standalone.jpg
http://www.hellenicdermatlas.com/photos/0000/1038/00001038_standalone.jpg
http://www.hellenicdermatlas.com/photos/0000/1039/00001039_standalone.jpg
http://www.hellenicdermatlas.com/photos/0000/1040/00001040_standalone.jpg
http://www.hellenicdermatlas.com/photos/0000/1041/00001041_standalone.jpg
http://www.hellenicdermatlas.com/photos/0000/1042/00001042_standalone.jpg
http://www.hellenicdermatlas.com/photos/0000/1043/00001043_standalone.jpg
http://www.hellenicdermatlas.com/photos/0000/1044/00001044_standalone.jpg
http://www.hellenicdermatlas.com/photos/0000/1045/00001045_standalone.jpg
http://www.hellenicdermatlas.com/photos/0000/1046/00001046_standalone.jpg
http://www.hellenicdermatlas.com/photos/0000/1047/00001047_standalone.jpg
http://www.hellenicdermatlas.com/photos/0000/1048/00001048_standalone.jpg
http://www.hellenicdermatlas.com/photos/0000/1049/00001049_standalone.jpg
http://www.hellenicdermatlas.com/photos/0000/1050/00001050_standalone.jpg
http://www.hellenicdermatlas.com/photos/0000/1051/00001051_standalone.jpg
http://www.hellenicdermatlas.com/photos/0000/1052/00001052_standalone.jpg
http://www.hellenicdermatlas.com/photos/0000/1053/00001053_standalone.jpg
http://www.hellenicdermatlas.com/photos/0000/1054/00001054_standalone.jpg
http://www.hellenicdermatlas.com/photos/0000/1055/00001055_standalone.jpg
http://www.hellenicdermatlas.com/photos/0000/1056/00001056_standalone.jpg
http://www.hellenicdermatlas.com/photos/0000/1057/00001057_standalone.jpg
http://www.hellenicdermatlas.com/photos/0000/1058/00001058_standalone.jpg
http://www.hellenicdermatlas.com/photos/0000/1059/00001059_standalone.jpg
http://www.hellenicdermatlas.com/photos/0000/1060/00001060_standalone.jpg
http://www.hellenicdermatlas.com/photos/0000/1061/00001061_standalone.jpg
http://www.hellenicdermatlas.com/photos/0000/1062/00001062_standalone.jpg
http://www.hellenicdermatlas.com/photos/0000/1063/00001063_standalone.jpg
http://www.hellenicdermatlas.com/photos/0000/1064/00001064_standalone.jpg
http://www.hellenicdermatlas.com/photos/0000/1065/00001065_standalone.jpg
http://www.hellenicdermatlas.com/photos/0000/1066/00001066_standalone.jpg
http://www.hellenicdermatlas.com/photos/0000/1067/00001067_standalone.jpg

http://www.hellenicdermatlas.com/photos/0000/1068/00001068_standalone.jpg
http://www.hellenicdermatlas.com/photos/0000/1069/00001069_standalone.jpg
http://www.hellenicdermatlas.com/photos/0000/1070/00001070_standalone.jpg
http://www.hellenicdermatlas.com/photos/0000/1071/00001071_standalone.jpg
http://www.hellenicdermatlas.com/photos/0000/1072/00001072_standalone.jpg
http://www.hellenicdermatlas.com/photos/0000/1073/00001073_standalone.jpg
http://www.hellenicdermatlas.com/photos/0000/1074/00001074_standalone.jpg
http://www.hellenicdermatlas.com/photos/0000/1075/00001075_standalone.jpg
http://www.hellenicdermatlas.com/photos/0000/1076/00001076_standalone.jpg
http://www.hellenicdermatlas.com/photos/0000/1077/00001077_standalone.jpg
http://www.hellenicdermatlas.com/photos/0000/1078/00001078_standalone.jpg
http://www.hellenicdermatlas.com/photos/0000/1079/00001079_standalone.jpg
http://www.hellenicdermatlas.com/photos/0000/1080/00001080_standalone.jpg
http://www.hellenicdermatlas.com/photos/0000/1081/00001081_standalone.jpg
http://www.hellenicdermatlas.com/photos/0000/1082/00001082_standalone.jpg
http://www.hellenicdermatlas.com/photos/0000/1083/00001083_standalone.jpg
http://www.hellenicdermatlas.com/photos/0000/1084/00001084_standalone.jpg
http://www.hellenicdermatlas.com/photos/0000/1085/00001085_standalone.jpg
http://www.hellenicdermatlas.com/photos/0000/1086/00001086_standalone.jpg
http://www.hellenicdermatlas.com/photos/0000/1087/00001087_standalone.jpg
http://www.hellenicdermatlas.com/photos/0000/1088/00001088_standalone.jpg
http://www.hellenicdermatlas.com/photos/0000/1089/00001089_standalone.jpg
http://www.hellenicdermatlas.com/photos/0000/1090/00001090_standalone.jpg
http://www.hellenicdermatlas.com/photos/0000/1091/00001091_standalone.jpg
http://www.hellenicdermatlas.com/photos/0000/1092/00001092_standalone.jpg
http://www.hellenicdermatlas.com/photos/0000/1093/00001093_standalone.jpg
http://www.hellenicdermatlas.com/photos/0000/1094/00001094_standalone.jpg
http://www.hellenicdermatlas.com/photos/0000/1095/00001095_standalone.jpg
http://www.hellenicdermatlas.com/photos/0000/1096/00001096_standalone.jpg
http://www.hellenicdermatlas.com/photos/0000/1097/00001097_standalone.jpg
http://www.hellenicdermatlas.com/photos/0000/1098/00001098_standalone.jpg
http://www.hellenicdermatlas.com/photos/0000/1099/00001099_standalone.jpg
http://www.hellenicdermatlas.com/photos/0000/1100/00001100_standalone.jpg
http://www.hellenicdermatlas.com/photos/0000/1101/00001101_standalone.jpg
http://www.hellenicdermatlas.com/photos/0000/1102/00001102_standalone.jpg
http://www.hellenicdermatlas.com/photos/0000/1103/00001103_standalone.jpg
http://www.hellenicdermatlas.com/photos/0000/1104/00001104_standalone.jpg
http://www.hellenicdermatlas.com/photos/0000/1105/00001105_standalone.jpg
http://www.hellenicdermatlas.com/photos/0000/1106/00001106_standalone.jpg
http://www.hellenicdermatlas.com/photos/0000/1107/00001107_standalone.jpg
http://www.hellenicdermatlas.com/photos/0000/1108/00001108_standalone.jpg
http://www.hellenicdermatlas.com/photos/0000/1109/00001109_standalone.jpg
http://www.hellenicdermatlas.com/photos/0000/1110/00001110_standalone.jpg
http://www.hellenicdermatlas.com/photos/0000/1111/00001111_standalone.jpg
http://www.hellenicdermatlas.com/photos/0000/1112/00001112_standalone.jpg
http://www.hellenicdermatlas.com/photos/0000/1113/00001113_standalone.jpg
http://www.hellenicdermatlas.com/photos/0000/1114/00001114_standalone.jpg
http://www.hellenicdermatlas.com/photos/0000/1115/00001115_standalone.jpg

http://www.hellenicdermatlas.com/photos/0000/1116/00001116_standalone.jpg
http://www.hellenicdermatlas.com/photos/0000/1117/00001117_standalone.jpg
http://www.hellenicdermatlas.com/photos/0000/1118/00001118_standalone.jpg
http://www.hellenicdermatlas.com/photos/0000/1119/00001119_standalone.jpg
http://www.hellenicdermatlas.com/photos/0000/1120/00001120_standalone.jpg
http://www.hellenicdermatlas.com/photos/0000/1121/00001121_standalone.jpg
http://www.hellenicdermatlas.com/photos/0000/1122/00001122_standalone.jpg
http://www.hellenicdermatlas.com/photos/0000/1123/00001123_standalone.jpg
http://www.hellenicdermatlas.com/photos/0000/1124/00001124_standalone.jpg
http://www.hellenicdermatlas.com/photos/0000/1125/00001125_standalone.jpg
http://www.hellenicdermatlas.com/photos/0000/1126/00001126_standalone.jpg
http://www.hellenicdermatlas.com/photos/0000/1127/00001127_standalone.jpg
http://www.hellenicdermatlas.com/photos/0000/1128/00001128_standalone.jpg
http://www.hellenicdermatlas.com/photos/0000/1129/00001129_standalone.jpg
http://www.hellenicdermatlas.com/photos/0000/1130/00001130_standalone.jpg
http://www.hellenicdermatlas.com/photos/0000/1131/00001131_standalone.jpg
http://www.hellenicdermatlas.com/photos/0000/1132/00001132_standalone.jpg
http://www.hellenicdermatlas.com/photos/0000/1133/00001133_standalone.jpg
http://www.hellenicdermatlas.com/photos/0000/1134/00001134_standalone.jpg
http://www.hellenicdermatlas.com/photos/0000/1135/00001135_standalone.jpg
http://www.hellenicdermatlas.com/photos/0000/1136/00001136_standalone.jpg
http://www.hellenicdermatlas.com/photos/0000/1137/00001137_standalone.jpg
http://www.hellenicdermatlas.com/photos/0000/1138/00001138_standalone.jpg
http://www.hellenicdermatlas.com/photos/0000/1139/00001139_standalone.jpg
http://www.hellenicdermatlas.com/photos/0000/1140/00001140_standalone.jpg
http://www.hellenicdermatlas.com/photos/0000/1141/00001141_standalone.jpg
http://www.hellenicdermatlas.com/photos/0000/1142/00001142_standalone.jpg
http://www.hellenicdermatlas.com/photos/0000/1143/00001143_standalone.jpg
http://www.hellenicdermatlas.com/photos/0000/1144/00001144_standalone.jpg
http://www.hellenicdermatlas.com/photos/0000/1145/00001145_standalone.jpg
http://www.hellenicdermatlas.com/photos/0000/1146/00001146_standalone.jpg
http://www.hellenicdermatlas.com/photos/0000/1147/00001147_standalone.jpg
http://www.hellenicdermatlas.com/photos/0000/1148/00001148_standalone.jpg
http://www.hellenicdermatlas.com/photos/0000/1149/00001149_standalone.jpg
http://www.hellenicdermatlas.com/photos/0000/1150/00001150_standalone.jpg
http://www.hellenicdermatlas.com/photos/0000/1151/00001151_standalone.jpg
http://www.hellenicdermatlas.com/photos/0000/1152/00001152_standalone.jpg
http://www.hellenicdermatlas.com/photos/0000/1153/00001153_standalone.jpg
http://www.hellenicdermatlas.com/photos/0000/1154/00001154_standalone.jpg
http://www.hellenicdermatlas.com/photos/0000/1155/00001155_standalone.jpg
http://www.hellenicdermatlas.com/photos/0000/1156/00001156_standalone.jpg
http://www.hellenicdermatlas.com/photos/0000/1157/00001157_standalone.jpg
http://www.hellenicdermatlas.com/photos/0000/1158/00001158_standalone.jpg
http://www.hellenicdermatlas.com/photos/0000/1159/00001159_standalone.jpg
http://www.hellenicdermatlas.com/photos/0000/1160/00001160_standalone.jpg
http://www.hellenicdermatlas.com/photos/0000/1161/00001161_standalone.jpg
http://www.hellenicdermatlas.com/photos/0000/1162/00001162_standalone.jpg
http://www.hellenicdermatlas.com/photos/0000/1163/00001163_standalone.jpg

http://www.hellenicdermatlas.com/photos/0000/1164/00001164_standalone.jpg
http://www.hellenicdermatlas.com/photos/0000/1165/00001165_standalone.jpg
http://www.hellenicdermatlas.com/photos/0000/1166/00001166_standalone.jpg
http://www.hellenicdermatlas.com/photos/0000/1167/00001167_standalone.jpg
http://www.hellenicdermatlas.com/photos/0000/1168/00001168_standalone.jpg
http://www.hellenicdermatlas.com/photos/0000/1169/00001169_standalone.jpg
http://www.hellenicdermatlas.com/photos/0000/1170/00001170_standalone.jpg
http://www.hellenicdermatlas.com/photos/0000/1171/00001171_standalone.jpg
http://www.hellenicdermatlas.com/photos/0000/1172/00001172_standalone.jpg
http://www.hellenicdermatlas.com/photos/0000/1173/00001173_standalone.jpg
http://www.hellenicdermatlas.com/photos/0000/1174/00001174_standalone.jpg
http://www.hellenicdermatlas.com/photos/0000/1175/00001175_standalone.jpg
http://www.hellenicdermatlas.com/photos/0000/1176/00001176_standalone.jpg
http://www.hellenicdermatlas.com/photos/0000/1177/00001177_standalone.jpg
http://www.hellenicdermatlas.com/photos/0000/1178/00001178_standalone.jpg
http://www.hellenicdermatlas.com/photos/0000/1179/00001179_standalone.jpg
http://www.hellenicdermatlas.com/photos/0000/1180/00001180_standalone.jpg
http://www.hellenicdermatlas.com/photos/0000/1181/00001181_standalone.jpg
http://www.hellenicdermatlas.com/photos/0000/1182/00001182_standalone.jpg
http://www.hellenicdermatlas.com/photos/0000/1183/00001183_standalone.jpg
http://www.hellenicdermatlas.com/photos/0000/1184/00001184_standalone.jpg
http://www.hellenicdermatlas.com/photos/0000/1185/00001185_standalone.jpg
http://www.hellenicdermatlas.com/photos/0000/1186/00001186_standalone.jpg
http://www.hellenicdermatlas.com/photos/0000/1187/00001187_standalone.jpg
http://www.hellenicdermatlas.com/photos/0000/1188/00001188_standalone.jpg
http://www.hellenicdermatlas.com/photos/0000/1189/00001189_standalone.jpg
http://www.hellenicdermatlas.com/photos/0000/1190/00001190_standalone.jpg
http://www.hellenicdermatlas.com/photos/0000/1191/00001191_standalone.jpg
http://www.hellenicdermatlas.com/photos/0000/1192/00001192_standalone.jpg
http://www.hellenicdermatlas.com/photos/0000/1193/00001193_standalone.jpg
http://www.hellenicdermatlas.com/photos/0000/1194/00001194_standalone.jpg
http://www.hellenicdermatlas.com/photos/0000/1195/00001195_standalone.jpg
http://www.hellenicdermatlas.com/photos/0000/1196/00001196_standalone.jpg
http://www.hellenicdermatlas.com/photos/0000/1197/00001197_standalone.jpg
http://www.hellenicdermatlas.com/photos/0000/1198/00001198_standalone.jpg
http://www.hellenicdermatlas.com/photos/0000/1199/00001199_standalone.jpg
http://www.hellenicdermatlas.com/photos/0000/1200/00001200_standalone.jpg
http://www.hellenicdermatlas.com/photos/0000/1201/00001201_standalone.jpg
http://www.hellenicdermatlas.com/photos/0000/1202/00001202_standalone.jpg
http://www.hellenicdermatlas.com/photos/0000/1203/00001203_standalone.jpg
http://www.hellenicdermatlas.com/photos/0000/1204/00001204_standalone.jpg
http://www.hellenicdermatlas.com/photos/0000/1205/00001205_standalone.jpg
http://www.hellenicdermatlas.com/photos/0000/1206/00001206_standalone.jpg
http://www.hellenicdermatlas.com/photos/0000/1207/00001207_standalone.jpg
http://www.hellenicdermatlas.com/photos/0000/1208/00001208_standalone.jpg
http://www.hellenicdermatlas.com/photos/0000/1209/00001209_standalone.jpg
http://www.hellenicdermatlas.com/photos/0000/1210/00001210_standalone.jpg
http://www.hellenicdermatlas.com/photos/0000/1211/00001211_standalone.jpg

http://www.hellenicdermatlas.com/photos/0000/1212/00001212_standalone.jpg
http://www.hellenicdermatlas.com/photos/0000/1213/00001213_standalone.jpg
http://www.hellenicdermatlas.com/photos/0000/1214/00001214_standalone.jpg
http://www.hellenicdermatlas.com/photos/0000/1215/00001215_standalone.jpg
http://www.hellenicdermatlas.com/photos/0000/1216/00001216_standalone.jpg
http://www.hellenicdermatlas.com/photos/0000/1217/00001217_standalone.jpg
http://www.hellenicdermatlas.com/photos/0000/1218/00001218_standalone.jpg
http://www.hellenicdermatlas.com/photos/0000/1219/00001219_standalone.jpg
http://www.hellenicdermatlas.com/photos/0000/1220/00001220_standalone.jpg
http://www.hellenicdermatlas.com/photos/0000/1221/00001221_standalone.jpg
http://www.hellenicdermatlas.com/photos/0000/1222/00001222_standalone.jpg
http://www.hellenicdermatlas.com/photos/0000/1223/00001223_standalone.jpg
http://www.hellenicdermatlas.com/photos/0000/1224/00001224_standalone.jpg
http://www.hellenicdermatlas.com/photos/0000/1225/00001225_standalone.jpg
http://www.hellenicdermatlas.com/photos/0000/1226/00001226_standalone.jpg
http://www.hellenicdermatlas.com/photos/0000/1227/00001227_standalone.jpg
http://www.hellenicdermatlas.com/photos/0000/1228/00001228_standalone.jpg
http://www.hellenicdermatlas.com/photos/0000/1229/00001229_standalone.jpg
http://www.hellenicdermatlas.com/photos/0000/1230/00001230_standalone.jpg
http://www.hellenicdermatlas.com/photos/0000/1231/00001231_standalone.jpg
http://www.hellenicdermatlas.com/photos/0000/1232/00001232_standalone.jpg
http://www.hellenicdermatlas.com/photos/0000/1233/00001233_standalone.jpg
http://www.hellenicdermatlas.com/photos/0000/1234/00001234_standalone.jpg
http://www.hellenicdermatlas.com/photos/0000/1235/00001235_standalone.jpg
http://www.hellenicdermatlas.com/photos/0000/1236/00001236_standalone.jpg
http://www.hellenicdermatlas.com/photos/0000/1237/00001237_standalone.jpg
http://www.hellenicdermatlas.com/photos/0000/1238/00001238_standalone.jpg
http://www.hellenicdermatlas.com/photos/0000/1239/00001239_standalone.jpg
http://www.hellenicdermatlas.com/photos/0000/1240/00001240_standalone.jpg
http://www.hellenicdermatlas.com/photos/0000/1241/00001241_standalone.jpg
http://www.hellenicdermatlas.com/photos/0000/1242/00001242_standalone.jpg
http://www.hellenicdermatlas.com/photos/0000/1243/00001243_standalone.jpg
http://www.hellenicdermatlas.com/photos/0000/1244/00001244_standalone.jpg
http://www.hellenicdermatlas.com/photos/0000/1245/00001245_standalone.jpg
http://www.hellenicdermatlas.com/photos/0000/1246/00001246_standalone.jpg
http://www.hellenicdermatlas.com/photos/0000/1247/00001247_standalone.jpg
http://www.hellenicdermatlas.com/photos/0000/1248/00001248_standalone.jpg
http://www.hellenicdermatlas.com/photos/0000/1249/00001249_standalone.jpg
http://www.hellenicdermatlas.com/photos/0000/1250/00001250_standalone.jpg
http://www.hellenicdermatlas.com/photos/0000/1251/00001251_standalone.jpg
http://www.hellenicdermatlas.com/photos/0000/1252/00001252_standalone.jpg
http://www.hellenicdermatlas.com/photos/0000/1253/00001253_standalone.jpg
http://www.hellenicdermatlas.com/photos/0000/1254/00001254_standalone.jpg
http://www.hellenicdermatlas.com/photos/0000/1255/00001255_standalone.jpg
http://www.hellenicdermatlas.com/photos/0000/1256/00001256_standalone.jpg
http://www.hellenicdermatlas.com/photos/0000/1257/00001257_standalone.jpg
http://www.hellenicdermatlas.com/photos/0000/1258/00001258_standalone.jpg
http://www.hellenicdermatlas.com/photos/0000/1259/00001259_standalone.jpg

http://www.hellenicdermatlas.com/photos/0000/1260/00001260_standalone.jpg
http://www.hellenicdermatlas.com/photos/0000/1261/00001261_standalone.jpg
http://www.hellenicdermatlas.com/photos/0000/1262/00001262_standalone.jpg
http://www.hellenicdermatlas.com/photos/0000/1263/00001263_standalone.jpg
http://www.hellenicdermatlas.com/photos/0000/1264/00001264_standalone.jpg
http://www.hellenicdermatlas.com/photos/0000/1265/00001265_standalone.jpg
http://www.hellenicdermatlas.com/photos/0000/1266/00001266_standalone.jpg
http://www.hellenicdermatlas.com/photos/0000/1267/00001267_standalone.jpg
http://www.hellenicdermatlas.com/photos/0000/1268/00001268_standalone.jpg
http://www.hellenicdermatlas.com/photos/0000/1269/00001269_standalone.jpg
http://www.hellenicdermatlas.com/photos/0000/1270/00001270_standalone.jpg
http://www.hellenicdermatlas.com/photos/0000/1271/00001271_standalone.jpg
http://www.hellenicdermatlas.com/photos/0000/1272/00001272_standalone.jpg
http://www.hellenicdermatlas.com/photos/0000/1273/00001273_standalone.jpg
http://www.hellenicdermatlas.com/photos/0000/1274/00001274_standalone.jpg
http://www.hellenicdermatlas.com/photos/0000/1275/00001275_standalone.jpg
http://www.hellenicdermatlas.com/photos/0000/1276/00001276_standalone.jpg
http://www.hellenicdermatlas.com/photos/0000/1277/00001277_standalone.jpg
http://www.hellenicdermatlas.com/photos/0000/1278/00001278_standalone.jpg
http://www.hellenicdermatlas.com/photos/0000/1279/00001279_standalone.jpg
http://www.hellenicdermatlas.com/photos/0000/1280/00001280_standalone.jpg
http://www.hellenicdermatlas.com/photos/0000/1281/00001281_standalone.jpg
http://www.hellenicdermatlas.com/photos/0000/1282/00001282_standalone.jpg
http://www.hellenicdermatlas.com/photos/0000/1283/00001283_standalone.jpg
http://www.hellenicdermatlas.com/photos/0000/1284/00001284_standalone.jpg
http://www.hellenicdermatlas.com/photos/0000/1285/00001285_standalone.jpg
http://www.hellenicdermatlas.com/photos/0000/1286/00001286_standalone.jpg
http://www.hellenicdermatlas.com/photos/0000/1287/00001287_standalone.jpg
http://www.hellenicdermatlas.com/photos/0000/1288/00001288_standalone.jpg
http://www.hellenicdermatlas.com/photos/0000/1289/00001289_standalone.jpg
http://www.hellenicdermatlas.com/photos/0000/1290/00001290_standalone.jpg
http://www.hellenicdermatlas.com/photos/0000/1291/00001291_standalone.jpg
http://www.hellenicdermatlas.com/photos/0000/1292/00001292_standalone.jpg
http://www.hellenicdermatlas.com/photos/0000/1293/00001293_standalone.jpg
http://www.hellenicdermatlas.com/photos/0000/1294/00001294_standalone.jpg
http://www.hellenicdermatlas.com/photos/0000/1295/00001295_standalone.jpg
http://www.hellenicdermatlas.com/photos/0000/1296/00001296_standalone.jpg
http://www.hellenicdermatlas.com/photos/0000/1297/00001297_standalone.jpg
http://www.hellenicdermatlas.com/photos/0000/1298/00001298_standalone.jpg
http://www.hellenicdermatlas.com/photos/0000/1299/00001299_standalone.jpg
http://www.hellenicdermatlas.com/photos/0000/1300/00001300_standalone.jpg
http://www.hellenicdermatlas.com/photos/0000/1301/00001301_standalone.jpg
http://www.hellenicdermatlas.com/photos/0000/1302/00001302_standalone.jpg
http://www.hellenicdermatlas.com/photos/0000/1303/00001303_standalone.jpg
http://www.hellenicdermatlas.com/photos/0000/1304/00001304_standalone.jpg
http://www.hellenicdermatlas.com/photos/0000/1305/00001305_standalone.jpg
http://www.hellenicdermatlas.com/photos/0000/1306/00001306_standalone.jpg
http://www.hellenicdermatlas.com/photos/0000/1307/00001307_standalone.jpg

http://www.hellenicdermatlas.com/photos/0000/1308/00001308_standalone.jpg
http://www.hellenicdermatlas.com/photos/0000/1309/00001309_standalone.jpg
http://www.hellenicdermatlas.com/photos/0000/1310/00001310_standalone.jpg
http://www.hellenicdermatlas.com/photos/0000/1311/00001311_standalone.jpg
http://www.hellenicdermatlas.com/photos/0000/1312/00001312_standalone.jpg
http://www.hellenicdermatlas.com/photos/0000/1313/00001313_standalone.jpg
http://www.hellenicdermatlas.com/photos/0000/1314/00001314_standalone.jpg
http://www.hellenicdermatlas.com/photos/0000/1315/00001315_standalone.jpg
http://www.hellenicdermatlas.com/photos/0000/1316/00001316_standalone.jpg
http://www.hellenicdermatlas.com/photos/0000/1317/00001317_standalone.jpg
http://www.hellenicdermatlas.com/photos/0000/1318/00001318_standalone.jpg
http://www.hellenicdermatlas.com/photos/0000/1319/00001319_standalone.jpg
http://www.hellenicdermatlas.com/photos/0000/1320/00001320_standalone.jpg
http://www.hellenicdermatlas.com/photos/0000/1321/00001321_standalone.jpg
http://www.hellenicdermatlas.com/photos/0000/1322/00001322_standalone.jpg
http://www.hellenicdermatlas.com/photos/0000/1323/00001323_standalone.jpg
http://www.hellenicdermatlas.com/photos/0000/1324/00001324_standalone.jpg
http://www.hellenicdermatlas.com/photos/0000/1325/00001325_standalone.jpg
http://www.hellenicdermatlas.com/photos/0000/1326/00001326_standalone.jpg
http://www.hellenicdermatlas.com/photos/0000/1327/00001327_standalone.jpg
http://www.hellenicdermatlas.com/photos/0000/1328/00001328_standalone.jpg
http://www.hellenicdermatlas.com/photos/0000/1329/00001329_standalone.jpg
http://www.hellenicdermatlas.com/photos/0000/1330/00001330_standalone.jpg
http://www.hellenicdermatlas.com/photos/0000/1331/00001331_standalone.jpg
http://www.hellenicdermatlas.com/photos/0000/1332/00001332_standalone.jpg
http://www.hellenicdermatlas.com/photos/0000/1333/00001333_standalone.jpg
http://www.hellenicdermatlas.com/photos/0000/1334/00001334_standalone.jpg
http://www.hellenicdermatlas.com/photos/0000/1335/00001335_standalone.jpg
http://www.hellenicdermatlas.com/photos/0000/1336/00001336_standalone.jpg
http://www.hellenicdermatlas.com/photos/0000/1337/00001337_standalone.jpg
http://www.hellenicdermatlas.com/photos/0000/1338/00001338_standalone.jpg
http://www.hellenicdermatlas.com/photos/0000/1339/00001339_standalone.jpg
http://www.hellenicdermatlas.com/photos/0000/1340/00001340_standalone.jpg
http://www.hellenicdermatlas.com/photos/0000/1341/00001341_standalone.jpg
http://www.hellenicdermatlas.com/photos/0000/1342/00001342_standalone.jpg
http://www.hellenicdermatlas.com/photos/0000/1343/00001343_standalone.jpg
http://www.hellenicdermatlas.com/photos/0000/1344/00001344_standalone.jpg
http://www.hellenicdermatlas.com/photos/0000/1345/00001345_standalone.jpg
http://www.hellenicdermatlas.com/photos/0000/1346/00001346_standalone.jpg
http://www.hellenicdermatlas.com/photos/0000/1347/00001347_standalone.jpg
http://www.hellenicdermatlas.com/photos/0000/1348/00001348_standalone.jpg
http://www.hellenicdermatlas.com/photos/0000/1349/00001349_standalone.jpg
http://www.hellenicdermatlas.com/photos/0000/1350/00001350_standalone.jpg
http://www.hellenicdermatlas.com/photos/0000/1351/00001351_standalone.jpg
http://www.hellenicdermatlas.com/photos/0000/1352/00001352_standalone.jpg
http://www.hellenicdermatlas.com/photos/0000/1353/00001353_standalone.jpg
http://www.hellenicdermatlas.com/photos/0000/1354/00001354_standalone.jpg
http://www.hellenicdermatlas.com/photos/0000/1355/00001355_standalone.jpg

http://www.hellenicdermatlas.com/photos/0000/1356/00001356_standalone.jpg
http://www.hellenicdermatlas.com/photos/0000/1357/00001357_standalone.jpg
http://www.hellenicdermatlas.com/photos/0000/1358/00001358_standalone.jpg
http://www.hellenicdermatlas.com/photos/0000/1359/00001359_standalone.jpg
http://www.hellenicdermatlas.com/photos/0000/1360/00001360_standalone.jpg
http://www.hellenicdermatlas.com/photos/0000/1361/00001361_standalone.jpg
http://www.hellenicdermatlas.com/photos/0000/1362/00001362_standalone.jpg
http://www.hellenicdermatlas.com/photos/0000/1363/00001363_standalone.jpg
http://www.hellenicdermatlas.com/photos/0000/1364/00001364_standalone.jpg
http://www.hellenicdermatlas.com/photos/0000/1365/00001365_standalone.jpg
http://www.hellenicdermatlas.com/photos/0000/1366/00001366_standalone.jpg
http://www.hellenicdermatlas.com/photos/0000/1367/00001367_standalone.jpg
http://www.hellenicdermatlas.com/photos/0000/1368/00001368_standalone.jpg
http://www.hellenicdermatlas.com/photos/0000/1369/00001369_standalone.jpg
http://www.hellenicdermatlas.com/photos/0000/1370/00001370_standalone.jpg
http://www.hellenicdermatlas.com/photos/0000/1371/00001371_standalone.jpg
http://www.hellenicdermatlas.com/photos/0000/1372/00001372_standalone.jpg
http://www.hellenicdermatlas.com/photos/0000/1373/00001373_standalone.jpg
http://www.hellenicdermatlas.com/photos/0000/1374/00001374_standalone.jpg
http://www.hellenicdermatlas.com/photos/0000/1375/00001375_standalone.jpg
http://www.hellenicdermatlas.com/photos/0000/1376/00001376_standalone.jpg
http://www.hellenicdermatlas.com/photos/0000/1377/00001377_standalone.jpg
http://www.hellenicdermatlas.com/photos/0000/1378/00001378_standalone.jpg
http://www.hellenicdermatlas.com/photos/0000/1379/00001379_standalone.jpg
http://www.hellenicdermatlas.com/photos/0000/1380/00001380_standalone.jpg
http://www.hellenicdermatlas.com/photos/0000/1381/00001381_standalone.jpg
http://www.hellenicdermatlas.com/photos/0000/1382/00001382_standalone.jpg
http://www.hellenicdermatlas.com/photos/0000/1383/00001383_standalone.jpg
http://www.hellenicdermatlas.com/photos/0000/1384/00001384_standalone.jpg
http://www.hellenicdermatlas.com/photos/0000/1385/00001385_standalone.jpg
http://www.hellenicdermatlas.com/photos/0000/1386/00001386_standalone.jpg
http://www.hellenicdermatlas.com/photos/0000/1387/00001387_standalone.jpg
http://www.hellenicdermatlas.com/photos/0000/1388/00001388_standalone.jpg
http://www.hellenicdermatlas.com/photos/0000/1389/00001389_standalone.jpg
http://www.hellenicdermatlas.com/photos/0000/1390/00001390_standalone.jpg
http://www.hellenicdermatlas.com/photos/0000/1391/00001391_standalone.jpg
http://www.hellenicdermatlas.com/photos/0000/1392/00001392_standalone.jpg
http://www.hellenicdermatlas.com/photos/0000/1393/00001393_standalone.jpg
http://www.hellenicdermatlas.com/photos/0000/1394/00001394_standalone.jpg
http://www.hellenicdermatlas.com/photos/0000/1395/00001395_standalone.jpg
http://www.hellenicdermatlas.com/photos/0000/1396/00001396_standalone.jpg
http://www.hellenicdermatlas.com/photos/0000/1397/00001397_standalone.jpg
http://www.hellenicdermatlas.com/photos/0000/1398/00001398_standalone.jpg
http://www.hellenicdermatlas.com/photos/0000/1399/00001399_standalone.jpg
http://www.hellenicdermatlas.com/photos/0000/1400/00001400_standalone.jpg
http://www.hellenicdermatlas.com/photos/0000/1401/00001401_standalone.jpg
http://www.hellenicdermatlas.com/photos/0000/1402/00001402_standalone.jpg
http://www.hellenicdermatlas.com/photos/0000/1403/00001403_standalone.jpg

http://www.hellenicdermatlas.com/photos/0000/1404/00001404_standalone.jpg
http://www.hellenicdermatlas.com/photos/0000/1405/00001405_standalone.jpg
http://www.hellenicdermatlas.com/photos/0000/1406/00001406_standalone.jpg
http://www.hellenicdermatlas.com/photos/0000/1407/00001407_standalone.jpg
http://www.hellenicdermatlas.com/photos/0000/1408/00001408_standalone.jpg
http://www.hellenicdermatlas.com/photos/0000/1409/00001409_standalone.jpg
http://www.hellenicdermatlas.com/photos/0000/1410/00001410_standalone.jpg
http://www.hellenicdermatlas.com/photos/0000/1411/00001411_standalone.jpg
http://www.hellenicdermatlas.com/photos/0000/1412/00001412_standalone.jpg
http://www.hellenicdermatlas.com/photos/0000/1413/00001413_standalone.jpg
http://www.hellenicdermatlas.com/photos/0000/1414/00001414_standalone.jpg
http://www.hellenicdermatlas.com/photos/0000/1415/00001415_standalone.jpg
http://www.hellenicdermatlas.com/photos/0000/1416/00001416_standalone.jpg
http://www.hellenicdermatlas.com/photos/0000/1417/00001417_standalone.jpg
http://www.hellenicdermatlas.com/photos/0000/1418/00001418_standalone.jpg
http://www.hellenicdermatlas.com/photos/0000/1419/00001419_standalone.jpg
http://www.hellenicdermatlas.com/photos/0000/1420/00001420_standalone.jpg
http://www.hellenicdermatlas.com/photos/0000/1421/00001421_standalone.jpg
http://www.hellenicdermatlas.com/photos/0000/1422/00001422_standalone.jpg
http://www.hellenicdermatlas.com/photos/0000/1423/00001423_standalone.jpg
http://www.hellenicdermatlas.com/photos/0000/1424/00001424_standalone.jpg
http://www.hellenicdermatlas.com/photos/0000/1425/00001425_standalone.jpg
http://www.hellenicdermatlas.com/photos/0000/1426/00001426_standalone.jpg
http://www.hellenicdermatlas.com/photos/0000/1427/00001427_standalone.jpg
http://www.hellenicdermatlas.com/photos/0000/1428/00001428_standalone.jpg
http://www.hellenicdermatlas.com/photos/0000/1429/00001429_standalone.jpg
http://www.hellenicdermatlas.com/photos/0000/1430/00001430_standalone.jpg
http://www.hellenicdermatlas.com/photos/0000/1431/00001431_standalone.jpg
http://www.hellenicdermatlas.com/photos/0000/1432/00001432_standalone.jpg
http://www.hellenicdermatlas.com/photos/0000/1433/00001433_standalone.jpg
http://www.hellenicdermatlas.com/photos/0000/1434/00001434_standalone.jpg
http://www.hellenicdermatlas.com/photos/0000/1435/00001435_standalone.jpg
http://www.hellenicdermatlas.com/photos/0000/1436/00001436_standalone.jpg
http://www.hellenicdermatlas.com/photos/0000/1437/00001437_standalone.jpg
http://www.hellenicdermatlas.com/photos/0000/1438/00001438_standalone.jpg
http://www.hellenicdermatlas.com/photos/0000/1439/00001439_standalone.jpg
http://www.hellenicdermatlas.com/photos/0000/1440/00001440_standalone.jpg
http://www.hellenicdermatlas.com/photos/0000/1441/00001441_standalone.jpg
http://www.hellenicdermatlas.com/photos/0000/1442/00001442_standalone.jpg
http://www.hellenicdermatlas.com/photos/0000/1443/00001443_standalone.jpg
http://www.hellenicdermatlas.com/photos/0000/1444/00001444_standalone.jpg
http://www.hellenicdermatlas.com/photos/0000/1445/00001445_standalone.jpg
http://www.hellenicdermatlas.com/photos/0000/1446/00001446_standalone.jpg
http://www.hellenicdermatlas.com/photos/0000/1447/00001447_standalone.jpg
http://www.hellenicdermatlas.com/photos/0000/1448/00001448_standalone.jpg
http://www.hellenicdermatlas.com/photos/0000/1449/00001449_standalone.jpg
http://www.hellenicdermatlas.com/photos/0000/1450/00001450_standalone.jpg
http://www.hellenicdermatlas.com/photos/0000/1451/00001451_standalone.jpg

http://www.hellenicdermatlas.com/photos/0000/1452/00001452_standalone.jpg
http://www.hellenicdermatlas.com/photos/0000/1453/00001453_standalone.jpg
http://www.hellenicdermatlas.com/photos/0000/1454/00001454_standalone.jpg
http://www.hellenicdermatlas.com/photos/0000/1455/00001455_standalone.jpg
http://www.hellenicdermatlas.com/photos/0000/1456/00001456_standalone.jpg
http://www.hellenicdermatlas.com/photos/0000/1457/00001457_standalone.jpg
http://www.hellenicdermatlas.com/photos/0000/1458/00001458_standalone.jpg
http://www.hellenicdermatlas.com/photos/0000/1459/00001459_standalone.jpg
http://www.hellenicdermatlas.com/photos/0000/1460/00001460_standalone.jpg
http://www.hellenicdermatlas.com/photos/0000/1461/00001461_standalone.jpg
http://www.hellenicdermatlas.com/photos/0000/1462/00001462_standalone.jpg
http://www.hellenicdermatlas.com/photos/0000/1463/00001463_standalone.jpg
http://www.hellenicdermatlas.com/photos/0000/1464/00001464_standalone.jpg
http://www.hellenicdermatlas.com/photos/0000/1465/00001465_standalone.jpg
http://www.hellenicdermatlas.com/photos/0000/1466/00001466_standalone.jpg
http://www.hellenicdermatlas.com/photos/0000/1467/00001467_standalone.jpg
http://www.hellenicdermatlas.com/photos/0000/1468/00001468_standalone.jpg
http://www.hellenicdermatlas.com/photos/0000/1469/00001469_standalone.jpg
http://www.hellenicdermatlas.com/photos/0000/1470/00001470_standalone.jpg
http://www.hellenicdermatlas.com/photos/0000/1471/00001471_standalone.jpg
http://www.hellenicdermatlas.com/photos/0000/1472/00001472_standalone.jpg
http://www.hellenicdermatlas.com/photos/0000/1473/00001473_standalone.jpg
http://www.hellenicdermatlas.com/photos/0000/1474/00001474_standalone.jpg
http://www.hellenicdermatlas.com/photos/0000/1475/00001475_standalone.jpg
http://www.hellenicdermatlas.com/photos/0000/1476/00001476_standalone.jpg
http://www.hellenicdermatlas.com/photos/0000/1477/00001477_standalone.jpg
http://www.hellenicdermatlas.com/photos/0000/1478/00001478_standalone.jpg
http://www.hellenicdermatlas.com/photos/0000/1479/00001479_standalone.jpg
http://www.hellenicdermatlas.com/photos/0000/1480/00001480_standalone.jpg
http://www.hellenicdermatlas.com/photos/0000/1481/00001481_standalone.jpg
http://www.hellenicdermatlas.com/photos/0000/1482/00001482_standalone.jpg
http://www.hellenicdermatlas.com/photos/0000/1483/00001483_standalone.jpg
http://www.hellenicdermatlas.com/photos/0000/1484/00001484_standalone.jpg
http://www.hellenicdermatlas.com/photos/0000/1485/00001485_standalone.jpg
http://www.hellenicdermatlas.com/photos/0000/1486/00001486_standalone.jpg
http://www.hellenicdermatlas.com/photos/0000/1487/00001487_standalone.jpg
http://www.hellenicdermatlas.com/photos/0000/1488/00001488_standalone.jpg
http://www.hellenicdermatlas.com/photos/0000/1489/00001489_standalone.jpg
http://www.hellenicdermatlas.com/photos/0000/1490/00001490_standalone.jpg
http://www.hellenicdermatlas.com/photos/0000/1491/00001491_standalone.jpg
http://www.hellenicdermatlas.com/photos/0000/1492/00001492_standalone.jpg
http://www.hellenicdermatlas.com/photos/0000/1493/00001493_standalone.jpg
http://www.hellenicdermatlas.com/photos/0000/1494/00001494_standalone.jpg
http://www.hellenicdermatlas.com/photos/0000/1495/00001495_standalone.jpg
http://www.hellenicdermatlas.com/photos/0000/1496/00001496_standalone.jpg
http://www.hellenicdermatlas.com/photos/0000/1497/00001497_standalone.jpg
http://www.hellenicdermatlas.com/photos/0000/1498/00001498_standalone.jpg
http://www.hellenicdermatlas.com/photos/0000/1499/00001499_standalone.jpg

http://www.hellenicdermatlas.com/photos/0000/1500/00001500_standalone.jpg
http://www.hellenicdermatlas.com/photos/0000/1501/00001501_standalone.jpg
http://www.hellenicdermatlas.com/photos/0000/1502/00001502_standalone.jpg
http://www.hellenicdermatlas.com/photos/0000/1503/00001503_standalone.jpg
http://www.hellenicdermatlas.com/photos/0000/1504/00001504_standalone.jpg
http://www.hellenicdermatlas.com/photos/0000/1505/00001505_standalone.jpg
http://www.hellenicdermatlas.com/photos/0000/1506/00001506_standalone.jpg
http://www.hellenicdermatlas.com/photos/0000/1507/00001507_standalone.jpg
http://www.hellenicdermatlas.com/photos/0000/1508/00001508_standalone.jpg
http://www.hellenicdermatlas.com/photos/0000/1509/00001509_standalone.jpg
http://www.hellenicdermatlas.com/photos/0000/1510/00001510_standalone.jpg
http://www.hellenicdermatlas.com/photos/0000/1511/00001511_standalone.jpg
http://www.hellenicdermatlas.com/photos/0000/1512/00001512_standalone.jpg
http://www.hellenicdermatlas.com/photos/0000/1513/00001513_standalone.jpg
http://www.hellenicdermatlas.com/photos/0000/1514/00001514_standalone.jpg
http://www.hellenicdermatlas.com/photos/0000/1515/00001515_standalone.jpg
http://www.hellenicdermatlas.com/photos/0000/1516/00001516_standalone.jpg
http://www.hellenicdermatlas.com/photos/0000/1517/00001517_standalone.jpg
http://www.hellenicdermatlas.com/photos/0000/1518/00001518_standalone.jpg
http://www.hellenicdermatlas.com/photos/0000/1519/00001519_standalone.jpg
http://www.hellenicdermatlas.com/photos/0000/1520/00001520_standalone.jpg
http://www.hellenicdermatlas.com/photos/0000/1521/00001521_standalone.jpg
http://www.hellenicdermatlas.com/photos/0000/1522/00001522_standalone.jpg
http://www.hellenicdermatlas.com/photos/0000/1523/00001523_standalone.jpg
http://www.hellenicdermatlas.com/photos/0000/1524/00001524_standalone.jpg
http://www.hellenicdermatlas.com/photos/0000/1525/00001525_standalone.jpg
http://www.hellenicdermatlas.com/photos/0000/1526/00001526_standalone.jpg
http://www.hellenicdermatlas.com/photos/0000/1527/00001527_standalone.jpg
http://www.hellenicdermatlas.com/photos/0000/1528/00001528_standalone.jpg
http://www.hellenicdermatlas.com/photos/0000/1529/00001529_standalone.jpg
http://www.hellenicdermatlas.com/photos/0000/1530/00001530_standalone.jpg
http://www.hellenicdermatlas.com/photos/0000/1531/00001531_standalone.jpg
http://www.hellenicdermatlas.com/photos/0000/1532/00001532_standalone.jpg
http://www.hellenicdermatlas.com/photos/0000/1533/00001533_standalone.jpg
http://www.hellenicdermatlas.com/photos/0000/1534/00001534_standalone.jpg
http://www.hellenicdermatlas.com/photos/0000/1535/00001535_standalone.jpg
http://www.hellenicdermatlas.com/photos/0000/1536/00001536_standalone.jpg
http://www.hellenicdermatlas.com/photos/0000/1537/00001537_standalone.jpg
http://www.hellenicdermatlas.com/photos/0000/1538/00001538_standalone.jpg
http://www.hellenicdermatlas.com/photos/0000/1539/00001539_standalone.jpg
http://www.hellenicdermatlas.com/photos/0000/1540/00001540_standalone.jpg
http://www.hellenicdermatlas.com/photos/0000/1541/00001541_standalone.jpg
http://www.hellenicdermatlas.com/photos/0000/1542/00001542_standalone.jpg
http://www.hellenicdermatlas.com/photos/0000/1543/00001543_standalone.jpg
http://www.hellenicdermatlas.com/photos/0000/1544/00001544_standalone.jpg
http://www.hellenicdermatlas.com/photos/0000/1545/00001545_standalone.jpg
http://www.hellenicdermatlas.com/photos/0000/1546/00001546_standalone.jpg
http://www.hellenicdermatlas.com/photos/0000/1547/00001547_standalone.jpg

http://www.hellenicdermatlas.com/photos/0000/1548/00001548_standalone.jpg
http://www.hellenicdermatlas.com/photos/0000/1549/00001549_standalone.jpg
http://www.hellenicdermatlas.com/photos/0000/1550/00001550_standalone.jpg
http://www.hellenicdermatlas.com/photos/0000/1551/00001551_standalone.jpg
http://www.hellenicdermatlas.com/photos/0000/1552/00001552_standalone.jpg
http://www.hellenicdermatlas.com/photos/0000/1553/00001553_standalone.jpg
http://www.hellenicdermatlas.com/photos/0000/1554/00001554_standalone.jpg
http://www.hellenicdermatlas.com/photos/0000/1555/00001555_standalone.jpg
http://www.hellenicdermatlas.com/photos/0000/1556/00001556_standalone.jpg
http://www.hellenicdermatlas.com/photos/0000/1557/00001557_standalone.jpg
http://www.hellenicdermatlas.com/photos/0000/1558/00001558_standalone.jpg
http://www.hellenicdermatlas.com/photos/0000/1559/00001559_standalone.jpg
http://www.hellenicdermatlas.com/photos/0000/1560/00001560_standalone.jpg
http://www.hellenicdermatlas.com/photos/0000/1561/00001561_standalone.jpg
http://www.hellenicdermatlas.com/photos/0000/1562/00001562_standalone.jpg
http://www.hellenicdermatlas.com/photos/0000/1563/00001563_standalone.jpg
http://www.hellenicdermatlas.com/photos/0000/1564/00001564_standalone.jpg
http://www.hellenicdermatlas.com/photos/0000/1565/00001565_standalone.jpg
http://www.hellenicdermatlas.com/photos/0000/1566/00001566_standalone.jpg
http://www.hellenicdermatlas.com/photos/0000/1567/00001567_standalone.jpg
http://www.hellenicdermatlas.com/photos/0000/1568/00001568_standalone.jpg
http://www.hellenicdermatlas.com/photos/0000/1569/00001569_standalone.jpg
http://www.hellenicdermatlas.com/photos/0000/1570/00001570_standalone.jpg
http://www.hellenicdermatlas.com/photos/0000/1571/00001571_standalone.jpg
http://www.hellenicdermatlas.com/photos/0000/1572/00001572_standalone.jpg
http://www.hellenicdermatlas.com/photos/0000/1573/00001573_standalone.jpg
http://www.hellenicdermatlas.com/photos/0000/1574/00001574_standalone.jpg
http://www.hellenicdermatlas.com/photos/0000/1575/00001575_standalone.jpg
http://www.hellenicdermatlas.com/photos/0000/1576/00001576_standalone.jpg
http://www.hellenicdermatlas.com/photos/0000/1577/00001577_standalone.jpg
http://www.hellenicdermatlas.com/photos/0000/1578/00001578_standalone.jpg
http://www.hellenicdermatlas.com/photos/0000/1579/00001579_standalone.jpg
http://www.hellenicdermatlas.com/photos/0000/1580/00001580_standalone.jpg
http://www.hellenicdermatlas.com/photos/0000/1581/00001581_standalone.jpg
http://www.hellenicdermatlas.com/photos/0000/1582/00001582_standalone.jpg
http://www.hellenicdermatlas.com/photos/0000/1583/00001583_standalone.jpg
http://www.hellenicdermatlas.com/photos/0000/1584/00001584_standalone.jpg
http://www.hellenicdermatlas.com/photos/0000/1585/00001585_standalone.jpg
http://www.hellenicdermatlas.com/photos/0000/1586/00001586_standalone.jpg
http://www.hellenicdermatlas.com/photos/0000/1587/00001587_standalone.jpg
http://www.hellenicdermatlas.com/photos/0000/1588/00001588_standalone.jpg
http://www.hellenicdermatlas.com/photos/0000/1589/00001589_standalone.jpg
http://www.hellenicdermatlas.com/photos/0000/1591/00001591_standalone.jpg
http://www.hellenicdermatlas.com/photos/0000/1594/00001594_standalone.jpg
http://www.hellenicdermatlas.com/photos/0000/1596/00001596_standalone.jpg
http://www.hellenicdermatlas.com/photos/0000/1598/00001598_standalone.jpg
http://www.hellenicdermatlas.com/photos/0000/1599/00001599_standalone.jpg
http://www.hellenicdermatlas.com/photos/0000/1600/00001600_standalone.jpg

http://www.hellenicdermatlas.com/photos/0000/1601/00001601_standalone.jpg
http://www.hellenicdermatlas.com/photos/0000/1603/00001603_standalone.jpg
http://www.hellenicdermatlas.com/photos/0000/1605/00001605_standalone.jpg
http://www.hellenicdermatlas.com/photos/0000/1606/00001606_standalone.jpg
http://www.hellenicdermatlas.com/photos/0000/1607/00001607_standalone.jpg
http://www.hellenicdermatlas.com/photos/0000/1610/00001610_standalone.jpg
http://www.hellenicdermatlas.com/photos/0000/1611/00001611_standalone.jpg
http://www.hellenicdermatlas.com/photos/0000/1612/00001612_standalone.jpg
http://www.hellenicdermatlas.com/photos/0000/1613/00001613_standalone.jpg
http://www.hellenicdermatlas.com/photos/0000/1614/00001614_standalone.jpg
http://www.hellenicdermatlas.com/photos/0000/1615/00001615_standalone.jpg
http://www.hellenicdermatlas.com/photos/0000/1616/00001616_standalone.jpg
http://www.hellenicdermatlas.com/photos/0000/1617/00001617_standalone.jpg
http://www.hellenicdermatlas.com/photos/0000/1618/00001618_standalone.jpg
http://www.hellenicdermatlas.com/photos/0000/1619/00001619_standalone.jpg
http://www.hellenicdermatlas.com/photos/0000/1620/00001620_standalone.jpg
http://www.hellenicdermatlas.com/photos/0000/1622/00001622_standalone.jpg
http://www.hellenicdermatlas.com/photos/0000/1623/00001623_standalone.jpg
http://www.hellenicdermatlas.com/photos/0000/1624/00001624_standalone.jpg
http://www.hellenicdermatlas.com/photos/0000/1625/00001625_standalone.jpg
http://www.hellenicdermatlas.com/photos/0000/1626/00001626_standalone.jpg
http://www.hellenicdermatlas.com/photos/0000/1627/00001627_standalone.jpg
http://www.hellenicdermatlas.com/photos/0000/1629/00001629_standalone.jpg
http://www.hellenicdermatlas.com/photos/0000/1630/00001630_standalone.jpg
http://www.hellenicdermatlas.com/photos/0000/1631/00001631_standalone.jpg
http://www.hellenicdermatlas.com/photos/0000/1632/00001632_standalone.jpg
http://www.hellenicdermatlas.com/photos/0000/1633/00001633_standalone.jpg
http://www.hellenicdermatlas.com/photos/0000/1635/00001635_standalone.jpg
http://www.hellenicdermatlas.com/photos/0000/1637/00001637_standalone.jpg
http://www.hellenicdermatlas.com/photos/0000/1638/00001638_standalone.jpg
http://www.hellenicdermatlas.com/photos/0000/1639/00001639_standalone.jpg
http://www.hellenicdermatlas.com/photos/0000/1640/00001640_standalone.jpg
http://www.hellenicdermatlas.com/photos/0000/1642/00001642_standalone.jpg
http://www.hellenicdermatlas.com/photos/0000/1644/00001644_standalone.jpg
http://www.hellenicdermatlas.com/photos/0000/1645/00001645_standalone.jpg
http://www.hellenicdermatlas.com/photos/0000/1646/00001646_standalone.jpg
http://www.hellenicdermatlas.com/photos/0000/1647/00001647_standalone.jpg
http://www.hellenicdermatlas.com/photos/0000/1648/00001648_standalone.jpg
http://www.hellenicdermatlas.com/photos/0000/1650/00001650_standalone.jpg
http://www.hellenicdermatlas.com/photos/0000/1651/00001651_standalone.jpg
http://www.hellenicdermatlas.com/photos/0000/1652/00001652_standalone.jpg
http://www.hellenicdermatlas.com/photos/0000/1653/00001653_standalone.jpg
http://www.hellenicdermatlas.com/photos/0000/1654/00001654_standalone.jpg
http://www.hellenicdermatlas.com/photos/0000/1655/00001655_standalone.jpg
http://www.hellenicdermatlas.com/photos/0000/1656/00001656_standalone.jpg
http://www.hellenicdermatlas.com/photos/0000/1657/00001657_standalone.jpg
http://www.hellenicdermatlas.com/photos/0000/1658/00001658_standalone.jpg
http://www.hellenicdermatlas.com/photos/0000/1660/00001660_standalone.jpg

http://www.hellenicdermatlas.com/photos/0000/1662/00001662_standalone.jpg
http://www.hellenicdermatlas.com/photos/0000/1663/00001663_standalone.jpg
http://www.hellenicdermatlas.com/photos/0000/1665/00001665_standalone.jpg
http://www.hellenicdermatlas.com/photos/0000/1666/00001666_standalone.jpg
http://www.hellenicdermatlas.com/photos/0000/1667/00001667_standalone.jpg
http://www.hellenicdermatlas.com/photos/0000/1668/00001668_standalone.jpg
http://www.hellenicdermatlas.com/photos/0000/1669/00001669_standalone.jpg
http://www.hellenicdermatlas.com/photos/0000/1670/00001670_standalone.jpg
http://www.hellenicdermatlas.com/photos/0000/1671/00001671_standalone.jpg
http://www.hellenicdermatlas.com/photos/0000/1672/00001672_standalone.jpg
http://www.hellenicdermatlas.com/photos/0000/1674/00001674_standalone.jpg
http://www.hellenicdermatlas.com/photos/0000/1675/00001675_standalone.jpg
http://www.hellenicdermatlas.com/photos/0000/1676/00001676_standalone.jpg
http://www.hellenicdermatlas.com/photos/0000/1677/00001677_standalone.jpg
http://www.hellenicdermatlas.com/photos/0000/1679/00001679_standalone.jpg
http://www.hellenicdermatlas.com/photos/0000/1680/00001680_standalone.jpg
http://www.hellenicdermatlas.com/photos/0000/1681/00001681_standalone.jpg
http://www.hellenicdermatlas.com/photos/0000/1682/00001682_standalone.jpg
http://www.hellenicdermatlas.com/photos/0000/1683/00001683_standalone.jpg
http://www.hellenicdermatlas.com/photos/0000/1684/00001684_standalone.jpg
http://www.hellenicdermatlas.com/photos/0000/1686/00001686_standalone.jpg
http://www.hellenicdermatlas.com/photos/0000/1687/00001687_standalone.jpg
http://www.hellenicdermatlas.com/photos/0000/1688/00001688_standalone.jpg
http://www.hellenicdermatlas.com/photos/0000/1689/00001689_standalone.jpg
http://www.hellenicdermatlas.com/photos/0000/1691/00001691_standalone.jpg
http://www.hellenicdermatlas.com/photos/0000/1693/00001693_standalone.jpg
http://www.hellenicdermatlas.com/photos/0000/1694/00001694_standalone.jpg
http://www.hellenicdermatlas.com/photos/0000/1695/00001695_standalone.jpg
http://www.hellenicdermatlas.com/photos/0000/1698/00001698_standalone.jpg
http://www.hellenicdermatlas.com/photos/0000/1699/00001699_standalone.jpg
http://www.hellenicdermatlas.com/photos/0000/1701/00001701_standalone.jpg
http://www.hellenicdermatlas.com/photos/0000/1702/00001702_standalone.jpg
http://www.hellenicdermatlas.com/photos/0000/1703/00001703_standalone.jpg
http://www.hellenicdermatlas.com/photos/0000/1704/00001704_standalone.jpg
http://www.hellenicdermatlas.com/photos/0000/1705/00001705_standalone.jpg
http://www.hellenicdermatlas.com/photos/0000/1707/00001707_standalone.jpg
http://www.hellenicdermatlas.com/photos/0000/1708/00001708_standalone.jpg
http://www.hellenicdermatlas.com/photos/0000/1709/00001709_standalone.jpg
http://www.hellenicdermatlas.com/photos/0000/1710/00001710_standalone.jpg
http://www.hellenicdermatlas.com/photos/0000/1711/00001711_standalone.jpg
http://www.hellenicdermatlas.com/photos/0000/1712/00001712_standalone.jpg
http://www.hellenicdermatlas.com/photos/0000/1713/00001713_standalone.jpg
http://www.hellenicdermatlas.com/photos/0000/1714/00001714_standalone.jpg
http://www.hellenicdermatlas.com/photos/0000/1715/00001715_standalone.jpg
http://www.hellenicdermatlas.com/photos/0000/1716/00001716_standalone.jpg
http://www.hellenicdermatlas.com/photos/0000/1717/00001717_standalone.jpg
http://www.hellenicdermatlas.com/photos/0000/1718/00001718_standalone.jpg
http://www.hellenicdermatlas.com/photos/0000/1719/00001719_standalone.jpg

http://www.hellenicdermatlas.com/photos/0000/1721/00001721_standalone.jpg
http://www.hellenicdermatlas.com/photos/0000/1722/00001722_standalone.jpg
http://www.hellenicdermatlas.com/photos/0000/1723/00001723_standalone.jpg
http://www.hellenicdermatlas.com/photos/0000/1725/00001725_standalone.jpg
http://www.hellenicdermatlas.com/photos/0000/1726/00001726_standalone.jpg
http://www.hellenicdermatlas.com/photos/0000/1727/00001727_standalone.jpg
http://www.hellenicdermatlas.com/photos/0000/1728/00001728_standalone.jpg
http://www.hellenicdermatlas.com/photos/0000/1729/00001729_standalone.jpg
http://www.hellenicdermatlas.com/photos/0000/1730/00001730_standalone.jpg
http://www.hellenicdermatlas.com/photos/0000/1731/00001731_standalone.jpg
http://www.hellenicdermatlas.com/photos/0000/1732/00001732_standalone.jpg
http://www.hellenicdermatlas.com/photos/0000/1733/00001733_standalone.jpg
http://www.hellenicdermatlas.com/photos/0000/1734/00001734_standalone.jpg
http://www.hellenicdermatlas.com/photos/0000/1735/00001735_standalone.jpg
http://www.hellenicdermatlas.com/photos/0000/1736/00001736_standalone.jpg
http://www.hellenicdermatlas.com/photos/0000/1737/00001737_standalone.jpg
http://www.hellenicdermatlas.com/photos/0000/1739/00001739_standalone.jpg
http://www.hellenicdermatlas.com/photos/0000/1740/00001740_standalone.jpg
http://www.hellenicdermatlas.com/photos/0000/1741/00001741_standalone.jpg
http://www.hellenicdermatlas.com/photos/0000/1742/00001742_standalone.jpg
http://www.hellenicdermatlas.com/photos/0000/1744/00001744_standalone.jpg
http://www.hellenicdermatlas.com/photos/0000/1745/00001745_standalone.jpg
http://www.hellenicdermatlas.com/photos/0000/1747/00001747_standalone.jpg
http://www.hellenicdermatlas.com/photos/0000/1748/00001748_standalone.jpg
http://www.hellenicdermatlas.com/photos/0000/1750/00001750_standalone.jpg
http://www.hellenicdermatlas.com/photos/0000/1752/00001752_standalone.jpg
http://www.hellenicdermatlas.com/photos/0000/1753/00001753_standalone.jpg
http://www.hellenicdermatlas.com/photos/0000/1754/00001754_standalone.jpg
http://www.hellenicdermatlas.com/photos/0000/1756/00001756_standalone.jpg
http://www.hellenicdermatlas.com/photos/0000/1757/00001757_standalone.jpg
http://www.hellenicdermatlas.com/photos/0000/1758/00001758_standalone.jpg
http://www.hellenicdermatlas.com/photos/0000/1759/00001759_standalone.jpg
http://www.hellenicdermatlas.com/photos/0000/1761/00001761_standalone.jpg
http://www.hellenicdermatlas.com/photos/0000/1762/00001762_standalone.jpg
http://www.hellenicdermatlas.com/photos/0000/1763/00001763_standalone.jpg
http://www.hellenicdermatlas.com/photos/0000/1764/00001764_standalone.jpg
http://www.hellenicdermatlas.com/photos/0000/1765/00001765_standalone.jpg
http://www.hellenicdermatlas.com/photos/0000/1766/00001766_standalone.jpg
http://www.hellenicdermatlas.com/photos/0000/1767/00001767_standalone.jpg
http://www.hellenicdermatlas.com/photos/0000/1768/00001768_standalone.jpg
http://www.hellenicdermatlas.com/photos/0000/1769/00001769_standalone.jpg
http://www.hellenicdermatlas.com/photos/0000/1770/00001770_standalone.jpg
http://www.hellenicdermatlas.com/photos/0000/1771/00001771_standalone.jpg
http://www.hellenicdermatlas.com/photos/0000/1772/00001772_standalone.jpg
http://www.hellenicdermatlas.com/photos/0000/1773/00001773_standalone.jpg
http://www.hellenicdermatlas.com/photos/0000/1774/00001774_standalone.jpg
http://www.hellenicdermatlas.com/photos/0000/1775/00001775_standalone.jpg
http://www.hellenicdermatlas.com/photos/0000/1776/00001776_standalone.jpg

http://www.hellenicdermatlas.com/photos/0000/1777/00001777_standalone.jpg
http://www.hellenicdermatlas.com/photos/0000/1778/00001778_standalone.jpg
http://www.hellenicdermatlas.com/photos/0000/1779/00001779_standalone.jpg
http://www.hellenicdermatlas.com/photos/0000/1780/00001780_standalone.jpg
http://www.hellenicdermatlas.com/photos/0000/1781/00001781_standalone.jpg
http://www.hellenicdermatlas.com/photos/0000/1782/00001782_standalone.jpg
http://www.hellenicdermatlas.com/photos/0000/1783/00001783_standalone.jpg
http://www.hellenicdermatlas.com/photos/0000/1784/00001784_standalone.jpg
http://www.hellenicdermatlas.com/photos/0000/1785/00001785_standalone.jpg
http://www.hellenicdermatlas.com/photos/0000/1786/00001786_standalone.jpg
http://www.hellenicdermatlas.com/photos/0000/1787/00001787_standalone.jpg
http://www.hellenicdermatlas.com/photos/0000/1788/00001788_standalone.jpg
http://www.hellenicdermatlas.com/photos/0000/1790/00001790_standalone.jpg
http://www.hellenicdermatlas.com/photos/0000/1791/00001791_standalone.jpg
http://www.hellenicdermatlas.com/photos/0000/1793/00001793_standalone.jpg
http://www.hellenicdermatlas.com/photos/0000/1794/00001794_standalone.jpg
http://www.hellenicdermatlas.com/photos/0000/1797/00001797_standalone.jpg
http://www.hellenicdermatlas.com/photos/0000/1798/00001798_standalone.jpg
http://www.hellenicdermatlas.com/photos/0000/1799/00001799_standalone.jpg
http://www.hellenicdermatlas.com/photos/0000/1800/00001800_standalone.jpg
http://www.hellenicdermatlas.com/photos/0000/1801/00001801_standalone.jpg
http://www.hellenicdermatlas.com/photos/0000/1802/00001802_standalone.jpg
http://www.hellenicdermatlas.com/photos/0000/1803/00001803_standalone.jpg
http://www.hellenicdermatlas.com/photos/0000/1804/00001804_standalone.jpg
http://www.hellenicdermatlas.com/photos/0000/1805/00001805_standalone.jpg
http://www.hellenicdermatlas.com/photos/0000/1807/00001807_standalone.jpg
http://www.hellenicdermatlas.com/photos/0000/1808/00001808_standalone.jpg
http://www.hellenicdermatlas.com/photos/0000/1809/00001809_standalone.jpg
http://www.hellenicdermatlas.com/photos/0000/1810/00001810_standalone.jpg
http://www.hellenicdermatlas.com/photos/0000/1811/00001811_standalone.jpg
http://www.hellenicdermatlas.com/photos/0000/1812/00001812_standalone.jpg
http://www.hellenicdermatlas.com/photos/0000/1813/00001813_standalone.jpg
http://www.hellenicdermatlas.com/photos/0000/1814/00001814_standalone.jpg
http://www.hellenicdermatlas.com/photos/0000/1819/00001819_standalone.jpg
http://www.hellenicdermatlas.com/photos/0000/1820/00001820_standalone.jpg
http://www.hellenicdermatlas.com/photos/0000/1821/00001821_standalone.jpg
http://www.hellenicdermatlas.com/photos/0000/1823/00001823_standalone.jpg
http://www.hellenicdermatlas.com/photos/0000/1824/00001824_standalone.jpg
http://www.hellenicdermatlas.com/photos/0000/1825/00001825_standalone.jpg
http://www.hellenicdermatlas.com/photos/0000/1826/00001826_standalone.jpg
http://www.hellenicdermatlas.com/photos/0000/1827/00001827_standalone.jpg
http://www.hellenicdermatlas.com/photos/0000/1828/00001828_standalone.jpg
http://www.hellenicdermatlas.com/photos/0000/1829/00001829_standalone.jpg
http://www.hellenicdermatlas.com/photos/0000/1830/00001830_standalone.jpg
http://www.hellenicdermatlas.com/photos/0000/1831/00001831_standalone.jpg
http://www.hellenicdermatlas.com/photos/0000/1832/00001832_standalone.jpg
http://www.hellenicdermatlas.com/photos/0000/1833/00001833_standalone.jpg
http://www.hellenicdermatlas.com/photos/0000/1834/00001834_standalone.jpg

http://www.hellenicdermatlas.com/photos/0000/1835/00001835_standalone.jpg
http://www.hellenicdermatlas.com/photos/0000/1836/00001836_standalone.jpg
http://www.hellenicdermatlas.com/photos/0000/1838/00001838_standalone.jpg
http://www.hellenicdermatlas.com/photos/0000/1839/00001839_standalone.jpg
http://www.hellenicdermatlas.com/photos/0000/1840/00001840_standalone.jpg
http://www.hellenicdermatlas.com/photos/0000/1841/00001841_standalone.jpg
http://www.hellenicdermatlas.com/photos/0000/1842/00001842_standalone.jpg
http://www.hellenicdermatlas.com/photos/0000/1843/00001843_standalone.jpg
http://www.hellenicdermatlas.com/photos/0000/1844/00001844_standalone.jpg
http://www.hellenicdermatlas.com/photos/0000/1846/00001846_standalone.jpg
http://www.hellenicdermatlas.com/photos/0000/1847/00001847_standalone.jpg
http://www.hellenicdermatlas.com/photos/0000/1848/00001848_standalone.jpg
http://www.hellenicdermatlas.com/photos/0000/1849/00001849_standalone.jpg
http://www.hellenicdermatlas.com/photos/0000/1850/00001850_standalone.jpg
http://www.hellenicdermatlas.com/photos/0000/1851/00001851_standalone.jpg
http://www.hellenicdermatlas.com/photos/0000/1852/00001852_standalone.jpg
http://www.hellenicdermatlas.com/photos/0000/1853/00001853_standalone.jpg
http://www.hellenicdermatlas.com/photos/0000/1854/00001854_standalone.jpg
http://www.hellenicdermatlas.com/photos/0000/1855/00001855_standalone.jpg
http://www.hellenicdermatlas.com/photos/0000/1856/00001856_standalone.jpg
http://www.hellenicdermatlas.com/photos/0000/1857/00001857_standalone.jpg
http://www.hellenicdermatlas.com/photos/0000/1859/00001859_standalone.jpg
http://www.hellenicdermatlas.com/photos/0000/1860/00001860_standalone.jpg
http://www.hellenicdermatlas.com/photos/0000/1861/00001861_standalone.jpg
http://www.hellenicdermatlas.com/photos/0000/1863/00001863_standalone.jpg
http://www.hellenicdermatlas.com/photos/0000/1864/00001864_standalone.jpg
http://www.hellenicdermatlas.com/photos/0000/1865/00001865_standalone.jpg
http://www.hellenicdermatlas.com/photos/0000/1866/00001866_standalone.jpg
http://www.hellenicdermatlas.com/photos/0000/1867/00001867_standalone.jpg
http://www.hellenicdermatlas.com/photos/0000/1868/00001868_standalone.jpg
http://www.hellenicdermatlas.com/photos/0000/1870/00001870_standalone.jpg
http://www.hellenicdermatlas.com/photos/0000/1871/00001871_standalone.jpg
http://www.hellenicdermatlas.com/photos/0000/1872/00001872_standalone.jpg
http://www.hellenicdermatlas.com/photos/0000/1873/00001873_standalone.jpg
http://www.hellenicdermatlas.com/photos/0000/1874/00001874_standalone.jpg
http://www.hellenicdermatlas.com/photos/0000/1876/00001876_standalone.jpg
http://www.hellenicdermatlas.com/photos/0000/1877/00001877_standalone.jpg
http://www.hellenicdermatlas.com/photos/0000/1878/00001878_standalone.jpg
http://www.hellenicdermatlas.com/photos/0000/1879/00001879_standalone.jpg
http://www.hellenicdermatlas.com/photos/0000/1881/00001881_standalone.jpg
http://www.hellenicdermatlas.com/photos/0000/1882/00001882_standalone.jpg
http://www.hellenicdermatlas.com/photos/0000/1883/00001883_standalone.jpg
http://www.hellenicdermatlas.com/photos/0000/1884/00001884_standalone.jpg
http://www.hellenicdermatlas.com/photos/0000/1885/00001885_standalone.jpg
http://www.hellenicdermatlas.com/photos/0000/1886/00001886_standalone.jpg
http://www.hellenicdermatlas.com/photos/0000/1889/00001889_standalone.jpg
http://www.hellenicdermatlas.com/photos/0000/1890/00001890_standalone.jpg
http://www.hellenicdermatlas.com/photos/0000/1891/00001891_standalone.jpg

http://www.hellenicdermatlas.com/photos/0000/1892/00001892_standalone.jpg
http://www.hellenicdermatlas.com/photos/0000/1893/00001893_standalone.jpg
http://www.hellenicdermatlas.com/photos/0000/1894/00001894_standalone.jpg
http://www.hellenicdermatlas.com/photos/0000/1895/00001895_standalone.jpg
http://www.hellenicdermatlas.com/photos/0000/1896/00001896_standalone.jpg
http://www.hellenicdermatlas.com/photos/0000/1897/00001897_standalone.jpg
http://www.hellenicdermatlas.com/photos/0000/1898/00001898_standalone.jpg
http://www.hellenicdermatlas.com/photos/0000/1899/00001899_standalone.jpg
http://www.hellenicdermatlas.com/photos/0000/1900/00001900_standalone.jpg
http://www.hellenicdermatlas.com/photos/0000/1901/00001901_standalone.jpg
http://www.hellenicdermatlas.com/photos/0000/1902/00001902_standalone.jpg
http://www.hellenicdermatlas.com/photos/0000/1903/00001903_standalone.jpg
http://www.hellenicdermatlas.com/photos/0000/1904/00001904_standalone.jpg
http://www.hellenicdermatlas.com/photos/0000/1905/00001905_standalone.jpg
http://www.hellenicdermatlas.com/photos/0000/1906/00001906_standalone.jpg
http://www.hellenicdermatlas.com/photos/0000/1908/00001908_standalone.jpg
http://www.hellenicdermatlas.com/photos/0000/1909/00001909_standalone.jpg
http://www.hellenicdermatlas.com/photos/0000/1910/00001910_standalone.jpg
http://www.hellenicdermatlas.com/photos/0000/1912/00001912_standalone.jpg
http://www.hellenicdermatlas.com/photos/0000/1913/00001913_standalone.jpg
http://www.hellenicdermatlas.com/photos/0000/1914/00001914_standalone.jpg
http://www.hellenicdermatlas.com/photos/0000/1915/00001915_standalone.jpg
http://www.hellenicdermatlas.com/photos/0000/1919/00001919_standalone.jpg
http://www.hellenicdermatlas.com/photos/0000/1920/00001920_standalone.jpg
http://www.hellenicdermatlas.com/photos/0000/1921/00001921_standalone.jpg
http://www.hellenicdermatlas.com/photos/0000/1922/00001922_standalone.jpg
http://www.hellenicdermatlas.com/photos/0000/1923/00001923_standalone.jpg
http://www.hellenicdermatlas.com/photos/0000/1924/00001924_standalone.jpg
http://www.hellenicdermatlas.com/photos/0000/1925/00001925_standalone.jpg
http://www.hellenicdermatlas.com/photos/0000/1926/00001926_standalone.jpg
http://www.hellenicdermatlas.com/photos/0000/1928/00001928_standalone.jpg
http://www.hellenicdermatlas.com/photos/0000/1929/00001929_standalone.jpg
http://www.hellenicdermatlas.com/photos/0000/1930/00001930_standalone.jpg
http://www.hellenicdermatlas.com/photos/0000/1932/00001932_standalone.jpg
http://www.hellenicdermatlas.com/photos/0000/1933/00001933_standalone.jpg
http://www.hellenicdermatlas.com/photos/0000/1934/00001934_standalone.jpg
http://www.hellenicdermatlas.com/photos/0000/1935/00001935_standalone.jpg
http://www.hellenicdermatlas.com/photos/0000/1936/00001936_standalone.jpg
http://www.hellenicdermatlas.com/photos/0000/1937/00001937_standalone.jpg
http://www.hellenicdermatlas.com/photos/0000/1938/00001938_standalone.jpg
http://www.hellenicdermatlas.com/photos/0000/1939/00001939_standalone.jpg
http://www.hellenicdermatlas.com/photos/0000/1940/00001940_standalone.jpg
http://www.hellenicdermatlas.com/photos/0000/1941/00001941_standalone.jpg
http://www.hellenicdermatlas.com/photos/0000/1942/00001942_standalone.jpg
http://www.hellenicdermatlas.com/photos/0000/1943/00001943_standalone.jpg
http://www.hellenicdermatlas.com/photos/0000/1944/00001944_standalone.jpg
http://www.hellenicdermatlas.com/photos/0000/1945/00001945_standalone.jpg
http://www.hellenicdermatlas.com/photos/0000/1948/00001948_standalone.jpg

http://www.hellenicdermatlas.com/photos/0000/1949/00001949_standalone.jpg
http://www.hellenicdermatlas.com/photos/0000/1950/00001950_standalone.jpg
http://www.hellenicdermatlas.com/photos/0000/1951/00001951_standalone.jpg
http://www.hellenicdermatlas.com/photos/0000/1953/00001953_standalone.jpg
http://www.hellenicdermatlas.com/photos/0000/1954/00001954_standalone.jpg
http://www.hellenicdermatlas.com/photos/0000/1955/00001955_standalone.jpg
http://www.hellenicdermatlas.com/photos/0000/1956/00001956_standalone.jpg
http://www.hellenicdermatlas.com/photos/0000/1957/00001957_standalone.jpg
http://www.hellenicdermatlas.com/photos/0000/1958/00001958_standalone.jpg
http://www.hellenicdermatlas.com/photos/0000/1960/00001960_standalone.jpg
http://www.hellenicdermatlas.com/photos/0000/1961/00001961_standalone.jpg
http://www.hellenicdermatlas.com/photos/0000/1962/00001962_standalone.jpg
http://www.hellenicdermatlas.com/photos/0000/1963/00001963_standalone.jpg
http://www.hellenicdermatlas.com/photos/0000/1964/00001964_standalone.jpg
http://www.hellenicdermatlas.com/photos/0000/1967/00001967_standalone.jpg
http://www.hellenicdermatlas.com/photos/0000/1968/00001968_standalone.jpg
http://www.hellenicdermatlas.com/photos/0000/1969/00001969_standalone.jpg
http://www.hellenicdermatlas.com/photos/0000/1970/00001970_standalone.jpg
http://www.hellenicdermatlas.com/photos/0000/1971/00001971_standalone.jpg
http://www.hellenicdermatlas.com/photos/0000/1972/00001972_standalone.jpg
http://www.hellenicdermatlas.com/photos/0000/1973/00001973_standalone.jpg
http://www.hellenicdermatlas.com/photos/0000/1974/00001974_standalone.jpg
http://www.hellenicdermatlas.com/photos/0000/1977/00001977_standalone.jpg
http://www.hellenicdermatlas.com/photos/0000/1978/00001978_standalone.jpg
http://www.hellenicdermatlas.com/photos/0000/1979/00001979_standalone.jpg
http://www.hellenicdermatlas.com/photos/0000/1980/00001980_standalone.jpg
http://www.hellenicdermatlas.com/photos/0000/1981/00001981_standalone.jpg
http://www.hellenicdermatlas.com/photos/0000/1982/00001982_standalone.jpg
http://www.hellenicdermatlas.com/photos/0000/1984/00001984_standalone.jpg
http://www.hellenicdermatlas.com/photos/0000/1985/00001985_standalone.jpg
http://www.hellenicdermatlas.com/photos/0000/1986/00001986_standalone.jpg
http://www.hellenicdermatlas.com/photos/0000/1987/00001987_standalone.jpg
http://www.hellenicdermatlas.com/photos/0000/1988/00001988_standalone.jpg
http://www.hellenicdermatlas.com/photos/0000/1989/00001989_standalone.jpg
http://www.hellenicdermatlas.com/photos/0000/1990/00001990_standalone.jpg
http://www.hellenicdermatlas.com/photos/0000/1991/00001991_standalone.jpg
http://www.hellenicdermatlas.com/photos/0000/1992/00001992_standalone.jpg
http://www.hellenicdermatlas.com/photos/0000/1993/00001993_standalone.jpg
http://www.hellenicdermatlas.com/photos/0000/1994/00001994_standalone.jpg
http://www.hellenicdermatlas.com/photos/0000/1995/00001995_standalone.jpg
http://www.hellenicdermatlas.com/photos/0000/1997/00001997_standalone.jpg
http://www.hellenicdermatlas.com/photos/0000/1998/00001998_standalone.jpg
http://www.hellenicdermatlas.com/photos/0000/1999/00001999_standalone.jpg
http://www.hellenicdermatlas.com/photos/0000/2000/00002000_standalone.jpg
http://www.hellenicdermatlas.com/photos/0000/2002/00002002_standalone.jpg
http://www.hellenicdermatlas.com/photos/0000/2004/00002004_standalone.jpg
http://www.hellenicdermatlas.com/photos/0000/2005/00002005_standalone.jpg
http://www.hellenicdermatlas.com/photos/0000/2006/00002006_standalone.jpg

http://www.hellenicdermatlas.com/photos/0000/2007/00002007_standalone.jpg
http://www.hellenicdermatlas.com/photos/0000/2008/00002008_standalone.jpg
http://www.hellenicdermatlas.com/photos/0000/2009/00002009_standalone.jpg
http://www.hellenicdermatlas.com/photos/0000/2011/00002011_standalone.jpg
http://www.hellenicdermatlas.com/photos/0000/2012/00002012_standalone.jpg
http://www.hellenicdermatlas.com/photos/0000/2013/00002013_standalone.jpg
http://www.hellenicdermatlas.com/photos/0000/2014/00002014_standalone.jpg
http://www.hellenicdermatlas.com/photos/0000/2015/00002015_standalone.jpg
http://www.hellenicdermatlas.com/photos/0000/2016/00002016_standalone.jpg
http://www.hellenicdermatlas.com/photos/0000/2017/00002017_standalone.jpg
http://www.hellenicdermatlas.com/photos/0000/2018/00002018_standalone.jpg
http://www.hellenicdermatlas.com/photos/0000/2019/00002019_standalone.jpg
http://www.hellenicdermatlas.com/photos/0000/2020/00002020_standalone.jpg
http://www.hellenicdermatlas.com/photos/0000/2021/00002021_standalone.jpg
http://www.hellenicdermatlas.com/photos/0000/2022/00002022_standalone.jpg
http://www.hellenicdermatlas.com/photos/0000/2024/00002024_standalone.jpg
http://www.hellenicdermatlas.com/photos/0000/2025/00002025_standalone.jpg
http://www.hellenicdermatlas.com/photos/0000/2026/00002026_standalone.jpg
http://www.hellenicdermatlas.com/photos/0000/2027/00002027_standalone.jpg
http://www.hellenicdermatlas.com/photos/0000/2028/00002028_standalone.jpg
http://www.hellenicdermatlas.com/photos/0000/2029/00002029_standalone.jpg
http://www.hellenicdermatlas.com/photos/0000/2030/00002030_standalone.jpg
http://www.hellenicdermatlas.com/photos/0000/2031/00002031_standalone.jpg
http://www.hellenicdermatlas.com/photos/0000/2032/00002032_standalone.jpg
http://www.hellenicdermatlas.com/photos/0000/2033/00002033_standalone.jpg
http://www.hellenicdermatlas.com/photos/0000/2035/00002035_standalone.jpg
http://www.hellenicdermatlas.com/photos/0000/2036/00002036_standalone.jpg
http://www.hellenicdermatlas.com/photos/0000/2037/00002037_standalone.jpg
http://www.hellenicdermatlas.com/photos/0000/2038/00002038_standalone.jpg
http://www.hellenicdermatlas.com/photos/0000/2039/00002039_standalone.jpg
http://www.hellenicdermatlas.com/photos/0000/2040/00002040_standalone.jpg
http://www.hellenicdermatlas.com/photos/0000/2041/00002041_standalone.jpg
http://www.hellenicdermatlas.com/photos/0000/2043/00002043_standalone.jpg
http://www.hellenicdermatlas.com/photos/0000/2044/00002044_standalone.jpg
http://www.hellenicdermatlas.com/photos/0000/2045/00002045_standalone.jpg
http://www.hellenicdermatlas.com/photos/0000/2046/00002046_standalone.jpg
http://www.hellenicdermatlas.com/photos/0000/2047/00002047_standalone.jpg
http://www.hellenicdermatlas.com/photos/0000/2049/00002049_standalone.jpg
http://www.hellenicdermatlas.com/photos/0000/2051/00002051_standalone.jpg
http://www.hellenicdermatlas.com/photos/0000/2052/00002052_standalone.jpg
http://www.hellenicdermatlas.com/photos/0000/2053/00002053_standalone.jpg
http://www.hellenicdermatlas.com/photos/0000/2054/00002054_standalone.jpg
http://www.hellenicdermatlas.com/photos/0000/2055/00002055_standalone.jpg
http://www.hellenicdermatlas.com/photos/0000/2056/00002056_standalone.jpg
http://www.hellenicdermatlas.com/photos/0000/2059/00002059_standalone.jpg
http://www.hellenicdermatlas.com/photos/0000/2060/00002060_standalone.jpg
http://www.hellenicdermatlas.com/photos/0000/2061/00002061_standalone.jpg
http://www.hellenicdermatlas.com/photos/0000/2062/00002062_standalone.jpg

http://www.hellenicdermatlas.com/photos/0000/2063/00002063_standalone.jpg
http://www.hellenicdermatlas.com/photos/0000/2064/00002064_standalone.jpg
http://www.hellenicdermatlas.com/photos/0000/2065/00002065_standalone.jpg
http://www.hellenicdermatlas.com/photos/0000/2066/00002066_standalone.jpg
http://www.hellenicdermatlas.com/photos/0000/2070/00002070_standalone.jpg
http://www.hellenicdermatlas.com/photos/0000/2071/00002071_standalone.jpg
http://www.hellenicdermatlas.com/photos/0000/2072/00002072_standalone.jpg
http://www.hellenicdermatlas.com/photos/0000/2076/00002076_standalone.jpg
http://www.hellenicdermatlas.com/photos/0000/2078/00002078_standalone.jpg
http://www.hellenicdermatlas.com/photos/0000/2079/00002079_standalone.jpg
http://www.hellenicdermatlas.com/photos/0000/2080/00002080_standalone.jpg
http://www.hellenicdermatlas.com/photos/0000/2081/00002081_standalone.jpg
http://www.hellenicdermatlas.com/photos/0000/2082/00002082_standalone.jpg
http://www.hellenicdermatlas.com/photos/0000/2083/00002083_standalone.jpg
http://www.hellenicdermatlas.com/photos/0000/2084/00002084_standalone.jpg
http://www.hellenicdermatlas.com/photos/0000/2085/00002085_standalone.jpg
http://www.hellenicdermatlas.com/photos/0000/2087/00002087_standalone.jpg
http://www.hellenicdermatlas.com/photos/0000/2088/00002088_standalone.jpg
http://www.hellenicdermatlas.com/photos/0000/2090/00002090_standalone.jpg
http://www.hellenicdermatlas.com/photos/0000/2091/00002091_standalone.jpg
http://www.hellenicdermatlas.com/photos/0000/2092/00002092_standalone.jpg
http://www.hellenicdermatlas.com/photos/0000/2093/00002093_standalone.jpg
http://www.hellenicdermatlas.com/photos/0000/2094/00002094_standalone.jpg
http://www.hellenicdermatlas.com/photos/0000/2095/00002095_standalone.jpg
http://www.hellenicdermatlas.com/photos/0000/2096/00002096_standalone.jpg
http://www.hellenicdermatlas.com/photos/0000/2097/00002097_standalone.jpg
http://www.hellenicdermatlas.com/photos/0000/2098/00002098_standalone.jpg
http://www.hellenicdermatlas.com/photos/0000/2099/00002099_standalone.jpg
http://www.hellenicdermatlas.com/photos/0000/2102/00002102_standalone.jpg
http://www.hellenicdermatlas.com/photos/0000/2104/00002104_standalone.jpg
http://www.hellenicdermatlas.com/photos/0000/2105/00002105_standalone.jpg
http://www.hellenicdermatlas.com/photos/0000/2106/00002106_standalone.jpg
http://www.hellenicdermatlas.com/photos/0000/2107/00002107_standalone.jpg
http://www.hellenicdermatlas.com/photos/0000/2109/00002109_standalone.jpg
http://www.hellenicdermatlas.com/photos/0000/2110/00002110_standalone.jpg
http://www.hellenicdermatlas.com/photos/0000/2114/00002114_standalone.jpg
http://www.hellenicdermatlas.com/photos/0000/2115/00002115_standalone.jpg
http://www.hellenicdermatlas.com/photos/0000/2116/00002116_standalone.jpg
http://www.hellenicdermatlas.com/photos/0000/2117/00002117_standalone.jpg
http://www.hellenicdermatlas.com/photos/0000/2118/00002118_standalone.jpg
http://www.hellenicdermatlas.com/photos/0000/2120/00002120_standalone.jpg
http://www.hellenicdermatlas.com/photos/0000/2121/00002121_standalone.jpg
http://www.hellenicdermatlas.com/photos/0000/2122/00002122_standalone.jpg
http://www.hellenicdermatlas.com/photos/0000/2123/00002123_standalone.jpg
http://www.hellenicdermatlas.com/photos/0000/2124/00002124_standalone.jpg
http://www.hellenicdermatlas.com/photos/0000/2125/00002125_standalone.jpg
http://www.hellenicdermatlas.com/photos/0000/2127/00002127_standalone.jpg
http://www.hellenicdermatlas.com/photos/0000/2128/00002128_standalone.jpg

http://www.hellenicdermatlas.com/photos/0000/2130/00002130_standalone.jpg
http://www.hellenicdermatlas.com/photos/0000/2131/00002131_standalone.jpg
http://www.hellenicdermatlas.com/photos/0000/2132/00002132_standalone.jpg
http://www.hellenicdermatlas.com/photos/0000/2133/00002133_standalone.jpg
http://www.hellenicdermatlas.com/photos/0000/2137/00002137_standalone.jpg
http://www.hellenicdermatlas.com/photos/0000/2139/00002139_standalone.jpg
http://www.hellenicdermatlas.com/photos/0000/2141/00002141_standalone.jpg
http://www.hellenicdermatlas.com/photos/0000/2142/00002142_standalone.jpg
http://www.hellenicdermatlas.com/photos/0000/2143/00002143_standalone.jpg
http://www.hellenicdermatlas.com/photos/0000/2144/00002144_standalone.jpg
http://www.hellenicdermatlas.com/photos/0000/2145/00002145_standalone.jpg
http://www.hellenicdermatlas.com/photos/0000/2146/00002146_standalone.jpg
http://www.hellenicdermatlas.com/photos/0000/2147/00002147_standalone.jpg
http://www.hellenicdermatlas.com/photos/0000/2148/00002148_standalone.jpg
http://www.hellenicdermatlas.com/photos/0000/2150/00002150_standalone.jpg
http://www.hellenicdermatlas.com/photos/0000/2152/00002152_standalone.jpg
http://www.hellenicdermatlas.com/photos/0000/2153/00002153_standalone.jpg
http://www.hellenicdermatlas.com/photos/0000/2156/00002156_standalone.jpg
http://www.hellenicdermatlas.com/photos/0000/2157/00002157_standalone.jpg
http://www.hellenicdermatlas.com/photos/0000/2158/00002158_standalone.jpg
http://www.hellenicdermatlas.com/photos/0000/2161/00002161_standalone.jpg
http://www.hellenicdermatlas.com/photos/0000/2162/00002162_standalone.jpg
http://www.hellenicdermatlas.com/photos/0000/2163/00002163_standalone.jpg
http://www.hellenicdermatlas.com/photos/0000/2165/00002165_standalone.jpg
http://www.hellenicdermatlas.com/photos/0000/2166/00002166_standalone.jpg
http://www.hellenicdermatlas.com/photos/0000/2167/00002167_standalone.jpg
http://www.hellenicdermatlas.com/photos/0000/2168/00002168_standalone.jpg
http://www.hellenicdermatlas.com/photos/0000/2170/00002170_standalone.jpg
http://www.hellenicdermatlas.com/photos/0000/2171/00002171_standalone.jpg
http://www.hellenicdermatlas.com/photos/0000/2172/00002172_standalone.jpg
http://www.hellenicdermatlas.com/photos/0000/2173/00002173_standalone.jpg
http://www.hellenicdermatlas.com/photos/0000/2174/00002174_standalone.jpg
http://www.hellenicdermatlas.com/photos/0000/2175/00002175_standalone.jpg
http://www.hellenicdermatlas.com/photos/0000/2176/00002176_standalone.jpg
http://www.hellenicdermatlas.com/photos/0000/2177/00002177_standalone.jpg
http://www.hellenicdermatlas.com/photos/0000/2178/00002178_standalone.jpg
http://www.hellenicdermatlas.com/photos/0000/2179/00002179_standalone.jpg
http://www.hellenicdermatlas.com/photos/0000/2180/00002180_standalone.jpg
http://www.hellenicdermatlas.com/photos/0000/2181/00002181_standalone.jpg
http://www.hellenicdermatlas.com/photos/0000/2182/00002182_standalone.jpg
http://www.hellenicdermatlas.com/photos/0000/2183/00002183_standalone.jpg
http://www.hellenicdermatlas.com/photos/0000/2184/00002184_standalone.jpg
http://www.hellenicdermatlas.com/photos/0000/2185/00002185_standalone.jpg
http://www.hellenicdermatlas.com/photos/0000/2187/00002187_standalone.jpg
http://www.hellenicdermatlas.com/photos/0000/2188/00002188_standalone.jpg
http://www.hellenicdermatlas.com/photos/0000/2189/00002189_standalone.jpg
http://www.hellenicdermatlas.com/photos/0000/2190/00002190_standalone.jpg
http://www.hellenicdermatlas.com/photos/0000/2191/00002191_standalone.jpg

http://www.hellenicdermatlas.com/photos/0000/2192/00002192_standalone.jpg
http://www.hellenicdermatlas.com/photos/0000/2193/00002193_standalone.jpg
http://www.hellenicdermatlas.com/photos/0000/2194/00002194_standalone.jpg
http://www.hellenicdermatlas.com/photos/0000/2197/00002197_standalone.jpg
http://www.hellenicdermatlas.com/photos/0000/2198/00002198_standalone.jpg
http://www.hellenicdermatlas.com/photos/0000/2199/00002199_standalone.jpg
http://www.hellenicdermatlas.com/photos/0000/2201/00002201_standalone.jpg
http://www.hellenicdermatlas.com/photos/0000/2202/00002202_standalone.jpg
http://www.hellenicdermatlas.com/photos/0000/2203/00002203_standalone.jpg
http://www.hellenicdermatlas.com/photos/0000/2204/00002204_standalone.jpg
http://www.hellenicdermatlas.com/photos/0000/2205/00002205_standalone.jpg
http://www.hellenicdermatlas.com/photos/0000/2206/00002206_standalone.jpg
http://www.hellenicdermatlas.com/photos/0000/2207/00002207_standalone.jpg
http://www.hellenicdermatlas.com/photos/0000/2208/00002208_standalone.jpg
http://www.hellenicdermatlas.com/photos/0000/2209/00002209_standalone.jpg
http://www.hellenicdermatlas.com/photos/0000/2210/00002210_standalone.jpg
http://www.hellenicdermatlas.com/photos/0000/2211/00002211_standalone.jpg
http://www.hellenicdermatlas.com/photos/0000/2214/00002214_standalone.jpg
http://www.hellenicdermatlas.com/photos/0000/2215/00002215_standalone.jpg
http://www.hellenicdermatlas.com/photos/0000/2216/00002216_standalone.jpg
http://www.hellenicdermatlas.com/photos/0000/2218/00002218_standalone.jpg
http://www.hellenicdermatlas.com/photos/0000/2219/00002219_standalone.jpg
http://www.hellenicdermatlas.com/photos/0000/2220/00002220_standalone.jpg
http://www.hellenicdermatlas.com/photos/0000/2221/00002221_standalone.jpg
http://www.hellenicdermatlas.com/photos/0000/2222/00002222_standalone.jpg
http://www.hellenicdermatlas.com/photos/0000/2223/00002223_standalone.jpg
http://www.hellenicdermatlas.com/photos/0000/2225/00002225_standalone.jpg
http://www.hellenicdermatlas.com/photos/0000/2226/00002226_standalone.jpg
http://www.hellenicdermatlas.com/photos/0000/2227/00002227_standalone.jpg
http://www.hellenicdermatlas.com/photos/0000/2228/00002228_standalone.jpg
http://www.hellenicdermatlas.com/photos/0000/2229/00002229_standalone.jpg
http://www.hellenicdermatlas.com/photos/0000/2230/00002230_standalone.jpg
http://www.hellenicdermatlas.com/photos/0000/2232/00002232_standalone.jpg
http://www.hellenicdermatlas.com/photos/0000/2233/00002233_standalone.jpg
http://www.hellenicdermatlas.com/photos/0000/2236/00002236_standalone.jpg
http://www.hellenicdermatlas.com/photos/0000/2237/00002237_standalone.jpg
http://www.hellenicdermatlas.com/photos/0000/2238/00002238_standalone.jpg
http://www.hellenicdermatlas.com/photos/0000/2239/00002239_standalone.jpg
http://www.hellenicdermatlas.com/photos/0000/2240/00002240_standalone.jpg
http://www.hellenicdermatlas.com/photos/0000/2241/00002241_standalone.jpg
http://www.hellenicdermatlas.com/photos/0000/2242/00002242_standalone.jpg
http://www.hellenicdermatlas.com/photos/0000/2243/00002243_standalone.jpg
http://www.hellenicdermatlas.com/photos/0000/2246/00002246_standalone.jpg
http://www.hellenicdermatlas.com/photos/0000/2247/00002247_standalone.jpg
http://www.hellenicdermatlas.com/photos/0000/2248/00002248_standalone.jpg
http://www.hellenicdermatlas.com/photos/0000/2249/00002249_standalone.jpg
http://www.hellenicdermatlas.com/photos/0000/2250/00002250_standalone.jpg
http://www.hellenicdermatlas.com/photos/0000/2251/00002251_standalone.jpg

http://www.hellenicdermatlas.com/photos/0000/2252/00002252_standalone.jpg
http://www.hellenicdermatlas.com/photos/0000/2254/00002254_standalone.jpg
http://www.hellenicdermatlas.com/photos/0000/2255/00002255_standalone.jpg
http://www.hellenicdermatlas.com/photos/0000/2256/00002256_standalone.jpg
http://www.hellenicdermatlas.com/photos/0000/2257/00002257_standalone.jpg
http://www.hellenicdermatlas.com/photos/0000/2258/00002258_standalone.jpg
http://www.hellenicdermatlas.com/photos/0000/2259/00002259_standalone.jpg
http://www.hellenicdermatlas.com/photos/0000/2262/00002262_standalone.jpg
http://www.hellenicdermatlas.com/photos/0000/2263/00002263_standalone.jpg
http://www.hellenicdermatlas.com/photos/0000/2265/00002265_standalone.jpg
http://www.hellenicdermatlas.com/photos/0000/2266/00002266_standalone.jpg
http://www.hellenicdermatlas.com/photos/0000/2267/00002267_standalone.jpg
http://www.hellenicdermatlas.com/photos/0000/2268/00002268_standalone.jpg
http://www.hellenicdermatlas.com/photos/0000/2269/00002269_standalone.jpg
http://www.hellenicdermatlas.com/photos/0000/2277/00002277_standalone.jpg
http://www.hellenicdermatlas.com/photos/0000/2278/00002278_standalone.jpg
http://www.hellenicdermatlas.com/photos/0000/2279/00002279_standalone.jpg
http://www.hellenicdermatlas.com/photos/0000/2281/00002281_standalone.jpg
http://www.hellenicdermatlas.com/photos/0000/2282/00002282_standalone.jpg
http://www.hellenicdermatlas.com/photos/0000/2283/00002283_standalone.jpg
http://www.hellenicdermatlas.com/photos/0000/2284/00002284_standalone.jpg
http://www.hellenicdermatlas.com/photos/0000/2285/00002285_standalone.jpg
http://www.hellenicdermatlas.com/photos/0000/2287/00002287_standalone.jpg
http://www.hellenicdermatlas.com/photos/0000/2288/00002288_standalone.jpg
http://www.hellenicdermatlas.com/photos/0000/2290/00002290_standalone.jpg
http://www.hellenicdermatlas.com/photos/0000/2291/00002291_standalone.jpg
http://www.hellenicdermatlas.com/photos/0000/2292/00002292_standalone.jpg
http://www.hellenicdermatlas.com/photos/0000/2295/00002295_standalone.jpg
http://www.hellenicdermatlas.com/photos/0000/2296/00002296_standalone.jpg
http://www.hellenicdermatlas.com/photos/0000/2297/00002297_standalone.jpg
http://www.hellenicdermatlas.com/photos/0000/2298/00002298_standalone.jpg
http://www.hellenicdermatlas.com/photos/0000/2299/00002299_standalone.jpg
http://www.hellenicdermatlas.com/photos/0000/2301/00002301_standalone.jpg
http://www.hellenicdermatlas.com/photos/0000/2302/00002302_standalone.jpg
http://www.hellenicdermatlas.com/photos/0000/2303/00002303_standalone.jpg
http://www.hellenicdermatlas.com/photos/0000/2304/00002304_standalone.jpg
http://www.hellenicdermatlas.com/photos/0000/2306/00002306_standalone.jpg
http://www.hellenicdermatlas.com/photos/0000/2307/00002307_standalone.jpg
http://www.hellenicdermatlas.com/photos/0000/2308/00002308_standalone.jpg
http://www.hellenicdermatlas.com/photos/0000/2309/00002309_standalone.jpg
http://www.hellenicdermatlas.com/photos/0000/2312/00002312_standalone.jpg
http://www.hellenicdermatlas.com/photos/0000/2313/00002313_standalone.jpg
http://www.hellenicdermatlas.com/photos/0000/2314/00002314_standalone.jpg
http://www.hellenicdermatlas.com/photos/0000/2315/00002315_standalone.jpg
http://www.hellenicdermatlas.com/photos/0000/2317/00002317_standalone.jpg
http://www.hellenicdermatlas.com/photos/0000/2320/00002320_standalone.jpg
http://www.hellenicdermatlas.com/photos/0000/2322/00002322_standalone.jpg
http://www.hellenicdermatlas.com/photos/0000/2323/00002323_standalone.jpg

http://www.hellenicdermatlas.com/photos/0000/2324/00002324_standalone.jpg
http://www.hellenicdermatlas.com/photos/0000/2325/00002325_standalone.jpg
http://www.hellenicdermatlas.com/photos/0000/2327/00002327_standalone.jpg
http://www.hellenicdermatlas.com/photos/0000/2328/00002328_standalone.jpg
http://www.hellenicdermatlas.com/photos/0000/2329/00002329_standalone.jpg
http://www.hellenicdermatlas.com/photos/0000/2330/00002330_standalone.jpg
http://www.hellenicdermatlas.com/photos/0000/2331/00002331_standalone.jpg
http://www.hellenicdermatlas.com/photos/0000/2332/00002332_standalone.jpg
http://www.hellenicdermatlas.com/photos/0000/2333/00002333_standalone.jpg
http://www.hellenicdermatlas.com/photos/0000/2337/00002337_standalone.jpg
http://www.hellenicdermatlas.com/photos/0000/2338/00002338_standalone.jpg
http://www.hellenicdermatlas.com/photos/0000/2339/00002339_standalone.jpg
http://www.hellenicdermatlas.com/photos/0000/2341/00002341_standalone.jpg
http://www.hellenicdermatlas.com/photos/0000/2342/00002342_standalone.jpg
http://www.hellenicdermatlas.com/photos/0000/2343/00002343_standalone.jpg
http://www.hellenicdermatlas.com/photos/0000/2345/00002345_standalone.jpg
http://www.hellenicdermatlas.com/photos/0000/2346/00002346_standalone.jpg
http://www.hellenicdermatlas.com/photos/0000/2348/00002348_standalone.jpg
http://www.hellenicdermatlas.com/photos/0000/2349/00002349_standalone.jpg
http://www.hellenicdermatlas.com/photos/0000/2350/00002350_standalone.jpg
http://www.hellenicdermatlas.com/photos/0000/2351/00002351_standalone.jpg
http://www.hellenicdermatlas.com/photos/0000/2352/00002352_standalone.jpg
http://www.hellenicdermatlas.com/photos/0000/2355/00002355_standalone.jpg
http://www.hellenicdermatlas.com/photos/0000/2356/00002356_standalone.jpg
http://www.hellenicdermatlas.com/photos/0000/2357/00002357_standalone.jpg
http://www.hellenicdermatlas.com/photos/0000/2358/00002358_standalone.jpg
http://www.hellenicdermatlas.com/photos/0000/2359/00002359_standalone.jpg
http://www.hellenicdermatlas.com/photos/0000/2360/00002360_standalone.jpg
http://www.hellenicdermatlas.com/photos/0000/2362/00002362_standalone.jpg
http://www.hellenicdermatlas.com/photos/0000/2364/00002364_standalone.jpg
http://www.hellenicdermatlas.com/photos/0000/2366/00002366_standalone.jpg
http://www.hellenicdermatlas.com/photos/0000/2368/00002368_standalone.jpg
http://www.hellenicdermatlas.com/photos/0000/2369/00002369_standalone.jpg
http://www.hellenicdermatlas.com/photos/0000/2370/00002370_standalone.jpg
http://www.hellenicdermatlas.com/photos/0000/2371/00002371_standalone.jpg
http://www.hellenicdermatlas.com/photos/0000/2372/00002372_standalone.jpg
http://www.hellenicdermatlas.com/photos/0000/2400/00002400_standalone.jpg
http://www.hellenicdermatlas.com/photos/0000/2401/00002401_standalone.jpg
http://www.hellenicdermatlas.com/photos/0000/2402/00002402_standalone.jpg
http://www.hellenicdermatlas.com/photos/0000/2403/00002403_standalone.jpg
http://www.hellenicdermatlas.com/photos/0000/2404/00002404_standalone.jpg
http://www.hellenicdermatlas.com/photos/0000/2405/00002405_standalone.jpg
http://www.hellenicdermatlas.com/photos/0000/2406/00002406_standalone.jpg
http://www.hellenicdermatlas.com/photos/0000/2407/00002407_standalone.jpg
http://www.hellenicdermatlas.com/photos/0000/2408/00002408_standalone.jpg
http://www.hellenicdermatlas.com/photos/0000/2409/00002409_standalone.jpg
http://www.hellenicdermatlas.com/photos/0000/2410/00002410_standalone.jpg
http://www.hellenicdermatlas.com/photos/0000/2411/00002411_standalone.jpg

http://www.hellenicdermatlas.com/photos/0000/2412/00002412_standalone.jpg
http://www.hellenicdermatlas.com/photos/0000/2413/00002413_standalone.jpg
http://www.hellenicdermatlas.com/photos/0000/2414/00002414_standalone.jpg
http://www.hellenicdermatlas.com/photos/0000/2416/00002416_standalone.jpg
http://www.hellenicdermatlas.com/photos/0000/2417/00002417_standalone.jpg
http://www.hellenicdermatlas.com/photos/0000/2418/00002418_standalone.jpg
http://www.hellenicdermatlas.com/photos/0000/2419/00002419_standalone.jpg
http://www.hellenicdermatlas.com/photos/0000/2420/00002420_standalone.jpg
http://www.hellenicdermatlas.com/photos/0000/2421/00002421_standalone.jpg
http://www.hellenicdermatlas.com/photos/0000/2422/00002422_standalone.jpg
http://www.hellenicdermatlas.com/photos/0000/2423/00002423_standalone.jpg
http://www.hellenicdermatlas.com/photos/0000/2424/00002424_standalone.jpg
http://www.hellenicdermatlas.com/photos/0000/2425/00002425_standalone.jpg
http://www.hellenicdermatlas.com/photos/0000/2426/00002426_standalone.jpg
http://www.hellenicdermatlas.com/photos/0000/2427/00002427_standalone.jpg
http://www.hellenicdermatlas.com/photos/0000/2428/00002428_standalone.jpg
http://www.hellenicdermatlas.com/photos/0000/2429/00002429_standalone.jpg
http://www.hellenicdermatlas.com/photos/0000/2430/00002430_standalone.jpg
http://www.hellenicdermatlas.com/photos/0000/2432/00002432_standalone.jpg
http://www.hellenicdermatlas.com/photos/0000/2433/00002433_standalone.jpg
http://www.hellenicdermatlas.com/photos/0000/2435/00002435_standalone.jpg
http://www.hellenicdermatlas.com/photos/0000/2436/00002436_standalone.jpg
http://www.hellenicdermatlas.com/photos/0000/2437/00002437_standalone.jpg
http://www.hellenicdermatlas.com/photos/0000/2438/00002438_standalone.jpg
http://www.hellenicdermatlas.com/photos/0000/2439/00002439_standalone.jpg
http://www.hellenicdermatlas.com/photos/0000/2440/00002440_standalone.jpg
http://www.hellenicdermatlas.com/photos/0000/2441/00002441_standalone.jpg
http://www.hellenicdermatlas.com/photos/0000/2442/00002442_standalone.jpg
http://www.hellenicdermatlas.com/photos/0000/2443/00002443_standalone.jpg
http://www.hellenicdermatlas.com/photos/0000/2444/00002444_standalone.jpg
http://www.hellenicdermatlas.com/photos/0000/2445/00002445_standalone.jpg
http://www.hellenicdermatlas.com/photos/0000/2446/00002446_standalone.jpg
http://www.hellenicdermatlas.com/photos/0000/2447/00002447_standalone.jpg
http://www.hellenicdermatlas.com/photos/0000/2448/00002448_standalone.jpg
http://www.hellenicdermatlas.com/photos/0000/2449/00002449_standalone.jpg
http://www.hellenicdermatlas.com/photos/0000/2450/00002450_standalone.jpg
http://www.hellenicdermatlas.com/photos/0000/2451/00002451_standalone.jpg
http://www.hellenicdermatlas.com/photos/0000/2452/00002452_standalone.jpg
http://www.hellenicdermatlas.com/photos/0000/2453/00002453_standalone.jpg
http://www.hellenicdermatlas.com/photos/0000/2454/00002454_standalone.jpg
http://www.hellenicdermatlas.com/photos/0000/2460/00002460_standalone.jpg
http://www.hellenicdermatlas.com/photos/0000/2461/00002461_standalone.jpg
http://www.hellenicdermatlas.com/photos/0000/2462/00002462_standalone.jpg
http://www.hellenicdermatlas.com/photos/0000/2463/00002463_standalone.jpg
http://www.hellenicdermatlas.com/photos/0000/2464/00002464_standalone.jpg
http://www.hellenicdermatlas.com/photos/0000/2465/00002465_standalone.jpg
http://www.hellenicdermatlas.com/photos/0000/2466/00002466_standalone.jpg
http://www.hellenicdermatlas.com/photos/0000/2467/00002467_standalone.jpg

http://www.hellenicdermatlas.com/photos/0000/2468/00002468_standalone.jpg
http://www.hellenicdermatlas.com/photos/0000/2469/00002469_standalone.jpg
http://www.hellenicdermatlas.com/photos/0000/2470/00002470_standalone.jpg
http://www.hellenicdermatlas.com/photos/0000/2471/00002471_standalone.jpg
http://www.hellenicdermatlas.com/photos/0000/2472/00002472_standalone.jpg
http://www.hellenicdermatlas.com/photos/0000/2473/00002473_standalone.jpg
http://www.hellenicdermatlas.com/photos/0000/2474/00002474_standalone.jpg
http://www.hellenicdermatlas.com/photos/0000/2476/00002476_standalone.jpg
http://www.hellenicdermatlas.com/photos/0000/2477/00002477_standalone.jpg
http://www.hellenicdermatlas.com/photos/0000/2478/00002478_standalone.jpg
http://www.hellenicdermatlas.com/photos/0000/2479/00002479_standalone.jpg
http://www.hellenicdermatlas.com/photos/0000/2480/00002480_standalone.jpg
http://www.hellenicdermatlas.com/photos/0000/2481/00002481_standalone.jpg
http://www.hellenicdermatlas.com/photos/0000/2482/00002482_standalone.jpg
http://www.hellenicdermatlas.com/photos/0000/2483/00002483_standalone.jpg
http://www.hellenicdermatlas.com/photos/0000/2484/00002484_standalone.jpg
http://www.hellenicdermatlas.com/photos/0000/2485/00002485_standalone.jpg
http://www.hellenicdermatlas.com/photos/0000/2486/00002486_standalone.jpg
http://www.hellenicdermatlas.com/photos/0000/2487/00002487_standalone.jpg
http://www.hellenicdermatlas.com/photos/0000/2488/00002488_standalone.jpg
http://www.hellenicdermatlas.com/photos/0000/2489/00002489_standalone.jpg
http://www.hellenicdermatlas.com/photos/0000/2490/00002490_standalone.jpg
http://www.hellenicdermatlas.com/photos/0000/2491/00002491_standalone.jpg
http://www.hellenicdermatlas.com/photos/0000/2492/00002492_standalone.jpg
http://www.hellenicdermatlas.com/photos/0000/2493/00002493_standalone.jpg
http://www.hellenicdermatlas.com/photos/0000/2494/00002494_standalone.jpg
http://www.hellenicdermatlas.com/photos/0000/2495/00002495_standalone.jpg
http://www.hellenicdermatlas.com/photos/0000/2496/00002496_standalone.jpg
http://www.hellenicdermatlas.com/photos/0000/2497/00002497_standalone.jpg
http://www.hellenicdermatlas.com/photos/0000/2498/00002498_standalone.jpg
http://www.hellenicdermatlas.com/photos/0000/2499/00002499_standalone.jpg
http://www.hellenicdermatlas.com/photos/0000/2500/00002500_standalone.jpg
http://www.hellenicdermatlas.com/photos/0000/2501/00002501_standalone.jpg
http://www.hellenicdermatlas.com/photos/0000/2502/00002502_standalone.jpg
http://www.hellenicdermatlas.com/photos/0000/2503/00002503_standalone.jpg
http://www.hellenicdermatlas.com/photos/0000/2504/00002504_standalone.jpg
http://www.hellenicdermatlas.com/photos/0000/2505/00002505_standalone.jpg
http://www.hellenicdermatlas.com/photos/0000/2506/00002506_standalone.jpg
http://www.hellenicdermatlas.com/photos/0000/2507/00002507_standalone.jpg
http://www.hellenicdermatlas.com/photos/0000/2508/00002508_standalone.jpg
http://www.hellenicdermatlas.com/photos/0000/2509/00002509_standalone.jpg
http://www.hellenicdermatlas.com/photos/0000/2510/00002510_standalone.jpg
http://www.hellenicdermatlas.com/photos/0000/2511/00002511_standalone.jpg
http://www.hellenicdermatlas.com/photos/0000/2512/00002512_standalone.jpg
http://www.hellenicdermatlas.com/photos/0000/2513/00002513_standalone.jpg
http://www.hellenicdermatlas.com/photos/0000/2514/00002514_standalone.jpg
http://www.hellenicdermatlas.com/photos/0000/2515/00002515_standalone.jpg
http://www.hellenicdermatlas.com/photos/0000/2516/00002516_standalone.jpg

http://www.hellenicdermatlas.com/photos/0000/2517/00002517_standalone.jpg
http://www.hellenicdermatlas.com/photos/0000/2518/00002518_standalone.jpg
http://www.hellenicdermatlas.com/photos/0000/2519/00002519_standalone.jpg
http://www.hellenicdermatlas.com/photos/0000/2520/00002520_standalone.jpg
http://www.hellenicdermatlas.com/photos/0000/2521/00002521_standalone.jpg
http://www.hellenicdermatlas.com/photos/0000/2522/00002522_standalone.jpg
http://www.hellenicdermatlas.com/photos/0000/2523/00002523_standalone.jpg
http://www.hellenicdermatlas.com/photos/0000/2524/00002524_standalone.jpg
http://www.hellenicdermatlas.com/photos/0000/2525/00002525_standalone.jpg
http://www.hellenicdermatlas.com/photos/0000/2526/00002526_standalone.jpg
http://www.hellenicdermatlas.com/photos/0000/2527/00002527_standalone.jpg
http://www.hellenicdermatlas.com/photos/0000/2528/00002528_standalone.jpg
http://www.hellenicdermatlas.com/photos/0000/2529/00002529_standalone.jpg
http://www.hellenicdermatlas.com/photos/0000/2530/00002530_standalone.jpg
http://www.hellenicdermatlas.com/photos/0000/2531/00002531_standalone.jpg
http://www.hellenicdermatlas.com/photos/0000/2532/00002532_standalone.jpg
http://www.hellenicdermatlas.com/photos/0000/2533/00002533_standalone.jpg
http://www.hellenicdermatlas.com/photos/0000/2534/00002534_standalone.jpg
http://www.hellenicdermatlas.com/photos/0000/2535/00002535_standalone.jpg
http://www.hellenicdermatlas.com/photos/0000/2536/00002536_standalone.jpg
http://www.hellenicdermatlas.com/photos/0000/2537/00002537_standalone.jpg
http://www.hellenicdermatlas.com/photos/0000/2538/00002538_standalone.jpg
http://www.hellenicdermatlas.com/photos/0000/2539/00002539_standalone.jpg
http://www.hellenicdermatlas.com/photos/0000/2540/00002540_standalone.jpg
http://www.hellenicdermatlas.com/photos/0000/2541/00002541_standalone.jpg
http://www.hellenicdermatlas.com/photos/0000/2542/00002542_standalone.jpg
http://www.hellenicdermatlas.com/photos/0000/2543/00002543_standalone.jpg
http://www.hellenicdermatlas.com/photos/0000/2544/00002544_standalone.jpg
http://www.hellenicdermatlas.com/photos/0000/2545/00002545_standalone.jpg
http://www.hellenicdermatlas.com/photos/0000/2546/00002546_standalone.jpg
http://www.hellenicdermatlas.com/photos/0000/2547/00002547_standalone.jpg
http://www.hellenicdermatlas.com/photos/0000/2548/00002548_standalone.jpg
http://www.hellenicdermatlas.com/photos/0000/2549/00002549_standalone.jpg
http://www.hellenicdermatlas.com/photos/0000/2550/00002550_standalone.jpg
http://www.hellenicdermatlas.com/photos/0000/2551/00002551_standalone.jpg
http://www.hellenicdermatlas.com/photos/0000/2552/00002552_standalone.jpg
http://www.hellenicdermatlas.com/photos/0000/2553/00002553_standalone.jpg
http://www.hellenicdermatlas.com/photos/0000/2554/00002554_standalone.jpg
http://www.hellenicdermatlas.com/photos/0000/2555/00002555_standalone.jpg
http://www.hellenicdermatlas.com/photos/0000/2556/00002556_standalone.jpg
http://www.hellenicdermatlas.com/photos/0000/2557/00002557_standalone.jpg
http://www.hellenicdermatlas.com/photos/0000/2558/00002558_standalone.jpg
http://www.hellenicdermatlas.com/photos/0000/2559/00002559_standalone.jpg
http://www.hellenicdermatlas.com/photos/0000/2560/00002560_standalone.jpg
http://www.hellenicdermatlas.com/photos/0000/2561/00002561_standalone.jpg
http://www.hellenicdermatlas.com/photos/0000/2562/00002562_standalone.jpg
http://www.hellenicdermatlas.com/photos/0000/2563/00002563_standalone.jpg
http://www.hellenicdermatlas.com/photos/0000/2564/00002564_standalone.jpg

http://www.hellenicdermatlas.com/photos/0000/2565/00002565_standalone.jpg
http://www.hellenicdermatlas.com/photos/0000/2566/00002566_standalone.jpg
http://www.hellenicdermatlas.com/photos/0000/2567/00002567_standalone.jpg
http://www.hellenicdermatlas.com/photos/0000/2568/00002568_standalone.jpg
http://www.hellenicdermatlas.com/photos/0000/2569/00002569_standalone.jpg
http://www.hellenicdermatlas.com/photos/0000/2570/00002570_standalone.jpg
http://www.hellenicdermatlas.com/photos/0000/2571/00002571_standalone.jpg
http://www.hellenicdermatlas.com/photos/0000/2572/00002572_standalone.jpg
http://www.hellenicdermatlas.com/photos/0000/2573/00002573_standalone.jpg
http://www.hellenicdermatlas.com/photos/0000/2575/00002575_standalone.jpg
http://www.hellenicdermatlas.com/photos/0000/2576/00002576_standalone.jpg
http://www.hellenicdermatlas.com/photos/0000/2577/00002577_standalone.jpg
http://www.hellenicdermatlas.com/photos/0000/2578/00002578_standalone.jpg
http://www.hellenicdermatlas.com/photos/0000/2579/00002579_standalone.jpg
http://www.hellenicdermatlas.com/photos/0000/2580/00002580_standalone.jpg
http://www.hellenicdermatlas.com/photos/0000/2581/00002581_standalone.jpg
http://www.hellenicdermatlas.com/photos/0000/2582/00002582_standalone.jpg
http://www.hellenicdermatlas.com/photos/0000/2583/00002583_standalone.jpg
http://www.hellenicdermatlas.com/photos/0000/2584/00002584_standalone.jpg
http://www.hellenicdermatlas.com/photos/0000/2585/00002585_standalone.jpg
http://www.hellenicdermatlas.com/photos/0000/2586/00002586_standalone.jpg
http://www.hellenicdermatlas.com/photos/0000/2587/00002587_standalone.jpg
http://www.hellenicdermatlas.com/photos/0000/2588/00002588_standalone.jpg
http://www.hellenicdermatlas.com/photos/0000/2589/00002589_standalone.jpg
http://www.hellenicdermatlas.com/photos/0000/2590/00002590_standalone.jpg
http://www.hellenicdermatlas.com/photos/0000/2591/00002591_standalone.jpg
http://www.hellenicdermatlas.com/photos/0000/2592/00002592_standalone.jpg
http://www.hellenicdermatlas.com/photos/0000/2594/00002594_standalone.jpg
http://www.hellenicdermatlas.com/photos/0000/2595/00002595_standalone.jpg
http://www.hellenicdermatlas.com/photos/0000/2596/00002596_standalone.jpg
http://www.hellenicdermatlas.com/photos/0000/2597/00002597_standalone.jpg
http://www.hellenicdermatlas.com/photos/0000/2598/00002598_standalone.jpg
http://www.hellenicdermatlas.com/photos/0000/2599/00002599_standalone.jpg
http://www.hellenicdermatlas.com/photos/0000/2601/00002601_standalone.jpg
http://www.hellenicdermatlas.com/photos/0000/2602/00002602_standalone.jpg
http://www.hellenicdermatlas.com/photos/0000/2603/00002603_standalone.jpg
http://www.hellenicdermatlas.com/photos/0000/2604/00002604_standalone.jpg
http://www.hellenicdermatlas.com/photos/0000/2605/00002605_standalone.jpg
http://www.hellenicdermatlas.com/photos/0000/2606/00002606_standalone.jpg
http://www.hellenicdermatlas.com/photos/0000/2607/00002607_standalone.jpg
http://www.hellenicdermatlas.com/photos/0000/2608/00002608_standalone.jpg
http://www.hellenicdermatlas.com/photos/0000/2609/00002609_standalone.jpg
http://www.hellenicdermatlas.com/photos/0000/2610/00002610_standalone.jpg
http://www.hellenicdermatlas.com/photos/0000/2611/00002611_standalone.jpg
http://www.hellenicdermatlas.com/photos/0000/2612/00002612_standalone.jpg
http://www.hellenicdermatlas.com/photos/0000/2613/00002613_standalone.jpg
http://www.hellenicdermatlas.com/photos/0000/2614/00002614_standalone.jpg
http://www.hellenicdermatlas.com/photos/0000/2615/00002615_standalone.jpg

http://www.hellenicdermatlas.com/photos/0000/2616/00002616_standalone.jpg
http://www.hellenicdermatlas.com/photos/0000/2617/00002617_standalone.jpg
http://www.hellenicdermatlas.com/photos/0000/2618/00002618_standalone.jpg
http://www.hellenicdermatlas.com/photos/0000/2619/00002619_standalone.jpg
http://www.hellenicdermatlas.com/photos/0000/2620/00002620_standalone.jpg
http://www.hellenicdermatlas.com/photos/0000/2621/00002621_standalone.jpg
http://www.hellenicdermatlas.com/photos/0000/2622/00002622_standalone.jpg
http://www.hellenicdermatlas.com/photos/0000/2623/00002623_standalone.jpg
http://www.hellenicdermatlas.com/photos/0000/2624/00002624_standalone.jpg
http://www.hellenicdermatlas.com/photos/0000/2625/00002625_standalone.jpg
http://www.hellenicdermatlas.com/photos/0000/2626/00002626_standalone.jpg
http://www.hellenicdermatlas.com/photos/0000/2627/00002627_standalone.jpg
http://www.hellenicdermatlas.com/photos/0000/2628/00002628_standalone.jpg
http://www.hellenicdermatlas.com/photos/0000/2629/00002629_standalone.jpg
http://www.hellenicdermatlas.com/photos/0000/2630/00002630_standalone.jpg
http://www.hellenicdermatlas.com/photos/0000/2631/00002631_standalone.jpg
http://www.hellenicdermatlas.com/photos/0000/2632/00002632_standalone.jpg
http://www.hellenicdermatlas.com/photos/0000/2633/00002633_standalone.jpg
http://www.hellenicdermatlas.com/photos/0000/2634/00002634_standalone.jpg
http://www.hellenicdermatlas.com/photos/0000/2635/00002635_standalone.jpg
http://www.hellenicdermatlas.com/photos/0000/2636/00002636_standalone.jpg
http://www.hellenicdermatlas.com/photos/0000/2637/00002637_standalone.jpg
http://www.hellenicdermatlas.com/photos/0000/2638/00002638_standalone.jpg
http://www.hellenicdermatlas.com/photos/0000/2639/00002639_standalone.jpg
http://www.hellenicdermatlas.com/photos/0000/2640/00002640_standalone.jpg
http://www.hellenicdermatlas.com/photos/0000/2641/00002641_standalone.jpg
http://www.hellenicdermatlas.com/photos/0000/2642/00002642_standalone.jpg
http://www.hellenicdermatlas.com/photos/0000/2643/00002643_standalone.jpg
http://www.hellenicdermatlas.com/photos/0000/2644/00002644_standalone.jpg
http://www.hellenicdermatlas.com/photos/0000/2645/00002645_standalone.jpg
http://www.hellenicdermatlas.com/photos/0000/2646/00002646_standalone.jpg
http://www.hellenicdermatlas.com/photos/0000/2647/00002647_standalone.jpg
http://www.hellenicdermatlas.com/photos/0000/2648/00002648_standalone.jpg
http://www.hellenicdermatlas.com/photos/0000/2649/00002649_standalone.jpg
http://www.hellenicdermatlas.com/photos/0000/2650/00002650_standalone.jpg
http://www.hellenicdermatlas.com/photos/0000/2651/00002651_standalone.jpg
http://www.hellenicdermatlas.com/photos/0000/2652/00002652_standalone.jpg
http://www.hellenicdermatlas.com/photos/0000/2653/00002653_standalone.jpg
http://www.hellenicdermatlas.com/photos/0000/2654/00002654_standalone.jpg
http://www.hellenicdermatlas.com/photos/0000/2655/00002655_standalone.jpg
http://www.hellenicdermatlas.com/photos/0000/2656/00002656_standalone.jpg
http://www.hellenicdermatlas.com/photos/0000/2657/00002657_standalone.jpg
http://www.hellenicdermatlas.com/photos/0000/2658/00002658_standalone.jpg
http://www.hellenicdermatlas.com/photos/0000/2659/00002659_standalone.jpg
http://www.hellenicdermatlas.com/photos/0000/2660/00002660_standalone.jpg
http://www.hellenicdermatlas.com/photos/0000/2661/00002661_standalone.jpg
http://www.hellenicdermatlas.com/photos/0000/2662/00002662_standalone.jpg
http://www.hellenicdermatlas.com/photos/0000/2663/00002663_standalone.jpg

http://www.hellenicdermatlas.com/photos/0000/2664/00002664_standalone.jpg
http://www.hellenicdermatlas.com/photos/0000/2665/00002665_standalone.jpg
http://www.hellenicdermatlas.com/photos/0000/2666/00002666_standalone.jpg
http://www.hellenicdermatlas.com/photos/0000/2667/00002667_standalone.jpg
http://www.hellenicdermatlas.com/photos/0000/2676/00002676_standalone.jpg
http://www.hellenicdermatlas.com/photos/0000/2677/00002677_standalone.jpg
http://www.hellenicdermatlas.com/photos/0000/2678/00002678_standalone.jpg
http://www.hellenicdermatlas.com/photos/0000/2679/00002679_standalone.jpg
http://www.hellenicdermatlas.com/photos/0000/2680/00002680_standalone.jpg
http://www.hellenicdermatlas.com/photos/0000/2681/00002681_standalone.jpg
http://www.hellenicdermatlas.com/photos/0000/2682/00002682_standalone.jpg
http://www.hellenicdermatlas.com/photos/0000/2683/00002683_standalone.jpg
http://www.hellenicdermatlas.com/photos/0000/2684/00002684_standalone.jpg
http://www.hellenicdermatlas.com/photos/0000/2685/00002685_standalone.jpg
http://www.hellenicdermatlas.com/photos/0000/2686/00002686_standalone.jpg
http://www.hellenicdermatlas.com/photos/0000/2687/00002687_standalone.jpg
http://www.hellenicdermatlas.com/photos/0000/2688/00002688_standalone.jpg
http://www.hellenicdermatlas.com/photos/0000/2689/00002689_standalone.jpg
http://www.hellenicdermatlas.com/photos/0000/2690/00002690_standalone.jpg
http://www.hellenicdermatlas.com/photos/0000/2691/00002691_standalone.jpg
http://www.hellenicdermatlas.com/photos/0000/2692/00002692_standalone.jpg
http://www.hellenicdermatlas.com/photos/0000/2693/00002693_standalone.jpg
http://www.hellenicdermatlas.com/photos/0000/2694/00002694_standalone.jpg
http://www.hellenicdermatlas.com/photos/0000/2695/00002695_standalone.jpg
http://www.hellenicdermatlas.com/photos/0000/2696/00002696_standalone.jpg
http://www.hellenicdermatlas.com/photos/0000/2697/00002697_standalone.jpg
http://www.hellenicdermatlas.com/photos/0000/2698/00002698_standalone.jpg
http://www.hellenicdermatlas.com/photos/0000/2699/00002699_standalone.jpg
http://www.hellenicdermatlas.com/photos/0000/2700/00002700_standalone.jpg
http://www.hellenicdermatlas.com/photos/0000/2701/00002701_standalone.jpg
http://www.hellenicdermatlas.com/photos/0000/2702/00002702_standalone.jpg
http://www.hellenicdermatlas.com/photos/0000/2703/00002703_standalone.jpg
http://www.hellenicdermatlas.com/photos/0000/2708/00002708_standalone.jpg
http://www.hellenicdermatlas.com/photos/0000/2709/00002709_standalone.jpg
http://www.hellenicdermatlas.com/photos/0000/2711/00002711_standalone.jpg
http://www.hellenicdermatlas.com/photos/0000/2712/00002712_standalone.jpg
http://www.hellenicdermatlas.com/photos/0000/2713/00002713_standalone.jpg
http://www.hellenicdermatlas.com/photos/0000/2714/00002714_standalone.jpg
http://www.hellenicdermatlas.com/photos/0000/2715/00002715_standalone.jpg
http://www.hellenicdermatlas.com/photos/0000/2716/00002716_standalone.jpg
http://www.hellenicdermatlas.com/photos/0000/2717/00002717_standalone.jpg
http://www.hellenicdermatlas.com/photos/0000/2718/00002718_standalone.jpg
http://www.hellenicdermatlas.com/photos/0000/2719/00002719_standalone.jpg
http://www.hellenicdermatlas.com/photos/0000/2720/00002720_standalone.jpg
http://www.hellenicdermatlas.com/photos/0000/2721/00002721_standalone.jpg
http://www.hellenicdermatlas.com/photos/0000/2722/00002722_standalone.jpg
http://www.hellenicdermatlas.com/photos/0000/2723/00002723_standalone.jpg
http://www.hellenicdermatlas.com/photos/0000/2724/00002724_standalone.jpg

http://www.hellenicdermatlas.com/photos/0000/2725/00002725_standalone.jpg
http://www.hellenicdermatlas.com/photos/0000/2726/00002726_standalone.jpg
http://www.hellenicdermatlas.com/photos/0000/2727/00002727_standalone.jpg
http://www.hellenicdermatlas.com/photos/0000/2728/00002728_standalone.jpg
http://www.hellenicdermatlas.com/photos/0000/2729/00002729_standalone.jpg
http://www.hellenicdermatlas.com/photos/0000/2730/00002730_standalone.jpg
http://www.hellenicdermatlas.com/photos/0000/2731/00002731_standalone.jpg
http://www.hellenicdermatlas.com/photos/0000/2732/00002732_standalone.jpg
http://www.hellenicdermatlas.com/photos/0000/2733/00002733_standalone.jpg
http://www.hellenicdermatlas.com/photos/0000/2734/00002734_standalone.jpg
http://www.hellenicdermatlas.com/photos/0000/2735/00002735_standalone.jpg
http://www.hellenicdermatlas.com/photos/0000/2736/00002736_standalone.jpg
http://www.hellenicdermatlas.com/photos/0000/2737/00002737_standalone.jpg
http://www.hellenicdermatlas.com/photos/0000/2738/00002738_standalone.jpg
http://www.hellenicdermatlas.com/photos/0000/2739/00002739_standalone.jpg
http://www.hellenicdermatlas.com/photos/0000/2741/00002741_standalone.jpg
http://www.hellenicdermatlas.com/photos/0000/2742/00002742_standalone.jpg
http://www.hellenicdermatlas.com/photos/0000/2743/00002743_standalone.jpg
http://www.hellenicdermatlas.com/photos/0000/2744/00002744_standalone.jpg
http://www.hellenicdermatlas.com/photos/0000/2745/00002745_standalone.jpg
http://www.hellenicdermatlas.com/photos/0000/2746/00002746_standalone.jpg
http://www.hellenicdermatlas.com/photos/0000/2747/00002747_standalone.jpg
http://www.hellenicdermatlas.com/photos/0000/2748/00002748_standalone.jpg
http://www.hellenicdermatlas.com/photos/0000/2749/00002749_standalone.jpg
http://www.hellenicdermatlas.com/photos/0000/2750/00002750_standalone.jpg
http://www.hellenicdermatlas.com/photos/0000/2751/00002751_standalone.jpg
http://www.hellenicdermatlas.com/photos/0000/2752/00002752_standalone.jpg
http://www.hellenicdermatlas.com/photos/0000/2753/00002753_standalone.jpg
http://www.hellenicdermatlas.com/photos/0000/2754/00002754_standalone.jpg
http://www.hellenicdermatlas.com/photos/0000/2755/00002755_standalone.jpg
http://www.hellenicdermatlas.com/photos/0000/2756/00002756_standalone.jpg
http://www.hellenicdermatlas.com/photos/0000/2757/00002757_standalone.jpg
http://www.hellenicdermatlas.com/photos/0000/2758/00002758_standalone.jpg
http://www.hellenicdermatlas.com/photos/0000/2759/00002759_standalone.jpg
http://www.hellenicdermatlas.com/photos/0000/2760/00002760_standalone.jpg
http://www.hellenicdermatlas.com/photos/0000/2761/00002761_standalone.jpg
http://www.hellenicdermatlas.com/photos/0000/2762/00002762_standalone.jpg
http://www.hellenicdermatlas.com/photos/0000/2763/00002763_standalone.jpg
http://www.hellenicdermatlas.com/photos/0000/2764/00002764_standalone.jpg
http://www.hellenicdermatlas.com/photos/0000/2765/00002765_standalone.jpg
http://www.hellenicdermatlas.com/photos/0000/2766/00002766_standalone.jpg
http://www.hellenicdermatlas.com/photos/0000/2767/00002767_standalone.jpg
http://www.hellenicdermatlas.com/photos/0000/2768/00002768_standalone.jpg
http://www.hellenicdermatlas.com/photos/0000/2769/00002769_standalone.jpg
http://www.hellenicdermatlas.com/photos/0000/2770/00002770_standalone.jpg
http://www.hellenicdermatlas.com/photos/0000/2771/00002771_standalone.jpg
http://www.hellenicdermatlas.com/photos/0000/2772/00002772_standalone.jpg
http://www.hellenicdermatlas.com/photos/0000/2773/00002773_standalone.jpg

http://www.hellenicdermatlas.com/photos/0000/2774/00002774_standalone.jpg
http://www.hellenicdermatlas.com/photos/0000/2775/00002775_standalone.jpg
http://www.hellenicdermatlas.com/photos/0000/2776/00002776_standalone.jpg
http://www.hellenicdermatlas.com/photos/0000/2777/00002777_standalone.jpg
http://www.hellenicdermatlas.com/photos/0000/2778/00002778_standalone.jpg
http://www.hellenicdermatlas.com/photos/0000/2779/00002779_standalone.jpg
http://www.hellenicdermatlas.com/photos/0000/2780/00002780_standalone.jpg
http://www.hellenicdermatlas.com/photos/0000/2781/00002781_standalone.jpg
http://www.hellenicdermatlas.com/photos/0000/2782/00002782_standalone.jpg
http://www.hellenicdermatlas.com/photos/0000/2783/00002783_standalone.jpg
http://www.hellenicdermatlas.com/photos/0000/2784/00002784_standalone.jpg
http://www.hellenicdermatlas.com/photos/0000/2785/00002785_standalone.jpg
http://www.hellenicdermatlas.com/photos/0000/2786/00002786_standalone.jpg
http://www.hellenicdermatlas.com/photos/0000/2787/00002787_standalone.jpg
http://www.hellenicdermatlas.com/photos/0000/2788/00002788_standalone.jpg
http://www.hellenicdermatlas.com/photos/0000/2789/00002789_standalone.jpg
http://www.hellenicdermatlas.com/photos/0000/2790/00002790_standalone.jpg
http://www.hellenicdermatlas.com/photos/0000/2791/00002791_standalone.jpg
http://www.hellenicdermatlas.com/photos/0000/2792/00002792_standalone.jpg
http://www.hellenicdermatlas.com/photos/0000/2793/00002793_standalone.jpg
http://www.hellenicdermatlas.com/photos/0000/2794/00002794_standalone.jpg
http://www.hellenicdermatlas.com/photos/0000/2795/00002795_standalone.jpg
http://www.hellenicdermatlas.com/photos/0000/2796/00002796_standalone.jpg
http://www.hellenicdermatlas.com/photos/0000/2797/00002797_standalone.jpg
http://www.hellenicdermatlas.com/photos/0000/2798/00002798_standalone.jpg
http://www.hellenicdermatlas.com/photos/0000/2799/00002799_standalone.jpg
http://www.hellenicdermatlas.com/photos/0000/2800/00002800_standalone.jpg
http://www.hellenicdermatlas.com/photos/0000/2801/00002801_standalone.jpg
http://www.hellenicdermatlas.com/photos/0000/2802/00002802_standalone.jpg
http://www.hellenicdermatlas.com/photos/0000/2803/00002803_standalone.jpg
http://www.hellenicdermatlas.com/photos/0000/2804/00002804_standalone.jpg
http://www.hellenicdermatlas.com/photos/0000/2805/00002805_standalone.jpg
http://www.hellenicdermatlas.com/photos/0000/2806/00002806_standalone.jpg
http://www.hellenicdermatlas.com/photos/0000/2807/00002807_standalone.jpg
http://www.hellenicdermatlas.com/photos/0000/2808/00002808_standalone.jpg
http://www.hellenicdermatlas.com/photos/0000/2809/00002809_standalone.jpg
http://www.hellenicdermatlas.com/photos/0000/2810/00002810_standalone.jpg
http://www.hellenicdermatlas.com/photos/0000/2811/00002811_standalone.jpg
http://www.hellenicdermatlas.com/photos/0000/2812/00002812_standalone.jpg
http://www.hellenicdermatlas.com/photos/0000/2813/00002813_standalone.jpg
http://www.hellenicdermatlas.com/photos/0000/2814/00002814_standalone.jpg
http://www.hellenicdermatlas.com/photos/0000/2815/00002815_standalone.jpg
http://www.hellenicdermatlas.com/photos/0000/2816/00002816_standalone.jpg
http://www.hellenicdermatlas.com/photos/0000/2817/00002817_standalone.jpg
http://www.hellenicdermatlas.com/photos/0000/2818/00002818_standalone.jpg
http://www.hellenicdermatlas.com/photos/0000/2819/00002819_standalone.jpg
http://www.hellenicdermatlas.com/photos/0000/2820/00002820_standalone.jpg
http://www.hellenicdermatlas.com/photos/0000/2821/00002821_standalone.jpg

http://www.hellenicdermatlas.com/photos/0000/2822/00002822_standalone.jpg
http://www.hellenicdermatlas.com/photos/0000/2823/00002823_standalone.jpg
http://www.hellenicdermatlas.com/photos/0000/2824/00002824_standalone.jpg
http://www.hellenicdermatlas.com/photos/0000/2825/00002825_standalone.jpg
http://www.hellenicdermatlas.com/photos/0000/2826/00002826_standalone.jpg
http://www.hellenicdermatlas.com/photos/0000/2827/00002827_standalone.jpg
http://www.hellenicdermatlas.com/photos/0000/2828/00002828_standalone.jpg
http://www.hellenicdermatlas.com/photos/0000/2829/00002829_standalone.jpg
http://www.hellenicdermatlas.com/photos/0000/2830/00002830_standalone.jpg
http://www.hellenicdermatlas.com/photos/0000/2831/00002831_standalone.jpg
http://www.hellenicdermatlas.com/photos/0000/2832/00002832_standalone.jpg
http://www.hellenicdermatlas.com/photos/0000/0017/00000017_standalone.jpg
http://www.hellenicdermatlas.com/photos/0000/1755/00001755_standalone.jpg
http://www.hellenicdermatlas.com/photos/0000/2155/00002155_standalone.jpg
http://www.hellenicdermatlas.com/photos/0000/2833/00002833_standalone.jpg
