# Supplementary material for: A deep-learning algorithm to classify skin lesions from mpox virus infection
Source: Nat Med. 2023 Mar 2;29(3):738–47. doi: 10.1038/s41591-023-02225-7 (PMC10033450; doi:10.1038/s41591-023-02225-7)
Supplement: Supplementary file 2 — Reporting Summary [file 41591_2023_2225_MOESM2_ESM.pdf]

nature portfolio

Corresponding author(s): Dr. Alexander Thieme

Last updated by author(s): Dec 23, 2022

Reporting Summary

Nature Portfolio wishes to improve the reproducibility of the work that we publish. This form provides structure for consistency and transparency in reporting. For further information on Nature Portfolio policies, see our Editorial Policies and the Editorial Policy Checklist.

Statistics

For all statistical analyses, confirm that the following items are present in the figure legend, table legend, main text, or Methods section.

n/a	Confirmed	
☐	☒	The exact sample size (*n*) for each experimental group/condition, given as a discrete number and unit of measurement
☐	☒	A statement on whether measurements were taken from distinct samples or whether the same sample was measured repeatedly
☒	☐	The statistical test(s) used AND whether they are one- or two-sided<br>*Only common tests should be described solely by name; describe more complex techniques in the Methods section.*
☐	☒	A description of all covariates tested
☐	☒	A description of any assumptions or corrections, such as tests of normality and adjustment for multiple comparisons
☐	☒	A full description of the statistical parameters including central tendency (e.g. means) or other basic estimates (e.g. regression coefficient) AND variation (e.g. standard deviation) or associated estimates of uncertainty (e.g. confidence intervals)
☒	☐	For null hypothesis testing, the test statistic (e.g. *F*, *t*, *r*) with confidence intervals, effect sizes, degrees of freedom and *P* value noted<br>*Give P values as exact values whenever suitable.*
☒	☐	For Bayesian analysis, information on the choice of priors and Markov chain Monte Carlo settings
☒	☐	For hierarchical and complex designs, identification of the appropriate level for tests and full reporting of outcomes
☒	☐	Estimates of effect sizes (e.g. Cohen's *d*, Pearson's *r*), indicating how they were calculated

Our web collection on statistics for biologists contains articles on many of the points above.

Software and code

Policy information about availability of computer code

Data collection	wget 1.21
Data analysis	The deep learning framework (FastAI v2) used in this study is available at https://www.fast.ai/. The pre-trained ResNet34 architecture used for the MPXV-CNN in this work is publicly available within the FastAI framework. The SHAP library used for explainability in this study is available at https://github.com/slundberg/shap. The code of PoxApp and the MPXV-CNN is available at https://github.com/PoxApp. The following packages were used which can be installed with the python package installer (pip): pytorch 1.12.0, fastai 2.7.7,scikit-image 0.19.3, python 3.7.13, torchvision 0.13.0, cudatoolkit 11.6.0, matplotlib 3.5.2. We used dupeGuru 4.31 to identify duplicate images which is available at https://dupeguru.voltaicideas.net/.

For manuscripts utilizing custom algorithms or software that are central to the research but not yet described in published literature, software must be made available to editors and reviewers. We strongly encourage code deposition in a community repository (e.g. GitHub). See the Nature Portfolio guidelines for submitting code & software for further information.

Data

Policy information about availability of data

All manuscripts must include a data availability statement. This statement should provide the following information, where applicable:

- Accession codes, unique identifiers, or web links for publicly available datasets
- A description of any restrictions on data availability
- For clinical datasets or third party data, please ensure that the statement adheres to our policy

Skin lesion images that were used for this study are available from:
Danderm: https://danderm-pdv.is.kkh.dk
DermIs: https://dermis.net
DermNet NZ: htttps://dermnetnz.org
DermNet: https://www.kaggle.com/datasets/shubhamgoel27/dermnet
Fitzpatrick 17k: github.com/mattgroh/fitzpatrick17k
HDA: https://hellenicdermatlas.com
PAD-uefs 20: data.mendeley.com/datasets/zr7vgbcyr2/1

Social media references are available upon request.

Images from the Stanford University Medical Center and the Esteva dataset are non-public and cannot be shared.

Human research participants

Policy information about studies involving human research participants and Sex and Gender in Research.

Reporting on sex and gender	For the prospective cohort, sex was defined as sex at birth self-reported by the patient. For other sources, sex was defined as reported in the textual information of the source. If no information on sex was reported, sex was assigned following evaluation of the image if sexual anatomy was visible. Sex-based analyses were performed in the testing cohort for non-monkeypox skin lesion images and not performed for monkeypox skin lesion images due to the scarcity of data for female individuals. Data from female individuals were included in the training cohort. Zoomed-in monkeypox skin lesion images not showing the anatomy of the patient had a high classification performance similar to zoomed-out images. Additionally, body regions without visible sexual anatomy such as the upper and lower extremeties had a high classification performance. Explanations by SHAP showed that the MPXV-CNN uses the part of the image that contains the monkeypox skin lesion and perilesional inflammation for prediction. There is no evidence that monkeypox skin lesion images have a different appearance for male and female patients. Therefore, our research findings apply to both sexes.
Population characteristics	All study participants of the prospective cohort were male, mostly between 20-40 yrs old, mostly living in the San Francisco Bay Area
Recruitment	Eligible participants of the prospective cohort were 18 and older and had a laboratory-confirmed infection with the monkeypox virus and received no prior treatment for monkeypox. Patients were recruited in the clinic of infectious diseases of the Stanford University Medical Center. Patients were recruited by a study physician when a test for monkeypox infection was pending or confirmed. The patient population at the Stanford University Medical Center might not be representative for the US population, which might introduce biases.
Ethics oversight	Stanford IRB approval was obtained for protocols 66980, 67068, and 36050. Written informed consent was obtained from patients before the initation of study procedures.

Note that full information on the approval of the study protocol must also be provided in the manuscript.

Field-specific reporting

Please select the one below that is the best fit for your research. If you are not sure, read the appropriate sections before making your selection.

☒ Life sciences ☐ Behavioural & social sciences ☐ Ecological, evolutionary & environmental sciences

For a reference copy of the document with all sections, see nature.com/documents/nr-reporting-summary-flat.pdf

Life sciences study design

All studies must disclose on these points even when the disclosure is negative.

Sample size	No sample size calculation was performed for the prospective cohort. Images of all patients with a confirmed monkeypox virus infection and consent were included. The number of participants was sufficient since the images were not used for training the MPXV-CNN, but as an external test cohort.

Data exclusions	The primary goal of the prospective image collection was to create an atlas of monkeypox skin lesion images. Therefore, patients with a negative test for the monkeypox virus were excluded. Furthermore, patients with prior treatment for monkeypox were excluded, since the treatment might change the appearance of the lesions. The exclusion criteria were pre-established.
Replication	The source code for MPXV-CNN model generation and evaluation was audited by multiple researchers of the study team. Results were obtained independently at least three times by multiple researchers and discrepancies were discussed with the entire study team. All attempts of the results reproduction were successful.
Randomization	Observational cohort study without randomization.
Blinding	Due to the study design (single arm prospective image collection), blinding was not possible.

Behavioural & social sciences study design

All studies must disclose on these points even when the disclosure is negative.

Study description	*Briefly describe the study type including whether data are quantitative, qualitative, or mixed-methods (e.g. qualitative cross-sectional, quantitative experimental, mixed-methods case study).*
Research sample	*State the research sample (e.g. Harvard university undergraduates, villagers in rural India) and provide relevant demographic information (e.g. age, sex) and indicate whether the sample is representative. Provide a rationale for the study sample chosen. For studies involving existing datasets, please describe the dataset and source.*
Sampling strategy	*Describe the sampling procedure (e.g. random, snowball, stratified, convenience). Describe the statistical methods that were used to predetermine sample size OR if no sample-size calculation was performed, describe how sample sizes were chosen and provide a rationale for why these sample sizes are sufficient. For qualitative data, please indicate whether data saturation was considered, and what criteria were used to decide that no further sampling was needed.*
Data collection	*Provide details about the data collection procedure, including the instruments or devices used to record the data (e.g. pen and paper, computer, eye tracker, video or audio equipment) whether anyone was present besides the participant(s) and the researcher, and whether the researcher was blind to experimental condition and/or the study hypothesis during data collection.*
Timing	*Indicate the start and stop dates of data collection. If there is a gap between collection periods, state the dates for each sample cohort.*
Data exclusions	*If no data were excluded from the analyses, state so OR if data were excluded, provide the exact number of exclusions and the rationale behind them, indicating whether exclusion criteria were pre-established.*
Non-participation	*State how many participants dropped out/declined participation and the reason(s) given OR provide response rate OR state that no participants dropped out/declined participation.*
Randomization	*If participants were not allocated into experimental groups, state so OR describe how participants were allocated to groups, and if allocation was not random, describe how covariates were controlled.*

Ecological, evolutionary & environmental sciences study design

All studies must disclose on these points even when the disclosure is negative.

Study description	*Briefly describe the study. For quantitative data include treatment factors and interactions, design structure (e.g. factorial, nested, hierarchical), nature and number of experimental units and replicates.*
Research sample	*Describe the research sample (e.g. a group of tagged Passer domesticus, all Stenocereus thurberi within Organ Pipe Cactus National Monument), and provide a rationale for the sample choice. When relevant, describe the organism taxa, source, sex, age range and any manipulations. State what population the sample is meant to represent when applicable. For studies involving existing datasets, describe the data and its source.*
Sampling strategy	*Note the sampling procedure. Describe the statistical methods that were used to predetermine sample size OR if no sample-size calculation was performed, describe how sample sizes were chosen and provide a rationale for why these sample sizes are sufficient.*
Data collection	*Describe the data collection procedure, including who recorded the data and how.*
Timing and spatial scale	*Indicate the start and stop dates of data collection, noting the frequency and periodicity of sampling and providing a rationale for these choices. If there is a gap between collection periods, state the dates for each sample cohort. Specify the spatial scale from which the data are taken*
Data exclusions	*If no data were excluded from the analyses, state so OR if data were excluded, describe the exclusions and the rationale behind them, indicating whether exclusion criteria were pre-established.*

Reproducibility	*Describe the measures taken to verify the reproducibility of experimental findings. For each experiment, note whether any attempts to repeat the experiment failed OR state that all attempts to repeat the experiment were successful.*
Randomization	*Describe how samples/organisms/participants were allocated into groups. If allocation was not random, describe how covariates were controlled. If this is not relevant to your study, explain why.*
Blinding	*Describe the extent of blinding used during data acquisition and analysis. If blinding was not possible, describe why OR explain why blinding was not relevant to your study.*

Did the study involve field work? ☐ Yes ☐ No

Field work, collection and transport

Field conditions	*Describe the study conditions for field work, providing relevant parameters (e.g. temperature, rainfall).*
Location	*State the location of the sampling or experiment, providing relevant parameters (e.g. latitude and longitude, elevation, water depth).*
Access & import/export	*Describe the efforts you have made to access habitats and to collect and import/export your samples in a responsible manner and in compliance with local, national and international laws, noting any permits that were obtained (give the name of the issuing authority, the date of issue, and any identifying information).*
Disturbance	*Describe any disturbance caused by the study and how it was minimized.*

Reporting for specific materials, systems and methods

We require information from authors about some types of materials, experimental systems and methods used in many studies. Here, indicate whether each material, system or method listed is relevant to your study. If you are not sure if a list item applies to your research, read the appropriate section before selecting a response.

Materials & experimental systems

n/a	Involved in the study
☒	☐ Antibodies
☒	☐ Eukaryotic cell lines
☒	☐ Palaeontology and archaeology
☒	☐ Animals and other organisms
☒	☐ Clinical data
☒	☐ Dual use research of concern

Methods

n/a	Involved in the study
☒	☐ ChIP-seq
☒	☐ Flow cytometry
☒	☐ MRI-based neuroimaging
